# Identification of potential drug targets for varicose veins: a Mendelian randomization analysis

**DOI:** 10.3389/fcvm.2023.1126208

**Published:** 2023-06-19

**Authors:** Jianfeng Lin, Jiawei Zhou, Zhili Liu, Rong Zeng, Lei Wang, Fangda Li, Liqiang Cui, Yuehong Zheng

**Affiliations:** ^1^ Chinese Academy of Medical Sciences and Peking Union Medical College, Beijing, China; ^2^ Department of Vascular Surgery, Peking Union Medical College Hospital, Chinese Academy of Medical Sciences and Peking Union Medical College, Beijing, China

**Keywords:** varicose veins, proteomics, genetics, drug targets, Mendelian randomization

## Abstract

**Introduction:**

Varicose veins are a common chronic disease that creates a significant economic burden on the healthcare system. Current treatment options, including pharmacological treatments, are not always effective, and there is a need for more targeted therapies. A Mendelian randomization (MR) method uses genetic variants as instrumental variables to estimate the causal effect of an exposure on an outcome, and it has been successful in identifying therapeutic targets in other diseases. However, few studies have used MR to explore potential protein drug targets for varicose veins.

**Methods:**

To identify potential drug targets for varicose veins of lower extremities, we undertook a comprehensive screen of plasma protein with a two-sample MR method. We used recently reported *cis*-variants as genetic instruments of 2,004 plasma proteins, then applied MR to a recent meta-analysis of genome-wide association study on varicose veins (22,037 cases and 437,665 controls). Furthermore, pleiotropy detection, reverse causality testing, colocalization analysis, and external replication were utilized to strengthen the causal effects of prioritized proteins. Phenome-wide MR (PheW-MR) of the prioritized proteins for the risk of 525 diseases was conducted to screen potential side effects.

**Results:**

We identified eight plasma proteins that are significantly associated with the risk of varicose veins after Bonferroni correction (*P* < 2.495 × 10^−5^), with five being protective (LUM, POSTN, RPN1, RSPO3, and VAT1) and three harmful (COLEC11, IRF3, and SARS2). Most identified proteins showed no pleiotropic effects except for COLLEC11. Bidirectional MR and MR Steiger testing excluded reverse causal relationship between varicose veins and prioritized proteins. The colocalization analysis indicated that COLEC11, IRF3, LUM, POSTN, RSPO3, and SARS2 shared the same causal variant with varicose veins. Finally, seven identified proteins replicated with alternative instruments except for VAT1. Furthermore, PheW-MR revealed that only IRF3 had potential harmful adverse side effects.

**Conclusions:**

We identified eight potential causal proteins for varicose veins with MR. A comprehensive analysis indicated that IRF3, LUM, POSTN, RSPO3, and SARS2 might be potential drug targets for varicose veins.

## 1. Introduction

Varicose veins are one of the most common chronic diseases. In the United States, about 23% of adults suffer from varicose veins, creating a tremendous economic burden on the healthcare system (1). Patients with varicose veins typically exhibited skin changes and lower-limb discomfort, including swelling, restlessness, and itching, and up to 20% of the patients eventually developed life-threatening ulceration (2, 3). The current treatment options for varicose veins include conservative management, such as compression stockings and lifestyle modifications, as well as more invasive procedures such as sclerotherapy, endovenous laser therapy, and surgery (4, 5). Despite these treatment options, there remains a need for more targeted and effective therapies. Pharmacological treatments targeting specific molecular pathways have shown promise in recent years, with some drugs focusing on improving venous tone and reducing inflammation, such as micronized purified flavonoid fraction (MPFF) and horse chestnut seed extract (HCSE) (6, 7). Despite the available treatment options for varicose veins, there is still a need for more targeted and effective therapies. Traditional methods of identifying potential drug targets can be challenging due to confounding and reverse causality, which can lead to inaccurate results (8). Therefore, alternative methods are needed to facilitate the identification of potential drug targets for varicose veins.

One such method is the Mendelian randomization (MR). At conception, single-nucleotide polymorphisms (SNPs) are randomly assigned and not influenced by environmental factors, making them ideal instruments for causal inference. MR is a form of instrumental variable analysis that uses mainly SNPs as genetic instruments to estimate the causal effect of an exposure (in this case, circulating protein) on an outcome (varicose veins) (9). When conducting MR, SNPs that are significantly associated with the exposure of interest must be identified and used as the instrument variables. When multiple SNPs are available for a given exposure, the effects estimated from each single SNP could be synthesized by various algorithms, such as the inverse variance-weighted (IVW) method (10) and the Egger regression method (11). By using genetic variants as instrumental variables, MR can bypass the influence of confounding and reverse causality, providing more reliable estimates of causal effects than the traditional observational studies (12). MR has been used successfully in previous studies to identify biomarkers and therapeutic targets in a wide range of diseases, including stroke (13), multiple sclerosis (14), and type 1 diabetes (15). However, few studies established the causation of circulating protein on varicose veins based on MR.

**Figure 1:**
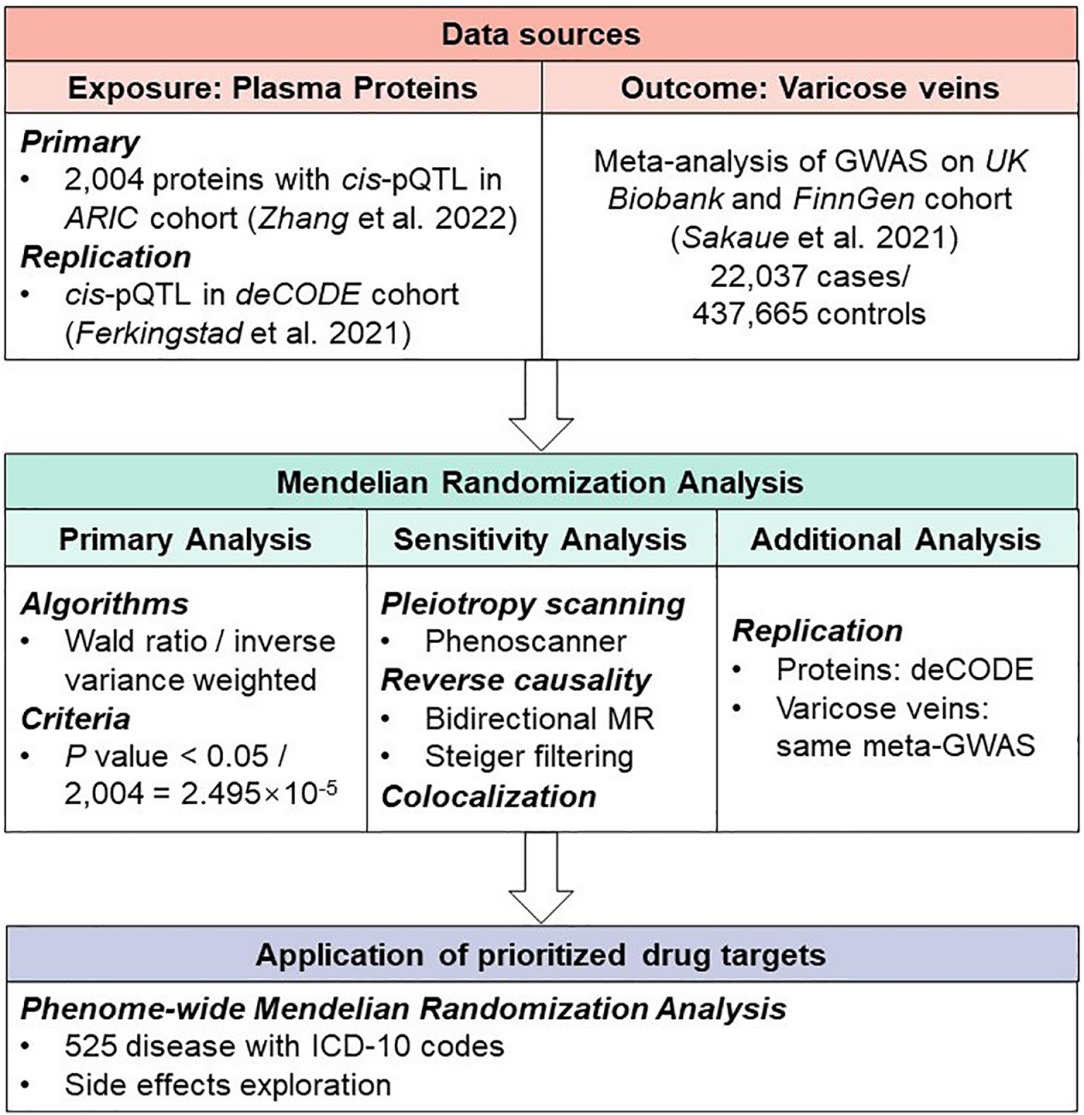
Study design of Mendelian Randomization study to reveal potential drug targets for varicose veins.

The present study aimed to identify plasma proteins that could influence the risk of varicose veins of lower extremities. We utilized MR to systematically screen plasma proteins of proteomic data to identify the causal proteins of varicose veins. The primary results were further validated by colocalization and external replication to identify potential drug targets. Finally, we conducted a phenome-wide MR analysis on 525 disorders to predict potential side effects of identified drug targets (Figure 1).

## 2. Methods

### 2.1. Selection of genetic instruments for proteins

We selected SNPs as genetic instruments for plasma proteins (pQTLs) from a recent study conducted by Zhang et al. (16), which reported 2,004 proteins that showed associations with common variants in 7,213 European American populations. First, to satisfy the relevance assumption of MR, we selected SNPs that are strongly (*P* < 5 × 10^−8^, *F* statistics >10) and independently (clumping threshold: LD *r*^2^ < 0.001) associated with the protein (17, 18). Second, according to the independence assumption, the genetic instruments should not be associated with factors confounding the relationship between the exposure and the outcome. Using mixed populations of various ancestries in MR might violate the independence assumption, which is reduced by ensuring that all the included genome-wide association studies (GWASs) were conducted in people of European origin (19). The third MR assumption, known as the exclusion restriction assumption, requires a null association between the genetic instrument and outcome unless through its impact on the exposure. Horizontal pleiotropy of SNP might lead to violation of exclusion restriction assumption (20). Hence, only cis-acting SNPs (cis-pQTL) were used for following analysis. We then computed the proportion of the variance explained (PVE) of the respective protein according to the formula: PVE = beta^2^/(beta^2 ^+ N × se^2^) (21) (Supplementary Table S1).

We also used pQTL determined in the deCODE cohort by Ferkingstad et al. (22), which reported 4,907 plasma proteins measured in 35,559 European participants for replication. We included only the instruments for prioritized proteins that were identified by primary analysis, and the inclusion criteria were the same as above (Supplementary Table S2).

### 2.2. Data sources of varicose veins GWAS

We extracted the genetic instruments for varicose veins from the meta-analysis of GWASs conducted in two European cohorts: the UK Biobank and FinnGen Cohort (release 3). The details of both original GWASs and the meta-analysis have been discussed previously (23). Briefly, 22,037 participants with varicose veins and 437,665 controls were enrolled. In both cohorts, the varicose veins were recorded by the International Classification of Diseases 10 (ICD-10) diagnosis code I83: Varicose veins of lower extremities. Then the inverse variance-weighted meta-analysis was conducted for all variants passed quality control with METAL software (v.2011-03-25).

### 2.3. Statistical analysis

In the initial MR analysis, proteins were regarded as the exposures and varicose veins as the outcome. When only one pQTL was available, the Wald ratio algorithm was used, while the inverse variance-weighted (MR-IVW) was applied when two or more instruments were available (24). We selected a *P*-value threshold of 0.05, corrected for the number of independent tests, as our threshold for prioritizing MR results for follow-up analyses (*P *< 0.05/2,004 = 2.495 × 10^−5^). For replication, we set *P *< 0.05 as the criteria for successfully replicated. Odds ratios were expressed per standard deviation (SD) increase in genetically determined plasma protein levels. All Mendelian randomization analyses were conducted with “TwoSampleMR” (https://github.com/MRCIEU/TwoSampleMR).

Several sensitivity analyses were then employed to assess the validity of primary MR findings. First, we used “phenoscanner” (https://github.com/phenoscanner/phenoscanner) to determine any pleiotropy of SNPs used in primary analysis. Specifically, phenoscanner searched previous GWASs to identify the reported SNP-traits association. We considered an SNP as pleiotropic when the reported SNP-traits association was genome-wide significant (*P* < 5 × 10**^−^**^8^) in European population. Second, to detect reverse causality between varicose veins and plasma proteins, we conducted both bidirectional MR analysis (25) and MR Steiger filtering to orient the causal relationship (26). For bidirectional MR, genetic instruments for varicose veins were extracted from the meta-analysis of GWASs mentioned above, and the same inclusion criteria for pQTL were also applied (Supplementary Table S3), while summary statistics for proteins were obtained from deCODE cohorts (22) since full summary statistics were not available in Zhang et al. study. Reverse causality was confirmed when the bidirectional MR reached significance (*P* < 0.05) or MR Steiger filtering failed (*P* > 0.05 and direction is wrong). Finally, colocalization analysis was completed to further strengthen the reliability of causal inference. We used a stringent Bayesian model to estimate the posterior probability of that the genetic associations with both protein and phenotype shared the same causal variant (posterior probability of hypothesis 4, PPH_4_) (27). The PPH_4_ > 80% supported the hypothesis that plasma protein and varicose veins were causally related by the same variant.

### 2.4. Phenome-wide MR analysis

To further explore the potential side effects associated with hypothetical interventions that reduce the risk of varicose veins by targeting identified potential drug targets, we performed an agnostic phenome-wide MR (PheW-MR) analysis. While MR can identify causal relationships between an exposure and an outcome, it is important to consider the potential unintended effects of targeting a protein for therapeutic intervention. The PheW-MR method provides a way to assess the potential side effects of reducing the levels of proteins that were identified as potential drug targets in the primary MR analysis on a wide range of disease outcomes (13). The PheW-MR used the same genetic instruments of prioritized proteins as primary analysis. We selected genetic instruments for disease traits from IEU Open GWAS (PheW-MR). The IEU Open GWAS contained more than 40,000 GWASs, and we included GWASs conducted in UK Biobank with disease traits defined by ICD-10 diagnosis code (Supplementary Table S4). Then, Mendelian randomization was conducted with exposure as protein and outcome as any of the disease traits. The PheW-MR findings were standardized to a change in protein level corresponding to a 10% reduction in the risk of varicose veins to identify potential side effects when proteins are therapeutically targeted for varicose veins.

## 3. Results

### 3.1. Screening the proteome for varicose veins causal proteins

**Figure 2:**
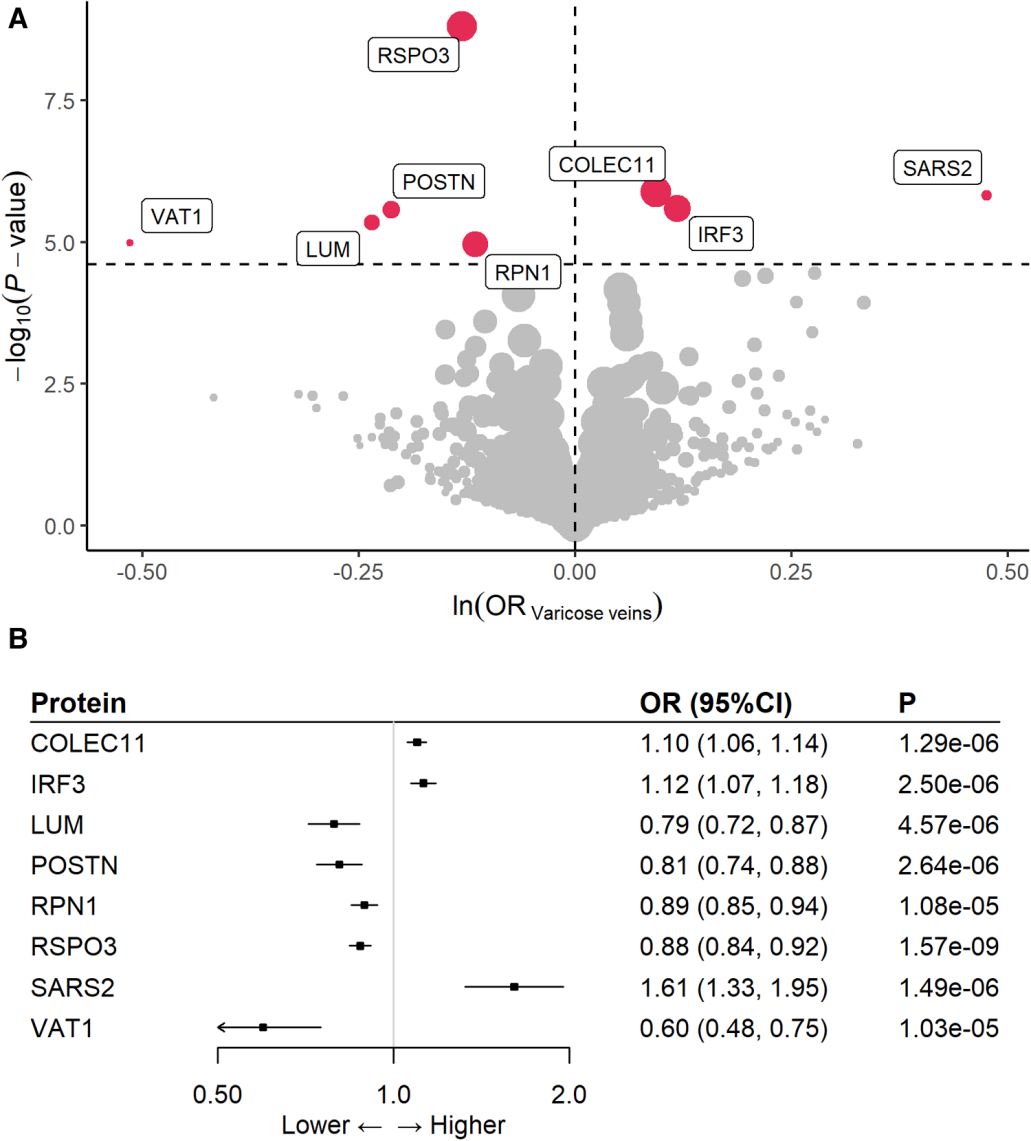
Effects of eight proteins with risk of varicose veins. (A) volcano plot; (B) forest plot.

At Bonferroni significance (*P* < 2.495 × 10^−5^), MR analysis revealed eight varicose veins-related proteins, including three deleterious proteins for varicose vein as collectin-11 (COLEC11), interferon regulatory factor 3 (IRF3), and mitochondrial serine–tRNA ligase (SARS2), and five protective proteins as lumican (LUM), periostin (POSTN), dolichyl-diphosphooligosaccharide–protein glycosyltransferase subunit 1 (RPN1), R-spondin-3 (RSPO3), and synaptic vesicle membrane protein VAT-1 homolog (VAT1) (Table 1, Figure 2A). In detail, genetically increased COLEC11 (OR = 1.10; 95% CI, 1.06–1.14; *P* = 1.29 × 10^−6^), IRF3 (OR = 1.12; 95% CI, 1.07–1.18; *P* = 2.50 × 10^−6^), and SARS2 (OR = 1.61; 95% CI, 1.33–1.95; *P* = 1.49 × 10^−6^) were associated with an increased risk of varicose veins, while LUM (OR = 0.79; 95% CI, 0.72–0.87; *P* = 4.57 × 10^−6^), POSTN (OR = 0.81; 95% CI, 0.74–0.88; *P* = 2.64 × 10^−6^), RPN1 (OR = 0.89; 95% CI, 0.85–0.94; *P* = 1.08 × 10^−5^), RSPO3 (OR = 0.88; 95% CI, 0.84–0.92; *P* = 1.57 × 10^−9^), and VAT1 (OR = 0.60; 95% CI, 0.48–0.75; *P* = 1.03 × 10^−5^) could decrease the risk of varicose veins (Figure 2B).

**Table 1:** Summary of pleiotropy scanning, reverse causality detection, and Bayesian colocalization analysis on the eight prioritized proteins.

Protein	Protein full name	UniProt ID	SNP	Previously reported associations	Bidirectional MR (95% CI)^a^	Steiger filtering	Colocalization PPH_4_^b^
COLEC11	Collectin-11	Q9BWP8	rs6542680	Impedance, various proteins	0.990 (0.963–1.019)	Passed (2.33 × 10^−206^)	0.996
IRF3	Interferon regulatory factor 3	Q14653	rs10415576	N/A	0.986 (0.957–1.016)	Passed (3.84 × 10^−147^)	0.993
LUM	Lumican	P51884	rs3741835	N/A	1.003 (0.972–1.035)	Passed (1.16 × 10^−29^)	0.954
POSTN	Periostin	Q15063	rs7329947	N/A	0.986 (0.956–1.017)	Passed (4.59 × 10^−41^)	0.819
RPN1	Dolichyl-diphosphooligosaccharide–protein glycosyltransferase subunit 1	P04843	rs9880064	Blood cells	0.992 (0.962–1.023)	Passed (8.6 × 10^−126^)	2.37 × 10^−8^
RSPO3	R-spondin-3	Q9BXY4	rs1892172	Waist circumference, Hip circumference, WHR, fracture, RBC	0.964 (0.867–1.072)	Passed (1.12 × 10^−188^)	0.904
SARS2	Serine–tRNA ligase, mitochondrial	Q9NP81	rs1808661	N/A	0.991 (0.964–1.018)	Passed (2.19 × 10^−9^)	0.970
VAT1	Synaptic vesicle membrane protein VAT-1 homolog	Q99536	rs4239148	Age at menopause	0.998 (0.972–1.026)	Passed (1.15 × 10^−6^)	0.613

^a^ Odds ratio and 95% confidence interval were estimated with the inverse variance-weighted method.

^b^ SNP associated with traits mediated by its proxy.

CI, confidence level; MR, Mendelian randomization; N/A, not available; PPH_4_, posterior probability of hypothesis 4; RBC, red blood cell; SNP, single-nucleotide polymorphism; WHR, waist–hip ratio.

### 3.2. Sensitivity analysis for varicose veins causal proteins

We assessed the stability of our findings in a series of sensitivity analyses. Five of eight proteins in primary analysis were finally identified as potential drug targets for varicose veins, including IRF3, LUM, POSTN, RSPO3, and SARS2. First, pleiotropy scanning with phenoscanner revealed previously reported associations of genetic instruments for COLEC11, RPN1, RSPO3, VAT1, and COLEC11 was excluded as potential drug target since the genetic instrument for COLEC11 was associated with various proteins (Table 1, Supplementary Table S5). Second, bidirectional MR analysis did not reveal any causal effect of varicose veins on the eight prioritized proteins, and Steiger filtering also ensured the directionality (Table 1, Supplementary Table S6 , Figure S1). Third, Bayesian colocalization was utilized to test whether the proteins and varicose veins shared the same variant. Six proteins passed the test, including COLEC11 (PPH_4_ = 0.996), IRF3 (PPH_4_ = 0.993), LUM (PPH_4_ = 0.954), POSTN (PPH_4_ = 0.819), RSPO3 (PPH_4_ = 0.904), and SARS2 (PPH_4_ = 0.970) (Table 1, Supplementary Figures S2–S9).

### 3.3. PheW-MR analysis of the side effects of varicose veins causal proteins

**Figure 3:**
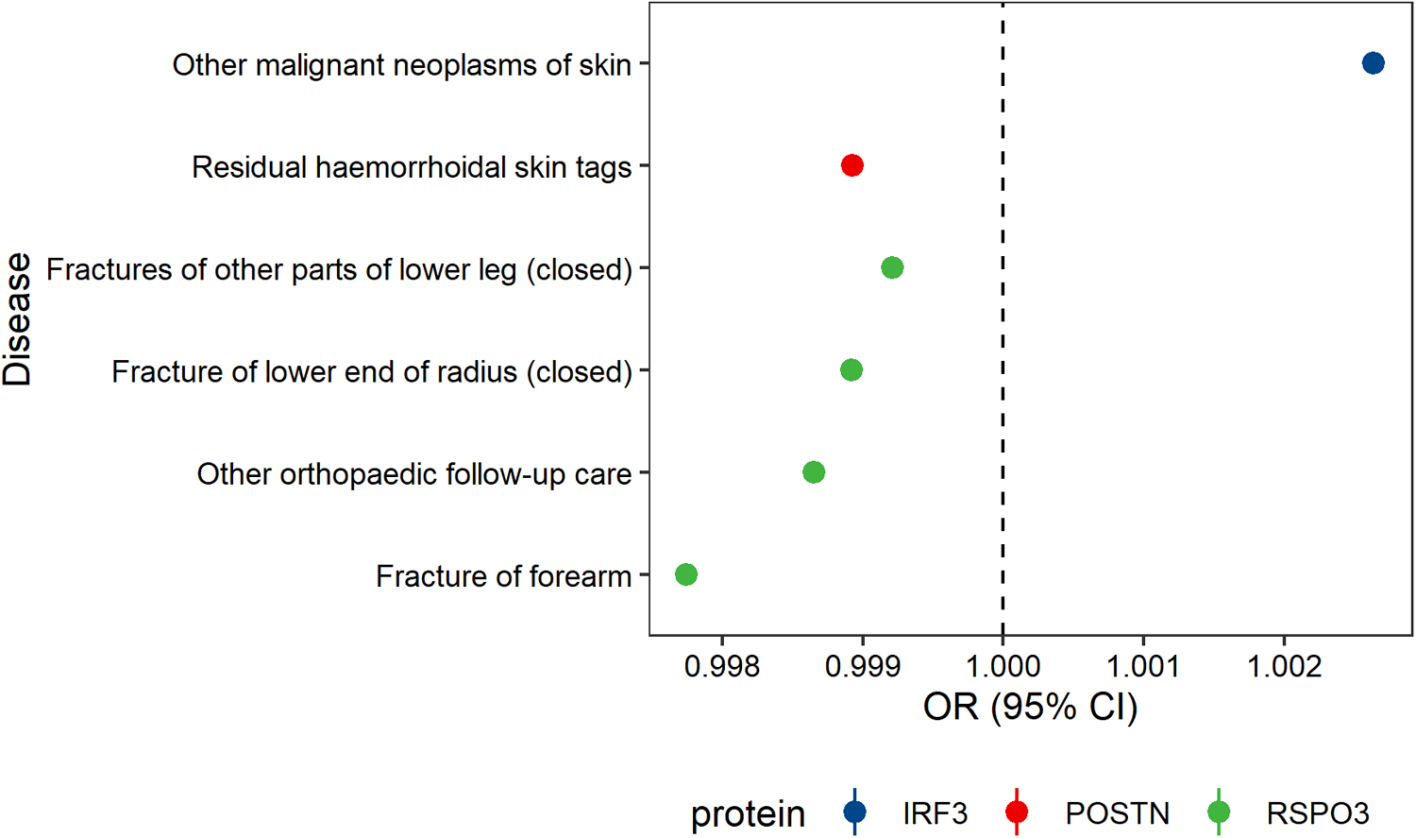
Potential on-target side effects associated with eight prioritized interventions.

Phenome-wide Mendelian randomization on 525 disease traits revealed that IRF3 and POSTN were associated with skin disorders, such as malignant neoplasm of skin and residual hemorrhoidal skin tags. In contrast, genetically increased level of RSPO3 was causally associated with a reduced risk of both varicose veins and certain fractures (Figure 3, Supplementary Tables S7–S14). No significant association was observed for the other five potential proteins.

### 3.4. External replication of varicose veins causal proteins

By substituting the data source of prioritized proteins, seven potential drug targets were replicated except VAT1 due to the absence of data: increasing COLEC11 (OR = 1.07; 95% CI, 1.04–1.10; *P* = 1.29 × 10^−6^), IRF3 (OR = 2.14; 95% CI, 1.56–1.10; *P* = 2.50 × 10^−6^), and SARS2 (OR = 1.65; 95% CI, 1.35–2.01; *P* = 7.23 × 10^−7^) could increase the risk of varicose veins while LUM (OR = 0.69; 95% CI, 0.59–0.80; *P* = 2.33 × 10^−6^), POSTN (OR = 0.73; 95% CI, 0.64–0.83; *P* = 1.73 × 10^−6^), RPN1 (OR = 0.72; 95% CI, 0.63–0.84; *P* = 1.04 × 10^−5^), and RSPO3 (OR = 0.77; 95% CI, 0.70–0.84; *P* = 8.56 × 10^−9^) showed a protective causal effect on varicose veins (Supplementary Figure S10).

## 4. Discussion

To date, we conducted the first study that systematically screens plasma proteins for varicose veins utilizing Mendelian randomization. As a result, eight proteins were found to be causally associated with varicose veins, including COLEC11, IRF3, LUM, POSTN, RPN1, RSPO3, SARS2, and VAT1. In addition, IRF3, LUM, POSTN, RSPO3, and SARS2 were further validated as potential drug targets by pleiotropy scanning, reverse causality detection, and colocalization analysis, which were further replicated.

Our study performed widely used MR to explore potential proteins for varicose veins, which was a common method for clinical translation of GWAS studies (28). Still, MR cannot entirely exclude the bias introduced by reverse causality, horizontal pleiotropy, and linkage disequilibrium. Therefore, we conducted a series of sensitivity analyses to test the MR assumptions. We used the bidirectional MR and Steiger filtering to orient the causal direction between proteins and varicose veins (26). We did not identify strong evidence for the reverse effect of varicose veins on surveyed proteins (29). To control the bias from horizontal pleiotropy, we restricted the instruments to cis-pQTLs (30). Besides, Bayesian colocalization was conducted to exclude the bias of linkage disequilibrium with 0.8 as the critical threshold for posterior probability. The COLEC11, IRF3, LUM, POSTN, RSPO3, and SARS2 were considered to share the same variant of varicose veins (27). When it comes to pleiotropy, genetic instruments for COLEC11, RPN1, RSPO3, and VAT1 were associated with traits beyond plasma proteins via phenotype scanning. The SNP rs6542680 as genetic instrument of COLEC11 seemed to be associated with various proteins in a trans-manner. COLEC11 might act as a hub regulating expression of multiple proteins, so the role of COLEC11 in varicose veins should be carefully interpreted. However, none of the other identified pleiotropy could completely explain the association with varicose veins, including RPN1, RSPO3, and VAT1.

Finally, we further conducted PheW-MR to explore potential side effects when proteins-targeted therapies were applied to varicose veins. Overall, per 10% reduction in the risk of varicose veins, we only found that IRF3 usage might be harmful to induce malignant neoplasms of skin, which has no relationship with varicose veins formation to date. Therefore, IRF3, LUM, POSTN, RSPO3, and SARS2 might be potential drug targets for varicose veins.

Varicose vein, characterized by a loss of vessel wall homeostasis, was considered related to degradation of extracellular matrix (ECM), activation of the endothelium, and apoptosis of smooth muscle cells (31, 32). Lumican, encoded by LUM, belonged to the family of small leucine-rich proteoglycans, which is an important part of non-collagenous ECM proteins (33). One research group explored the differences in the ECM composition between normal saphenous veins and varicose saphenous veins in which significant lower expression of lumican in diseased tissue (34). This proteoglycan could help maintain appropriate caliber, shape, and disposition of collagen fibers due to its horseshoe shapes (35). Therefore, we speculated that lumican could prevent the occurrence of varicose veins by its maintenance role in ECM structure. Furthermore, RSPO3 was the WNT signaling enhancer predominantly expressed within the vasculature (36). In RSPO3-deficient mice, micro-vessel density was reduced due to endothelial cell apoptosis and vascular pruning, indicating a crucial critical role in the vascular remodeling of endothelial RSPO3/WNT/Ca^2+^/NFAT signaling pathways (37). A recent GWAS identified RSPO3 as a risk locus for varicose veins, which was in consistent with our finding (38).

Of the three remaining proteins, IRF3 and POSTN were related to dysfunction of vascular smooth muscle cells (VSMCs). IRF3 is an important transcriptional regulator of the antiviral immune response (39). One study found that C-reactive protein could stimulate IL-6 production and inhibit peroxisome proliferator-activated receptor *γ* (PPAR*γ*) expression, a negative regulator of inflammatory responses, in rat VSMCs via the TLR4/IRF3/NF-*κ*B signaling pathway (40, 41). IRF3 might increase the risk of varicose veins through inflammation, which is consistent with our findings (42). Moreover, we identified another protein related to the function of VSMCs, POSTN. Periostin is a heparin-binding N-glycosylated protein mediating cell adhesion (43). Further research revealed its role in vascular cell differentiation and migration during the repair of vascular injury. *In vitro*, periostin expression was proven to be associated with smooth muscle cell differentiation and cell migration, which might explain the protective role in varicose veins (44).

Lastly, SARS2 encodes mitochondrial seryl-tRNA synthetase with the primary function of charging tRNASer with aminoacylated serine, which further participates in mitochondrial protein synthesis (45). In other words, SARS2 is related to the normal function of the mitochondrial respiratory chain and energy conversion (46). In our study, we identified its protective role in varicose veins, while the evidence is limited. Further research is needed to clarify the mechanisms.

## 5. Limitation

First, we used genetic data from the Britain and Finland, and it was known that the genetic data from the Finnish population differs from other European populations given their genetic makeup (47). Second, although we explored the association between the prioritized proteins and 525 disease traits, more side effects might be neglected since the 525 disorders only covered a small range of clinical conditions. Lastly, we did not explore the pharmaceutical mechanisms of identified proteins and did not investigate the predictive value of previous markers in external longitudinal case-control cohorts. Further studies and clinical trials are warranted to justify the feasibility of our findings.

## 6. Conclusion

To sum up, the present MR analysis of plasma proteome identified eight plasma proteins causally related to the varicose vein. Five proteins finally passed the sensitivity analyses and external replication including IRF3, LUM, POSTN, RSPO3, and SARS2, which might be more valuable in the clinical application. Hopefully, our study could predict a few meaningful drug targets for varicose veins.

## Data Availability

The data sets presented in this study can be found in online repositories. The names of the repository/repositories and accession number(s) can be found in the article/Supplementary Material.

## References

[1] Hamdan A. Management of varicose veins and venous insufficiency. JAMA. (2012) 308(24):2612–21. 10.1001/jama.2012.111352 23268520

[2] McGuckin M Waterman R Brooks J Cherry G Porten L Hurley S Validation of venous leg ulcer guidelines in the United States and United Kingdom. Am J Surg. (2002) 183(2):132–7. 10.1016/S0002-9610(01)00856-X 11918875

[3] Beebe-Dimmer JL Pfeifer JR Engle JS Schottenfeld D. The epidemiology of chronic venous insufficiency and varicose veins. Ann Epidemiol. (2005) 15(3):175–84. 10.1016/j.annepidem.2004.05.015 15723761

[4] Gloviczki P Comerota AJ Dalsing MC Eklof BG Gillespie DL Gloviczki ML The care of patients with varicose veins and associated chronic venous diseases: clinical practice guidelines of the society for vascular surgery and the American Venous Forum. J Vasc Surg. (2011) 53(5 Suppl):2s–48s. 10.1016/j.jvs.2011.01.079 21536172

[5] Eberhardt RT Raffetto JD. Chronic venous insufficiency. Circulation. (2014) 130(4):333–46. 10.1161/CIRCULATIONAHA.113.006898 25047584

[6] Kakkos SK Nicolaides AN. Efficacy of micronized purified flavonoid fraction (Daflon®) on improving individual symptoms, signs and quality of life in patients with chronic venous disease: a systematic review and meta-analysis of randomized double-blind placebo-controlled trials. Int Angiol. (2018) 37(2):143–54. 10.23736/S0392-9590.18.03975-5 29385792

[7] Pittler MH Ernst E. Horse chestnut seed extract for chronic venous insufficiency. Cochrane Database Syst Rev. (2012) 11(11):CD003230. 10.1002/14651858.CD003230.pub4 15106197

[8] Hu Y Zhao T Zhang N Zhang Y Cheng L. A review of recent advances and research on drug target identification methods. Curr Drug Metab. (2019) 20(3):209–16. 10.2174/1389200219666180925091851 30251599

[9] Holmes MV Ala-Korpela M Smith GD. Mendelian randomization in cardiometabolic disease: challenges in evaluating causality. Nat Rev Cardiol. (2017) 14(10):577–90. 10.1038/nrcardio.2017.78 28569269 PMC5600813

[10] Burgess S Butterworth A Thompson SG. Mendelian randomization analysis with multiple genetic variants using summarized data. Genet Epidemiol. (2013) 37(7):658–65. 10.1002/gepi.21758 24114802 PMC4377079

[11] Bowden J Davey Smith G Burgess S. Mendelian randomization with invalid instruments: effect estimation and bias detection through Egger regression. Int J Epidemiol. (2015) 44(2):512–25. 10.1093/ije/dyv080 26050253 PMC4469799

[12] Sanderson E Glymour MM Holmes MV Kang H Morrison J Munafò MR Mendelian randomization. Nat Rev Methods Primers. (2022) 2(1):6. 10.1038/s43586-021-00092-5 37325194 PMC7614635

[13] Chong M Sjaarda J Pigeyre M Mohammadi-Shemirani P Lali R Shoamanesh A Novel drug targets for ischemic stroke identified through Mendelian randomization analysis of the blood proteome. Circulation. (2019) 140(10):819–30. 10.1161/CIRCULATIONAHA.119.040180 31208196

[14] Lin J Zhou J Xu Y. Potential drug targets for multiple sclerosis identified through Mendelian randomization analysis. Brain. (2023):awad070. 10.1093/brain/awad070. [ahead of print] PMC10393411 36864689

[15] Yazdanpanah N Yazdanpanah M Wang Y Forgetta V Pollak M Polychronakos C Clinically relevant circulating protein biomarkers for type 1 diabetes: evidence from a two-sample Mendelian randomization study. Diabetes Care. (2022) 45(1):169–77. 10.2337/dc21-1049 34758976

[16] Zhang J Dutta D Köttgen A Tin A Schlosser P Grams ME Plasma proteome analyses in individuals of European and African ancestry identify cis-pQTLs and models for proteome-wide association studies. Nat Genet. (2022) 54(5):593–602. 10.1038/s41588-022-01051-w 35501419 PMC9236177

[17] Pierce BL Ahsan H Vanderweele TJ. Power and instrument strength requirements for Mendelian randomization studies using multiple genetic variants. Int J Epidemiol. (2011) 40(3):740–52. 10.1093/ije/dyq151 20813862 PMC3147064

[18] Davies NM Holmes MV Davey Smith G. Reading Mendelian randomisation studies: a guide, glossary, and checklist for clinicians. Br Med J. (2018) 362:k601. 10.1136/bmj.k601 30002074 PMC6041728

[19] Price AL Patterson NJ Plenge RM Weinblatt ME Shadick NA Reich D. Principal components analysis corrects for stratification in genome-wide association studies. Nat Genet. (2006) 38(8):904–9. 10.1038/ng1847 16862161

[20] Swerdlow DI Kuchenbaecker KB Shah S Sofat R Holmes MV White J Selecting instruments for Mendelian randomization in the wake of genome-wide association studies. Int J Epidemiol. (2016) 45(5):1600–16. 10.1093/ije/dyw088 27342221 PMC5100611

[21] Shim H Chasman DI Smith JD Mora S Ridker PM Nickerson DA A multivariate genome-wide association analysis of 10 LDL subfractions, and their response to statin treatment, in 1868 Caucasians. PLoS One. (2015) 10(4):e0120758. 10.1371/journal.pone.0120758 25898129 PMC4405269

[22] Ferkingstad E Sulem P Atlason BA Sveinbjornsson G Magnusson MI Styrmisdottir EL Large-scale integration of the plasma proteome with genetics and disease. Nat Genet. (2021) 53(12):1712–21. 10.1038/s41588-021-00978-w 34857953

[23] Sakaue S Kanai M Tanigawa Y Karjalainen J Kurki M Koshiba S A cross-population atlas of genetic associations for 220 human phenotypes. Nat Genet. (2021) 53(10):1415–24. 10.1038/s41588-021-00931-x 34594039 PMC12208603

[24] Deng YT Ou YN Wu BS Yang YX Jiang Y Huang YY Identifying causal genes for depression via integration of the proteome and transcriptome from brain and blood. Mol Psychiatry. (2022) 27(6):2849–57. 10.1038/s41380-022-01507-9 35296807

[25] Davey Smith G Hemani G. Mendelian randomization: genetic anchors for causal inference in epidemiological studies. Hum Mol Genet. (2014) 23(R1):R89–98. 10.1093/hmg/ddu328 25064373 PMC4170722

[26] Hemani G Tilling K Davey Smith G. Orienting the causal relationship between imprecisely measured traits using GWAS summary data. PLoS Genet. (2017) 13(11):e1007081. 10.1371/journal.pgen.1007081 29149188 PMC5711033

[27] Giambartolomei C Vukcevic D Schadt EE Franke L Hingorani AD Wallace C Bayesian test for colocalisation between pairs of genetic association studies using summary statistics. PLoS Genet. (2014) 10(5):e1004383. 10.1371/journal.pgen.1004383 24830394 PMC4022491

[28] McGowan LM Davey Smith G Gaunt TR Richardson TG. Integrating Mendelian randomization and multiple-trait colocalization to uncover cell-specific inflammatory drivers of autoimmune and atopic disease. Hum Mol Genet. (2019) 28(19):3293–300. 10.1093/hmg/ddz155 31276585 PMC6859431

[29] Lutz SM Wu AC Hokanson JE Vansteelandt S Lange C. Caution against examining the role of reverse causality in Mendelian randomization. Genet Epidemiol. (2021) 45(5):445–54. 10.1002/gepi.22385 34008876 PMC8222166

[30] Montgomery SB Dermitzakis ET. From expression QTLs to personalized transcriptomics. Nat Rev Genet. (2011) 12(4):277–82. 10.1038/nrg2969 21386863

[31] Lim CS Davies AH. Pathogenesis of primary varicose veins. Br J Surg. (2009) 96(11):1231–42. 10.1002/bjs.6798 19847861

[32] Chang M-Y Chiang P-T Chung Y-C Ho S-Y Lin S-D Lin S-R Apoptosis and angiogenesis in varicose veins using gene expression profiling. Fooyin J Health Sci. (2009) 1(2):85–91. 10.1016/S1877-8607(10)60005-7

[33] Nikitovic D Katonis P Tsatsakis A Karamanos NK Tzanakakis GN. Lumican, a small leucine-rich proteoglycan. IUBMB Life. (2008) 60(12):818–23. 10.1002/iub.131 18949819

[34] Barallobre-Barreiro J Oklu R Lynch M Fava M Baig F Yin X Extracellular matrix remodelling in response to venous hypertension: proteomics of human varicose veins. Cardiovasc Res. (2016) 110(3):419–30. 10.1093/cvr/cvw075 27068509 PMC4872879

[35] Chen S Birk DE. The regulatory roles of small leucine-rich proteoglycans in extracellular matrix assembly. FEBS J. (2013) 280(10):2120–37. 10.1111/febs.12136 23331954 PMC3651807

[36] de Lau WB Snel B Clevers HC. The R-spondin protein family. Genome Biol. (2012) 13(3):242. 10.1186/gb-2012-13-3-242 22439850 PMC3439965

[37] Scholz B Korn C Wojtarowicz J Mogler C Augustin I Boutros M Endothelial RSPO3 controls vascular stability and pruning through non-canonical WNT/Ca(2+)/NFAT signaling. Dev Cell. (2016) 36(1):79–93. 10.1016/j.devcel.2015.12.015 26766444

[38] Baylis RA Smith NL Klarin D Fukaya E. Epidemiology and genetics of venous thromboembolism and chronic venous disease. Circ Res. (2021) 128(12):1988–2002. 10.1161/CIRCRESAHA.121.318322 34110897 PMC8487638

[39] Doyle SE Vaidya SA O'Connell R Dadgostar H Dempsey PW Wu T-T IRF3 mediates a TLR3/TLR4-specific antiviral gene program. Immunity. (2002) 17(3):251–63. 10.1016/S1074-7613(02)00390-4 12354379

[40] Liu N Liu J-T Ji Y-Y Lu P-P. C-reactive protein triggers inflammatory responses partly via TLR4/IRF3/NF-*κ*B signaling pathway in rat vascular smooth muscle cells. Life Sci. (2010) 87(11):367–74. 10.1016/j.lfs.2010.07.012 20670634

[41] Marx N Duez H Fruchart J-C Staels B. Peroxisome proliferator-activated receptors and atherogenesis. Circ Res. (2004) 94(9):1168–78. 10.1161/01.RES.0000127122.22685.0A 15142970

[42] Ghaderian SM Lindsey NJ Graham AM Homer-Vanniasinkam S Akbarzadeh Najar R. Pathogenic mechanisms in varicose vein disease: the role of hypoxia and inflammation. Pathology. (2010) 42(5):446–53. 10.3109/00313025.2010.493865 20632821

[43] Kim JE Jeong HW Nam JO Lee BH Choi JY Park RW Identification of motifs in the fasciclin domains of the transforming growth factor-beta-induced matrix protein betaig-h3 that interact with the alphavbeta5 integrin. J Biol Chem. (2002) 277(48):46159–65. 10.1074/jbc.M207055200 12270930

[44] Lindner V Wang Q Conley BA Friesel RE Vary CPH. Vascular injury induces expression of periostin. Arterioscler Thromb Vasc Biol. (2005) 25(1):77–83. 10.1161/01.ATV.0000149141.81230.c6 15514205

[45] Antonellis A Green ED. The role of aminoacyl-tRNA synthetases in genetic diseases. Annu Rev Genomics Hum Genet. (2008) 9:87–107. 10.1146/annurev.genom.9.081307.164204 18767960

[46] Belostotsky R Ben-Shalom E Rinat C Becker-Cohen R Feinstein S Zeligson S Mutations in the mitochondrial seryl-tRNA synthetase cause hyperuricemia, pulmonary hypertension, renal failure in infancy and alkalosis, HUPRA syndrome. Am J Hum Genet. (2011) 88(2):193–200. 10.1016/j.ajhg.2010.12.010 21255763 PMC3035710

[47] Palo JU Ulmanen I Lukka M Ellonen P Sajantila A. Genetic markers and population history: Finland revisited. Eur J Hum Genet. (2009) 17(10):1336–46. 10.1038/ejhg.2009.53 19367325 PMC2986642

