# Supplementary material for: Identification of potential drug targets for varicose veins: a Mendelian randomization analysis
Source: Front Cardiovasc Med. 2023 Jun 19;10:1126208. doi: 10.3389/fcvm.2023.1126208 (PMC10315832; doi:10.3389/fcvm.2023.1126208)
Supplement: Supplementary file 1 [file Datasheet1.pdf]

Supplementary Material

Identification of Potential Drug Targets for Varicose Veins: A Mendelian Randomization Analysis

Jianfeng Lin[†1], Jiawei Zhou[†2], Zhili Liu[2], Rong Zeng[2], Lei Wang[2], Fangda Li[2], Liqiang Cui[2], Yuehong Zheng[*2]

*** Correspondence:** Yuehong Zheng: zhengyuehong2022@outlook.com

1 Supplementary Data

Figure S1 Bidirectional MR analysis for varicose veins on levels of eight prioritized proteins

Figure S2 Bayesian colocalization analysis for COLEC11 and varicose veins

Figure S3 Bayesian colocalization analysis for IRF3 and varicose veins

Figure S4 Bayesian colocalization analysis for LUM and varicose veins

Figure S5 Bayesian colocalization analysis for POSTN and varicose veins

Figure S6 Bayesian colocalization analysis for RPN1 and varicose veins

Figure S7 Bayesian colocalization analysis for RSPO3 and varicose veins

Figure S8 Bayesian colocalization analysis for SARS2 and varicose veins

Figure S9 Bayesian colocalization analysis for VAT1 and varicose veins

Figure S10 Replication of causal relationship between eight prioritized proteins and varicose veins

Table S1 Genetic instruments of plasma proteins used in primary MR analysis

Table S2 Genetic instruments of eight prioritized proteins for replication

Table S3 Genetic instruments of varicose veins used in bidirectional MR

Table S4 GWAS catalog of 525 diseases used for phenome-wide Mendelian Randomization analysis

Table S5 Genome-wide significant association of SNPs as genetic instruments of eight prioritized proteins

Table S6 MR Steiger filtering analysis of SNPs as genetic instruments of eight prioritized proteins

Table S7 Phenome-wide Mendelian Randomization analysis to reveal potential on-target side effects of COLEC11

Table S8 Phenome-wide Mendelian Randomization analysis to reveal potential on-target side effects of IRF3

Table S9 Phenome-wide Mendelian Randomization analysis to reveal potential on-target side effects of LUM

Table S10 Phenome-wide Mendelian Randomization analysis to reveal potential on-target side effects of POSTN

Table S11 Phenome-wide Mendelian Randomization analysis to reveal potential on-target side effects of RPN1

Table S12 Phenome-wide Mendelian Randomization analysis to reveal potential on-target side effects of RSPO3

Table S13 Phenome-wide Mendelian Randomization analysis to reveal potential on-target side effects of SARS2

Table S14 Phenome-wide Mendelian Randomization analysis to reveal potential on-target side effects of VAT1

Figure S1 Bidirectional MR analysis for varicose veins on levels of eight prioritized proteins

Protein	Method	OR (95%CI)	P-value
COLEC11	Inverse variance weighted	0.99 (0.96, 1.02)	0.501
	MR Egger	1.01 (0.94, 1.09)	0.840
	Weighted median	1.00 (0.96, 1.05)	0.918
	Simple mode	0.92 (0.84, 1.01)	0.096
	Weighted mode	1.00 (0.94, 1.05)	0.936
IRF3	Inverse variance weighted	0.99 (0.96, 1.02)	0.365
	MR Egger	1.00 (0.92, 1.08)	0.989
	Weighted median	0.99 (0.95, 1.03)	0.609
	Simple mode	0.94 (0.86, 1.03)	0.169
	Weighted mode	0.98 (0.93, 1.03)	0.443
LUM	Inverse variance weighted	1.00 (0.97, 1.03)	0.848
	MR Egger	1.02 (0.94, 1.11)	0.625
	Weighted median	1.01 (0.97, 1.06)	0.629
	Simple mode	1.01 (0.93, 1.10)	0.770
	Weighted mode	1.02 (0.97, 1.07)	0.458
POSTN	Inverse variance weighted	0.99 (0.96, 1.02)	0.385
	MR Egger	0.98 (0.91, 1.07)	0.712
	Weighted median	0.98 (0.94, 1.02)	0.372
	Simple mode	0.96 (0.89, 1.03)	0.239
	Weighted mode	0.98 (0.93, 1.03)	0.381
RPN1	Inverse variance weighted	0.99 (0.96, 1.02)	0.615
	MR Egger	1.00 (0.92, 1.09)	0.984
	Weighted median	1.00 (0.96, 1.04)	0.885
	Simple mode	1.00 (0.93, 1.07)	0.903
	Weighted mode	1.00 (0.95, 1.04)	0.858
RSPO3	Inverse variance weighted	0.96 (0.87, 1.07)	0.498
	MR Egger	1.08 (0.82, 1.43)	0.595
	Weighted median	0.99 (0.95, 1.04)	0.806
	Simple mode	0.99 (0.92, 1.07)	0.851
	Weighted mode	0.99 (0.95, 1.04)	0.753
SARS2	Inverse variance weighted	0.99 (0.96, 1.02)	0.498
	MR Egger	0.94 (0.88, 1.01)	0.117
	Weighted median	0.99 (0.95, 1.04)	0.764
	Simple mode	1.00 (0.92, 1.08)	0.919
	Weighted mode	0.99 (0.94, 1.05)	0.741
VAT1	Inverse variance weighted	1.00 (0.97, 1.03)	0.905
	MR Egger	0.99 (0.92, 1.07)	0.849
	Weighted median	1.00 (0.96, 1.05)	0.965
	Simple mode	1.02 (0.94, 1.11)	0.634
	Weighted mode	1.01 (0.94, 1.08)	0.796

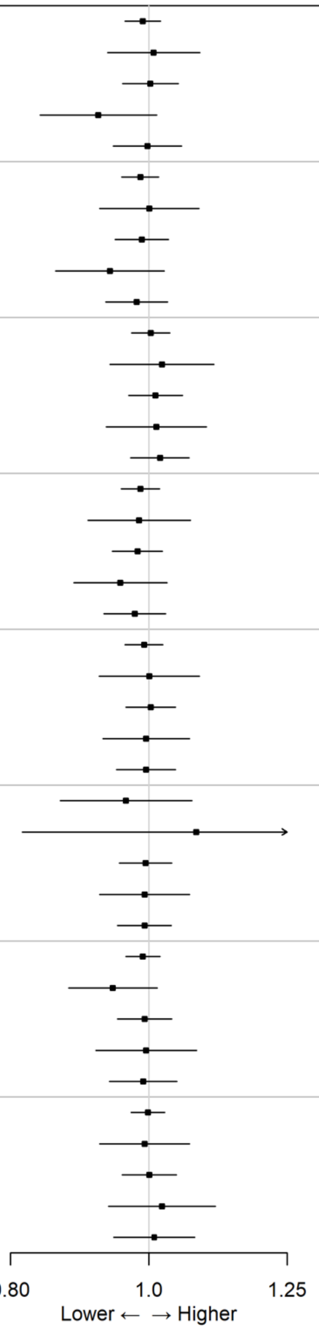

Figure S2 Bayesian colocalization analysis for COLEC11 and varicose veins

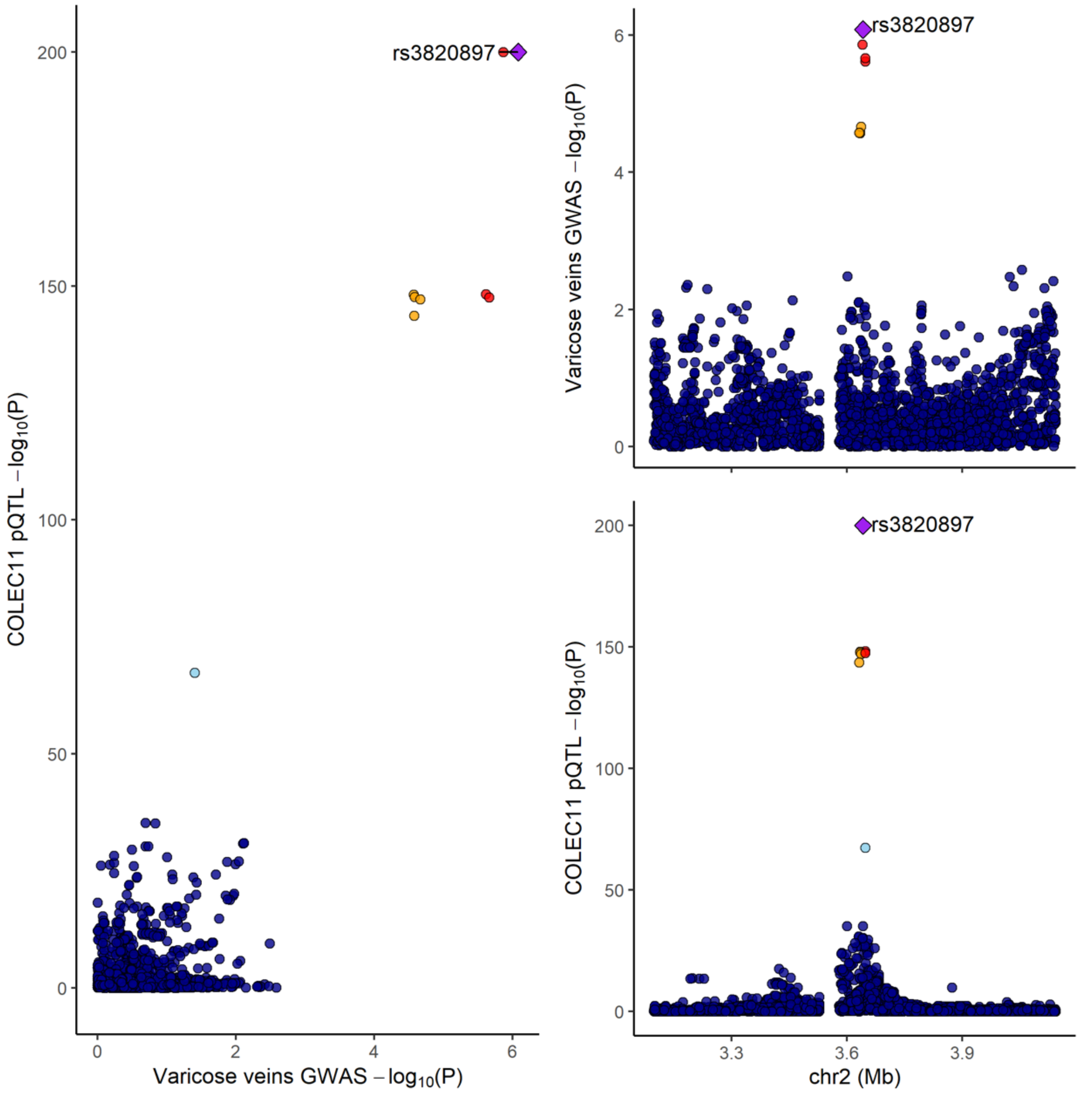

Figure S3 Bayesian colocalization analysis for IRF3 and varicose veins

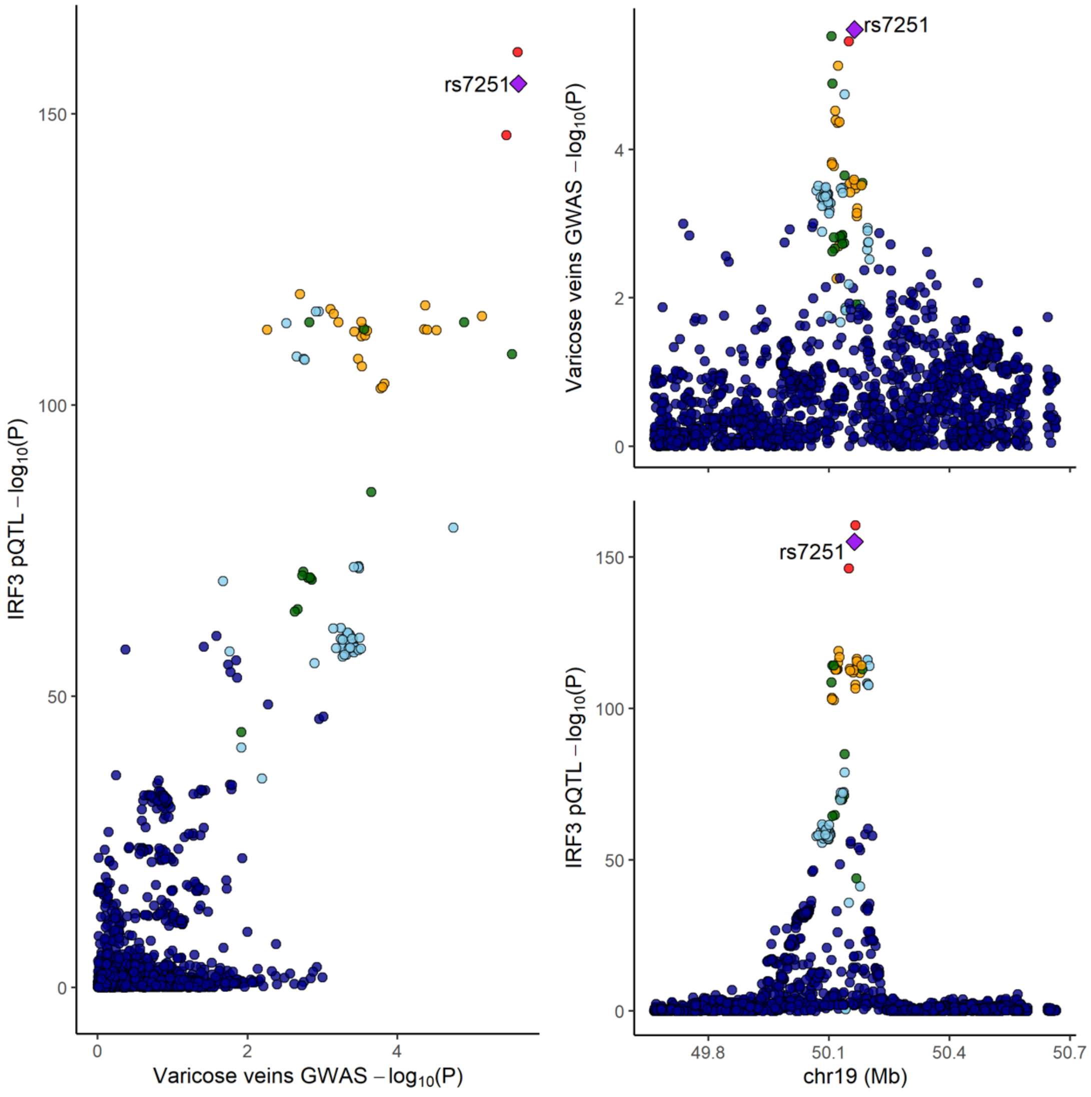

Figure S4 Bayesian colocalization analysis for LUM and varicose veins

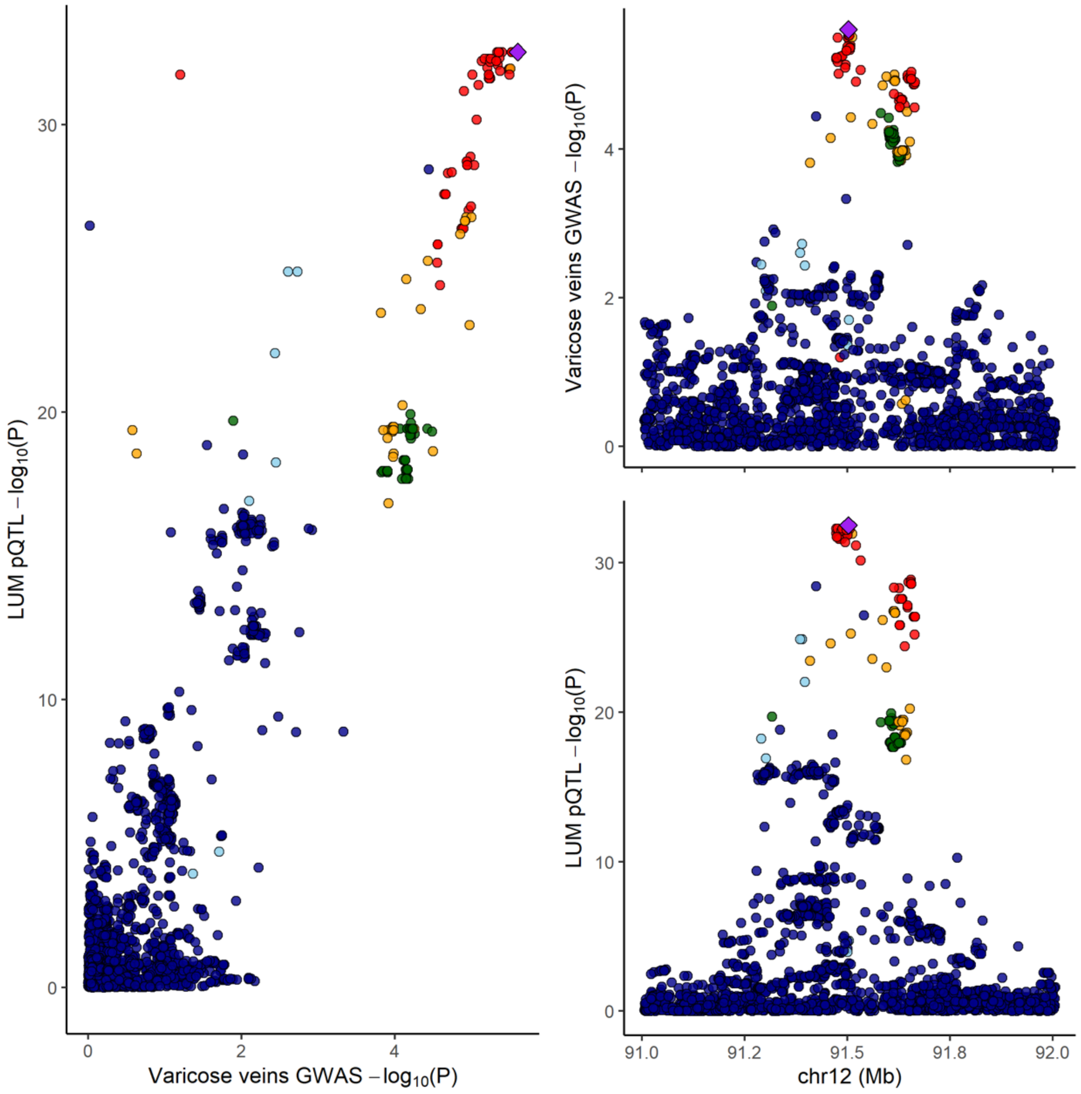

Figure S5 Bayesian colocalization analysis for POSTN and varicose veins

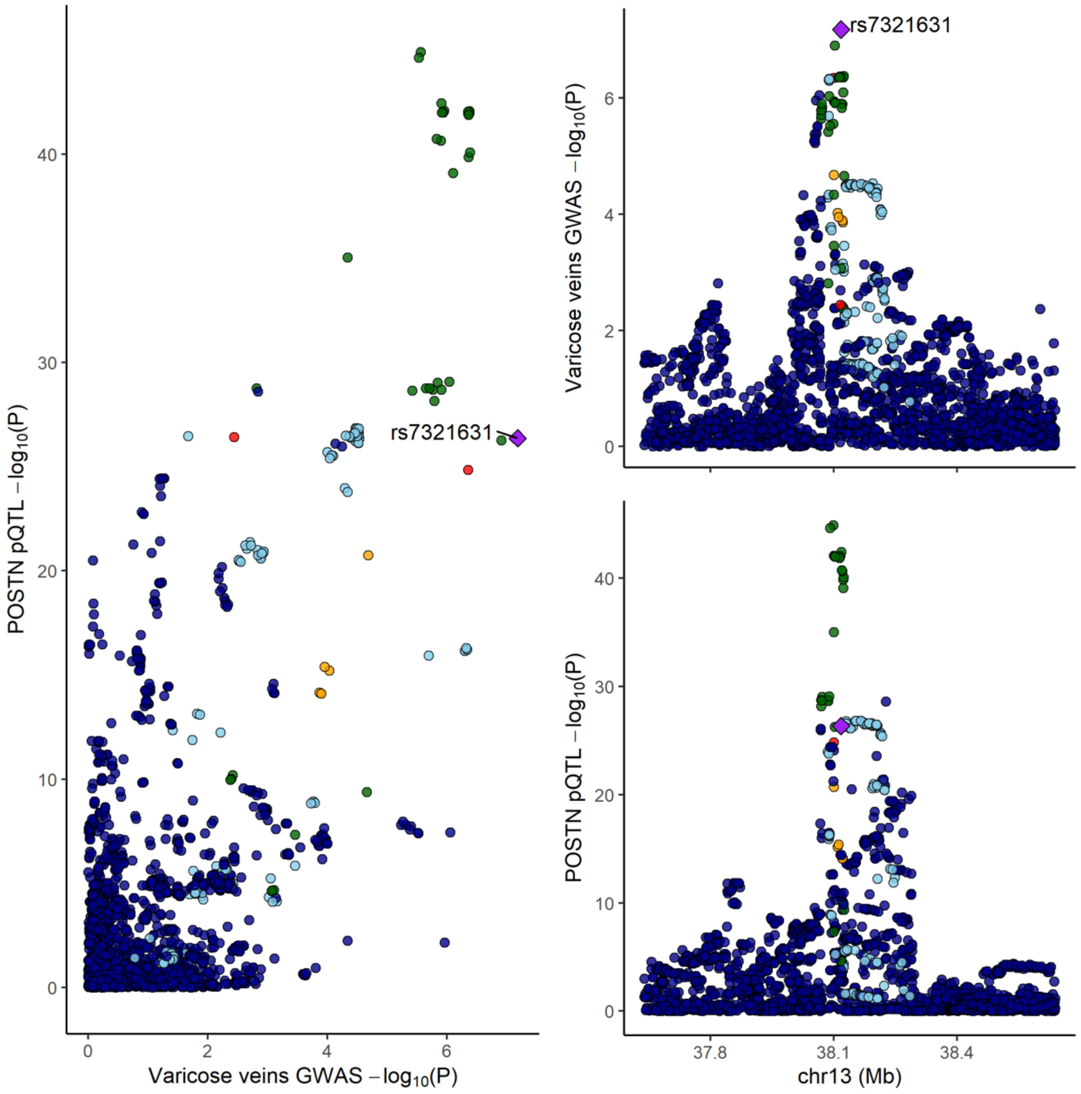

Figure S6 Bayesian colocalization analysis for RPN1 and varicose veins

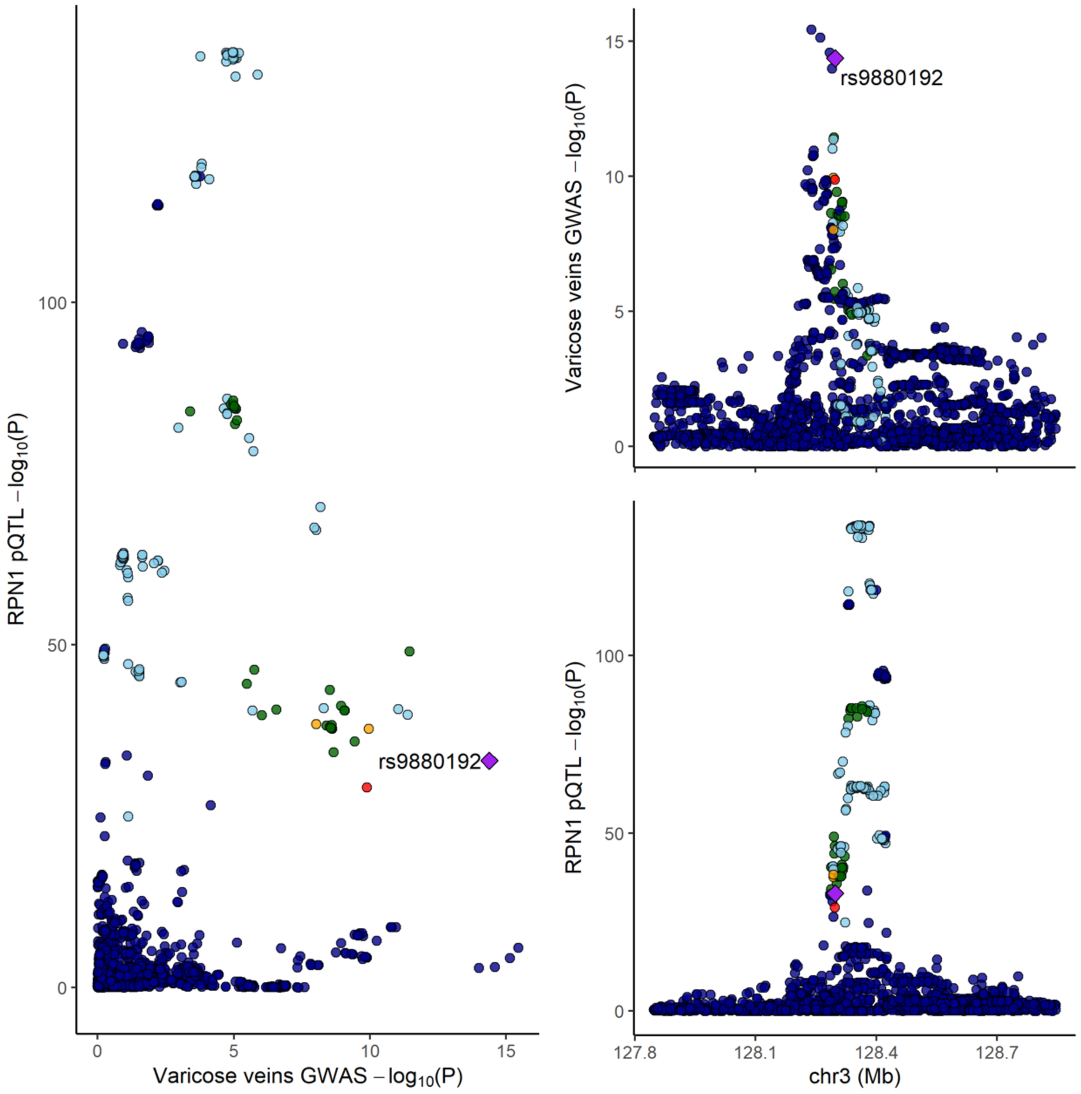

Figure S7 Bayesian colocalization analysis for RSPO3 and varicose veins

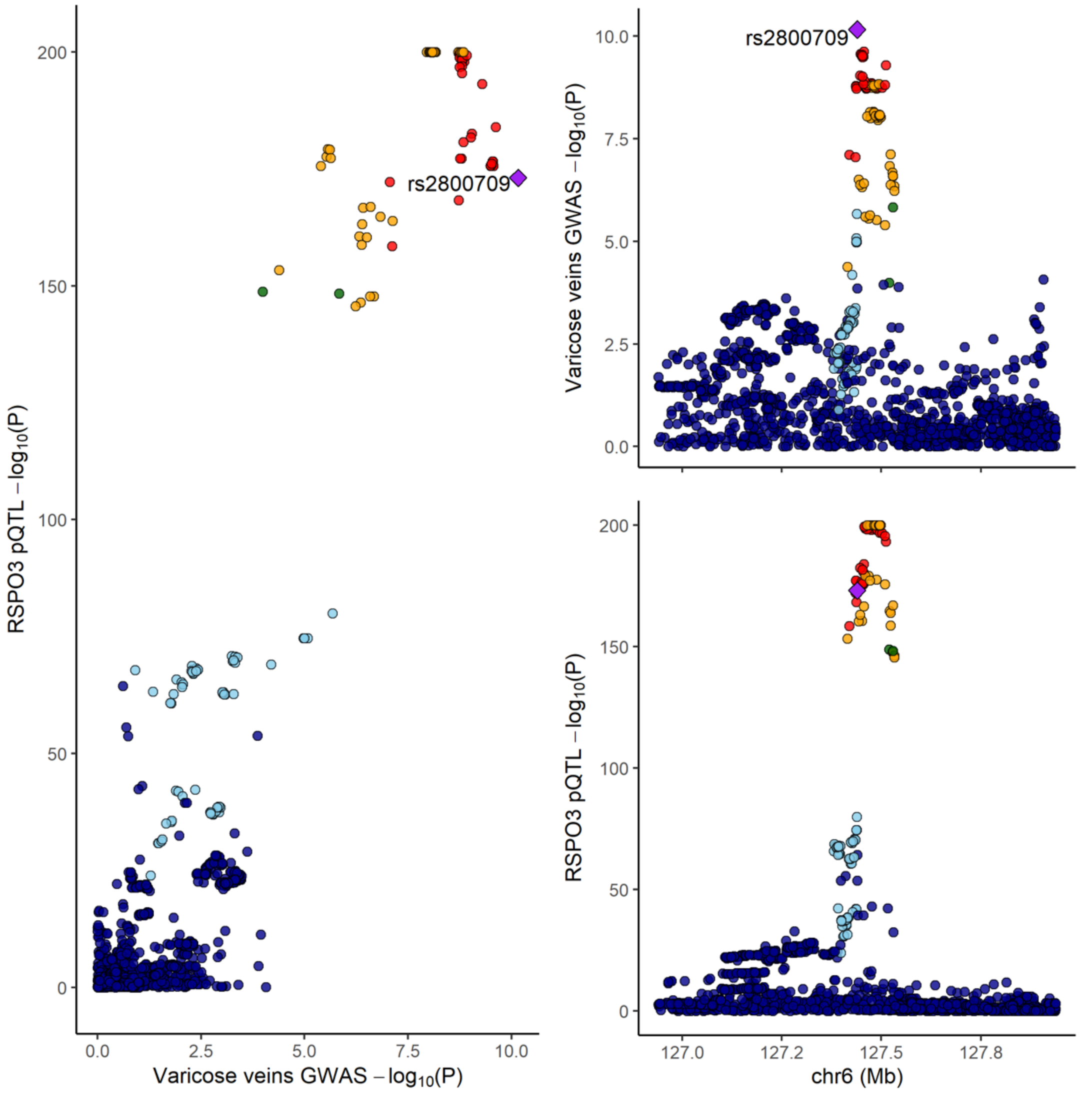

Figure S8 Bayesian colocalization analysis for SARS2 and varicose veins

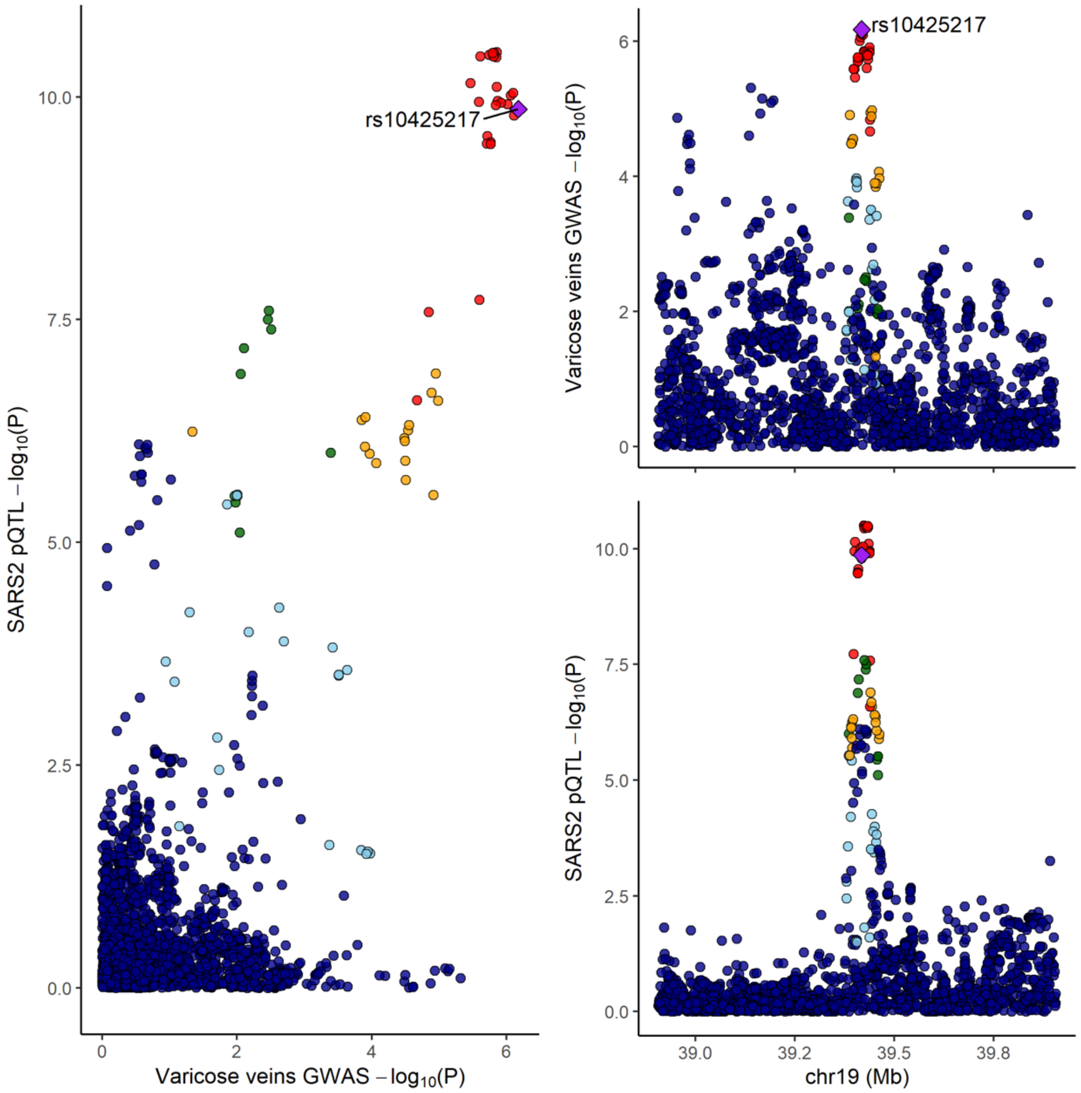

Figure S9 Bayesian colocalization analysis for VAT1 and varicose veins

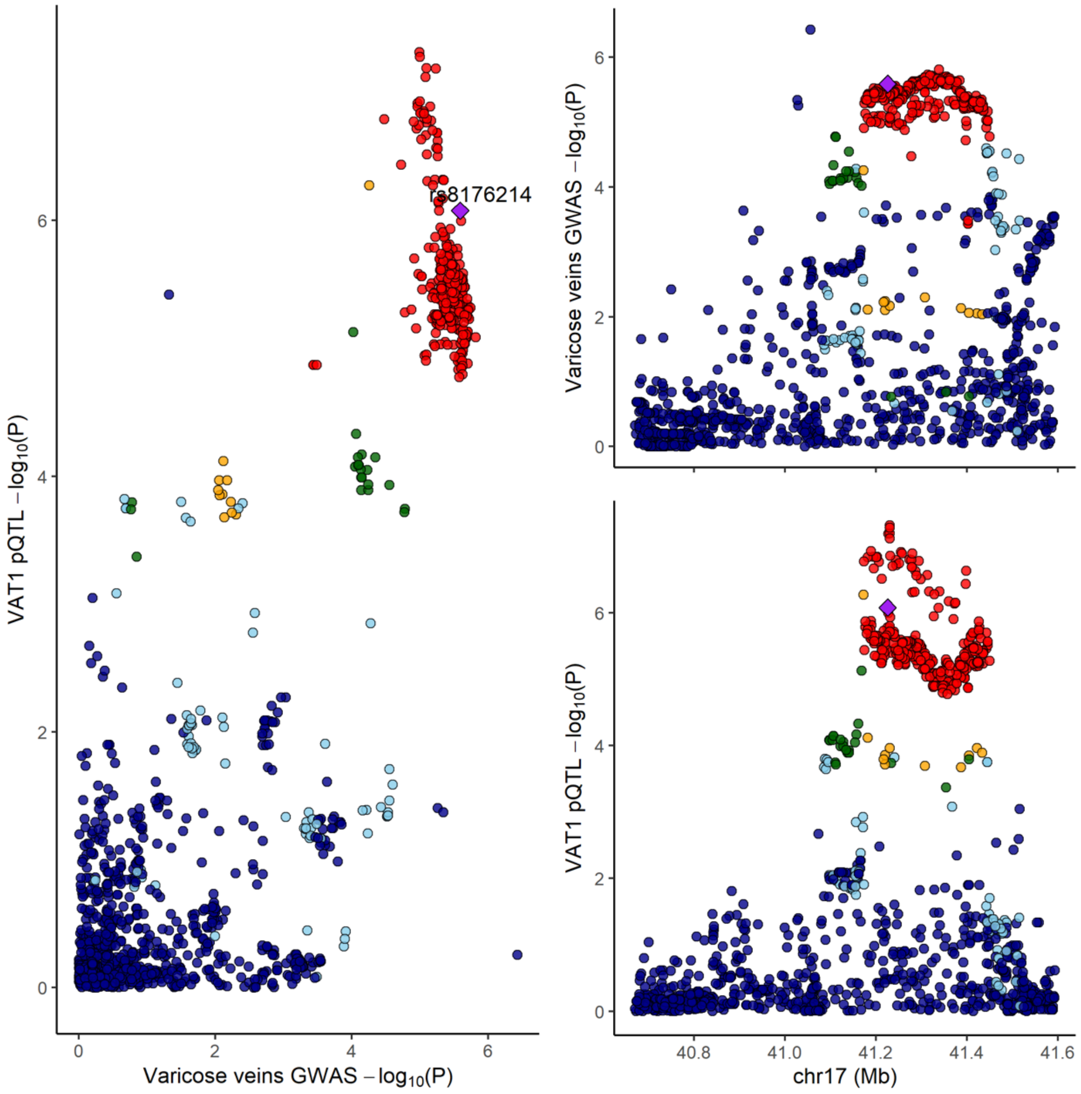

Figure S10 Replication of causal relationship between eight prioritized proteins and varicose veins

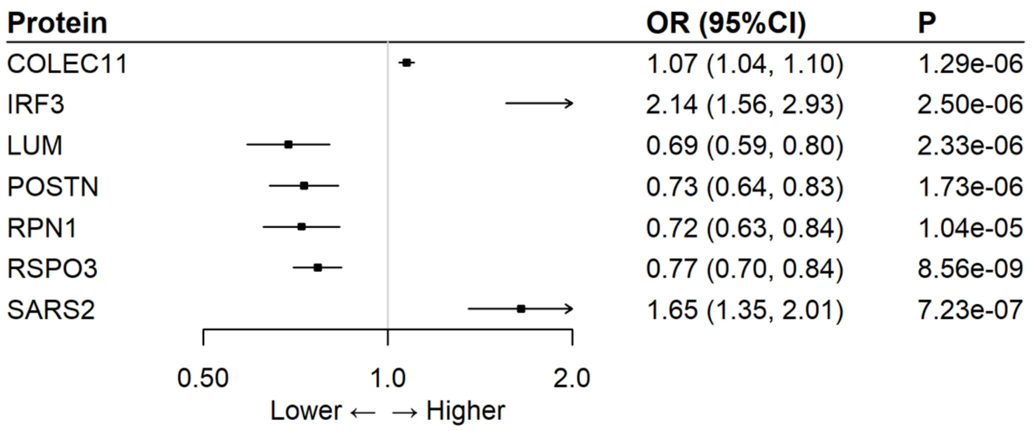

Table S1 Genetic instruments of plasma proteins used in primary MR analysis

UniProt id	protein	SNP	chromosome	position	effect allele	beta	P value	sample size	F statistics
A2RU67	FAM234B	rs182170698	12	13043986	A	0.824693	3.56596E-43	7213	189.7716436
A6NDG6	PGP	rs116977380	16	2213835	T	0.884351	1.58E-90	7213	407.0849792
A6NGN9	IGLON5	rs7246422	19	51309159	C	0.173209	2.87253E-25	7213	107.8682452
A6NH11	GLTPD2	rs34460487	17	4781933	A	0.284825	4.82476E-61	7213	271.704268
A6NHS7	MANSC4	rs12368838	12	27768744	G	0.889503	1E-200	7213	1373.872631
A6NI73	LILRA5	rs397600	19	54287934	G	-0.275889	2.20917E-56	7213	250.3220542
A8K2U0	A2ML1	rs1558526	12	8857224	A	-0.262192	5.51167E-43	7213	188.9053034
O00151	PDLIM1	rs1328599	10	95294018	G	0.451264	3.38156E-50	7213	221.9588489
O00182	LGALS9	rs4239242	17	27647232	C	-0.300878	7.06059E-70	7213	312.2511108
O00233	PSMD9	rs10743185	12	121918529	G	-0.205487	1.75144E-34	7213	149.9797072
O00241	SIRPB1	rs12480515	20	1558519	T	0.844241	1E-200	7213	1373.872631
O00244	ATOX1	rs1549921	5	151758650	G	-0.194396	3.53682E-31	7213	134.8634101
O00253	AGRP	rs114322795	16	67476655	T	0.663465	1.79556E-54	7213	241.5616532
O00292	LEFTY2	rs360077	1	225897466	C	0.750451	1E-200	7213	1373.872631
O00300	TNFRSF11B	rs2468184	8	119185624	A	-0.141371	4.42107E-17	7213	70.57941148
O00330	PDHX	rs2915221	11	34892933	C	0.335834	4.50712E-81	7213	363.6542197
O00339	MATN2	rs17831160	8	98033638	A	0.566124	1.79223E-36	7213	159.0857989
O00451	GFRA2	rs15881	8	21693256	C	0.359501	6.0557E-104	7213	468.7298524
O00462	MANBA	rs223492	4	102753951	C	0.697192	1E-200	7213	1373.872631
O00468	AGRN	rs4970350	1	1036800	G	-0.294348	2.29684E-68	7213	305.3090988
O00469	PLOD2	rs16857612	3	145939653	A	0.124749	3.31443E-09	7213	34.98976467
O00478	BTN3A3	rs9393711	6	26370431	T	-1.17022	1E-200	7213	1373.872631
O00481	BTN3A1	rs9467732	6	26379212	T	-0.179054	1.18058E-22	7213	95.94603586
O00499	BIN1	rs35103166	2	127124606	C	0.189816	9.16264E-29	7213	123.8333106
O00533	CHL1	rs990284	3	63289	G	-0.306673	1.9066E-71	7213	319.4520378
O00548	DLL1	rs4710790	6	170279144	C	0.175398	9.6805E-26	7213	110.0241331
O00584	RNASET2	rs9457245	6	166927227	T	-0.841235	3.1591E-165	7213	750.477583
O00585	CCL21	rs10972201	9	34707376	A	-0.121072	3.83139E-12	7213	48.20893257
O00587	MFNG	rs8192548	22	37470025	A	-0.7261	4.11671E-29	7213	125.4209283
O00602	FCN1	rs7037264	9	134883366	A	0.489293	2.9879E-196	7213	893.1756026
O00626	CCL22	rs170364	16	57376022	T	-0.227769	2.99025E-32	7213	139.7690955
O00746	NME4	rs6600214	16	394814	T	0.173731	9.08499E-23	7213	96.4646861
O00764	PDXK	rs79343092	21	43861371	G	-0.748543	2.69985E-24	7213	103.4283398
O14594	NCAN	rs2228603	19	19219115	T	-0.451966	3.17631E-50	7213	222.0835263
O14610	GNGT2	rs35638197	17	49207373	C	-0.221329	1.27953E-09	7213	36.84424851
O14625	CXCL11	rs6827617	4	75994993	G	0.192949	8.31923E-30	7213	128.5944583
O14737	PDCD5	rs4499344	19	32582525	A	0.713482	1E-200	7213	1373.872631
O14773	TPP1	rs11040938	11	6633382	G	0.229556	3.65256E-42	7213	185.1429201
O14786	NRP1	rs734186	10	33195901	A	-0.394068	2.0761E-120	7213	544.4045559
O14791	APOL1	rs9610469	22	36257706	G	-0.425378	1.8367E-127	7213	576.8281151
O14810	CPLX1	rs11248039	4	772737	C	-0.144202	3.74774E-16	7213	66.36459294
O14907	TAX1BP3	rs160589	17	3668569	A	0.488345	1.9974E-19	7213	81.24162766
O14960	LECT2	rs2428665	5	135977944	A	-0.769895	1E-200	7213	1373.872631
O15031	PLXNB2	rs28573806	22	50289363	C	0.644498	1E-200	7213	1373.872631
O15041	SEMA3E	rs73707827	7	83410464	A	-1.07026	1E-200	7213	1373.872631
O15075	DCLK1	rs7339267	13	36073931	A	0.309996	3.00331E-53	7213	235.9509946
O15123	ANGPT2	rs1968586	8	6421990	T	-0.218101	1.49006E-36	7213	159.4528111
O15197	EPHB6	rs7789303	7	142854785	G	-0.59113	1E-200	7213	1095.619758
O15232	MATN3	rs11694716	2	20007858	G	0.743662	1E-200	7213	1373.872631
O15264	MAPK13	rs12210904	6	36130414	A	0.146546	2.24498E-15	7213	62.8373754
O15305	PMM2	rs34258285	16	8813057	C	-0.660606	4.43159E-40	7213	175.5982771
O15335	CHAD	rs184613584	17	50430860	C	-0.597236	1.75525E-14	7213	58.78894165
O15382	BCAT2	rs62125920	19	48803019	G	-0.58207	3.9715E-182	7213	828.2094549
O15389	SIGLEC5	rs872629	19	51627385	A	-0.996177	1E-200	7213	1373.872631
O15394	NCAM2	rs2826851	21	21463626	G	0.549438	1E-200	7213	949.1986203
O15400	STX7	rs3813356	6	132513379	T	-0.222839	1.51498E-40	7213	177.7330388
O15444	CCL25	rs74959615	19	8056212	A	-1.17087	1E-200	7213	1373.872631
O15455	TLR3	rs3775291	4	186082920	T	-0.788587	1E-200	7213	1373.872631
O15467	CCL16	rs112689088	17	35980421	C	-1.30751	1E-200	7213	1373.872631
O43155	FLRT2	rs17796783	14	85343567	C	-0.536857	6.3092E-200	7213	910.0827255
O43240	KLK10	rs2569454	19	51019947	T	-0.658504	1E-200	7213	1373.872631
O43278	SPINT1	rs17658212	15	40853721	T	-0.480648	1.56005E-45	7213	200.5805742
O43291	SPINT2	rs71354995	19	38301201	G	-0.860904	1E-200	7213	1373.872631
O43300	LRRTM2	rs10071381	5	138937159	T	0.181441	2.49742E-23	7213	99.02176413
O43353	RIPK2	rs34211510	8	89771518	T	0.18844	5.42317E-10	7213	38.51869509
O43405	COCH	rs28400019	14	30874288	A	0.711235	2.9994E-181	7213	824.1705995
O43447	PPIH	rs78676038	1	42663404	C	0.299979	2.3818E-11	7213	44.62865589
O43488	AKR7A2	rs74430522	1	19262680	A	-0.521227	4.72472E-77	7213	345.1910545
O43508	TNFSF12	rs62059804	17	7550188	A	0.180916	2.13813E-21	7213	90.21336392
O43555	GNRH2	rs6138990	20	3041972	T	-0.161103	2.78905E-12	7213	48.83163332
O43598	DNPH1	rs114371775	6	43231992	C	-0.276876	3.23036E-25	7213	107.6355654
O43633	CHMP2A	rs11673465	19	58527528	C	0.140955	1.19274E-11	7213	45.98315764
O43639	NCK2	rs1465639	2	105822725	C	-0.177641	1.7119E-25	7213	108.8941254
O43699	SIGLEC6	rs4802807	19	51527194	A	0.603958	1E-200	7213	1373.872631
O43708	GSTZ1	rs7975	14	77326864	A	0.831763	1E-200	7213	1373.872631

O43813	LANCL1	rs2287418	2	210476432	C	-0.136082	3.4506E-16	7213	66.52742089
O43819	SCO2	rs11479	22	50525807	A	-0.278112	9.34361E-22	7213	91.85139281
O43854	EDIL3	rs10073371	5	83920960	C	-0.149468	4.28605E-09	7213	34.48928164
O43866	CD5L	rs2765501	1	157834858	A	0.376988	6.978E-113	7213	509.8091917
O43897	TLL1	rs1903176	4	165742950	A	-0.0989996	2.05377E-08	7213	31.44304452
O43916	CHST1	rs753198	11	45733411	C	-0.109412	3.38966E-09	7213	34.94606353
O60234	GMFG	rs251906	19	39336198	G	-0.396104	1.71584E-41	7213	182.0653854
O60243	HS6ST1	rs4662790	2	128294778	A	0.251496	1.56897E-51	7213	228.0729177
O60256	PRPSAP2	rs80251691	17	18859663	A	-0.190592	7.76152E-09	7213	33.33396377
O60259	KLK8	rs10410942	19	51000397	C	-0.796823	3.637E-131	7213	593.8533851
O60449	LY75	rs17231342	2	159870033	G	0.870925	1E-200	7213	1373.872631
O60462	NRP2	rs62172729	2	205182223	T	-0.252313	7.75233E-33	7213	142.450252
O60469	DSCAM	rs78111814	21	40688572	C	0.467451	6.48105E-33	7213	142.8060133
O60476	MAN1A2	rs4659047	1	117370451	G	-0.569835	2.8541E-109	7213	493.2094787
O60486	PLXNC1	rs2291331	12	94227410	A	-0.540059	1E-200	7213	1122.259987
O60496	DOK2	rs17615788	8	21957228	T	0.203088	7.15962E-34	7213	147.182253
O60507	TPST1	rs313829	7	66087510	A	-0.372838	5.85934E-98	7213	441.2249593
O60551	NMT2	rs11259539	10	15197030	C	-0.227925	5.00113E-42	7213	184.5177954
O60565	GREM1	rs58658771	15	32709533	A	0.937288	1E-200	7213	1373.872631
O60575	SPINK4	rs1630171	9	33234041	G	0.80272	1E-200	7213	1297.577045
O60667	FCMR	rs72758947	1	206913708	G	-0.187416	1.00714E-13	7213	55.35303978
O60684	KPNA6	rs72666741	1	32102997	A	0.858735	7.97468E-67	7213	298.237735
O60701	UGDH	rs1138891	4	39532402	T	-0.455336	2.5326E-161	7213	732.5231005
O60704	TPST2	rs4275	22	26525759	G	0.268985	1.13974E-57	7213	256.2277202
O60760	HPGDS	rs10033662	4	94318311	G	-0.571691	1E-200	7213	1201.353855
O60909	B4GALT2	rs1859728	1	43981741	C	-0.982315	2.623E-154	7213	700.2617113
O60911	CTSV	rs4743059	9	97137031	T	0.390775	1.12739E-35	7213	155.4306966
O75015	FCGR3B	rs10800573	1	161538134	G	0.462213	6.5836E-156	7213	707.6210838
O75022	LILRB3	rs12978577	19	54213755	A	0.523234	1.4985E-200	7213	912.954681
O75023	LILRB5	rs12975366	19	54255498	C	-0.920434	1E-200	7213	1373.872631
O75054	IGSF3	rs655735	1	116592497	A	-0.204503	1.58725E-34	7213	150.1752915
O75063	FAM20B	rs12568291	1	179017420	A	0.166602	5.50698E-22	7213	92.89765196
O75077	ADAM23	rs1448903	2	206444237	G	0.857603	1E-200	7213	930.4477396
O75096	LRP4	rs2306029	11	46871557	T	0.459609	9.9673E-171	7213	775.7774661
O75144	ICOSLG	rs2838537	21	44263692	G	0.159332	4.80089E-20	7213	84.0595693
O75326	SEMA7A	rs78994380	15	74400071	A	-0.179892	5.19815E-12	7213	47.6107933
O75340	PDCD6	rs56075848	5	308237	C	0.234281	3.80857E-12	7213	48.22064652
O75354	ENTPD6	rs6050446	20	25214873	A	-0.612944	3.48276E-34	7213	148.6139629
O75356	ENTPD5	rs73301475	14	73996165	T	-1.25585	1E-200	7213	1329.796583
O75382	TRIM3	rs2344827	11	6464439	G	-0.149853	2.09195E-11	7213	44.88272796
O75436	VPS26A	rs35773981	10	69185042	C	-0.487651	1.6064E-136	7213	618.4730968
O75462	CRLF1	rs141412226	19	18606827	A	0.337598	1.41945E-61	7213	274.142382
O75473	LGR5	rs17109804	12	71551792	G	0.156248	9.01677E-13	7213	51.04728117
O75493	CA11	rs62130341	19	48675495	T	0.167622	7.31395E-10	7213	37.93504678
O75503	CLN5	rs17067027	13	76965650	T	-0.525036	2.96804E-24	7213	103.2407108
O75509	TNFRSF21	rs6458555	6	47309833	A	0.286235	1.82177E-60	7213	269.0567236
O75521	ECI2	rs7757606	6	4119470	A	-0.445632	7.3036E-142	7213	643.0365464
O75594	PGLYRP1	rs2072563	19	46023390	A	-0.213745	6.66427E-33	7213	142.7506401
O75629	CREG1	rs7513428	1	167546035	T	0.334788	1.84613E-47	7213	209.4114926
O75636	FCN3	rs111257457	1	27389173	A	-0.542905	1.52558E-83	7213	375.0005676
O75828	CBR3	rs60376898	21	36133406	A	-0.846144	1E-200	7213	1373.872631
O75843	AP1G2	rs35309020	14	23554817	A	-0.446406	1.19683E-52	7213	233.1975419
O75874	IDH1	rs3928183	2	208247493	T	-0.845273	2.8401E-125	7213	566.7635424
O75882	ATRN	rs118065662	20	3582219	G	-1.9218	6.4796E-155	7213	703.054213
O75973	C1QL1	rs7225162	17	44958892	C	0.327862	1.6367E-90	7213	407.0146368
O76003	GLRX3	rs12269149	10	130146915	T	-0.126512	7.79015E-12	7213	46.81782805
O76036	NCR1	rs2915993	19	54921974	A	-0.17854	7.1851E-26	7213	110.6150776
O76074	PDE5A	rs58583086	4	119635207	A	0.502202	5.883E-194	7213	882.6221497
O76076	CCN5	rs6065746	20	44693361	G	-0.348585	3.81953E-98	7213	442.0788537
O76096	CST7	rs4462848	20	24922814	C	-0.561801	5.6679E-187	7213	850.4974492
O94760	DDAH1	rs233071	1	85340322	T	-0.124186	7.18005E-13	7213	51.49443055
O94766	B3GAT3	rs7122950	11	62617896	T	-0.416871	2.1239E-117	7213	530.5690812
O94779	CNTN5	rs4528296	11	99222947	C	0.731417	4.4461E-119	7213	538.2875101
O94813	SLIT2	rs587668	4	20189349	C	0.156569	8.99961E-18	7213	73.72057058
O94856	NFASC	rs6663324	1	204977636	C	0.823901	1E-200	7213	1001.443024
O94907	DKK1	rs11003047	10	52674751	G	0.570006	2.5105E-100	7213	452.1061873
O94923	GLCE	rs3865014	15	69269179	G	-0.831332	1E-200	7213	1373.872631
O94929	ABLIM3	rs114464628	5	149154161	A	-0.311998	1.20875E-11	7213	45.9570363
O94933	SLITRK3	rs62282372	3	165194166	G	-0.570619	7.5353E-118	7213	532.6377257
O94973	AP2A2	rs4963151	11	920911	C	-0.168879	4.72458E-24	7213	102.3197512
O94985	CLSTN1	rs35331030	1	9751595	T	1.78296	9.7685E-180	7213	817.2123921
O94991	SLITRK5	rs12856670	13	88034278	T	-0.22543	3.06515E-35	7213	153.4430232
O94992	HEXIM1	rs12051846	17	45149520	G	0.210601	2.05706E-28	7213	122.2286923
O95149	SNUPN	rs7170787	15	75593089	A	0.359476	7.94577E-80	7213	357.9308637
O95150	TNFSF15	rs56339337	9	114798680	G	-0.157907	1.78632E-11	7213	45.19201434
O95154	AKR7A3	rs55717136	1	19246561	T	-0.483248	1.1243E-181	7213	826.1307508
O95185	UNC5C	rs1994292	4	95521608	T	0.313902	3.79078E-74	7213	331.8551868

O95236	APOL3	rs132642	22	36149089	A	-1.06113	1E-200	7213	1373.872631
O95256	IL18RAP	rs4851010	2	102439667	T	0.290845	3.48923E-57	7213	253.9986288
O95274	LYPD3	rs11666797	19	43458246	A	-0.245002	9.81943E-27	7213	114.5610856
O95336	PGLS	rs73020480	19	17518363	A	0.4129	1.05676E-92	7213	417.0756247
O95379	TNFAIP8	rs1032859	5	119305605	T	-0.490494	6.17875E-84	7213	376.8034841
O95388	CCN4	rs60282000	8	133192798	G	-0.292064	5.48084E-63	7213	280.6275295
O95393	BMP10	rs34008398	2	68866281	A	-0.579365	1.21506E-11	7213	45.94683614
O95407	TNFRSF6B	rs8957	20	63742354	G	0.151545	4.01331E-16	7213	66.22964453
O95460	MATN4	rs11906694	20	45299492	T	-0.3569	1.09829E-78	7213	352.6929541
O95479	H6PD	rs34603401	1	9245386	C	0.712904	1E-200	7213	1028.069836
O95498	VNN2	rs9493425	6	132753506	G	-0.739014	1E-200	7213	1373.872631
O95502	NPTXR	rs3817727	22	38835163	T	1.62178	6.0931E-159	7213	721.571962
O95544	NADK	rs4648629	1	1764023	A	-0.311603	2.39666E-78	7213	351.1367082
O95630	STAMBP	rs148932286	2	73829050	G	0.406138	4.80509E-09	7213	34.26680176
O95631	NTN1	rs9897200	17	9082193	T	-0.664774	3.8801E-146	7213	662.6922047
O95633	FSTL3	rs3787004	19	657666	C	-0.138794	4.92616E-17	7213	70.36600325
O95711	LY86	rs977785	6	6588648	A	-0.187434	4.19648E-23	7213	97.99401977
O95727	CRTAM	rs2370794	11	122844074	G	0.307747	2.67903E-67	7213	300.4121518
O95841	ANGPTL1	rs10913568	1	178544276	C	0.435634	1.0553E-158	7213	720.4749416
O95861	BPNT1	rs7530481	1	220063279	T	0.23611	4.25301E-38	7213	166.5226638
O95881	TXNDC12	rs6686632	1	52117939	C	-0.640626	6.24442E-91	7213	408.9371051
O95897	OLFM2	rs62104305	19	9948235	A	0.840317	1E-200	7213	1373.872631
O95998	IL18BP	rs17884883	11	72097010	T	0.10036	2.11331E-09	7213	35.86631466
O96013	PAK4	rs11667387	19	39184097	T	-0.11375	8.17186E-11	7213	42.21623969
P00167	CYB5A	rs6566789	18	74266264	A	-0.147174	3.26078E-15	7213	62.10225382
P00325	ADH1B	rs1229984	4	99318162	T	-0.29095	4.38692E-10	7213	38.93260475
P00326	ADH1C	rs283415	4	99349450	C	-0.542217	1E-200	7213	1124.691466
P00338	LDHA	rs7112492	11	18383963	G	0.190473	2.03989E-18	7213	76.65113305
P00390	GSR	rs2551710	8	30699094	C	-0.174943	1.1458E-23	7213	100.5649061
P00450	CP	rs13089226	3	149080893	T	0.272277	2.19817E-11	7213	44.78574675
P00491	PNP	rs1049564	14	20472447	A	-0.659672	1E-200	7213	993.7520008
P00533	EGFR	rs75059484	7	54868647	T	-0.251438	5.42428E-26	7213	111.1723873
P00568	AK1	rs78181560	9	127878806	T	-0.714771	1.30449E-56	7213	251.3715127
P00734	F2	rs1799963	11	46739505	A	1.07751	2.40688E-53	7213	236.3919106
P00736	C1R	rs10849546	12	7068900	A	-0.603834	7.811E-135	7213	610.7174382
P00738	HP	rs77303550	16	72045758	T	0.680945	1E-200	7213	1112.368439
P00742	F10	rs547138	13	113137856	T	-0.286206	7.14426E-65	7213	289.277619
P00746	CFD	rs72984031	19	872089	G	-0.196222	2.12233E-25	7213	108.4681545
P00749	PLAU	rs2633322	10	73934174	T	0.413473	5.2139E-110	7213	496.6027031
P00750	PLAT	rs77346091	8	42162640	C	-0.52766	2.12574E-20	7213	85.67037369
P00797	REN	rs141995914	1	204237969	C	0.727601	5.08221E-21	7213	88.50049728
P00915	CA1	rs116866430	8	85333298	G	-1.58931	5.21648E-76	7213	340.4017463
P00995	SPINK1	rs6580502	5	147828480	T	0.16339	2.47129E-22	7213	94.48361952
P01009	SERPINA1	rs28929474	14	94378610	T	-1.8065	1E-200	7213	920.9253328
P01011	SERPINA3	rs17753556	14	94629097	A	-0.525621	1.546E-139	7213	632.3431744
P01019	AGT	rs2071404	1	230715194	A	0.458801	4.22045E-66	7213	294.9163493
P01023	A2M	rs226393	12	9103235	T	-0.161404	8.53396E-21	7213	87.47528363
P01024	C3	rs2230199	19	6718376	C	0.225902	3.94245E-28	7213	120.9380903
P01031	C5	rs1035029	9	120980540	G	-0.227462	2.72399E-41	7213	181.1460037
P01034	CST3	rs2405367	20	23642243	A	-0.612633	1E-200	7213	929.9045046
P01036	CST4	rs7263473	20	23709830	A	-0.458975	9.05679E-64	7213	284.2155706
P01037	CST1	rs4260306	20	23749699	T	-0.506384	1.2372E-172	7213	784.5443919
P01042	KNG1	rs76438938	3	186743735	T	-1.51067	1E-200	7213	941.1528477
P01133	EGF	rs10029654	4	109940771	A	0.383437	2.3096E-108	7213	489.0361692
P01178	OXT	rs2875830	20	3073387	T	-0.368173	8.87822E-96	7213	431.2063547
P01210	PENK	rs2576581	8	56453391	A	-0.535131	1E-200	7213	1134.052267
P01229	LHB	rs3795050	19	49017606	C	-0.546216	6.22045E-71	7213	317.0943583
P01589	IL2RA	rs12722497	10	6053965	A	0.228191	1.09087E-15	7213	64.25909867
P01730	CD4	rs73053728	12	6787380	G	-0.148931	7.42088E-17	7213	69.55777124
P01732	CD8A	rs3020726	2	86789383	G	0.510069	1.1488E-117	7213	531.7959075
P01833	PIGR	rs6540730	1	206945992	C	-0.205398	6.48652E-35	7213	151.9533955
P01857	IGHG1	rs11621145	14	105706543	G	-0.178848	1.33335E-23	7213	100.2646565
P02008	HBZ	rs2461286	16	153255	A	0.694949	1E-200	7213	1373.872631
P02458	COL2A1	rs719008	12	48022269	T	-0.449708	6.9553E-158	7213	716.7088453
P02649	APOE	rs429358	19	44908684	C	0.299981	8.69554E-38	7213	165.1007722
P02649	APOE	rs35136575	19	44935906	G	-0.134428	6.51209E-12	7213	47.16904235
P02654	APOC1	rs5112	19	44927023	C	-0.307528	1.24059E-77	7213	347.8578448
P02656	APOC3	rs5141	11	116831407	T	0.198931	4.01518E-12	7213	48.11705904
P02679	FGG	rs2227399	4	154564701	G	0.218955	4.97067E-27	7213	115.9111814
P02741	CRP	rs2794520	1	159709026	T	-0.273903	9.31131E-55	7213	242.8696413
P02743	APCS	rs77672383	1	159608353	T	-0.919614	3.64514E-65	7213	290.6188536
P02747	C1QC	rs75380810	1	22663039	C	0.962427	9.5435E-115	7213	518.3767177
P02748	C9	rs696766	5	39356024	A	0.463091	2.05762E-37	7213	163.388425
P02749	APOH	rs1801689	17	66214462	C	-1.53413	1E-200	7213	1156.905503
P02751	FN1	rs1250258	2	215435462	C	-0.285448	2.15129E-52	7213	232.0297375
P02753	RBP4	rs10882283	10	93601207	C	-0.18099	5.26186E-26	7213	111.2326559
P02760	AMBP	rs10982050	9	114066493	G	-0.150718	4.82487E-15	7213	61.3307444

P02765	AHSG	rs2518134	3	186614393	C	-0.517787	1E-200	7213	939.4277004
P02771	AFP	rs667	4	73414548	T	-0.129298	3.65439E-11	7213	43.79069142
P02774	GC	rs7697091	4	71759380	C	-0.160771	1.02807E-20	7213	87.10698161
P02776	PF4	rs3756074	4	73982348	C	-0.455359	2.30642E-30	7213	131.1408872
P02778	CXCL10	rs151000561	4	76155134	T	0.471447	2.8016E-09	7213	35.3171097
P02786	TFRC	rs62282694	3	196109704	T	-0.111702	7.17124E-09	7213	33.48780016
P02787	TF	rs8177245	3	133760386	G	0.171696	1.407E-22	7213	95.59868279
P03950	ANG	rs4982325	14	20688677	T	0.831794	1E-200	7213	1124.69424
P03951	F11	rs2289252	4	186286227	T	0.418577	1.6607E-139	7213	632.2002374
P03952	KLKB1	rs4253304	4	186252417	C	0.36051	3.5266E-105	7213	474.4043645
P03956	MMP1	rs1144396	11	102808321	C	-0.309328	4.21726E-79	7213	354.6018941
P03973	SLPI	rs6104034	20	45137017	G	-0.224728	7.23273E-42	7213	183.7838336
P04003	C4BPA	rs11120218	1	207105106	A	0.776306	1E-200	7213	1079.46788
P04040	CAT	rs4756148	11	34465650	T	-0.349438	1.26452E-70	7213	315.6799302
P04070	PROC	rs1799809	2	127418299	G	-0.231169	5.25207E-43	7213	189.0012914
P04083	ANXA1	rs2795108	9	73150562	C	0.255113	5.29939E-25	7213	106.6545616
P04118	CLPS	rs9380535	6	35783834	T	-0.415573	7.1073E-135	7213	610.9059581
P04155	TFF1	rs3761376	21	42366929	A	-0.365113	1.09633E-77	7213	348.1043781
P04196	HRG	rs7617554	3	186676762	T	0.736658	1E-200	7213	1373.872631
P04217	A1BG	rs1268538	19	58341997	T	-1.12335	4.5426E-191	7213	869.3388967
P04264	KRT1	rs117006546	12	52865871	T	0.30971	3.33225E-11	7213	43.97130745
P04278	SHBG	rs11651783	17	7544593	T	-0.306614	7.83088E-52	7213	229.4567792
P04424	ASL	rs28412533	7	66531410	G	0.21655	2.09005E-22	7213	94.81529031
P04745	AMY1A	rs78811372	1	103579772	A	-0.712255	8.60545E-63	7213	279.7284413
P04746	AMY2A	rs12076610	1	103521319	T	0.689138	1.4721E-133	7213	604.8543507
P04792	HSPB1	rs2868371	7	76301442	G	-0.849624	1E-200	7213	1373.872631
P04843	RPN1	rs9880064	3	128636666	A	0.418723	2.6542E-137	7213	622.0681716
P05060	CHGB	rs36054946	20	5886044	C	-0.410316	8.92004E-11	7213	42.04492478
P05067	APP	rs455047	21	26151670	C	0.16472	5.6768E-23	7213	97.39574111
P05121	SERPINE1	rs2227631	7	101126257	G	-0.185799	3.08949E-28	7213	121.421755
P05154	SERPINA5	rs10133793	14	94584813	C	0.155602	9.69081E-19	7213	78.12120383
P05155	SERPING1	rs10896631	11	57613790	C	-0.9075	1E-200	7213	1373.872631
P05156	CFI	rs13117504	4	109737700	G	0.386073	5.3092E-120	7213	542.5300292
P05160	F13B	rs1332668	1	197036606	T	-0.504522	1E-200	7213	965.8941208
P05161	ISG15	rs3128111	1	995371	G	0.307143	4.97268E-73	7213	326.7227359
P05162	LGALS2	rs2235338	22	37569873	G	0.290398	2.58988E-67	7213	300.4796152
P05164	MPO	rs75394768	17	58336274	A	-0.523014	3.74913E-54	7213	240.0952789
P05187	ALPP	rs35458538	2	232439642	A	0.393209	1.94999E-92	7213	415.8533125
P05362	ICAM1	rs5498	19	10285007	G	-1.04398	1E-200	7213	1373.872631
P05451	REG1A	rs11126696	2	79096762	A	-0.294665	7.84649E-67	7213	298.2700379
P05452	CLEC3B	rs10865936	3	44995308	A	-0.356188	4.78769E-72	7213	322.20722
P05546	SERPIND1	rs73160986	22	20752297	C	0.150383	1.00467E-08	7213	32.83219623
P05771	PRKCB	rs2023671	16	23838518	G	0.29896	1.70985E-56	7213	250.8324557
P06132	UROD	rs2236576	1	45012003	A	-0.796385	1E-200	7213	1373.872631
P06213	INSR	rs3745545	19	7211830	C	-0.139215	1.42225E-09	7213	36.63809433
P06241	FYN	rs9487731	6	111833645	C	-0.142789	2.6777E-11	7213	44.39939382
P06276	BCHE	rs62295996	3	165764276	A	-0.438648	2.6333E-104	7213	470.3918834
P06396	GSN	rs78573713	9	121248418	C	-1.0449	2.6194E-35	7213	153.7553181
P06702	S100A9	rs724781	1	153363542	G	-0.154398	2.25878E-16	7213	67.36273731
P06703	S100A6	rs60969679	1	153546477	A	0.518715	3.07965E-34	7213	148.8583594
P06727	APOA4	rs12721043	11	116821577	A	-1.19031	3.9527E-52	7213	230.8182656
P06734	FCER2	rs62110713	19	7689659	T	-0.364279	2.14051E-78	7213	351.362134
P06737	PYGL	rs12717412	14	50935708	A	-0.302739	1.56968E-70	7213	315.2489295
P07093	SERPINE2	rs13412535	2	224010157	A	-0.92683	1E-200	7213	1373.872631
P07108	DBI	rs6714264	2	119390517	C	-0.529076	6.6974E-146	7213	661.6021556
P07148	FABP1	rs2241883	2	88124547	C	-0.282662	1.20407E-59	7213	265.2937366
P07203	GPX1	rs9823546	3	49668079	A	-0.16557	1.29437E-19	7213	82.09901652
P07225	PROS1	rs9826711	3	93917625	C	-0.439497	1.0504E-16	7213	68.87248774
P07237	P4HB	rs56112103	17	82336294	A	-0.198497	3.28016E-33	7213	144.1586934
P07306	ASGR1	rs62061425	17	7169772	G	-0.243888	4.21387E-31	7213	134.5156481
P07327	ADH1A	rs28364331	4	99280138	G	-1.42062	3.14015E-94	7213	424.0912365
P07333	CSF1R	rs572474264	5	150110043	A	-0.887257	2.95254E-36	7213	158.0935686
P07355	ANXA2	rs8033800	15	60396980	T	0.397233	3.3497E-122	7213	552.6431232
P07359	GP1BA	rs1060431	17	4937573	A	0.368647	4.60812E-33	7213	143.4834697
P07360	C8G	rs7862602	9	136946019	G	0.497576	1E-200	7213	968.7211937
P07451	CA3	rs1543852	8	85440529	A	-0.288002	2.76894E-64	7213	286.5774522
P07477	PRSS1	rs62473563	7	142771005	T	0.229115	3.2944E-15	7213	62.08205416
P07478	PRSS2	rs1799886	7	142800839	C	0.390392	1.6722E-123	7213	558.6269997
P07585	DCN	rs3138190	12	91171750	G	-0.227633	1.60177E-11	7213	45.40558468
P07602	PSAP	rs4747200	10	71814648	G	0.222778	1.35488E-33	7213	145.9151033
P07686	HEXB	rs13164140	5	74734513	A	0.46273	7.3945E-132	7213	597.034096
P07738	BPGM	rs4732044	7	134656210	A	0.112028	2.6048E-11	7213	44.45343156
P07741	APRT	rs535070	16	88812323	G	-0.110548	3.22633E-10	7213	39.53257158
P07858	CTSB	rs709821	8	11845085	C	0.73774	1E-200	7213	1373.872631
P07948	LYN	rs1050855	8	55880027	A	-0.146682	1.2588E-18	7213	77.60454697
P07949	RET	rs2506008	10	43084776	C	-0.509789	1.5107E-163	7213	742.7529642
P07954	FH	rs10926507	1	241531114	A	-0.188048	4.05384E-24	7213	102.6230744

P07988	SFTPB	rs1130866	2	85666618	G	0.516735	1E-200	7213	1038.581498
P07998	RNASE1	rs17254387	14	20812519	G	-0.423488	6.0647E-128	7213	579.0404326
P08069	IGF1R	rs7162314	15	98946687	G	0.239139	9.70556E-32	7213	137.431046
P08118	MSMB	rs2446707	10	46150926	T	-0.438995	1.6758E-151	7213	687.3607756
P08134	RHOC	rs7415820	1	112626827	G	0.220126	8.1081E-40	7213	174.396856
P08174	CD55	rs11584921	1	207268630	G	-0.796334	1E-200	7213	1373.872631
P08217	CELA2A	rs2473355	1	15423721	G	-0.112078	4.49965E-11	7213	43.38350612
P08236	GUSB	rs2949690	7	66018255	T	-0.177169	1.57144E-26	7213	113.6286915
P08237	PFKM	rs3742074	12	48063602	T	0.143149	1.8151E-10	7213	40.65620448
P08246	ELANE	rs56283881	19	893183	T	-0.238824	1.20812E-36	7213	159.8697335
P08253	MMP2	rs243837	16	55498847	T	-0.11658	7.20037E-12	7213	46.97212061
P08263	GSTA1	rs4715316	6	52764200	C	-0.762353	1E-200	7213	1373.872631
P08294	SOD3	rs1799895	4	24800212	G	1.81962	4.5155E-110	7213	496.8897284
P08319	ADH4	rs6858148	4	99144766	C	-0.431801	9.0145E-144	7213	651.812444
P08397	HMBS	rs72995454	11	119060830	T	0.448389	2.0275E-18	7213	76.6631647
P08476	INHBA	rs1122291	7	41708910	T	-0.112634	6.64634E-09	7213	33.63564955
P08493	MGP	rs2900342	12	14876664	G	-0.428371	8.0186E-145	7213	656.6443897
P08567	PLEK	rs1867312	2	68392849	C	-0.443531	3.9212E-159	7213	722.4522661
P08571	CD14	rs5744441	5	140637262	A	-0.309891	2.2511E-59	7213	264.0469892
P08581	MET	rs41748	7	116806519	G	-0.195354	1.21187E-31	7213	136.9901128
P08603	CFH	rs1048663	1	196705852	A	-0.601286	2.6021E-167	7213	760.0632148
P08637	FCGR3A	rs396991	1	161544752	C	0.969359	1E-200	7213	1373.872631
P08670	VIM	rs359276	10	17271304	A	-0.111299	9.16023E-10	7213	37.49596481
P08697	SERPINF2	rs11078596	17	1714968	T	-0.349685	1.26748E-59	7213	265.1914729
P08709	F7	rs6046	13	113118845	A	-1.29858	1E-200	7213	1373.872631
P08887	IL6R	rs4129267	1	154453788	T	1.04115	1E-200	7213	1373.872631
P08949	NMB	rs35127183	15	84672508	A	-0.238447	2.36051E-43	7213	190.592483
P09038	FGF2	rs308403	4	122836593	T	-0.221641	2.34177E-36	7213	158.5542123
P09104	ENO2	rs2886400	12	6901816	T	0.196188	1.96374E-26	7213	113.1868053
P09110	ACAA1	rs2229528	3	38125604	G	-0.714983	2.91962E-71	7213	318.6024207
P09211	GSTP1	rs1695	11	67585218	G	-0.582943	1E-200	7213	1199.844706
P09228	CST2	rs4260306	20	23749699	T	-0.512363	6.4013E-177	7213	804.2581387
P09237	MMP7	rs11568819	11	102530902	A	0.795426	6.6853E-124	7213	560.4574042
P09238	MMP10	rs17860955	11	102778751	C	-1.07655	2.55651E-65	7213	291.3259433
P09326	CD48	rs352684	1	160711985	A	-0.353888	9.1654E-101	7213	454.1169913
P09341	CXCL1	rs1366946	4	73872752	C	0.626211	1E-200	7213	1031.123987
P09417	QDPR	rs7661303	4	17517297	T	-0.739172	1E-200	7213	1373.872631
P09455	RBP1	rs2071387	3	139538761	G	0.20106	5.63784E-21	7213	88.29525963
P09467	FBP1	rs2987899	9	94596959	A	-0.461261	6.33002E-43	7213	188.6298759
P09486	SPARC	rs59311424	5	152030389	A	-0.386049	6.92479E-16	7213	65.15456348
P09488	GSTM1	rs115929572	1	109703431	A	0.767389	2.2126E-136	7213	617.8338185
P09525	ANXA4	rs2228203	2	69806452	T	-0.51625	1.8901E-140	7213	636.5398283
P09529	INHBB	rs17050272	2	120548864	A	0.421393	2.0356E-145	7213	659.3822174
P09543	CNP	rs4432296	17	41966789	C	-0.284189	1.29856E-52	7213	233.0350739
P09619	PDGFRB	rs2304058	5	150128981	C	-1.048	1E-200	7213	1373.872631
P09622	DLD	rs3735602	7	107923141	A	-0.0968738	1.10057E-08	7213	32.65498123
P09668	CTSH	rs2289702	15	78944951	T	-1.26725	1E-200	7213	1373.872631
P09848	LCT	rs191079	2	135876201	C	-0.80806	1E-200	7213	1373.872631
P09871	C1S	rs12146727	12	7063032	A	-1.05667	1E-200	7213	1373.872631
P09960	LTA4H	rs2247570	12	96028599	C	0.154682	6.99756E-18	7213	74.21726547
P09972	ALDOC	rs141921160	17	28576910	T	-0.841634	2.57819E-24	7213	103.5196905
P0C6S8	LINGO3	rs117849041	19	2312904	T	-0.166257	7.80102E-10	7213	37.80926998
P0C862	C1QTNF9	rs56069023	13	24357997	G	-0.341222	1.65111E-82	7213	370.2499329
P0C8F1	PATE4	rs665677	11	125882266	A	0.277842	3.55189E-53	7213	235.6168703
P0CG30	GSTT2B	rs5751777	22	23924860	C	0.146477	5.99714E-18	7213	74.52183121
P0DJI8	SAA1	rs11024589	11	18256876	C	-0.798865	1E-200	7213	929.8436197
P0DJI9	SAA2	rs11024589	11	18256876	C	-0.578967	5.7678E-105	7213	473.4225146
P0DMM9	SULT1A3	rs148788997	16	30311847	C	0.185788	6.31456E-13	7213	51.7466149
P10082	PYY	rs12449853	17	44049191	C	0.138133	3.48411E-11	7213	43.8840818
P10092	CALCB	rs11023416	11	15052326	G	-0.300296	1.98029E-58	7213	259.7145902
P10124	SRGN	rs2229498	10	69097096	G	-0.50051	3.5307E-110	7213	497.3808055
P10147	CCL3	rs2015086	17	36064257	G	0.609656	2.9659E-147	7213	667.8270337
P10153	RNASE2	rs2233859	14	20891649	A	0.304277	7.02617E-77	7213	344.3996697
P10253	GAA	rs2304849	17	80101663	C	-0.376537	6.49905E-89	7213	399.6696403
P10451	SPP1	rs1471400	4	87853095	A	-0.102178	4.44361E-09	7213	34.41901388
P10619	CTSA	rs191805	20	45880903	T	0.22453	3.92051E-39	7213	171.2628991
P10643	C7	rs74480769	5	40972109	G	-1.50019	1E-200	7213	1159.078094
P10645	CHGA	rs729940	14	92932756	T	-0.326871	2.2892E-44	7213	195.2351797
P10646	TFPI	rs116011690	2	187459732	T	-0.557414	1.10227E-22	7213	96.081917
P10696	ALPG	rs35458538	2	232439642	A	0.325131	3.18307E-63	7213	281.7105291
P10720	PF4V1	rs872914	4	73852384	G	0.122594	2.10988E-11	7213	44.8660165
P10746	UROS	rs1935451	10	125816857	G	0.270185	1.34066E-19	7213	82.02956745
P10768	ESD	rs9778	13	46779966	T	-0.843938	1E-200	7213	970.4149138
P10912	GHR	rs4866942	5	42641860	T	0.338674	7.29338E-94	7213	422.4097976
P10915	HAPLN1	rs6886442	5	83677391	C	-0.172545	2.52028E-19	7213	80.78212033
P11171	EPB41	rs204074	1	28865051	C	-0.295365	3.43895E-20	7213	84.71919816
P11226	MBL2	rs7899547	10	52777079	T	-0.775361	1E-200	7213	1373.872631

P11310	ACADM	rs7534754	1	75719623	A	0.316964	4.61335E-71	7213	317.6902684
P11362	FGFR1	rs11997244	8	38701808	T	-0.148486	5.37886E-19	7213	79.28418348
P11464	PSG1	rs2005772	19	42881078	G	-0.142168	2.70615E-12	7213	48.89081539
P11586	MTHFD1	rs10146204	14	64352051	A	-0.189337	6.03146E-29	7213	124.6630266
P11717	IGF2R	rs2282140	6	160073489	T	0.870276	1E-200	7213	1373.872631
P11766	ADH5	rs28730611	4	99079107	C	-0.980676	2.0033E-115	7213	521.4929343
P12081	HARS1	rs112352450	5	140782616	A	0.463388	1.37033E-14	7213	59.27606765
P12104	FABP2	rs1799883	4	119320747	T	-0.922963	1E-200	7213	1373.872631
P12109	COL6A1	rs1053312	21	46003475	A	-1.07583	1E-200	7213	1373.872631
P12110	COL6A2	rs35548026	21	46132295	A	-0.548396	1.01031E-78	7213	352.8594791
P12111	COL6A3	rs1050785	2	237324109	C	-0.220663	5.2035E-39	7213	170.6999347
P12259	F5	rs61808983	1	169505159	T	-0.831266	1.1357E-107	7213	485.8570484
P12273	PIP	rs73170678	7	143139110	T	0.580026	9.6403E-132	7213	596.5045599
P12318	FCGR2A	rs1801274	1	161509955	A	-0.989651	1E-200	7213	1373.872631
P12532	CKMT1A	rs2614819	15	43604248	A	-0.260674	2.50916E-21	7213	89.89677255
P12544	GZMA	rs4865901	5	54993327	G	0.111919	2.75021E-09	7213	35.35316703
P12724	RNASE3	rs147307766	14	20917832	T	0.946463	1.0718E-141	7213	642.2706229
P12814	ACTN1	rs76640185	14	69092813	C	-0.36557	8.31867E-16	7213	64.79317686
P12821	ACE	rs4353	17	63493061	G	-0.744398	1E-200	7213	1373.872631
P13385	TDGF1	rs80045772	3	46577748	A	1.13312	1E-200	7213	1373.872631
P13497	BMP1	rs1126931	8	22164420	T	-0.198104	3.33053E-19	7213	80.23127936
P13501	CCL5	rs2107538	17	35880776	T	-0.612013	1.0967E-183	7213	835.3797495
P13591	NCAM1	rs2288158	11	113262954	G	0.459764	4.02123E-80	7213	359.2892006
P13647	KRT5	rs636676	12	52513634	C	-0.134218	6.50119E-13	7213	51.68942721
P13671	C6	rs191791682	5	41173883	C	-1.52568	1.66219E-91	7213	411.5778039
P13686	ACP5	rs2305799	19	11576536	T	-0.444933	7.55826E-71	7213	316.7059787
P13693	TPT1	rs2234222	13	45339402	C	0.274344	4.27742E-13	7213	52.5114738
P13716	ALAD	rs1800435	9	113391611	G	-0.67226	2.3064E-109	7213	493.634756
P13796	LCP1	rs12858505	13	46124637	A	-0.16006	2.38419E-15	7213	62.71888141
P13929	ENO3	rs238238	17	4953081	A	0.359933	2.33029E-89	7213	401.7158853
P13987	CD59	rs831630	11	33721473	T	-0.394336	4.1696E-112	7213	506.2409083
P14151	SELL	rs4987353	1	169697846	A	-0.583599	1E-200	7213	1132.980653
P14174	MIF	rs2330634	22	23908608	C	0.274135	7.72051E-60	7213	266.1792532
P14207	FOLR2	rs75608470	11	72216505	T	0.393716	1.13984E-13	7213	55.10975141
P14210	HGF	rs5745695	7	81728759	G	-0.240622	4.21989E-35	7213	152.8076946
P14314	PRKCSH	rs11557488	19	11447460	A	0.287358	1.91952E-45	7213	200.1678991
P14384	CPM	rs8181716	12	69024494	T	-0.221862	1.26884E-24	7213	104.924428
P14415	ATP1B2	rs1642762	17	7651454	C	0.431073	7.6791E-152	7213	688.9192537
P14543	NID1	rs2734807	1	236098248	T	-0.226066	1.50476E-41	7213	182.3265062
P14550	AKR1A1	rs2229540	1	45566639	G	-1.41296	1E-200	7213	1373.872631
P14555	PLA2G2A	rs11573156	1	19979653	C	0.813111	1E-200	7213	1373.872631
P14598	NCF1	rs1384976112	7	75164328	A	1.09623	3.291E-158	7213	718.2034201
P14621	ACYP2	rs11125524	2	54152396	C	0.383966	1.0186E-122	7213	555.0198287
P14625	HSP90B1	rs2722192	12	103952301	C	1.06302	1E-200	7213	1373.872631
P14778	IL1R1	rs2287047	2	102157594	A	-0.230468	2.10963E-37	7213	163.3387999
P14780	MMP9	rs8113877	20	46006406	G	0.276869	1.112E-60	7213	270.0403975
P14784	IL2RB	rs228953	22	37135396	A	0.154667	1.58536E-20	7213	86.25039166
P14868	DARS1	rs2304371	2	135803987	G	0.418086	1.06251E-84	7213	380.3152197
P15086	CPB1	rs13318851	3	148844610	C	0.409447	3.23915E-98	7213	442.4077462
P15090	FABP4	rs1486006	8	81478382	C	-0.172477	2.25663E-15	7213	62.82718098
P15104	GLUL	rs61805076	1	182185855	C	0.11505	9.54394E-11	7213	41.91272724
P15121	AKR1B1	rs782546	7	134502831	T	0.645917	6.92574E-41	7213	179.2898974
P15144	ANPEP	rs1042499	15	89785074	C	0.21923	5.29933E-10	7213	38.56377961
P15248	IL9	rs1799962	5	135896167	C	-0.181859	2.04247E-08	7213	31.45375726
P15259	PGAM2	rs6956492	7	44064620	G	-0.129707	3.76011E-11	7213	43.73487621
P15291	B4GALT1	rs7019909	9	33113324	T	0.630544	4.1064E-121	7213	547.639673
P15309	ACP3	rs61793793	3	132338990	A	0.225928	1.1975E-09	7213	36.97343012
P15498	VAV1	rs36097961	19	6850756	T	-0.580725	7.985E-80	7213	357.9210409
P15516	HTN3	rs78169960	4	70032094	G	-0.697929	6.8602E-21	7213	87.90709954
P15529	CD46	rs2488252	1	207764883	C	0.593582	1E-200	7213	1366.80757
P15559	NQO1	rs57488237	16	69707668	C	-0.790558	1E-200	7213	1373.872631
P15586	GNS	rs1690278	12	64739864	G	-0.109823	7.43088E-11	7213	42.40213709
P15692	VEGFA	rs6921438	6	43957870	A	-0.216499	2.67463E-38	7213	167.4448373
P15814	IGLL1	rs9624216	22	23580365	A	-0.5173	5.88513E-71	7213	317.204839
P15848	ARSB	rs11743362	5	78880898	A	-0.262654	1.0149E-55	7213	247.2846496
P16035	TIMP2	rs11077399	17	78868093	A	-0.128605	1.49154E-12	7213	50.05949897
P16083	NQO2	rs6596928	6	2998115	T	-0.645136	1E-200	7213	1373.872631
P16109	SELP	rs6136	1	169594713	G	-0.643764	6.7045E-134	7213	606.424811
P16112	ACAN	rs34949187	15	88843421	A	-0.219758	5.68378E-24	7213	101.9535935
P16152	CBR1	rs16993864	21	36074301	A	-1.4996	6.0802E-141	7213	638.8046243
P16234	PDGFRA	rs35597368	4	54273604	C	-0.282808	1.24608E-26	7213	114.0886989
P16435	POR	rs59882870	7	76009103	A	0.172892	2.47259E-14	7213	58.1148007
P16442	ABO	rs8176632	9	133277095	T	1.11785	1E-200	7213	1373.872631
P16444	DPEP1	rs409170	16	89615942	A	-0.526387	1E-200	7213	1043.830177
P16519	PCSK2	rs6044716	20	17269967	T	-0.131171	2.51464E-15	7213	62.61396558
P16562	CRISP2	rs555247	6	49742392	A	0.502713	1E-200	7213	955.7808123
P16581	SELE	rs4987388	1	169689445	A	0.156461	1.30183E-17	7213	72.99190032

P16591	FER	rs71592765	5	108755299	G	0.367916	7.38867E-36	7213	156.2704605
P16619	CCL3L1	rs1102934	17	36062554	G	0.464274	2.25854E-85	7213	383.4041734
P16860	NPPB	rs198379	1	11855410	C	0.204607	1.46637E-33	7213	145.7580121
P16871	IL7R	rs10058572	5	35880755	G	0.147757	1.81489E-18	7213	76.88194213
P16885	PLCG2	rs62045706	16	81851810	A	0.280027	5.91315E-43	7213	188.7654145
P16930	FAH	rs11555096	15	80180184	T	-1.65648	9.5365E-193	7213	877.0571745
P16949	STMN1	rs807060	1	25914964	G	-0.0984897	4.58885E-09	7213	34.35641645
P17181	IFNAR1	rs2257167	21	33343393	C	-0.998563	1E-200	7213	1373.872631
P17213	BPI	rs6127742	20	38322148	A	-0.749556	1.3849E-171	7213	779.7197429
P17252	PRKCA	rs61762372	17	66302564	A	0.305938	9.34709E-73	7213	325.4643592
P17301	ITGA2	rs13186378	5	52975201	G	0.143594	7.28101E-16	7213	65.05571463
P17405	SMPD1	rs1050239	11	6394233	A	-0.605083	1E-200	7213	1005.7933
P17516	AKR1C4	rs75811842	10	5219138	G	-1.05255	1E-200	7213	1373.872631
P17535	JUND	rs12610373	19	18287474	A	0.167005	7.44118E-22	7213	92.30192719
P17538	CTRB1	rs8057145	16	75222353	A	-0.614443	2.2888E-195	7213	889.1081212
P17655	CAPN2	rs188530181	1	223691121	C	-1.43837	1.12989E-72	7213	325.086234
P17813	ENG	rs11794565	9	127838998	T	0.241888	1.25914E-48	7213	214.7570143
P17900	GM2A	rs72794132	5	151239432	T	0.706237	1E-200	7213	1064.10061
P17927	CR1	rs679515	1	207577223	T	0.618221	3.3235E-193	7213	879.1629996
P17931	LGALS3	rs118083722	14	55129494	A	-0.80857	2.0924E-175	7213	797.2929014
P17936	IGFBP3	rs148562589	7	45956826	C	1.12319	5.1633E-66	7213	294.5144356
P18065	IGFBP2	rs4674091	2	216535725	T	-0.104725	4.13875E-10	7213	39.04628945
P18075	BMP7	rs6123685	20	57260984	A	0.111039	6.39815E-09	7213	33.70968044
P18085	ARF4	rs141818286	3	57935360	G	0.242484	7.70784E-11	7213	42.3305673
P18428	LBP	rs2232613	20	38369011	T	-1.21273	1E-200	7213	1373.872631
P18510	IL1RN	rs55709272	2	113109711	C	-0.37788	8.2325E-115	7213	518.6717053
P18627	LAG3	rs3782735	12	6775910	G	-0.170626	5.59377E-24	7213	101.9852152
P19021	PAM	rs12656561	5	102989988	C	-0.574245	1E-200	7213	1142.406099
P19174	PLCG1	rs753381	20	41168825	T	-0.153841	2.04847E-20	7213	85.74359187
P19224	UGT1A6	rs887829	2	233759924	T	-0.378993	5.5776E-104	7213	468.8939656
P19256	CD58	rs60612523	1	116517087	G	0.704774	1.95112E-27	7213	117.7658701
P19320	VCAM1	rs141294855	1	100681309	C	-0.128724	1.77673E-10	7213	40.6979552
P19438	TNFRSF1A	rs1800693	12	6330843	C	-0.191908	2.0961E-30	7213	131.3306982
P19801	AOC1	rs10452848	7	150826456	G	0.768101	1E-200	7213	1373.872631
P19823	ITIH2	rs17142907	10	7707923	G	1.35362	1E-200	7213	1373.872631
P19827	ITIH1	rs1042779	3	52786995	G	-0.691912	1E-200	7213	1373.872631
P19835	CEL	rs2075733	9	133072397	G	0.442834	2.90289E-26	7213	112.4118468
P19875	CXCL2	rs1893319	4	74107412	T	0.113199	5.89058E-11	7213	42.85650779
P19957	PI3	rs56168207	20	45175330	T	0.376689	3.49018E-69	7213	309.0652814
P19961	AMY2B	rs12076610	1	103521319	T	0.612807	4.281E-105	7213	474.0174768
P19971	TYMP	rs131805	22	50525724	T	-0.362562	2.87306E-75	7213	336.9994533
P20023	CR2	rs61821130	1	207457543	A	-0.237385	2.42444E-19	7213	80.85873339
P20061	TCN1	rs34324219	11	59855905	A	-0.571434	4.7144E-102	7213	460.0389021
P20062	TCN2	rs5753236	22	30613833	G	0.916242	1E-200	7213	1373.872631
P20073	ANXA7	rs117332875	10	73370976	G	-0.46588	2.52354E-15	7213	62.60700737
P20138	CD33	rs3865444	19	51224706	A	-0.942226	1E-200	7213	1373.872631
P20155	SPINK2	rs7694521	4	56755581	A	0.216886	1.90246E-39	7213	172.7007808
P20231	TPSB2	rs3874639	16	1244821	G	-0.793065	1E-200	7213	1373.872631
P20333	TNFRSF1B	rs519064	1	12182039	T	-1.14125	2.1361E-141	7213	640.8935063
P20339	RAB5A	rs4241540	3	19941480	C	-0.116966	1.28171E-11	7213	45.84222229
P20366	TAC1	rs2072099	7	97733023	T	-0.267924	3.73525E-34	7213	148.4749075
P20591	MX1	rs464783	21	41424553	T	-0.413879	1.4031E-135	7213	614.1456201
P20618	PSMB1	rs3734763	6	170576824	C	-0.529913	1E-200	7213	1035.040772
P20742	PZP	rs1017301	12	9210335	C	-0.508647	2.7109E-189	7213	861.1704277
P20774	OGN	rs10992291	9	92270727	C	0.567692	1E-200	7213	1187.43021
P20783	NTF3	rs67394720	12	5438593	T	-0.190756	1.57703E-11	7213	45.43607207
P20839	IMPDH1	rs1053124	7	128501811	T	0.120469	6.88623E-09	7213	33.56668012
P20849	COL9A1	rs13199337	6	70279372	T	0.143297	5.62031E-11	7213	42.94839125
P21128	ENDOU	rs1153977	12	47641073	T	-0.114451	5.48042E-12	7213	47.50713177
P21217	FUT3	rs708686	19	5840608	T	-0.682224	1E-200	7213	1373.872631
P21246	PTN	rs322329	7	137333710	C	0.293789	8.83179E-69	7213	307.2144407
P21266	GSTM3	rs1292096	1	109710758	A	0.540104	2.2284E-160	7213	728.17983
P21291	CSRP1	rs3767541	1	201486782	C	-0.180995	4.49169E-25	7213	106.982274
P21580	TNFAIP3	rs5029937	6	137874014	T	0.280835	4.45258E-09	7213	34.41508891
P21589	NT5E	rs6903114	6	85696953	T	0.464768	3.7293E-154	7213	699.5589075
P21695	GPD1	rs75732302	12	49959342	T	-0.444382	3.31992E-10	7213	39.4767304
P21709	EPHA1	rs4421280	7	143406124	G	-1.38881	1E-200	7213	1373.872631
P21741	MDK	rs61882743	11	46527204	G	-0.149262	8.53064E-12	7213	46.63987976
P21757	MSR1	rs41341748	8	16155085	A	-1.49438	3.86649E-79	7213	354.7750855
P21781	FGF7	rs12439429	15	49442437	C	-0.183085	2.57638E-28	7213	121.7820801
P21815	IBSP	rs2616262	4	87802058	G	0.116683	1.98456E-12	7213	49.49916671
P21854	CD72	rs75679360	9	35563364	A	0.526913	1.19328E-44	7213	196.5316203
P21860	ERBB3	rs2292238	12	56100038	C	-0.19803	1.0497E-32	7213	141.8482374
P21980	TGM2	rs13039547	20	38169451	C	-0.209571	1.29199E-34	7213	150.5842456
P22079	LPO	rs11656836	17	58257173	T	-0.275333	2.61003E-41	7213	181.2310116
P22309	UGT1A1	rs887829	2	233759924	T	-0.607257	1E-200	7213	1271.263986
P22392	NME2	rs115848216	17	51166498	A	-1.37989	1E-200	7213	1160.114212

P22607	FGFR3	rs79825628	4	1684828	A	0.366185	3.32301E-66	7213	295.392887
P22681	CBL	rs76683040	11	119281974	T	-0.709014	2.3147E-108	7213	489.0317839
P22749	GNLY	rs12151742	2	85705880	T	0.783971	1E-200	7213	1373.872631
P22792	CPN2	rs11711157	3	194341097	T	-0.754011	1E-200	7213	1373.872631
P22894	MMP8	rs11225395	11	102725749	A	0.232217	1.31284E-44	7213	196.3416042
P22897	MRC1	rs561612	10	17848848	A	0.704389	1E-200	7213	1281.687233
P23141	CES1	rs76336259	16	55816819	A	-0.460061	1.29524E-31	7213	136.8580013
P23142	FBLN1	rs11090631	22	45450490	T	-0.343656	1.86312E-60	7213	269.0120007
P23280	CA6	rs3765963	1	8974539	G	0.648589	1E-200	7213	1373.872631
P23381	WARS1	rs4905957	14	100370893	C	-0.632638	1E-200	7213	1158.5818
P23435	CBLN1	rs12596832	16	48971047	A	-1.03161	1E-200	7213	1373.872631
P23515	OMG	rs72813607	17	31134507	A	-0.195295	2.29839E-15	7213	62.79106631
P23560	BDNF	rs75994913	11	27764201	G	0.375336	1.95803E-18	7213	76.73201827
P23588	EIF4B	rs146008363	12	53037514	A	-0.212298	3.1861E-09	7213	35.06665611
P23919	DTYMK	rs142497237	2	241721906	T	0.595972	1.01536E-49	7213	219.7696906
P23921	RRM1	rs1662161	11	4115744	A	0.104714	2.47093E-10	7213	40.05356797
P24158	PRTN3	rs2930903	19	887824	C	-0.146841	7.32069E-18	7213	74.12815038
P24298	GPT	rs4244610	8	144512131	A	-0.137154	1.05779E-16	7213	68.8586615
P24387	CRHBP	rs10942798	5	76913570	C	0.648448	1E-200	7213	1373.872631
P24592	IGFBP6	rs822688	12	53099603	T	-0.221984	1.05911E-20	7213	87.04815118
P24666	ACP1	rs79716074	2	277003	G	1.03283	1E-200	7213	1373.872631
P24752	ACAT1	rs12577790	11	108115500	A	-0.135155	3.82401E-12	7213	48.21271321
P24821	TNC	rs1138545	9	115073620	T	1.19576	1E-200	7213	1373.872631
P25311	AZGP1	rs1981550	7	99944329	A	-0.51842	3.2008E-190	7213	865.4384355
P25445	FAS	rs7911226	10	89009208	G	-0.433387	1.0206E-131	7213	596.3907534
P25774	CTSS	rs41271951	1	150764744	G	-1.09777	1E-200	7213	1326.104652
P26196	DDX6	rs6589677	11	118713262	A	0.156736	1.68564E-09	7213	36.3069233
P26440	IVD	rs12440453	15	40399596	T	0.219407	6.23287E-35	7213	152.0326592
P26447	S100A4	rs79399329	1	153544257	A	0.245223	8.18705E-12	7213	46.72044249
P26639	TARS1	rs3777083	5	33442018	G	-0.216131	5.02706E-25	7213	106.7591122
P26885	FKBP2	rs72920394	11	64222830	A	-0.232746	2.1514E-16	7213	67.45876433
P26927	MST1	rs3197999	3	49684099	A	-1.04869	1E-200	7213	1373.872631
P26992	CNTFR	rs73645429	9	34594286	A	-0.996468	1.05376E-59	7213	265.5594285
P27352	CBLIF	rs150884181	11	59843108	G	-0.5299	8.28676E-11	7213	42.18893412
P27361	MAPK3	rs9932466	16	30130526	T	0.6286	1E-200	7213	1373.872631
P27487	DPP4	rs13015258	2	162074215	T	0.124202	1.72601E-13	7213	54.29430871
P27695	APEX1	rs1130409	14	20456995	G	0.203126	1.31047E-34	7213	150.5560262
P27930	IL1R2	rs2310170	2	101999692	G	-0.521562	1E-200	7213	988.6758772
P28070	PSMB4	rs4603	1	151401549	C	-0.710493	1E-200	7213	1197.907538
P28325	CST5	rs6138152	20	23869493	G	0.729782	1E-200	7213	1225.57948
P28332	ADH6	rs28730611	4	99079107	C	-1.16016	3.8714E-163	7213	740.8734256
P28676	GCA	rs17783344	2	162352383	G	-0.5921	9.7314E-134	7213	605.6808752
P28799	GRN	rs5848	17	44352876	T	-0.24927	5.39553E-42	7213	184.3667916
P28838	LAP3	rs114838597	4	17578371	T	0.520345	2.35653E-23	7213	99.1367618
P29034	S100A2	rs58056804	1	153552230	A	-0.309332	7.26727E-17	7213	69.5990281
P29120	PCSK1	rs6234	5	96393270	C	-1.00052	1E-200	7213	1373.872631
P29218	IMPA1	rs2142316	8	81652967	G	-0.147208	1.62876E-16	7213	68.00748592
P29317	EPHA2	rs924204	1	16187431	A	0.371275	3.8527E-112	7213	506.3986909
P29323	EPHB2	rs2043970	1	22735465	C	-0.8304	4.8801E-170	7213	772.6046697
P29353	SHC1	rs61751623	1	154965738	G	-0.295485	4.7725E-14	7213	56.8213775
P29373	CRABP2	rs3806412	1	156706761	G	-0.54454	1E-200	7213	1104.353785
P29460	IL12B	rs4244437	5	159346109	G	-0.58191	1E-200	7213	1152.942773
P29622	SERPINA4	rs5511	14	94567258	T	0.784962	1E-200	7213	1373.872631
P30041	PRDX6	rs33951697	1	173489187	T	0.513529	3.126E-133	7213	603.3507181
P30043	BLVRB	rs184723794	19	40442161	G	-1.15056	6.25243E-46	7213	202.4002958
P30046	DDT	rs4820571	22	23900786	A	0.218832	2.39995E-38	7213	167.6602927
P30048	PRDX3	rs1810159	10	119195930	A	0.168914	5.15367E-11	7213	43.11797093
P30085	CMPK1	rs35687416	1	47368537	T	-0.653522	1.33668E-62	7213	278.850805
P30086	PEBP1	rs1050625	12	118144672	T	-1.89546	4.081E-144	7213	653.3950186
P30101	PDIA3	rs12900924	15	43737329	T	-0.148016	8.13056E-12	7213	46.73401106
P30405	PPIF	rs12252016	10	79324912	A	-0.453721	1.40349E-12	7213	50.17889774
P30533	LRPAP1	rs16844464	4	3493229	T	-0.979512	1.33482E-95	7213	430.3926738
P30566	ADSL	rs8192455	22	40350207	C	0.129038	4.57544E-08	7213	29.88881795
P30626	SRI	rs76354035	7	88224953	C	0.28658	2.03497E-43	7213	190.887744
P30740	SERPINB1	rs316343	6	2839825	C	-0.302428	3.51492E-75	7213	336.5973619
P30838	ALDH3A1	rs887241	17	19742625	A	-0.216368	5.01911E-36	7213	157.0390055
P31025	LCN1	rs12340697	9	135513873	T	-0.20562	3.58083E-19	7213	80.08809722
P31151	S100A7	rs3014837	1	153458930	C	1.48186	1E-200	7213	1373.872631
P31946	YWHAB	rs6031847	20	44885562	T	-0.895903	1E-200	7213	1373.872631
P31994	FCGR2B	rs6665610	1	161671594	A	1.13568	1E-200	7213	1373.872631
P31997	CEACAM8	rs10420796	19	42569106	G	-0.178236	8.26821E-10	7213	37.69580688
P32019	INPP5B	rs61776662	1	37867155	G	-0.919903	1E-200	7213	1373.872631
P32455	GBP1	rs61798920	1	89051436	T	-0.168436	3.23899E-22	7213	93.94814576
P32456	GBP2	rs10922556	1	89054960	C	-0.161393	1.41758E-15	7213	63.74297737
P32927	CSF2RB	rs2239749	22	36935263	G	-0.69957	1E-200	7213	1373.872631
P32971	TNFSF8	rs3181348	9	114931904	A	-0.244436	1.56806E-48	7213	214.3202112
P33151	CDH5	rs6499080	16	66397516	T	-0.221214	6.28043E-36	7213	156.59344

P33316	DUT	rs6493314	15	48273292	T	-0.15394	2.44735E-10	7213	40.07229888
P34096	RNASE4	rs1888560	14	20696086	T	-0.51182	8.6538E-190	7213	863.4515036
P34810	CD68	rs9901673	17	7580783	A	0.587568	1.5119E-158	7213	719.7568369
P34896	SHMT1	rs4398149	17	18365685	A	-0.662513	1E-200	7213	1373.872631
P35030	PRSS3	rs83921	9	33797058	A	-0.550732	1E-200	7213	1086.688648
P35052	GPC1	rs1126920	2	240466062	T	0.799601	1E-200	7213	967.4521193
P35241	RDX	rs7935163	11	110336594	C	-0.0946592	4.22069E-08	7213	30.04530583
P35247	SFTPD	rs7084667	10	79988952	T	0.521653	1E-200	7213	965.3753814
P35442	THBS2	rs74507247	6	169224675	A	0.945145	1E-200	7213	1251.042536
P35443	THBS4	rs13167730	5	80074424	T	0.654834	5.7024E-119	7213	537.7907344
P35475	IDUA	rs3796622	4	989272	T	-0.623674	1E-200	7213	1373.872631
P35542	SAA4	rs7117890	11	18226956	G	0.590198	1E-200	7213	1373.872631
P35558	PCK1	rs2070756	20	57564347	T	-0.259345	1.46937E-42	7213	186.9544741
P35590	TIE1	rs1556580	1	43304250	T	-0.255506	1.53991E-52	7213	232.6955834
P35625	TIMP3	rs5749506	22	32771237	C	0.667918	1E-200	7213	1373.872631
P35754	GLRX	rs6884979	5	95844596	C	-0.110401	2.01072E-10	7213	40.45621342
P35813	PPM1A	rs183404450	14	60238965	C	-0.270711	2.07323E-10	7213	40.39639773
P35858	IGFALS	rs11641257	16	1822751	C	0.268751	3.71415E-17	7213	70.92314545
P35916	FLT4	rs1130379	5	180612606	T	-0.255045	1.97808E-21	7213	90.36730468
P35968	KDR	rs34231037	4	55106779	G	-1.11205	3.1622E-121	7213	548.1612785
P36222	CHI3L1	rs946259	1	203183049	C	0.834643	1E-200	7213	1373.872631
P36405	ARL3	rs8354	10	102676884	T	-0.741442	2.8682E-149	7213	677.0906854
P36551	CPOX	rs1675513	3	98589854	G	-0.267199	9.74682E-32	7213	137.4226222
P36871	PGM1	rs1126728	1	63631761	T	0.226637	3.97943E-30	7213	130.0581711
P36952	SERPINB5	rs7244879	18	63484141	A	-0.138506	1.04587E-14	7213	59.80781941
P36955	SERPINF1	rs62088172	17	1762959	T	-0.563544	1E-200	7213	1134.330993
P36959	GMPR	rs1042391	6	16290530	A	0.940174	1E-200	7213	1373.872631
P36980	CFHR2	rs4085749	1	196951018	T	-0.879405	1E-200	7213	1373.872631
P37023	ACVRL1	rs78197611	12	51924840	T	0.496669	1.51717E-66	7213	296.9556939
P37235	HPCAL1	rs7581642	2	10337292	T	0.130405	6.50963E-09	7213	33.67607834
P37802	TAGLN2	rs2789422	1	159922298	A	-0.252272	3.03023E-52	7213	231.3475154
P37837	TALDO1	rs10902210	11	733639	A	0.159003	1.64432E-08	7213	31.87485555
P39059	COL15A1	rs7867960	9	98999434	C	0.350249	2.99599E-93	7213	419.590677
P39060	COL18A1	rs144147445	21	45510178	A	-1.20663	1.73178E-70	7213	315.0529914
P39656	DDOST	rs150466875	1	20651922	G	-0.369582	3.89563E-34	7213	148.3913815
P39900	MMP12	rs2276109	11	102875061	C	-0.749039	1E-200	7213	979.4126305
P40121	CAPG	rs62623452	2	85402143	T	-1.7656	2.0553E-134	7213	608.7856405
P40189	IL6ST	rs13183065	5	55977599	A	0.654538	3.2431E-149	7213	676.8453227
P40197	GP5	rs1466733	3	194400269	G	-0.265821	1.14188E-44	7213	196.6192386
P40394	ADH7	rs1573496	4	99428512	G	-0.447271	1.99105E-59	7213	264.2915839
P40763	STAT3	rs4796791	17	42378745	T	-0.428859	2.719E-138	7213	626.6178954
P40818	USP8	rs62016940	15	50425726	G	0.246684	4.85651E-29	7213	125.0929748
P40925	MDH1	rs262505	2	63634652	C	0.340077	9.36708E-76	7213	339.2344045
P40926	MDH2	rs10256	7	76066295	G	0.345975	1.32876E-19	7213	82.0471893
P40967	PMEL	rs3213122	12	55969882	A	0.451595	1.07206E-47	7213	210.4934064
P41217	CD200	rs9823309	3	112311692	T	-0.219756	3.11157E-29	7213	125.9764304
P41222	PTGDS	rs11145948	9	136963460	G	-0.262711	9.44958E-58	7213	256.601105
P41240	CSK	rs34933034	15	74787133	A	0.489076	7.5293E-110	7213	495.8692135
P41271	NBL1	rs3020595	1	19644233	G	-0.223588	1.45418E-31	7213	136.628166
P41439	FOLR3	rs146694036	11	72145571	T	1.02158	1E-200	7213	1373.872631
P42126	ECI1	rs55650311	16	2251268	T	-0.175538	6.10796E-15	7213	60.8664942
P42127	ASIP	rs6059655	20	34077942	A	0.926197	1E-200	7213	993.2512221
P42167	TMPO	rs61932018	12	98573827	G	-0.230861	9.06181E-09	7213	33.03277977
P42226	STAT6	rs703817	12	57096045	C	-0.105123	2.67944E-10	7213	39.89532316
P42330	AKR1C3	rs4880709	10	5109163	G	0.135515	7.69064E-15	7213	60.41292316
P42331	ARHGAP25	rs13413887	2	68769536	A	-0.308586	1.08298E-55	7213	247.1553158
P42574	CASP3	rs72689255	4	184668987	C	-0.728277	2.2042E-193	7213	879.9833646
P42680	TEC	rs4543082	4	48186832	C	0.164147	2.58003E-15	7213	62.56340666
P42701	IL12RB1	rs375947	19	18069641	G	-0.2348	2.94394E-40	7213	176.4117257
P42702	LIFR	rs3729741	5	38496677	T	0.252336	9.36061E-48	7213	210.7634496
P42785	PRCP	rs2229437	11	82853252	G	0.344918	2.092E-57	7213	255.0177764
P43155	CRAT	rs3124499	9	129106712	G	-0.256183	1.13073E-44	7213	196.6387655
P43234	CTSO	rs2334114	4	155935041	A	-0.214937	8.23026E-26	7213	110.3458559
P43251	BTD	rs13078881	3	15645186	C	-1.4571	1E-200	7213	1281.637625
P43405	SYK	rs10993706	9	90840685	G	-0.371118	3.39173E-32	7213	139.5188962
P43628	KIR2DL3	rs4806594	19	54843895	G	0.245625	6.52538E-32	7213	138.2194056
P43652	AFM	rs72856662	4	73505396	T	-1.14918	1.62796E-89	7213	402.4314444
P45452	MMP13	rs655316	11	102961635	T	0.132737	7.4113E-14	7213	55.95595258
P45877	PPIC	rs17388251	5	123024708	C	-0.750978	1E-200	7213	1373.872631
P45984	MAPK9	rs11741142	5	180307139	C	0.340169	4.51469E-62	7213	276.4251846
P46063	RECQL	rs74626198	12	21543317	C	-0.390423	1.23399E-14	7213	59.48230023
P46108	CRK	rs12936625	17	1434769	T	0.20039	1.8743E-31	7213	136.1242228
P46109	CRKL	rs117858197	22	20784951	C	-0.639355	7.4213E-21	7213	87.75159656
P46531	NOTCH1	rs9411207	9	136514099	T	0.188335	4.76907E-27	7213	115.9932917
P47972	NPTX2	rs12673464	7	98655243	C	-0.300661	9.49959E-63	7213	279.531435
P48060	GLIPR1	rs7975447	12	75413201	C	-0.0957356	1.85471E-08	7213	31.64101211
P48061	CXCL12	rs1023264	10	44398308	C	0.176212	8.25634E-23	7213	96.65404809

P48147	PREP	rs1051484	6	105278161	T	0.793538	1E-200	7213	1298.138167
P48304	REG1B	rs11126696	2	79096762	A	-0.324922	3.22404E-81	7213	364.3224401
P48307	TFPI2	rs17165852	7	93896196	G	-0.278301	5.91633E-12	7213	47.35710445
P48357	LEPR	rs2376018	1	65608483	G	-1.25859	1E-200	7213	1373.872631
P48426	PIP4K2A	rs7098978	10	22855287	G	-0.481854	3.0967E-159	7213	722.9237162
P48507	GCLM	rs7521934	1	93939485	T	0.234163	5.19106E-45	7213	198.1880165
P48637	GSS	rs6088642	20	34895383	G	-0.438391	1.1254E-153	7213	697.3530291
P48643	CCT5	rs11557652	5	10256060	T	-0.516992	9.67837E-19	7213	78.12374116
P48723	HSPA13	rs2822638	21	14372785	C	0.26387	5.86373E-44	7213	193.3635488
P48740	MASP1	rs3774275	3	187247480	G	-0.213235	2.36716E-33	7213	144.8066712
P48745	CCN3	rs17793097	8	119453024	G	0.270322	7.59555E-23	7213	96.81921202
P48960	ADGRE5	rs3786656	19	14395852	T	0.122519	2.21769E-11	7213	44.76843592
P49137	MAPKAPK2	rs4256810	1	206717104	C	0.330034	1.53608E-77	7213	347.4317702
P49221	TGM4	rs6650899	3	44900485	G	-0.11859	1.59861E-12	7213	49.92346996
P49223	SPINT3	rs6073773	20	45519311	G	-0.409398	1.6747E-137	7213	622.9877641
P49247	RPIA	rs11686263	2	88811341	A	0.185536	3.01023E-27	7213	116.9058539
P49327	FASN	rs62078746	17	82095714	G	0.179629	9.05779E-28	7213	119.2879633
P49407	ARRB1	rs504683	11	75340189	G	0.5338	1.4802E-198	7213	903.778914
P49588	AARS1	rs2070203	16	70269677	A	0.108285	7.30214E-11	7213	42.4363191
P49589	CARS1	rs445375	11	3034705	A	0.149995	1.95825E-18	7213	76.73179639
P49746	THBS3	rs72704117	1	155205298	T	0.411296	5.36463E-12	7213	47.54899333
P49747	COMP	rs12974746	19	18795739	G	-0.698886	1.62411E-40	7213	177.5946935
P49748	ACADVL	rs446994	17	7213534	C	0.0969528	9.82651E-09	7213	32.87527558
P49767	VEGFC	rs112782083	4	176834019	C	1.07707	6.8596E-42	7213	183.8892014
P49773	HINT1	rs12332117	5	131157469	T	0.314202	6.87008E-61	7213	271.0000151
P49788	RARRES1	rs4680458	3	158724365	G	0.652282	1E-200	7213	1373.872631
P49789	FHIT	rs13070475	3	59983610	G	-0.15566	3.48704E-19	7213	80.14054077
P49840	GSK3A	rs61088131	19	42196795	C	0.124651	4.59412E-08	7213	29.88091829
P49862	KLK7	rs1654523	19	50981938	C	-0.590329	2.32075E-88	7213	397.1303435
P49863	GZMK	rs2407827	5	55011593	T	-0.168677	7.78169E-24	7213	101.331287
P49902	NT5C2	rs12257472	10	103173692	T	0.155142	7.88715E-18	7213	73.9810262
P49914	MTHFS	rs8039272	15	79904408	C	-0.707003	1E-200	7213	1373.872631
P49961	ENTPD1	rs11598475	10	95861509	A	0.292389	2.54009E-63	7213	282.1602384
P50395	GDI2	rs55913768	10	5707486	A	-0.471464	1.3452E-165	7213	752.1828128
P50452	SERPINB8	rs3826616	18	63987229	A	0.428375	4.3167E-150	7213	680.87267
P50454	SERPINH1	rs599816	11	75582937	G	-0.211427	4.10398E-37	7213	162.0159454
P50479	PDLIM4	rs4877	5	132271895	T	-0.449204	2.76838E-56	7213	249.8725466
P50502	ST13	rs138337	22	40835049	G	0.28252	5.46004E-65	7213	289.8134871
P50583	NUDT2	rs10971956	9	34151282	G	-0.761049	1E-200	7213	1373.872631
P50895	BCAM	rs28399656	19	44813447	A	1.51946	1.20751E-85	7213	384.6532539
P50897	PPT1	rs7533094	1	40094014	A	-1.11779	2.1773E-191	7213	870.8080858
P50995	ANXA11	rs2819952	10	80174568	T	-0.625259	1E-200	7213	1373.872631
P51124	GZMM	rs8107361	19	538160	A	-0.209342	3.78986E-11	7213	43.71945266
P51512	MMP16	rs4961090	8	88337442	A	0.144903	1.53976E-16	7213	68.1182875
P51570	GALK1	rs60521621	17	75765315	C	0.161404	2.49406E-11	7213	44.53848564
P51671	CCL11	rs6505397	17	34190977	A	-0.147153	2.59155E-18	7213	76.17844612
P51693	APLP1	rs58291714	19	35891884	C	0.320672	3.8783E-12	7213	48.18507047
P51857	AKR1D1	rs2306847	7	138113847	A	-0.186947	6.63093E-20	7213	83.42113375
P51858	HDGF	rs12145743	1	156730859	G	-0.778949	1E-200	7213	1373.872631
P51884	LUM	rs3741835	12	91111364	G	-0.712224	3.01971E-33	7213	144.3230322
P51911	CNN1	rs138405741	19	11543596	T	-0.325829	8.11042E-12	7213	46.73887139
P51946	CCNH	rs2230641	5	87399457	G	-0.165792	7.51496E-17	7213	69.53292283
P52209	PGD	rs111782199	1	10403410	T	-0.947738	5.82932E-57	7213	252.9761926
P52566	ARHGDIB	rs10444404	12	14961817	G	-0.151537	3.34477E-19	7213	80.22284901
P52594	AGFG1	rs11679534	2	227575661	C	0.413824	2.2647E-122	7213	553.4246075
P52758	RIDA	rs10955148	8	98107592	C	-0.410085	5.7797E-120	7213	542.3605205
P52789	HK2	rs641180	2	74812957	A	0.147991	1.21274E-15	7213	64.05044332
P52790	HK3	rs61749653	5	176889558	T	-0.521669	8.95358E-20	7213	82.82749624
P52797	EFNA3	rs17723260	1	155086187	A	-0.138962	4.41163E-09	7213	34.43307213
P52798	EFNA4	rs3806256	1	155063135	T	0.101451	9.83984E-10	7213	37.35638115
P52799	EFNB2	rs59166663	13	106647317	C	0.278786	3.71131E-32	7213	139.3400714
P52803	EFNA5	rs6596742	5	107602566	A	-0.28344	1.03576E-65	7213	293.1268373
P52823	STC1	rs28588821	8	23836188	T	0.188057	7.57112E-13	7213	51.39031321
P52848	NDST1	rs11746198	5	150543647	T	0.228075	1.24923E-41	7213	182.6967115
P52907	CAPZA1	rs6700673	1	112614432	T	0.136276	3.96401E-16	7213	66.25400749
P53004	BLVRA	rs1050916	7	43791281	G	-1.11345	3.9762E-47	7213	207.8842736
P53367	ARFIP1	rs4619875	4	152779978	T	0.425011	5.6073E-145	7213	657.3587055
P53582	METAP1	rs6822589	4	98980016	A	0.122328	6.15998E-13	7213	51.79527727
P53674	CRYBB1	rs5761621	22	26603394	G	-0.201325	1.05242E-29	7213	128.1278238
P53778	MAPK12	rs3817818	22	50260902	C	0.206294	3.25915E-35	7213	153.3210684
P53814	SMTN	rs7287178	22	31043611	C	0.126388	6.89508E-12	7213	47.05703287
P54252	ATXN3	rs1133441	14	91968721	A	-0.59418	1E-200	7213	1226.853875
P54296	MYOM2	rs3824184	8	2098172	A	-0.172151	3.75556E-13	7213	52.7670135
P54315	PNLIPRP1	rs7906926	10	116597627	T	-0.218421	3.96366E-36	7213	157.5082284
P54317	PNLIPRP2	rs2286779	10	116635040	G	-0.853435	1E-200	7213	1373.872631
P54727	RAD23B	rs11573709	9	107322974	A	0.18305	8.87281E-21	7213	87.39826926
P54756	EPHA5	rs28663440	4	65595644	T	0.403083	1.93412E-37	7213	163.5114762

P54760	EPHB4	rs314345	7	100799705	G	-0.258365	3.02103E-54	7213	240.5253557
P54762	EPHB1	rs10935143	3	134946317	A	0.398707	1.2711E-129	7213	586.7575529
P54764	EPHA4	rs16862777	2	221490753	C	-0.291608	8.95007E-56	7213	247.5350731
P54802	NAGLU	rs41283429	17	42480711	T	1.0998	1.1526E-162	7213	738.6943296
P54826	GAS1	rs4878043	9	87072939	C	-0.155893	9.41886E-21	7213	87.28014734
P54920	NAPA	rs71363738	19	47515000	T	0.582813	2.19576E-37	7213	163.25925
P55001	MFAP2	rs910214	1	16974497	C	0.393641	1.2275E-129	7213	586.8273691
P55058	PLTP	rs111602331	20	45928835	C	-0.738606	1E-200	7213	1285.731853
P55083	MFAP4	rs139356332	17	19385973	C	-0.866868	1.04713E-52	7213	233.4636576
P55103	INHBC	rs2229357	12	57449928	A	-1.16205	1E-200	7213	1373.872631
P55145	MANF	rs4611808	3	51431657	C	-0.356673	8.20662E-50	7213	220.1935553
P55263	ADK	rs12571531	10	73967891	G	0.123916	2.42336E-09	7213	35.59961043
P55773	CCL23	rs712048	17	35999179	A	-0.891227	1E-200	7213	1373.872631
P55774	CCL18	rs2015086	17	36064257	G	0.855831	1E-200	7213	1373.872631
P55854	SUMO3	rs8133302	21	44835300	C	0.216968	9.93431E-28	7213	119.1047371
P56159	GFRA1	rs10885876	10	116202766	G	0.339095	2.77302E-78	7213	350.8458106
P56279	TCL1A	rs78986913	14	95696081	A	-0.492879	3.36906E-35	7213	153.2551582
P56470	LGALS4	rs9304578	19	38759697	A	-0.273497	7.16763E-63	7213	280.0927901
P58062	SPINK7	rs9784645	5	148337418	T	0.293556	6.30026E-56	7213	248.2344151
P58215	LOXL3	rs17010021	2	74534412	A	-0.290539	5.62863E-11	7213	42.94549731
P58335	ANTXR2	rs10009674	4	79880234	A	0.379264	3.82647E-91	7213	409.9142212
P58499	FAM3B	rs57529409	21	41346335	T	-1.08511	1E-200	7213	919.803361
P58753	TIRAP	rs8177399	11	126290931	T	-1.35517	1.5498E-143	7213	650.7303192
P59901	LILRA4	rs12976217	19	54338192	T	0.4063	3.6362E-124	7213	561.6731878
P60022	DEFB1	rs2741117	8	6868447	G	-0.416227	2.8126E-144	7213	654.1383478
P60174	TPI1	rs2071066	12	6868111	C	-0.353781	9.39221E-86	7213	385.1544881
P60520	GABARAPL2	rs2454867	16	75522922	C	-0.303645	6.64678E-32	7213	138.1828001
P61086	UBE2K	rs58339883	4	39781456	C	0.265989	3.10447E-27	7213	116.8447154
P61088	UBE2N	rs2291266	12	93426357	A	-0.400189	1.26912E-74	7213	334.0371681
P61244	MAX	rs762810	14	65077649	A	-0.39197	1.7359E-117	7213	530.971819
P61457	PCBD1	rs72816586	10	70826163	T	-0.714534	9.25055E-65	7213	288.7626412
P61647	ST8SIA6	rs11595070	10	17448760	C	-0.371752	9.98468E-17	7213	68.97248466
P61981	YWHAG	rs73140051	7	76335899	A	-0.118233	1.40728E-10	7213	41.15354209
P62820	RAB1A	rs7607039	2	65096216	A	-0.251404	1.48013E-40	7213	177.7793259
P62937	PPIA	rs62460480	7	44775017	G	-0.387659	1.55965E-33	7213	145.6354989
P62993	GRB2	rs9889402	17	75368135	G	-0.133859	2.99157E-13	7213	53.21375277
P63000	RAC1	rs4724795	7	6338205	A	0.151851	1.00776E-16	7213	68.95421594
P63241	EIF5A	rs11658072	17	7311505	C	0.195134	2.76556E-30	7213	130.7805053
P63279	UBE2I	rs2401949	16	1296685	A	-0.261959	4.74837E-45	7213	198.3654037
P63302	SELENOW	rs11879299	19	47777350	T	-0.311165	1.8643E-78	7213	351.6376714
P68106	FKBP1B	rs72781698	2	24048636	T	-0.630194	1E-200	7213	1023.213693
P78314	SH3BP2	rs7695151	4	2770803	T	0.176218	1.9152E-26	7213	113.2364319
P78324	SIRPA	rs6136376	20	1915598	G	-1.05266	1E-200	7213	1373.872631
P78333	GPC5	rs342706	13	91764804	C	-0.686856	1E-200	7213	1373.872631
P78417	GSTO1	rs641071	10	104268399	T	-0.930001	1E-200	7213	1373.872631
P78423	CX3CL1	rs62037115	16	57424884	A	1.11873	2.05737E-42	7213	186.2848355
P78504	JAG1	rs2423507	20	10633691	G	0.108984	3.61923E-10	7213	39.30817421
P78560	CRADD	rs60169972	12	93837506	C	-0.149609	2.03768E-12	7213	49.44734619
P80075	CCL8	rs3138037	17	34320725	C	-1.12245	1E-200	7213	1373.872631
P80098	CCL7	rs3138037	17	34320725	C	-0.659528	1E-200	7213	914.4748705
P80162	CXCL6	rs16850073	4	73838282	T	0.624649	1E-200	7213	1373.872631
P80370	DLK1	rs12881545	14	100709875	G	-0.484803	1.4794E-176	7213	802.5848291
P80511	S100A12	rs3014874	1	153365467	A	-0.278032	1.66768E-48	7213	214.1975898
P82980	RBP5	rs2290239	12	7123655	G	-0.378575	7.9087E-104	7213	468.1970464
P84157	MXRA7	rs4789345	17	76692234	T	0.496034	6.0443E-194	7213	882.5681063
P98066	TNFAIP6	rs2278089	2	151290158	G	0.45787	3.2821E-172	7213	782.5955832
P98160	HSPG2	rs12742444	1	21854230	T	0.731954	7.08969E-97	7213	436.2498684
Q00796	SORD	rs56060952	15	45036783	C	0.467346	4.35189E-76	7213	340.7631167
Q00872	MYBPC1	rs80087033	12	101654669	A	-0.202723	3.785E-19	7213	79.97853143
Q00887	PSG9	rs4802159	19	43189921	T	-0.550983	4.89953E-57	7213	253.3223579
Q00888	PSG4	rs4802160	19	43190048	G	0.713441	1E-200	7213	1373.872631
Q01344	IL5RA	rs77400868	3	3109280	G	0.500071	2.1473E-99	7213	447.8229852
Q01638	IL1RL1	rs13020553	2	102315366	G	-0.653433	1E-200	7213	1373.872631
Q01973	ROR1	rs2224876	1	64148805	G	-0.653146	1E-200	7213	936.5112597
Q01974	ROR2	rs10118816	9	91929196	T	0.34275	7.11433E-84	7213	376.5222367
Q02083	NAAA	rs28451731	4	75934616	T	-0.815525	1E-200	7213	1373.872631
Q02223	TNFRSF17	rs387871	16	11958626	A	-0.167918	2.53448E-24	7213	103.5535675
Q02246	CNTN2	rs3753847	1	205045411	T	0.84936	1E-200	7213	1373.872631
Q02487	DSC2	rs1789064	18	31094439	A	0.371646	5.34985E-83	7213	372.4978409
Q02750	MAP2K1	rs77428206	15	66484142	A	0.179974	2.57891E-10	7213	39.97001833
Q02763	TEK	rs35030851	9	27197488	T	0.859212	1.4454E-101	7213	457.8030911
Q02790	FKBP4	rs56196860	12	2799164	A	-1.06599	1.123E-105	7213	476.6882429
Q02818	NUCB1	rs28450126	19	48904758	A	-0.385366	2.4256E-52	7213	231.7907368
Q03013	GSTM4	rs687643	1	109669912	C	-0.391214	1.3292E-125	7213	568.2794338
Q03154	ACY1	rs148804382	3	52078217	A	-0.679851	5.13997E-20	7213	83.92464765
Q03167	TGFBR3	rs79859654	1	91620183	G	0.189129	7.52489E-21	7213	87.72417842
Q03403	TFF2	rs58206891	21	42354303	G	-0.125366	5.26202E-09	7213	34.09002945

Q03405	PLAUR	rs2302524	19	43652320	C	-0.415355	1.04874E-79	7213	357.3773332
Q03591	CFHR1	rs7519758	1	196856157	T	-0.939936	1E-200	7213	1373.872631
Q03692	COL10A1	rs9488842	6	116123005	C	-0.362481	1.9009E-84	7213	379.1548727
Q04609	FOLH1	rs55728336	11	49156256	C	0.341771	2.31156E-19	7213	80.9529519
Q04756	HGFAC	rs2498323	4	3449382	A	-1.33214	1E-200	7213	1373.872631
Q04760	GLO1	rs12209477	6	38702023	G	-0.194455	2.91711E-30	7213	130.6746029
Q04917	YWHAH	rs5994442	22	31875946	T	-0.106774	1.55824E-08	7213	31.97930264
Q06033	ITIH3	rs2535629	3	52799203	A	0.684643	1E-200	7213	1373.872631
Q06141	REG3A	rs114874655	2	79162850	A	0.465378	9.04849E-40	7213	174.1786255
Q06418	TYRO3	rs2588323	15	41549880	C	0.25169	5.23488E-44	7213	193.5892778
Q06481	APLP2	rs61349706	11	130094940	C	0.409769	5.29322E-29	7213	124.9221072
Q06520	SULT2A1	rs296384	19	47867143	G	-0.364053	2.35372E-56	7213	250.1957944
Q06828	FMOD	rs4971252	1	203351280	G	-0.440737	1.46131E-71	7213	319.9823658
Q06830	PRDX1	rs11211133	1	45525407	A	0.357692	1.27587E-73	7213	329.4351791
Q07075	ENPEP	rs33966350	4	110510288	A	-1.82228	5.9282E-148	7213	671.0423653
Q07325	CXCL9	rs884304	4	76014110	A	-0.115804	3.17451E-10	7213	39.5641921
Q07507	DPT	rs1018454	1	168728523	A	-0.431681	1.2117E-152	7213	692.6068476
Q07820	MCL1	rs190596489	1	150589567	C	-1.31999	7.78804E-72	7213	321.2371344
Q07866	KLC1	rs2273175	14	103693804	C	0.110967	2.25731E-10	7213	40.23020518
Q07960	ARHGAP1	rs11039024	11	46901617	T	-0.138597	8.28483E-09	7213	33.20708076
Q08257	CRYZ	rs3819946	1	74710202	C	1.07253	1E-200	7213	1373.872631
Q08334	IL10RB	rs2515717	21	33289977	A	0.233191	8.79964E-45	7213	197.1377318
Q08380	LGALS3BP	rs3826311	17	78975444	C	0.218327	6.79447E-24	7213	101.6000173
Q08431	MFGE8	rs34239095	15	88912537	G	0.442825	1.3337E-130	7213	591.2590347
Q08708	CD300C	rs62087200	17	74463637	A	-0.764918	1.27575E-90	7213	407.5117178
Q08752	PPID	rs17843929	4	158715961	T	0.879733	1E-200	7213	1373.872631
Q08830	FGL1	rs3739406	8	17882029	T	-0.72243	1E-200	7213	1373.872631
Q08ET2	SIGLEC14	rs872629	19	51627385	A	-1.00953	1E-200	7213	1373.872631
Q10469	MGAT2	rs12147470	14	49561954	T	-0.274587	2.90538E-61	7213	272.7149754
Q10588	BST1	rs6842052	4	15717077	G	-1.20899	1E-200	7213	1373.872631
Q11128	FUT5	rs778809	19	5830291	A	-0.509359	1.4017E-192	7213	876.287808
Q11201	ST3GAL1	rs9643300	8	133490905	C	0.357289	9.9972E-102	7213	458.5387891
Q12794	HYAL1	rs116482870	3	50302191	T	-0.362857	5.07925E-27	7213	115.8683272
Q12797	ASPH	rs11775077	8	61685128	A	-0.331558	3.69426E-52	7213	230.9529246
Q12805	EFEMP1	rs3791679	2	55869757	G	0.427352	3.7803E-109	7213	492.6485519
Q12841	FSTL1	rs916510	3	120425010	C	0.168963	1.32532E-19	7213	82.05231276
Q12860	CNTN1	rs11177604	12	40651583	A	0.289771	7.91064E-66	7213	293.6640423
Q12864	CDH17	rs12056840	8	94191166	A	-0.340079	2.39724E-95	7213	429.2243364
Q12884	FAP	rs12477178	2	162209363	A	1.11477	2.04516E-54	7213	241.3024002
Q12907	LMAN2	rs28419182	5	177301399	G	-0.138173	1.02897E-11	7213	46.27252076
Q12913	PTPRJ	rs1566734	11	48123823	C	0.232257	6.04316E-25	7213	106.3942904
Q12918	KLRB1	rs2241006	12	9595613	A	-0.389975	2.6321E-120	7213	543.9308313
Q13011	ECH1	rs2229259	19	38816463	T	0.703511	2.0951E-160	7213	728.302981
Q13018	PLA2R1	rs3749117	2	160028931	T	0.744998	1E-200	7213	1373.872631
Q13045	FLII	rs145840264	17	18258631	T	0.611659	9.8833E-32	7213	137.3950095
Q13046	PSG7	rs1209671921	19	42935542	G	0.18469	6.7731E-17	7213	69.73793148
Q13093	PLA2G7	rs953062	6	46658616	G	0.130824	2.3754E-13	7213	53.66683061
Q13136	PPFIA1	rs12577692	11	70503671	G	-0.133938	6.17622E-14	7213	56.31437593
Q13137	CALCOCO2	rs318100	17	48876268	G	-0.281609	4.57472E-36	7213	157.2232616
Q13231	CHIT1	rs872583	1	203215638	C	-0.97891	1E-200	7213	1373.872631
Q13253	NOG	rs227715	17	56705153	G	-0.17296	1.3083E-18	7213	77.52836588
Q13261	IL15RA	rs8177643	10	5974657	T	0.651228	1E-200	7213	1373.872631
Q13283	G3BP1	rs2964584	5	151763317	C	-0.190465	9.62753E-24	7213	100.9096715
Q13287	NMI	rs6713190	2	151302945	C	-0.195191	3.86036E-32	7213	139.2618739
Q13291	SLAMF1	rs6704124	1	160632458	T	0.116482	4.05216E-11	7213	43.58848685
Q13308	PTK7	rs140927825	6	43082066	T	0.549598	9.98813E-36	7213	155.6713546
Q13426	XRCC4	rs1056503	5	83353158	G	0.31539	2.0175E-33	7213	145.1241739
Q13442	PDAP1	rs28495024	7	99419060	C	-0.178251	1.94356E-12	7213	49.54012188
Q13449	LSAMP	rs1835674	3	116319644	T	-0.348407	3.52783E-58	7213	258.5641169
Q13478	IL18R1	rs11465583	2	102366006	C	0.873465	1E-200	7213	1373.872631
Q13508	ART3	rs4859610	4	76079288	A	0.397073	5.77401E-93	7213	418.2816028
Q13522	PPP1R1A	rs10876568	12	54590778	C	0.148809	3.24696E-13	7213	53.05283511
Q13557	CAMK2D	rs3822295	4	113748360	C	-0.140797	2.68906E-08	7213	30.91985913
Q13586	STIM1	rs1869085	11	3883134	C	-0.122929	7.75091E-12	7213	46.8277246
Q13591	SEMA5A	rs56015217	5	9547721	T	0.939395	1E-200	7213	1140.131255
Q13596	SNX1	rs138194169	15	64107669	C	0.679498	9.16354E-33	7213	142.1180766
Q13740	ALCAM	rs751554	3	105418999	T	-0.350204	1.52651E-41	7213	182.2979597
Q13753	LAMC2	rs2276543	1	183186170	A	0.733742	1E-200	7213	1373.872631
Q13790	APOF	rs2066818	12	56360038	A	-0.602174	5.50813E-76	7213	340.2932586
Q13822	ENPP2	rs13267597	8	119641985	G	-0.191541	2.71962E-27	7213	117.1072099
Q13884	SNTB1	rs6982091	8	120534562	A	-0.120568	1.19563E-11	7213	45.97841657
Q13907	IDI1	rs4880760	10	1048969	G	-0.350284	2.92735E-13	7213	53.25638112
Q14005	IL16	rs4778639	15	81308110	G	-1.40934	1E-200	7213	1373.872631
Q14112	NID2	rs941622	14	52032320	G	-0.507152	1E-200	7213	976.1504162
Q14114	LRP8	rs7542607	1	53270941	G	0.255027	3.07428E-52	7213	231.3187742
Q14126	DSG2	rs1460602	18	31501112	T	-0.350854	7.05133E-97	7213	436.2606944
Q14162	SCARF1	rs2272011	17	1638896	G	0.821118	1E-200	7213	1373.872631

Q14213	EBI3	rs4905	19	4237070	G	-1.04542	1E-200	7213	1373.872631
Q14232	EIF2B1	rs117647122	12	123567177	T	-0.554848	4.28599E-16	7213	66.10007717
Q14242	SELPLG	rs73191242	12	108620180	A	-0.355148	3.1227E-63	7213	281.7486909
Q14392	LRRC32	rs12275758	11	76638585	A	-0.172226	4.10581E-08	7213	30.09881985
Q14393	GAS6	rs6602909	13	113849020	C	-0.405253	1.161E-118	7213	536.3714491
Q14397	GCKR	rs1260326	2	27508073	T	-0.197844	1.35064E-31	7213	136.7748354
Q14435	GALNT3	rs7590559	2	165915153	T	0.0936173	2.29272E-08	7213	31.22936158
Q14507	EDDM3A	rs34552133	14	20747764	T	-0.220957	1.34815E-16	7213	68.38033949
Q14508	WFDC2	rs6032226	20	45485639	T	-0.101732	1.22336E-09	7213	36.93177311
Q14512	FGFBP1	rs73230203	4	15962080	A	0.192551	1.88179E-11	7213	45.09005099
Q14515	SPARCL1	rs7688567	4	87505346	A	0.642186	1E-200	7213	1373.872631
Q14554	PDIA5	rs3804749	3	123114156	C	1.00175	1E-200	7213	1373.872631
Q14558	PRPSAP1	rs9907526	17	76319096	C	0.12222	8.40303E-09	7213	33.1795344
Q14574	DSC3	rs35630063	18	31024408	C	-0.338506	4.84088E-09	7213	34.25236017
Q14624	ITIH4	rs3617	3	52799789	A	0.16143	2.39332E-22	7213	94.54707912
Q14626	IL11RA	rs11575578	9	34656482	A	0.943428	2.7148E-181	7213	824.3697244
Q14653	IRF3	rs10415576	19	49661133	C	-0.452409	2.613E-161	7213	732.460696
Q14677	CLINT1	rs12284	5	157787179	G	-0.246025	7.48457E-25	7213	105.9703778
Q14696	MESD	rs1012540	15	80953751	T	-0.439129	1.03993E-29	7213	128.1515194
Q14749	GNMT	rs2395943	6	42972935	A	0.428481	7.7012E-147	7213	665.9215131
Q14914	PTGR1	rs76781427	9	111566655	C	1.12077	3.2459E-186	7213	847.0111937
Q14956	GPNMB	rs28458177	7	23255253	A	-0.30341	7.49192E-71	7213	316.7235554
Q14C87	TMEM132D	rs7299188	12	129289849	G	0.170914	2.95067E-23	7213	98.69149252
Q14DG7	TMEM132B	rs3825381	12	125652317	T	-0.327018	1.00655E-67	7213	302.3635716
Q15063	POSTN	rs7329947	13	37524954	A	0.240615	1.22593E-45	7213	201.0602451
Q15113	PCOLCE	rs7385804	7	100638347	C	-0.225072	3.48937E-39	7213	171.4945637
Q15172	PPP2R5A	rs11119862	1	212167815	G	0.155083	8.74388E-12	7213	46.59149798
Q15181	PPA1	rs12570967	10	70212594	T	-0.227206	3.40705E-09	7213	34.93609992
Q15198	PDGFRL	rs77670928	8	17596804	T	-0.825112	3.42677E-78	7213	350.4236455
Q15238	PSG5	rs7253874	19	43140467	A	-0.612888	1E-200	7213	1373.872631
Q15389	ANGPT1	rs1461993	8	107072760	A	0.138851	5.19993E-16	7213	65.71910678
Q15399	TLR1	rs5743618	4	38797027	A	0.290832	4.914E-56	7213	248.7294438
Q15465	SHH	rs872723	7	155813050	T	-0.193114	2.14999E-17	7213	72.00181982
Q15485	FCN2	rs57136797	9	134860694	T	-0.723795	1E-200	7213	994.5490173
Q15555	MAPRE2	rs1443641	18	35110792	T	-0.371504	1.7567E-113	7213	512.5624458
Q15582	TGFBI	rs13159365	5	136053744	C	0.66756	1E-200	7213	1373.872631
Q15642	TRIP10	rs8106212	19	6802560	T	0.561283	1.25612E-15	7213	63.98120283
Q15661	TPSAB1	rs35116045	16	1257403	T	0.358361	1.0432E-99	7213	449.2636045
Q15691	MAPRE1	rs414049	20	32831982	C	-0.517638	2.2969E-189	7213	861.5014788
Q15782	CHI3L2	rs11556868	1	111235703	T	0.294902	2.81115E-31	7213	135.3193519
Q15796	SMAD2	rs11874858	18	47931447	A	0.135265	6.69185E-16	7213	65.22199335
Q15797	SMAD1	rs2118438	4	145507232	A	0.12324	3.7003E-09	7213	34.77534472
Q15813	TBCE	rs4469707	1	235407319	T	0.403095	3.0118E-122	7213	552.8554328
Q15818	NPTX1	rs111307499	17	80564758	G	0.670141	7.4846E-115	7213	518.8618331
Q15828	CST6	rs12577165	11	66008958	C	0.616729	2.45323E-31	7213	135.5897603
Q15848	ADIPOQ	rs76071583	3	186840083	G	-0.88664	9.6595E-70	7213	311.6262747
Q15942	ZYX	rs7801889	7	143499080	C	0.107615	2.28901E-10	7213	40.20296134
Q16270	IGFBP7	rs1718860	4	57083351	G	0.529674	1.5206E-176	7213	802.5298671
Q16288	NTRK3	rs28714295	15	88027853	A	0.450297	1.0062E-74	7213	334.5000772
Q16539	MAPK14	rs12200998	6	36114905	G	-0.146343	1.91204E-18	7213	76.77895797
Q16549	PCSK7	rs171052	11	117211047	G	-0.978177	1E-200	7213	1373.872631
Q16557	PSG3	rs2355433	19	43174936	A	0.711818	1E-200	7213	1373.872631
Q16610	ECM1	rs3737240	1	150510879	T	-0.844053	1E-200	7213	1373.872631
Q16620	NTRK2	rs1624327	9	84814375	A	-0.133728	1.28983E-12	7213	50.34462339
Q16627	CCL14	rs7222922	17	36008654	T	-1.0676	1E-200	7213	1373.872631
Q16651	PRSS8	rs1060506	16	31122128	T	0.124778	9.38297E-12	7213	46.45326876
Q16661	GUCA2B	rs1047047	1	42153468	G	0.213397	1.2443E-20	7213	86.72945077
Q16663	CCL15	rs854624	17	36000884	G	1.41036	1E-200	7213	1373.872631
Q16666	IFI16	rs856051	1	159031502	T	-0.242172	1.946E-29	7213	126.9078788
Q16674	MIA	rs2233159	19	40777460	C	1.36764	1E-200	7213	1373.872631
Q16698	DECR1	rs1805806	8	90005517	C	-0.193941	1.25874E-29	7213	127.772521
Q16719	KYNU	rs112211045	2	143045501	A	-0.879417	1E-200	7213	1373.872631
Q16769	QPCT	rs75573663	2	37345976	C	-1.2178	2.42299E-52	7213	231.7928809
Q16774	GUK1	rs4653908	1	228140298	T	1.32568	1E-200	7213	1116.637411
Q16775	HAGH	rs13166	16	1827912	G	0.561284	2.1177E-125	7213	567.3495534
Q16831	UPP1	rs10278152	7	48089060	T	-0.144709	6.15751E-18	7213	74.46973335
Q16836	HADH	rs72890511	4	107967387	C	0.260889	1.98482E-17	7213	72.15955827
Q16881	TXNRD1	rs7298739	12	104296440	T	0.139226	8.96696E-17	7213	69.18450879
Q24JP5	TMEM132A	rs555835	11	60921973	T	-0.698568	1E-200	7213	1373.872631
Q2I0M5	RSPO4	rs75904281	20	963194	T	-0.685955	6.71031E-23	7213	97.06456559
Q2M296	MTHFSD	rs9937599	16	86527683	A	-0.436248	5.9846E-152	7213	689.4171661
Q2MKA7	RSPO1	rs36043533	1	37613845	G	0.760438	1.7432E-92	7213	416.0769808
Q2TAL6	VWC2	rs1876569	7	49782154	T	-0.639182	6.4806E-173	7213	785.8359482
Q3ZCW2	LGALSL	rs76392037	2	64341766	C	0.57949	1.81929E-55	7213	246.1220124
Q495A1	TIGIT	rs6792290	3	114294347	G	0.125569	4.1591E-12	7213	48.04800776
Q49AH0	CDNF	rs61738953	10	14820083	G	-1.09924	9.7437E-137	7213	619.4714108
Q4G0W2	DUSP28	rs148245427	2	240446758	G	-1.56543	3.0808E-128	7213	580.3927123

Q4G148	GXYLT1	rs139695917	12	42133239	C	1.68275	5.7621E-128	7213	579.1426202
Q4KMG0	CDON	rs112978541	11	126018717	C	-1.03696	1E-200	7213	1365.546782
Q4LDE5	SVEP1	rs61751937	9	110549951	C	1.10566	1.9593E-118	7213	535.3267389
Q504Y2	PKDCC	rs2424	2	42058435	A	0.38507	2.10831E-83	7213	374.3552568
Q53FA7	TP53I3	rs10191964	2	24064728	A	-0.991794	1E-200	7213	1373.872631
Q53S33	BOLA3	rs1620482	2	74160099	C	-0.119896	1.26872E-12	7213	50.37700748
Q5GFL6	VWA2	rs35060624	10	114139781	T	0.463603	1.33869E-53	7213	237.5603005
Q5JXM2	METTL24	rs7767851	6	110356906	T	-0.291075	2.53232E-24	7213	103.5552567
Q5JZY3	EPHA10	rs12074120	1	37731729	G	0.155682	8.42702E-18	7213	73.85033273
Q5KU26	COLEC12	rs2846666	18	469797	C	-0.222127	1.16576E-32	7213	141.6399492
Q5QGZ9	CLEC12A	rs1060648	12	9983600	C	1.06488	1E-200	7213	1373.872631
Q5SRI9	MANEA	rs35772543	6	95606046	A	-1.44736	1E-200	7213	1373.872631
Q5SW96	LDLRAP1	rs4523506	1	25566730	T	-0.358204	9.0431E-104	7213	467.9295477
Q5SZK8	FREM2	rs9603416	13	38755978	A	0.155925	2.62502E-18	7213	76.15310588
Q5T2D2	TREML2	rs4714431	6	41200599	C	0.505081	8.5362E-200	7213	909.4787696
Q5VWK5	IL23R	rs12569203	1	67132664	A	0.101877	7.45363E-10	7213	37.89813918
Q5VXI9	LIPN	rs10509554	10	88766035	T	0.806182	1E-200	7213	1373.872631
Q5VY43	PEAR1	rs12041331	1	156899922	A	-0.541775	3.97903E-76	7213	340.94174
Q63HQ2	EGFLAM	rs2434498	5	38351387	T	-0.198109	4.97792E-24	7213	102.2162771
Q66K79	CPZ	rs6845969	4	8400738	T	-0.144787	3.28312E-16	7213	66.62549689
Q6B8I1	DUSP13	rs6480771	10	75101922	C	-0.307987	2.44567E-75	7213	337.3206234
Q6BAA4	FCRLB	rs61801180	1	161718974	C	0.697481	3.87876E-81	7213	363.9536867
Q6BCY4	CYB5R2	rs7942293	11	7656420	G	-0.535655	1E-200	7213	1019.317338
Q6DN72	FCRL6	rs6656979	1	159812391	A	-0.470415	7.3551E-114	7213	514.3003612
Q6E0U4	DMKN	rs7408798	19	35512067	A	-0.212346	6.64161E-15	7213	60.70160115
Q6EMK4	VASN	rs757593	16	4391616	A	-0.133906	9.78806E-13	7213	50.88617462
Q6FHJ7	SFRP4	rs75207237	7	37932788	A	0.402321	1.07716E-57	7213	256.340229
Q6GPI1	CTRB2	rs8048956	16	75221068	G	0.961322	1E-200	7213	1373.872631
Q6IQ23	PLEKHA7	rs1638457	11	16858401	T	0.389561	9.69332E-48	7213	210.6939234
Q6MZW2	FSTL4	rs111914149	5	133617238	A	0.144165	1.6064E-14	7213	58.96330441
Q6NUJ1	PSAPL1	rs35791045	4	7425380	G	0.578542	6.7009E-160	7213	725.9808853
Q6NVY1	HIBCH	rs291466	2	190319749	A	-0.827138	1E-200	7213	1373.872631
Q6NW40	RGMB	rs2368553	5	98446049	C	-0.339456	3.17047E-95	7213	428.6665073
Q6P179	ERAP2	rs2910686	5	96916885	C	0.98165	1E-200	7213	1373.872631
Q6P4F1	FUT10	rs2732317	8	33473169	A	0.784589	1E-200	7213	1373.872631
Q6P587	FAHD1	rs3743853	16	1827557	A	-0.326481	9.9033E-48	7213	210.6512619
Q6P988	NOTUM	rs111343749	17	81966908	A	-0.267293	3.78006E-11	7213	43.72451993
Q6P995	FAM171B	rs12612335	2	186701929	A	-0.264785	1.65453E-35	7213	154.6683286
Q6PCB0	VWA1	rs116279124	1	1525927	T	-1.60513	7.6355E-114	7213	514.2256714
Q6PI48	DARS2	rs12564699	1	173708900	A	-0.301577	1.51354E-60	7213	269.4260763
Q6PI73	LILRA6	rs34810796	19	54244555	C	1.07816	1E-200	7213	1373.872631
Q6Q788	APOA5	rs3135506	11	116791691	C	1.32942	1E-200	7213	1373.872631
Q6UW15	REG3G	rs430298	2	79022646	T	0.584061	1.8969E-197	7213	898.683373
Q6UWB1	IL27RA	rs35026308	19	14042481	C	-1.01263	1E-200	7213	1373.872631
Q6UWL6	KIRREL2	rs35854130	19	35861033	T	-0.17087	6.33984E-17	7213	69.86832423
Q6UWN8	SPINK6	rs11741093	5	148221967	C	-1.01238	1E-200	7213	1091.171851
Q6UWV6	ENPP7	rs8077074	17	79723622	T	0.946027	1E-200	7213	1373.872631
Q6UWY0	ARSK	rs956274	5	95611429	T	-0.133637	6.6708E-15	7213	60.69296826
Q6UWY2	PRSS57	rs2301741	19	675106	C	-0.332643	1.39682E-91	7213	411.9248407
Q6UX15	LAYN	rs4938792	11	111550211	C	-0.249856	3.91568E-50	7213	221.66685
Q6UX41	BTNL8	rs2387717	5	180938103	A	-0.374847	2.5331E-117	7213	530.2173762
Q6UXB4	CLEC4G	rs115293707	19	7729723	C	0.542015	4.54713E-67	7213	299.3575624
Q6UXI7	VIT	rs1468810	2	36766797	C	0.520041	1E-200	7213	1001.365419
Q6UXI9	NPNT	rs34712979	4	105897896	A	-0.387159	5.18555E-92	7213	413.9018904
Q6UXK2	ISLR2	rs4887134	15	74317938	A	0.289794	5.60471E-68	7213	303.5307567
Q6UXM1	LRIG3	rs11172791	12	58879191	C	-0.846134	2.3751E-101	7213	456.8119428
Q6UXV0	GFRAL	rs73439277	6	55393672	A	0.29143	3.42952E-34	7213	148.6445692
Q6UXZ4	UNC5D	rs3108622	8	35257097	A	-0.290632	1.80978E-68	7213	305.7842118
Q6UY11	DLK2	rs2125739	6	43445127	C	-0.413378	6.6486E-112	7213	505.3095587
Q6WN34	CHRDL2	rs61389091	11	74716876	T	-0.751669	2.60634E-74	7213	332.6022013
Q6Y288	B3GLCT	rs4943306	13	31320378	C	-0.346313	3.88024E-69	7213	308.8540733
Q6YHK3	CD109	rs6925924	6	73794004	C	0.729675	1E-200	7213	1373.872631
Q6YP21	KYAT3	rs12130865	1	88930764	A	-0.186871	2.0629E-29	7213	126.7921041
Q6ZMC9	SIGLEC15	rs515373	18	45826966	A	0.136359	5.89139E-16	7213	65.47305765
Q6ZMJ2	SCARA5	rs2685422	8	27947867	A	-0.253335	7.7278E-44	7213	192.8142881
Q6ZRP7	QSOX2	rs10858248	9	136216478	G	-0.456393	1.5948E-171	7213	779.4379543
Q6ZVL6	KIAA1549L	rs2753408	11	33378145	A	-0.545943	1E-200	7213	1155.708406
Q76LX8	ADAMTS13	rs28647808	9	133440409	G	-0.891989	1E-200	7213	1043.503638
Q76M96	CCDC80	rs9870933	3	112653470	A	0.147132	7.42893E-18	7213	74.09917691
Q7L0X0	TRIL	rs740250	7	28957328	T	0.299451	1.64266E-33	7213	145.5324861
Q7L1S5	CHST9	rs11660451	18	27118770	C	0.304102	4.12418E-66	7213	294.9623435
Q7L266	ASRGL1	rs3017096	11	62410486	T	0.140228	5.11124E-17	7213	70.29324573
Q7LFX5	CHST15	rs7078438	10	124109086	C	-0.193143	8.31386E-27	7213	114.891135
Q7LG56	RRM2B	rs11785514	8	102191915	C	0.411628	1.62634E-36	7213	159.278858
Q7Z3B1	NEGR1	rs11209871	1	71934973	G	-0.238782	1.62219E-23	7213	99.87629833
Q7Z4F1	LRP10	rs182793305	14	22894897	A	0.531241	3.08882E-19	7213	80.38015366
Q7Z4H8	POGLUT3	rs141379009	11	108278480	G	-1.38995	2.5264E-188	7213	856.7113848

Q7Z4W1	DCXR	rs7207947	17	82187415	T	0.140266	4.08694E-17	7213	70.73444586
Q7Z553	MDGA2	rs145891385	14	47374910	G	0.758481	3.9308E-103	7213	464.9969768
Q7Z5A7	TAFA5	rs5768649	22	48471219	T	-0.169841	1.3244E-10	7213	41.27218698
Q7Z7M8	B3GNT8	rs284663	19	41426707	C	-0.827732	1E-200	7213	1373.872631
Q86SJ2	AMIGO2	rs1101751	12	47092599	G	0.149186	1.84772E-16	7213	67.75878196
Q86TD4	SRL	rs11640587	16	4214222	C	0.187831	3.22775E-28	7213	121.3349007
Q86TH1	ADAMTSL2	rs10120207	9	133547919	C	0.178086	1.89025E-09	7213	36.08366255
Q86U17	SERPINA11	rs55911632	14	94452451	G	-0.346229	1.77844E-52	7213	232.4087811
Q86UD1	OAF	rs2845705	11	120230383	A	0.706956	1E-200	7213	1270.021173
Q86UW8	HAPLN4	rs55762233	19	19256510	G	0.281069	1.99214E-34	7213	149.7238502
Q86UX2	ITIH5	rs6602258	10	7656775	G	-0.427218	1.1401E-115	7213	522.6181845
Q86UX7	FERMT3	rs7948329	11	64139829	G	-0.142634	2.43564E-17	7213	71.75566245
Q86V40	TRABD2A	rs7598011	2	84863432	G	0.141713	1.22061E-08	7213	32.45378482
Q86VB7	CD163	rs11054859	12	7617180	A	-0.174473	1.08729E-10	7213	41.65783118
Q86VR8	FJX1	rs10768174	11	36057706	G	0.273935	1.35752E-61	7213	274.23128
Q86VW2	ARHGEF25	rs10437954	12	57610139	G	0.310478	2.75258E-29	7213	126.2197081
Q86VZ4	LRP11	rs3805753	6	149795662	T	0.912601	1E-200	7213	1373.872631
Q86WA6	BPHL	rs3799225	6	3152008	G	0.217967	1.91837E-08	7213	31.57547624
Q86WD7	SERPINA9	rs11160181	14	94461852	T	-0.193143	7.4216E-31	7213	133.3918901
Q86XP6	GKN2	rs62133344	2	68950137	A	-0.964384	5.9006E-159	7213	721.6360994
Q86YB8	ERO1B	rs2463201	1	236242197	T	0.328498	9.6596E-87	7213	389.6918629
Q86Z14	KLB	rs13112140	4	39450504	A	-0.635276	1E-200	7213	1373.872631
Q8IU57	IFNLR1	rs139958347	1	24187233	C	-0.907799	2.44001E-97	7213	438.3783023
Q8IUC8	GALNT13	rs67335822	2	154010909	T	0.145814	1.83969E-10	7213	40.62990991
Q8IUL8	CILP2	rs4808205	19	19534684	G	0.184904	1.80958E-16	7213	67.79990726
Q8IV08	PLD3	rs4803321	19	40237362	A	-0.55044	9.82579E-36	7213	155.7039217
Q8IVD9	NUDCD3	rs73105364	7	44440865	T	0.376946	3.1464E-17	7213	71.25044431
Q8IVM0	CCDC50	rs147604673	3	191389569	T	-0.55557	6.6278E-18	7213	74.32443616
Q8IW75	SERPINA12	rs4900236	14	94538816	T	0.530021	3.1306E-166	7213	755.0947292
Q8IWQ3	BRSK2	rs4255564	11	1384495	C	0.121941	1.02347E-10	7213	41.7760973
Q8IWV2	CNTN4	rs13071423	3	2098694	A	0.370997	2.04677E-68	7213	305.5388939
Q8IWY4	SCUBE1	rs5759290	22	43337746	A	0.254109	4.91622E-28	7213	120.5001849
Q8IX30	SCUBE3	rs942374	6	35176115	T	-0.356218	1.18327E-55	7213	246.9788936
Q8IXB1	DNAJC10	rs288334	2	182757820	T	-0.211479	1.00255E-34	7213	151.0882211
Q8IYB5	SMAP1	rs1917342	6	70640051	G	-0.751995	1E-200	7213	1373.872631
Q8IYJ0	PIANP	rs11064321	12	6700730	C	0.345008	1.02114E-93	7213	421.7383058
Q8IYR6	TMEFF1	rs10989102	9	100475300	C	-0.241732	2.11068E-21	7213	90.23892942
Q8IYS0	GRAMD1C	rs61634901	3	113907112	A	0.575464	1E-200	7213	1140.429472
Q8IZF2	ADGRF5	rs586024	6	46866948	G	-1.37574	1E-200	7213	1373.872631
Q8IZI9	IFNL3	rs62120535	19	39270392	G	0.119778	1.58758E-08	7213	31.94306681
Q8IZJ1	UNC5B	rs7907590	10	71292875	G	0.262621	6.4963E-29	7213	124.5157035
Q8IZP7	HS6ST3	rs12860877	13	96036886	C	0.128073	1.3747E-14	7213	59.26980218
Q8IZS8	CACNA2D3	rs9882539	3	54129177	G	0.273787	2.56579E-61	7213	272.9626697
Q8N128	FAM177A1	rs799473	14	35047007	G	0.896028	1E-200	7213	1373.872631
Q8N142	ADSS1	rs10142660	14	104759283	T	0.185815	1.73343E-13	7213	54.28587929
Q8N149	LILRA2	rs2151868	19	54593448	C	-0.583312	5.7933E-108	7213	487.2006261
Q8N1Q1	CA13	rs113014331	8	85272310	T	-1.22592	1E-200	7213	1373.872631
Q8N2Q7	NLGN1	rs497383	3	173407133	G	0.230434	2.25826E-35	7213	154.050128
Q8N2S1	LTBP4	rs34093919	19	40611394	A	-0.748276	3.31312E-26	7213	112.149774
Q8N3J6	CADM2	rs9880919	3	85412152	A	0.154652	2.74936E-15	7213	62.43821593
Q8N3T6	TMEM132C	rs9669390	12	128277877	T	-0.32637	1.10558E-79	7213	357.2720649
Q8N423	LILRB2	rs383925	19	54279666	T	-1.10992	1E-200	7213	1373.872631
Q8N428	GALNT16	rs12100668	14	69326758	G	-0.314204	2.64756E-78	7213	350.9381459
Q8N474	SFRP1	rs147918758	8	41310043	G	-0.209407	2.16427E-35	7213	154.1346096
Q8N4Q0	PTGR3	rs12969004	18	75208110	A	-0.165514	9.68214E-23	7213	96.338649
Q8N6G6	ADAMTSL1	rs117766170	9	18406352	A	0.355752	1.42611E-25	7213	109.2561712
Q8N6Q3	CD177	rs79338083	19	43322051	T	1.54842	8.0857E-152	7213	688.8162313
Q8N729	NPW	rs111276210	16	2016341	A	-0.305531	1.21854E-19	7213	82.21833889
Q8N8U9	BMPER	rs16879245	7	33764873	G	-0.381904	1.71716E-37	7213	163.7480091
Q8N9N8	EIF1AD	rs182527881	11	65955537	T	0.622983	8.60767E-32	7213	137.6694278
Q8NAT1	POMGNT2	rs12639294	3	43109244	G	-0.404904	1.994E-134	7213	608.8461098
Q8NBI6	XXYLT1	rs13072883	3	195274025	G	-0.302452	1.25368E-72	7213	324.8789421
Q8NBJ4	GOLM1	rs138447426	9	86060717	G	1.159	1.99868E-74	7213	333.1315405
Q8NBJ5	COLGALT1	rs3746317	19	17556110	T	-0.141092	3.43301E-17	7213	71.07844073
Q8NBL1	POGLUT1	rs17203139	3	119486370	G	1.19901	1E-200	7213	1273.777164
Q8NBP7	PCSK9	rs11591147	1	55039974	T	-1.04454	2.39068E-51	7213	227.234253
Q8NBS9	TXNDC5	rs111331197	6	7883235	T	1.36486	5.22489E-57	7213	253.1942708
Q8NBZ7	UXS1	rs3888361	2	106173309	C	0.358402	2.22868E-46	7213	204.4534214
Q8NCG5	CHST4	rs7200431	16	71492891	A	0.203281	1.95297E-29	7213	126.9007833
Q8NCW5	NAXE	rs41267389	1	156593649	A	-1.46466	1E-200	7213	927.0175825
Q8NFI3	ENGASE	rs56107536	17	79074958	A	0.681166	7.146E-180	7213	817.8368332
Q8NFM7	IL17RD	rs6776722	3	57108631	G	0.624575	1E-200	7213	1314.318357
Q8NFP4	MDGA1	rs9349050	6	37695848	T	0.995718	1E-200	7213	1373.872631
Q8NFT8	DNER	rs35975053	2	229729774	A	-0.246397	7.33117E-45	7213	197.50106
Q8NFU3	TSTD1	rs10908821	1	161038745	G	0.998822	1E-200	7213	1373.872631
Q8NFZ4	NLGN2	rs150452493	17	7400489	T	-0.403137	2.87289E-41	7213	181.0401404
Q8NHL6	LILRB1	rs2098953	19	54634136	C	-1.47916	1E-200	7213	1373.872631

Q8TAT2	FGFBP3	rs11186737	10	91906592	T	-0.600419	1E-200	7213	1206.515164
Q8TB22	SPATA20	rs8076632	17	50548567	G	-0.676147	1E-200	7213	1373.872631
Q8TB73	NDNF	rs6840113	4	121015352	A	-0.102137	1.34044E-09	7213	36.75358347
Q8TCD5	NT5C	rs78625720	17	75144846	A	-1.52849	7.3542E-200	7213	909.7765146
Q8TCF1	ZFAND1	rs2912805	8	81689005	G	0.72832	1E-200	7213	1039.8471
Q8TCV5	WFDC5	rs6124684	20	45092852	T	-0.133065	5.56596E-13	7213	51.99438398
Q8TD46	CD200R1	rs62263730	3	112859161	A	0.245101	2.06611E-42	7213	186.276402
Q8TDL5	BPIFB1	rs11699448	20	33106766	T	0.320032	1.6357E-81	7213	365.6758809
Q8TDQ0	HAVCR2	rs1432872	5	157116385	C	0.975569	1E-200	7213	1373.872631
Q8TDX7	NEK7	rs142662761	1	198041651	G	-0.960715	1.97312E-32	7213	140.5947686
Q8TDY8	IGDCC4	rs76702386	15	65620544	C	-0.452024	3.08556E-58	7213	258.8309926
Q8TEA8	DTD1	rs6081231	20	18590320	A	-0.761554	1E-200	7213	1373.872631
Q8TEU8	WFIKKN2	rs11079936	17	50830473	T	0.706933	1E-200	7213	1373.872631
Q8TEY5	CREB3L4	rs4845586	1	153970121	G	0.288777	2.1575E-68	7213	305.4338611
Q8TF42	UBASH3B	rs3937027	11	122655065	A	-0.367631	1.67254E-82	7213	370.2242108
Q8TF66	LRRC15	rs923930	3	194364494	A	0.841539	1E-200	7213	1081.4884
Q8WTT0	CLEC4C	rs10845821	12	7747588	T	0.593882	1E-200	7213	1323.702555
Q8WU39	MZB1	rs116498185	5	139190262	T	0.20891	1.21882E-08	7213	32.45663678
Q8WUJ1	CYB5D2	rs183282334	17	4148540	A	-1.30617	1.22292E-83	7213	375.4416647
Q8WUM4	PDCD6IP	rs1141054	3	33798622	C	-0.669194	1E-200	7213	1373.872631
Q8WVF2	UCMA	rs1537771	10	13241177	T	-0.440307	1.18332E-79	7213	357.1365343
Q8WVN6	SECTM1	rs1132115	17	82321536	T	-0.350322	1.2531E-100	7213	453.4928771
Q8WVY7	UBLCP1	rs71593333	5	159263022	A	-0.72001	2.83336E-20	7213	85.10219387
Q8WW12	PCNP	rs13060373	3	101631980	A	-0.346945	2.3694E-101	7213	456.8166959
Q8WW22	DNAJA4	rs11639195	15	78255151	T	0.115394	5.68178E-09	7213	33.94069384
Q8WW52	FAM151A	rs2060385	1	54623799	T	0.302959	1.82683E-69	7213	310.3558829
Q8WWA0	ITLN1	rs61803286	1	160861601	T	0.181304	5.09426E-09	7213	34.15307798
Q8WWV3	RTN4IP1	rs17565019	6	106610517	C	0.0942912	4.9443E-08	7213	29.73850245
Q8WWX9	SELENOM	rs11705054	22	31105995	A	0.285625	1.40443E-13	7213	54.69948625
Q8WXD2	SCG3	rs2607116	15	51682236	A	-0.809605	1E-200	7213	1373.872631
Q8WXI7	MUC16	rs193266507	19	8854282	C	-0.288148	2.9477E-18	7213	75.92416886
Q8WZ59	TMEM190	rs4806666	19	55376727	T	-0.828066	1E-200	7213	1373.872631
Q8WZ82	OVCA2	rs145234879	17	2042060	A	1.40274	1.77486E-91	7213	411.4469493
Q92484	SMPDL3A	rs28385609	6	122801319	T	-0.482748	1.63587E-96	7213	434.5814463
Q92496	CFHR4	rs10494745	1	196918327	A	-0.936087	1E-200	7213	1306.329666
Q92563	SPOCK2	rs3312	10	72097226	G	0.251867	1.5552E-52	7213	232.6759069
Q92583	CCL17	rs4396523	16	57408279	C	0.510802	8.35521E-67	7213	298.144817
Q92598	HSPH1	rs9538558	13	31143416	A	0.199646	1.6577E-23	7213	99.8334116
Q92619	ARHGAP45	rs2240051	19	1076684	G	0.351603	2.43352E-94	7213	424.599907
Q92626	PXDN	rs10202812	2	1742696	T	0.249433	5.19642E-38	7213	166.1243448
Q92692	NECTIN2	rs440277	19	44857967	A	-0.141434	1.89789E-15	7213	63.16818719
Q92729	PTPRU	rs2179795	1	29315806	T	-0.253223	3.43845E-43	7213	189.8440909
Q92743	HTRA1	rs7074542	10	122353283	T	-0.190676	2.73656E-26	7213	112.5288347
Q92752	TNR	rs2235256	1	175599911	G	-0.218953	3.33005E-39	7213	171.5874959
Q92765	FRZB	rs288326	2	182838608	A	0.637811	5.291E-138	7213	625.2885046
Q92769	HDAC2	rs13212298	6	113921735	C	-0.10362	4.553E-08	7213	29.89835054
Q92820	GGH	rs4739037	8	62990765	A	0.910339	6.9157E-173	7213	785.7061652
Q92832	NELL1	rs8176786	11	20937848	T	1.09483	4.2692E-184	7213	837.2643618
Q92854	SEMA4D	rs45464494	9	89379518	T	-1.33561	1E-200	7213	1373.872631
Q92859	NEO1	rs13379875	15	73050440	C	-0.352341	4.35297E-98	7213	441.8179794
Q92947	GCDH	rs2238641	19	12895871	C	-0.170847	1.08157E-23	7213	100.6791709
Q93070	ART4	rs7311924	12	14837871	G	0.968653	1E-200	7213	1373.872631
Q93091	RNASE6	rs1045922	14	20781965	A	1.05847	1E-200	7213	1373.872631
Q969E1	LEAP2	rs60545802	5	132868885	G	0.41656	4.50278E-81	7213	363.6561412
Q969G6	RFK	rs594586	9	76699375	G	-0.165003	2.09346E-17	7213	72.05439881
Q969J5	IL22RA2	rs6917836	6	137132089	A	-0.223758	1.03369E-16	7213	68.90411335
Q969M7	UBE2F	rs2326005	2	238008106	A	0.118121	1.65429E-12	7213	49.85629241
Q969P0	IGSF8	rs12401728	1	160092169	T	0.293985	3.85132E-48	7213	212.5313626
Q969T7	NT5C3B	rs57672173	17	41837233	C	0.211134	8.19296E-28	7213	119.4870226
Q969X2	ST6GALNAC6	rs61740973	9	127894674	T	-0.380105	4.34461E-08	7213	29.98919271
Q969Z4	RELT	rs56801796	11	73379220	G	0.154046	9.55301E-15	7213	59.98609084
Q96AP7	ESAM	rs12541	11	124753596	C	-0.392821	1.77723E-90	7213	406.8502908
Q96AT9	RPE	rs2723211	2	210015768	A	0.147632	7.50596E-19	7213	78.62588045
Q96AW1	VOPP1	rs117042408	7	55555698	A	-0.489085	1.15961E-24	7213	105.1027991
Q96B86	RGMA	rs10852188	15	93064200	A	0.241869	3.17964E-47	7213	208.3292726
Q96BJ3	AIDA	rs28709375	1	222649657	A	0.260654	9.26418E-45	7213	197.035357
Q96BQ1	FAM3D	rs7433100	3	58665459	T	-0.438052	1.03638E-57	7213	256.4171195
Q96BS2	TESC	rs2393124	12	117055665	G	-0.357116	4.12392E-94	7213	423.5474464
Q96CN7	ISOC1	rs77655181	5	128959744	T	0.249239	1.78906E-43	7213	191.1439977
Q96D42	HAVCR1	rs6889164	5	157039767	C	-0.466529	2.6618E-145	7213	658.8465592
Q96DB5	RMDN1	rs7818213	8	86491192	C	0.948348	1E-200	7213	1373.872631
Q96DU3	SLAMF6	rs1041067	1	160487337	T	-0.388563	1.45289E-79	7213	356.7272135
Q96DX8	RTP4	rs1878861	3	187375904	G	0.506623	1E-200	7213	967.0207255
Q96DZ1	ERLEC1	rs2692523	2	53823074	T	-0.247753	7.01305E-30	7213	128.933459
Q96EE4	CCDC126	rs35121828	7	23595366	A	0.497549	2.4013E-157	7213	714.2341563
Q96EK5	KIFBP	rs17471869	10	68990113	G	0.0938728	1.29163E-08	7213	32.34388555
Q96EK6	GNPNAT1	rs2296494	14	52783575	C	-0.348992	6.67357E-94	7213	422.5870045

Q96EY8	MMAB	rs7970557	12	109564328	T	-0.67284	1E-200	7213	1046.78911
Q96F10	SAT2	rs13894	17	7626584	A	-0.317648	1.94016E-22	7213	94.96260403
Q96F46	IL17RA	rs4819959	22	17105741	G	-0.799341	1E-200	7213	1373.872631
Q96F85	CNRIP1	rs7604489	2	68352779	T	-0.815743	1E-200	7213	1364.563933
Q96FE5	LINGO1	rs62007781	15	77734259	A	0.134419	1.13695E-15	7213	64.17758385
Q96FJ2	DYNLL2	rs9902118	17	58093234	C	-0.3846	2.0548E-107	7213	484.673718
Q96FN9	DTD2	rs17097898	14	31449817	T	-0.460686	1.105E-77	7213	348.0886687
Q96FQ6	S100A16	rs9729773	1	153622147	A	-0.319067	2.20961E-85	7213	383.4478651
Q96GP6	SCARF2	rs5763025	22	20432201	A	-0.362552	4.91179E-68	7213	303.7938337
Q96GW7	BCAN	rs2365715	1	156645322	G	-0.31023	1.22372E-76	7213	343.2931917
Q96H15	TIMD4	rs1501908	5	156971158	G	0.2057	7.03433E-33	7213	142.6432981
Q96HD1	CRELD1	rs6762702	3	10014105	A	-0.964466	1E-200	7213	1373.872631
Q96I25	RBM17	rs11256726	10	6100347	C	-0.745598	4.65464E-66	7213	294.7211613
Q96IY4	CPB2	rs7988836	13	46069238	G	0.766245	1E-200	7213	1373.872631
Q96J42	TXNDC15	rs3733897	5	134887903	G	0.472112	4.34194E-86	7213	386.693639
Q96JA1	LRIG1	rs2306272	3	66384219	C	-0.753717	1E-200	7213	1373.872631
Q96KN2	CNDP1	rs17817077	18	74542308	A	0.244799	1.5304E-47	7213	209.7848527
Q96LA6	FCRL1	rs4971154	1	157802090	C	0.407539	1.1601E-138	7213	628.3186381
Q96LB9	PGLYRP3	rs55991125	1	153307220	T	-0.38828	4.26638E-26	7213	111.6484269
Q96LL9	DNAJC30	rs9647712	7	73682207	A	1.04623	1.448E-121	7213	549.7206112
Q96MK3	FAM20A	rs12453086	17	68535757	T	-0.283788	1.2514E-46	7213	205.602165
Q96MU8	KREMEN1	rs2205771	22	29098605	A	0.427977	3.36664E-76	7213	341.2750143
Q96NZ8	WFIKKN1	rs55798945	16	619708	G	0.170856	4.33351E-20	7213	84.26206426
Q96P31	FCRL3	rs3761959	1	157699488	T	0.711587	1E-200	7213	1373.872631
Q96PD5	PGLYRP2	rs10164310	19	15475134	A	-0.423254	1.1196E-74	7213	334.287129
Q96PJ5	FCRL4	rs11582663	1	157589332	T	-1.00443	1E-200	7213	1373.872631
Q96PQ1	SIGLEC12	rs3826667	19	51500820	C	1.09955	1E-200	7213	1373.872631
Q96PX8	SLITRK1	rs2876803	13	83794650	A	0.158333	1.19132E-12	7213	50.50054016
Q96Q83	ALKBH3	rs2292889	11	43920227	G	-0.216182	6.65503E-31	7213	133.6083356
Q96QR1	SCGB3A1	rs307802	5	180592237	C	0.117237	6.1608E-12	7213	47.27773678
Q96QV1	HHIP	rs11727676	4	144737912	C	0.299585	3.301E-25	7213	107.592693
Q96R05	RBP7	rs35232720	1	9991107	T	-1.01757	7.4205E-180	7213	817.7615408
Q96SL4	GPX7	rs6669154	1	52609880	A	0.719894	1E-200	7213	1121.305338
Q96SM3	CPXM1	rs11697820	20	2800795	C	-0.709504	1E-200	7213	1373.872631
Q96T60	PNKP	rs2290773	19	49888382	G	-0.590237	1.00611E-11	7213	46.31654091
Q99075	HBEGF	rs1991801	5	140623810	G	0.109534	1.23752E-09	7213	36.90933525
Q99426	TBCB	rs2231569	19	36114973	T	0.822433	5.1194E-89	7213	400.1456933
Q99497	PARK7	rs17523802	1	7961680	A	0.79023	1E-200	7213	1373.872631
Q99536	VAT1	rs4239148	17	43077840	A	-0.0959998	4.8105E-08	7213	29.79168807
Q99538	LGMN	rs148659834	14	92709697	A	-0.853805	1.08352E-33	7213	146.3591011
Q99542	MMP19	rs56180965	12	55847804	C	-0.317774	2.36559E-16	7213	67.27164771
Q99650	OSMR	rs357277	5	38931817	G	-0.204229	4.68258E-33	7213	143.4516301
Q99706	KIR2DL4	rs685323	19	54795911	C	-0.689406	1E-200	7213	1373.872631
Q99727	TIMP4	rs184262	3	12093240	A	-0.560213	1.2131E-145	7213	660.4158165
Q99729	HNRNPAB	rs72648830	5	178181541	T	0.170735	1.32791E-11	7213	45.77285444
Q99733	NAP1L4	rs4758622	11	2988691	A	-0.839201	1E-200	7213	1373.872631
Q99784	OLFM1	rs10858334	9	135097939	G	0.198987	5.80182E-17	7213	70.04325424
Q99816	TSG101	rs1395319	11	18526636	G	0.527376	1E-200	7213	958.6752229
Q99829	CPNE1	rs113522583	20	35624405	A	-1.32815	1E-200	7213	1373.872631
Q99935	OPRPN	rs77611689	4	70181247	A	0.211482	2.34117E-10	7213	40.15894524
Q99959	PKP2	rs11052286	12	32886492	T	0.114803	3.15628E-10	7213	39.57543903
Q99969	RARRES2	rs3735167	7	150342466	T	0.50049	1.0243E-158	7213	720.5345868
Q99972	MYOC	rs235879	1	171646796	T	-0.434479	1.9977E-153	7213	696.2069133
Q99983	OMD	rs10992426	9	92703641	A	-0.155488	3.47563E-15	7213	61.97659997
Q99985	SEMA3C	rs917191	7	80941555	G	-0.17953	7.53137E-27	7213	115.0871528
Q99988	GDF15	rs1058587	19	18388612	G	0.672832	1E-200	7213	1373.872631
Q9BPX1	HSD17B14	rs12978094	19	48809982	C	-0.273356	1.34686E-10	7213	41.23931624
Q9BQ16	SPOCK3	rs35065151	4	167127749	A	-0.670458	1E-200	7213	1373.872631
Q9BQ51	PDCD1LG2	rs16923189	9	5510644	G	0.676326	1E-200	7213	1373.872631
Q9BQG2	NUDT12	rs11242495	5	103535378	G	-0.32002	1.28378E-44	7213	196.3861474
Q9BQI0	AIF1L	rs11244284	9	131098400	C	-0.544168	1E-200	7213	923.0077492
Q9BQR3	PRSS27	rs71386687	16	2717893	T	0.192378	7.80119E-10	7213	37.80922747
Q9BR61	ACBD6	rs75468761	1	180281203	G	-0.331618	2.45205E-16	7213	67.20087717
Q9BRK3	MXRA8	rs2765021	1	1362042	C	-0.252527	6.31177E-14	7213	56.27168997
Q9BRX8	PRXL2A	rs10788623	10	80439659	A	-0.093119	2.96361E-08	7213	30.73120184
Q9BSQ5	CCM2	rs7810512	7	45110732	C	0.107052	1.2656E-08	7213	32.38344645
Q9BT09	CNPY3	rs9471969	6	42938646	T	-0.185098	3.47788E-22	7213	93.80729304
Q9BUD6	SPON2	rs2279279	4	1170489	C	0.584408	1E-200	7213	1211.651001
Q9BUP0	EFHD1	rs200118999	2	232633721	G	0.386942	4.70022E-10	7213	38.79794674
Q9BUP3	HTATIP2	rs10437608	11	20364060	G	-0.297298	4.78022E-73	7213	326.8014412
Q9BUT1	BDH2	rs78336913	4	103190257	T	0.356888	5.97227E-62	7213	275.8676083
Q9BW91	NUDT9	rs28805573	4	87408035	A	-0.459196	8.06347E-83	7213	371.6794776
Q9BWD1	ACAT2	rs25683	6	159775311	A	0.344014	6.1277E-94	7213	422.7572744
Q9BWP8	COLEC11	rs6542680	2	3592552	C	0.657269	1E-200	7213	1038.749061
Q9BWV1	BOC	rs3856718	3	113277371	A	0.355246	9.0476E-60	7213	265.8631943
Q9BX59	TAPBPL	rs2244083	12	6461373	T	1.07554	1E-200	7213	1373.872631
Q9BX67	JAM3	rs655627	11	134151964	G	-0.215657	5.24279E-38	7213	166.1066821

Q9BX93	PLA2G12B	rs12257692	10	72944091	C	-0.323922	2.67851E-16	7213	67.02672737
Q9BXB1	LGR4	rs2448001	11	27372266	A	0.170415	5.85139E-23	7213	97.33576199
Q9BXD5	NPL	rs116699595	1	182951103	G	0.3465	4.18025E-15	7213	61.61311494
Q9BXJ0	C1QTNF5	rs9640	11	119339269	A	0.273382	1.74763E-27	7213	117.9843329
Q9BXJ4	C1QTNF3	rs840390	5	34018518	A	-0.213205	9.69063E-18	7213	73.57454642
Q9BXN1	ASPN	rs2516568	9	92425098	T	-0.510872	6.1317E-196	7213	891.7394126
Q9BXR6	CFHR5	rs35662416	1	196998224	A	-1.20255	2.5258E-114	7213	516.4338777
Q9BXS1	IDI2	rs1044261	10	1019770	T	-0.556295	6.63123E-72	7213	321.5577409
Q9BXY4	RSPO3	rs1892172	6	127155371	G	-0.492365	1E-200	7213	956.6975481
Q9BY32	ITPA	rs67002563	20	3213527	A	-1.36641	1E-200	7213	1373.872631
Q9BY67	CADM1	rs17564430	11	115172854	G	0.292195	5.3059E-54	7213	239.4035493
Q9BY76	ANGPTL4	rs116843064	19	8364439	A	-0.59482	3.53109E-25	7213	107.4591506
Q9BYB0	SHANK3	rs6009946	22	50656858	T	0.401098	2.6771E-104	7213	470.3589152
Q9BYC5	FUT8	rs8020305	14	65370785	C	0.728754	2.0213E-100	7213	452.5386757
Q9BYH1	SEZ6L	rs590671	22	26355557	C	0.188905	3.36758E-29	7213	125.8195237
Q9BYZ8	REG4	rs2298069	1	119811396	T	-0.259668	1.24421E-27	7213	118.6582493
Q9BZM4	ULBP3	rs72501730	6	150053632	A	-0.255101	1.35584E-23	7213	100.2315274
Q9BZR6	RTN4R	rs75765	22	20187436	C	0.401726	1.8018E-101	7213	457.3632067
Q9BZW8	CD244	rs114594620	1	160731600	C	-0.356236	2.661E-08	7213	30.94021728
Q9C005	DPY30	rs140841871	2	32226651	T	0.453859	1.3069E-29	7213	127.6980022
Q9GZM7	TINAGL1	rs9425884	1	31571326	C	-0.0943742	1.21625E-08	7213	32.46073885
Q9GZN4	PRSS22	rs7204669	16	2860735	A	0.30323	4.4165E-67	7213	299.4156671
Q9GZP0	PDGFD	rs10895596	11	104161315	A	-0.52836	2.7438E-196	7213	893.3458579
Q9GZQ8	MAP1LC3B	rs9972730	16	87384100	G	-0.124283	9.68377E-14	7213	55.4301901
Q9GZX9	TWSG1	rs62087477	18	9372548	T	0.423284	7.65471E-43	7213	188.2518259
Q9H0B8	CRISPLD2	rs535285054	16	84901047	C	-0.611036	2.23545E-14	7213	58.31314885
Q9H0E2	TOLLIP	rs2056083	11	1348325	A	-0.151326	9.18299E-11	7213	41.98811421
Q9H0P0	NT5C3A	rs12155117	7	33016438	A	0.185298	3.97161E-25	7213	107.2261526
Q9H0R4	HDHD2	rs78720782	18	47137644	T	1.31254	1E-200	7213	1373.872631
Q9H0V9	LMAN2L	rs58361269	2	96737373	A	0.248066	2.41484E-45	7213	199.711046
Q9H299	SH3BGRL3	rs4659423	1	26296483	C	-1.17867	1E-200	7213	1373.872631
Q9H2A7	CXCL16	rs7214635	17	4697725	G	-0.214211	5.38529E-29	7213	124.8878881
Q9H2E6	SEMA6A	rs3733724	5	116444277	T	0.385829	1.94313E-38	7213	168.0801164
Q9H2K0	MTIF3	rs1218825	13	27435783	G	-0.312736	2.11496E-15	7213	62.9548837
Q9H2R5	KLK15	rs73048483	19	50837287	A	0.389093	7.36988E-31	7213	133.405774
Q9H3H3	C11orf68	rs554169857	11	65919002	A	0.924467	5.47151E-52	7213	230.1707395
Q9H3S1	SEMA4A	rs12401997	1	156178005	A	0.268619	5.8533E-57	7213	252.9680141
Q9H3T3	SEMA6B	rs4353572	19	4563925	T	-0.138043	4.56669E-17	7213	70.51547985
Q9H3U1	UNC45A	rs8041035	15	90953012	T	-0.384635	1.78995E-14	7213	58.75042366
Q9H3U7	SMOC2	rs56296467	6	168463127	C	-0.471633	9.2518E-147	7213	665.5551809
Q9H488	POFUT1	rs76143353	20	32227952	T	-1.03236	1E-200	7213	959.2423587
Q9H4A4	RNPEP	rs6702334	1	201985696	A	0.536648	1E-200	7213	947.6791441
Q9H4A9	DPEP2	rs76359116	16	67963800	C	-0.603741	1.88868E-47	7213	209.366134
Q9H4D0	CLSTN2	rs9863263	3	139767054	A	0.592833	6.06693E-65	7213	289.6034141
Q9H4E7	DEF6	rs45482297	6	35297758	A	-0.547559	9.08178E-33	7213	142.1358778
Q9H4F8	SMOC1	rs1958078	14	69888141	A	-0.516105	6.292E-118	7213	532.9976797
Q9H4G4	GLIPR2	rs10814339	9	36154681	C	-0.353374	4.68346E-81	7213	363.5776715
Q9H5V8	CDCP1	rs2276862	3	45146293	G	-0.37749	1.1997E-69	7213	311.1942238
Q9H5Y7	SLITRK6	rs9547187	13	85358375	A	-0.346282	4.1965E-70	7213	313.2883708
Q9H665	IGFLR1	rs12459634	19	35739273	C	-1.00202	1E-200	7213	1373.872631
Q9H6B4	CLMP	rs2302605	11	123194975	C	-0.436321	4.3451E-159	7213	722.2472752
Q9H6E4	CCDC134	rs11090057	22	41735443	A	0.361625	1.4497E-99	7213	448.6069716
Q9H772	GREM2	rs116135446	1	240794633	T	0.229128	4.06797E-08	7213	30.11677563
Q9H7M9	VSIR	rs12415873	10	71770478	T	0.610659	2.9861E-151	7213	686.2071289
Q9H8J5	MANSC1	rs3741798	12	12343152	T	0.634892	1.6757E-140	7213	636.780264
Q9HAB8	PPCS	rs4660635	1	42439366	A	-0.45315	5.5465E-163	7213	740.155274
Q9HAP6	LIN7B	rs4802563	19	49116979	A	0.162023	4.68307E-22	7213	93.21840058
Q9HB21	PLEKHA1	rs10788274	10	122301287	G	0.22619	3.35385E-43	7213	189.8936562
Q9HB29	IL1RL2	rs1960510	2	102205805	T	0.332749	1.31977E-89	7213	402.85015
Q9HB63	NTN4	rs17288108	12	95738117	G	-0.372722	6.39835E-67	7213	298.6767366
Q9HB71	CACYBP	rs16847450	1	174793184	G	-0.703685	5.9757E-170	7213	772.2001177
Q9HBB8	CDHR5	rs12421646	11	578844	G	0.210369	1.52868E-26	7213	113.6833939
Q9HBG7	LY9	rs12128261	1	160792994	G	0.607541	1E-200	7213	969.9137478
Q9HBK9	AS3MT	rs79254677	10	102931810	G	-0.169067	1.85676E-10	7213	40.6118627
Q9HBL8	NMRAL1	rs11557236	16	4469438	A	-0.510755	4.54981E-67	7213	299.3563879
Q9HC16	APOBEC3G	rs3891126	22	39088173	G	-0.332835	7.64756E-34	7213	147.0512721
Q9HC23	PROK2	rs7644362	3	71780091	G	0.785577	1E-200	7213	1343.202875
Q9HC56	PCDH9	rs4884685	13	66678757	C	-0.126729	2.21803E-13	7213	53.80150282
Q9HC57	WFDC1	rs400345	16	84294888	T	-0.845478	1E-200	7213	1373.872631
Q9HCB6	SPON1	rs10832164	11	14026933	C	0.498155	1E-200	7213	957.2048932
Q9HCJ2	LRRC4C	rs10837377	11	40283726	C	0.27077	2.36704E-59	7213	263.946923
Q9HCM2	PLXNA4	rs62622406	7	132198552	T	-0.662224	2.15446E-24	7213	103.8754182
Q9HCN6	GP6	rs1613662	19	55025227	G	-1.06124	1E-200	7213	1373.872631
Q9HCU0	CD248	rs565972	11	66312316	A	0.0955749	1.12174E-08	7213	32.61794948
Q9HCY8	S100A14	rs9330298	1	153617778	A	-0.171167	6.73352E-25	7213	106.179929
Q9HD15	SRA1	rs801460	5	140552345	T	0.461083	4.019E-180	7213	818.9864972
Q9HD89	RETN	rs34124816	19	7668790	C	-0.748559	3.92279E-51	7213	226.248114

Q9HDC9	APMAP	rs227598	20	24926093	T	0.220866	5.49743E-19	7213	79.24110554
Q9NNX6	CD209	rs2335525	19	7720827	G	-0.399727	6.0905E-100	7213	450.3375712
Q9NP79	VTA1	rs225628	6	142151799	C	-0.266426	1.18744E-57	7213	256.1460374
Q9NP81	SARS2	rs1808661	19	38933313	G	0.113394	3.1417E-11	7213	44.08656437
Q9NP99	TREM1	rs2234243	6	41276253	T	-0.890395	1E-200	7213	948.8000055
Q9NPB1	NT5M	rs16961516	17	17304918	A	0.264647	1.11837E-10	7213	41.60272989
Q9NPC4	A4GALT	rs8138197	22	42718545	A	-0.372678	1.017E-113	7213	513.6536084
Q9NPF2	CHST11	rs1704878	12	104577480	A	-0.574916	1E-200	7213	1058.338517
Q9NPH0	ACP6	rs2153463	1	147652507	T	-0.924934	1E-200	7213	1373.872631
Q9NPH3	IL1RAP	rs7611887	3	190629602	C	1.27612	1E-200	7213	1373.872631
Q9NPH6	OBP2B	rs4454354	9	133214142	T	-0.35204	1.97838E-68	7213	305.6066428
Q9NPJ3	ACOT13	rs4544900	6	24677760	T	0.276588	2.365E-47	7213	208.9184548
Q9NQ25	SLAMF7	rs11581248	1	160750284	T	-1.21722	1E-200	7213	1373.872631
Q9NQ30	ESM1	rs4242051	5	54902947	T	-0.181186	3.83034E-21	7213	89.05991592
Q9NQ38	SPINK5	rs17599675	5	148158915	C	0.181518	2.35728E-22	7213	94.57711411
Q9NQ79	CRTAC1	rs684225	10	97882605	A	-0.36741	1.9006E-110	7213	498.6169691
Q9NQW7	XPNPEP1	rs3862006	10	109991006	A	0.386843	8.69155E-63	7213	279.7086006
Q9NQX5	NPDC1	rs71483301	9	137069995	C	-0.110541	1.24356E-09	7213	36.89984241
Q9NR31	SAR1A	rs4746970	10	70146394	C	0.396475	1.6205E-129	7213	586.2727074
Q9NR34	MAN1C1	rs11247595	1	25682168	T	0.292094	8.98231E-72	7213	320.9526792
Q9NR45	NANS	rs7855984	9	98056008	A	0.201298	1.12819E-25	7213	109.7206746
Q9NR46	SH3GLB2	rs11537529	9	129028252	T	-0.576641	1E-200	7213	1287.706655
Q9NR61	DLL4	rs117454282	15	40847504	T	0.375018	9.28639E-11	7213	41.96621943
Q9NR71	ASAH2	rs2842126	10	50258722	A	-0.639068	1E-200	7213	1046.409779
Q9NRB3	CHST12	rs884566	7	2374915	A	0.356557	4.62658E-69	7213	308.5033603
Q9NRJ3	CCL28	rs7710570	5	43378715	T	0.108661	1.13732E-10	7213	41.569881
Q9NRN5	OLFML3	rs3811018	1	113970317	A	-0.173273	1.21344E-21	7213	91.3342188
Q9NRV9	HEBP1	rs1941	12	12975329	G	-1.61272	1E-200	7213	1346.646985
Q9NRW1	RAB6B	rs9813363	3	133885899	A	-0.68858	1E-200	7213	980.8271861
Q9NRX4	PHPT1	rs872463	9	136850355	A	0.558795	1E-200	7213	932.7634533
Q9NS18	GLRX2	rs143131184	1	193166834	T	1.04886	4.14789E-60	7213	267.4172086
Q9NS62	THSD1	rs41292808	13	52397382	T	1.52287	5.9206E-188	7213	855.0100944
Q9NS68	TNFRSF19	rs3814787	13	23578231	C	0.191643	1.35222E-26	7213	113.9266067
Q9NS85	CA10	rs117399000	17	52136371	A	-0.734537	5.10347E-52	7213	230.3094102
Q9NS86	LANCL2	rs7786168	7	55364530	C	-0.118414	8.79925E-12	7213	46.57912825
Q9NS98	SEMA3G	rs2016575	3	52443064	T	0.362468	8.96964E-60	7213	265.8804379
Q9NSY2	STARD5	rs28555408	15	81345531	T	-0.530498	5.71679E-57	7213	253.0150261
Q9NT22	EMILIN3	rs61739314	20	41361737	C	-1.02302	1.2138E-108	7213	490.3202014
Q9NTK5	OLA1	rs10930634	2	174077998	T	0.697832	1E-200	7213	1216.873043
Q9NTU7	CBLN4	rs4811612	20	55694664	T	0.236976	2.18144E-42	7213	186.168341
Q9NUI1	DECR2	rs1204505	16	412881	T	0.276776	1.78436E-64	7213	287.4532313
Q9NUJ1	ABHD10	rs57797651	3	111994785	G	-0.125478	1.51006E-09	7213	36.52131376
Q9NUM4	TMEM106B	rs5011432	7	12229042	C	-0.25715	1.3258E-53	7213	237.579571
Q9NUU7	DDX19A	rs138325325	16	70077427	A	-1.07038	6.04538E-50	7213	220.8021135
Q9NVM6	DNAJC17	rs57825040	15	40800118	G	-0.115275	3.03725E-11	7213	44.15274873
Q9NVQ4	FAIM	rs641320	3	138629115	A	-0.727406	1.1401E-113	7213	513.4254349
Q9NWX6	THG1L	rs2270812	5	157737954	T	-0.842484	1E-200	7213	1373.872631
Q9NX01	TXNL4B	rs116891509	16	72071661	T	0.557124	4.09363E-70	7213	313.3378514
Q9NX62	BPNT2	rs112433249	8	56964017	C	-1.87713	5.2415E-178	7213	809.2569261
Q9NXS2	QPCTL	rs17850756	19	45703004	A	-0.47655	1.0949E-164	7213	747.9949953
Q9NXW2	DNAJB12	rs9415063	10	72326894	C	0.199928	4.31862E-31	7213	134.4668965
Q9NY97	B3GNT2	rs34361686	2	62405057	A	0.234086	8.61928E-45	7213	197.1789433
Q9NYA1	SPHK1	rs12941069	17	76464503	T	-0.152241	1.486E-18	7213	77.27681801
Q9NZ08	ERAP1	rs30186	5	96788744	G	1.04161	1E-200	7213	1373.872631
Q9NZ52	GGA3	rs7222782	17	75275977	T	-0.209533	1.14691E-12	7213	50.57509931
Q9NZA1	CLIC5	rs35822882	6	45949262	T	-1.27056	1.0093E-121	7213	550.4410795
Q9NZC2	TREM2	rs143332484	6	41161469	T	-1.5304	2.94865E-80	7213	359.90797
Q9NZK5	ADA2	rs2231495	22	17188416	C	-0.837861	1E-200	7213	1373.872631
Q9NZP8	C1RL	rs11613834	12	7097267	A	0.361685	2.87512E-98	7213	442.6456441
Q9NZQ7	CD274	rs822342	9	5453973	T	-0.36538	3.28166E-83	7213	373.4726793
Q9NZU0	FLRT3	rs11908351	20	14705863	T	0.890955	1E-200	7213	1373.872631
Q9P0G3	KLK14	rs11666870	19	51095412	A	-0.244253	7.76786E-51	7213	224.8877096
Q9P0K1	ADAM22	rs2279542	7	87935182	C	0.446487	1.2016E-167	7213	761.6065494
Q9P121	NTM	rs2511781	11	131328850	T	0.621831	1E-200	7213	1373.872631
Q9P126	CLEC1B	rs521040	12	9995251	C	0.426407	2.2912E-115	7213	521.2248108
Q9P1W8	SIRPG	rs6043409	20	1635560	A	-0.492955	2.5293E-183	7213	833.7104378
Q9P2B2	PTGFRN	rs4233450	1	116947639	G	-1.0095	1E-200	7213	1373.872631
Q9P2E7	PCDH10	rs4864200	4	133546774	T	0.235852	1.87148E-44	7213	195.6360945
Q9P2T1	GMPR2	rs34354104	14	24238273	A	-0.967493	6.1413E-132	7213	597.4048832
Q9UBD3	XCL2	rs4656601	1	168538235	A	0.221446	7.5367E-27	7213	115.0857499
Q9UBG0	MRC2	rs146385050	17	62559897	A	-0.414284	3.38612E-88	7213	396.3766451
Q9UBM8	MGAT4C	rs61929388	12	85926355	T	0.237014	1.01327E-18	7213	78.03312512
Q9UBN6	TNFRSF10D	rs4460371	8	23134288	T	0.10224	6.83745E-10	7213	38.066487
Q9UBP4	DKK3	rs11022114	11	12017327	A	0.47715	4.9149E-164	7213	744.9957471
Q9UBQ7	GRHPR	rs4878690	9	37422659	T	0.367175	5.10625E-97	7213	436.9047279
Q9UBR2	CTSZ	rs75940688	20	59220098	A	-0.925535	3.12257E-34	7213	148.8308609
Q9UBS4	DNAJB11	rs56227532	3	186576030	C	-0.570804	2.49456E-76	7213	341.8728798

Q9UBU2	DKK2	rs6815075	4	107118897	A	-0.145507	1.82637E-08	7213	31.67091536
Q9UBV7	B4GALT7	rs59621415	5	177589795	G	0.473376	4.16251E-30	7213	129.9688883
Q9UBX1	CTSF	rs1044522	11	66568361	A	0.298029	2.72219E-53	7213	236.1467289
Q9UBX5	FBLN5	rs2267994	14	91890602	G	0.14798	1.1487E-17	7213	73.2388891
Q9UBX7	KLK11	rs62115743	19	51011618	T	-1.05334	1E-200	7213	1304.347828
Q9UBX8	B4GALT6	rs113222817	18	31636351	G	-1.51803	1E-200	7213	1340.386117
Q9UDY4	DNAJB4	rs7514180	1	77995251	A	-0.34905	1.92358E-67	7213	301.0725039
Q9UEF7	KL	rs9527032	13	33056892	C	0.213542	8.96809E-21	7213	87.37714341
Q9UEU0	VTI1B	rs10483801	14	67650289	A	0.167751	2.45789E-15	7213	62.65892191
Q9UGC7	MTRF1L	rs3757067	6	152984732	A	-0.123684	1.15529E-08	7213	32.5606721
Q9UGM5	FETUB	rs114780909	3	186642466	A	-1.91827	3.2598E-134	7213	607.8646615
Q9UGN4	CD300A	rs2272111	17	74473827	A	-0.872801	1E-200	7213	1373.872631
Q9UH65	SWAP70	rs415895	11	9748015	C	0.627812	1E-200	7213	1373.872631
Q9UHC6	CNTNAP2	rs10261105	7	145651617	A	0.754089	1E-200	7213	1373.872631
Q9UHF4	IL20RA	rs1775296	6	137005667	T	0.126066	8.91952E-12	7213	46.55252599
Q9UHF5	IL17B	rs6862301	5	149404127	A	0.189295	2.04402E-25	7213	108.542672
Q9UHG3	PCYOX1	rs2706762	2	70261338	T	-0.952514	1E-200	7213	1373.872631
Q9UHL4	DPP7	rs4880198	9	137084985	T	-0.523638	1.4574E-157	7213	715.2314494
Q9UHN6	CEMIP2	rs1410988	9	71650180	G	-0.239721	1.36371E-39	7213	173.3628751
Q9UHX3	ADGRE2	rs67023356	19	14396821	G	0.490378	6.2756E-143	7213	647.9375433
Q9UI08	EVL	rs34612764	14	99946845	A	0.161853	1.54481E-20	7213	86.30163195
Q9UI42	CPA4	rs34587586	7	130298758	T	-0.989715	1E-200	7213	1373.872631
Q9UIK4	DAPK2	rs55986634	15	63983446	A	0.160119	1.27735E-21	7213	91.23265168
Q9UIV8	SERPINB13	rs77157727	18	63586727	A	-0.498763	5.48141E-25	7213	106.5876368
Q9UIW2	PLXNA1	rs9822602	3	126976937	G	-0.431118	8.1047E-135	7213	610.6437359
Q9UJ70	NAGK	rs11680831	2	71065392	C	-0.513707	1E-200	7213	929.4154609
Q9UJA9	ENPP5	rs1047153	6	46161008	C	0.799816	1E-200	7213	1373.872631
Q9UJC5	SH3BGRL2	rs2245410	6	79616140	A	-0.426901	2.6718E-149	7213	677.2323359
Q9UJJ9	GNPTG	rs6600137	16	1357440	A	0.685275	1.2523E-149	7213	678.7456609
Q9UJY5	GGA1	rs12483880	22	37655992	T	0.0931995	2.54448E-08	7213	31.02712105
Q9UK23	NAGPA	rs12599777	16	5029465	G	-0.574707	4.7505E-170	7213	772.6584319
Q9UK55	SERPINA10	rs12434093	14	94288847	C	0.865278	1E-200	7213	1373.872631
Q9UK85	DKKL1	rs1465697	19	49333989	T	-0.160239	5.70402E-17	7213	70.07678931
Q9UKG9	CROT	rs31659	7	87411313	C	-0.60755	2.5491E-114	7213	516.4155952
Q9UKJ1	PILRA	rs1859788	7	100374211	A	1.0957	1E-200	7213	1373.872631
Q9UKK9	NUDT5	rs7099650	10	12159177	T	-0.100384	2.04954E-09	7213	35.92601356
Q9UKR3	KLK13	rs2569459	19	51036176	T	-0.393619	2.0781E-116	7213	526.0161059
Q9UKZ9	PCOLCE2	rs11716897	3	142886714	A	0.491823	4.5515E-184	7213	837.1364312
Q9UL25	RAB21	rs2864808	12	71746926	G	0.20531	1.00895E-18	7213	78.04156466
Q9UL46	PSME2	rs2236352	14	24141524	C	0.312178	5.72232E-62	7213	275.9528072
Q9UM47	NOTCH3	rs4808238	19	15192918	A	0.217443	9.6473E-21	7213	87.23275069
Q9UM54	MYO6	rs41269323	6	75914821	A	-0.897535	8.78473E-28	7213	119.3486829
Q9UMF0	ICAM5	rs281439	19	10289434	G	-0.786982	1E-200	7213	1373.872631
Q9UMR2	DDX19B	rs138325325	16	70077427	A	-0.715572	4.5225E-23	7213	97.84586563
Q9UMS0	NFU1	rs74637005	2	69423598	A	-0.302771	3.85441E-12	7213	48.19718648
Q9UN19	DAPP1	rs3822103	4	99833508	T	-0.343316	6.97983E-98	7213	440.8757723
Q9UN37	VPS4A	rs9924894	16	69333482	G	0.133523	2.78066E-12	7213	48.83754228
Q9UNA0	ADAMTS5	rs2830585	21	26932893	T	-0.912609	1E-200	7213	1373.872631
Q9UNE0	EDAR	rs140661471	2	108987283	A	1.5456	7.1871E-101	7213	454.6022461
Q9UNK0	STX8	rs35082231	17	9437887	T	0.329143	6.65652E-65	7213	289.4185601
Q9UNP9	PPIE	rs1046988	1	39753393	T	-0.592572	1E-200	7213	1289.112883
Q9UNW1	MINPP1	rs59980852	10	87515989	T	-0.150282	1.75397E-19	7213	81.49847344
Q9UNZ2	NSFL1C	rs4814391	20	1622497	T	0.1842	4.29185E-14	7213	57.03013392
Q9UQ53	MGAT4B	rs113756550	5	179801377	A	-1.57189	5.0655E-166	7213	754.1335457
Q9UQN3	CHMP2B	rs300978	3	87274184	T	0.556863	1E-200	7213	1130.44476
Q9Y240	CLEC11A	rs116924815	19	50727476	T	1.28774	3.4752E-149	7213	676.7072875
Q9Y259	CHKB	rs5770922	22	50584979	G	-0.503567	6.53705E-39	7213	170.2462566
Q9Y274	ST3GAL6	rs28489284	3	98793696	A	-1.6391	1E-200	7213	1373.872631
Q9Y275	TNFSF13B	rs374039502	13	108308037	A	0.345389	5.97124E-11	7213	42.82990241
Q9Y286	SIGLEC7	rs140185670	19	51142883	C	-0.812856	7.9656E-140	7213	633.6673083
Q9Y294	ASF1A	rs4946366	6	118811617	T	0.130473	1.53505E-09	7213	36.48932051
Q9Y2C2	UST	rs7764228	6	148737672	A	0.15576	1.52475E-20	7213	86.32747998
Q9Y2E5	MAN2B2	rs2301790	4	6598285	G	0.925048	1E-200	7213	1373.872631
Q9Y2I2	NTNG1	rs115668827	1	107135646	C	1.29214	1E-200	7213	1311.877784
Q9Y2Y8	PRG3	rs12575356	11	57375990	T	-0.146239	1.85623E-08	7213	31.63942122
Q9Y2Z0	SUGT1	rs185192949	13	52645515	T	-0.263108	4.75953E-10	7213	38.77346962
Q9Y336	SIGLEC9	rs2075803	19	51125272	A	1.03012	1E-200	7213	1373.872631
Q9Y376	CAB39	rs7604335	2	230754380	C	-0.232338	2.13806E-33	7213	145.0088785
Q9Y3A5	SBDS	rs79344818	7	66988489	G	-1.18714	2.2224E-161	7213	732.7840437
Q9Y3B8	REXO2	rs4938104	11	114427673	G	-0.285735	2.42718E-34	7213	149.3313996
Q9Y3C6	PPIL1	rs12194408	6	36871822	G	-1.35786	1.1757E-174	7213	793.8449706
Q9Y3C8	UFC1	rs79923112	1	161158854	C	0.453104	1.10113E-09	7213	37.13702476
Q9Y3D6	FIS1	rs142501950	7	101230277	G	-0.70054	1.44712E-78	7213	352.1428643
Q9Y3E1	HDGFL3	rs28479338	15	83071593	C	0.213411	6.13152E-12	7213	47.28707479
Q9Y3E2	BOLA1	rs1044808	1	149900352	C	1.08971	1E-200	7213	1328.637286
Q9Y3E7	CHMP3	rs59613942	2	86552964	G	0.307012	1.55699E-40	7213	177.6786372
Q9Y4D7	PLXND1	rs1108584	3	129601458	G	-0.554971	3.79711E-96	7213	432.9011716

Q9Y4E8	USP15	rs11612349	12	62323777	A	0.851842	1E-200	7213	1104.802719
Q9Y4P3	TBL2	rs76029572	7	73578528	G	0.461667	1.68683E-25	7213	108.9233673
Q9Y570	PPME1	rs76970567	11	74205493	G	-0.298051	5.19944E-16	7213	65.71929251
Q9Y5C1	ANGPTL3	rs10889333	1	62491359	A	-0.33608	2.70082E-84	7213	378.4542567
Q9Y5P4	CERT1	rs5744681	5	75584525	A	0.259632	7.2043E-19	7213	78.70691363
Q9Y5W5	WIF1	rs462847	12	64920170	T	-0.10588	1.94791E-10	7213	40.51822131
Q9Y5X2	SNX8	rs28514741	7	2249281	C	-0.908082	1.92345E-31	7213	136.0728247
Q9Y5Y7	LYVE1	rs114758648	11	10593808	A	0.490929	1.0528E-32	7213	141.8423804
Q9Y617	PSAT1	rs2277148	9	78297199	G	-0.390402	1.48868E-85	7213	384.2356757
Q9Y646	CPQ	rs148684432	8	97029409	A	-1.69904	1.3971E-121	7213	549.7919939
Q9Y653	ADGRG1	rs1801257	16	57655473	G	0.162996	7.20074E-23	7213	96.92489996
Q9Y662	HS3ST3B1	rs62056073	17	14345059	G	-0.464273	2.72577E-16	7213	66.99224703
Q9Y663	HS3ST3A1	rs139707331	17	13657100	A	0.419599	3.26539E-09	7213	35.01879013
Q9Y680	FKBP7	rs1863671	2	178477603	C	0.767546	1E-200	7213	1305.048835
Q9Y696	CLIC4	rs4649017	1	24746529	C	-0.258788	2.96283E-53	7213	235.9780213
Q9Y6F6	IRAG1	rs1075768	11	10678202	A	0.214955	1.23405E-36	7213	159.8275223
Q9Y6N7	ROBO1	rs3773232	3	78725105	T	0.340381	4.46402E-60	7213	267.2708522
Q9Y6U3	SCIN	rs7803810	7	12568345	A	-0.106183	2.61678E-10	7213	39.94154366
Q9Y6Z7	COLEC10	rs2465383	8	119067509	G	-0.121627	2.78616E-13	7213	53.35348862
P00747	PLG	rs537579467	6	160734232	C	1.7343	8.8024E-133	7213	601.2836262

Table S2 Genetic instruments of eight prioritized proteins for replication

protein	SNP	chromosome	position	effect allele	beta	P value	F statistics
COLEC11	rs6542680	2	3592552	C	0.86	1E-200	1479.741295
IRF3	rs10415576	19	49661133	C	-0.07	8.51138E-18	73.83067039
LUM	rs142086573	12	90914869	T	0.28	2.04174E-20	85.75010122
LUM	rs10459147	12	91015895	T	-0.18	3.38844E-14	57.49497327
LUM	rs77751442	12	91091238	T	-0.38	6.45654E-38	165.6926682
POSTN	rs139240369	13	37472287	T	-0.2	5.01187E-15	61.25587736
POSTN	rs9532075	13	37530059	T	0.16	4.0738E-83	373.0413737
RPN1	rs7650156	3	128642712	T	0.15	1.28825E-78	352.3747917
RSPO3	rs9285458	6	127142500	A	0.2336	7.426E-187	849.8756599
SARS2	rs7508539	19	38926800	G	0.11	1.34896E-40	177.9638874
VAT1	rs143297645	17	43018676	T	-0.98	3.16228E-18	75.78541802
VAT1	rs3951053	17	43322922	G	-0.07	6.45654E-13	51.70295738

Table S3 Genetic instruments of varicose veins used in bidirectional MR

SNP	chromosome	position	effect allele	beta	P value	sample size	F statistics
rs7524424	1	8113309	T	0.074	1.229E-08	459702	32.40236686
rs11121615	1	10765520	T	-0.2478	6.18E-104	459702	472.4903047
rs58062906	1	42972728	T	0.0768	9.919E-09	459702	32.84829583
rs11589479	1	155060832	A	0.0849	1.053E-08	459702	32.90727721
rs4846565	1	219548762	A	0.0734	5.839E-11	459702	42.94929847
rs2089657	2	30247312	T	-0.0965	1.522E-12	459702	50.3473724
rs4296409	2	68212036	A	0.1392	5.41E-34	459702	146.5152363
rs4849007	2	112098588	A	-0.0741	9.862E-12	459702	46.21504924
rs13430544	2	172285832	A	0.1247	3.298E-13	459702	53.17906364
rs1873944	3	8511401	C	-0.0616	1.032E-08	459702	32.53223594
rs7614922	3	128519379	C	0.1365	3.605E-16	459702	66.80859837
rs10049090	3	157079913	A	-0.0615	3.523E-08	459702	30.15186543
rs496104	3	170733180	A	0.0865	2.656E-08	459702	30.74560322
rs28558138	4	26816458	C	-0.133	6.712E-34	459702	146.1900826
rs11728719	4	185775018	C	-0.0733	5.015E-09	459702	34.386496
rs10474599	5	79915807	C	-0.0656	1.773E-09	459702	36.22052016
rs2250127	5	128053152	A	0.1566	1.242E-31	459702	136.5758521
rs1468550	5	130576973	C	0.1051	1.702E-09	459702	36.06860408
rs12332693	5	158800890	A	-0.113	1.3E-25	459702	109.473594
rs111797764	6	12463340	T	0.1292	2.043E-10	459702	40.50726783
rs7773004	6	26267527	G	-0.0826	2.08E-14	459702	58.4941701
rs2844519	6	31403653	C	-0.0851	7.396E-10	459702	38.02777778
rs62401797	6	46989654	G	-0.1299	3.989E-08	459702	30.04149976
rs2800709	6	127118152	G	-0.0699	6.901E-11	459702	42.67630361
rs75522736	6	134694783	G	-0.0891	6.701E-09	459702	33.4744898
rs9719461	7	593078	T	0.0614	4.113E-08	459702	30.05389031
rs17559301	7	129891853	C	-0.1207	1.543E-10	459702	40.78410459
rs13282633	8	6789355	C	-0.0637	9.346E-09	459702	32.93312231
rs75731123	8	10716188	G	-0.1012	6.357E-10	459702	38.0779298
rs1824999	8	60297557	T	0.0589	4.328E-08	459702	30.30142371
rs62512472	8	86583362	A	0.0705	1.238E-10	459702	41.07644628
rs78216177	9	232148	C	0.0919	1.68E-08	459702	31.78745907
rs6479353	9	91530312	A	0.0744	4.487E-11	459702	43.34998825
rs10124673	9	115524523	T	0.0753	4.552E-10	459702	38.72747763
rs635634	9	133279427	C	-0.0851	5.36E-10	459702	38.58495391
rs3740357	10	62049201	C	-0.1796	1.678E-11	459702	45.24703671
rs61863928	10	62689789	T	-0.0655	1.282E-08	459702	32.44045369
rs7951820	11	1553838	C	-0.0823	7.921E-10	459702	37.72159724
rs1372809	11	10222558	G	0.0626	5.281E-09	459702	34.22796751
rs34516251	11	128440451	A	-0.0676	1.314E-08	459702	32.27003743
rs2544026	12	47792780	A	0.1202	6.845E-23	459702	97.07094867
rs3742062	12	50735049	G	0.0675	7.678E-09	459702	33.28402367
rs4772696	13	105612218	G	0.074	3.031E-11	459702	44.44444444
rs77651953	14	68495549	T	-0.0718	2.022E-08	459702	31.46508789
rs16943741	15	85735248	G	-0.0709	3.826E-11	459702	43.90610534
rs4561414	15	95629095	A	-0.1506	3.113E-11	459702	44.01474898
rs4786476	16	4229044	A	0.0628	1.862E-08	459702	31.44005102
rs752843	16	88702696	T	0.0973	9.65E-13	459702	51.1856077
rs2002833	16	88775709	A	-0.1791	1.369E-56	459702	251.2084736
rs67328311	17	7449657	T	-0.0664	1.585E-09	459702	36.43768595
rs236548	17	70235111	A	-0.1001	1.618E-15	459702	63.11419753
rs2058744	17	72044167	A	-0.0865	1.488E-15	459702	64.14823388
rs3745318	19	16325451	C	-0.0732	7.388E-09	459702	33.22115444
rs10422861	19	33403940	T	0.0617	4.799E-08	459702	29.81353277
rs2638282	19	48710576	A	0.0703	7.957E-11	459702	42.3704561

rs12625547	20	51538108	G	-0.1426	3.282E-23	459702	98.06500772
rs6062618	20	64051176	G	-0.1074	1.532E-18	459702	77.49771567
rs4822429	22	20668581	C	-0.0627	1.844E-08	459702	31.90723156

Table S4 GWAS catalog of 525 diseases used for phenome-wide Mendelian Randomization analysis

GWAS ID (IEU OpenGWAS project)	disease	ICD-10 code	number of cases	sample size	prevalence
ukb-d-A41	Diagnoses - main ICD10: A41 Other septicaemia	A41	1096	361194	0.30%
ukb-b-3683	Diagnoses - main ICD10: B34.9 Viral infection, unspecified	B34.9	1038	463010	0.22%
ukb-a-517	Diagnoses - main ICD10: B37 Candidiasis	B37	153	337199	0.05%
ukb-b-4423	Diagnoses - secondary ICD10: B95.6 Staphylococcus aureus as the cause of diseases classified to other chapters	B95.6	1716	463010	0.37%
ukb-b-8877	Diagnoses - secondary ICD10: B96.2 Escherichia coli [E. coli] as the cause of diseases classified to other chapters	B96.2	1551	463010	0.33%
ukb-b-20513	Diagnoses - secondary ICD10: B96.8 Other specified bacterial agents as the cause of diseases classified to other chapters	B96.8	4564	463010	0.99%
ukb-d-C18	Diagnoses - main ICD10: C18 Malignant neoplasm of colon	C18	2226	361194	0.62%
ukb-b-15775	Type of cancer: ICD10: C18.7 Sigmoid colon	C18.7	1241	463010	0.27%
ukb-b-1251	Type of cancer: ICD10: C20 Malignant neoplasm of rectum	C20	1470	463010	0.32%
ukb-d-C34	Diagnoses - main ICD10: C34 Malignant neoplasm of bronchus and lung	C34	1427	361194	0.40%
ukb-b-18798	Type of cancer: ICD10: C34.1 Upper lobe, bronchus or lung	C34.1	1002	463010	0.22%
ukb-d-40001_C349	Underlying (primary) cause of death: ICD10: C34.9 Bronchus or lung, unspecified	C34.9	1100	9092	12.10%
ukb-d-C43	Diagnoses - main ICD10: C43 Malignant melanoma of skin	C43	1672	361194	0.46%
ukb-b-2750	Type of cancer: ICD10: C43.7 Malignant melanoma of lower limb, including hip	C43.7	1058	463010	0.23%
ukb-a-518	Diagnoses - main ICD10: C44 Other malignant neoplasms of skin	C44	4826	337199	1.43%
ukb-b-10248	Type of cancer: ICD10: C44.3 Skin of other and unspecified parts of face	C44.3	6034	463010	1.30%
ukb-b-17174	Type of cancer: ICD10: C44.4 Skin of scalp and neck	C44.4	1225	463010	0.26%
ukb-b-15685	Type of cancer: ICD10: C44.5 Skin of trunk	C44.5	2605	463010	0.56%
ukb-b-19078	Type of cancer: ICD10: C44.6 Skin of upper limb, including shoulder	C44.6	1375	463010	0.30%
ukb-b-6994	Type of cancer: ICD10: C44.7 Skin of lower limb, including hip	C44.7	1067	463010	0.23%
ukb-b-8416	Type of cancer: ICD10: C44.9 Malignant neoplasm of skin, unspecified	C44.9	3372	463010	0.73%
ukb-a-519	Diagnoses - main ICD10: C50 Malignant neoplasm of breast	C50	5510	337199	1.63%
ukb-b-12115	Diagnoses - main ICD10: C50.4 Upper-outer quadrant of breast	C50.4	1445	463010	0.31%
ukb-b-19041	Type of cancer: ICD10: C50.9 Breast, unspecified	C50.9	6192	463010	1.34%
ukb-b-14956	Type of cancer: ICD10: C54.1 Endometrium	C54.1	1494	463010	0.32%
ukb-b-18157	Type of cancer: ICD10: C56 Malignant neoplasm of ovary	C56	1087	463010	0.23%
ukb-a-520	Diagnoses - main ICD10: C61 Malignant neoplasm of prostate	C61	2273	337199	0.67%
ukb-b-1316	Type of cancer: ICD10: C64 Malignant neoplasm of kidney, except renal pelvis	C64	1114	463010	0.24%
ukb-d-C67	Diagnoses - main ICD10: C67 Malignant neoplasm of bladder	C67	1554	361194	0.43%
ukb-b-5421	Diagnoses - main ICD10: C67.9 Bladder, unspecified	C67.9	1380	463010	0.30%
ukb-b-1702	Diagnoses - secondary ICD10: C77.3 Axillary and upper limb lymph nodes	C77.3	1580	463010	0.34%
ukb-d-C78	Diagnoses - main ICD10: C78 Secondary malignant neoplasm of respiratory and digestive organs	C78	1378	361194	0.38%
ukb-b-16713	Diagnoses - secondary ICD10: C78.7 Secondary malignant neoplasm of liver	C78.7	1139	463010	0.25%
ukb-d-C79	Diagnoses - main ICD10: C79 Secondary malignant neoplasm of other sites	C79	1099	361194	0.30%
ukb-d-D05	Diagnoses - main ICD10: D05 Carcinoma in situ of breast	D05	1454	361194	0.40%
ukb-b-4674	Type of cancer: ICD10: D05.1 Intraductal carcinoma in situ	D05.1	1917	463010	0.41%
ukb-b-918	Type of cancer: ICD10: D06.9 Cervix, unspecified	D06.9	3175	463010	0.69%
ukb-a-521	Diagnoses - main ICD10: D12 Benign neoplasm of colon_rectum_anus and anal canal	D12	4794	337199	1.42%
ukb-b-10623	Diagnoses - main ICD10: D12.5 Sigmoid colon	D12.5	2447	463010	0.53%
ukb-b-19266	Diagnoses - main ICD10: D12.6 Colon, unspecified	D12.6	1870	463010	0.40%
ukb-b-9321	Diagnoses - main ICD10: D12.8 Rectum	D12.8	1509	463010	0.33%
ukb-d-D17	Diagnoses - main ICD10: D17 Benign lipomatous neoplasm	D17	4314	361194	1.19%
ukb-b-2561	Diagnoses - main ICD10: D17.1 Benign lipomatous neoplasm of skin and subcutaneous tissue of trunk	D17.1	2006	463010	0.43%
ukb-b-212	Diagnoses - main ICD10: D17.2 Benign lipomatous neoplasm of skin and subcutaneous tissue of limbs	D17.2	1514	463010	0.33%
ukb-d-D22	Diagnoses - main ICD10: D22 Melanocytic naevi	D22	3501	361194	0.97%
ukb-b-3596	Diagnoses - main ICD10: D22.3 Melanocytic naevi of other and unspecified parts of face	D22.3	1472	463010	0.32%
ukb-d-D23	Diagnoses - main ICD10: D23 Other benign neoplasms of skin	D23	3085	361194	0.85%
ukb-b-8549	Diagnoses - main ICD10: D24 Benign neoplasm of breast	D24	1244	463010	0.27%
ukb-a-522	Diagnoses - main ICD10: D25 Leiomyoma of uterus	D25	4351	337199	1.29%
ukb-b-6528	Diagnoses - secondary ICD10: D25.9 Leiomyoma of uterus, unspecified	D25.9	3024	463010	0.65%
ukb-d-D50	Diagnoses - main ICD10: D50 Iron deficiency anaemia	D50	3222	361194	0.89%

ukb-b-3355	Diagnoses - main ICD10: D50.9 Iron deficiency anaemia, unspecified	D50.9	2066	463010	0.45%
ukb-d-D64	Diagnoses - main ICD10: D64 Other anaemias	D64	3702	361194	1.02%
ukb-b-14765	Diagnoses - main ICD10: D64.9 Anaemia, unspecified	D64.9	3709	463010	0.80%
ukb-b-3229	Diagnoses - secondary ICD10: D70 Agranulocytosis	D70	1108	463010	0.24%
ukb-a-523	Diagnoses - main ICD10: E03 Other hypothyroidism	E03	89	337199	0.03%
ukb-b-4226	Diagnoses - secondary ICD10: E03.9 Hypothyroidism, unspecified	E03.9	9674	463010	2.09%
ukb-a-524	Diagnoses - main ICD10: E04 Other non-toxic goitre	E04	745	337199	0.22%
ukb-b-10694	Diagnoses - secondary ICD10: E10.9 Without complications	E10.9	2076	463010	0.45%
ukb-b-970	Diagnoses - secondary ICD10: E11.9 Without complications	E11.9	12045	463010	2.60%
ukb-b-17532	Diagnoses - secondary ICD10: E14.9 Without complications	E14.9	1305	463010	0.28%
ukb-b-15541	Diagnoses - secondary ICD10: E66.9 Obesity, unspecified	E66.9	4688	463010	1.01%
ukb-b-12651	Diagnoses - secondary ICD10: E78.0 Pure hypercholesterolaemia	E78.0	22622	463010	4.89%
ukb-b-17462	Diagnoses - secondary ICD10: E78.5 Hyperlipidaemia, unspecified	E78.5	3439	463010	0.74%
ukb-a-357	Underlying (primary) cause of death: ICD10: E85.4 Organ-limited amyloidosis	E85.4	7	7637	0.09%
ukb-b-16319	Diagnoses - secondary ICD10: E86 Volume depletion	E86	1149	463010	0.25%
ukb-b-1176	Diagnoses - secondary ICD10: F10.0 Acute intoxication	F10.0	1204	463010	0.26%
ukb-b-16056	Diagnoses - secondary ICD10: F10.1 Harmful use	F10.1	1802	463010	0.39%
ukb-b-19651	Diagnoses - secondary ICD10: F10.2 Dependence syndrome	F10.2	1290	463010	0.28%
ukb-b-2964	Diagnoses - secondary ICD10: F17.1 Harmful use	F17.1	1483	463010	0.32%
ukb-a-525	Diagnoses - main ICD10: F31 Bipolar affective disorder	F31	303	337199	0.09%
ukb-b-20045	Diagnoses - secondary ICD10: F32.9 Depressive episode, unspecified	F32.9	5842	463010	1.26%
ukb-b-11311	Diagnoses - secondary ICD10: F41.9 Anxiety disorder, unspecified	F41.9	1523	463010	0.33%
ukb-a-526	Diagnoses - main ICD10: F43 Reaction to severe stress and adjustment disorders	F43	182	337199	0.05%
ukb-b-1600	Diagnoses - secondary ICD10: G40.9 Epilepsy, unspecified	G40.9	2326	463010	0.50%
ukb-d-G43	Diagnoses - main ICD10: G43 Migraine	G43	1072	361194	0.30%
ukb-b-13190	Diagnoses - main ICD10: G43.9 Migraine, unspecified	G43.9	1001	463010	0.22%
ukb-d-G45	Diagnoses - main ICD10: G45 Transient cerebral ischaemic attacks and related syndromes	G45	1744	361194	0.48%
ukb-b-13391	Diagnoses - main ICD10: G45.9 Transient cerebral ischaemic attack, unspecified	G45.9	1364	463010	0.29%
ukb-a-527	Diagnoses - main ICD10: G47 Sleep disorders	G47	2025	337199	0.60%
ukb-b-16781	Diagnoses - main ICD10: G47.3 Sleep apnoea	G47.3	2320	463010	0.50%
ukb-b-1220	Diagnoses - secondary ICD10: G55.1 Nerve root and plexus compressions in intervertebral disk disorders	G55.1	2298	463010	0.50%
ukb-a-528	Diagnoses - main ICD10: G56 Mononeuropathies of upper limb	G56	5886	337199	1.75%
ukb-b-3965	Diagnoses - main ICD10: G56.0 Carpal tunnel syndrome	G56.0	8289	463010	1.79%
ukb-d-G57	Diagnoses - main ICD10: G57 Mononeuropathies of lower limb	G57	1111	361194	0.31%
ukb-d-H00	Diagnoses - main ICD10: H00 Hordeolum and chalazion	H00	1467	361194	0.41%
ukb-b-4637	Diagnoses - main ICD10: H00.1 Chalazion	H00.1	1551	463010	0.33%
ukb-d-H02	Diagnoses - main ICD10: H02 Other disorders of eyelid	H02	4294	361194	1.19%
ukb-b-9565	Diagnoses - main ICD10: H02.8 Other specified disorders of eyelid	H02.8	1739	463010	0.38%
ukb-d-H04	Diagnoses - main ICD10: H04 Disorders of lachrymal system	H04	1476	361194	0.41%
ukb-a-529	Diagnoses - main ICD10: H25 Senile cataract	H25	2651	337199	0.79%
ukb-b-2285	Diagnoses - main ICD10: H25.0 Senile incipient cataract	H25.0	1061	463010	0.23%
ukb-b-3095	Diagnoses - main ICD10: H25.1 Senile nuclear cataract	H25.1	1960	463010	0.42%
ukb-a-530	Diagnoses - main ICD10: H26 Other cataract	H26	6025	337199	1.79%
ukb-b-12397	Diagnoses - main ICD10: H26.9 Cataract, unspecified	H26.9	8890	463010	1.92%
ukb-d-H33	Diagnoses - main ICD10: H33 Retinal detachments and breaks	H33	2671	361194	0.74%
ukb-d-H35	Diagnoses - main ICD10: H35 Other retinal disorders	H35	1551	361194	0.43%
ukb-d-H40	Diagnoses - main ICD10: H40 Glaucoma	H40	1715	361194	0.47%
ukb-a-531	Diagnoses - main ICD10: I10 Essential (primary) hypertension	I10	500	337199	0.15%
ukb-b-19955	Diagnoses - secondary ICD10: I12.0 Hypertensive renal disease with renal failure	I12.0	1146	463010	0.25%
ukb-a-532	Diagnoses - main ICD10: I20 Angina pectoris	I20	4837	337199	1.43%
ukb-b-10756	Diagnoses - secondary ICD10: I20.0 Unstable angina	I20.0	1543	463010	0.33%
ukb-b-14206	Diagnoses - main ICD10: I20.9 Angina pectoris, unspecified	I20.9	4256	463010	0.92%
ukb-a-533	Diagnoses - main ICD10: I21 Acute myocardial infarction	I21	3927	337199	1.16%

ukb-b-453	Diagnoses - main ICD10: I21.0 Acute transmural myocardial infarction of anterior wall	I21.0	1294	463010	0.28%
ukb-b-5126	Diagnoses - main ICD10: I21.1 Acute transmural myocardial infarction of inferior wall	I21.1	1673	463010	0.36%
ukb-b-3469	Diagnoses - main ICD10: I21.9 Acute myocardial infarction, unspecified	I21.9	2321	463010	0.50%
ukb-a-534	Diagnoses - main ICD10: I25 Chronic ischaemic heart disease	I25	8755	337199	2.60%
ukb-b-1668	Diagnoses - main ICD10: I25.1 Atherosclerotic heart disease	I25.1	12171	463010	2.63%
ukb-b-16662	Diagnoses - secondary ICD10: I25.2 Old myocardial infarction	I25.2	3340	463010	0.72%
ukb-b-16606	Diagnoses - secondary ICD10: I25.8 Other forms of chronic ischaemic heart disease	I25.8	5738	463010	1.24%
ukb-b-3983	Diagnoses - main ICD10: I25.9 Chronic ischaemic heart disease, unspecified	I25.9	1195	463010	0.26%
ukb-d-I26	Diagnoses - main ICD10: I26 Pulmonary embolism	I26	2118	361194	0.59%
ukb-b-18366	Diagnoses - main ICD10: I26.9 Pulmonary embolism without mention of acute cor pulmonale	I26.9	1846	463010	0.40%
ukb-a-535	Diagnoses - main ICD10: I30 Acute pericarditis	I30	107	337199	0.03%
ukb-b-13525	Diagnoses - secondary ICD10: I34.0 Mitral (valve) insufficiency	I34.0	1021	463010	0.22%
ukb-d-I47	Diagnoses - main ICD10: I47 Paroxysmal tachycardia	I47	1685	361194	0.47%
ukb-b-11748	Diagnoses - main ICD10: I47.1 Supraventricular tachycardia	I47.1	1306	463010	0.28%
ukb-a-536	Diagnoses - main ICD10: I48 Atrial fibrillation and flutter	I48	3818	337199	1.13%
ukb-d-I50	Diagnoses - main ICD10: I50 Heart failure	I50	1088	361194	0.30%
ukb-b-10555	Diagnoses - secondary ICD10: I50.1 Left ventricular failure	I50.1	2046	463010	0.44%
ukb-b-16292	Diagnoses - secondary ICD10: I51.7 Cardiomegaly	I51.7	1326	463010	0.29%
ukb-d-I63	Diagnoses - main ICD10: I63 Cerebral infarction	I63	2353	361194	0.65%
ukb-b-19350	Diagnoses - main ICD10: I63.9 Cerebral infarction, unspecified	I63.9	1420	463010	0.31%
ukb-b-4929	Diagnoses - secondary ICD10: I73.9 Peripheral vascular disease, unspecified	I73.9	1456	463010	0.31%
ukb-a-537	Diagnoses - main ICD10: I80 Phlebitis and thrombophlebitis	I80	1613	337199	0.48%
ukb-b-12267	Diagnoses - main ICD10: I80.2 Phlebitis and thrombophlebitis of other deep vessels of lower extremities	I80.2	1832	463010	0.40%
ukb-a-539	Diagnoses - main ICD10: I84 Haemorrhoids	I84	8190	337199	2.43%
ukb-b-14416	Diagnoses - main ICD10: I84.1 Internal haemorrhoids with other complications	I84.1	1549	463010	0.33%
ukb-b-1259	Diagnoses - secondary ICD10: I84.2 Internal haemorrhoids without complication	I84.2	1229	463010	0.27%
ukb-b-1228	Diagnoses - secondary ICD10: I84.6 Residual haemorrhoidal skin tags	I84.6	1710	463010	0.37%
ukb-b-7444	Diagnoses - main ICD10: I84.8 Unspecified haemorrhoids with other complications	I84.8	2321	463010	0.50%
ukb-b-11261	Diagnoses - main ICD10: I84.9 Unspecified haemorrhoids without complication	I84.9	6198	463010	1.34%
ukb-b-8855	Diagnoses - secondary ICD10: I95.9 Hypotension, unspecified	I95.9	1666	463010	0.36%
ukb-d-J18	Diagnoses - main ICD10: J18 Pneumonia, organism unspecified	J18	4630	361194	1.28%
ukb-b-1761	Diagnoses - secondary ICD10: J18.1 Lobar pneumonia, unspecified	J18.1	1045	463010	0.23%
ukb-b-15606	Diagnoses - main ICD10: J18.9 Pneumonia, unspecified	J18.9	1611	463010	0.35%
ukb-a-540	Diagnoses - main ICD10: J22 Unspecified acute lower respiratory infection	J22	1992	337199	0.59%
ukb-d-J32	Diagnoses - main ICD10: J32 Chronic sinusitis	J32	1179	361194	0.33%
ukb-a-541	Diagnoses - main ICD10: J33 Nasal polyp	J33	1637	337199	0.49%
ukb-b-7211	Diagnoses - main ICD10: J33.9 Nasal polyp, unspecified	J33.9	1373	463010	0.30%
ukb-a-542	Diagnoses - main ICD10: J34 Other disorders of nose and nasal sinuses	J34	3433	337199	1.02%
ukb-b-3770	Diagnoses - main ICD10: J34.2 Deviated nasal septum	J34.2	3256	463010	0.70%
ukb-b-5815	Diagnoses - secondary ICD10: J34.3 Hypertrophy of nasal turbinates	J34.3	1208	463010	0.26%
ukb-b-19045	Diagnoses - main ICD10: J34.8 Other specified disorders of nose and nasal sinuses	J34.8	1567	463010	0.34%
ukb-d-J38	Diagnoses - main ICD10: J38 Diseases of vocal cords and larynx, not elsewhere classified	J38	1168	361194	0.32%
ukb-a-543	Diagnoses - main ICD10: J44 Other chronic obstructive pulmonary disease	J44	874	337199	0.26%
ukb-b-16751	Diagnoses - secondary ICD10: J44.9 Chronic obstructive pulmonary disease, unspecified	J44.9	3871	463010	0.84%
ukb-d-J45	Diagnoses - main ICD10: J45 Asthma	J45	1693	361194	0.47%
ukb-b-17219	Diagnoses - secondary ICD10: J45.9 Asthma, unspecified	J45.9	18988	463010	4.10%
ukb-a-358	Underlying (primary) cause of death: ICD10: J84.1 Other interstitial pulmonary diseases with fibrosis	J84.1	96	7637	1.26%
ukb-b-9925	Diagnoses - secondary ICD10: J90 Pleural effusion, not elsewhere classified	J90	2411	463010	0.52%
ukb-d-K01	Diagnoses - main ICD10: K01 Embedded and impacted teeth	K01	1443	361194	0.40%
ukb-b-7534	Diagnoses - main ICD10: K01.1 Impacted teeth	K01.1	1596	463010	0.34%
ukb-d-K02	Diagnoses - main ICD10: K02 Dental caries	K02	2110	361194	0.58%
ukb-b-4770	Diagnoses - main ICD10: K02.9 Dental caries, unspecified	K02.9	1997	463010	0.43%
ukb-d-K04	Diagnoses - main ICD10: K04 Diseases of pulp and periapical tissues	K04	1609	361194	0.45%

ukb-d-K08	Diagnoses - main ICD10: K08 Other disorders of teeth and supporting structures	K08	1838	361194	0.51%
ukb-b-11771	Diagnoses - main ICD10: K08.3 Retained dental root	K08.3	1039	463010	0.22%
ukb-d-K13	Diagnoses - main ICD10: K13 Other diseases of lip and oral mucosa	K13	2157	361194	0.60%
ukb-b-18785	Diagnoses - main ICD10: K13.7 Other and unspecified lesions of oral mucosa	K13.7	1124	463010	0.24%
ukb-a-544	Diagnoses - main ICD10: K20 Oesophagitis	K20	3369	337199	1.00%
ukb-a-545	Diagnoses - main ICD10: K21 Gastro-oesophageal reflux disease	K21	7282	337199	2.16%
ukb-b-10144	Diagnoses - secondary ICD10: K21.0 Gastro-oesophageal reflux disease with oesophagitis	K21.0	2241	463010	0.48%
ukb-b-1386	Diagnoses - main ICD10: K21.9 Gastro-oesophageal reflux disease without oesophagitis	K21.9	4651	463010	1.00%
ukb-a-546	Diagnoses - main ICD10: K22 Other diseases of oesophagus	K22	3460	337199	1.03%
ukb-b-13731	Diagnoses - main ICD10: K22.1 Ulcer of oesophagus	K22.1	3251	463010	0.70%
ukb-d-K25	Diagnoses - main ICD10: K25 Gastric ulcer	K25	1834	361194	0.51%
ukb-b-12202	Diagnoses - secondary ICD10: K25.9 Unspecified as acute or chronic, without haemorrhage or perforation	K25.9	1355	463010	0.29%
ukb-d-K26	Diagnoses - main ICD10: K26 Duodenal ulcer	K26	1291	361194	0.36%
ukb-b-6616	Diagnoses - secondary ICD10: K26.9 Unspecified as acute or chronic, without haemorrhage or perforation	K26.9	1031	463010	0.22%
ukb-a-547	Diagnoses - main ICD10: K29 Gastritis and duodenitis	K29	8080	337199	2.40%
ukb-b-12191	Diagnoses - secondary ICD10: K29.5 Chronic gastritis, unspecified	K29.5	1062	463010	0.23%
ukb-b-1306	Diagnoses - main ICD10: K29.6 Other gastritis	K29.6	1830	463010	0.40%
ukb-b-15340	Diagnoses - secondary ICD10: K29.7 Gastritis, unspecified	K29.7	5718	463010	1.23%
ukb-b-12316	Diagnoses - main ICD10: K29.8 Duodenitis	K29.8	1800	463010	0.39%
ukb-b-12556	Diagnoses - secondary ICD10: K30 Dyspepsia	K30	2024	463010	0.44%
ukb-d-K31	Diagnoses - main ICD10: K31 Other diseases of stomach and duodenum	K31	2374	361194	0.66%
ukb-b-3027	Diagnoses - secondary ICD10: K31.7 Polyp of stomach and duodenum	K31.7	1233	463010	0.27%
ukb-b-2973	Diagnoses - secondary ICD10: K31.8 Other specified diseases of stomach and duodenum	K31.8	1247	463010	0.27%
ukb-a-548	Diagnoses - main ICD10: K35 Acute appendicitis	K35	1676	337199	0.50%
ukb-b-686	Diagnoses - main ICD10: K35.9 Acute appendicitis, unspecified	K35.9	1568	463010	0.34%
ukb-a-549	Diagnoses - main ICD10: K40 Inguinal hernia	K40	8815	337199	2.61%
ukb-b-11369	Diagnoses - main ICD10: K40.2 Bilateral inguinal hernia, without obstruction or gangrene	K40.2	1125	463010	0.24%
ukb-b-5369	Diagnoses - main ICD10: K40.9 Unilateral or unspecified inguinal hernia, without obstruction or gangrene	K40.9	11789	463010	2.55%
ukb-d-K42	Diagnoses - main ICD10: K42 Umbilical hernia	K42	2528	361194	0.70%
ukb-b-14541	Diagnoses - main ICD10: K42.9 Umbilical hernia without obstruction or gangrene	K42.9	2315	463010	0.50%
ukb-a-550	Diagnoses - main ICD10: K43 Ventral hernia	K43	1536	337199	0.46%
ukb-b-677	Diagnoses - main ICD10: K43.9 Ventral hernia without obstruction or gangrene	K43.9	2188	463010	0.47%
ukb-a-551	Diagnoses - main ICD10: K44 Diaphragmatic hernia	K44	5321	337199	1.58%
ukb-b-3672	Diagnoses - secondary ICD10: K44.9 Diaphragmatic hernia without obstruction or gangrene	K44.9	14326	463010	3.09%
ukb-a-552	Diagnoses - main ICD10: K50 Crohn's disease [regional enteritis]	K50	732	337199	0.22%
ukb-a-553	Diagnoses - main ICD10: K51 Ulcerative colitis	K51	1579	337199	0.47%
ukb-b-19386	Diagnoses - main ICD10: K51.9 Ulcerative colitis, unspecified	K51.9	1987	463010	0.43%
ukb-a-554	Diagnoses - main ICD10: K52 Other non-infective gastro-enteritis and colitis	K52	6453	337199	1.91%
ukb-b-20189	Diagnoses - main ICD10: K52.9 Non-infective gastro-enteritis and colitis, unspecified	K52.9	9463	463010	2.04%
ukb-d-K56	Diagnoses - main ICD10: K56 Paralytic ileus and intestinal obstruction without hernia	K56	1851	361194	0.51%
ukb-b-9797	Diagnoses - main ICD10: K56.6 Other and unspecified intestinal obstruction	K56.6	1006	463010	0.22%
ukb-a-555	Diagnoses - main ICD10: K57 Diverticular disease of intestine	K57	6865	337199	2.04%
ukb-b-10859	Diagnoses - main ICD10: K57.3 Diverticular disease of large intestine without perforation or abscess	K57.3	8429	463010	1.82%
ukb-b-14429	Diagnoses - secondary ICD10: K57.9 Diverticular disease of intestine, part unspecified, without perforation or abscess	K57.9	2622	463010	0.57%
ukb-d-K58	Diagnoses - main ICD10: K58 Irritable bowel syndrome	K58	1121	361194	0.31%
ukb-b-17961	Diagnoses - main ICD10: K58.9 Irritable bowel syndrome without diarrhoea	K58.9	1047	463010	0.23%
ukb-d-K59	Diagnoses - main ICD10: K59 Other functional intestinal disorders	K59	3712	361194	1.03%
ukb-b-18008	Diagnoses - secondary ICD10: K59.0 Constipation	K59.0	3862	463010	0.83%
ukb-a-556	Diagnoses - main ICD10: K60 Fissure and fistula of anal and rectal regions	K60	1578	337199	0.47%
ukb-b-11274	Diagnoses - main ICD10: K60.2 Anal fissure, unspecified	K60.2	1093	463010	0.24%
ukb-b-6721	Diagnoses - main ICD10: K60.3 Anal fistula	K60.3	1003	463010	0.22%
ukb-d-K61	Diagnoses - main ICD10: K61 Abscess of anal and rectal regions	K61	1053	361194	0.29%
ukb-b-5018	Diagnoses - main ICD10: K61.0 Anal abscess	K61.0	1034	463010	0.22%

ukb-a-557	Diagnoses - main ICD10: K62 Other diseases of anus and rectum	K62	9853	337199	2.92%
ukb-b-19805	Diagnoses - main ICD10: K62.1 Rectal polyp	K62.1	2800	463010	0.60%
ukb-b-11582	Diagnoses - main ICD10: K62.5 Haemorrhage of anus and rectum	K62.5	9579	463010	2.07%
ukb-b-13464	Diagnoses - main ICD10: K62.8 Other specified diseases of anus and rectum	K62.8	1406	463010	0.30%
ukb-d-K63	Diagnoses - main ICD10: K63 Other diseases of intestine	K63	8041	361194	2.23%
ukb-b-17845	Diagnoses - secondary ICD10: K63.5 Polyp of colon	K63.5	2437	463010	0.53%
ukb-b-11675	Diagnoses - secondary ICD10: K66.0 Peritoneal adhesions	K66.0	1457	463010	0.31%
ukb-a-558	Diagnoses - main ICD10: K76 Other diseases of liver	K76	356	337199	0.11%
ukb-a-559	Diagnoses - main ICD10: K80 Cholelithiasis	K80	6986	337199	2.07%
ukb-b-10362	Diagnoses - main ICD10: K80.0 Calculus of gallbladder with acute cholecystitis	K80.0	1100	463010	0.24%
ukb-b-8988	Diagnoses - main ICD10: K80.1 Calculus of gallbladder with other cholecystitis	K80.1	3932	463010	0.85%
ukb-b-11020	Diagnoses - main ICD10: K80.2 Calculus of gallbladder without cholecystitis	K80.2	5766	463010	1.25%
ukb-b-8268	Diagnoses - main ICD10: K80.5 Calculus of bile duct without cholangitis or cholecystitis	K80.5	1706	463010	0.37%
ukb-d-K81	Diagnoses - main ICD10: K81 Cholecystitis	K81	1930	361194	0.53%
ukb-b-19388	Diagnoses - main ICD10: K85 Acute pancreatitis	K85	1215	463010	0.26%
ukb-d-K92	Diagnoses - main ICD10: K92 Other diseases of digestive system	K92	5019	361194	1.39%
ukb-b-1853	Diagnoses - main ICD10: K92.0 Haematemesis	K92.0	1065	463010	0.23%
ukb-b-5590	Diagnoses - main ICD10: K92.1 Melaena	K92.1	1318	463010	0.28%
ukb-b-14999	Diagnoses - main ICD10: K92.2 Gastro-intestinal haemorrhage, unspecified	K92.2	1449	463010	0.31%
ukb-d-L02	Diagnoses - main ICD10: L02 Cutaneous abscess, furuncle and carbuncle	L02	1697	361194	0.47%
ukb-a-560	Diagnoses - main ICD10: L03 Cellulitis	L03	2777	337199	0.82%
ukb-b-11908	Diagnoses - main ICD10: L03.1 Cellulitis of other parts of limb	L03.1	3360	463010	0.73%
ukb-d-L57	Diagnoses - main ICD10: L57 Skin changes due to chronic exposure to nonionising radiation	L57	1447	361194	0.40%
ukb-d-L72	Diagnoses - main ICD10: L72 Follicular cysts of skin and subcutaneous tissue	L72	6644	361194	1.84%
ukb-b-15003	Diagnoses - main ICD10: L72.0 Epidermal cyst	L72.0	1863	463010	0.40%
ukb-b-14436	Diagnoses - main ICD10: L72.9 Follicular cyst of skin and subcutaneous tissue, unspecified	L72.9	1001	463010	0.22%
ukb-b-501	Diagnoses - main ICD10: L82 Seborrhoeic keratosis	L82	1957	463010	0.42%
ukb-d-L90	Diagnoses - main ICD10: L90 Atrophic disorders of skin	L90	1683	361194	0.47%
ukb-b-11403	Diagnoses - main ICD10: L90.5 Scar conditions and fibrosis of skin	L90.5	1464	463010	0.32%
ukb-d-L98	Diagnoses - main ICD10: L98 Other disorders of skin and subcutaneous tissue, not elsewhere classified	L98	4035	361194	1.12%
ukb-b-20190	Diagnoses - main ICD10: L98.9 Disorder of skin and subcutaneous tissue, unspecified	L98.9	3223	463010	0.70%
ukb-d-M06	Diagnoses - main ICD10: M06 Other rheumatoid arthritis	M06	1401	361194	0.39%
ukb-b-11874	Diagnoses - secondary ICD10: M06.99 Rheumatoid arthritis, unspecified (Site unspecified)	M06.99	1523	463010	0.33%
ukb-a-561	Diagnoses - main ICD10: M10 Gout	M10	181	337199	0.05%
ukb-b-12765	Diagnoses - secondary ICD10: M10.99 Gout, unspecified (Site unspecified)	M10.99	1042	463010	0.23%
ukb-d-M13	Diagnoses - main ICD10: M13 Other arthritis	M13	1110	361194	0.31%
ukb-b-11045	Diagnoses - secondary ICD10: M13.9 Arthritis, unspecified	M13.9	1477	463010	0.32%
ukb-b-15026	Diagnoses - secondary ICD10: M13.99 Arthritis, unspecified (Site unspecified)	M13.99	2785	463010	0.60%
ukb-d-M15	Diagnoses - main ICD10: M15 Polyarthrosis	M15	1264	361194	0.35%
ukb-b-6998	Diagnoses - secondary ICD10: M15.9 Polyarthrosis, unspecified	M15.9	1165	463010	0.25%
ukb-a-562	Diagnoses - main ICD10: M16 Coxarthrosis [arthrosis of hip]	M16	4934	337199	1.46%
ukb-b-2875	Diagnoses - main ICD10: M16.1 Other primary coxarthrosis	M16.1	2876	463010	0.62%
ukb-b-19829	Diagnoses - main ICD10: M16.9 Coxarthrosis, unspecified	M16.9	4255	463010	0.92%
ukb-a-563	Diagnoses - main ICD10: M17 Gonarthrosis [arthrosis of knee]	M17	6560	337199	1.95%
ukb-b-12964	Diagnoses - secondary ICD10: M17.1 Other primary gonarthrosis	M17.1	1492	463010	0.32%
ukb-b-18379	Diagnoses - main ICD10: M17.9 Gonarthrosis, unspecified	M17.9	6497	463010	1.40%
ukb-d-M19	Diagnoses - main ICD10: M19 Other arthrosis	M19	4165	361194	1.15%
ukb-b-9493	Diagnoses - secondary ICD10: M19.9 Arthrosis, unspecified	M19.9	1797	463010	0.39%
ukb-b-20233	Diagnoses - secondary ICD10: M19.99 Arthrosis, unspecified (Site unspecified)	M19.99	2905	463010	0.63%
ukb-a-564	Diagnoses - main ICD10: M20 Acquired deformities of fingers and toes	M20	5148	337199	1.53%
ukb-b-6026	Diagnoses - main ICD10: M20.1 Hallux valgus (acquired)	M20.1	5304	463010	1.15%
ukb-b-4280	Diagnoses - main ICD10: M20.2 Hallux rigidus	M20.2	1083	463010	0.23%
ukb-a-565	Diagnoses - main ICD10: M21 Other acquired deformities of limbs	M21	379	337199	0.11%

ukb-a-566	Diagnoses - main ICD10: M23 Internal derangement of knee	M23	7489	337199	2.22%
ukb-b-8213	Diagnoses - main ICD10: M23.2 Derangement of meniscus due to old tear or injury	M23.2	1433	463010	0.31%
ukb-b-9694	Diagnoses - main ICD10: M23.22 Derangement of meniscus due to old tear or injury (Posterior cruciate ligament or Poste	M23.22	1958	463010	0.42%
ukb-b-7118	Diagnoses - main ICD10: M23.23 Derangement of meniscus due to old tear or injury (Medial collateral ligament or Other	M23.23	2596	463010	0.56%
ukb-a-567	Diagnoses - main ICD10: M24 Other specific joint derangements	M24	838	337199	0.25%
ukb-a-568	Diagnoses - main ICD10: M25 Other joint disorders not elsewhere classified	M25	4908	337199	1.46%
ukb-b-13019	Diagnoses - main ICD10: M25.5 Pain in joint	M25.5	1451	463010	0.31%
ukb-b-2083	Diagnoses - main ICD10: M25.56 Pain in joint (Lower leg)	M25.56	1624	463010	0.35%
ukb-d-M47	Diagnoses - main ICD10: M47 Spondylosis	M47	2004	361194	0.55%
ukb-b-2625	Diagnoses - secondary ICD10: M47.82 Other spondylosis (Cervical region)	M47.82	1018	463010	0.22%
ukb-d-M48	Diagnoses - main ICD10: M48 Other spondylopathies	M48	1890	361194	0.52%
ukb-d-M51	Diagnoses - main ICD10: M51 Other intervertebral disk disorders	M51	4690	361194	1.30%
ukb-b-18279	Diagnoses - main ICD10: M51.1 Lumbar and other intervertebral disk disorders with radiculopathy	M51.1	2214	463010	0.48%
ukb-b-12872	Diagnoses - main ICD10: M51.2 Other specified intervertebral disk displacement	M51.2	1433	463010	0.31%
ukb-b-19807	Diagnoses - main ICD10: M51.3 Other specified intervertebral disk degeneration	M51.3	1045	463010	0.23%
ukb-a-569	Diagnoses - main ICD10: M54 Dorsalgia	M54	5660	337199	1.68%
ukb-b-1557	Diagnoses - main ICD10: M54.5 Low back pain	M54.5	2439	463010	0.53%
ukb-b-19813	Diagnoses - main ICD10: M54.56 Low back pain (Lumbar region)	M54.56	1423	463010	0.31%
ukb-b-10873	Diagnoses - main ICD10: M54.59 Low back pain (Site unspecified)	M54.59	1446	463010	0.31%
ukb-d-M65	Diagnoses - main ICD10: M65 Synovitis and tenosynovitis	M65	2812	361194	0.78%
ukb-b-8085	Diagnoses - main ICD10: M65.3 Trigger finger	M65.3	1059	463010	0.23%
ukb-a-570	Diagnoses - main ICD10: M67 Other disorders of synovium and tendon	M67	1787	337199	0.53%
ukb-b-12364	Diagnoses - main ICD10: M67.4 Ganglion	M67.4	2172	463010	0.47%
ukb-a-571	Diagnoses - main ICD10: M70 Soft tissue disorders related to use overuse and pressure	M70	673	337199	0.20%
ukb-a-572	Diagnoses - main ICD10: M72 Fibroblastic disorders	M72	1862	337199	0.55%
ukb-b-13517	Diagnoses - main ICD10: M72.0 Palmar fascial fibromatosis [Dupuytren]	M72.0	1366	463010	0.30%
ukb-b-4772	Diagnoses - main ICD10: M72.04 Palmar fascial fibromatosis [Dupuytren]-Hand	M72.04	1537	463010	0.33%
ukb-d-M75	Diagnoses - main ICD10: M75 Shoulder lesions	M75	7040	361194	1.95%
ukb-b-4921	Diagnoses - main ICD10: M75.0 Adhesive capsulitis of shoulder	M75.0	1083	463010	0.23%
ukb-b-50	Diagnoses - main ICD10: M75.1 Rotator cuff syndrome	M75.1	1767	463010	0.38%
ukb-b-1418	Diagnoses - main ICD10: M75.4 Impingement syndrome of shoulder	M75.4	2901	463010	0.63%
ukb-d-M79	Diagnoses - main ICD10: M79 Other soft tissue disorders, not elsewhere classified	M79	6946	361194	1.92%
ukb-b-9708	Diagnoses - main ICD10: M79.66 Pain in limb (Lower leg)	M79.66	1689	463010	0.36%
ukb-b-12620	Diagnoses - main ICD10: M79.86 Other specified soft tissue disorders (Lower leg)	M79.86	1592	463010	0.34%
ukb-b-17796	Diagnoses - secondary ICD10: M81.99 Osteoporosis, unspecified (Site unspecified)	M81.99	1976	463010	0.43%
ukb-d-M84	Diagnoses - main ICD10: M84 Disorders of continuity of bone	M84	1065	361194	0.29%
ukb-d-N13	Diagnoses - main ICD10: N13 Obstructive and reflux uropathy	N13	1359	361194	0.38%
ukb-b-4963	Diagnoses - secondary ICD10: N17.9 Acute renal failure, unspecified	N17.9	1415	463010	0.31%
ukb-a-573	Diagnoses - main ICD10: N19 Unspecified renal failure	N19	126	337199	0.04%
ukb-a-574	Diagnoses - main ICD10: N20 Calculus of kidney and ureter	N20	2427	337199	0.72%
ukb-b-18372	Diagnoses - main ICD10: N20.0 Calculus of kidney	N20.0	2186	463010	0.47%
ukb-b-18629	Diagnoses - main ICD10: N20.1 Calculus of ureter	N20.1	1979	463010	0.43%
ukb-b-16313	Diagnoses - main ICD10: N23 Unspecified renal colic	N23	1675	463010	0.36%
ukb-d-N30	Diagnoses - main ICD10: N30 Cystitis	N30	1575	361194	0.44%
ukb-d-N31	Diagnoses - main ICD10: N31 Neuromuscular dysfunction of bladder, not elsewhere classified	N31	1014	361194	0.28%
ukb-a-575	Diagnoses - main ICD10: N32 Other disorders of bladder	N32	2749	337199	0.82%
ukb-b-123	Diagnoses - main ICD10: N32.0 Bladder-neck obstruction	N32.0	1385	463010	0.30%
ukb-b-373	Diagnoses - main ICD10: N32.8 Other specified disorders of bladder	N32.8	2225	463010	0.48%
ukb-d-N35	Diagnoses - main ICD10: N35 Urethral stricture	N35	2037	361194	0.56%
ukb-b-9074	Diagnoses - main ICD10: N35.9 Urethral stricture, unspecified	N35.9	2013	463010	0.43%
ukb-d-N39	Diagnoses - main ICD10: N39 Other disorders of urinary system	N39	10551	361194	2.92%
ukb-b-6077	Diagnoses - secondary ICD10: N39.0 Urinary tract infection, site not specified	N39.0	4180	463010	0.90%
ukb-b-5266	Diagnoses - secondary ICD10: N39.3 Stress incontinence	N39.3	1404	463010	0.30%

ukb-a-576	Diagnoses - main ICD10: N40 Hyperplasia of prostate	N40	3140	337199	0.93%
ukb-b-10668	Diagnoses - main ICD10: N47 Redundant prepuce, phimosis and paraphimosis	N47	1541	463010	0.33%
ukb-d-N48	Diagnoses - main ICD10: N48 Other disorders of penis	N48	1317	361194	0.36%
ukb-d-N50	Diagnoses - main ICD10: N50 Other disorders of male genital organs	N50	1829	361194	0.51%
ukb-b-3802	Diagnoses - main ICD10: N50.8 Other specified disorders of male genital organs	N50.8	1408	463010	0.30%
ukb-d-N60	Diagnoses - main ICD10: N60 Benign mammary dysplasia	N60	1157	361194	0.32%
ukb-b-7428	Diagnoses - main ICD10: N63 Unspecified lump in breast	N63	1481	463010	0.32%
ukb-b-6413	Diagnoses - secondary ICD10: N73.6 Female pelvic peritoneal adhesions	N73.6	1790	463010	0.39%
ukb-d-N80	Diagnoses - main ICD10: N80 Endometriosis	N80	1496	361194	0.41%
ukb-b-9668	Diagnoses - secondary ICD10: N80.0 Endometriosis of uterus	N80.0	1121	463010	0.24%
ukb-a-577	Diagnoses - main ICD10: N81 Female genital prolapse	N81	4840	337199	1.44%
ukb-b-11298	Diagnoses - main ICD10: N81.1 Cystocele	N81.1	2554	463010	0.55%
ukb-b-2055	Diagnoses - secondary ICD10: N81.2 Incomplete uterovaginal prolapse	N81.2	1042	463010	0.23%
ukb-b-3412	Diagnoses - secondary ICD10: N81.6 Rectocele	N81.6	2093	463010	0.45%
ukb-d-N83	Diagnoses - main ICD10: N83 Noninflammatory disorders of ovary, Fallopian tube and broad ligament	N83	2215	361194	0.61%
ukb-b-4790	Diagnoses - secondary ICD10: N83.2 Other and unspecified ovarian cysts	N83.2	1656	463010	0.36%
ukb-d-N84	Diagnoses - main ICD10: N84 Polyp of female genital tract	N84	6986	361194	1.93%
ukb-b-12944	Diagnoses - main ICD10: N84.0 Polyp of corpus uteri	N84.0	5419	463010	1.17%
ukb-b-19110	Diagnoses - main ICD10: N84.1 Polyp of cervix uteri	N84.1	2307	463010	0.50%
ukb-d-N85	Diagnoses - main ICD10: N85 Other noninflammatory disorders of uterus, except cervix	N85	1489	361194	0.41%
ukb-b-8685	Diagnoses - secondary ICD10: N85.8 Other specified noninflammatory disorders of uterus	N85.8	1450	463010	0.31%
ukb-d-N87	Diagnoses - main ICD10: N87 Dysplasia of cervix uteri	N87	1102	361194	0.31%
ukb-d-N90	Diagnoses - main ICD10: N90 Other noninflammatory disorders of vulva and perineum	N90	1077	361194	0.30%
ukb-a-578	Diagnoses - main ICD10: N92 Excessive frequent and irregular menstruation	N92	6882	337199	2.04%
ukb-b-11572	Diagnoses - main ICD10: N92.0 Excessive and frequent menstruation with regular cycle	N92.0	6641	463010	1.43%
ukb-b-9591	Diagnoses - main ICD10: N92.1 Excessive and frequent menstruation with irregular cycle	N92.1	2048	463010	0.44%
ukb-b-14674	Diagnoses - main ICD10: N92.6 Irregular menstruation, unspecified	N92.6	1279	463010	0.28%
ukb-d-N93	Diagnoses - main ICD10: N93 Other abnormal uterine and vaginal bleeding	N93	2455	361194	0.68%
ukb-b-14086	Diagnoses - main ICD10: N93.8 Other specified abnormal uterine and vaginal bleeding	N93.8	1392	463010	0.30%
ukb-b-10849	Diagnoses - main ICD10: N93.9 Abnormal uterine and vaginal bleeding, unspecified	N93.9	1145	463010	0.25%
ukb-d-N94	Diagnoses - main ICD10: N94 Pain and other conditions associated with female genital organs and menstrual cycle	N94	1295	361194	0.36%
ukb-b-12604	Diagnoses - secondary ICD10: N94.6 Dysmenorrhoea, unspecified	N94.6	1145	463010	0.25%
ukb-d-N95	Diagnoses - main ICD10: N95 Menopausal and other perimenopausal disorders	N95	6496	361194	1.80%
ukb-b-16879	Diagnoses - secondary ICD10: N95.0 Postmenopausal bleeding	N95.0	2161	463010	0.47%
ukb-d-O02	Diagnoses - main ICD10: O02 Other abnormal products of conception	O02	1106	361194	0.31%
ukb-b-13414	Diagnoses - main ICD10: O02.1 Missed abortion	O02.1	1317	463010	0.28%
ukb-d-O03	Diagnoses - main ICD10: O03 Spontaneous abortion	O03	1150	361194	0.32%
ukb-b-13696	Diagnoses - main ICD10: O04.9 Complete or unspecified, without complication	O04.9	1068	463010	0.23%
ukb-d-O26	Diagnoses - main ICD10: O26 Maternal care for other conditions predominantly related to pregnancy	O26	1289	361194	0.36%
ukb-b-9712	Diagnoses - main ICD10: O26.8 Other specified pregnancy-related conditions	O26.8	1302	463010	0.28%
ukb-d-O36	Diagnoses - main ICD10: O36 Maternal care for other known or suspected foetal problems	O36	1149	361194	0.32%
ukb-d-O63	Diagnoses - main ICD10: O63 Long labour	O63	1060	361194	0.29%
ukb-b-2194	Diagnoses - main ICD10: O63.1 Prolonged second stage (of labour)	O63.1	1027	463010	0.22%
ukb-d-O68	Diagnoses - main ICD10: O68 Labour and delivery complicated by foetal stress [distress]	O68	1882	361194	0.52%
ukb-b-9270	Diagnoses - main ICD10: O68.0 Labour and delivery complicated by foetal heart rate anomaly	O68.0	1009	463010	0.22%
ukb-d-O70	Diagnoses - main ICD10: O70 Perineal laceration during delivery	O70	3077	361194	0.85%
ukb-b-9597	Diagnoses - main ICD10: O70.0 First degree perineal laceration during delivery	O70.0	1620	463010	0.35%
ukb-b-10712	Diagnoses - main ICD10: O70.1 Second degree perineal laceration during delivery	O70.1	2469	463010	0.53%
ukb-a-579	Diagnoses - main ICD10: O75 Other complications of labour and delivery not elsewhere classified	O75	181	337199	0.05%
ukb-d-O80	Diagnoses - main ICD10: O80 Single spontaneous delivery	O80	1672	361194	0.46%
ukb-b-6330	Diagnoses - main ICD10: O80.0 Spontaneous vertex delivery	O80.0	1726	463010	0.37%
ukb-d-R00	Diagnoses - main ICD10: R00 Abnormalities of heart beat	R00	2542	361194	0.70%
ukb-b-17309	Diagnoses - secondary ICD10: R00.0 Tachycardia, unspecified	R00.0	1005	463010	0.22%

ukb-b-11664	Diagnoses - secondary ICD10: R00.1 Bradycardia, unspecified	R00.1	1254	463010	0.27%
ukb-b-1480	Diagnoses - secondary ICD10: R00.2 Palpitations	R00.2	1380	463010	0.30%
ukb-b-16587	Diagnoses - secondary ICD10: R03.0 Elevated blood-pressure reading, without diagnosis of hypertension	R03.0	1134	463010	0.24%
ukb-a-580	Diagnoses - main ICD10: R04 Haemorrhage from respiratory passages	R04	1788	337199	0.53%
ukb-b-11412	Diagnoses - main ICD10: R04.0 Epistaxis	R04.0	1512	463010	0.33%
ukb-b-4507	Diagnoses - main ICD10: R04.2 Haemoptysis	R04.2	1240	463010	0.27%
ukb-b-20009	Diagnoses - secondary ICD10: R05 Cough	R05	1072	463010	0.23%
ukb-d-R06	Diagnoses - main ICD10: R06 Abnormalities of breathing	R06	4126	361194	1.14%
ukb-b-19391	Diagnoses - secondary ICD10: R06.0 Dyspnoea	R06.0	2248	463010	0.49%
ukb-b-19769	Diagnoses - main ICD10: R06.5 Mouth breathing	R06.5	1536	463010	0.33%
ukb-a-581	Diagnoses - main ICD10: R07 Pain in throat and chest	R07	16552	337199	4.91%
ukb-b-20386	Diagnoses - main ICD10: R07.2 Precordial pain	R07.2	2140	463010	0.46%
ukb-b-14603	Diagnoses - main ICD10: R07.3 Other chest pain	R07.3	7146	463010	1.54%
ukb-b-10771	Diagnoses - main ICD10: R07.4 Chest pain, unspecified	R07.4	18203	463010	3.93%
ukb-a-582	Diagnoses - main ICD10: R10 Abdominal and pelvic pain	R10	14203	337199	4.21%
ukb-b-10409	Diagnoses - secondary ICD10: R10.1 Pain localised to upper abdomen	R10.1	1543	463010	0.33%
ukb-b-17456	Diagnoses - main ICD10: R10.3 Pain localised to other parts of lower abdomen	R10.3	4823	463010	1.04%
ukb-b-19409	Diagnoses - secondary ICD10: R10.4 Other and unspecified abdominal pain	R10.4	4649	463010	1.00%
ukb-a-583	Diagnoses - main ICD10: R11 Nausea and vomiting	R11	1365	337199	0.40%
ukb-b-7073	Diagnoses - main ICD10: R13 Dysphagia	R13	3012	463010	0.65%
ukb-a-584	Diagnoses - main ICD10: R14 Flatulence and related conditions	R14	201	337199	0.06%
ukb-d-R19	Diagnoses - main ICD10: R19 Other symptoms and signs involving the digestive system and abdomen	R19	8796	361194	2.44%
ukb-b-10368	Diagnoses - secondary ICD10: R19.4 Change in bowel habit	R19.4	2443	463010	0.53%
ukb-d-R22	Diagnoses - main ICD10: R22 Localised swelling, mass and lump of skin and subcutaneous tissue	R22	1358	361194	0.38%
ukb-a-585	Diagnoses - main ICD10: R31 Unspecified haematuria	R31	7363	337199	2.18%
ukb-b-11531	Diagnoses - main ICD10: R32 Unspecified urinary incontinence	R32	1011	463010	0.22%
ukb-b-19207	Diagnoses - secondary ICD10: R33 Retention of urine	R33	2599	463010	0.56%
ukb-a-586	Diagnoses - main ICD10: R35 Polyuria	R35	1349	337199	0.40%
ukb-d-R39	Diagnoses - main ICD10: R39 Other symptoms and signs involving the urinary system	R39	2698	361194	0.75%
ukb-b-17287	Diagnoses - main ICD10: R39.8 Other and unspecified symptoms and signs involving the urinary system	R39.8	1943	463010	0.42%
ukb-b-20542	Diagnoses - secondary ICD10: R42 Dizziness and giddiness	R42	1756	463010	0.38%
ukb-d-R50	Diagnoses - main ICD10: R50 Fever of unknown origin	R50	1156	361194	0.32%
ukb-b-19086	Diagnoses - secondary ICD10: R50.9 Fever, unspecified	R50.9	1794	463010	0.39%
ukb-b-1977	Diagnoses - main ICD10: R51 Headache	R51	4293	463010	0.93%
ukb-b-11972	Diagnoses - secondary ICD10: R53 Malaise and fatigue	R53	1245	463010	0.27%
ukb-a-587	Diagnoses - main ICD10: R55 Syncope and collapse	R55	3057	337199	0.91%
ukb-d-R56	Diagnoses - main ICD10: R56 Convulsions, not elsewhere classified	R56	1077	361194	0.30%
ukb-d-R59	Diagnoses - main ICD10: R59 Enlarged lymph nodes	R59	1011	361194	0.28%
ukb-d-R63	Diagnoses - main ICD10: R63 Symptoms and signs concerning food and fluid intake	R63	1279	361194	0.35%
ukb-b-13845	Diagnoses - secondary ICD10: R63.4 Abnormal weight loss	R63.4	2751	463010	0.59%
ukb-a-588	Diagnoses - main ICD10: R69 Unknown and unspecified causes of morbidity	R69	6717	337199	1.99%
ukb-d-R79	Diagnoses - main ICD10: R79 Other abnormal findings of blood chemistry	R79	2622	361194	0.73%
ukb-b-11052	Diagnoses - main ICD10: R79.8 Other specified abnormal findings of blood chemistry	R79.8	1972	463010	0.43%
ukb-d-R87	Diagnoses - main ICD10: R87 Abnormal findings in specimens from female genital organs	R87	1084	361194	0.30%
ukb-b-15836	Diagnoses - main ICD10: R87.6 Abnormal cytological findings	R87.6	1283	463010	0.28%
ukb-d-R93	Diagnoses - main ICD10: R93 Abnormal findings on diagnostic imaging of other body structures	R93	1217	361194	0.34%
ukb-b-4579	Diagnoses - secondary ICD10: R94.5 Abnormal results of liver function studies	R94.5	1802	463010	0.39%
ukb-d-S01	Diagnoses - main ICD10: S01 Open wound of head	S01	1694	361194	0.47%
ukb-d-S02	Diagnoses - main ICD10: S02 Fracture of skull and facial bones	S02	1351	361194	0.37%
ukb-a-589	Diagnoses - main ICD10: S09 Other and unspecified injuries of head	S09	932	337199	0.28%
ukb-b-16917	Diagnoses - main ICD10: S09.9 Unspecified injury of head	S09.9	1394	463010	0.30%
ukb-d-S42	Diagnoses - main ICD10: S42 Fracture of shoulder and upper arm	S42	1791	361194	0.50%
ukb-a-590	Diagnoses - main ICD10: S52 Fracture of forearm	S52	3284	337199	0.97%

ukb-b-2804	Diagnoses - main ICD10: S52.50 Fracture of lower end of radius (closed)	S52.50	2696	463010	0.58%
ukb-d-S61	Diagnoses - main ICD10: S61 Open wound of wrist and hand	S61	1865	361194	0.52%
ukb-d-S62	Diagnoses - main ICD10: S62 Fracture at wrist and hand level	S62	1763	361194	0.49%
ukb-a-591	Diagnoses - main ICD10: S66 Injury of muscle and tendon at wrist and hand level	S66	553	337199	0.16%
ukb-d-S72	Diagnoses - main ICD10: S72 Fracture of femur	S72	1803	361194	0.50%
ukb-a-592	Diagnoses - main ICD10: S76 Injury of muscle and tendon at hip and thigh level	S76	154	337199	0.05%
ukb-d-S82	Diagnoses - main ICD10: S82 Fracture of lower leg, including ankle	S82	4557	361194	1.26%
ukb-b-5946	Diagnoses - main ICD10: S82.80 Fractures of other parts of lower leg (closed)	S82.80	1479	463010	0.32%
ukb-d-T39	Diagnoses - main ICD10: T39 Poisoning by nonopioid analgesics, antipyretics and antirheumatics	T39	1161	361194	0.32%
ukb-b-12187	Diagnoses - main ICD10: T39.1 4-Aminophenol derivatives	T39.1	1014	463010	0.22%
ukb-d-T81	Diagnoses - main ICD10: T81 Complications of procedures, not elsewhere classified	T81	5550	361194	1.54%
ukb-b-17587	Diagnoses - main ICD10: T81.0 Haemorrhage and haematoma complicating a procedure, not elsewhere classified	T81.0	1896	463010	0.41%
ukb-b-4586	Diagnoses - secondary ICD10: T81.4 Infection following a procedure, not elsewhere classified	T81.4	1506	463010	0.33%
ukb-d-T82	Diagnoses - main ICD10: T82 Complications of cardiac and vascular prosthetic devices, implants and grafts	T82	1133	361194	0.31%
ukb-a-593	Diagnoses - main ICD10: T84 Complications of internal orthopaedic prosthetic devices implants and grafts	T84	2053	337199	0.61%
ukb-b-14753	Diagnoses - main ICD10: T84.0 Mechanical complication of internal joint prosthesis	T84.0	1178	463010	0.25%
ukb-b-11680	Diagnoses - main ICD10: T84.8 Other complications of internal orthopaedic prosthetic devices, implants and grafts	T84.8	1027	463010	0.22%
ukb-d-T85	Diagnoses - main ICD10: T85 Complications of other internal prosthetic devices, implants and grafts	T85	1501	361194	0.42%
ukb-b-11065	Diagnoses - secondary ICD10: W01.0 Home	W01.0	1122	463010	0.24%
ukb-b-12058	Diagnoses - secondary ICD10: W01.9 Unspecified place	W01.9	1345	463010	0.29%
ukb-b-19088	Diagnoses - secondary ICD10: W10.0 Home	W10.0	1120	463010	0.24%
ukb-b-2854	Diagnoses - secondary ICD10: W19.9 Unspecified place	W19.9	2506	463010	0.54%
ukb-b-14257	Diagnoses - secondary ICD10: X59.9 Unspecified place	X59.9	3232	463010	0.70%
ukb-b-4745	Diagnoses - secondary ICD10: Y43.3 Other antineoplastic drugs	Y43.3	1101	463010	0.24%
ukb-b-13841	Diagnoses - secondary ICD10: Y83.1 Surgical operation with implant of artificial internal device	Y83.1	4135	463010	0.89%
ukb-b-3115	Diagnoses - secondary ICD10: Y83.2 Surgical operation with anastomosis, bypass or graft	Y83.2	1411	463010	0.30%
ukb-b-7702	Diagnoses - secondary ICD10: Y83.6 Removal of other organ (partial) (total)	Y83.6	3054	463010	0.66%
ukb-b-17823	Diagnoses - secondary ICD10: Y83.8 Other surgical procedures	Y83.8	4789	463010	1.03%
ukb-d-Z01	Diagnoses - main ICD10: Z01 Other special examinations and investigations of persons without complaint or reported dia	Z01	1370	361194	0.38%
ukb-d-Z03	Diagnoses - main ICD10: Z03 Medical observation and evaluation for suspected diseases and conditions	Z03	4951	361194	1.37%
ukb-b-6295	Diagnoses - secondary ICD10: Z03.4 Observation for suspected myocardial infarction	Z03.4	2559	463010	0.55%
ukb-b-10490	Diagnoses - secondary ICD10: Z03.8 Observation for other suspected diseases and conditions	Z03.8	2219	463010	0.48%
ukb-d-Z08	Diagnoses - main ICD10: Z08 Follow-up examination after treatment for malignant neoplasm	Z08	4214	361194	1.17%
ukb-b-1261	Diagnoses - main ICD10: Z08.0 Follow-up examination after surgery for malignant neoplasm	Z08.0	3005	463010	0.65%
ukb-a-594	Diagnoses - main ICD10: Z09 Follow-up examination after treatment for conditions other than malignant neoplasms	Z09	5162	337199	1.53%
ukb-b-10102	Diagnoses - main ICD10: Z09.0 Follow-up examination after surgery for other conditions	Z09.0	3424	463010	0.74%
ukb-b-14479	Diagnoses - main ICD10: Z09.8 Follow-up examination after other treatment for other conditions	Z09.8	2243	463010	0.48%
ukb-b-10646	Diagnoses - main ICD10: Z09.9 Follow-up examination after unspecified treatment for other conditions	Z09.9	1942	463010	0.42%
ukb-d-Z12	Diagnoses - main ICD10: Z12 Special screening examination for neoplasms	Z12	4152	361194	1.15%
ukb-b-203	Diagnoses - main ICD10: Z12.1 Special screening examination for neoplasm of intestinal tract	Z12.1	3103	463010	0.67%
ukb-d-Z13	Diagnoses - main ICD10: Z13 Special screening examination for other diseases and disorders	Z13	2236	361194	0.62%
ukb-b-5443	Diagnoses - secondary ICD10: Z13.0 Special screening examination for diseases of the blood and blood-forming organs a	Z13.0	1060	463010	0.23%
ukb-b-20038	Diagnoses - main ICD10: Z13.8 Special screening examination for other specified diseases and disorders	Z13.8	1246	463010	0.27%
ukb-d-Z30	Diagnoses - main ICD10: Z30 Contraceptive management	Z30	6392	361194	1.77%
ukb-b-7418	Diagnoses - main ICD10: Z30.2 Sterilisation	Z30.2	7023	463010	1.52%
ukb-b-4193	Diagnoses - secondary ICD10: Z37.0 Single live birth	Z37.0	12567	463010	2.71%
ukb-d-Z42	Diagnoses - main ICD10: Z42 Follow-up care involving plastic surgery	Z42	1963	361194	0.54%
ukb-b-18897	Diagnoses - main ICD10: Z42.1 Follow-up care involving plastic surgery of breast	Z42.1	1522	463010	0.33%
ukb-d-Z43	Diagnoses - main ICD10: Z43 Attention to artificial openings	Z43	1356	361194	0.38%
ukb-d-Z45	Diagnoses - main ICD10: Z45 Adjustment and management of implanted device	Z45	2209	361194	0.61%
ukb-d-Z46	Diagnoses - main ICD10: Z46 Fitting and adjustment of other devices	Z46	3614	361194	1.00%
ukb-b-7463	Diagnoses - main ICD10: Z46.6 Fitting and adjustment of urinary device	Z46.6	2729	463010	0.59%
ukb-a-595	Diagnoses - main ICD10: Z47 Other orthopaedic follow-up care	Z47	1954	337199	0.58%

ukb-b-19542	Diagnoses - main ICD10: Z47.0 Follow-up care involving removal of fracture plate and other internal fixation device	Z47.0	2730	463010	0.59%
ukb-b-10061	Diagnoses - secondary ICD10: Z50.1 Other physical therapy	Z50.1	1824	463010	0.39%
ukb-b-16024	Diagnoses - secondary ICD10: Z51.0 Radiotherapy session	Z51.0	1942	463010	0.42%
ukb-b-11484	Diagnoses - secondary ICD10: Z51.1 Chemotherapy session for neoplasm	Z51.1	7714	463010	1.67%
ukb-b-712	Diagnoses - secondary ICD10: Z51.2 Other chemotherapy	Z51.2	3035	463010	0.66%
ukb-b-15983	Diagnoses - secondary ICD10: Z51.5 Palliative care	Z51.5	1231	463010	0.27%
ukb-d-Z53	Diagnoses - main ICD10: Z53 Persons encountering health services for specifie procedures, not carried out	Z53	1754	361194	0.49%
ukb-b-9019	Diagnoses - secondary ICD10: Z53.0 Procedure not carried out because of contraindication	Z53.0	4779	463010	1.03%
ukb-b-5045	Diagnoses - secondary ICD10: Z53.2 Procedure not carried out because of patient's decision for other and unspecified rea	Z53.2	1283	463010	0.28%
ukb-b-9043	Diagnoses - secondary ICD10: Z53.8 Procedure not carried out for other reasons	Z53.8	10952	463010	2.37%
ukb-b-9216	Diagnoses - secondary ICD10: Z53.9 Procedure not carried out, unspecified reason	Z53.9	1877	463010	0.41%
ukb-b-2895	Diagnoses - secondary ICD10: Z60.2 Living alone	Z60.2	3497	463010	0.76%
ukb-b-5115	Diagnoses - secondary ICD10: Z72.0 Tobacco use	Z72.0	13421	463010	2.90%
ukb-b-391	Diagnoses - secondary ICD10: Z72.1 Alcohol use	Z72.1	3685	463010	0.80%
ukb-a-596	Diagnoses - main ICD10: Z80 Family history of malignant neoplasm	Z80	326	337199	0.10%
ukb-b-13247	Diagnoses - secondary ICD10: Z80.0 Family history of malignant neoplasm of digestive organs	Z80.0	5989	463010	1.29%
ukb-b-7643	Diagnoses - secondary ICD10: Z80.3 Family history of malignant neoplasm of breast	Z80.3	1091	463010	0.24%
ukb-b-2205	Diagnoses - secondary ICD10: Z82.4 Family history of ischaemic heart disease and other diseases of the circulatory systen	Z82.4	9330	463010	2.02%
ukb-b-16471	Diagnoses - secondary ICD10: Z85.0 Personal history of malignant neoplasm of digestive organs	Z85.0	3570	463010	0.77%
ukb-b-20142	Diagnoses - secondary ICD10: Z85.3 Personal history of malignant neoplasm of breast	Z85.3	5208	463010	1.12%
ukb-b-11709	Diagnoses - secondary ICD10: Z85.4 Personal history of malignant neoplasm of genital organs	Z85.4	2692	463010	0.58%
ukb-b-7299	Diagnoses - secondary ICD10: Z85.5 Personal history of malignant neoplasm of urinary tract	Z85.5	2117	463010	0.46%
ukb-b-11231	Diagnoses - secondary ICD10: Z85.8 Personal history of malignant neoplasms of other organs and systems	Z85.8	3509	463010	0.76%
ukb-b-3897	Diagnoses - secondary ICD10: Z86.0 Personal history of other neoplasms	Z86.0	4011	463010	0.87%
ukb-b-19864	Diagnoses - secondary ICD10: Z86.1 Personal history of infectious and parasitic diseases	Z86.1	1375	463010	0.30%
ukb-b-1585	Diagnoses - secondary ICD10: Z86.4 Personal history of psychoactive substance abuse	Z86.4	11793	463010	2.55%
ukb-b-546	Diagnoses - secondary ICD10: Z86.6 Personal history of diseases of the nervous system and sense organs	Z86.6	3119	463010	0.67%
ukb-b-13782	Diagnoses - secondary ICD10: Z86.7 Personal history of diseases of the circulatory system	Z86.7	10173	463010	2.20%
ukb-b-16364	Diagnoses - secondary ICD10: Z87.1 Personal history of diseases of the digestive system	Z87.1	10458	463010	2.26%
ukb-b-18023	Diagnoses - secondary ICD10: Z87.3 Personal history of diseases of the musculoskeletal system and connective tissue	Z87.3	1285	463010	0.28%
ukb-b-10880	Diagnoses - secondary ICD10: Z87.4 Personal history of diseases of the genito-urinary system	Z87.4	4538	463010	0.98%
ukb-b-5911	Diagnoses - secondary ICD10: Z88.0 Personal history of allergy to penicillin	Z88.0	11348	463010	2.45%
ukb-b-9841	Diagnoses - secondary ICD10: Z88.1 Personal history of allergy to other antibiotic agents	Z88.1	2150	463010	0.46%
ukb-b-4267	Diagnoses - secondary ICD10: Z88.6 Personal history of allergy to analgesic agent	Z88.6	2685	463010	0.58%
ukb-b-4601	Diagnoses - secondary ICD10: Z88.8 Personal history of allergy to other drugs, medicaments and biological substances	Z88.8	2694	463010	0.58%
ukb-b-16641	Diagnoses - secondary ICD10: Z90.1 Acquired absence of breast(s)	Z90.1	1661	463010	0.36%
ukb-b-14829	Diagnoses - secondary ICD10: Z90.4 Acquired absence of other parts of digestive tract	Z90.4	3438	463010	0.74%
ukb-b-20424	Diagnoses - secondary ICD10: Z90.7 Acquired absence of genital organ(s)	Z90.7	3446	463010	0.74%
ukb-b-16702	Diagnoses - secondary ICD10: Z91.0 Personal history of allergy, other than to drugs and biological substances	Z91.0	1658	463010	0.36%
ukb-b-7352	Diagnoses - secondary ICD10: Z92.1 Personal history of long-term (current) use of anticoagulants	Z92.1	5254	463010	1.13%
ukb-b-10911	Diagnoses - secondary ICD10: Z92.2 Personal history of long-term (current) use of other medicaments	Z92.2	7915	463010	1.71%
ukb-b-14446	Diagnoses - secondary ICD10: Z92.3 Personal history of irradiation	Z92.3	1124	463010	0.24%
ukb-b-8092	Diagnoses - secondary ICD10: Z92.4 Personal history of major surgery, not elsewhere classified	Z92.4	1039	463010	0.22%
ukb-b-16967	Diagnoses - secondary ICD10: Z95.0 Presence of cardiac pacemaker	Z95.0	1266	463010	0.27%
ukb-b-11064	Diagnoses - secondary ICD10: Z95.1 Presence of aortocoronary bypass graft	Z95.1	3358	463010	0.73%
ukb-b-15748	Diagnoses - secondary ICD10: Z95.5 Presence of coronary angioplasty implant and graft	Z95.5	3724	463010	0.80%
ukb-b-15230	Diagnoses - secondary ICD10: Z95.8 Presence of other cardiac and vascular implants and grafts	Z95.8	1034	463010	0.22%
ukb-b-11639	Diagnoses - secondary ICD10: Z96.1 Presence of intraocular lens	Z96.1	2240	463010	0.48%
ukb-b-20477	Diagnoses - secondary ICD10: Z96.6 Presence of orthopaedic joint implants	Z96.6	5687	463010	1.23%
ukb-b-2630	Diagnoses - secondary ICD10: Z98.0 Intestinal bypass and anastomosis status	Z98.0	1178	463010	0.25%

Table S5 Genome-wide significant association of SNPs as genetic instruments of eight prioritized proteins

protein	SNP	trait	catalog	pubmed id	beta	standard error (beta)	P value
COLEC11	rs6542680	Impedance of leg right	GWAS	UKBB	0.01694	0.002888	4.451E-09
COLEC11	rs6542680	Impedance of whole body	GWAS	UKBB	0.01413	0.002434	6.449E-09
COLEC11	rs6542680	Collectin-11	pQTL	29875488	1.02	0.0283	4.79E-285
COLEC11	rs6542680	Protein C-ets-2	pQTL	29875488	0.222	0.0332	2.14E-11
COLEC11	rs6542680	Sterol-4-alpha-carboxylate 3-dehydrogenase, decarboxylating	pQTL	29875488	0.3844	0.0327	6.17E-32
COLEC11	rs6542680	Transcription factor IIIB 90 kDa subunit	pQTL	29875488	0.2024	0.0332	1.07E-09
COLEC11	rs6542680	Collectin-10	pQTL	29875488	-0.4223	0.0326	2.19E-38
COLEC11	rs6542680	Interleukin-19	pQTL	29875488	0.8113	0.0303	3.09E-158
COLEC11	rs6542680	Isochorismatase domain-containing protein 1	pQTL	29875488	0.2366	0.0332	9.77E-13
COLEC11	rs6542680	Sialic acid-binding Ig-like lectin 14	pQTL	29875488	0.4776	0.0323	2.51E-49
COLEC11	rs6542680	Splicing factor 1	pQTL	29875488	0.2755	0.0331	7.94E-17
COLEC11	rs6542680	Tumor necrosis factor receptor superfamily member 1A	pQTL	29875488	0.2089	0.0332	3.24E-10
COLEC11	rs6542680	Activated Protein C	pQTL	29875488	0.1829	0.0328	2.51E-08
COLEC11	rs6542680	Basigin	pQTL	29875488	0.1937	0.0328	3.47E-09
COLEC11	rs6542680	Cellular retinoic acid-binding protein 2	pQTL	29875488	0.1908	0.0328	6.03E-09
COLEC11	rs6542680	Immunoglobulin superfamily member 8	pQTL	29875488	0.1933	0.0328	3.8E-09
COLEC11	rs6542680	MAX gene-associated protein	pQTL	29875488	0.1847	0.0328	1.86E-08
COLEC11	rs6542680	Neurexin-3-beta	pQTL	29875488	0.2472	0.0327	4.07E-14
COLEC11	rs6542680	Peptidyl-prolyl cis-trans isomerase B	pQTL	29875488	0.1969	0.0328	1.95E-09
COLEC11	rs6542680	Angiopoietin-related protein 4	pQTL	29875488	0.206	0.0329	3.72E-10
COLEC11	rs6542680	Dipeptidyl peptidase 2	pQTL	29875488	0.205	0.0329	4.57E-10
COLEC11	rs6542680	Guanine deaminase	pQTL	29875488	0.2199	0.0328	2.14E-11
COLEC11	rs6542680	Interleukin-1 beta	pQTL	29875488	0.3247	0.0326	2.19E-23
COLEC11	rs6542680	Isochorismatase domain-containing protein 1	pQTL	29875488	0.2342	0.0328	9.77E-13
COLEC11	rs6542680	Peptidyl-prolyl cis-trans isomerase FKBP14	pQTL	29875488	0.2768	0.0327	2.69E-17
COLEC11	rs6542680	Disintegrin and metalloproteinase domain-containing protein 11	pQTL	29875488	0.3383	0.032	3.89E-26
COLEC11	rs6542680	High affinity nerve growth factor receptor	pQTL	29875488	0.198	0.0323	9.12E-10
COLEC11	rs6542680	Melanoma-associated antigen 3	pQTL	29875488	0.2748	0.0322	1.32E-17
POSTN	rs7329947	Periostin	pQTL	28240269	0.2462	0.04403	2.896E-08
POSTN	rs7329947	Periostin	pQTL	29875488	0.1684	0.0264	1.86E-10
RPN1	rs9880064	Basophil count	GWAS	27863252	-0.03911	0.003589	1.195E-27
RPN1	rs9880064	Basophil percentage of granulocytes	GWAS	27863252	-0.03888	0.003593	2.774E-27
RPN1	rs9880064	Basophil percentage of white cells	GWAS	27863252	-0.03861	0.003562	2.217E-27
RPN1	rs9880064	Monocyte count	GWAS	27863252	-0.07206	0.003661	2.851E-86
RPN1	rs9880064	Monocyte percentage of white cells	GWAS	27863252	-0.0755	0.003654	7.95E-95
RPN1	rs9880064	Monocyte count	GWAS	23314186	0.23	0.02797	2E-16
RPN1	rs9880064	Granulocyte percentage of myeloid white cells	GWAS	27863252	0.0629	0.003666	5.407E-66
RPN1	rs9880064	Eosinophil count	GWAS	27863252	-0.02125	0.003664	6.582E-09
RPN1	rs9880064	Eosinophil percentage of granulocytes	GWAS	27863252	-0.02053	0.003676	2.317E-08
RPN1	rs9880064	Neutrophil percentage of granulocytes	GWAS	27863252	0.02711	0.003676	1.647E-13
RPN1	rs9880064	Sum eosinophil basophil counts	GWAS	27863252	-0.02827	0.003667	1.274E-14
RPN1	rs9880064	Dolichyl-diphosphooligosaccharide--protein glycosyltransferase subunit 1	pQTL	29875488	0.3442	0.0247	3.89E-44
RSPO3	rs1892172	Waist hip ratio in females	GWAS	23754948	-0.046	0.007	7.9E-11
RSPO3	rs1892172	Waist hip ratio in males	GWAS	25673412	-0.029	0.0047	1.4E-09
RSPO3	rs1892172	Waist hip ratio in female non-smokers	GWAS	28443625	-0.0206	0.0033	4.904E-10
RSPO3	rs1892172	Waist hip ratio in non-smokers	GWAS	28443625	-0.0188	0.0029	8.076E-11
RSPO3	rs1892172	Waist circumference adjusted for smoking in females	GWAS	28443625	-0.04	0.0044	2.519E-19
RSPO3	rs1892172	Waist hip ratio adjusted for smoking	GWAS	28443625	-0.0372	0.0036	1.236E-24
RSPO3	rs1892172	Waist hip ratio	GWAS	23754948	-0.03866	0.005149	6.012E-14
RSPO3	rs1892172	Waist hip ratio adjusted for BMI in females less than or equal to 50 years of age	GWAS	26426971	-0.053	0.0071	1.2E-13
RSPO3	rs1892172	Waist hip ratio adjusted for BMI in males less than or equal to 50 years of age	GWAS	26426971	-0.044	0.0078	0.000000019
RSPO3	rs1892172	Waist circumference in non-smokers	GWAS	28443625	-0.0268	0.0041	4.241E-11
RSPO3	rs1892172	Fractured or broken bones in last 5 years	GWAS	UKBB	0.004624	0.0007222	1.529E-10
RSPO3	rs1892172	Heel bone mineral density right	GWAS	UKBB	-0.07063	0.004142	3.976E-65
RSPO3	rs1892172	Waist hip ratio in females	GWAS	23754948	-0.046	0.007	7.9E-11
RSPO3	rs1892172	Waist hip ratio	GWAS	23754948	-0.03866	0.005149	6.012E-14
RSPO3	rs1892172	Heel bone mineral density left	GWAS	UKBB	-0.07134	0.004167	1.291E-65
RSPO3	rs1892172	Hip circumference	GWAS	UKBB	0.01448	0.002416	2.031E-09
RSPO3	rs1892172	Hematocrit	GWAS	27863252	-0.02162	0.003544	1.062E-09
RSPO3	rs1892172	Hemoglobin concentration	GWAS	27863252	-0.0231	0.00356	8.727E-11
RSPO3	rs1892172	Waist circumference adjusted for BMI	GWAS	25673412	-0.025	0.0032	3.7E-15
RSPO3	rs1892172	Waist hip ratio in females	GWAS	23754948	-0.046	0.007	7.9E-11
RSPO3	rs1892172	Waist hip ratio	GWAS	23754948	-0.03866	0.005149	6.012E-14
RSPO3	rs1892172	Waist hip ratio adjusted for BMI in females less than or equal to 50 years of age	GWAS	26426971	-0.053	0.0071	1.2E-13
RSPO3	rs1892172	Waist circumference in female non-smokers	GWAS	28443625	-0.0312	0.0048	7.133E-11
RSPO3	rs1892172	Waist circumference adjusted for smoking	GWAS	28443625	-0.0224	0.0036	7.798E-10
RSPO3	rs1892172	High density lipoprotein	GWAS	24097068	0.0185	0.0034	6.089E-09
RSPO3	rs1892172	Fracture of forearm	GWAS	UKBB	0.001391	0.0002402	6.978E-09
RSPO3	rs1892172	Hip circumference adjusted for BMI	GWAS	25673412	0.02	0.0035	0.000000003
RSPO3	rs1892172	Waist hip ratio in physically active females	GWAS	28448500	-0.0501	0.0055	5.01E-20
RSPO3	rs1892172	Waist hip ratio in physically active indivdiuals	GWAS	28448500	-0.0429	0.0045	5.93E-22
RSPO3	rs1892172	Waist hip ratio in females	GWAS	25673412	-0.04	0.0043	2.1E-20
RSPO3	rs1892172	Waist hip ratio adjusted for physical activity in females	GWAS	28448500	-0.0469	0.0048	1.27E-22
RSPO3	rs1892172	Waist hip ratio adjusted for physical activity	GWAS	28448500	-0.0406	0.004	2.97E-24
RSPO3	rs1892172	Waist hip ratio	GWAS	25673412	-0.036	0.0033	8.2E-27
RSPO3	rs1892172	Waist hip ratio adjusted for BMI in females greater than 50 years of age	GWAS	26426971	-0.041	0.0056	2.2E-13
RSPO3	rs1892172	Waist hip ratio adjusted for BMI	GWAS	25673412	-0.038	0.0033	1.3E-30
RSPO3	rs1892172	Waist circumference in physically active indivdiuals	GWAS	28448500	-0.0265	0.0043	4.45E-10
RSPO3	rs1892172	Waist circumference adjusted for physical activity	GWAS	28448500	-0.0229	0.0037	1.02E-09
RSPO3	rs1892172	Heel bone mineral density	GWAS	UKBB	-0.06482	0.003124	1.606E-95
RSPO3	rs1892172	Red blood cell count	GWAS	27863252	-0.02215	0.003565	5.162E-10
RSPO3	rs1892172	Chymotrypsinogen B	pQTL	29875488	-0.1427	0.0249	1.02E-08
RSPO3	rs1892172	R-spondin-3	pQTL	29875488	-0.2622	0.0246	1.78E-26
VAT1	rs4239148	Age at menopause	GWAS	26414677	0.13	0.02	1.3E-09

Table S6 MR Steiger filtering analysis of SNPs as genetic instruments of eight prioritized proteins

protein	SNP	chromosome	position	effect allele	beta (protein)	beta (varicose veins)	P value (protein)	P value (varicose veins)	sample size (protein)	sample size (varicose veins)	Steiger direction	Steiger P value
COLEC11	rs6542680	2	3592552	C	0.657269	0.061	1E-200	0.000001362	7213	459702	TRUE	2.3269E-206
IRF3	rs10415576	19	49661133	C	-0.452409	-0.0532	2.613E-161	0.000002489	7213	459702	TRUE	3.8414E-147
LUM	rs3741835	12	91111364	G	-0.712224	0.1673	3.01971E-33	0.000004628	7213	459702	TRUE	1.16387E-29
POSTN	rs7329947	13	37524954	A	0.240615	-0.0512	1.22593E-45	0.000002747	7213	459702	TRUE	4.59126E-41
RPN1	rs9880064	3	128636666	A	0.418723	-0.0484	2.6542E-137	0.00001088	7213	459702	TRUE	8.5997E-126
RSPO3	rs1892172	6	127155371	G	-0.492365	0.0646	1E-200	1.818E-09	7213	459702	TRUE	1.1188E-188
SARS2	rs1808661	19	38933313	G	0.113394	0.0539	3.1417E-11	0.00000139	7213	459702	TRUE	2.18888E-09
VAT1	rs4239148	17	43077840	A	-0.0959998	0.0494	4.8105E-08	0.00001023	7213	459702	TRUE	1.15226E-06

Table S7 Phenome-wide Mendelian Randomization analysis to reveal potential on-target side effects of COLEC11

protein	disease	ICD-10 code	OR (95%CI)	P value
COLEC11	Underlying (primary) cause of death: ICD10: E85.4 Organ-limited amyloidosis	E85.4	1.000 (0.998, 1.002)	0.670917428
COLEC11	Underlying (primary) cause of death: ICD10: J84.1 Other interstitial pulmonary diseases with fibrosis	J84.1	0.993 (0.986, 1.000)	0.040837276
COLEC11	Diagnoses - main ICD10: B37 Candidiasis	B37	1.000 (1.000, 1.000)	0.004439908
COLEC11	Diagnoses - main ICD10: C44 Other malignant neoplasms of skin	C44	1.000 (0.999, 1.002)	0.499110557
COLEC11	Diagnoses - main ICD10: C50 Malignant neoplasm of breast	C50	1.000 (0.999, 1.002)	0.518603615
COLEC11	Diagnoses - main ICD10: C61 Malignant neoplasm of prostate	C61	1.000 (0.999, 1.000)	0.220597181
COLEC11	Diagnoses - main ICD10: D12 Benign neoplasm of colon rectum anus and anal canal	D12	1.000 (0.999, 1.001)	0.895963844
COLEC11	Diagnoses - main ICD10: D25 Leiomyoma of uterus	D25	0.999 (0.998, 1.001)	0.298911753
COLEC11	Diagnoses - main ICD10: E03 Other hypothyroidism	E03	1.000 (1.000, 1.000)	0.948530045
COLEC11	Diagnoses - main ICD10: E04 Other non-toxic goitre	E04	1.000 (1.000, 1.000)	0.988137765
COLEC11	Diagnoses - main ICD10: F31 Bipolar affective disorder	F31	1.000 (1.000, 1.000)	0.497702936
COLEC11	Diagnoses - main ICD10: F43 Reaction to severe stress and adjustment disorders	F43	1.000 (1.000, 1.000)	0.783567347
COLEC11	Diagnoses - main ICD10: G47 Sleep disorders	G47	1.000 (0.999, 1.001)	0.866292169
COLEC11	Diagnoses - main ICD10: G56 Mononeuropathies of upper limb	G56	1.000 (0.999, 1.001)	0.873529563
COLEC11	Diagnoses - main ICD10: H25 Senile cataract	H25	1.001 (1.000, 1.001)	0.180305777
COLEC11	Diagnoses - main ICD10: H26 Other cataract	H26	1.001 (0.999, 1.002)	0.282107822
COLEC11	Diagnoses - main ICD10: I10 Essential (primary) hypertension	I10	1.000 (0.999, 1.000)	0.372034827
COLEC11	Diagnoses - main ICD10: I20 Angina pectoris	I20	1.002 (1.001, 1.003)	0.001981213
COLEC11	Diagnoses - main ICD10: I21 Acute myocardial infarction	I21	0.999 (0.998, 1.000)	0.098657033
COLEC11	Diagnoses - main ICD10: I25 Chronic ischaemic heart disease	I25	1.001 (1.000, 1.003)	0.157666746
COLEC11	Diagnoses - main ICD10: I30 Acute pericarditis	I30	1.000 (1.000, 1.000)	0.525298547
COLEC11	Diagnoses - main ICD10: I48 Atrial fibrillation and flutter	I48	1.000 (0.999, 1.001)	0.443582244
COLEC11	Diagnoses - main ICD10: I80 Phlebitis and thrombophlebitis	I80	1.000 (0.999, 1.001)	0.7049886
COLEC11	Diagnoses - main ICD10: I84 Haemorrhoids	I84	1.002 (1.001, 1.004)	0.003947244
COLEC11	Diagnoses - main ICD10: J22 Unspecified acute lower respiratory infection	J22	1.000 (0.999, 1.001)	0.650326862
COLEC11	Diagnoses - main ICD10: J33 Nasal polyp	J33	1.000 (0.999, 1.001)	0.830288279
COLEC11	Diagnoses - main ICD10: J34 Other disorders of nose and nasal sinuses	J34	0.999 (0.998, 1.000)	0.098384475
COLEC11	Diagnoses - main ICD10: J44 Other chronic obstructive pulmonary disease	J44	1.000 (1.000, 1.001)	0.750756624
COLEC11	Diagnoses - main ICD10: K20 Oesophagitis	K20	1.001 (1.000, 1.002)	0.219864269
COLEC11	Diagnoses - main ICD10: K21 Gastro-oesophageal reflux disease	K21	1.000 (0.999, 1.002)	0.51776719
COLEC11	Diagnoses - main ICD10: K22 Other diseases of oesophagus	K22	0.999 (0.998, 1.000)	0.04825145
COLEC11	Diagnoses - main ICD10: K29 Gastritis and duodenitis	K29	1.002 (1.001, 1.003)	0.00739318
COLEC11	Diagnoses - main ICD10: K35 Acute appendicitis	K35	1.000 (0.999, 1.000)	0.151561043
COLEC11	Diagnoses - main ICD10: K40 Inguinal hernia	K40	0.999 (0.998, 1.001)	0.396330154
COLEC11	Diagnoses - main ICD10: K43 Ventral hernia	K43	1.000 (0.999, 1.001)	0.960076712
COLEC11	Diagnoses - main ICD10: K44 Diaphragmatic hernia	K44	1.000 (0.998, 1.001)	0.534158993
COLEC11	Diagnoses - main ICD10: K50 Crohn's disease [regional enteritis]	K50	1.000 (1.000, 1.001)	0.656668813
COLEC11	Diagnoses - main ICD10: K51 Ulcerative colitis	K51	1.000 (0.999, 1.001)	0.824393061

COLEC11	Diagnoses - main ICD10: K52 Other non-infective gastro-enteritis and colitis	K52	1.000 (0.999, 1.001)	0.891761507
COLEC11	Diagnoses - main ICD10: K57 Diverticular disease of intestine	K57	0.999 (0.998, 1.001)	0.257906677
COLEC11	Diagnoses - main ICD10: K60 Fissure and fistula of anal and rectal regions	K60	1.000 (0.999, 1.000)	0.443812667
COLEC11	Diagnoses - main ICD10: K62 Other diseases of anus and rectum	K62	1.001 (0.999, 1.003)	0.259743773
COLEC11	Diagnoses - main ICD10: K76 Other diseases of liver	K76	1.000 (1.000, 1.000)	0.6811209
COLEC11	Diagnoses - main ICD10: K80 Cholelithiasis	K80	1.000 (0.999, 1.002)	0.715174233
COLEC11	Diagnoses - main ICD10: L03 Cellulitis	L03	1.001 (1.000, 1.002)	0.003112885
COLEC11	Diagnoses - main ICD10: M10 Gout	M10	1.000 (1.000, 1.000)	0.275616986
COLEC11	Diagnoses - main ICD10: M16 Coxarthrosis [arthrosis of hip]	M16	1.000 (0.999, 1.001)	0.860281518
COLEC11	Diagnoses - main ICD10: M17 Gonarthrosis [arthrosis of knee]	M17	1.000 (0.999, 1.001)	0.830906663
COLEC11	Diagnoses - main ICD10: M20 Acquired deformities of fingers and toes	M20	0.999 (0.998, 1.000)	0.04791927
COLEC11	Diagnoses - main ICD10: M21 Other acquired deformities of limbs	M21	1.000 (1.000, 1.000)	0.91228777
COLEC11	Diagnoses - main ICD10: M23 Internal derangement of knee	M23	1.000 (0.998, 1.001)	0.681355076
COLEC11	Diagnoses - main ICD10: M24 Other specific joint derangements	M24	1.000 (1.000, 1.001)	0.073820155
COLEC11	Diagnoses - main ICD10: M25 Other joint disorders not elsewhere classified	M25	1.001 (1.000, 1.003)	0.014071369
COLEC11	Diagnoses - main ICD10: M54 Dorsalgia	M54	1.001 (1.000, 1.002)	0.19400345
COLEC11	Diagnoses - main ICD10: M67 Other disorders of synovium and tendon	M67	1.001 (1.000, 1.001)	0.101668338
COLEC11	Diagnoses - main ICD10: M70 Soft tissue disorders related to use overuse and pressure	M70	1.000 (1.000, 1.001)	0.539106123
COLEC11	Diagnoses - main ICD10: M72 Fibroblastic disorders	M72	1.000 (0.999, 1.000)	0.431592582
COLEC11	Diagnoses - main ICD10: N19 Unspecified renal failure	N19	1.000 (1.000, 1.000)	0.398777235
COLEC11	Diagnoses - main ICD10: N20 Calculus of kidney and ureter	N20	1.000 (1.000, 1.001)	0.275550748
COLEC11	Diagnoses - main ICD10: N32 Other disorders of bladder	N32	0.999 (0.999, 1.000)	0.245874004
COLEC11	Diagnoses - main ICD10: N40 Hyperplasia of prostate	N40	1.000 (0.999, 1.001)	0.495977585
COLEC11	Diagnoses - main ICD10: N81 Female genital prolapse	N81	1.001 (1.000, 1.003)	0.017005729
COLEC11	Diagnoses - main ICD10: N92 Excessive frequent and irregular menstruation	N92	1.000 (0.999, 1.002)	0.600876975
COLEC11	Diagnoses - main ICD10: O75 Other complications of labour and delivery not elsewhere classified	O75	1.000 (1.000, 1.000)	0.903124341
COLEC11	Diagnoses - main ICD10: R04 Haemorrhage from respiratory passages	R04	1.000 (0.999, 1.001)	0.627540682
COLEC11	Diagnoses - main ICD10: R07 Pain in throat and chest	R07	1.001 (0.999, 1.003)	0.455791464
COLEC11	Diagnoses - main ICD10: R10 Abdominal and pelvic pain	R10	0.999 (0.997, 1.001)	0.358593703
COLEC11	Diagnoses - main ICD10: R11 Nausea and vomiting	R11	1.000 (0.999, 1.001)	0.947557978
COLEC11	Diagnoses - main ICD10: R14 Flatulence and related conditions	R14	1.000 (1.000, 1.000)	0.666292565
COLEC11	Diagnoses - main ICD10: R31 Unspecified haematuria	R31	1.001 (0.999, 1.002)	0.251943864
COLEC11	Diagnoses - main ICD10: R35 Polyuria	R35	1.000 (1.000, 1.001)	0.658228849
COLEC11	Diagnoses - main ICD10: R55 Syncope and collapse	R55	1.000 (0.999, 1.001)	0.681302221
COLEC11	Diagnoses - main ICD10: R69 Unknown and unspecified causes of morbidity	R69	1.000 (0.999, 1.001)	0.917302829
COLEC11	Diagnoses - main ICD10: S09 Other and unspecified injuries of head	S09	1.000 (0.999, 1.000)	0.631527772
COLEC11	Diagnoses - main ICD10: S52 Fracture of forearm	S52	1.001 (1.000, 1.002)	0.069183436
COLEC11	Diagnoses - main ICD10: S66 Injury of muscle and tendon at wrist and hand level	S66	1.000 (1.000, 1.000)	0.916501104
COLEC11	Diagnoses - main ICD10: S76 Injury of muscle and tendon at hip and thigh level	S76	1.000 (1.000, 1.000)	0.230808183
COLEC11	Diagnoses - main ICD10: T84 Complications of internal orthopaedic prosthetic devices implants and grafts	T84	1.000 (0.999, 1.001)	0.954684859

COLEC11	Diagnoses - main ICD10: Z09 Follow-up examination after treatment for conditions other than malignant neoplasm	Z09	1.001 (1.000, 1.002)	0.11831875
COLEC11	Diagnoses - main ICD10: Z47 Other orthopaedic follow-up care	Z47	1.000 (1.000, 1.001)	0.468310844
COLEC11	Diagnoses - main ICD10: Z80 Family history of malignant neoplasm	Z80	1.000 (1.000, 1.000)	0.269638011
COLEC11	Diagnoses - main ICD10: Z09.0 Follow-up examination after surgery for other conditions	Z09.0	1.000 (0.999, 1.001)	0.766991007
COLEC11	Diagnoses - secondary ICD10: K21.0 Gastro-oesophageal reflux disease with oesophagitis	K21.0	1.001 (1.000, 1.001)	0.077392907
COLEC11	Type of cancer: ICD10: C44.3 Skin of other and unspecified parts of face	C44.3	1.000 (0.999, 1.001)	0.511524314
COLEC11	Diagnoses - secondary ICD10: R19.4 Change in bowel habit	R19.4	1.000 (0.999, 1.000)	0.584400446
COLEC11	Diagnoses - secondary ICD10: Z03.8 Observation for other suspected diseases and conditions	Z03.8	1.000 (0.999, 1.000)	0.623117798
COLEC11	Diagnoses - secondary ICD10: I50.1 Left ventricular failure	I50.1	1.000 (1.000, 1.001)	0.196038045
COLEC11	Diagnoses - main ICD10: D12.5 Sigmoid colon	D12.5	1.000 (1.000, 1.001)	0.670812086
COLEC11	Diagnoses - main ICD10: Z09.9 Follow-up examination after unspecified treatment for other conditions	Z09.9	1.000 (0.999, 1.000)	0.110512196
COLEC11	Diagnoses - secondary ICD10: E10.9 Without complications	E10.9	1.000 (0.999, 1.000)	0.412653921
COLEC11	Diagnoses - main ICD10: O70.1 Second degree perineal laceration during delivery	O70.1	1.000 (0.999, 1.001)	0.941958001
COLEC11	Diagnoses - main ICD10: R07.4 Chest pain, unspecified	R07.4	1.000 (0.998, 1.001)	0.915722633
COLEC11	Diagnoses - main ICD10: K57.3 Diverticular disease of large intestine without perforation or abscess	K57.3	0.999 (0.998, 1.000)	0.239493941
COLEC11	Diagnoses - secondary ICD10: Z87.4 Personal history of diseases of the genito-urinary system	Z87.4	1.000 (1.000, 1.001)	0.258097199
COLEC11	Diagnoses - secondary ICD10: Z92.2 Personal history of long-term (current) use of other medicaments	Z92.2	1.000 (0.999, 1.002)	0.369991486
COLEC11	Diagnoses - main ICD10: K80.2 Calculus of gallbladder without cholecystitis	K80.2	1.000 (0.999, 1.001)	0.639795337
COLEC11	Diagnoses - main ICD10: R79.8 Other specified abnormal findings of blood chemistry	R79.8	1.000 (0.999, 1.000)	0.738437857
COLEC11	Diagnoses - secondary ICD10: Z95.1 Presence of aortocoronary bypass graft	Z95.1	1.000 (0.999, 1.001)	0.994904009
COLEC11	Diagnoses - secondary ICD10: Z85.8 Personal history of malignant neoplasms of other organs and systems	Z85.8	0.999 (0.999, 1.000)	0.071187688
COLEC11	Diagnoses - main ICD10: I84.9 Unspecified haemorrhoids without complication	I84.9	1.000 (1.000, 1.001)	0.337929319
COLEC11	Diagnoses - main ICD10: N81.1 Cystocele	N81.1	1.001 (1.000, 1.001)	0.029682052
COLEC11	Diagnoses - secondary ICD10: Z51.1 Chemotherapy session for neoplasm	Z51.1	1.000 (0.999, 1.001)	0.950155116
COLEC11	Diagnoses - main ICD10: N92.0 Excessive and frequent menstruation with regular cycle	N92.0	1.000 (0.999, 1.001)	0.924335841
COLEC11	Diagnoses - main ICD10: K62.5 Haemorrhage of anus and rectum	K62.5	1.000 (0.999, 1.001)	0.655570773
COLEC11	Diagnoses - secondary ICD10: Z96.1 Presence of intraocular lens	Z96.1	1.000 (1.000, 1.001)	0.566531715
COLEC11	Diagnoses - secondary ICD10: Z85.4 Personal history of malignant neoplasm of genital organs	Z85.4	0.999 (0.999, 1.000)	0.078952993
COLEC11	Diagnoses - main ICD10: L03.1 Cellulitis of other parts of limb	L03.1	1.001 (1.000, 1.002)	0.003524028
COLEC11	Diagnoses - secondary ICD10: G55.1 Nerve root and plexus compressions in intervertebral disk disorders	G55.1	1.000 (0.999, 1.000)	0.246453846
COLEC11	Diagnoses - main ICD10: M67.4 Ganglion	M67.4	1.001 (1.000, 1.001)	0.049791183
COLEC11	Diagnoses - main ICD10: H26.9 Cataract, unspecified	H26.9	1.001 (0.999, 1.002)	0.299456951
COLEC11	Diagnoses - secondary ICD10: K30 Dyspepsia	K30	1.000 (1.000, 1.001)	0.229216708
COLEC11	Diagnoses - main ICD10: Z08.0 Follow-up examination after surgery for malignant neoplasm	Z08.0	1.000 (0.999, 1.000)	0.295881745
COLEC11	Diagnoses - secondary ICD10: E78.0 Pure hypercholesterolaemia	E78.0	1.003 (1.002, 1.005)	0.000127554
COLEC11	Diagnoses - main ICD10: N84.0 Polyp of corpus uteri	N84.0	0.999 (0.998, 1.000)	0.106445652
COLEC11	Diagnoses - secondary ICD10: Z80.0 Family history of malignant neoplasm of digestive organs	Z80.0	1.000 (0.999, 1.001)	0.623779079
COLEC11	Diagnoses - main ICD10: K22.1 Ulcer of oesophagus	K22.1	1.000 (0.999, 1.000)	0.499634212
COLEC11	Diagnoses - secondary ICD10: Z86.7 Personal history of diseases of the circulatory system	Z86.7	1.000 (0.999, 1.002)	0.520644631
COLEC11	Diagnoses - secondary ICD10: Y83.1 Surgical operation with implant of artificial internal device	Y83.1	1.001 (1.000, 1.002)	0.014512172

COLEC11	Diagnoses - secondary ICD10: R63.4 Abnormal weight loss	R63.4	1.000 (0.999, 1.000)	0.665166848
COLEC11	Diagnoses - main ICD10: K21.9 Gastro-oesophageal reflux disease without oesophagitis	K21.9	1.000 (0.999, 1.001)	0.89468319
COLEC11	Diagnoses - main ICD10: M75.4 Impingement syndrome of shoulder	M75.4	1.000 (0.999, 1.001)	0.964274214
COLEC11	Diagnoses - main ICD10: I20.9 Angina pectoris, unspecified	I20.9	1.000 (0.999, 1.001)	0.504234619
COLEC11	Diagnoses - secondary ICD10: X59.9 Unspecified place	X59.9	1.000 (0.999, 1.001)	0.879503353
COLEC11	Diagnoses - secondary ICD10: K57.9 Diverticular disease of intestine, part unspecified, without perforation or abs	K57.9	1.000 (0.999, 1.001)	0.880482878
COLEC11	Diagnoses - main ICD10: Z09.8 Follow-up examination after other treatment for other conditions	Z09.8	1.000 (0.999, 1.001)	0.952261451
COLEC11	Diagnoses - main ICD10: K42.9 Umbilical hernia without obstruction or gangrene	K42.9	1.001 (1.000, 1.001)	0.02603877
COLEC11	Diagnoses - main ICD10: R07.3 Other chest pain	R07.3	1.000 (0.999, 1.001)	0.370336161
COLEC11	Diagnoses - main ICD10: D64.9 Anaemia, unspecified	D64.9	1.001 (1.000, 1.001)	0.154085634
COLEC11	Diagnoses - secondary ICD10: Z90.4 Acquired absence of other parts of digestive tract	Z90.4	1.000 (0.999, 1.001)	0.788576568
COLEC11	Diagnoses - secondary ICD10: M13.99 Arthritis, unspecified (Site unspecified)	M13.99	1.000 (1.000, 1.001)	0.499764866
COLEC11	Diagnoses - secondary ICD10: K29.7 Gastritis, unspecified	K29.7	0.999 (0.999, 1.000)	0.264007632
COLEC11	Diagnoses - secondary ICD10: E66.9 Obesity, unspecified	E66.9	1.001 (1.000, 1.002)	0.089447483
COLEC11	Diagnoses - main ICD10: M54.5 Low back pain	M54.5	1.000 (0.999, 1.001)	0.944868862
COLEC11	Type of cancer: ICD10: C44.5 Skin of trunk	C44.5	1.000 (1.000, 1.001)	0.314297858
COLEC11	Diagnoses - secondary ICD10: Z95.5 Presence of coronary angioplasty implant and graft	Z95.5	1.001 (1.000, 1.002)	0.032820492
COLEC11	Diagnoses - secondary ICD10: Z86.4 Personal history of psychoactive substance abuse	Z86.4	1.001 (1.000, 1.002)	0.068999534
COLEC11	Diagnoses - secondary ICD10: G40.9 Epilepsy, unspecified	G40.9	1.000 (0.999, 1.001)	0.854325704
COLEC11	Diagnoses - secondary ICD10: Z51.0 Radiotherapy session	Z51.0	1.000 (1.000, 1.001)	0.434791909
COLEC11	Diagnoses - secondary ICD10: Z87.1 Personal history of diseases of the digestive system	Z87.1	1.001 (0.999, 1.002)	0.31848636
COLEC11	Diagnoses - secondary ICD10: Z85.0 Personal history of malignant neoplasm of digestive organs	Z85.0	1.000 (0.999, 1.000)	0.202744982
COLEC11	Diagnoses - secondary ICD10: I25.8 Other forms of chronic ischaemic heart disease	I25.8	1.001 (1.000, 1.001)	0.197847029
COLEC11	Diagnoses - secondary ICD10: I25.2 Old myocardial infarction	I25.2	1.001 (1.000, 1.001)	0.054605128
COLEC11	Diagnoses - main ICD10: I25.1 Atherosclerotic heart disease	I25.1	1.001 (1.000, 1.003)	0.037205859
COLEC11	Diagnoses - secondary ICD10: J44.9 Chronic obstructive pulmonary disease, unspecified	J44.9	1.001 (1.001, 1.002)	0.000700943
COLEC11	Diagnoses - main ICD10: G47.3 Sleep apnoea	G47.3	1.000 (0.999, 1.000)	0.714363286
COLEC11	Diagnoses - secondary ICD10: N95.0 Postmenopausal bleeding	N95.0	1.000 (0.999, 1.000)	0.298620826
COLEC11	Diagnoses - secondary ICD10: J45.9 Asthma, unspecified	J45.9	1.001 (1.000, 1.003)	0.164760636
COLEC11	Diagnoses - main ICD10: R39.8 Other and unspecified symptoms and signs involving the urinary system	R39.8	0.999 (0.999, 1.000)	0.059575898
COLEC11	Diagnoses - main ICD10: R10.3 Pain localised to other parts of lower abdomen	R10.3	1.000 (0.999, 1.001)	0.937803711
COLEC11	Diagnoses - secondary ICD10: E78.5 Hyperlipidaemia, unspecified	E78.5	1.000 (0.999, 1.000)	0.416673155
COLEC11	Diagnoses - secondary ICD10: M81.99 Osteoporosis, unspecified (Site unspecified)	M81.99	1.000 (1.000, 1.001)	0.472799663
COLEC11	Diagnoses - secondary ICD10: Y83.8 Other surgical procedures	Y83.8	1.001 (1.000, 1.001)	0.179130615
COLEC11	Diagnoses - secondary ICD10: K63.5 Polyp of colon	K63.5	0.999 (0.999, 1.000)	0.07583259
COLEC11	Diagnoses - secondary ICD10: K59.0 Constipation	K59.0	1.000 (1.000, 1.001)	0.333863743
COLEC11	Diagnoses - main ICD10: M51.1 Lumbar and other intervertebral disk disorders with radiculopathy	M51.1	1.000 (0.999, 1.001)	0.883252216
COLEC11	Diagnoses - main ICD10: N20.0 Calculus of kidney	N20.0	1.000 (1.000, 1.001)	0.164430483
COLEC11	Diagnoses - main ICD10: M17.9 Gonarthrosis, unspecified	M17.9	1.000 (0.999, 1.001)	0.481871376
COLEC11	Diagnoses - main ICD10: N20.1 Calculus of ureter	N20.1	1.000 (1.000, 1.001)	0.075609474

COLEC11	Type of cancer: ICD10: C50.9 Breast, unspecified	C50.9	1.001 (1.000, 1.001)	0.269960564
COLEC11	Diagnoses - main ICD10: N84.1 Polyp of cervix uteri	N84.1	1.000 (0.999, 1.000)	0.735957224
COLEC11	Diagnoses - secondary ICD10: R33 Retention of urine	R33	1.000 (0.999, 1.001)	0.91565561
COLEC11	Diagnoses - main ICD10: K51.9 Ulcerative colitis, unspecified	K51.9	1.000 (1.000, 1.001)	0.55937696
COLEC11	Diagnoses - secondary ICD10: R06.0 Dyspnoea	R06.0	1.000 (0.999, 1.000)	0.723746855
COLEC11	Diagnoses - secondary ICD10: R10.4 Other and unspecified abdominal pain	R10.4	0.999 (0.998, 1.000)	0.060616324
COLEC11	Diagnoses - main ICD10: Z47.0 Follow-up care involving removal of fracture plate and other internal fixation dev	Z47.0	1.000 (1.000, 1.001)	0.166821556
COLEC11	Diagnoses - main ICD10: R51 Headache	R51	0.999 (0.999, 1.000)	0.16624487
COLEC11	Diagnoses - main ICD10: K62.1 Rectal polyp	K62.1	1.000 (0.999, 1.000)	0.143330417
COLEC11	Diagnoses - main ICD10: M16.9 Coxarthrosis, unspecified	M16.9	1.000 (0.999, 1.001)	0.982707582
COLEC11	Diagnoses - secondary ICD10: F32.9 Depressive episode, unspecified	F32.9	1.001 (1.000, 1.002)	0.136195291
COLEC11	Diagnoses - secondary ICD10: Z85.3 Personal history of malignant neoplasm of breast	Z85.3	1.000 (0.999, 1.001)	0.897607511
COLEC11	Diagnoses - main ICD10: K52.9 Non-infective gastro-enteritis and colitis, unspecified	K52.9	1.000 (0.999, 1.001)	0.818700641
COLEC11	Diagnoses - main ICD10: L98.9 Disorder of skin and subcutaneous tissue, unspecified	L98.9	1.000 (0.999, 1.001)	0.692368965
COLEC11	Diagnoses - secondary ICD10: M19.99 Arthrosis, unspecified (Site unspecified)	M19.99	1.000 (1.000, 1.001)	0.315193014
COLEC11	Diagnoses - main ICD10: Z12.1 Special screening examination for neoplasm of intestinal tract	Z12.1	1.000 (0.999, 1.000)	0.280226631
COLEC11	Diagnoses - main ICD10: R07.2 Precordial pain	R07.2	1.000 (0.999, 1.000)	0.672004231
COLEC11	Diagnoses - secondary ICD10: Z90.7 Acquired absence of genital organ(s)	Z90.7	1.000 (0.999, 1.000)	0.322365034
COLEC11	Diagnoses - secondary ICD10: Z96.6 Presence of orthopaedic joint implants	Z96.6	1.001 (1.000, 1.002)	0.128173723
COLEC11	Diagnoses - secondary ICD10: B96.8 Other specified bacterial agents as the cause of diseases classified to other cha	B96.8	1.000 (0.999, 1.001)	0.626849834
COLEC11	Diagnoses - secondary ICD10: Z82.4 Family history of ischaemic heart disease and other diseases of the circulatory	Z82.4	1.001 (1.000, 1.002)	0.053873959
COLEC11	Diagnoses - main ICD10: D17.1 Benign lipomatous neoplasm of skin and subcutaneous tissue of trunk	D17.1	1.000 (0.999, 1.000)	0.895465543
COLEC11	Diagnoses - main ICD10: S52.50 Fracture of lower end of radius (closed)	S52.50	1.000 (0.999, 1.000)	0.469215873
COLEC11	Diagnoses - secondary ICD10: W19.9 Unspecified place	W19.9	1.001 (1.000, 1.001)	0.024604362
COLEC11	Diagnoses - main ICD10: M16.1 Other primary coxarthrosis	M16.1	1.000 (0.999, 1.000)	0.50156356
COLEC11	Diagnoses - secondary ICD10: Z60.2 Living alone	Z60.2	1.000 (0.999, 1.000)	0.326388342
COLEC11	Diagnoses - main ICD10: H25.1 Senile nuclear cataract	H25.1	1.001 (1.000, 1.001)	0.018876
COLEC11	Diagnoses - main ICD10: D50.9 Iron deficiency anaemia, unspecified	D50.9	1.000 (1.000, 1.001)	0.130219352
COLEC11	Diagnoses - secondary ICD10: N81.6 Rectocele	N81.6	1.000 (1.000, 1.001)	0.115662165
COLEC11	Diagnoses - main ICD10: I21.9 Acute myocardial infarction, unspecified	I21.9	0.999 (0.999, 1.000)	0.033186976
COLEC11	Diagnoses - secondary ICD10: K44.9 Diaphragmatic hernia without obstruction or gangrene	K44.9	1.000 (0.999, 1.001)	0.985322528
COLEC11	Diagnoses - main ICD10: N32.8 Other specified disorders of bladder	N32.8	1.000 (0.999, 1.000)	0.649505979
COLEC11	Diagnoses - main ICD10: J34.2 Deviated nasal septum	J34.2	1.000 (0.999, 1.000)	0.435100152
COLEC11	Diagnoses - secondary ICD10: Z86.0 Personal history of other neoplasms	Z86.0	1.000 (0.999, 1.001)	0.626022053
COLEC11	Diagnoses - secondary ICD10: Z72.1 Alcohol use	Z72.1	1.000 (0.999, 1.001)	0.700728528
COLEC11	Diagnoses - main ICD10: G56.0 Carpal tunnel syndrome	G56.0	1.000 (0.999, 1.001)	0.544903213
COLEC11	Diagnoses - secondary ICD10: Z37.0 Single live birth	Z37.0	1.000 (0.999, 1.002)	0.616104203
COLEC11	Diagnoses - secondary ICD10: E03.9 Hypothyroidism, unspecified	E03.9	1.001 (0.999, 1.002)	0.308147846
COLEC11	Diagnoses - secondary ICD10: Z88.6 Personal history of allergy to analgesic agent	Z88.6	1.000 (0.999, 1.000)	0.398359031
COLEC11	Diagnoses - secondary ICD10: Z88.8 Personal history of allergy to other drugs, medicaments and biological substa	Z88.8	1.000 (0.999, 1.000)	0.506620075

COLEC11	Type of cancer: ICD10: D05.1 Intraductal carcinoma in situ	D05.1	1.001 (1.000, 1.001)	0.032145724
COLEC11	Diagnoses - main ICD10: K02.9 Dental caries, unspecified	K02.9	1.000 (1.000, 1.001)	0.680525176
COLEC11	Diagnoses - main ICD10: L82 Seborrhoeic keratosis	L82	1.000 (0.999, 1.000)	0.42161144
COLEC11	Diagnoses - secondary ICD10: Z72.0 Tobacco use	Z72.0	1.000 (0.999, 1.002)	0.504614251
COLEC11	Diagnoses - main ICD10: K40.9 Unilateral or unspecified inguinal hernia, without obstruction or gangrene	K40.9	1.000 (0.999, 1.001)	0.901760961
COLEC11	Diagnoses - secondary ICD10: Z86.6 Personal history of diseases of the nervous system and sense organs	Z86.6	1.000 (0.999, 1.001)	0.920629831
COLEC11	Diagnoses - secondary ICD10: Z88.0 Personal history of allergy to penicillin	Z88.0	1.000 (0.999, 1.001)	0.747994345
COLEC11	Diagnoses - main ICD10: M20.1 Hallux valgus (acquired)	M20.1	0.999 (0.998, 1.000)	0.084602538
COLEC11	Diagnoses - secondary ICD10: N39.0 Urinary tract infection, site not specified	N39.0	1.000 (0.999, 1.001)	0.775856164
COLEC11	Diagnoses - secondary ICD10: Z03.4 Observation for suspected myocardial infarction	Z03.4	1.000 (1.000, 1.001)	0.257220793
COLEC11	Diagnoses - secondary ICD10: D25.9 Leiomyoma of uterus, unspecified	D25.9	1.000 (1.000, 1.001)	0.303441049
COLEC11	Diagnoses - main ICD10: K43.9 Ventral hernia without obstruction or gangrene	K43.9	1.000 (0.999, 1.000)	0.796125102
COLEC11	Diagnoses - main ICD10: R13 Dysphagia	R13	1.000 (1.000, 1.001)	0.222827519
COLEC11	Diagnoses - main ICD10: M23.23 Derangement of meniscus due to old tear or injury (Medial collateral ligament o	M23.23	1.000 (0.999, 1.001)	0.747225082
COLEC11	Diagnoses - secondary ICD10: Z51.2 Other chemotherapy	Z51.2	1.000 (0.999, 1.000)	0.197532535
COLEC11	Diagnoses - secondary ICD10: Z85.5 Personal history of malignant neoplasm of urinary tract	Z85.5	1.000 (1.000, 1.001)	0.236494918
COLEC11	Diagnoses - secondary ICD10: Z92.1 Personal history of long-term (current) use of anticoagulants	Z92.1	0.999 (0.998, 1.000)	0.091659476
COLEC11	Diagnoses - main ICD10: Z30.2 Sterilisation	Z30.2	1.000 (0.999, 1.001)	0.792165802
COLEC11	Diagnoses - main ICD10: I84.8 Unspecified haemorrhoids with other complications	I84.8	1.000 (1.000, 1.001)	0.494454349
COLEC11	Diagnoses - main ICD10: Z46.6 Fitting and adjustment of urinary device	Z46.6	1.000 (0.999, 1.000)	0.138231013
COLEC11	Diagnoses - secondary ICD10: Y83.6 Removal of other organ (partial) (total)	Y83.6	1.000 (1.000, 1.001)	0.556584692
COLEC11	Type of cancer: ICD10: C44.9 Malignant neoplasm of skin, unspecified	C44.9	1.000 (1.000, 1.001)	0.474791421
COLEC11	Diagnoses - main ICD10: K80.1 Calculus of gallbladder with other cholecystitis	K80.1	1.000 (0.999, 1.001)	0.860116114
COLEC11	Diagnoses - secondary ICD10: Z53.0 Procedure not carried out because of contraindication	Z53.0	0.999 (0.998, 1.000)	0.090656421
COLEC11	Diagnoses - secondary ICD10: Z53.8 Procedure not carried out for other reasons	Z53.8	1.000 (0.999, 1.002)	0.513596921
COLEC11	Diagnoses - main ICD10: N35.9 Urethral stricture, unspecified	N35.9	0.999 (0.999, 1.000)	0.044666161
COLEC11	Type of cancer: ICD10: D06.9 Cervix, unspecified	D06.9	1.000 (0.999, 1.000)	0.151439834
COLEC11	Diagnoses - main ICD10: N92.1 Excessive and frequent menstruation with irregular cycle	N92.1	1.000 (1.000, 1.001)	0.43227917
COLEC11	Diagnoses - main ICD10: M23.22 Derangement of meniscus due to old tear or injury (Posterior cruciate ligament c	M23.22	1.000 (0.999, 1.000)	0.698842994
COLEC11	Diagnoses - secondary ICD10: E11.9 Without complications	E11.9	1.001 (0.999, 1.002)	0.445462247
COLEC11	Diagnoses - secondary ICD10: Z88.1 Personal history of allergy to other antibiotic agents	Z88.1	1.000 (1.000, 1.001)	0.24554528
COLEC11	Diagnoses - secondary ICD10: J90 Pleural effusion, not elsewhere classified	J90	1.000 (1.000, 1.001)	0.496769545
COLEC11	Underlying (primary) cause of death: ICD10: C34.9 Bronchus or lung, unspecified	C34.9	1.010 (0.991, 1.029)	0.322740687
COLEC11	Diagnoses - main ICD10: A41 Other septicaemia	A41	0.999 (0.999, 1.000)	0.048148145
COLEC11	Diagnoses - main ICD10: C18 Malignant neoplasm of colon	C18	0.999 (0.999, 1.000)	0.115462738
COLEC11	Diagnoses - main ICD10: C34 Malignant neoplasm of bronchus and lung	C34	1.000 (0.999, 1.001)	0.941624838
COLEC11	Diagnoses - main ICD10: C43 Malignant melanoma of skin	C43	1.000 (0.999, 1.001)	0.74049056
COLEC11	Diagnoses - main ICD10: C67 Malignant neoplasm of bladder	C67	1.000 (0.999, 1.001)	0.872067193
COLEC11	Diagnoses - main ICD10: C78 Secondary malignant neoplasm of respiratory and digestive organs	C78	1.000 (0.999, 1.001)	0.936573983
COLEC11	Diagnoses - main ICD10: C79 Secondary malignant neoplasm of other sites	C79	1.000 (0.999, 1.001)	0.912332578

COLEC11	Diagnoses - main ICD10: D05 Carcinoma in situ of breast	D05	1.000 (0.999, 1.001)	0.8571532
COLEC11	Diagnoses - main ICD10: D17 Benign lipomatous neoplasm	D17	1.000 (0.999, 1.001)	0.850236211
COLEC11	Diagnoses - main ICD10: D22 Melanocytic naevi	D22	0.999 (0.999, 1.000)	0.255321673
COLEC11	Diagnoses - main ICD10: D23 Other benign neoplasms of skin	D23	1.000 (0.999, 1.001)	0.851704784
COLEC11	Diagnoses - main ICD10: D50 Iron deficiency anaemia	D50	1.001 (1.000, 1.001)	0.286921386
COLEC11	Diagnoses - main ICD10: D64 Other anaemias	D64	1.000 (0.999, 1.001)	0.862273626
COLEC11	Diagnoses - main ICD10: G43 Migraine	G43	1.000 (1.000, 1.001)	0.635606296
COLEC11	Diagnoses - main ICD10: G45 Transient cerebral ischaemic attacks and related syndromes	G45	1.000 (0.999, 1.000)	0.442328585
COLEC11	Diagnoses - main ICD10: G57 Mononeuropathies of lower limb	G57	1.000 (0.999, 1.000)	0.13384546
COLEC11	Diagnoses - main ICD10: H00 Hordeolum and chalazion	H00	1.000 (1.000, 1.001)	0.29962534
COLEC11	Diagnoses - main ICD10: H02 Other disorders of eyelid	H02	1.000 (0.999, 1.001)	0.682173797
COLEC11	Diagnoses - main ICD10: H04 Disorders of lachrymal system	H04	1.000 (1.000, 1.001)	0.169743073
COLEC11	Diagnoses - main ICD10: H33 Retinal detachments and breaks	H33	1.000 (0.999, 1.001)	0.49816737
COLEC11	Diagnoses - main ICD10: H35 Other retinal disorders	H35	1.001 (1.000, 1.001)	0.063808372
COLEC11	Diagnoses - main ICD10: H40 Glaucoma	H40	1.000 (0.999, 1.001)	0.864321565
COLEC11	Diagnoses - main ICD10: I26 Pulmonary embolism	I26	1.000 (0.999, 1.001)	0.574115081
COLEC11	Diagnoses - main ICD10: I47 Paroxysmal tachycardia	I47	0.999 (0.999, 1.000)	0.076194054
COLEC11	Diagnoses - main ICD10: I50 Heart failure	I50	1.000 (1.000, 1.001)	0.275102631
COLEC11	Diagnoses - main ICD10: I63 Cerebral infarction	I63	1.000 (0.999, 1.001)	0.993673067
COLEC11	Diagnoses - main ICD10: J18 Pneumonia, organism unspecified	J18	1.000 (0.999, 1.001)	0.591880826
COLEC11	Diagnoses - main ICD10: J32 Chronic sinusitis	J32	1.000 (1.000, 1.001)	0.407347229
COLEC11	Diagnoses - main ICD10: J38 Diseases of vocal cords and larynx, not elsewhere classified	J38	1.000 (0.999, 1.001)	0.951503238
COLEC11	Diagnoses - main ICD10: J45 Asthma	J45	1.000 (0.999, 1.000)	0.334285105
COLEC11	Diagnoses - main ICD10: K01 Embedded and impacted teeth	K01	1.000 (0.999, 1.000)	0.355327302
COLEC11	Diagnoses - main ICD10: K02 Dental caries	K02	1.000 (1.000, 1.001)	0.46728926
COLEC11	Diagnoses - main ICD10: K04 Diseases of pulp and periapical tissues	K04	1.000 (0.999, 1.000)	0.170180388
COLEC11	Diagnoses - main ICD10: K08 Other disorders of teeth and supporting structures	K08	1.000 (1.000, 1.001)	0.224423982
COLEC11	Diagnoses - main ICD10: K13 Other diseases of lip and oral mucosa	K13	1.000 (0.999, 1.000)	0.39818774
COLEC11	Diagnoses - main ICD10: K25 Gastric ulcer	K25	1.000 (0.999, 1.000)	0.489565785
COLEC11	Diagnoses - main ICD10: K26 Duodenal ulcer	K26	1.000 (1.000, 1.001)	0.53468756
COLEC11	Diagnoses - main ICD10: K31 Other diseases of stomach and duodenum	K31	1.000 (1.000, 1.001)	0.326515381
COLEC11	Diagnoses - main ICD10: K42 Umbilical hernia	K42	1.000 (0.999, 1.001)	0.49307075
COLEC11	Diagnoses - main ICD10: K56 Paralytic ileus and intestinal obstruction without hernia	K56	0.999 (0.998, 1.000)	0.023065242
COLEC11	Diagnoses - main ICD10: K58 Irritable bowel syndrome	K58	1.000 (0.999, 1.000)	0.784493887
COLEC11	Diagnoses - main ICD10: K59 Other functional intestinal disorders	K59	1.000 (0.999, 1.001)	0.847090254
COLEC11	Diagnoses - main ICD10: K61 Abscess of anal and rectal regions	K61	1.000 (0.999, 1.000)	0.246462102
COLEC11	Diagnoses - main ICD10: K63 Other diseases of intestine	K63	1.000 (0.999, 1.001)	0.949024792
COLEC11	Diagnoses - main ICD10: K81 Cholecystitis	K81	1.000 (0.999, 1.001)	0.589657971
COLEC11	Diagnoses - main ICD10: K92 Other diseases of digestive system	K92	1.000 (0.999, 1.001)	0.614146929
COLEC11	Diagnoses - main ICD10: L02 Cutaneous abscess, furuncle and carbuncle	L02	1.000 (1.000, 1.001)	0.242411988

COLEC11	Diagnoses - main ICD10: L57 Skin changes due to chronic exposure to nonionising radiation	L57	1.000 (1.000, 1.001)	0.247086381
COLEC11	Diagnoses - main ICD10: L72 Follicular cysts of skin and subcutaneous tissue	L72	1.000 (0.999, 1.002)	0.645759311
COLEC11	Diagnoses - main ICD10: L90 Atrophic disorders of skin	L90	1.000 (0.999, 1.001)	0.992520887
COLEC11	Diagnoses - main ICD10: L98 Other disorders of skin and subcutaneous tissue, not elsewhere classified	L98	1.000 (0.999, 1.001)	0.84970395
COLEC11	Diagnoses - main ICD10: M06 Other rheumatoid arthritis	M06	1.000 (0.999, 1.000)	0.234603709
COLEC11	Diagnoses - main ICD10: M13 Other arthritis	M13	1.000 (1.000, 1.001)	0.114632119
COLEC11	Diagnoses - main ICD10: M15 Polyarthrosis	M15	1.000 (1.000, 1.001)	0.39308514
COLEC11	Diagnoses - main ICD10: M19 Other arthrosis	M19	1.000 (0.999, 1.001)	0.893814406
COLEC11	Diagnoses - main ICD10: M47 Spondylosis	M47	1.000 (1.000, 1.001)	0.421946838
COLEC11	Diagnoses - main ICD10: M48 Other spondylopathies	M48	1.000 (0.999, 1.001)	0.975562679
COLEC11	Diagnoses - main ICD10: M51 Other intervertebral disk disorders	M51	1.000 (0.999, 1.001)	0.535034834
COLEC11	Diagnoses - main ICD10: M65 Synovitis and tenosynovitis	M65	1.000 (0.999, 1.001)	0.734889319
COLEC11	Diagnoses - main ICD10: M75 Shoulder lesions	M75	0.999 (0.998, 1.001)	0.414745
COLEC11	Diagnoses - main ICD10: M79 Other soft tissue disorders, not elsewhere classified	M79	1.000 (0.999, 1.001)	0.822092883
COLEC11	Diagnoses - main ICD10: M84 Disorders of continuity of bone	M84	1.000 (1.000, 1.001)	0.254821287
COLEC11	Diagnoses - main ICD10: N13 Obstructive and reflux uropathy	N13	1.000 (0.999, 1.000)	0.467922851
COLEC11	Diagnoses - main ICD10: N30 Cystitis	N30	1.000 (0.999, 1.001)	0.742704319
COLEC11	Diagnoses - main ICD10: N31 Neuromuscular dysfunction of bladder, not elsewhere classified	N31	1.000 (1.000, 1.001)	0.539603006
COLEC11	Diagnoses - main ICD10: N35 Urethral stricture	N35	0.999 (0.999, 1.000)	0.050296138
COLEC11	Diagnoses - main ICD10: N39 Other disorders of urinary system	N39	0.999 (0.998, 1.001)	0.519329776
COLEC11	Diagnoses - main ICD10: N48 Other disorders of penis	N48	1.000 (0.999, 1.000)	0.595367776
COLEC11	Diagnoses - main ICD10: N50 Other disorders of male genital organs	N50	1.000 (1.000, 1.001)	0.432885992
COLEC11	Diagnoses - main ICD10: N60 Benign mammary dysplasia	N60	1.000 (1.000, 1.001)	0.253439405
COLEC11	Diagnoses - main ICD10: N80 Endometriosis	N80	1.000 (0.999, 1.000)	0.377840661
COLEC11	Diagnoses - main ICD10: N83 Noninflammatory disorders of ovary, Fallopian tube and broad ligament	N83	1.000 (1.000, 1.001)	0.459238262
COLEC11	Diagnoses - main ICD10: N84 Polyp of female genital tract	N84	0.999 (0.998, 1.000)	0.199723893
COLEC11	Diagnoses - main ICD10: N85 Other noninflammatory disorders of uterus, except cervix	N85	1.000 (0.999, 1.000)	0.690877773
COLEC11	Diagnoses - main ICD10: N87 Dysplasia of cervix uteri	N87	1.000 (1.000, 1.001)	0.645086615
COLEC11	Diagnoses - main ICD10: N90 Other noninflammatory disorders of vulva and perineum	N90	1.000 (1.000, 1.001)	0.802995992
COLEC11	Diagnoses - main ICD10: N93 Other abnormal uterine and vaginal bleeding	N93	1.000 (1.000, 1.001)	0.437035264
COLEC11	Diagnoses - main ICD10: N94 Pain and other conditions associated with female genital organs and menstrual cycle	N94	1.000 (1.000, 1.001)	0.171731726
COLEC11	Diagnoses - main ICD10: N95 Menopausal and other perimenopausal disorders	N95	1.000 (0.999, 1.001)	0.825000075
COLEC11	Diagnoses - main ICD10: O02 Other abnormal products of conception	O02	1.000 (0.999, 1.000)	0.549105882
COLEC11	Diagnoses - main ICD10: O03 Spontaneous abortion	O03	1.000 (0.999, 1.000)	0.093165135
COLEC11	Diagnoses - main ICD10: O26 Maternal care for other conditions predominantly related to pregnancy	O26	1.000 (0.999, 1.000)	0.70350215
COLEC11	Diagnoses - main ICD10: O36 Maternal care for other known or suspected foetal problems	O36	1.000 (0.999, 1.000)	0.392303621
COLEC11	Diagnoses - main ICD10: O63 Long labour	O63	1.001 (1.000, 1.001)	0.022701074
COLEC11	Diagnoses - main ICD10: O68 Labour and delivery complicated by foetal stress [distress]	O68	1.000 (0.999, 1.001)	0.694875951
COLEC11	Diagnoses - main ICD10: O70 Perineal laceration during delivery	O70	1.000 (1.000, 1.001)	0.351376382
COLEC11	Diagnoses - main ICD10: O80 Single spontaneous delivery	O80	1.000 (0.999, 1.001)	0.942007067

COLEC11	Diagnoses - main ICD10: R00 Abnormalities of heart beat	R00	1.000 (0.999, 1.001)	0.888210921
COLEC11	Diagnoses - main ICD10: R06 Abnormalities of breathing	R06	1.001 (1.000, 1.002)	0.037981801
COLEC11	Diagnoses - main ICD10: R19 Other symptoms and signs involving the digestive system and abdomen	R19	1.002 (1.001, 1.004)	0.004429453
COLEC11	Diagnoses - main ICD10: R22 Localised swelling, mass and lump of skin and subcutaneous tissue	R22	1.000 (0.999, 1.001)	0.867517042
COLEC11	Diagnoses - main ICD10: R39 Other symptoms and signs involving the urinary system	R39	0.999 (0.998, 1.000)	0.025877832
COLEC11	Diagnoses - main ICD10: R50 Fever of unknown origin	R50	1.000 (0.999, 1.000)	0.535624092
COLEC11	Diagnoses - main ICD10: R56 Convulsions, not elsewhere classified	R56	0.999 (0.999, 1.000)	0.01605819
COLEC11	Diagnoses - main ICD10: R59 Enlarged lymph nodes	R59	1.000 (0.999, 1.000)	0.284504724
COLEC11	Diagnoses - main ICD10: R63 Symptoms and signs concerning food and fluid intake	R63	1.000 (1.000, 1.001)	0.350893528
COLEC11	Diagnoses - main ICD10: R79 Other abnormal findings of blood chemistry	R79	1.000 (0.999, 1.000)	0.318491044
COLEC11	Diagnoses - main ICD10: R87 Abnormal findings in specimens from female genital organs	R87	1.000 (1.000, 1.001)	0.872203489
COLEC11	Diagnoses - main ICD10: R93 Abnormal findings on diagnostic imaging of other body structures	R93	1.000 (0.999, 1.000)	0.588827337
COLEC11	Diagnoses - main ICD10: S01 Open wound of head	S01	1.000 (0.999, 1.001)	0.741686507
COLEC11	Diagnoses - main ICD10: S02 Fracture of skull and facial bones	S02	1.000 (1.000, 1.001)	0.329383256
COLEC11	Diagnoses - main ICD10: S42 Fracture of shoulder and upper arm	S42	1.000 (1.000, 1.001)	0.285489387
COLEC11	Diagnoses - main ICD10: S61 Open wound of wrist and hand	S61	1.000 (0.999, 1.000)	0.583771777
COLEC11	Diagnoses - main ICD10: S62 Fracture at wrist and hand level	S62	1.000 (0.999, 1.001)	0.829665616
COLEC11	Diagnoses - main ICD10: S72 Fracture of femur	S72	1.001 (1.000, 1.001)	0.078596588
COLEC11	Diagnoses - main ICD10: S82 Fracture of lower leg, including ankle	S82	1.001 (1.000, 1.002)	0.027561032
COLEC11	Diagnoses - main ICD10: T39 Poisoning by nonopioid analgesics, antipyretics and antirheumatics	T39	1.000 (1.000, 1.001)	0.524618881
COLEC11	Diagnoses - main ICD10: T81 Complications of procedures, not elsewhere classified	T81	1.001 (1.000, 1.002)	0.138583941
COLEC11	Diagnoses - main ICD10: T82 Complications of cardiac and vascular prosthetic devices, implants and grafts	T82	1.000 (1.000, 1.001)	0.63951521
COLEC11	Diagnoses - main ICD10: T85 Complications of other internal prosthetic devices, implants and grafts	T85	1.000 (0.999, 1.000)	0.36766344
COLEC11	Diagnoses - main ICD10: Z01 Other special examinations and investigations of persons without complaint or repor	Z01	1.000 (0.999, 1.001)	0.958516318
COLEC11	Diagnoses - main ICD10: Z03 Medical observation and evaluation for suspected diseases and conditions	Z03	1.000 (0.999, 1.001)	0.51357032
COLEC11	Diagnoses - main ICD10: Z08 Follow-up examination after treatment for malignant neoplasm	Z08	1.000 (0.999, 1.001)	0.63055261
COLEC11	Diagnoses - main ICD10: Z12 Special screening examination for neoplasms	Z12	0.999 (0.998, 1.000)	0.160318159
COLEC11	Diagnoses - main ICD10: Z13 Special screening examination for other diseases and disorders	Z13	1.001 (1.000, 1.001)	0.079319316
COLEC11	Diagnoses - main ICD10: Z30 Contraceptive management	Z30	0.999 (0.998, 1.001)	0.402612527
COLEC11	Diagnoses - main ICD10: Z42 Follow-up care involving plastic surgery	Z42	1.000 (0.999, 1.000)	0.254323619
COLEC11	Diagnoses - main ICD10: Z43 Attention to artificial openings	Z43	1.000 (0.999, 1.000)	0.280953285
COLEC11	Diagnoses - main ICD10: Z45 Adjustment and management of implanted device	Z45	1.000 (0.999, 1.001)	0.74239072
COLEC11	Diagnoses - main ICD10: Z46 Fitting and adjustment of other devices	Z46	0.999 (0.998, 1.000)	0.046443645
COLEC11	Diagnoses - main ICD10: Z53 Persons encountering health services for specifie procedures, not carried out	Z53	1.000 (0.999, 1.000)	0.380137585

Table S8 Phenome-wide Mendelian Randomization analysis to reveal potential on-target side effects of IRF3

protein	disease	ICD-10 code	OR (95%CI)	P value
IRF3	Underlying (primary) cause of death: ICD10: E85.4 Organ-limited amyloidosis	E85.4	0.998 (0.996, 1.001)	0.130295787
IRF3	Underlying (primary) cause of death: ICD10: J84.1 Other interstitial pulmonary diseases with fibrosis	J84.1	1.006 (0.998, 1.014)	0.166644197
IRF3	Diagnoses - main ICD10: B37 Candidiasis	B37	1.000 (1.000, 1.000)	0.282372646
IRF3	Diagnoses - main ICD10: C44 Other malignant neoplasms of skin	C44	0.997 (0.996, 0.998)	9.00678E-06
IRF3	Diagnoses - main ICD10: C50 Malignant neoplasm of breast	C50	1.000 (0.999, 1.001)	0.982186586
IRF3	Diagnoses - main ICD10: C61 Malignant neoplasm of prostate	C61	1.000 (0.999, 1.001)	0.540316665
IRF3	Diagnoses - main ICD10: D12 Benign neoplasm of colon rectum anus and anal canal	D12	1.000 (0.998, 1.001)	0.634283884
IRF3	Diagnoses - main ICD10: D25 Leiomyoma of uterus	D25	1.000 (0.999, 1.002)	0.489580224
IRF3	Diagnoses - main ICD10: E03 Other hypothyroidism	E03	1.000 (1.000, 1.000)	0.463449863
IRF3	Diagnoses - main ICD10: E04 Other non-toxic goitre	E04	1.000 (1.000, 1.001)	0.692194477
IRF3	Diagnoses - main ICD10: F31 Bipolar affective disorder	F31	1.000 (1.000, 1.000)	0.487282069
IRF3	Diagnoses - main ICD10: F43 Reaction to severe stress and adjustment disorders	F43	1.000 (1.000, 1.000)	0.701835912
IRF3	Diagnoses - main ICD10: G47 Sleep disorders	G47	1.000 (0.999, 1.001)	0.984783274
IRF3	Diagnoses - main ICD10: G56 Mononeuropathies of upper limb	G56	1.000 (0.999, 1.002)	0.856680457
IRF3	Diagnoses - main ICD10: H25 Senile cataract	H25	1.000 (0.999, 1.001)	0.61989626
IRF3	Diagnoses - main ICD10: H26 Other cataract	H26	1.000 (0.998, 1.001)	0.607770859
IRF3	Diagnoses - main ICD10: I10 Essential (primary) hypertension	I10	1.000 (1.000, 1.000)	0.815562844
IRF3	Diagnoses - main ICD10: I20 Angina pectoris	I20	0.999 (0.998, 1.001)	0.441042399
IRF3	Diagnoses - main ICD10: I21 Acute myocardial infarction	I21	1.000 (0.999, 1.002)	0.467980295
IRF3	Diagnoses - main ICD10: I25 Chronic ischaemic heart disease	I25	1.002 (1.000, 1.004)	0.04422428
IRF3	Diagnoses - main ICD10: I30 Acute pericarditis	I30	1.000 (1.000, 1.000)	0.810452036
IRF3	Diagnoses - main ICD10: I48 Atrial fibrillation and flutter	I48	1.000 (0.999, 1.001)	0.826405699
IRF3	Diagnoses - main ICD10: I80 Phlebitis and thrombophlebitis	I80	1.000 (0.999, 1.001)	0.628472567
IRF3	Diagnoses - main ICD10: I84 Haemorrhoids	I84	1.001 (0.999, 1.002)	0.540619449
IRF3	Diagnoses - main ICD10: J22 Unspecified acute lower respiratory infection	J22	1.000 (0.999, 1.001)	0.593475031
IRF3	Diagnoses - main ICD10: J33 Nasal polyp	J33	1.000 (0.999, 1.001)	0.872728397
IRF3	Diagnoses - main ICD10: J34 Other disorders of nose and nasal sinuses	J34	1.001 (1.000, 1.002)	0.214866489
IRF3	Diagnoses - main ICD10: J44 Other chronic obstructive pulmonary disease	J44	1.000 (0.999, 1.000)	0.174795616
IRF3	Diagnoses - main ICD10: K20 Oesophagitis	K20	1.001 (0.999, 1.002)	0.321539308
IRF3	Diagnoses - main ICD10: K21 Gastro-oesophageal reflux disease	K21	0.999 (0.998, 1.001)	0.331958756
IRF3	Diagnoses - main ICD10: K22 Other diseases of oesophagus	K22	1.001 (0.999, 1.002)	0.297069169
IRF3	Diagnoses - main ICD10: K29 Gastritis and duodenitis	K29	1.000 (0.999, 1.002)	0.82944192
IRF3	Diagnoses - main ICD10: K35 Acute appendicitis	K35	1.001 (1.000, 1.002)	0.001750729
IRF3	Diagnoses - main ICD10: K40 Inguinal hernia	K40	1.000 (0.999, 1.002)	0.696066859
IRF3	Diagnoses - main ICD10: K43 Ventral hernia	K43	1.000 (1.000, 1.001)	0.287793361
IRF3	Diagnoses - main ICD10: K44 Diaphragmatic hernia	K44	1.000 (0.999, 1.002)	0.759983924
IRF3	Diagnoses - main ICD10: K50 Crohn's disease [regional enteritis]	K50	1.000 (0.999, 1.000)	0.456015457
IRF3	Diagnoses - main ICD10: K51 Ulcerative colitis	K51	0.999 (0.998, 1.000)	0.012959602

IRF3	Diagnoses - main ICD10: K52 Other non-infective gastro-enteritis and colitis	K52	1.000 (0.998, 1.001)	0.558872293
IRF3	Diagnoses - main ICD10: K57 Diverticular disease of intestine	K57	1.002 (1.001, 1.004)	0.001627792
IRF3	Diagnoses - main ICD10: K60 Fissure and fistula of anal and rectal regions	K60	1.000 (0.999, 1.000)	0.425009369
IRF3	Diagnoses - main ICD10: K62 Other diseases of anus and rectum	K62	0.999 (0.998, 1.001)	0.554393612
IRF3	Diagnoses - main ICD10: K76 Other diseases of liver	K76	1.000 (1.000, 1.000)	0.968704422
IRF3	Diagnoses - main ICD10: K80 Cholelithiasis	K80	0.999 (0.997, 1.000)	0.103052066
IRF3	Diagnoses - main ICD10: L03 Cellulitis	L03	1.000 (0.999, 1.001)	0.601613071
IRF3	Diagnoses - main ICD10: M10 Gout	M10	1.000 (1.000, 1.000)	0.290386714
IRF3	Diagnoses - main ICD10: M16 Coxarthrosis [arthrosis of hip]	M16	1.001 (0.999, 1.002)	0.350542737
IRF3	Diagnoses - main ICD10: M17 Gonarthrosis [arthrosis of knee]	M17	1.001 (0.999, 1.002)	0.328025401
IRF3	Diagnoses - main ICD10: M20 Acquired deformities of fingers and toes	M20	0.999 (0.998, 1.000)	0.090660208
IRF3	Diagnoses - main ICD10: M21 Other acquired deformities of limbs	M21	1.000 (1.000, 1.000)	0.913990202
IRF3	Diagnoses - main ICD10: M23 Internal derangement of knee	M23	1.001 (1.000, 1.003)	0.156984033
IRF3	Diagnoses - main ICD10: M24 Other specific joint derangements	M24	1.000 (0.999, 1.000)	0.740816584
IRF3	Diagnoses - main ICD10: M25 Other joint disorders not elsewhere classified	M25	1.001 (0.999, 1.002)	0.246554115
IRF3	Diagnoses - main ICD10: M54 Dorsalgia	M54	0.999 (0.998, 1.001)	0.338591469
IRF3	Diagnoses - main ICD10: M67 Other disorders of synovium and tendon	M67	1.000 (0.999, 1.001)	0.899542859
IRF3	Diagnoses - main ICD10: M70 Soft tissue disorders related to use overuse and pressure	M70	1.000 (1.000, 1.001)	0.857118689
IRF3	Diagnoses - main ICD10: M72 Fibroblastic disorders	M72	1.000 (0.999, 1.001)	0.767858596
IRF3	Diagnoses - main ICD10: N19 Unspecified renal failure	N19	1.000 (1.000, 1.000)	0.310695275
IRF3	Diagnoses - main ICD10: N20 Calculus of kidney and ureter	N20	1.000 (0.999, 1.001)	0.556382893
IRF3	Diagnoses - main ICD10: N32 Other disorders of bladder	N32	1.000 (0.999, 1.000)	0.333498305
IRF3	Diagnoses - main ICD10: N40 Hyperplasia of prostate	N40	1.000 (0.999, 1.001)	0.493600051
IRF3	Diagnoses - main ICD10: N81 Female genital prolapse	N81	1.000 (0.999, 1.002)	0.693602829
IRF3	Diagnoses - main ICD10: N92 Excessive frequent and irregular menstruation	N92	1.001 (1.000, 1.003)	0.13603212
IRF3	Diagnoses - main ICD10: O75 Other complications of labour and delivery not elsewhere classified	O75	1.000 (1.000, 1.000)	0.590332227
IRF3	Diagnoses - main ICD10: R04 Haemorrhage from respiratory passages	R04	1.000 (0.999, 1.000)	0.222037927
IRF3	Diagnoses - main ICD10: R07 Pain in throat and chest	R07	0.999 (0.997, 1.002)	0.669520958
IRF3	Diagnoses - main ICD10: R10 Abdominal and pelvic pain	R10	1.001 (0.999, 1.004)	0.239023032
IRF3	Diagnoses - main ICD10: R11 Nausea and vomiting	R11	1.000 (0.999, 1.001)	0.586021401
IRF3	Diagnoses - main ICD10: R14 Flatulence and related conditions	R14	1.000 (1.000, 1.000)	0.334069565
IRF3	Diagnoses - main ICD10: R31 Unspecified haematuria	R31	0.999 (0.998, 1.001)	0.301827057
IRF3	Diagnoses - main ICD10: R35 Polyuria	R35	1.000 (0.999, 1.000)	0.48329854
IRF3	Diagnoses - main ICD10: R55 Syncope and collapse	R55	1.000 (0.999, 1.001)	0.470063891
IRF3	Diagnoses - main ICD10: R69 Unknown and unspecified causes of morbidity	R69	0.999 (0.998, 1.001)	0.479089488
IRF3	Diagnoses - main ICD10: S09 Other and unspecified injuries of head	S09	1.000 (0.999, 1.000)	0.284016765
IRF3	Diagnoses - main ICD10: S52 Fracture of forearm	S52	1.000 (0.999, 1.001)	0.996091711
IRF3	Diagnoses - main ICD10: S66 Injury of muscle and tendon at wrist and hand level	S66	1.001 (1.000, 1.001)	0.005476009
IRF3	Diagnoses - main ICD10: S76 Injury of muscle and tendon at hip and thigh level	S76	1.000 (1.000, 1.000)	0.342650246
IRF3	Diagnoses - main ICD10: T84 Complications of internal orthopaedic prosthetic devices implants and grafts	T84	1.001 (1.001, 1.002)	0.00176411

IRF3	Diagnoses - main ICD10: Z09 Follow-up examination after treatment for conditions other than malignant neoplasms	Z09	1.000 (0.999, 1.001)	0.89193915
IRF3	Diagnoses - main ICD10: Z47 Other orthopaedic follow-up care	Z47	1.000 (0.999, 1.001)	0.667787392
IRF3	Diagnoses - main ICD10: Z80 Family history of malignant neoplasm	Z80	1.000 (1.000, 1.001)	0.239394058
IRF3	Diagnoses - secondary ICD10: Z50.1 Other physical therapy	Z50.1	1.000 (1.000, 1.001)	0.317123785
IRF3	Diagnoses - main ICD10: Z09.0 Follow-up examination after surgery for other conditions	Z09.0	1.000 (0.999, 1.001)	0.482653986
IRF3	Diagnoses - secondary ICD10: K21.0 Gastro-oesophageal reflux disease with oesophagitis	K21.0	1.000 (1.000, 1.001)	0.165669817
IRF3	Type of cancer: ICD10: C44.3 Skin of other and unspecified parts of face	C44.3	0.998 (0.997, 0.999)	0.001352511
IRF3	Diagnoses - main ICD10: K80.0 Calculus of gallbladder with acute cholecystitis	K80.0	1.000 (1.000, 1.000)	0.995858392
IRF3	Diagnoses - secondary ICD10: R19.4 Change in bowel habit	R19.4	1.000 (1.000, 1.001)	0.487663399
IRF3	Diagnoses - secondary ICD10: R10.1 Pain localised to upper abdomen	R10.1	1.000 (0.999, 1.001)	0.895460572
IRF3	Diagnoses - secondary ICD10: Z03.8 Observation for other suspected diseases and conditions	Z03.8	1.000 (0.999, 1.000)	0.647852535
IRF3	Diagnoses - secondary ICD10: I50.1 Left ventricular failure	I50.1	1.000 (1.000, 1.001)	0.234401928
IRF3	Diagnoses - main ICD10: D12.5 Sigmoid colon	D12.5	1.000 (0.999, 1.001)	0.622415867
IRF3	Diagnoses - main ICD10: Z09.9 Follow-up examination after unspecified treatment for other conditions	Z09.9	1.000 (0.999, 1.000)	0.458878438
IRF3	Diagnoses - main ICD10: N47 Redundant prepuce, phimosis and paraphimosis	N47	1.000 (1.000, 1.001)	0.658845934
IRF3	Diagnoses - secondary ICD10: E10.9 Without complications	E10.9	1.001 (1.000, 1.002)	0.001602775
IRF3	Diagnoses - main ICD10: O70.1 Second degree perineal laceration during delivery	O70.1	1.000 (0.999, 1.001)	0.847112084
IRF3	Diagnoses - secondary ICD10: I20.0 Unstable angina	I20.0	1.000 (0.999, 1.001)	0.92208994
IRF3	Diagnoses - main ICD10: R07.4 Chest pain, unspecified	R07.4	1.001 (0.999, 1.002)	0.500299318
IRF3	Diagnoses - main ICD10: N93.9 Abnormal uterine and vaginal bleeding, unspecified	N93.9	1.000 (0.999, 1.000)	0.725542527
IRF3	Diagnoses - main ICD10: K57.3 Diverticular disease of large intestine without perforation or abscess	K57.3	1.002 (1.001, 1.003)	0.001373208
IRF3	Diagnoses - main ICD10: M54.59 Low back pain (Site unspecified)	M54.59	1.000 (0.999, 1.000)	0.386907554
IRF3	Diagnoses - secondary ICD10: Z87.4 Personal history of diseases of the genito-urinary system	Z87.4	1.000 (0.999, 1.000)	0.363682923
IRF3	Diagnoses - secondary ICD10: Z92.2 Personal history of long-term (current) use of other medicaments	Z92.2	1.000 (0.999, 1.001)	0.77186667
IRF3	Diagnoses - main ICD10: K80.2 Calculus of gallbladder without cholecystitis	K80.2	1.000 (0.999, 1.001)	0.835428261
IRF3	Diagnoses - secondary ICD10: M13.9 Arthritis, unspecified	M13.9	1.000 (0.999, 1.000)	0.49443313
IRF3	Diagnoses - main ICD10: R79.8 Other specified abnormal findings of blood chemistry	R79.8	1.000 (0.999, 1.001)	0.916407027
IRF3	Diagnoses - secondary ICD10: Z95.1 Presence of aortocoronary bypass graft	Z95.1	1.001 (1.000, 1.002)	0.002893792
IRF3	Diagnoses - secondary ICD10: W01.0 Home	W01.0	1.001 (1.000, 1.001)	0.014047272
IRF3	Diagnoses - secondary ICD10: Z85.8 Personal history of malignant neoplasms of other organs and systems	Z85.8	0.999 (0.999, 1.000)	0.195796581
IRF3	Diagnoses - main ICD10: I84.9 Unspecified haemorrhoids without complication	I84.9	1.001 (1.000, 1.002)	0.156345263
IRF3	Diagnoses - main ICD10: K60.2 Anal fissure, unspecified	K60.2	1.000 (0.999, 1.000)	0.459011158
IRF3	Diagnoses - main ICD10: N81.1 Cystocele	N81.1	1.000 (0.999, 1.000)	0.200611959
IRF3	Diagnoses - secondary ICD10: F41.9 Anxiety disorder, unspecified	F41.9	1.000 (1.000, 1.001)	0.591382825
IRF3	Diagnoses - main ICD10: K40.2 Bilateral inguinal hernia, without obstruction or gangrene	K40.2	1.000 (1.000, 1.001)	0.627920046
IRF3	Diagnoses - main ICD10: L90.5 Scar conditions and fibrosis of skin	L90.5	1.000 (0.999, 1.000)	0.17568921
IRF3	Diagnoses - main ICD10: R04.0 Epistaxis	R04.0	1.000 (1.000, 1.001)	0.787686018
IRF3	Diagnoses - secondary ICD10: Z51.1 Chemotherapy session for neoplasm	Z51.1	1.000 (0.999, 1.001)	0.746610999
IRF3	Diagnoses - main ICD10: R32 Unspecified urinary incontinence	R32	1.000 (0.999, 1.000)	0.713979175
IRF3	Diagnoses - main ICD10: N92.0 Excessive and frequent menstruation with regular cycle	N92.0	1.001 (1.000, 1.002)	0.177064122

IRF3	Diagnoses - main ICD10: K62.5 Haemorrhage of anus and rectum	K62.5	1.001 (1.000, 1.002)	0.218820087
IRF3	Diagnoses - secondary ICD10: Z96.1 Presence of intraocular lens	Z96.1	1.000 (0.999, 1.001)	0.934359413
IRF3	Diagnoses - secondary ICD10: R00.1 Bradycardia, unspecified	R00.1	1.000 (1.000, 1.001)	0.902581309
IRF3	Diagnoses - secondary ICD10: K66.0 Peritoneal adhesions	K66.0	1.000 (1.000, 1.001)	0.293682432
IRF3	Diagnoses - main ICD10: T84.8 Other complications of internal orthopaedic prosthetic devices, implants and grafts	T84.8	1.000 (1.000, 1.001)	0.237933091
IRF3	Diagnoses - secondary ICD10: Z85.4 Personal history of malignant neoplasm of genital organs	Z85.4	1.000 (0.999, 1.000)	0.402436639
IRF3	Diagnoses - main ICD10: I47.1 Supraventricular tachycardia	I47.1	1.000 (0.999, 1.000)	0.800765752
IRF3	Diagnoses - secondary ICD10: F10.0 Acute intoxication	F10.0	1.000 (0.999, 1.000)	0.910009781
IRF3	Diagnoses - main ICD10: K08.3 Retained dental root	K08.3	1.000 (1.000, 1.000)	0.87854621
IRF3	Diagnoses - secondary ICD10: M06.99 Rheumatoid arthritis, unspecified (Site unspecified)	M06.99	1.000 (0.999, 1.000)	0.426822816
IRF3	Diagnoses - main ICD10: L03.1 Cellulitis of other parts of limb	L03.1	1.001 (1.000, 1.002)	0.070316464
IRF3	Diagnoses - secondary ICD10: R53 Malaise and fatigue	R53	1.000 (0.999, 1.000)	0.276524695
IRF3	Diagnoses - secondary ICD10: W01.9 Unspecified place	W01.9	1.000 (0.999, 1.000)	0.085609214
IRF3	Diagnoses - main ICD10: C50.4 Upper-outer quadrant of breast	C50.4	1.000 (0.999, 1.000)	0.10486081
IRF3	Diagnoses - main ICD10: T39.1 4-Aminophenol derivatives	T39.1	1.000 (1.000, 1.001)	0.24089627
IRF3	Diagnoses - secondary ICD10: K29.5 Chronic gastritis, unspecified	K29.5	1.000 (0.999, 1.000)	0.691259231
IRF3	Diagnoses - secondary ICD10: G55.1 Nerve root and plexus compressions in intervertebral disk disorders	G55.1	1.000 (0.999, 1.000)	0.501381707
IRF3	Diagnoses - secondary ICD10: K25.9 Unspecified as acute or chronic, without haemorrhage or perforation	K25.9	1.000 (1.000, 1.001)	0.801577865
IRF3	Diagnoses - main ICD10: I80.2 Phlebitis and thrombophlebitis of other deep vessels of lower extremities	I80.2	1.000 (0.999, 1.001)	0.914301316
IRF3	Diagnoses - secondary ICD10: I84.6 Residual haemorrhoidal skin tags	I84.6	1.000 (1.000, 1.001)	0.811070322
IRF3	Diagnoses - main ICD10: N32.0 Bladder-neck obstruction	N32.0	1.000 (1.000, 1.001)	0.341257255
IRF3	Diagnoses - main ICD10: K29.8 Duodenitis	K29.8	1.000 (1.000, 1.001)	0.672009895
IRF3	Diagnoses - main ICD10: M67.4 Ganglion	M67.4	1.000 (0.999, 1.001)	0.778739443
IRF3	Diagnoses - main ICD10: H26.9 Cataract, unspecified	H26.9	1.000 (0.999, 1.001)	0.87483493
IRF3	Type of cancer: ICD10: C20 Malignant neoplasm of rectum	C20	1.000 (1.000, 1.001)	0.334211756
IRF3	Diagnoses - secondary ICD10: K30 Dyspepsia	K30	1.000 (0.999, 1.001)	0.726270738
IRF3	Diagnoses - secondary ICD10: I84.2 Internal haemorrhoids without complication	I84.2	1.000 (1.000, 1.001)	0.546467856
IRF3	Diagnoses - secondary ICD10: N94.6 Dysmenorrhoea, unspecified	N94.6	1.000 (0.999, 1.000)	0.719001019
IRF3	Diagnoses - main ICD10: Z08.0 Follow-up examination after surgery for malignant neoplasm	Z08.0	1.000 (0.999, 1.001)	0.55950751
IRF3	Diagnoses - main ICD10: M79.86 Other specified soft tissue disorders (Lower leg)	M79.86	1.000 (1.000, 1.001)	0.343279287
IRF3	Diagnoses - secondary ICD10: E78.0 Pure hypercholesterolaemia	E78.0	1.001 (0.999, 1.003)	0.387272289
IRF3	Diagnoses - secondary ICD10: M10.99 Gout, unspecified (Site unspecified)	M10.99	1.000 (1.000, 1.001)	0.525359702
IRF3	Diagnoses - main ICD10: M51.2 Other specified intervertebral disk displacement	M51.2	1.000 (0.999, 1.000)	0.30065613
IRF3	Diagnoses - main ICD10: N84.0 Polyp of corpus uteri	N84.0	1.000 (0.999, 1.001)	0.847813829
IRF3	Diagnoses - secondary ICD10: M17.1 Other primary gonarthrosis	M17.1	1.000 (1.000, 1.001)	0.740704413
IRF3	Diagnoses - main ICD10: M25.5 Pain in joint	M25.5	1.000 (0.999, 1.000)	0.663859647
IRF3	Diagnoses - main ICD10: K29.6 Other gastritis	K29.6	1.000 (0.999, 1.000)	0.312504449
IRF3	Type of cancer: ICD10: C64 Malignant neoplasm of kidney, except renal pelvis	C64	1.000 (0.999, 1.000)	0.661385912
IRF3	Diagnoses - main ICD10: G43.9 Migraine, unspecified	G43.9	1.000 (0.999, 1.000)	0.746401597
IRF3	Diagnoses - secondary ICD10: Z80.0 Family history of malignant neoplasm of digestive organs	Z80.0	1.000 (0.999, 1.001)	0.955843436

IRF3	Diagnoses - main ICD10: G45.9 Transient cerebral ischaemic attack, unspecified	G45.9	1.000 (1.000, 1.001)	0.647251398
IRF3	Diagnoses - main ICD10: O02.1 Missed abortion	O02.1	1.000 (1.000, 1.000)	0.987524214
IRF3	Diagnoses - main ICD10: K62.8 Other specified diseases of anus and rectum	K62.8	1.000 (0.999, 1.000)	0.897239755
IRF3	Diagnoses - main ICD10: M72.0 Palmar fascial fibromatosis [Dupuytren]	M72.0	1.000 (1.000, 1.001)	0.104999826
IRF3	Diagnoses - secondary ICD10: I34.0 Mitral (valve) insufficiency	I34.0	1.000 (1.000, 1.000)	0.976098357
IRF3	Diagnoses - main ICD10: O04.9 Complete or unspecified, without complication	O04.9	1.000 (0.999, 1.000)	0.058488934
IRF3	Diagnoses - main ICD10: K22.1 Ulcer of oesophagus	K22.1	1.001 (1.000, 1.002)	0.024659946
IRF3	Diagnoses - secondary ICD10: Z86.7 Personal history of diseases of the circulatory system	Z86.7	0.999 (0.998, 1.001)	0.349322982
IRF3	Diagnoses - secondary ICD10: Y83.1 Surgical operation with implant of artificial internal device	Y83.1	1.001 (1.000, 1.002)	0.082441315
IRF3	Diagnoses - secondary ICD10: R63.4 Abnormal weight loss	R63.4	1.000 (0.999, 1.001)	0.595442645
IRF3	Diagnoses - main ICD10: K21.9 Gastro-oesophageal reflux disease without oesophagitis	K21.9	1.000 (0.999, 1.001)	0.390360268
IRF3	Diagnoses - main ICD10: N93.8 Other specified abnormal uterine and vaginal bleeding	N93.8	1.000 (0.999, 1.000)	0.577392572
IRF3	Diagnoses - main ICD10: M75.4 Impingement syndrome of shoulder	M75.4	1.000 (0.999, 1.001)	0.575477991
IRF3	Diagnoses - main ICD10: I20.9 Angina pectoris, unspecified	I20.9	1.000 (0.999, 1.001)	0.43389477
IRF3	Diagnoses - secondary ICD10: X59.9 Unspecified place	X59.9	1.000 (1.000, 1.001)	0.452649496
IRF3	Diagnoses - main ICD10: I84.1 Internal haemorrhoids with other complications	I84.1	1.001 (1.000, 1.001)	0.043149391
IRF3	Diagnoses - secondary ICD10: K57.9 Diverticular disease of intestine, part unspecified, without perforation or absce	K57.9	1.000 (0.999, 1.001)	0.662053688
IRF3	Diagnoses - main ICD10: L72.9 Follicular cyst of skin and subcutaneous tissue, unspecified	L72.9	1.000 (1.000, 1.001)	0.210087675
IRF3	Diagnoses - secondary ICD10: Z92.3 Personal history of irradiation	Z92.3	1.000 (0.999, 1.000)	0.657101427
IRF3	Diagnoses - main ICD10: Z09.8 Follow-up examination after other treatment for other conditions	Z09.8	1.000 (1.000, 1.001)	0.464406753
IRF3	Diagnoses - main ICD10: K42.9 Umbilical hernia without obstruction or gangrene	K42.9	1.000 (0.999, 1.001)	0.903113823
IRF3	Diagnoses - main ICD10: R07.3 Other chest pain	R07.3	1.000 (0.999, 1.001)	0.771604692
IRF3	Diagnoses - main ICD10: N92.6 Irregular menstruation, unspecified	N92.6	1.000 (1.000, 1.001)	0.372406299
IRF3	Diagnoses - main ICD10: T84.0 Mechanical complication of internal joint prosthesis	T84.0	1.000 (1.000, 1.001)	0.26199153
IRF3	Diagnoses - main ICD10: D64.9 Anaemia, unspecified	D64.9	1.000 (1.000, 1.001)	0.398685424
IRF3	Diagnoses - secondary ICD10: R00.2 Palpitations	R00.2	1.000 (1.000, 1.001)	0.292722317
IRF3	Diagnoses - secondary ICD10: Z90.4 Acquired absence of other parts of digestive tract	Z90.4	1.001 (1.000, 1.002)	0.07850492
IRF3	Type of cancer: ICD10: C54.1 Endometrium	C54.1	0.999 (0.999, 1.000)	0.048951577
IRF3	Diagnoses - main ICD10: K92.2 Gastro-intestinal haemorrhage, unspecified	K92.2	1.000 (0.999, 1.000)	0.933630724
IRF3	Diagnoses - main ICD10: L72.0 Epidermal cyst	L72.0	1.000 (1.000, 1.001)	0.643365257
IRF3	Diagnoses - secondary ICD10: M13.99 Arthritis, unspecified (Site unspecified)	M13.99	1.000 (0.999, 1.001)	0.982650324
IRF3	Diagnoses - secondary ICD10: Z95.8 Presence of other cardiac and vascular implants and grafts	Z95.8	1.000 (1.000, 1.001)	0.7883916
IRF3	Diagnoses - secondary ICD10: K29.7 Gastritis, unspecified	K29.7	0.999 (0.998, 1.001)	0.329736239
IRF3	Diagnoses - secondary ICD10: E66.9 Obesity, unspecified	E66.9	0.999 (0.998, 1.000)	0.19246537
IRF3	Diagnoses - main ICD10: M54.5 Low back pain	M54.5	1.000 (0.999, 1.001)	0.839146406
IRF3	Diagnoses - main ICD10: J18.9 Pneumonia, unspecified	J18.9	1.001 (1.000, 1.001)	0.064931342
IRF3	Type of cancer: ICD10: C44.5 Skin of trunk	C44.5	1.000 (0.999, 1.000)	0.533586649
IRF3	Diagnoses - secondary ICD10: Z95.5 Presence of coronary angioplasty implant and graft	Z95.5	1.000 (0.999, 1.001)	0.737181443
IRF3	Type of cancer: ICD10: C18.7 Sigmoid colon	C18.7	1.000 (1.000, 1.001)	0.390126296
IRF3	Diagnoses - main ICD10: R87.6 Abnormal cytological findings	R87.6	1.000 (0.999, 1.000)	0.764731601

IRF3	Diagnoses - secondary ICD10: Z86.4 Personal history of psychoactive substance abuse	Z86.4	1.001 (1.000, 1.002)	0.180822439
IRF3	Diagnoses - secondary ICD10: Z51.5 Palliative care	Z51.5	1.000 (0.999, 1.000)	0.063352128
IRF3	Diagnoses - secondary ICD10: G40.9 Epilepsy, unspecified	G40.9	1.000 (0.999, 1.001)	0.948426682
IRF3	Diagnoses - secondary ICD10: Z51.0 Radiotherapy session	Z51.0	1.000 (0.999, 1.000)	0.119098074
IRF3	Diagnoses - secondary ICD10: F10.1 Harmful use	F10.1	1.000 (1.000, 1.001)	0.681291525
IRF3	Diagnoses - secondary ICD10: I51.7 Cardiomegaly	I51.7	1.000 (0.999, 1.000)	0.228548763
IRF3	Diagnoses - main ICD10: N23 Unspecified renal colic	N23	1.000 (1.000, 1.001)	0.522084473
IRF3	Diagnoses - secondary ICD10: E86 Volume depletion	E86	1.000 (1.000, 1.000)	0.931839498
IRF3	Diagnoses - secondary ICD10: Z87.1 Personal history of diseases of the digestive system	Z87.1	1.000 (0.998, 1.001)	0.770536732
IRF3	Diagnoses - secondary ICD10: Z85.0 Personal history of malignant neoplasm of digestive organs	Z85.0	1.000 (0.999, 1.001)	0.783985442
IRF3	Diagnoses - secondary ICD10: R03.0 Elevated blood-pressure reading, without diagnosis of hypertension	R03.0	1.000 (0.999, 1.000)	0.296589807
IRF3	Diagnoses - secondary ICD10: I25.8 Other forms of chronic ischaemic heart disease	I25.8	1.000 (0.999, 1.001)	0.977318329
IRF3	Diagnoses - secondary ICD10: Z90.1 Acquired absence of breast(s)	Z90.1	1.000 (0.999, 1.000)	0.453291288
IRF3	Diagnoses - secondary ICD10: I25.2 Old myocardial infarction	I25.2	1.000 (0.999, 1.001)	0.496862514
IRF3	Diagnoses - main ICD10: I25.1 Atherosclerotic heart disease	I25.1	1.001 (0.999, 1.002)	0.196035311
IRF3	Diagnoses - secondary ICD10: Z91.0 Personal history of allergy, other than to drugs and biological substances	Z91.0	0.999 (0.999, 1.000)	0.01073861
IRF3	Diagnoses - secondary ICD10: C78.7 Secondary malignant neoplasm of liver	C78.7	1.000 (1.000, 1.001)	0.397514884
IRF3	Diagnoses - secondary ICD10: J44.9 Chronic obstructive pulmonary disease, unspecified	J44.9	1.001 (1.000, 1.002)	0.029072058
IRF3	Diagnoses - main ICD10: G47.3 Sleep apnoea	G47.3	1.000 (1.000, 1.001)	0.35989326
IRF3	Diagnoses - secondary ICD10: N95.0 Postmenopausal bleeding	N95.0	1.000 (0.999, 1.000)	0.501025066
IRF3	Diagnoses - main ICD10: S09.9 Unspecified injury of head	S09.9	1.000 (0.999, 1.000)	0.238926571
IRF3	Diagnoses - secondary ICD10: Z95.0 Presence of cardiac pacemaker	Z95.0	1.000 (1.000, 1.001)	0.621435456
IRF3	Diagnoses - secondary ICD10: C77.3 Axillary and upper limb lymph nodes	C77.3	1.000 (1.000, 1.001)	0.498171182
IRF3	Type of cancer: ICD10: C44.4 Skin of scalp and neck	C44.4	1.000 (0.999, 1.000)	0.924369576
IRF3	Diagnoses - secondary ICD10: J45.9 Asthma, unspecified	J45.9	1.000 (0.999, 1.002)	0.674762089
IRF3	Diagnoses - main ICD10: R39.8 Other and unspecified symptoms and signs involving the urinary system	R39.8	1.000 (0.999, 1.000)	0.683970561
IRF3	Diagnoses - secondary ICD10: R00.0 Tachycardia, unspecified	R00.0	1.000 (0.999, 1.000)	0.418621866
IRF3	Diagnoses - main ICD10: R10.3 Pain localised to other parts of lower abdomen	R10.3	1.000 (0.999, 1.001)	0.948978674
IRF3	Diagnoses - secondary ICD10: E78.5 Hyperlipidaemia, unspecified	E78.5	1.000 (0.999, 1.001)	0.533312405
IRF3	Diagnoses - secondary ICD10: E14.9 Without complications	E14.9	1.000 (0.999, 1.000)	0.143356062
IRF3	Diagnoses - main ICD10: T81.0 Haemorrhage and haematoma complicating a procedure, not elsewhere classified	T81.0	1.001 (1.000, 1.001)	0.084344296
IRF3	Diagnoses - secondary ICD10: J18.1 Lobar pneumonia, unspecified	J18.1	1.000 (1.000, 1.000)	0.836569853
IRF3	Diagnoses - secondary ICD10: M81.99 Osteoporosis, unspecified (Site unspecified)	M81.99	1.000 (0.999, 1.000)	0.465080565
IRF3	Diagnoses - secondary ICD10: Y83.8 Other surgical procedures	Y83.8	1.001 (1.000, 1.002)	0.012056134
IRF3	Diagnoses - secondary ICD10: K63.5 Polyp of colon	K63.5	1.000 (0.999, 1.000)	0.240403782
IRF3	Diagnoses - main ICD10: K58.9 Irritable bowel syndrome without diarrhoea	K58.9	1.000 (1.000, 1.001)	0.250761684
IRF3	Diagnoses - secondary ICD10: K59.0 Constipation	K59.0	1.000 (0.999, 1.001)	0.580327411
IRF3	Diagnoses - secondary ICD10: Z87.3 Personal history of diseases of the musculoskeletal system and connective tissu	Z87.3	1.000 (1.000, 1.001)	0.476882518
IRF3	Type of cancer: ICD10: C56 Malignant neoplasm of ovary	C56	1.000 (0.999, 1.000)	0.677636427
IRF3	Diagnoses - main ICD10: M51.1 Lumbar and other intervertebral disk disorders with radiculopathy	M51.1	1.000 (0.999, 1.000)	0.251062768

IRF3	Diagnoses - main ICD10: I26.9 Pulmonary embolism without mention of acute cor pulmonale	I26.9	1.000 (0.999, 1.001)	0.833667155
IRF3	Diagnoses - main ICD10: N20.0 Calculus of kidney	N20.0	1.000 (0.999, 1.000)	0.583475355
IRF3	Diagnoses - main ICD10: M17.9 Gonarthrosis, unspecified	M17.9	1.000 (0.999, 1.001)	0.569128963
IRF3	Diagnoses - main ICD10: K92.0 Haematemesis	K92.0	1.000 (1.000, 1.001)	0.512192514
IRF3	Diagnoses - main ICD10: N20.1 Calculus of ureter	N20.1	1.000 (0.999, 1.001)	0.866219453
IRF3	Diagnoses - main ICD10: K13.7 Other and unspecified lesions of oral mucosa	K13.7	1.000 (0.999, 1.000)	0.711109389
IRF3	Type of cancer: ICD10: C34.1 Upper lobe, bronchus or lung	C34.1	1.000 (0.999, 1.000)	0.702274516
IRF3	Diagnoses - main ICD10: Z42.1 Follow-up care involving plastic surgery of breast	Z42.1	1.001 (1.000, 1.001)	0.024011956
IRF3	Type of cancer: ICD10: C50.9 Breast, unspecified	C50.9	1.000 (0.999, 1.001)	0.874324135
IRF3	Diagnoses - main ICD10: J34.8 Other specified disorders of nose and nasal sinuses	J34.8	1.000 (0.999, 1.000)	0.159322343
IRF3	Type of cancer: ICD10: C44.6 Skin of upper limb, including shoulder	C44.6	1.000 (0.999, 1.000)	0.292711758
IRF3	Diagnoses - secondary ICD10: R50.9 Fever, unspecified	R50.9	1.000 (0.999, 1.001)	0.853721721
IRF3	Diagnoses - secondary ICD10: W10.0 Home	W10.0	1.000 (1.000, 1.000)	0.990101112
IRF3	Diagnoses - main ICD10: N84.1 Polyp of cervix uteri	N84.1	1.000 (0.999, 1.000)	0.202377102
IRF3	Diagnoses - secondary ICD10: R33 Retention of urine	R33	1.000 (0.999, 1.001)	0.941133091
IRF3	Diagnoses - main ICD10: D12.6 Colon, unspecified	D12.6	1.000 (0.999, 1.001)	0.792483833
IRF3	Diagnoses - main ICD10: I63.9 Cerebral infarction, unspecified	I63.9	1.000 (0.999, 1.000)	0.323370801
IRF3	Diagnoses - main ICD10: K51.9 Ulcerative colitis, unspecified	K51.9	0.999 (0.999, 1.000)	0.009125007
IRF3	Diagnoses - main ICD10: K85 Acute pancreatitis	K85	1.000 (0.999, 1.000)	0.904536128
IRF3	Diagnoses - secondary ICD10: R06.0 Dyspnoea	R06.0	1.000 (0.999, 1.001)	0.800436797
IRF3	Diagnoses - secondary ICD10: R10.4 Other and unspecified abdominal pain	R10.4	1.000 (0.999, 1.001)	0.561332762
IRF3	Diagnoses - main ICD10: Z47.0 Follow-up care involving removal of fracture plate and other internal fixation devic	Z47.0	1.000 (0.999, 1.001)	0.864336099
IRF3	Diagnoses - secondary ICD10: F10.2 Dependence syndrome	F10.2	1.000 (1.000, 1.001)	0.40495417
IRF3	Diagnoses - main ICD10: R06.5 Mouth breathing	R06.5	1.000 (0.999, 1.000)	0.275453681
IRF3	Diagnoses - main ICD10: R51 Headache	R51	1.000 (0.999, 1.001)	0.643416541
IRF3	Diagnoses - main ICD10: K62.1 Rectal polyp	K62.1	1.000 (0.999, 1.000)	0.294739812
IRF3	Diagnoses - main ICD10: M51.3 Other specified intervertebral disk degeneration	M51.3	1.000 (1.000, 1.001)	0.141862817
IRF3	Diagnoses - main ICD10: M54.56 Low back pain (Lumbar region)	M54.56	0.999 (0.999, 1.000)	0.026950979
IRF3	Diagnoses - main ICD10: M16.9 Coxarthrosis, unspecified	M16.9	1.001 (1.000, 1.002)	0.152671461
IRF3	Diagnoses - secondary ICD10: Z86.1 Personal history of infectious and parasitic diseases	Z86.1	1.000 (0.999, 1.000)	0.148298815
IRF3	Diagnoses - secondary ICD10: I12.0 Hypertensive renal disease with renal failure	I12.0	1.000 (1.000, 1.001)	0.186180048
IRF3	Diagnoses - secondary ICD10: R05 Cough	R05	1.000 (0.999, 1.000)	0.213434326
IRF3	Diagnoses - main ICD10: Z13.8 Special screening examination for other specified diseases and disorders	Z13.8	1.000 (0.999, 1.000)	0.527401255
IRF3	Diagnoses - secondary ICD10: F32.9 Depressive episode, unspecified	F32.9	1.000 (0.999, 1.001)	0.494518079
IRF3	Diagnoses - secondary ICD10: Z85.3 Personal history of malignant neoplasm of breast	Z85.3	1.000 (0.999, 1.001)	0.730961309
IRF3	Diagnoses - main ICD10: K52.9 Non-infective gastro-enteritis and colitis, unspecified	K52.9	1.000 (0.999, 1.002)	0.714527874
IRF3	Diagnoses - main ICD10: L98.9 Disorder of skin and subcutaneous tissue, unspecified	L98.9	1.000 (0.999, 1.000)	0.255173842
IRF3	Diagnoses - secondary ICD10: M19.99 Arthrosis, unspecified (Site unspecified)	M19.99	1.000 (0.999, 1.001)	0.800237999
IRF3	Diagnoses - main ICD10: Z12.1 Special screening examination for neoplasm of intestinal tract	Z12.1	1.000 (0.999, 1.000)	0.39060995
IRF3	Diagnoses - main ICD10: R07.2 Precordial pain	R07.2	1.000 (0.999, 1.000)	0.275445901

IRF3	Diagnoses - secondary ICD10: Z90.7 Acquired absence of genital organ(s)	Z90.7	0.999 (0.999, 1.000)	0.177913009
IRF3	Diagnoses - secondary ICD10: Z96.6 Presence of orthopaedic joint implants	Z96.6	1.001 (1.000, 1.002)	0.110149039
IRF3	Diagnoses - secondary ICD10: B96.8 Other specified bacterial agents as the cause of diseases classified to other chap	B96.8	1.000 (0.999, 1.001)	0.720776703
IRF3	Diagnoses - secondary ICD10: R42 Dizziness and giddiness	R42	1.000 (0.999, 1.001)	0.856492021
IRF3	Diagnoses - secondary ICD10: N81.2 Incomplete uterovaginal prolapse	N81.2	1.000 (0.999, 1.000)	0.624644597
IRF3	Diagnoses - main ICD10: M25.56 Pain in joint (Lower leg)	M25.56	1.000 (1.000, 1.001)	0.07538066
IRF3	Diagnoses - main ICD10: D17.2 Benign lipomatous neoplasm of skin and subcutaneous tissue of limbs	D17.2	1.000 (1.000, 1.001)	0.53459666
IRF3	Diagnoses - main ICD10: O63.1 Prolonged second stage (of labour)	O63.1	1.000 (1.000, 1.001)	0.184435789
IRF3	Diagnoses - secondary ICD10: Z82.4 Family history of ischaemic heart disease and other diseases of the circulatory s	Z82.4	1.000 (0.999, 1.001)	0.907932903
IRF3	Diagnoses - main ICD10: H25.0 Senile incipient cataract	H25.0	1.000 (1.000, 1.001)	0.293443505
IRF3	Diagnoses - main ICD10: D17.1 Benign lipomatous neoplasm of skin and subcutaneous tissue of trunk	D17.1	1.001 (1.000, 1.001)	0.05585709
IRF3	Diagnoses - secondary ICD10: M47.82 Other spondylosis (Cervical region)	M47.82	1.000 (1.000, 1.001)	0.174005492
IRF3	Diagnoses - secondary ICD10: Z98.0 Intestinal bypass and anastomosis status	Z98.0	1.000 (1.000, 1.000)	0.906683933
IRF3	Type of cancer: ICD10: C43.7 Malignant melanoma of lower limb, including hip	C43.7	1.000 (0.999, 1.000)	0.649339769
IRF3	Diagnoses - main ICD10: S52.50 Fracture of lower end of radius (closed)	S52.50	1.001 (1.000, 1.001)	0.037446059
IRF3	Diagnoses - secondary ICD10: W19.9 Unspecified place	W19.9	1.000 (0.999, 1.000)	0.372448494
IRF3	Diagnoses - main ICD10: M16.1 Other primary coxarthrosis	M16.1	1.000 (1.000, 1.001)	0.228854206
IRF3	Diagnoses - secondary ICD10: Z60.2 Living alone	Z60.2	1.000 (0.999, 1.001)	0.782696056
IRF3	Diagnoses - secondary ICD10: F17.1 Harmful use	F17.1	1.000 (0.999, 1.001)	0.997977302
IRF3	Diagnoses - secondary ICD10: K31.8 Other specified diseases of stomach and duodenum	K31.8	1.000 (1.000, 1.001)	0.556674403
IRF3	Diagnoses - secondary ICD10: K31.7 Polyp of stomach and duodenum	K31.7	1.000 (1.000, 1.000)	0.941380562
IRF3	Diagnoses - main ICD10: H25.1 Senile nuclear cataract	H25.1	1.000 (0.999, 1.000)	0.520354701
IRF3	Diagnoses - secondary ICD10: Y83.2 Surgical operation with anastomosis, bypass or graft	Y83.2	1.000 (1.000, 1.001)	0.751881348
IRF3	Diagnoses - secondary ICD10: D70 Agranulocytosis	D70	1.000 (0.999, 1.000)	0.217508323
IRF3	Diagnoses - main ICD10: D50.9 Iron deficiency anaemia, unspecified	D50.9	1.000 (0.999, 1.000)	0.42769317
IRF3	Diagnoses - secondary ICD10: N81.6 Rectocele	N81.6	1.000 (0.999, 1.000)	0.236926139
IRF3	Diagnoses - main ICD10: I21.9 Acute myocardial infarction, unspecified	I21.9	1.000 (1.000, 1.001)	0.173198492
IRF3	Diagnoses - main ICD10: D22.3 Melanocytic naevi of other and unspecified parts of face	D22.3	1.000 (1.000, 1.001)	0.755042206
IRF3	Diagnoses - secondary ICD10: K44.9 Diaphragmatic hernia without obstruction or gangrene	K44.9	1.001 (1.000, 1.003)	0.139148875
IRF3	Diagnoses - main ICD10: B34.9 Viral infection, unspecified	B34.9	1.000 (1.000, 1.001)	0.530626734
IRF3	Diagnoses - main ICD10: N32.8 Other specified disorders of bladder	N32.8	1.000 (0.999, 1.000)	0.291080501
IRF3	Diagnoses - main ICD10: J34.2 Deviated nasal septum	J34.2	1.000 (1.000, 1.001)	0.227911222
IRF3	Diagnoses - main ICD10: N50.8 Other specified disorders of male genital organs	N50.8	1.000 (0.999, 1.000)	0.247859381
IRF3	Diagnoses - secondary ICD10: Z86.0 Personal history of other neoplasms	Z86.0	1.000 (0.999, 1.001)	0.979153254
IRF3	Diagnoses - secondary ICD10: Z72.1 Alcohol use	Z72.1	1.000 (1.000, 1.001)	0.249688088
IRF3	Diagnoses - main ICD10: G56.0 Carpal tunnel syndrome	G56.0	1.000 (0.999, 1.002)	0.629894321
IRF3	Diagnoses - main ICD10: I25.9 Chronic ischaemic heart disease, unspecified	I25.9	1.000 (1.000, 1.001)	0.184715763
IRF3	Diagnoses - secondary ICD10: Z37.0 Single live birth	Z37.0	1.000 (0.999, 1.002)	0.698033895
IRF3	Diagnoses - secondary ICD10: E03.9 Hypothyroidism, unspecified	E03.9	1.002 (1.000, 1.003)	0.007780257
IRF3	Diagnoses - secondary ICD10: Z88.6 Personal history of allergy to analgesic agent	Z88.6	1.001 (1.000, 1.001)	0.076834076

IRF3	Diagnoses - main ICD10: M20.2 Hallux rigidus	M20.2	1.000 (0.999, 1.000)	0.438897611
IRF3	Diagnoses - secondary ICD10: B95.6 Staphylococcus aureus as the cause of diseases classified to other chapters	B95.6	1.000 (1.000, 1.001)	0.653850967
IRF3	Diagnoses - main ICD10: R04.2 Haemoptysis	R04.2	1.000 (0.999, 1.000)	0.332369935
IRF3	Diagnoses - main ICD10: I21.0 Acute transmural myocardial infarction of anterior wall	I21.0	1.000 (0.999, 1.000)	0.831244758
IRF3	Diagnoses - secondary ICD10: R94.5 Abnormal results of liver function studies	R94.5	1.000 (0.999, 1.000)	0.693096078
IRF3	Diagnoses - secondary ICD10: T81.4 Infection following a procedure, not elsewhere classified	T81.4	1.000 (1.000, 1.001)	0.328811952
IRF3	Diagnoses - secondary ICD10: Z88.8 Personal history of allergy to other drugs, medicaments and biological substan	Z88.8	1.000 (0.999, 1.001)	0.899380041
IRF3	Diagnoses - main ICD10: H00.1 Chalazion	H00.1	1.000 (0.999, 1.000)	0.118843869
IRF3	Type of cancer: ICD10: D05.1 Intraductal carcinoma in situ	D05.1	1.000 (0.999, 1.001)	0.922827643
IRF3	Diagnoses - secondary ICD10: Y43.3 Other antineoplastic drugs	Y43.3	1.000 (0.999, 1.000)	0.361381705
IRF3	Diagnoses - main ICD10: K02.9 Dental caries, unspecified	K02.9	1.000 (1.000, 1.001)	0.468615154
IRF3	Diagnoses - main ICD10: M72.04 Palmar fascial fibromatosis [Dupuytren]-Hand	M72.04	1.000 (1.000, 1.001)	0.405006491
IRF3	Diagnoses - secondary ICD10: N83.2 Other and unspecified ovarian cysts	N83.2	1.000 (0.999, 1.000)	0.47090426
IRF3	Diagnoses - main ICD10: M75.0 Adhesive capsulitis of shoulder	M75.0	1.000 (1.000, 1.001)	0.062080303
IRF3	Diagnoses - secondary ICD10: I73.9 Peripheral vascular disease, unspecified	I73.9	1.000 (1.000, 1.001)	0.617021842
IRF3	Diagnoses - secondary ICD10: N17.9 Acute renal failure, unspecified	N17.9	1.000 (0.999, 1.000)	0.567768462
IRF3	Diagnoses - main ICD10: M75.1 Rotator cuff syndrome	M75.1	1.000 (1.000, 1.001)	0.377715226
IRF3	Diagnoses - main ICD10: L82 Seborrhoeic keratosis	L82	0.999 (0.999, 1.000)	0.012964999
IRF3	Diagnoses - main ICD10: K61.0 Anal abscess	K61.0	1.000 (0.999, 1.000)	0.440518825
IRF3	Diagnoses - secondary ICD10: Z53.2 Procedure not carried out because of patient's decision for other and unspecifi	Z53.2	1.000 (0.999, 1.000)	0.6619078
IRF3	Diagnoses - secondary ICD10: Z72.0 Tobacco use	Z72.0	1.001 (1.000, 1.003)	0.116213429
IRF3	Diagnoses - main ICD10: I21.1 Acute transmural myocardial infarction of inferior wall	I21.1	1.000 (0.999, 1.000)	0.380381827
IRF3	Diagnoses - secondary ICD10: N39.3 Stress incontinence	N39.3	1.000 (0.999, 1.000)	0.425779411
IRF3	Diagnoses - main ICD10: K40.9 Unilateral or unspecified inguinal hernia, without obstruction or gangrene	K40.9	1.000 (0.998, 1.001)	0.638888678
IRF3	Diagnoses - main ICD10: C67.9 Bladder, unspecified	C67.9	1.000 (0.999, 1.000)	0.494340802
IRF3	Diagnoses - secondary ICD10: Z13.0 Special screening examination for diseases of the blood and blood-forming or	Z13.0	1.000 (1.000, 1.001)	0.413032592
IRF3	Diagnoses - secondary ICD10: Z86.6 Personal history of diseases of the nervous system and sense organs	Z86.6	0.999 (0.999, 1.000)	0.150704504
IRF3	Diagnoses - main ICD10: K92.1 Melaena	K92.1	1.000 (0.999, 1.000)	0.598844765
IRF3	Diagnoses - secondary ICD10: J34.3 Hypertrophy of nasal turbinates	J34.3	1.000 (1.000, 1.001)	0.278826337
IRF3	Diagnoses - secondary ICD10: Z88.0 Personal history of allergy to penicillin	Z88.0	0.999 (0.998, 1.001)	0.226804843
IRF3	Diagnoses - main ICD10: S82.80 Fractures of other parts of lower leg (closed)	S82.80	1.000 (0.999, 1.000)	0.315447962
IRF3	Diagnoses - main ICD10: M20.1 Hallux valgus (acquired)	M20.1	1.000 (0.999, 1.001)	0.977503562
IRF3	Diagnoses - secondary ICD10: N39.0 Urinary tract infection, site not specified	N39.0	1.000 (0.999, 1.001)	0.571283382
IRF3	Diagnoses - secondary ICD10: Z03.4 Observation for suspected myocardial infarction	Z03.4	1.000 (1.000, 1.001)	0.401112634
IRF3	Diagnoses - main ICD10: O80.0 Spontaneous vertex delivery	O80.0	1.000 (0.999, 1.000)	0.719119118
IRF3	Diagnoses - secondary ICD10: N73.6 Female pelvic peritoneal adhesions	N73.6	1.000 (1.000, 1.001)	0.185263594
IRF3	Diagnoses - secondary ICD10: D25.9 Leiomyoma of uterus, unspecified	D25.9	1.000 (0.999, 1.000)	0.350201591
IRF3	Diagnoses - secondary ICD10: K26.9 Unspecified as acute or chronic, without haemorrhage or perforation	K26.9	1.000 (1.000, 1.001)	0.220474708
IRF3	Diagnoses - main ICD10: K60.3 Anal fistula	K60.3	1.000 (0.999, 1.000)	0.258330755
IRF3	Diagnoses - main ICD10: K43.9 Ventral hernia without obstruction or gangrene	K43.9	1.000 (1.000, 1.001)	0.20174689

IRF3	Diagnoses - main ICD10: K35.9 Acute appendicitis, unspecified	K35.9	1.001 (1.000, 1.001)	0.0054037
IRF3	Type of cancer: ICD10: C44.7 Skin of lower limb, including hip	C44.7	1.000 (1.000, 1.000)	0.953500556
IRF3	Diagnoses - secondary ICD10: M15.9 Polyarthrosis, unspecified	M15.9	1.000 (1.000, 1.001)	0.312177382
IRF3	Diagnoses - main ICD10: R13 Dysphagia	R13	1.001 (1.000, 1.001)	0.102190321
IRF3	Diagnoses - main ICD10: M23.23 Derangement of meniscus due to old tear or injury (Medial collateral ligament or	M23.23	1.000 (1.000, 1.001)	0.341950114
IRF3	Diagnoses - secondary ICD10: Z51.2 Other chemotherapy	Z51.2	1.000 (0.999, 1.001)	0.716557109
IRF3	Diagnoses - main ICD10: J33.9 Nasal polyp, unspecified	J33.9	1.000 (0.999, 1.000)	0.67219364
IRF3	Diagnoses - secondary ICD10: Z85.5 Personal history of malignant neoplasm of urinary tract	Z85.5	1.000 (0.999, 1.000)	0.617436388
IRF3	Diagnoses - secondary ICD10: Z92.1 Personal history of long-term (current) use of anticoagulants	Z92.1	1.000 (0.999, 1.001)	0.46191021
IRF3	Diagnoses - main ICD10: Z30.2 Sterilisation	Z30.2	1.000 (0.999, 1.001)	0.742751079
IRF3	Diagnoses - main ICD10: N63 Unspecified lump in breast	N63	1.000 (1.000, 1.001)	0.727149265
IRF3	Diagnoses - main ICD10: I84.8 Unspecified haemorrhoids with other complications	I84.8	1.000 (0.999, 1.001)	0.967941097
IRF3	Diagnoses - main ICD10: Z46.6 Fitting and adjustment of urinary device	Z46.6	1.000 (0.999, 1.000)	0.218899703
IRF3	Diagnoses - main ICD10: K01.1 Impacted teeth	K01.1	1.000 (0.999, 1.000)	0.244376236
IRF3	Diagnoses - secondary ICD10: Z80.3 Family history of malignant neoplasm of breast	Z80.3	1.000 (0.999, 1.000)	0.557228284
IRF3	Diagnoses - secondary ICD10: Y83.6 Removal of other organ (partial) (total)	Y83.6	1.000 (0.999, 1.001)	0.934460168
IRF3	Diagnoses - main ICD10: M65.3 Trigger finger	M65.3	1.000 (0.999, 1.000)	0.158644601
IRF3	Diagnoses - secondary ICD10: Z92.4 Personal history of major surgery, not elsewhere classified	Z92.4	1.000 (1.000, 1.000)	0.861017103
IRF3	Diagnoses - main ICD10: M23.2 Derangement of meniscus due to old tear or injury	M23.2	1.001 (1.000, 1.001)	0.015632646
IRF3	Diagnoses - main ICD10: K80.5 Calculus of bile duct without cholangitis or cholecystitis	K80.5	1.000 (0.999, 1.001)	0.917216629
IRF3	Type of cancer: ICD10: C44.9 Malignant neoplasm of skin, unspecified	C44.9	0.999 (0.998, 1.000)	0.065376431
IRF3	Diagnoses - main ICD10: D24 Benign neoplasm of breast	D24	1.000 (0.999, 1.000)	0.783483068
IRF3	Diagnoses - secondary ICD10: N85.8 Other specified noninflammatory disorders of uterus	N85.8	1.000 (0.999, 1.000)	0.54534243
IRF3	Diagnoses - secondary ICD10: I95.9 Hypotension, unspecified	I95.9	1.000 (1.000, 1.001)	0.103839747
IRF3	Diagnoses - secondary ICD10: B96.2 Escherichia coli [E. coli] as the cause of diseases classified to other chapters	B96.2	1.000 (1.000, 1.001)	0.833535486
IRF3	Diagnoses - main ICD10: K80.1 Calculus of gallbladder with other cholecystitis	K80.1	0.999 (0.998, 1.000)	0.015662699
IRF3	Diagnoses - secondary ICD10: Z53.0 Procedure not carried out because of contraindication	Z53.0	1.000 (0.999, 1.001)	0.724166804
IRF3	Diagnoses - secondary ICD10: Z53.8 Procedure not carried out for other reasons	Z53.8	1.001 (0.999, 1.002)	0.363467269
IRF3	Diagnoses - main ICD10: N35.9 Urethral stricture, unspecified	N35.9	1.000 (0.999, 1.000)	0.19899889
IRF3	Type of cancer: ICD10: D06.9 Cervix, unspecified	D06.9	1.000 (0.999, 1.001)	0.581348548
IRF3	Diagnoses - secondary ICD10: Z53.9 Procedure not carried out, unspecified reason	Z53.9	1.000 (0.999, 1.001)	0.80356866
IRF3	Diagnoses - main ICD10: O68.0 Labour and delivery complicated by foetal heart rate anomaly	O68.0	1.000 (1.000, 1.001)	0.135521874
IRF3	Diagnoses - main ICD10: D12.8 Rectum	D12.8	1.000 (1.000, 1.001)	0.680384575
IRF3	Diagnoses - secondary ICD10: M19.9 Arthrosis, unspecified	M19.9	1.000 (1.000, 1.001)	0.586889732
IRF3	Diagnoses - main ICD10: H02.8 Other specified disorders of eyelid	H02.8	1.000 (0.999, 1.000)	0.304182921
IRF3	Diagnoses - main ICD10: N92.1 Excessive and frequent menstruation with irregular cycle	N92.1	1.000 (1.000, 1.001)	0.647906465
IRF3	Diagnoses - main ICD10: O70.0 First degree perineal laceration during delivery	O70.0	1.000 (1.000, 1.001)	0.091991013
IRF3	Diagnoses - secondary ICD10: N80.0 Endometriosis of uterus	N80.0	1.000 (1.000, 1.001)	0.148440743
IRF3	Diagnoses - main ICD10: M23.22 Derangement of meniscus due to old tear or injury (Posterior cruciate ligament or	M23.22	1.000 (1.000, 1.001)	0.12768057
IRF3	Diagnoses - secondary ICD10: E11.9 Without complications	E11.9	1.001 (0.999, 1.002)	0.274531671

IRF3	Diagnoses - main ICD10: M79.66 Pain in limb (Lower leg)	M79.66	1.000 (0.999, 1.000)	0.102839846
IRF3	Diagnoses - main ICD10: O26.8 Other specified pregnancy-related conditions	O26.8	1.000 (1.000, 1.001)	0.109936699
IRF3	Diagnoses - main ICD10: K56.6 Other and unspecified intestinal obstruction	K56.6	1.000 (1.000, 1.000)	0.848628465
IRF3	Diagnoses - secondary ICD10: Z88.1 Personal history of allergy to other antibiotic agents	Z88.1	1.000 (0.999, 1.001)	0.7003706
IRF3	Diagnoses - secondary ICD10: J90 Pleural effusion, not elsewhere classified	J90	1.000 (1.000, 1.001)	0.390748245
IRF3	Underlying (primary) cause of death: ICD10: C34.9 Bronchus or lung, unspecified	C34.9	1.004 (0.982, 1.026)	0.724328862
IRF3	Diagnoses - main ICD10: A41 Other septicaemia	A41	1.000 (0.999, 1.001)	0.758404191
IRF3	Diagnoses - main ICD10: C18 Malignant neoplasm of colon	C18	1.000 (0.999, 1.001)	0.404292939
IRF3	Diagnoses - main ICD10: C34 Malignant neoplasm of bronchus and lung	C34	1.001 (1.000, 1.001)	0.140740382
IRF3	Diagnoses - main ICD10: C43 Malignant melanoma of skin	C43	1.000 (0.999, 1.000)	0.2809137
IRF3	Diagnoses - main ICD10: C67 Malignant neoplasm of bladder	C67	1.000 (0.999, 1.001)	0.731505329
IRF3	Diagnoses - main ICD10: C78 Secondary malignant neoplasm of respiratory and digestive organs	C78	1.000 (0.999, 1.001)	0.758331163
IRF3	Diagnoses - main ICD10: C79 Secondary malignant neoplasm of other sites	C79	1.000 (0.999, 1.001)	0.728473777
IRF3	Diagnoses - main ICD10: D05 Carcinoma in situ of breast	D05	1.000 (0.999, 1.000)	0.354892395
IRF3	Diagnoses - main ICD10: D17 Benign lipomatous neoplasm	D17	1.001 (1.000, 1.003)	0.019595229
IRF3	Diagnoses - main ICD10: D22 Melanocytic naevi	D22	0.999 (0.998, 1.000)	0.292778548
IRF3	Diagnoses - main ICD10: D23 Other benign neoplasms of skin	D23	1.000 (0.999, 1.001)	0.964420297
IRF3	Diagnoses - main ICD10: D50 Iron deficiency anaemia	D50	1.000 (0.999, 1.002)	0.408319303
IRF3	Diagnoses - main ICD10: D64 Other anaemias	D64	1.000 (0.999, 1.002)	0.392935442
IRF3	Diagnoses - main ICD10: G43 Migraine	G43	1.000 (0.999, 1.001)	0.726772619
IRF3	Diagnoses - main ICD10: G45 Transient cerebral ischaemic attacks and related syndromes	G45	1.000 (0.999, 1.001)	0.517069038
IRF3	Diagnoses - main ICD10: G57 Mononeuropathies of lower limb	G57	1.000 (1.000, 1.001)	0.449759402
IRF3	Diagnoses - main ICD10: H00 Hordeolum and chalazion	H00	1.000 (0.999, 1.001)	0.711624371
IRF3	Diagnoses - main ICD10: H02 Other disorders of eyelid	H02	1.000 (0.998, 1.001)	0.437228457
IRF3	Diagnoses - main ICD10: H04 Disorders of lachrymal system	H04	1.000 (0.999, 1.001)	0.750940006
IRF3	Diagnoses - main ICD10: H33 Retinal detachments and breaks	H33	1.000 (0.999, 1.000)	0.362477161
IRF3	Diagnoses - main ICD10: H35 Other retinal disorders	H35	1.000 (0.999, 1.001)	0.80905701
IRF3	Diagnoses - main ICD10: H40 Glaucoma	H40	1.000 (0.999, 1.001)	0.671318986
IRF3	Diagnoses - main ICD10: I26 Pulmonary embolism	I26	0.999 (0.999, 1.000)	0.195512731
IRF3	Diagnoses - main ICD10: I47 Paroxysmal tachycardia	I47	1.000 (0.999, 1.000)	0.212917704
IRF3	Diagnoses - main ICD10: I50 Heart failure	I50	1.000 (1.000, 1.001)	0.15608065
IRF3	Diagnoses - main ICD10: I63 Cerebral infarction	I63	1.000 (0.999, 1.001)	0.611229197
IRF3	Diagnoses - main ICD10: J18 Pneumonia, organism unspecified	J18	1.001 (0.999, 1.002)	0.253276775
IRF3	Diagnoses - main ICD10: J32 Chronic sinusitis	J32	1.001 (1.000, 1.001)	0.051389982
IRF3	Diagnoses - main ICD10: J38 Diseases of vocal cords and larynx, not elsewhere classified	J38	1.000 (0.999, 1.001)	0.725454174
IRF3	Diagnoses - main ICD10: J45 Asthma	J45	1.000 (0.999, 1.000)	0.432829625
IRF3	Diagnoses - main ICD10: K01 Embedded and impacted teeth	K01	0.999 (0.999, 1.000)	0.045484284
IRF3	Diagnoses - main ICD10: K02 Dental caries	K02	1.000 (0.999, 1.001)	0.947493487
IRF3	Diagnoses - main ICD10: K04 Diseases of pulp and periapical tissues	K04	1.001 (1.000, 1.001)	0.155497213
IRF3	Diagnoses - main ICD10: K08 Other disorders of teeth and supporting structures	K08	1.000 (0.999, 1.001)	0.890175778

IRF3	Diagnoses - main ICD10: K13 Other diseases of lip and oral mucosa	K13	1.000 (0.999, 1.001)	0.852214146
IRF3	Diagnoses - main ICD10: K25 Gastric ulcer	K25	1.000 (0.999, 1.001)	0.854094057
IRF3	Diagnoses - main ICD10: K26 Duodenal ulcer	K26	1.001 (1.000, 1.001)	0.021150131
IRF3	Diagnoses - main ICD10: K31 Other diseases of stomach and duodenum	K31	1.000 (0.999, 1.001)	0.813399871
IRF3	Diagnoses - main ICD10: K42 Umbilical hernia	K42	1.000 (0.999, 1.001)	0.861134724
IRF3	Diagnoses - main ICD10: K56 Paralytic ileus and intestinal obstruction without hernia	K56	1.000 (1.000, 1.001)	0.421395139
IRF3	Diagnoses - main ICD10: K58 Irritable bowel syndrome	K58	1.000 (1.000, 1.001)	0.4794718
IRF3	Diagnoses - main ICD10: K59 Other functional intestinal disorders	K59	1.000 (0.999, 1.001)	0.974196809
IRF3	Diagnoses - main ICD10: K61 Abscess of anal and rectal regions	K61	1.000 (0.999, 1.000)	0.760229162
IRF3	Diagnoses - main ICD10: K63 Other diseases of intestine	K63	1.000 (0.999, 1.002)	0.800923228
IRF3	Diagnoses - main ICD10: K81 Cholecystitis	K81	1.000 (0.999, 1.001)	0.512795222
IRF3	Diagnoses - main ICD10: K92 Other diseases of digestive system	K92	1.001 (1.000, 1.002)	0.074111489
IRF3	Diagnoses - main ICD10: L02 Cutaneous abscess, furuncle and carbuncle	L02	1.001 (1.000, 1.001)	0.077370174
IRF3	Diagnoses - main ICD10: L57 Skin changes due to chronic exposure to nonionising radiation	L57	1.000 (0.999, 1.000)	0.318407736
IRF3	Diagnoses - main ICD10: L72 Follicular cysts of skin and subcutaneous tissue	L72	1.001 (0.999, 1.002)	0.481979948
IRF3	Diagnoses - main ICD10: L90 Atrophic disorders of skin	L90	1.000 (0.999, 1.000)	0.485505438
IRF3	Diagnoses - main ICD10: L98 Other disorders of skin and subcutaneous tissue, not elsewhere classified	L98	1.000 (0.999, 1.001)	0.604011103
IRF3	Diagnoses - main ICD10: M06 Other rheumatoid arthritis	M06	1.000 (0.999, 1.000)	0.465756107
IRF3	Diagnoses - main ICD10: M13 Other arthritis	M13	1.000 (0.999, 1.001)	0.782989617
IRF3	Diagnoses - main ICD10: M15 Polyarthrosis	M15	1.000 (1.000, 1.001)	0.167494505
IRF3	Diagnoses - main ICD10: M19 Other arthrosis	M19	1.000 (0.999, 1.001)	0.702836267
IRF3	Diagnoses - main ICD10: M47 Spondylosis	M47	1.000 (0.999, 1.001)	0.667149402
IRF3	Diagnoses - main ICD10: M48 Other spondylopathies	M48	1.000 (0.999, 1.001)	0.885076873
IRF3	Diagnoses - main ICD10: M51 Other intervertebral disk disorders	M51	1.000 (0.999, 1.001)	0.899403407
IRF3	Diagnoses - main ICD10: M65 Synovitis and tenosynovitis	M65	0.999 (0.998, 1.000)	0.110606542
IRF3	Diagnoses - main ICD10: M75 Shoulder lesions	M75	1.001 (0.999, 1.002)	0.321883462
IRF3	Diagnoses - main ICD10: M79 Other soft tissue disorders, not elsewhere classified	M79	1.001 (1.000, 1.003)	0.149579068
IRF3	Diagnoses - main ICD10: M84 Disorders of continuity of bone	M84	1.000 (0.999, 1.001)	0.935437789
IRF3	Diagnoses - main ICD10: N13 Obstructive and reflux uropathy	N13	1.000 (0.999, 1.000)	0.217171032
IRF3	Diagnoses - main ICD10: N30 Cystitis	N30	1.000 (0.999, 1.001)	0.881399843
IRF3	Diagnoses - main ICD10: N31 Neuromuscular dysfunction of bladder, not elsewhere classified	N31	1.000 (1.000, 1.001)	0.349474747
IRF3	Diagnoses - main ICD10: N35 Urethral stricture	N35	0.999 (0.998, 1.000)	0.084331812
IRF3	Diagnoses - main ICD10: N39 Other disorders of urinary system	N39	1.002 (1.000, 1.004)	0.059859845
IRF3	Diagnoses - main ICD10: N48 Other disorders of penis	N48	1.000 (0.999, 1.000)	0.236738885
IRF3	Diagnoses - main ICD10: N50 Other disorders of male genital organs	N50	1.000 (0.999, 1.001)	0.990264629
IRF3	Diagnoses - main ICD10: N60 Benign mammary dysplasia	N60	1.000 (0.999, 1.000)	0.284164251
IRF3	Diagnoses - main ICD10: N80 Endometriosis	N80	1.000 (0.999, 1.001)	0.663640664
IRF3	Diagnoses - main ICD10: N83 Noninflammatory disorders of ovary, Fallopian tube and broad ligament	N83	1.000 (0.999, 1.001)	0.59889249
IRF3	Diagnoses - main ICD10: N84 Polyp of female genital tract	N84	1.000 (0.998, 1.001)	0.57359452
IRF3	Diagnoses - main ICD10: N85 Other noninflammatory disorders of uterus, except cervix	N85	1.000 (1.000, 1.001)	0.48756769

IRF3	Diagnoses - main ICD10: N87 Dysplasia of cervix uteri	N87	1.000 (0.999, 1.001)	0.935486298
IRF3	Diagnoses - main ICD10: N90 Other noninflammatory disorders of vulva and perineum	N90	1.000 (0.999, 1.001)	0.92127428
IRF3	Diagnoses - main ICD10: N93 Other abnormal uterine and vaginal bleeding	N93	0.999 (0.999, 1.000)	0.211253616
IRF3	Diagnoses - main ICD10: N94 Pain and other conditions associated with female genital organs and menstrual cycle	N94	1.000 (1.000, 1.001)	0.154494427
IRF3	Diagnoses - main ICD10: N95 Menopausal and other perimenopausal disorders	N95	1.000 (0.998, 1.001)	0.8454584
IRF3	Diagnoses - main ICD10: O02 Other abnormal products of conception	O02	1.000 (1.000, 1.001)	0.372652075
IRF3	Diagnoses - main ICD10: O03 Spontaneous abortion	O03	1.001 (1.000, 1.001)	0.069781838
IRF3	Diagnoses - main ICD10: O26 Maternal care for other conditions predominantly related to pregnancy	O26	1.001 (1.000, 1.001)	0.045043667
IRF3	Diagnoses - main ICD10: O36 Maternal care for other known or suspected foetal problems	O36	1.000 (1.000, 1.001)	0.2248637
IRF3	Diagnoses - main ICD10: O63 Long labour	O63	1.000 (1.000, 1.001)	0.174214592
IRF3	Diagnoses - main ICD10: O68 Labour and delivery complicated by foetal stress [distress]	O68	1.001 (1.000, 1.001)	0.055217222
IRF3	Diagnoses - main ICD10: O70 Perineal laceration during delivery	O70	1.000 (0.999, 1.001)	0.480117835
IRF3	Diagnoses - main ICD10: O80 Single spontaneous delivery	O80	1.000 (0.999, 1.001)	0.733981504
IRF3	Diagnoses - main ICD10: R00 Abnormalities of heart beat	R00	1.000 (0.999, 1.001)	0.539140585
IRF3	Diagnoses - main ICD10: R06 Abnormalities of breathing	R06	0.999 (0.998, 1.001)	0.391266175
IRF3	Diagnoses - main ICD10: R19 Other symptoms and signs involving the digestive system and abdomen	R19	1.000 (0.998, 1.001)	0.750057823
IRF3	Diagnoses - main ICD10: R22 Localised swelling, mass and lump of skin and subcutaneous tissue	R22	0.999 (0.998, 1.000)	0.004174309
IRF3	Diagnoses - main ICD10: R39 Other symptoms and signs involving the urinary system	R39	1.000 (0.999, 1.001)	0.73088378
IRF3	Diagnoses - main ICD10: R50 Fever of unknown origin	R50	1.000 (1.000, 1.001)	0.318869247
IRF3	Diagnoses - main ICD10: R56 Convulsions, not elsewhere classified	R56	0.999 (0.999, 1.000)	0.049314667
IRF3	Diagnoses - main ICD10: R59 Enlarged lymph nodes	R59	1.000 (1.000, 1.001)	0.597793437
IRF3	Diagnoses - main ICD10: R63 Symptoms and signs concerning food and fluid intake	R63	1.000 (0.999, 1.000)	0.29760087
IRF3	Diagnoses - main ICD10: R79 Other abnormal findings of blood chemistry	R79	1.000 (0.999, 1.001)	0.491982462
IRF3	Diagnoses - main ICD10: R87 Abnormal findings in specimens from female genital organs	R87	1.000 (0.999, 1.000)	0.699992455
IRF3	Diagnoses - main ICD10: R93 Abnormal findings on diagnostic imaging of other body structures	R93	1.000 (1.000, 1.001)	0.270595658
IRF3	Diagnoses - main ICD10: S01 Open wound of head	S01	1.001 (1.000, 1.002)	0.009657708
IRF3	Diagnoses - main ICD10: S02 Fracture of skull and facial bones	S02	1.000 (0.999, 1.001)	0.872460635
IRF3	Diagnoses - main ICD10: S42 Fracture of shoulder and upper arm	S42	1.000 (1.000, 1.001)	0.332422438
IRF3	Diagnoses - main ICD10: S61 Open wound of wrist and hand	S61	1.000 (0.999, 1.001)	0.909863092
IRF3	Diagnoses - main ICD10: S62 Fracture at wrist and hand level	S62	1.000 (0.999, 1.000)	0.241020599
IRF3	Diagnoses - main ICD10: S72 Fracture of femur	S72	1.000 (1.000, 1.001)	0.48597948
IRF3	Diagnoses - main ICD10: S82 Fracture of lower leg, including ankle	S82	1.000 (0.999, 1.001)	0.752212541
IRF3	Diagnoses - main ICD10: T39 Poisoning by nonopioid analgesics, antipyretics and antirheumatics	T39	1.000 (1.000, 1.001)	0.3541762
IRF3	Diagnoses - main ICD10: T81 Complications of procedures, not elsewhere classified	T81	1.001 (1.000, 1.003)	0.057620968
IRF3	Diagnoses - main ICD10: T82 Complications of cardiac and vascular prosthetic devices, implants and grafts	T82	1.000 (1.000, 1.001)	0.203333231
IRF3	Diagnoses - main ICD10: T85 Complications of other internal prosthetic devices, implants and grafts	T85	1.000 (0.999, 1.001)	0.681808233
IRF3	Diagnoses - main ICD10: Z01 Other special examinations and investigations of persons without complaint or reporte	Z01	1.001 (1.000, 1.001)	0.104508974
IRF3	Diagnoses - main ICD10: Z03 Medical observation and evaluation for suspected diseases and conditions	Z03	1.000 (0.999, 1.001)	0.950932372
IRF3	Diagnoses - main ICD10: Z08 Follow-up examination after treatment for malignant neoplasm	Z08	1.000 (0.999, 1.002)	0.441224086
IRF3	Diagnoses - main ICD10: Z12 Special screening examination for neoplasms	Z12	1.000 (0.999, 1.001)	0.625042499

IRF3	Diagnoses - main ICD10: Z13 Special screening examination for other diseases and disorders	Z13	1.000 (0.999, 1.001)	0.652091012
IRF3	Diagnoses - main ICD10: Z30 Contraceptive management	Z30	1.000 (0.999, 1.001)	0.910516432
IRF3	Diagnoses - main ICD10: Z42 Follow-up care involving plastic surgery	Z42	1.001 (1.000, 1.001)	0.125754476
IRF3	Diagnoses - main ICD10: Z43 Attention to artificial openings	Z43	1.001 (1.000, 1.001)	0.077704857
IRF3	Diagnoses - main ICD10: Z45 Adjustment and management of implanted device	Z45	1.000 (0.999, 1.001)	0.936236037
IRF3	Diagnoses - main ICD10: Z46 Fitting and adjustment of other devices	Z46	0.999 (0.998, 1.000)	0.122997842
IRF3	Diagnoses - main ICD10: Z53 Persons encountering health services for specifie procedures, not carried out	Z53	1.000 (0.999, 1.001)	0.833610072

Table S9 Phenome-wide Mendelian Randomization analysis to reveal potential on-target side effects of LUM

protein	disease	ICD-10 code	OR (95%CI)	P value
LUM	Underlying (primary) cause of death: ICD10: E85.4 Organ-limited amyloidosis	E85.4	0.997 (0.992, 1.002)	0.183539946
LUM	Underlying (primary) cause of death: ICD10: J84.1 Other interstitial pulmonary diseases with fibrosis	J84.1	1.009 (0.991, 1.027)	0.322522111
LUM	Diagnoses - main ICD10: B37 Candidiasis	B37	1.000 (0.999, 1.000)	0.571637479
LUM	Diagnoses - main ICD10: C44 Other malignant neoplasms of skin	C44	1.000 (0.998, 1.003)	0.74896819
LUM	Diagnoses - main ICD10: C50 Malignant neoplasm of breast	C50	1.000 (0.997, 1.003)	0.864689364
LUM	Diagnoses - main ICD10: C61 Malignant neoplasm of prostate	C61	1.001 (0.999, 1.003)	0.536768403
LUM	Diagnoses - main ICD10: D12 Benign neoplasm of colon rectum anus and anal canal	D12	1.000 (0.997, 1.003)	0.861010389
LUM	Diagnoses - main ICD10: D25 Leiomyoma of uterus	D25	1.002 (0.999, 1.005)	0.19587939
LUM	Diagnoses - main ICD10: E03 Other hypothyroidism	E03	1.000 (1.000, 1.000)	0.874648824
LUM	Diagnoses - main ICD10: E04 Other non-toxic goitre	E04	0.999 (0.998, 1.000)	0.12800527
LUM	Diagnoses - main ICD10: F31 Bipolar affective disorder	F31	1.001 (1.000, 1.002)	0.02665225
LUM	Diagnoses - main ICD10: F43 Reaction to severe stress and adjustment disorders	F43	1.000 (1.000, 1.001)	0.77107213
LUM	Diagnoses - main ICD10: G47 Sleep disorders	G47	1.001 (0.999, 1.003)	0.3189732
LUM	Diagnoses - main ICD10: G56 Mononeuropathies of upper limb	G56	0.999 (0.996, 1.002)	0.582322266
LUM	Diagnoses - main ICD10: H25 Senile cataract	H25	1.000 (0.997, 1.002)	0.756589592
LUM	Diagnoses - main ICD10: H26 Other cataract	H26	0.999 (0.996, 1.003)	0.729794457
LUM	Diagnoses - main ICD10: I10 Essential (primary) hypertension	I10	1.000 (0.999, 1.001)	0.4200396
LUM	Diagnoses - main ICD10: I20 Angina pectoris	I20	1.001 (0.998, 1.004)	0.476760563
LUM	Diagnoses - main ICD10: I21 Acute myocardial infarction	I21	1.001 (0.999, 1.004)	0.344587337
LUM	Diagnoses - main ICD10: I25 Chronic ischaemic heart disease	I25	1.004 (1.001, 1.008)	0.025801705
LUM	Diagnoses - main ICD10: I30 Acute pericarditis	I30	1.000 (1.000, 1.000)	0.993820694
LUM	Diagnoses - main ICD10: I48 Atrial fibrillation and flutter	I48	0.999 (0.997, 1.002)	0.691712892
LUM	Diagnoses - main ICD10: I80 Phlebitis and thrombophlebitis	I80	1.001 (0.999, 1.002)	0.386215661
LUM	Diagnoses - main ICD10: I84 Haemorrhoids	I84	0.999 (0.995, 1.003)	0.537964277
LUM	Diagnoses - main ICD10: J22 Unspecified acute lower respiratory infection	J22	1.001 (0.999, 1.002)	0.59839381
LUM	Diagnoses - main ICD10: J33 Nasal polyp	J33	0.999 (0.997, 1.001)	0.305260218
LUM	Diagnoses - main ICD10: J34 Other disorders of nose and nasal sinuses	J34	1.000 (0.998, 1.003)	0.759333627
LUM	Diagnoses - main ICD10: J44 Other chronic obstructive pulmonary disease	J44	1.001 (0.999, 1.002)	0.273652548
LUM	Diagnoses - main ICD10: K20 Oesophagitis	K20	0.999 (0.996, 1.001)	0.33468583
LUM	Diagnoses - main ICD10: K21 Gastro-oesophageal reflux disease	K21	1.000 (0.996, 1.003)	0.844181241
LUM	Diagnoses - main ICD10: K22 Other diseases of oesophagus	K22	1.000 (0.998, 1.003)	0.864204744
LUM	Diagnoses - main ICD10: K29 Gastritis and duodenitis	K29	0.999 (0.995, 1.002)	0.48803756
LUM	Diagnoses - main ICD10: K35 Acute appendicitis	K35	1.000 (0.999, 1.002)	0.73329999
LUM	Diagnoses - main ICD10: K40 Inguinal hernia	K40	1.003 (0.999, 1.007)	0.173659217
LUM	Diagnoses - main ICD10: K43 Ventral hernia	K43	1.000 (0.998, 1.001)	0.761559342

LUM	Diagnoses - main ICD10: K44 Diaphragmatic hernia	K44	1.002 (0.999, 1.005)	0.190818913
LUM	Diagnoses - main ICD10: K50 Crohn's disease [regional enteritis]	K50	0.999 (0.998, 1.000)	0.133353308
LUM	Diagnoses - main ICD10: K51 Ulcerative colitis	K51	1.000 (0.998, 1.002)	0.942010108
LUM	Diagnoses - main ICD10: K52 Other non-infective gastro-enteritis and colitis	K52	1.001 (0.998, 1.005)	0.463069103
LUM	Diagnoses - main ICD10: K57 Diverticular disease of intestine	K57	0.998 (0.995, 1.002)	0.305746217
LUM	Diagnoses - main ICD10: K60 Fissure and fistula of anal and rectal regions	K60	0.998 (0.997, 1.000)	0.060925198
LUM	Diagnoses - main ICD10: K62 Other diseases of anus and rectum	K62	0.996 (0.992, 1.000)	0.045272191
LUM	Diagnoses - main ICD10: K76 Other diseases of liver	K76	1.000 (0.999, 1.000)	0.248917286
LUM	Diagnoses - main ICD10: K80 Cholelithiasis	K80	1.002 (0.998, 1.005)	0.302317877
LUM	Diagnoses - main ICD10: L03 Cellulitis	L03	0.999 (0.996, 1.001)	0.244828463
LUM	Diagnoses - main ICD10: M10 Gout	M10	1.000 (0.999, 1.000)	0.567523735
LUM	Diagnoses - main ICD10: M16 Coxarthrosis [arthrosis of hip]	M16	1.001 (0.998, 1.004)	0.500223113
LUM	Diagnoses - main ICD10: M17 Gonarthrosis [arthrosis of knee]	M17	0.999 (0.996, 1.003)	0.717107819
LUM	Diagnoses - main ICD10: M20 Acquired deformities of fingers and toes	M20	0.998 (0.995, 1.001)	0.128557358
LUM	Diagnoses - main ICD10: M21 Other acquired deformities of limbs	M21	1.000 (0.999, 1.001)	0.563122038
LUM	Diagnoses - main ICD10: M23 Internal derangement of knee	M23	1.000 (0.996, 1.003)	0.876169052
LUM	Diagnoses - main ICD10: M24 Other specific joint derangements	M24	1.000 (0.999, 1.001)	0.842800896
LUM	Diagnoses - main ICD10: M25 Other joint disorders not elsewhere classified	M25	1.000 (0.997, 1.003)	0.901050011
LUM	Diagnoses - main ICD10: M54 Dorsalgia	M54	0.999 (0.996, 1.002)	0.553939354
LUM	Diagnoses - main ICD10: M67 Other disorders of synovium and tendon	M67	1.001 (0.999, 1.002)	0.51039334
LUM	Diagnoses - main ICD10: M70 Soft tissue disorders related to use overuse and pressure	M70	1.000 (0.999, 1.001)	0.848683685
LUM	Diagnoses - main ICD10: M72 Fibroblastic disorders	M72	0.999 (0.998, 1.001)	0.585871885
LUM	Diagnoses - main ICD10: N19 Unspecified renal failure	N19	1.000 (0.999, 1.000)	0.884951887
LUM	Diagnoses - main ICD10: N20 Calculus of kidney and ureter	N20	1.000 (0.998, 1.002)	0.796581643
LUM	Diagnoses - main ICD10: N32 Other disorders of bladder	N32	1.000 (0.997, 1.002)	0.807797092
LUM	Diagnoses - main ICD10: N40 Hyperplasia of prostate	N40	0.999 (0.996, 1.001)	0.348222293
LUM	Diagnoses - main ICD10: N81 Female genital prolapse	N81	1.003 (1.000, 1.006)	0.075247795
LUM	Diagnoses - main ICD10: N92 Excessive frequent and irregular menstruation	N92	0.998 (0.995, 1.002)	0.312656768
LUM	Diagnoses - main ICD10: O75 Other complications of labour and delivery not elsewhere classified	O75	1.000 (1.000, 1.001)	0.27892096
LUM	Diagnoses - main ICD10: R04 Haemorrhage from respiratory passages	R04	0.999 (0.998, 1.001)	0.53999375
LUM	Diagnoses - main ICD10: R07 Pain in throat and chest	R07	1.000 (0.994, 1.005)	0.860897085
LUM	Diagnoses - main ICD10: R10 Abdominal and pelvic pain	R10	1.002 (0.997, 1.007)	0.540484787
LUM	Diagnoses - main ICD10: R11 Nausea and vomiting	R11	0.998 (0.997, 1.000)	0.050916433
LUM	Diagnoses - main ICD10: R14 Flatulence and related conditions	R14	1.000 (0.999, 1.000)	0.588596065
LUM	Diagnoses - main ICD10: R31 Unspecified haematuria	R31	0.999 (0.995, 1.003)	0.59832302
LUM	Diagnoses - main ICD10: R35 Polyuria	R35	1.001 (0.999, 1.003)	0.191200295
LUM	Diagnoses - main ICD10: R55 Syncope and collapse	R55	1.000 (0.998, 1.003)	0.849097666

LUM	Diagnoses - main ICD10: R69 Unknown and unspecified causes of morbidity	R69	1.002 (0.999, 1.006)	0.247914358
LUM	Diagnoses - main ICD10: S09 Other and unspecified injuries of head	S09	1.000 (0.999, 1.001)	0.788394719
LUM	Diagnoses - main ICD10: S52 Fracture of forearm	S52	1.000 (0.998, 1.003)	0.761467931
LUM	Diagnoses - main ICD10: S66 Injury of muscle and tendon at wrist and hand level	S66	1.000 (0.999, 1.001)	0.429103922
LUM	Diagnoses - main ICD10: S76 Injury of muscle and tendon at hip and thigh level	S76	1.000 (0.999, 1.000)	0.749260269
LUM	Diagnoses - main ICD10: T84 Complications of internal orthopaedic prosthetic devices implants and g	T84	1.000 (0.998, 1.002)	0.703185208
LUM	Diagnoses - main ICD10: Z09 Follow-up examination after treatment for conditions other than malign	Z09	0.998 (0.995, 1.001)	0.305683592
LUM	Diagnoses - main ICD10: Z47 Other orthopaedic follow-up care	Z47	0.999 (0.997, 1.001)	0.381326987
LUM	Diagnoses - main ICD10: Z80 Family history of malignant neoplasm	Z80	1.000 (0.999, 1.001)	0.554926649
LUM	Diagnoses - main ICD10: R07.4 Chest pain, unspecified	R07.4	0.998 (0.994, 1.002)	0.343200912
LUM	Diagnoses - secondary ICD10: E78.0 Pure hypercholesterolaemia	E78.0	1.000 (0.995, 1.004)	0.861701981
LUM	Diagnoses - secondary ICD10: J45.9 Asthma, unspecified	J45.9	1.000 (0.996, 1.005)	0.887158945
LUM	Underlying (primary) cause of death: ICD10: C34.9 Bronchus or lung, unspecified	C34.9	0.998 (0.951, 1.048)	0.934770947
LUM	Diagnoses - main ICD10: A41 Other septicaemia	A41	1.001 (1.000, 1.003)	0.08662662
LUM	Diagnoses - main ICD10: C18 Malignant neoplasm of colon	C18	1.000 (0.998, 1.002)	0.846456396
LUM	Diagnoses - main ICD10: C34 Malignant neoplasm of bronchus and lung	C34	0.999 (0.998, 1.001)	0.44729106
LUM	Diagnoses - main ICD10: C43 Malignant melanoma of skin	C43	1.000 (0.998, 1.002)	0.892901405
LUM	Diagnoses - main ICD10: C67 Malignant neoplasm of bladder	C67	1.000 (0.999, 1.002)	0.683611487
LUM	Diagnoses - main ICD10: C78 Secondary malignant neoplasm of respiratory and digestive organs	C78	0.999 (0.998, 1.001)	0.283766387
LUM	Diagnoses - main ICD10: C79 Secondary malignant neoplasm of other sites	C79	0.999 (0.998, 1.001)	0.308755598
LUM	Diagnoses - main ICD10: D05 Carcinoma in situ of breast	D05	1.001 (1.000, 1.003)	0.071837421
LUM	Diagnoses - main ICD10: D17 Benign lipomatous neoplasm	D17	1.000 (0.998, 1.003)	0.740182249
LUM	Diagnoses - main ICD10: D22 Melanocytic naevi	D22	1.001 (0.999, 1.004)	0.228165435
LUM	Diagnoses - main ICD10: D23 Other benign neoplasms of skin	D23	1.000 (0.998, 1.003)	0.767738729
LUM	Diagnoses - main ICD10: D50 Iron deficiency anaemia	D50	0.999 (0.996, 1.001)	0.296791568
LUM	Diagnoses - main ICD10: D64 Other anaemias	D64	0.999 (0.997, 1.002)	0.536063018
LUM	Diagnoses - main ICD10: G43 Migraine	G43	1.000 (0.999, 1.002)	0.499091025
LUM	Diagnoses - main ICD10: G45 Transient cerebral ischaemic attacks and related syndromes	G45	1.000 (0.998, 1.002)	0.891221421
LUM	Diagnoses - main ICD10: G57 Mononeuropathies of lower limb	G57	1.000 (0.998, 1.001)	0.736038407
LUM	Diagnoses - main ICD10: H00 Hordeolum and chalazion	H00	1.001 (0.999, 1.002)	0.248599737
LUM	Diagnoses - main ICD10: H02 Other disorders of eyelid	H02	0.997 (0.995, 1.000)	0.053241258
LUM	Diagnoses - main ICD10: H04 Disorders of lachrymal system	H04	1.000 (0.998, 1.001)	0.868772943
LUM	Diagnoses - main ICD10: H33 Retinal detachments and breaks	H33	1.001 (0.999, 1.003)	0.264161955
LUM	Diagnoses - main ICD10: H35 Other retinal disorders	H35	1.000 (0.998, 1.001)	0.623564742
LUM	Diagnoses - main ICD10: H40 Glaucoma	H40	1.001 (0.999, 1.002)	0.506297319
LUM	Diagnoses - main ICD10: I26 Pulmonary embolism	I26	1.001 (0.999, 1.003)	0.366383121
LUM	Diagnoses - main ICD10: I47 Paroxysmal tachycardia	I47	0.997 (0.996, 0.999)	0.001482012

LUM	Diagnoses - main ICD10: I50 Heart failure	I50	1.000 (0.998, 1.001)	0.65777575
LUM	Diagnoses - main ICD10: I63 Cerebral infarction	I63	1.000 (0.998, 1.002)	0.720067446
LUM	Diagnoses - main ICD10: J18 Pneumonia, organism unspecified	J18	1.001 (0.999, 1.004)	0.323359943
LUM	Diagnoses - main ICD10: J32 Chronic sinusitis	J32	1.000 (0.998, 1.001)	0.795700302
LUM	Diagnoses - main ICD10: J38 Diseases of vocal cords and larynx, not elsewhere classified	J38	1.001 (1.000, 1.003)	0.119724731
LUM	Diagnoses - main ICD10: J45 Asthma	J45	0.999 (0.998, 1.001)	0.467481656
LUM	Diagnoses - main ICD10: K01 Embedded and impacted teeth	K01	1.000 (0.998, 1.001)	0.804152004
LUM	Diagnoses - main ICD10: K02 Dental caries	K02	1.000 (0.998, 1.002)	0.834572684
LUM	Diagnoses - main ICD10: K04 Diseases of pulp and periapical tissues	K04	1.001 (0.999, 1.002)	0.417782279
LUM	Diagnoses - main ICD10: K08 Other disorders of teeth and supporting structures	K08	1.001 (0.999, 1.003)	0.265542876
LUM	Diagnoses - main ICD10: K13 Other diseases of lip and oral mucosa	K13	1.001 (0.999, 1.003)	0.351911231
LUM	Diagnoses - main ICD10: K25 Gastric ulcer	K25	1.000 (0.998, 1.002)	0.768180877
LUM	Diagnoses - main ICD10: K26 Duodenal ulcer	K26	0.999 (0.997, 1.000)	0.118182911
LUM	Diagnoses - main ICD10: K31 Other diseases of stomach and duodenum	K31	1.000 (0.998, 1.003)	0.646711097
LUM	Diagnoses - main ICD10: K42 Umbilical hernia	K42	1.000 (0.998, 1.002)	0.885371518
LUM	Diagnoses - main ICD10: K56 Paralytic ileus and intestinal obstruction without hernia	K56	1.001 (0.999, 1.002)	0.500700031
LUM	Diagnoses - main ICD10: K58 Irritable bowel syndrome	K58	0.999 (0.997, 1.000)	0.069960517
LUM	Diagnoses - main ICD10: K59 Other functional intestinal disorders	K59	1.002 (0.999, 1.004)	0.17837906
LUM	Diagnoses - main ICD10: K61 Abscess of anal and rectal regions	K61	1.000 (0.998, 1.001)	0.731529903
LUM	Diagnoses - main ICD10: K63 Other diseases of intestine	K63	1.001 (0.997, 1.005)	0.631290545
LUM	Diagnoses - main ICD10: K81 Cholecystitis	K81	1.000 (0.998, 1.002)	0.895999643
LUM	Diagnoses - main ICD10: K92 Other diseases of digestive system	K92	0.998 (0.996, 1.001)	0.293373647
LUM	Diagnoses - main ICD10: L02 Cutaneous abscess, furuncle and carbuncle	L02	1.000 (0.998, 1.001)	0.678610145
LUM	Diagnoses - main ICD10: L57 Skin changes due to chronic exposure to nonionising radiation	L57	1.000 (0.999, 1.002)	0.674528391
LUM	Diagnoses - main ICD10: L72 Follicular cysts of skin and subcutaneous tissue	L72	0.998 (0.994, 1.001)	0.134789503
LUM	Diagnoses - main ICD10: L90 Atrophic disorders of skin	L90	0.998 (0.996, 0.999)	0.009715219
LUM	Diagnoses - main ICD10: L98 Other disorders of skin and subcutaneous tissue, not elsewhere classified	L98	1.000 (0.997, 1.002)	0.704657853
LUM	Diagnoses - main ICD10: M06 Other rheumatoid arthritis	M06	1.001 (0.999, 1.002)	0.344434014
LUM	Diagnoses - main ICD10: M13 Other arthritis	M13	1.001 (1.000, 1.002)	0.148723696
LUM	Diagnoses - main ICD10: M15 Polyarthrosis	M15	1.001 (0.999, 1.002)	0.455941118
LUM	Diagnoses - main ICD10: M19 Other arthrosis	M19	1.001 (0.999, 1.004)	0.39651748
LUM	Diagnoses - main ICD10: M47 Spondylosis	M47	0.999 (0.997, 1.001)	0.415341071
LUM	Diagnoses - main ICD10: M48 Other spondylopathies	M48	1.001 (0.999, 1.003)	0.368746478
LUM	Diagnoses - main ICD10: M51 Other intervertebral disk disorders	M51	0.998 (0.995, 1.001)	0.17257369
LUM	Diagnoses - main ICD10: M65 Synovitis and tenosynovitis	M65	1.000 (0.998, 1.002)	0.926403005
LUM	Diagnoses - main ICD10: M75 Shoulder lesions	M75	0.999 (0.996, 1.003)	0.667082573
LUM	Diagnoses - main ICD10: M79 Other soft tissue disorders, not elsewhere classified	M79	0.999 (0.996, 1.003)	0.734506012

LUM	Diagnoses - main ICD10: M84 Disorders of continuity of bone	M84	1.000 (0.999, 1.001)	0.955091234
LUM	Diagnoses - main ICD10: N13 Obstructive and reflux uropathy	N13	0.998 (0.996, 0.999)	0.008969715
LUM	Diagnoses - main ICD10: N30 Cystitis	N30	1.001 (0.999, 1.002)	0.47052715
LUM	Diagnoses - main ICD10: N31 Neuromuscular dysfunction of bladder, not elsewhere classified	N31	1.001 (0.999, 1.002)	0.234944782
LUM	Diagnoses - main ICD10: N35 Urethral stricture	N35	1.001 (0.999, 1.003)	0.428961281
LUM	Diagnoses - main ICD10: N39 Other disorders of urinary system	N39	1.001 (0.997, 1.005)	0.532796891
LUM	Diagnoses - main ICD10: N48 Other disorders of penis	N48	0.999 (0.998, 1.001)	0.34178995
LUM	Diagnoses - main ICD10: N50 Other disorders of male genital organs	N50	1.001 (0.999, 1.002)	0.419820414
LUM	Diagnoses - main ICD10: N60 Benign mammary dysplasia	N60	1.001 (0.999, 1.002)	0.355836438
LUM	Diagnoses - main ICD10: N80 Endometriosis	N80	1.000 (0.999, 1.002)	0.791463348
LUM	Diagnoses - main ICD10: N83 Noninflammatory disorders of ovary, Fallopian tube and broad ligamer	N83	1.001 (0.999, 1.003)	0.301665783
LUM	Diagnoses - main ICD10: N84 Polyp of female genital tract	N84	1.000 (0.997, 1.004)	0.853248713
LUM	Diagnoses - main ICD10: N85 Other noninflammatory disorders of uterus, except cervix	N85	1.001 (0.999, 1.002)	0.249408503
LUM	Diagnoses - main ICD10: N87 Dysplasia of cervix uteri	N87	1.000 (0.999, 1.001)	0.941643876
LUM	Diagnoses - main ICD10: N90 Other noninflammatory disorders of vulva and perineum	N90	1.000 (0.998, 1.001)	0.687578182
LUM	Diagnoses - main ICD10: N93 Other abnormal uterine and vaginal bleeding	N93	1.002 (1.000, 1.004)	0.046588267
LUM	Diagnoses - main ICD10: N94 Pain and other conditions associated with female genital organs and me	N94	1.001 (1.000, 1.002)	0.188167318
LUM	Diagnoses - main ICD10: N95 Menopausal and other perimenopausal disorders	N95	0.999 (0.996, 1.002)	0.661362483
LUM	Diagnoses - main ICD10: O02 Other abnormal products of conception	O02	0.999 (0.998, 1.000)	0.178392752
LUM	Diagnoses - main ICD10: O03 Spontaneous abortion	O03	0.999 (0.997, 1.000)	0.046683381
LUM	Diagnoses - main ICD10: O26 Maternal care for other conditions predominantly related to pregnancy	O26	0.999 (0.997, 1.000)	0.122427072
LUM	Diagnoses - main ICD10: O36 Maternal care for other known or suspected foetal problems	O36	1.000 (0.999, 1.002)	0.72513774
LUM	Diagnoses - main ICD10: O63 Long labour	O63	1.000 (0.998, 1.001)	0.499767634
LUM	Diagnoses - main ICD10: O68 Labour and delivery complicated by foetal stress [distress]	O68	1.000 (0.998, 1.001)	0.717762089
LUM	Diagnoses - main ICD10: O70 Perineal laceration during delivery	O70	0.997 (0.995, 1.000)	0.020859785
LUM	Diagnoses - main ICD10: O80 Single spontaneous delivery	O80	1.001 (0.999, 1.002)	0.343807697
LUM	Diagnoses - main ICD10: R00 Abnormalities of heart beat	R00	1.001 (0.999, 1.004)	0.158359299
LUM	Diagnoses - main ICD10: R06 Abnormalities of breathing	R06	1.000 (0.997, 1.003)	0.941517899
LUM	Diagnoses - main ICD10: R19 Other symptoms and signs involving the digestive system and abdomen	R19	0.998 (0.994, 1.002)	0.239650483
LUM	Diagnoses - main ICD10: R22 Localised swelling, mass and lump of skin and subcutaneous tissue	R22	1.000 (0.998, 1.001)	0.64296249
LUM	Diagnoses - main ICD10: R39 Other symptoms and signs involving the urinary system	R39	0.999 (0.997, 1.001)	0.344235614
LUM	Diagnoses - main ICD10: R50 Fever of unknown origin	R50	1.000 (0.999, 1.002)	0.788709872
LUM	Diagnoses - main ICD10: R56 Convulsions, not elsewhere classified	R56	1.001 (0.999, 1.002)	0.376050761
LUM	Diagnoses - main ICD10: R59 Enlarged lymph nodes	R59	1.001 (0.999, 1.002)	0.42890019
LUM	Diagnoses - main ICD10: R63 Symptoms and signs concerning food and fluid intake	R63	1.000 (0.999, 1.002)	0.554401886
LUM	Diagnoses - main ICD10: R79 Other abnormal findings of blood chemistry	R79	1.000 (0.998, 1.002)	0.878442754
LUM	Diagnoses - main ICD10: R87 Abnormal findings in specimens from female genital organs	R87	0.999 (0.997, 1.000)	0.070153816

LUM	Diagnoses - main ICD10: R93 Abnormal findings on diagnostic imaging of other body structures	R93	1.001 (0.999, 1.002)	0.47360187
LUM	Diagnoses - main ICD10: S01 Open wound of head	S01	1.000 (0.998, 1.001)	0.730850064
LUM	Diagnoses - main ICD10: S02 Fracture of skull and facial bones	S02	0.999 (0.998, 1.001)	0.235828104
LUM	Diagnoses - main ICD10: S42 Fracture of shoulder and upper arm	S42	1.001 (0.999, 1.003)	0.360024362
LUM	Diagnoses - main ICD10: S61 Open wound of wrist and hand	S61	1.001 (0.999, 1.002)	0.502671632
LUM	Diagnoses - main ICD10: S62 Fracture at wrist and hand level	S62	0.999 (0.998, 1.001)	0.485891929
LUM	Diagnoses - main ICD10: S72 Fracture of femur	S72	0.998 (0.996, 1.000)	0.013008814
LUM	Diagnoses - main ICD10: S82 Fracture of lower leg, including ankle	S82	0.999 (0.997, 1.002)	0.581770044
LUM	Diagnoses - main ICD10: T39 Poisoning by nonopioid analgesics, antipyretics and antirheumatics	T39	1.000 (0.998, 1.001)	0.680251104
LUM	Diagnoses - main ICD10: T81 Complications of procedures, not elsewhere classified	T81	1.001 (0.998, 1.004)	0.483067773
LUM	Diagnoses - main ICD10: T82 Complications of cardiac and vascular prosthetic devices, implants and g	T82	1.001 (1.000, 1.003)	0.041090182
LUM	Diagnoses - main ICD10: T85 Complications of other internal prosthetic devices, implants and grafts	T85	1.002 (1.000, 1.003)	0.051224943
LUM	Diagnoses - main ICD10: Z01 Other special examinations and investigations of persons without compl	Z01	0.999 (0.998, 1.001)	0.364276892
LUM	Diagnoses - main ICD10: Z03 Medical observation and evaluation for suspected diseases and condition	Z03	0.999 (0.996, 1.002)	0.546729412
LUM	Diagnoses - main ICD10: Z08 Follow-up examination after treatment for malignant neoplasm	Z08	1.000 (0.998, 1.003)	0.910424058
LUM	Diagnoses - main ICD10: Z12 Special screening examination for neoplasms	Z12	0.998 (0.996, 1.001)	0.257806037
LUM	Diagnoses - main ICD10: Z13 Special screening examination for other diseases and disorders	Z13	1.001 (0.999, 1.003)	0.29795248
LUM	Diagnoses - main ICD10: Z30 Contraceptive management	Z30	1.000 (0.997, 1.003)	0.938283317
LUM	Diagnoses - main ICD10: Z42 Follow-up care involving plastic surgery	Z42	1.001 (0.999, 1.003)	0.317159353
LUM	Diagnoses - main ICD10: Z43 Attention to artificial openings	Z43	1.000 (0.998, 1.001)	0.827041188
LUM	Diagnoses - main ICD10: Z45 Adjustment and management of implanted device	Z45	1.002 (1.000, 1.003)	0.126492312
LUM	Diagnoses - main ICD10: Z46 Fitting and adjustment of other devices	Z46	0.998 (0.996, 1.001)	0.151229507
LUM	Diagnoses - main ICD10: Z53 Persons encountering health services for specifie procedures, not carried	Z53	1.002 (1.000, 1.003)	0.069422732

Table S10 Phenome-wide Mendelian Randomization analysis to reveal potential on-target side effects of POSTN

protein	disease	ICD-10 code	OR (95%CI)	P value
POSTN	Underlying (primary) cause of death: ICD10: E85.4 Organ-limited amyloidosis	E85.4	1.003 (0.999, 1.007)	0.171069941
POSTN	Underlying (primary) cause of death: ICD10: J84.1 Other interstitial pulmonary diseases with fibrosis	J84.1	1.010 (0.995, 1.026)	0.196369681
POSTN	Diagnoses - main ICD10: B37 Candidiasis	B37	1.000 (0.999, 1.000)	0.297266709
POSTN	Diagnoses - main ICD10: C44 Other malignant neoplasms of skin	C44	1.002 (1.000, 1.005)	0.049387263
POSTN	Diagnoses - main ICD10: C50 Malignant neoplasm of breast	C50	1.000 (0.998, 1.003)	0.800479489
POSTN	Diagnoses - main ICD10: C61 Malignant neoplasm of prostate	C61	1.000 (0.999, 1.002)	0.709131295
POSTN	Diagnoses - main ICD10: D12 Benign neoplasm of colon rectum anus and anal canal	D12	1.001 (0.999, 1.004)	0.382173619
POSTN	Diagnoses - main ICD10: D25 Leiomyoma of uterus	D25	1.000 (0.997, 1.002)	0.747479505
POSTN	Diagnoses - main ICD10: E03 Other hypothyroidism	E03	1.000 (1.000, 1.001)	0.060250367
POSTN	Diagnoses - main ICD10: E04 Other non-toxic goitre	E04	1.000 (0.999, 1.001)	0.847934656
POSTN	Diagnoses - main ICD10: F31 Bipolar affective disorder	F31	1.000 (1.000, 1.001)	0.68642373
POSTN	Diagnoses - main ICD10: F43 Reaction to severe stress and adjustment disorders	F43	1.000 (0.999, 1.000)	0.845664482
POSTN	Diagnoses - main ICD10: G47 Sleep disorders	G47	1.000 (0.999, 1.002)	0.844958666
POSTN	Diagnoses - main ICD10: G56 Mononeuropathies of upper limb	G56	1.001 (0.998, 1.003)	0.702024957
POSTN	Diagnoses - main ICD10: H25 Senile cataract	H25	0.999 (0.997, 1.001)	0.257805089
POSTN	Diagnoses - main ICD10: H26 Other cataract	H26	1.001 (0.998, 1.003)	0.586527874
POSTN	Diagnoses - main ICD10: I10 Essential (primary) hypertension	I10	1.000 (0.999, 1.001)	0.814723276
POSTN	Diagnoses - main ICD10: I20 Angina pectoris	I20	1.000 (0.997, 1.002)	0.796142691
POSTN	Diagnoses - main ICD10: I21 Acute myocardial infarction	I21	1.001 (0.999, 1.003)	0.374267234
POSTN	Diagnoses - main ICD10: I25 Chronic ischaemic heart disease	I25	1.000 (0.997, 1.004)	0.86553609
POSTN	Diagnoses - main ICD10: I30 Acute pericarditis	I30	1.000 (1.000, 1.000)	0.702992417
POSTN	Diagnoses - main ICD10: I48 Atrial fibrillation and flutter	I48	0.999 (0.997, 1.002)	0.591187351
POSTN	Diagnoses - main ICD10: I80 Phlebitis and thrombophlebitis	I80	0.998 (0.997, 1.000)	0.024509248
POSTN	Diagnoses - main ICD10: I84 Haemorrhoids	I84	0.998 (0.995, 1.001)	0.146246383
POSTN	Diagnoses - main ICD10: J22 Unspecified acute lower respiratory infection	J22	0.999 (0.997, 1.001)	0.187114693
POSTN	Diagnoses - main ICD10: J33 Nasal polyp	J33	1.000 (0.998, 1.001)	0.715124049
POSTN	Diagnoses - main ICD10: J34 Other disorders of nose and nasal sinuses	J34	1.001 (0.998, 1.003)	0.619075665
POSTN	Diagnoses - main ICD10: J44 Other chronic obstructive pulmonary disease	J44	1.001 (1.000, 1.002)	0.15714613
POSTN	Diagnoses - main ICD10: K20 Oesophagitis	K20	1.001 (0.999, 1.003)	0.445009784
POSTN	Diagnoses - main ICD10: K21 Gastro-oesophageal reflux disease	K21	0.998 (0.995, 1.001)	0.242937218
POSTN	Diagnoses - main ICD10: K22 Other diseases of oesophagus	K22	0.999 (0.997, 1.001)	0.519076367
POSTN	Diagnoses - main ICD10: K29 Gastritis and duodenitis	K29	1.001 (0.998, 1.004)	0.504764306
POSTN	Diagnoses - main ICD10: K35 Acute appendicitis	K35	1.000 (0.998, 1.001)	0.606932253
POSTN	Diagnoses - main ICD10: K40 Inguinal hernia	K40	1.003 (1.000, 1.006)	0.069605319
POSTN	Diagnoses - main ICD10: K43 Ventral hernia	K43	1.000 (0.999, 1.002)	0.759046942

POSTN	Diagnoses - main ICD10: K44 Diaphragmatic hernia	K44	1.001 (0.999, 1.004)	0.332446831
POSTN	Diagnoses - main ICD10: K50 Crohn's disease [regional enteritis]	K50	0.999 (0.998, 1.000)	0.131092868
POSTN	Diagnoses - main ICD10: K51 Ulcerative colitis	K51	0.999 (0.998, 1.001)	0.317656101
POSTN	Diagnoses - main ICD10: K52 Other non-infective gastro-enteritis and colitis	K52	1.002 (1.000, 1.005)	0.09902844
POSTN	Diagnoses - main ICD10: K57 Diverticular disease of intestine	K57	1.002 (0.999, 1.005)	0.20768878
POSTN	Diagnoses - main ICD10: K60 Fissure and fistula of anal and rectal regions	K60	1.000 (0.999, 1.002)	0.856810913
POSTN	Diagnoses - main ICD10: K62 Other diseases of anus and rectum	K62	0.999 (0.996, 1.003)	0.677092945
POSTN	Diagnoses - main ICD10: K76 Other diseases of liver	K76	1.000 (0.999, 1.001)	0.713805909
POSTN	Diagnoses - main ICD10: K80 Cholelithiasis	K80	1.001 (0.998, 1.004)	0.647361472
POSTN	Diagnoses - main ICD10: L03 Cellulitis	L03	1.000 (0.998, 1.002)	0.791817822
POSTN	Diagnoses - main ICD10: M10 Gout	M10	1.000 (0.999, 1.000)	0.116846382
POSTN	Diagnoses - main ICD10: M16 Coxarthrosis [arthrosis of hip]	M16	1.001 (0.998, 1.003)	0.645000848
POSTN	Diagnoses - main ICD10: M17 Gonarthrosis [arthrosis of knee]	M17	0.997 (0.995, 1.000)	0.072040124
POSTN	Diagnoses - main ICD10: M20 Acquired deformities of fingers and toes	M20	1.000 (0.998, 1.003)	0.915292429
POSTN	Diagnoses - main ICD10: M21 Other acquired deformities of limbs	M21	1.000 (0.999, 1.001)	0.69154407
POSTN	Diagnoses - main ICD10: M23 Internal derangement of knee	M23	0.998 (0.995, 1.001)	0.148051753
POSTN	Diagnoses - main ICD10: M24 Other specific joint derangements	M24	1.000 (0.999, 1.001)	0.578086295
POSTN	Diagnoses - main ICD10: M25 Other joint disorders not elsewhere classified	M25	1.000 (0.997, 1.002)	0.832074546
POSTN	Diagnoses - main ICD10: M54 Dorsalgia	M54	1.001 (0.998, 1.004)	0.525401279
POSTN	Diagnoses - main ICD10: M67 Other disorders of synovium and tendon	M67	1.001 (0.999, 1.002)	0.203530599
POSTN	Diagnoses - main ICD10: M70 Soft tissue disorders related to use overuse and pressure	M70	1.000 (0.999, 1.001)	0.908542754
POSTN	Diagnoses - main ICD10: M72 Fibroblastic disorders	M72	1.000 (0.999, 1.002)	0.79393841
POSTN	Diagnoses - main ICD10: N19 Unspecified renal failure	N19	1.000 (0.999, 1.000)	0.253270387
POSTN	Diagnoses - main ICD10: N20 Calculus of kidney and ureter	N20	0.999 (0.998, 1.001)	0.509602432
POSTN	Diagnoses - main ICD10: N32 Other disorders of bladder	N32	1.001 (0.999, 1.002)	0.517540819
POSTN	Diagnoses - main ICD10: N40 Hyperplasia of prostate	N40	1.001 (0.999, 1.003)	0.505433015
POSTN	Diagnoses - main ICD10: N81 Female genital prolapse	N81	1.000 (0.998, 1.003)	0.735843419
POSTN	Diagnoses - main ICD10: N92 Excessive frequent and irregular menstruation	N92	0.999 (0.996, 1.002)	0.649211359
POSTN	Diagnoses - main ICD10: O75 Other complications of labour and delivery not elsewhere classified	O75	1.000 (0.999, 1.000)	0.856435379
POSTN	Diagnoses - main ICD10: R04 Haemorrhage from respiratory passages	R04	1.000 (0.998, 1.001)	0.673318555
POSTN	Diagnoses - main ICD10: R07 Pain in throat and chest	R07	0.999 (0.994, 1.003)	0.582281606
POSTN	Diagnoses - main ICD10: R10 Abdominal and pelvic pain	R10	1.001 (0.997, 1.005)	0.74601136
POSTN	Diagnoses - main ICD10: R11 Nausea and vomiting	R11	1.000 (0.999, 1.002)	0.67231314
POSTN	Diagnoses - main ICD10: R14 Flatulence and related conditions	R14	1.000 (1.000, 1.001)	0.947190378
POSTN	Diagnoses - main ICD10: R31 Unspecified haematuria	R31	0.997 (0.994, 1.000)	0.037937996
POSTN	Diagnoses - main ICD10: R35 Polyuria	R35	1.000 (0.999, 1.001)	0.813709955
POSTN	Diagnoses - main ICD10: R55 Syncope and collapse	R55	1.000 (0.998, 1.002)	0.895237424

POSTN	Diagnoses - main ICD10: R69 Unknown and unspecified causes of morbidity	R69	0.999 (0.996, 1.002)	0.638197811
POSTN	Diagnoses - main ICD10: S09 Other and unspecified injuries of head	S09	1.000 (0.998, 1.001)	0.411589182
POSTN	Diagnoses - main ICD10: S52 Fracture of forearm	S52	1.000 (0.998, 1.002)	0.710415166
POSTN	Diagnoses - main ICD10: S66 Injury of muscle and tendon at wrist and hand level	S66	1.000 (0.999, 1.001)	0.803384932
POSTN	Diagnoses - main ICD10: S76 Injury of muscle and tendon at hip and thigh level	S76	1.000 (1.000, 1.001)	0.31732397
POSTN	Diagnoses - main ICD10: T84 Complications of internal orthopaedic prosthetic devices implants and gra	T84	1.000 (0.999, 1.002)	0.597360777
POSTN	Diagnoses - main ICD10: Z09 Follow-up examination after treatment for conditions other than malignant	Z09	0.997 (0.994, 0.999)	0.010230138
POSTN	Diagnoses - main ICD10: Z47 Other orthopaedic follow-up care	Z47	1.000 (0.998, 1.002)	0.978217855
POSTN	Diagnoses - main ICD10: Z80 Family history of malignant neoplasm	Z80	1.000 (0.999, 1.000)	0.243667729
POSTN	Diagnoses - secondary ICD10: Z50.1 Other physical therapy	Z50.1	1.000 (0.999, 1.001)	0.785442374
POSTN	Diagnoses - main ICD10: Z09.0 Follow-up examination after surgery for other conditions	Z09.0	0.998 (0.997, 1.000)	0.040559554
POSTN	Diagnoses - secondary ICD10: K21.0 Gastro-oesophageal reflux disease with oesophagitis	K21.0	0.999 (0.998, 1.001)	0.399560919
POSTN	Type of cancer: ICD10: C44.3 Skin of other and unspecified parts of face	C44.3	1.001 (0.999, 1.003)	0.45784998
POSTN	Diagnoses - main ICD10: K80.0 Calculus of gallbladder with acute cholecystitis	K80.0	1.000 (0.999, 1.001)	0.784728079
POSTN	Diagnoses - secondary ICD10: R19.4 Change in bowel habit	R19.4	0.999 (0.998, 1.000)	0.167524354
POSTN	Diagnoses - secondary ICD10: R10.1 Pain localised to upper abdomen	R10.1	1.000 (0.999, 1.001)	0.971483497
POSTN	Diagnoses - secondary ICD10: Z03.8 Observation for other suspected diseases and conditions	Z03.8	1.000 (0.999, 1.002)	0.589685014
POSTN	Diagnoses - secondary ICD10: I50.1 Left ventricular failure	I50.1	0.999 (0.998, 1.000)	0.179236032
POSTN	Diagnoses - main ICD10: D12.5 Sigmoid colon	D12.5	0.999 (0.998, 1.001)	0.337864904
POSTN	Diagnoses - main ICD10: Z09.9 Follow-up examination after unspecified treatment for other conditions	Z09.9	1.000 (0.999, 1.001)	0.857881193
POSTN	Diagnoses - main ICD10: N47 Redundant prepuce, phimosis and paraphimosis	N47	1.001 (1.000, 1.002)	0.3030122
POSTN	Diagnoses - secondary ICD10: E10.9 Without complications	E10.9	1.001 (1.000, 1.002)	0.076399622
POSTN	Diagnoses - main ICD10: O70.1 Second degree perineal laceration during delivery	O70.1	0.999 (0.998, 1.001)	0.299331596
POSTN	Diagnoses - secondary ICD10: I20.0 Unstable angina	I20.0	1.001 (1.000, 1.002)	0.271336327
POSTN	Diagnoses - main ICD10: R07.4 Chest pain, unspecified	R07.4	0.999 (0.996, 1.002)	0.555359901
POSTN	Diagnoses - main ICD10: N93.9 Abnormal uterine and vaginal bleeding, unspecified	N93.9	1.000 (0.999, 1.001)	0.894788561
POSTN	Diagnoses - main ICD10: K57.3 Diverticular disease of large intestine without perforation or abscess	K57.3	1.001 (0.998, 1.003)	0.593914956
POSTN	Diagnoses - main ICD10: M54.59 Low back pain (Site unspecified)	M54.59	1.000 (0.999, 1.001)	0.722120295
POSTN	Diagnoses - secondary ICD10: Z87.4 Personal history of diseases of the genito-urinary system	Z87.4	1.000 (0.998, 1.001)	0.716293218
POSTN	Diagnoses - secondary ICD10: Z92.2 Personal history of long-term (current) use of other medicaments	Z92.2	1.000 (0.997, 1.002)	0.679848801
POSTN	Diagnoses - main ICD10: K80.2 Calculus of gallbladder without cholecystitis	K80.2	0.999 (0.997, 1.001)	0.274110511
POSTN	Diagnoses - secondary ICD10: M13.9 Arthritis, unspecified	M13.9	1.000 (0.999, 1.001)	0.566750668
POSTN	Diagnoses - main ICD10: R79.8 Other specified abnormal findings of blood chemistry	R79.8	1.001 (1.000, 1.002)	0.161071489
POSTN	Diagnoses - secondary ICD10: Z95.1 Presence of aortocoronary bypass graft	Z95.1	1.001 (0.999, 1.002)	0.326315898
POSTN	Diagnoses - secondary ICD10: W01.0 Home	W01.0	1.000 (0.999, 1.001)	0.693663267
POSTN	Diagnoses - secondary ICD10: Z85.8 Personal history of malignant neoplasms of other organs and systen	Z85.8	1.000 (0.998, 1.001)	0.93581138
POSTN	Diagnoses - main ICD10: I84.9 Unspecified haemorrhoids without complication	I84.9	0.998 (0.996, 1.000)	0.099313162

POSTN	Diagnoses - main ICD10: K60.2 Anal fissure, unspecified	K60.2	1.000 (0.999, 1.001)	0.713440425
POSTN	Diagnoses - main ICD10: N81.1 Cystocele	N81.1	1.000 (0.999, 1.002)	0.494015987
POSTN	Diagnoses - secondary ICD10: F41.9 Anxiety disorder, unspecified	F41.9	1.000 (0.999, 1.001)	0.955003831
POSTN	Diagnoses - main ICD10: K40.2 Bilateral inguinal hernia, without obstruction or gangrene	K40.2	1.000 (0.999, 1.001)	0.428971152
POSTN	Diagnoses - main ICD10: L90.5 Scar conditions and fibrosis of skin	L90.5	1.000 (0.999, 1.001)	0.736923667
POSTN	Diagnoses - main ICD10: R04.0 Epistaxis	R04.0	1.000 (0.999, 1.001)	0.741928311
POSTN	Diagnoses - secondary ICD10: Z51.1 Chemotherapy session for neoplasm	Z51.1	1.000 (0.998, 1.002)	0.846280573
POSTN	Diagnoses - main ICD10: R32 Unspecified urinary incontinence	R32	1.000 (0.999, 1.001)	0.5378368
POSTN	Diagnoses - main ICD10: N92.0 Excessive and frequent menstruation with regular cycle	N92.0	1.000 (0.997, 1.002)	0.686841366
POSTN	Diagnoses - main ICD10: K62.5 Haemorrhage of anus and rectum	K62.5	0.999 (0.997, 1.002)	0.503824133
POSTN	Diagnoses - secondary ICD10: Z96.1 Presence of intraocular lens	Z96.1	1.000 (0.999, 1.001)	0.931131015
POSTN	Diagnoses - secondary ICD10: R00.1 Bradycardia, unspecified	R00.1	1.000 (0.999, 1.001)	0.744881968
POSTN	Diagnoses - secondary ICD10: K66.0 Peritoneal adhesions	K66.0	0.999 (0.998, 1.000)	0.097372469
POSTN	Diagnoses - main ICD10: T84.8 Other complications of internal orthopaedic prosthetic devices, implants	T84.8	1.001 (1.000, 1.001)	0.215212392
POSTN	Diagnoses - secondary ICD10: Z85.4 Personal history of malignant neoplasm of genital organs	Z85.4	1.001 (1.000, 1.003)	0.087704906
POSTN	Diagnoses - main ICD10: I47.1 Supraventricular tachycardia	I47.1	1.000 (0.999, 1.001)	0.675214862
POSTN	Diagnoses - secondary ICD10: F10.0 Acute intoxication	F10.0	0.999 (0.999, 1.000)	0.214621573
POSTN	Diagnoses - main ICD10: K08.3 Retained dental root	K08.3	1.000 (0.999, 1.001)	0.637470038
POSTN	Diagnoses - secondary ICD10: M06.99 Rheumatoid arthritis, unspecified (Site unspecified)	M06.99	0.999 (0.998, 1.000)	0.057877656
POSTN	Diagnoses - main ICD10: L03.1 Cellulitis of other parts of limb	L03.1	1.000 (0.999, 1.002)	0.834624131
POSTN	Diagnoses - secondary ICD10: R53 Malaise and fatigue	R53	1.000 (1.000, 1.001)	0.359685196
POSTN	Diagnoses - secondary ICD10: W01.9 Unspecified place	W01.9	1.000 (0.999, 1.001)	0.569748668
POSTN	Diagnoses - main ICD10: C50.4 Upper-outer quadrant of breast	C50.4	0.999 (0.998, 1.000)	0.099624002
POSTN	Diagnoses - main ICD10: T39.1 4-Aminophenol derivatives	T39.1	1.000 (0.999, 1.000)	0.357421144
POSTN	Diagnoses - secondary ICD10: K29.5 Chronic gastritis, unspecified	K29.5	1.000 (0.999, 1.001)	0.48233388
POSTN	Diagnoses - secondary ICD10: G55.1 Nerve root and plexus compressions in intervertebral disk disorders	G55.1	1.000 (0.999, 1.001)	0.675033297
POSTN	Diagnoses - secondary ICD10: K25.9 Unspecified as acute or chronic, without haemorrhage or perforatio	K25.9	0.999 (0.999, 1.000)	0.28931194
POSTN	Diagnoses - main ICD10: I80.2 Phlebitis and thrombophlebitis of other deep vessels of lower extremities	I80.2	1.000 (0.998, 1.001)	0.382626782
POSTN	Diagnoses - secondary ICD10: I84.6 Residual haemorrhoidal skin tags	I84.6	0.998 (0.997, 0.999)	6.50586E-05
POSTN	Diagnoses - main ICD10: N32.0 Bladder-neck obstruction	N32.0	1.000 (0.999, 1.001)	0.458622518
POSTN	Diagnoses - main ICD10: K29.8 Duodenitis	K29.8	1.000 (0.999, 1.001)	0.893072587
POSTN	Diagnoses - main ICD10: M67.4 Ganglion	M67.4	1.000 (0.999, 1.002)	0.568140283
POSTN	Diagnoses - main ICD10: H26.9 Cataract, unspecified	H26.9	1.001 (0.998, 1.003)	0.590686355
POSTN	Type of cancer: ICD10: C20 Malignant neoplasm of rectum	C20	1.000 (0.999, 1.001)	0.521499698
POSTN	Diagnoses - secondary ICD10: K30 Dyspepsia	K30	1.000 (0.999, 1.001)	0.987226268
POSTN	Diagnoses - secondary ICD10: I84.2 Internal haemorrhoids without complication	I84.2	0.999 (0.998, 1.000)	0.186223293
POSTN	Diagnoses - secondary ICD10: N94.6 Dysmenorrhoea, unspecified	N94.6	1.000 (0.999, 1.001)	0.547839943

POSTN	Diagnoses - main ICD10: Z08.0 Follow-up examination after surgery for malignant neoplasm	Z08.0	1.001 (0.999, 1.002)	0.464059932
POSTN	Diagnoses - main ICD10: M79.86 Other specified soft tissue disorders (Lower leg)	M79.86	0.999 (0.998, 1.000)	0.252862779
POSTN	Diagnoses - secondary ICD10: E78.0 Pure hypercholesterolaemia	E78.0	1.001 (0.997, 1.005)	0.632297029
POSTN	Diagnoses - secondary ICD10: M10.99 Gout, unspecified (Site unspecified)	M10.99	1.000 (1.000, 1.001)	0.349054541
POSTN	Diagnoses - main ICD10: M51.2 Other specified intervertebral disk displacement	M51.2	0.999 (0.998, 1.000)	0.221582963
POSTN	Diagnoses - main ICD10: N84.0 Polyp of corpus uteri	N84.0	1.000 (0.998, 1.002)	0.823170165
POSTN	Diagnoses - secondary ICD10: M17.1 Other primary gonarthrosis	M17.1	0.999 (0.998, 1.000)	0.138189054
POSTN	Diagnoses - main ICD10: M25.5 Pain in joint	M25.5	1.000 (0.999, 1.001)	0.694118758
POSTN	Diagnoses - main ICD10: K29.6 Other gastritis	K29.6	1.000 (0.999, 1.001)	0.92243447
POSTN	Type of cancer: ICD10: C64 Malignant neoplasm of kidney, except renal pelvis	C64	1.001 (1.000, 1.001)	0.228552061
POSTN	Diagnoses - main ICD10: G43.9 Migraine, unspecified	G43.9	1.000 (1.000, 1.001)	0.30450271
POSTN	Diagnoses - secondary ICD10: Z80.0 Family history of malignant neoplasm of digestive organs	Z80.0	0.999 (0.997, 1.001)	0.233764329
POSTN	Diagnoses - main ICD10: G45.9 Transient cerebral ischaemic attack, unspecified	G45.9	0.999 (0.998, 1.000)	0.224756375
POSTN	Diagnoses - main ICD10: O02.1 Missed abortion	O02.1	1.000 (0.999, 1.001)	0.800606169
POSTN	Diagnoses - main ICD10: K62.8 Other specified diseases of anus and rectum	K62.8	1.000 (0.999, 1.001)	0.534431816
POSTN	Diagnoses - main ICD10: M72.0 Palmar fascial fibromatosis [Dupuytren]	M72.0	1.000 (0.999, 1.001)	0.585603224
POSTN	Diagnoses - secondary ICD10: I34.0 Mitral (valve) insufficiency	I34.0	1.000 (0.999, 1.000)	0.267815014
POSTN	Diagnoses - main ICD10: O04.9 Complete or unspecified, without complication	O04.9	1.000 (0.999, 1.000)	0.33814691
POSTN	Diagnoses - main ICD10: K22.1 Ulcer of oesophagus	K22.1	0.999 (0.998, 1.001)	0.374793224
POSTN	Diagnoses - secondary ICD10: Z86.7 Personal history of diseases of the circulatory system	Z86.7	0.999 (0.996, 1.001)	0.32547379
POSTN	Diagnoses - secondary ICD10: Y83.1 Surgical operation with implant of artificial internal device	Y83.1	1.001 (0.999, 1.002)	0.437642689
POSTN	Diagnoses - secondary ICD10: R63.4 Abnormal weight loss	R63.4	1.000 (0.999, 1.001)	0.833939077
POSTN	Diagnoses - main ICD10: K21.9 Gastro-oesophageal reflux disease without oesophagitis	K21.9	1.000 (0.998, 1.001)	0.669123151
POSTN	Diagnoses - main ICD10: N93.8 Other specified abnormal uterine and vaginal bleeding	N93.8	1.000 (0.999, 1.001)	0.571137207
POSTN	Diagnoses - main ICD10: M75.4 Impingement syndrome of shoulder	M75.4	1.001 (1.000, 1.003)	0.11079685
POSTN	Diagnoses - main ICD10: I20.9 Angina pectoris, unspecified	I20.9	0.999 (0.997, 1.001)	0.202124613
POSTN	Diagnoses - secondary ICD10: X59.9 Unspecified place	X59.9	0.999 (0.998, 1.001)	0.271442225
POSTN	Diagnoses - main ICD10: I84.1 Internal haemorrhoids with other complications	I84.1	1.000 (0.998, 1.001)	0.343442688
POSTN	Diagnoses - secondary ICD10: K57.9 Diverticular disease of intestine, part unspecified, without perforati	K57.9	1.000 (0.998, 1.001)	0.498449458
POSTN	Diagnoses - main ICD10: L72.9 Follicular cyst of skin and subcutaneous tissue, unspecified	L72.9	1.000 (0.999, 1.001)	0.944329976
POSTN	Diagnoses - secondary ICD10: Z92.3 Personal history of irradiation	Z92.3	1.000 (0.999, 1.001)	0.437303666
POSTN	Diagnoses - main ICD10: Z09.8 Follow-up examination after other treatment for other conditions	Z09.8	0.999 (0.998, 1.001)	0.298974868
POSTN	Diagnoses - main ICD10: K42.9 Umbilical hernia without obstruction or gangrene	K42.9	1.000 (0.999, 1.001)	0.899344722
POSTN	Diagnoses - main ICD10: R07.3 Other chest pain	R07.3	0.999 (0.997, 1.002)	0.596025888
POSTN	Diagnoses - main ICD10: N92.6 Irregular menstruation, unspecified	N92.6	1.000 (0.999, 1.001)	0.597218948
POSTN	Diagnoses - main ICD10: T84.0 Mechanical complication of internal joint prosthesis	T84.0	1.000 (0.999, 1.001)	0.68521881
POSTN	Diagnoses - main ICD10: D64.9 Anaemia, unspecified	D64.9	0.999 (0.998, 1.001)	0.264663377

POSTN	Diagnoses - secondary ICD10: R00.2 Palpitations	R00.2	1.000 (0.999, 1.001)	0.930809197
POSTN	Diagnoses - secondary ICD10: Z90.4 Acquired absence of other parts of digestive tract	Z90.4	1.000 (0.999, 1.002)	0.696538462
POSTN	Type of cancer: ICD10: C54.1 Endometrium	C54.1	1.001 (1.000, 1.002)	0.263285448
POSTN	Diagnoses - main ICD10: K92.2 Gastro-intestinal haemorrhage, unspecified	K92.2	1.000 (0.999, 1.001)	0.827747454
POSTN	Diagnoses - main ICD10: L72.0 Epidermal cyst	L72.0	0.999 (0.998, 1.000)	0.161765063
POSTN	Diagnoses - secondary ICD10: M13.99 Arthritis, unspecified (Site unspecified)	M13.99	1.000 (0.998, 1.001)	0.762344754
POSTN	Diagnoses - secondary ICD10: Z95.8 Presence of other cardiac and vascular implants and grafts	Z95.8	1.000 (0.999, 1.001)	0.985123432
POSTN	Diagnoses - secondary ICD10: K29.7 Gastritis, unspecified	K29.7	1.001 (0.999, 1.003)	0.29369073
POSTN	Diagnoses - secondary ICD10: E66.9 Obesity, unspecified	E66.9	1.000 (0.998, 1.002)	0.78323384
POSTN	Diagnoses - main ICD10: M54.5 Low back pain	M54.5	1.001 (1.000, 1.002)	0.073810338
POSTN	Diagnoses - main ICD10: J18.9 Pneumonia, unspecified	J18.9	1.000 (0.999, 1.001)	0.422608442
POSTN	Type of cancer: ICD10: C44.5 Skin of trunk	C44.5	1.000 (0.999, 1.002)	0.751991752
POSTN	Diagnoses - secondary ICD10: Z95.5 Presence of coronary angioplasty implant and graft	Z95.5	1.000 (0.998, 1.001)	0.924133003
POSTN	Type of cancer: ICD10: C18.7 Sigmoid colon	C18.7	1.000 (0.999, 1.001)	0.582933538
POSTN	Diagnoses - main ICD10: R87.6 Abnormal cytological findings	R87.6	1.000 (0.999, 1.000)	0.310346954
POSTN	Diagnoses - secondary ICD10: Z86.4 Personal history of psychoactive substance abuse	Z86.4	1.001 (0.998, 1.004)	0.39759522
POSTN	Diagnoses - secondary ICD10: Z51.5 Palliative care	Z51.5	1.000 (0.999, 1.001)	0.956813638
POSTN	Diagnoses - secondary ICD10: G40.9 Epilepsy, unspecified	G40.9	0.998 (0.997, 1.000)	0.016720767
POSTN	Diagnoses - secondary ICD10: Z51.0 Radiotherapy session	Z51.0	0.999 (0.998, 1.000)	0.22443053
POSTN	Diagnoses - secondary ICD10: F10.1 Harmful use	F10.1	1.000 (0.999, 1.001)	0.576532249
POSTN	Diagnoses - secondary ICD10: I51.7 Cardiomegaly	I51.7	1.000 (0.999, 1.001)	0.670001627
POSTN	Diagnoses - main ICD10: N23 Unspecified renal colic	N23	1.000 (0.999, 1.001)	0.502282263
POSTN	Diagnoses - secondary ICD10: E86 Volume depletion	E86	0.999 (0.998, 1.000)	0.050761728
POSTN	Diagnoses - secondary ICD10: Z87.1 Personal history of diseases of the digestive system	Z87.1	0.998 (0.996, 1.001)	0.258885923
POSTN	Diagnoses - secondary ICD10: Z85.0 Personal history of malignant neoplasm of digestive organs	Z85.0	1.001 (0.999, 1.002)	0.318766192
POSTN	Diagnoses - secondary ICD10: R03.0 Elevated blood-pressure reading, without diagnosis of hypertensior	R03.0	0.999 (0.999, 1.000)	0.243221332
POSTN	Diagnoses - secondary ICD10: I25.8 Other forms of chronic ischaemic heart disease	I25.8	1.000 (0.998, 1.002)	0.998093731
POSTN	Diagnoses - secondary ICD10: Z90.1 Acquired absence of breast(s)	Z90.1	1.001 (1.000, 1.002)	0.008328466
POSTN	Diagnoses - secondary ICD10: I25.2 Old myocardial infarction	I25.2	1.000 (0.999, 1.002)	0.770056795
POSTN	Diagnoses - main ICD10: I25.1 Atherosclerotic heart disease	I25.1	1.001 (0.998, 1.004)	0.517754655
POSTN	Diagnoses - secondary ICD10: Z91.0 Personal history of allergy, other than to drugs and biological subst	Z91.0	1.000 (0.999, 1.002)	0.398783914
POSTN	Diagnoses - secondary ICD10: C78.7 Secondary malignant neoplasm of liver	C78.7	1.000 (0.999, 1.001)	0.735537283
POSTN	Diagnoses - secondary ICD10: J44.9 Chronic obstructive pulmonary disease, unspecified	J44.9	1.000 (0.998, 1.001)	0.687850946
POSTN	Diagnoses - main ICD10: G47.3 Sleep apnoea	G47.3	1.000 (0.999, 1.001)	0.986832731
POSTN	Diagnoses - secondary ICD10: N95.0 Postmenopausal bleeding	N95.0	1.001 (0.999, 1.002)	0.393905921
POSTN	Diagnoses - main ICD10: S09.9 Unspecified injury of head	S09.9	1.000 (0.999, 1.001)	0.613170932
POSTN	Diagnoses - secondary ICD10: Z95.0 Presence of cardiac pacemaker	Z95.0	1.000 (0.999, 1.001)	0.983551421

POSTN	Diagnoses - secondary ICD10: C77.3 Axillary and upper limb lymph nodes	C77.3	1.000 (0.999, 1.001)	0.615058515
POSTN	Type of cancer: ICD10: C44.4 Skin of scalp and neck	C44.4	1.000 (0.999, 1.001)	0.433418773
POSTN	Diagnoses - secondary ICD10: J45.9 Asthma, unspecified	J45.9	0.996 (0.993, 1.000)	0.040306259
POSTN	Diagnoses - main ICD10: R39.8 Other and unspecified symptoms and signs involving the urinary system	R39.8	1.001 (1.000, 1.002)	0.224397462
POSTN	Diagnoses - secondary ICD10: R00.0 Tachycardia, unspecified	R00.0	0.999 (0.998, 1.000)	0.100789762
POSTN	Diagnoses - main ICD10: R10.3 Pain localised to other parts of lower abdomen	R10.3	0.998 (0.996, 1.000)	0.054657422
POSTN	Diagnoses - secondary ICD10: E78.5 Hyperlipidaemia, unspecified	E78.5	1.001 (1.000, 1.003)	0.065761643
POSTN	Diagnoses - secondary ICD10: E14.9 Without complications	E14.9	1.000 (0.999, 1.001)	0.73565118
POSTN	Diagnoses - main ICD10: T81.0 Haemorrhage and haematoma complicating a procedure, not elsewhere c	T81.0	1.000 (0.999, 1.001)	0.788672136
POSTN	Diagnoses - secondary ICD10: J18.1 Lobar pneumonia, unspecified	J18.1	1.000 (0.999, 1.001)	0.709073978
POSTN	Diagnoses - secondary ICD10: M81.99 Osteoporosis, unspecified (Site unspecified)	M81.99	1.000 (0.999, 1.001)	0.915299212
POSTN	Diagnoses - secondary ICD10: Y83.8 Other surgical procedures	Y83.8	1.001 (0.999, 1.002)	0.438491262
POSTN	Diagnoses - secondary ICD10: K63.5 Polyp of colon	K63.5	0.999 (0.998, 1.000)	0.078777008
POSTN	Diagnoses - main ICD10: K58.9 Irritable bowel syndrome without diarrhoea	K58.9	1.000 (0.999, 1.001)	0.674993639
POSTN	Diagnoses - secondary ICD10: K59.0 Constipation	K59.0	1.000 (0.998, 1.002)	0.94101786
POSTN	Diagnoses - secondary ICD10: Z87.3 Personal history of diseases of the musculoskeletal system and conn	Z87.3	1.001 (1.000, 1.002)	0.085253115
POSTN	Type of cancer: ICD10: C56 Malignant neoplasm of ovary	C56	1.000 (0.999, 1.001)	0.971092597
POSTN	Diagnoses - main ICD10: M51.1 Lumbar and other intervertebral disk disorders with radiculopathy	M51.1	1.000 (0.999, 1.001)	0.920094325
POSTN	Diagnoses - main ICD10: I26.9 Pulmonary embolism without mention of acute cor pulmonale	I26.9	1.000 (0.999, 1.001)	0.51770568
POSTN	Diagnoses - main ICD10: N20.0 Calculus of kidney	N20.0	1.000 (0.999, 1.001)	0.851102188
POSTN	Diagnoses - main ICD10: M17.9 Gonarthrosis, unspecified	M17.9	0.998 (0.996, 1.000)	0.098351559
POSTN	Diagnoses - main ICD10: K92.0 Haematemesis	K92.0	1.000 (0.999, 1.001)	0.712443446
POSTN	Diagnoses - main ICD10: N20.1 Calculus of ureter	N20.1	0.999 (0.998, 1.001)	0.357751927
POSTN	Diagnoses - main ICD10: K13.7 Other and unspecified lesions of oral mucosa	K13.7	1.000 (0.999, 1.001)	0.698138547
POSTN	Type of cancer: ICD10: C34.1 Upper lobe, bronchus or lung	C34.1	1.000 (0.999, 1.001)	0.961106849
POSTN	Diagnoses - main ICD10: Z42.1 Follow-up care involving plastic surgery of breast	Z42.1	1.000 (0.999, 1.002)	0.335015303
POSTN	Type of cancer: ICD10: C50.9 Breast, unspecified	C50.9	1.001 (0.999, 1.003)	0.499703268
POSTN	Diagnoses - main ICD10: J34.8 Other specified disorders of nose and nasal sinuses	J34.8	0.999 (0.998, 1.000)	0.193457208
POSTN	Type of cancer: ICD10: C44.6 Skin of upper limb, including shoulder	C44.6	1.000 (0.999, 1.001)	0.592571737
POSTN	Diagnoses - secondary ICD10: R50.9 Fever, unspecified	R50.9	1.001 (1.000, 1.002)	0.222586798
POSTN	Diagnoses - secondary ICD10: W10.0 Home	W10.0	1.001 (1.000, 1.002)	0.118058316
POSTN	Diagnoses - main ICD10: N84.1 Polyp of cervix uteri	N84.1	1.001 (1.000, 1.002)	0.098411096
POSTN	Diagnoses - secondary ICD10: R33 Retention of urine	R33	1.000 (0.998, 1.001)	0.656038707
POSTN	Diagnoses - main ICD10: D12.6 Colon, unspecified	D12.6	1.001 (0.999, 1.002)	0.368362484
POSTN	Diagnoses - main ICD10: I63.9 Cerebral infarction, unspecified	I63.9	1.000 (0.999, 1.001)	0.727992059
POSTN	Diagnoses - main ICD10: K51.9 Ulcerative colitis, unspecified	K51.9	0.999 (0.998, 1.000)	0.221475981
POSTN	Diagnoses - main ICD10: K85 Acute pancreatitis	K85	1.000 (0.999, 1.001)	0.997165811

POSTN	Diagnoses - secondary ICD10: R06.0 Dyspnoea	R06.0	0.998 (0.997, 1.000)	0.013334785
POSTN	Diagnoses - secondary ICD10: R10.4 Other and unspecified abdominal pain	R10.4	1.001 (0.999, 1.002)	0.498849348
POSTN	Diagnoses - main ICD10: Z47.0 Follow-up care involving removal of fracture plate and other internal fix	Z47.0	1.000 (0.998, 1.001)	0.5183188
POSTN	Diagnoses - secondary ICD10: F10.2 Dependence syndrome	F10.2	0.999 (0.998, 1.000)	0.057883242
POSTN	Diagnoses - main ICD10: R06.5 Mouth breathing	R06.5	1.001 (1.000, 1.002)	0.186903926
POSTN	Diagnoses - main ICD10: R51 Headache	R51	1.001 (0.999, 1.002)	0.48519908
POSTN	Diagnoses - main ICD10: K62.1 Rectal polyp	K62.1	1.000 (0.999, 1.002)	0.567433849
POSTN	Diagnoses - main ICD10: M51.3 Other specified intervertebral disk degeneration	M51.3	1.000 (1.000, 1.001)	0.367838197
POSTN	Diagnoses - main ICD10: M54.56 Low back pain (Lumbar region)	M54.56	1.000 (0.999, 1.001)	0.60650805
POSTN	Diagnoses - main ICD10: M16.9 Coxarthrosis, unspecified	M16.9	1.000 (0.999, 1.002)	0.700261926
POSTN	Diagnoses - secondary ICD10: Z86.1 Personal history of infectious and parasitic diseases	Z86.1	1.000 (0.999, 1.001)	0.691670428
POSTN	Diagnoses - secondary ICD10: I12.0 Hypertensive renal disease with renal failure	I12.0	1.000 (0.999, 1.001)	0.670380959
POSTN	Diagnoses - secondary ICD10: R05 Cough	R05	1.000 (1.000, 1.001)	0.273538004
POSTN	Diagnoses - main ICD10: Z13.8 Special screening examination for other specified diseases and disorders	Z13.8	1.000 (0.999, 1.001)	0.474662424
POSTN	Diagnoses - secondary ICD10: F32.9 Depressive episode, unspecified	F32.9	0.999 (0.997, 1.001)	0.194883484
POSTN	Diagnoses - secondary ICD10: Z85.3 Personal history of malignant neoplasm of breast	Z85.3	1.002 (1.000, 1.004)	0.034023138
POSTN	Diagnoses - main ICD10: K52.9 Non-infective gastro-enteritis and colitis, unspecified	K52.9	1.002 (0.999, 1.004)	0.122655546
POSTN	Diagnoses - main ICD10: L98.9 Disorder of skin and subcutaneous tissue, unspecified	L98.9	1.000 (0.999, 1.002)	0.932813029
POSTN	Diagnoses - secondary ICD10: M19.99 Arthrosis, unspecified (Site unspecified)	M19.99	1.000 (0.999, 1.001)	0.912211634
POSTN	Diagnoses - main ICD10: Z12.1 Special screening examination for neoplasm of intestinal tract	Z12.1	0.999 (0.998, 1.001)	0.322053538
POSTN	Diagnoses - main ICD10: R07.2 Precordial pain	R07.2	1.000 (0.999, 1.001)	0.917959209
POSTN	Diagnoses - secondary ICD10: Z90.7 Acquired absence of genital organ(s)	Z90.7	1.000 (0.998, 1.001)	0.890022916
POSTN	Diagnoses - secondary ICD10: Z96.6 Presence of orthopaedic joint implants	Z96.6	1.002 (1.000, 1.004)	0.08701892
POSTN	Diagnoses - secondary ICD10: B96.8 Other specified bacterial agents as the cause of diseases classified to	B96.8	1.000 (0.998, 1.001)	0.731947751
POSTN	Diagnoses - secondary ICD10: R42 Dizziness and giddiness	R42	1.000 (0.999, 1.001)	0.521705851
POSTN	Diagnoses - secondary ICD10: N81.2 Incomplete uterovaginal prolapse	N81.2	0.999 (0.999, 1.000)	0.170356671
POSTN	Diagnoses - main ICD10: M25.56 Pain in joint (Lower leg)	M25.56	1.001 (1.000, 1.002)	0.088566077
POSTN	Diagnoses - main ICD10: D17.2 Benign lipomatous neoplasm of skin and subcutaneous tissue of limbs	D17.2	0.999 (0.998, 1.000)	0.312209005
POSTN	Diagnoses - main ICD10: O63.1 Prolonged second stage (of labour)	O63.1	1.000 (0.999, 1.001)	0.976297861
POSTN	Diagnoses - secondary ICD10: Z82.4 Family history of ischaemic heart disease and other diseases of the c	Z82.4	1.000 (0.998, 1.003)	0.963736421
POSTN	Diagnoses - main ICD10: H25.0 Senile incipient cataract	H25.0	1.000 (0.999, 1.001)	0.962607177
POSTN	Diagnoses - main ICD10: D17.1 Benign lipomatous neoplasm of skin and subcutaneous tissue of trunk	D17.1	1.000 (0.999, 1.001)	0.932075771
POSTN	Diagnoses - secondary ICD10: M47.82 Other spondylosis (Cervical region)	M47.82	1.000 (0.999, 1.000)	0.291370776
POSTN	Diagnoses - secondary ICD10: Z98.0 Intestinal bypass and anastomosis status	Z98.0	1.000 (0.999, 1.001)	0.829090839
POSTN	Type of cancer: ICD10: C43.7 Malignant melanoma of lower limb, including hip	C43.7	1.000 (1.000, 1.001)	0.311229307
POSTN	Diagnoses - main ICD10: S52.50 Fracture of lower end of radius (closed)	S52.50	1.000 (0.999, 1.001)	0.956186472
POSTN	Diagnoses - secondary ICD10: W19.9 Unspecified place	W19.9	0.999 (0.998, 1.001)	0.369126113

POSTN	Diagnoses - main ICD10: M16.1 Other primary coxarthrosis	M16.1	1.000 (0.998, 1.001)	0.781760326
POSTN	Diagnoses - secondary ICD10: Z60.2 Living alone	Z60.2	1.001 (1.000, 1.003)	0.06870445
POSTN	Diagnoses - secondary ICD10: F17.1 Harmful use	F17.1	0.999 (0.998, 1.000)	0.202642505
POSTN	Diagnoses - secondary ICD10: K31.8 Other specified diseases of stomach and duodenum	K31.8	1.000 (0.999, 1.001)	0.801096535
POSTN	Diagnoses - secondary ICD10: K31.7 Polyp of stomach and duodenum	K31.7	1.000 (0.999, 1.001)	0.89510703
POSTN	Diagnoses - main ICD10: H25.1 Senile nuclear cataract	H25.1	1.000 (0.998, 1.001)	0.519892245
POSTN	Diagnoses - secondary ICD10: Y83.2 Surgical operation with anastomosis, bypass or graft	Y83.2	1.001 (1.000, 1.002)	0.055643335
POSTN	Diagnoses - secondary ICD10: D70 Agranulocytosis	D70	1.001 (1.000, 1.001)	0.167598965
POSTN	Diagnoses - main ICD10: D50.9 Iron deficiency anaemia, unspecified	D50.9	0.999 (0.998, 1.001)	0.358043049
POSTN	Diagnoses - secondary ICD10: N81.6 Rectocele	N81.6	1.000 (0.999, 1.001)	0.826718525
POSTN	Diagnoses - main ICD10: I21.9 Acute myocardial infarction, unspecified	I21.9	1.000 (0.999, 1.001)	0.741872442
POSTN	Diagnoses - main ICD10: D22.3 Melanocytic naevi of other and unspecified parts of face	D22.3	1.000 (0.999, 1.001)	0.653324568
POSTN	Diagnoses - secondary ICD10: K44.9 Diaphragmatic hernia without obstruction or gangrene	K44.9	1.000 (0.997, 1.003)	0.942053071
POSTN	Diagnoses - main ICD10: B34.9 Viral infection, unspecified	B34.9	1.000 (0.999, 1.001)	0.924740921
POSTN	Diagnoses - main ICD10: N32.8 Other specified disorders of bladder	N32.8	1.001 (0.999, 1.002)	0.302368242
POSTN	Diagnoses - main ICD10: J34.2 Deviated nasal septum	J34.2	1.000 (0.998, 1.001)	0.787948431
POSTN	Diagnoses - main ICD10: N50.8 Other specified disorders of male genital organs	N50.8	1.000 (0.999, 1.001)	0.69430507
POSTN	Diagnoses - secondary ICD10: Z86.0 Personal history of other neoplasms	Z86.0	1.000 (0.998, 1.001)	0.782601647
POSTN	Diagnoses - secondary ICD10: Z72.1 Alcohol use	Z72.1	1.000 (0.999, 1.002)	0.542188263
POSTN	Diagnoses - main ICD10: G56.0 Carpal tunnel syndrome	G56.0	1.000 (0.998, 1.002)	0.944914736
POSTN	Diagnoses - main ICD10: I25.9 Chronic ischaemic heart disease, unspecified	I25.9	1.000 (0.999, 1.001)	0.598816618
POSTN	Diagnoses - secondary ICD10: Z37.0 Single live birth	Z37.0	1.001 (0.998, 1.003)	0.688842433
POSTN	Diagnoses - secondary ICD10: E03.9 Hypothyroidism, unspecified	E03.9	1.002 (0.999, 1.004)	0.179941522
POSTN	Diagnoses - secondary ICD10: Z88.6 Personal history of allergy to analgesic agent	Z88.6	1.001 (1.000, 1.002)	0.119754118
POSTN	Diagnoses - main ICD10: M20.2 Hallux rigidus	M20.2	1.000 (0.999, 1.001)	0.522802424
POSTN	Diagnoses - secondary ICD10: B95.6 Staphylococcus aureus as the cause of diseases classified to other ch	B95.6	1.000 (0.999, 1.002)	0.408645144
POSTN	Diagnoses - main ICD10: R04.2 Haemoptysis	R04.2	1.000 (0.999, 1.001)	0.738846369
POSTN	Diagnoses - main ICD10: I21.0 Acute transmural myocardial infarction of anterior wall	I21.0	1.000 (0.999, 1.001)	0.375874297
POSTN	Diagnoses - secondary ICD10: R94.5 Abnormal results of liver function studies	R94.5	1.000 (0.999, 1.001)	0.708264431
POSTN	Diagnoses - secondary ICD10: T81.4 Infection following a procedure, not elsewhere classified	T81.4	1.001 (1.000, 1.002)	0.144433066
POSTN	Diagnoses - secondary ICD10: Z88.8 Personal history of allergy to other drugs, medicaments and biologi	Z88.8	1.001 (0.999, 1.002)	0.419967769
POSTN	Diagnoses - main ICD10: H00.1 Chalazion	H00.1	1.000 (0.999, 1.001)	0.356043618
POSTN	Type of cancer: ICD10: D05.1 Intraductal carcinoma in situ	D05.1	1.000 (0.999, 1.001)	0.896426441
POSTN	Diagnoses - secondary ICD10: Y43.3 Other antineoplastic drugs	Y43.3	1.000 (0.999, 1.001)	0.829062049
POSTN	Diagnoses - main ICD10: K02.9 Dental caries, unspecified	K02.9	1.000 (0.999, 1.001)	0.672044289
POSTN	Diagnoses - main ICD10: M72.04 Palmar fascial fibromatosis [Dupuytren]-Hand	M72.04	1.000 (0.999, 1.001)	0.595456672
POSTN	Diagnoses - secondary ICD10: N83.2 Other and unspecified ovarian cysts	N83.2	1.000 (0.999, 1.001)	0.624958581

POSTN	Diagnoses - main ICD10: M75.0 Adhesive capsulitis of shoulder	M75.0	1.000 (0.999, 1.001)	0.713781358
POSTN	Diagnoses - secondary ICD10: I73.9 Peripheral vascular disease, unspecified	I73.9	1.000 (0.999, 1.001)	0.896807951
POSTN	Diagnoses - secondary ICD10: N17.9 Acute renal failure, unspecified	N17.9	1.000 (0.999, 1.001)	0.471098359
POSTN	Diagnoses - main ICD10: M75.1 Rotator cuff syndrome	M75.1	1.000 (0.999, 1.001)	0.702618982
POSTN	Diagnoses - main ICD10: L82 Seborrhoeic keratosis	L82	1.000 (0.999, 1.001)	0.645007521
POSTN	Diagnoses - main ICD10: K61.0 Anal abscess	K61.0	1.001 (1.000, 1.001)	0.185375714
POSTN	Diagnoses - secondary ICD10: Z53.2 Procedure not carried out because of patient's decision for other an	Z53.2	0.999 (0.998, 1.000)	0.178936167
POSTN	Diagnoses - secondary ICD10: Z72.0 Tobacco use	Z72.0	1.002 (0.999, 1.005)	0.233889854
POSTN	Diagnoses - main ICD10: I21.1 Acute transmural myocardial infarction of inferior wall	I21.1	1.000 (0.999, 1.001)	0.873447929
POSTN	Diagnoses - secondary ICD10: N39.3 Stress incontinence	N39.3	1.000 (0.999, 1.001)	0.519989044
POSTN	Diagnoses - main ICD10: K40.9 Unilateral or unspecified inguinal hernia, without obstruction or gangre	K40.9	1.001 (0.998, 1.004)	0.474313987
POSTN	Diagnoses - main ICD10: C67.9 Bladder, unspecified	C67.9	0.999 (0.998, 1.000)	0.142349994
POSTN	Diagnoses - secondary ICD10: Z13.0 Special screening examination for diseases of the blood and blood-	Z13.0	1.000 (0.999, 1.000)	0.397970522
POSTN	Diagnoses - secondary ICD10: Z86.6 Personal history of diseases of the nervous system and sense organs	Z86.6	1.000 (0.998, 1.001)	0.628090861
POSTN	Diagnoses - main ICD10: K92.1 Melaena	K92.1	1.000 (0.999, 1.001)	0.539607329
POSTN	Diagnoses - secondary ICD10: J34.3 Hypertrophy of nasal turbinates	J34.3	1.000 (0.999, 1.001)	0.599463386
POSTN	Diagnoses - secondary ICD10: Z88.0 Personal history of allergy to penicillin	Z88.0	1.000 (0.997, 1.002)	0.800731667
POSTN	Diagnoses - main ICD10: S82.80 Fractures of other parts of lower leg (closed)	S82.80	1.000 (0.999, 1.001)	0.804081524
POSTN	Diagnoses - main ICD10: M20.1 Hallux valgus (acquired)	M20.1	1.001 (0.999, 1.003)	0.469989172
POSTN	Diagnoses - secondary ICD10: N39.0 Urinary tract infection, site not specified	N39.0	0.998 (0.997, 1.000)	0.039727292
POSTN	Diagnoses - secondary ICD10: Z03.4 Observation for suspected myocardial infarction	Z03.4	1.000 (0.999, 1.001)	0.840475838
POSTN	Diagnoses - main ICD10: O80.0 Spontaneous vertex delivery	O80.0	1.000 (0.999, 1.001)	0.879453763
POSTN	Diagnoses - secondary ICD10: N73.6 Female pelvic peritoneal adhesions	N73.6	1.001 (1.000, 1.002)	0.143842328
POSTN	Diagnoses - secondary ICD10: D25.9 Leiomyoma of uterus, unspecified	D25.9	1.001 (0.999, 1.002)	0.447135454
POSTN	Diagnoses - secondary ICD10: K26.9 Unspecified as acute or chronic, without haemorrhage or perforatio	K26.9	1.000 (0.999, 1.001)	0.914995253
POSTN	Diagnoses - main ICD10: K60.3 Anal fistula	K60.3	1.000 (1.000, 1.001)	0.266806588
POSTN	Diagnoses - main ICD10: K43.9 Ventral hernia without obstruction or gangrene	K43.9	1.000 (0.999, 1.001)	0.678549767
POSTN	Diagnoses - main ICD10: K35.9 Acute appendicitis, unspecified	K35.9	1.000 (0.999, 1.001)	0.957897224
POSTN	Type of cancer: ICD10: C44.7 Skin of lower limb, including hip	C44.7	1.000 (1.000, 1.001)	0.312453911
POSTN	Diagnoses - secondary ICD10: M15.9 Polyarthrosis, unspecified	M15.9	1.000 (0.999, 1.001)	0.823319224
POSTN	Diagnoses - main ICD10: R13 Dysphagia	R13	1.001 (0.999, 1.002)	0.275718627
POSTN	Diagnoses - main ICD10: M23.23 Derangement of meniscus due to old tear or injury (Medial collateral l	M23.23	1.000 (0.999, 1.002)	0.586895647
POSTN	Diagnoses - secondary ICD10: Z51.2 Other chemotherapy	Z51.2	1.001 (1.000, 1.002)	0.18085805
POSTN	Diagnoses - main ICD10: J33.9 Nasal polyp, unspecified	J33.9	1.000 (0.999, 1.001)	0.583560102
POSTN	Diagnoses - secondary ICD10: Z85.5 Personal history of malignant neoplasm of urinary tract	Z85.5	1.000 (0.999, 1.001)	0.611354645
POSTN	Diagnoses - secondary ICD10: Z92.1 Personal history of long-term (current) use of anticoagulants	Z92.1	1.000 (0.999, 1.002)	0.619279605
POSTN	Diagnoses - main ICD10: Z30.2 Sterilisation	Z30.2	1.001 (0.999, 1.003)	0.455785475

POSTN	Diagnoses - main ICD10: N63 Unspecified lump in breast	N63	1.000 (0.999, 1.001)	0.643260181
POSTN	Diagnoses - main ICD10: I84.8 Unspecified haemorrhoids with other complications	I84.8	1.000 (0.999, 1.001)	0.930114608
POSTN	Diagnoses - main ICD10: Z46.6 Fitting and adjustment of urinary device	Z46.6	0.999 (0.998, 1.001)	0.434766491
POSTN	Diagnoses - main ICD10: K01.1 Impacted teeth	K01.1	1.000 (0.999, 1.001)	0.829771602
POSTN	Diagnoses - secondary ICD10: Z80.3 Family history of malignant neoplasm of breast	Z80.3	1.000 (0.999, 1.001)	0.635665737
POSTN	Diagnoses - secondary ICD10: Y83.6 Removal of other organ (partial) (total)	Y83.6	1.000 (0.998, 1.001)	0.780474494
POSTN	Diagnoses - main ICD10: M65.3 Trigger finger	M65.3	0.999 (0.999, 1.000)	0.19928001
POSTN	Diagnoses - secondary ICD10: Z92.4 Personal history of major surgery, not elsewhere classified	Z92.4	1.000 (0.999, 1.000)	0.278536705
POSTN	Diagnoses - main ICD10: M23.2 Derangement of meniscus due to old tear or injury	M23.2	0.999 (0.998, 1.000)	0.13150765
POSTN	Diagnoses - main ICD10: K80.5 Calculus of bile duct without cholangitis or cholecystitis	K80.5	1.000 (0.999, 1.001)	0.738711651
POSTN	Type of cancer: ICD10: C44.9 Malignant neoplasm of skin, unspecified	C44.9	1.001 (0.999, 1.002)	0.29856403
POSTN	Diagnoses - main ICD10: D24 Benign neoplasm of breast	D24	1.000 (0.999, 1.001)	0.39197736
POSTN	Diagnoses - secondary ICD10: N85.8 Other specified noninflammatory disorders of uterus	N85.8	1.000 (0.999, 1.001)	0.697148699
POSTN	Diagnoses - secondary ICD10: I95.9 Hypotension, unspecified	I95.9	1.000 (0.999, 1.001)	0.810324858
POSTN	Diagnoses - secondary ICD10: B96.2 Escherichia coli [E. coli] as the cause of diseases classified to other	B96.2	1.000 (0.999, 1.001)	0.352054585
POSTN	Diagnoses - main ICD10: K80.1 Calculus of gallbladder with other cholecystitis	K80.1	1.001 (0.999, 1.003)	0.253237903
POSTN	Diagnoses - secondary ICD10: Z53.0 Procedure not carried out because of contraindication	Z53.0	0.999 (0.997, 1.000)	0.128954528
POSTN	Diagnoses - secondary ICD10: Z53.8 Procedure not carried out for other reasons	Z53.8	1.000 (0.997, 1.002)	0.714216054
POSTN	Diagnoses - main ICD10: N35.9 Urethral stricture, unspecified	N35.9	1.000 (0.999, 1.001)	0.871132759
POSTN	Type of cancer: ICD10: D06.9 Cervix, unspecified	D06.9	1.000 (0.998, 1.001)	0.558995627
POSTN	Diagnoses - secondary ICD10: Z53.9 Procedure not carried out, unspecified reason	Z53.9	1.000 (0.999, 1.001)	0.715819591
POSTN	Diagnoses - main ICD10: O68.0 Labour and delivery complicated by foetal heart rate anomaly	O68.0	1.000 (0.999, 1.001)	0.998216288
POSTN	Diagnoses - main ICD10: D12.8 Rectum	D12.8	1.001 (1.000, 1.002)	0.234680161
POSTN	Diagnoses - secondary ICD10: M19.9 Arthrosis, unspecified	M19.9	1.000 (0.999, 1.001)	0.478677103
POSTN	Diagnoses - main ICD10: H02.8 Other specified disorders of eyelid	H02.8	1.000 (0.999, 1.001)	0.589058837
POSTN	Diagnoses - main ICD10: N92.1 Excessive and frequent menstruation with irregular cycle	N92.1	1.000 (0.998, 1.001)	0.423120988
POSTN	Diagnoses - main ICD10: O70.0 First degree perineal laceration during delivery	O70.0	1.000 (0.999, 1.001)	0.986810477
POSTN	Diagnoses - secondary ICD10: N80.0 Endometriosis of uterus	N80.0	1.000 (0.999, 1.001)	0.458018566
POSTN	Diagnoses - main ICD10: M23.22 Derangement of meniscus due to old tear or injury (Posterior cruciate	M23.22	1.000 (0.999, 1.001)	0.582260114
POSTN	Diagnoses - secondary ICD10: E11.9 Without complications	E11.9	0.999 (0.996, 1.002)	0.540325296
POSTN	Diagnoses - main ICD10: M79.66 Pain in limb (Lower leg)	M79.66	1.000 (0.999, 1.001)	0.600641582
POSTN	Diagnoses - main ICD10: O26.8 Other specified pregnancy-related conditions	O26.8	1.000 (0.999, 1.001)	0.990940237
POSTN	Diagnoses - main ICD10: K56.6 Other and unspecified intestinal obstruction	K56.6	1.000 (0.999, 1.001)	0.506743401
POSTN	Diagnoses - secondary ICD10: Z88.1 Personal history of allergy to other antibiotic agents	Z88.1	1.001 (0.999, 1.002)	0.282309859
POSTN	Diagnoses - secondary ICD10: J90 Pleural effusion, not elsewhere classified	J90	1.001 (0.999, 1.002)	0.373997068
POSTN	Underlying (primary) cause of death: ICD10: C34.9 Bronchus or lung, unspecified	C34.9	0.995 (0.955, 1.036)	0.792355805
POSTN	Diagnoses - main ICD10: A41 Other septicaemia	A41	1.001 (1.000, 1.002)	0.160062473

POSTN	Diagnoses - main ICD10: C18 Malignant neoplasm of colon	C18	1.000 (0.999, 1.002)	0.771157949
POSTN	Diagnoses - main ICD10: C34 Malignant neoplasm of bronchus and lung	C34	1.000 (0.999, 1.001)	0.860453651
POSTN	Diagnoses - main ICD10: C43 Malignant melanoma of skin	C43	1.001 (0.999, 1.002)	0.445595511
POSTN	Diagnoses - main ICD10: C67 Malignant neoplasm of bladder	C67	1.000 (0.998, 1.001)	0.670139225
POSTN	Diagnoses - main ICD10: C78 Secondary malignant neoplasm of respiratory and digestive organs	C78	1.001 (0.999, 1.002)	0.412142477
POSTN	Diagnoses - main ICD10: C79 Secondary malignant neoplasm of other sites	C79	1.000 (0.999, 1.001)	0.776775976
POSTN	Diagnoses - main ICD10: D05 Carcinoma in situ of breast	D05	1.000 (0.998, 1.001)	0.527413549
POSTN	Diagnoses - main ICD10: D17 Benign lipomatous neoplasm	D17	1.000 (0.998, 1.002)	0.891466073
POSTN	Diagnoses - main ICD10: D22 Melanocytic naevi	D22	1.001 (0.999, 1.003)	0.444683488
POSTN	Diagnoses - main ICD10: D23 Other benign neoplasms of skin	D23	1.001 (0.999, 1.003)	0.406795687
POSTN	Diagnoses - main ICD10: D50 Iron deficiency anaemia	D50	1.001 (0.999, 1.003)	0.505547699
POSTN	Diagnoses - main ICD10: D64 Other anaemias	D64	0.998 (0.996, 1.000)	0.129778523
POSTN	Diagnoses - main ICD10: G43 Migraine	G43	1.000 (0.999, 1.001)	0.602392917
POSTN	Diagnoses - main ICD10: G45 Transient cerebral ischaemic attacks and related syndromes	G45	0.999 (0.998, 1.001)	0.408502287
POSTN	Diagnoses - main ICD10: G57 Mononeuropathies of lower limb	G57	1.001 (1.000, 1.002)	0.174266909
POSTN	Diagnoses - main ICD10: H00 Hordeolum and chalazion	H00	0.999 (0.998, 1.000)	0.075124633
POSTN	Diagnoses - main ICD10: H02 Other disorders of eyelid	H02	1.002 (1.000, 1.004)	0.098851388
POSTN	Diagnoses - main ICD10: H04 Disorders of lachrymal system	H04	1.001 (1.000, 1.002)	0.10906267
POSTN	Diagnoses - main ICD10: H33 Retinal detachments and breaks	H33	1.001 (0.999, 1.003)	0.356043083
POSTN	Diagnoses - main ICD10: H35 Other retinal disorders	H35	1.002 (1.000, 1.003)	0.011894448
POSTN	Diagnoses - main ICD10: H40 Glaucoma	H40	1.000 (0.999, 1.001)	0.920122148
POSTN	Diagnoses - main ICD10: I26 Pulmonary embolism	I26	0.999 (0.997, 1.000)	0.085429026
POSTN	Diagnoses - main ICD10: I47 Paroxysmal tachycardia	I47	1.000 (0.999, 1.002)	0.717758102
POSTN	Diagnoses - main ICD10: I50 Heart failure	I50	1.000 (0.999, 1.001)	0.705871009
POSTN	Diagnoses - main ICD10: I63 Cerebral infarction	I63	1.000 (0.999, 1.002)	0.826211053
POSTN	Diagnoses - main ICD10: J18 Pneumonia, organism unspecified	J18	0.999 (0.996, 1.001)	0.288866817
POSTN	Diagnoses - main ICD10: J32 Chronic sinusitis	J32	0.999 (0.998, 1.000)	0.159395416
POSTN	Diagnoses - main ICD10: J38 Diseases of vocal cords and larynx, not elsewhere classified	J38	1.000 (0.999, 1.001)	0.845362865
POSTN	Diagnoses - main ICD10: J45 Asthma	J45	1.000 (0.999, 1.001)	0.977881811
POSTN	Diagnoses - main ICD10: K01 Embedded and impacted teeth	K01	1.000 (0.998, 1.001)	0.499937032
POSTN	Diagnoses - main ICD10: K02 Dental caries	K02	1.001 (0.999, 1.002)	0.298085545
POSTN	Diagnoses - main ICD10: K04 Diseases of pulp and periapical tissues	K04	1.000 (0.999, 1.001)	0.896152804
POSTN	Diagnoses - main ICD10: K08 Other disorders of teeth and supporting structures	K08	1.000 (0.998, 1.001)	0.719303647
POSTN	Diagnoses - main ICD10: K13 Other diseases of lip and oral mucosa	K13	0.999 (0.998, 1.001)	0.26033051
POSTN	Diagnoses - main ICD10: K25 Gastric ulcer	K25	0.999 (0.998, 1.001)	0.248959572
POSTN	Diagnoses - main ICD10: K26 Duodenal ulcer	K26	1.000 (0.999, 1.001)	0.662544853
POSTN	Diagnoses - main ICD10: K31 Other diseases of stomach and duodenum	K31	1.000 (0.998, 1.001)	0.73278113

POSTN	Diagnoses - main ICD10: K42 Umbilical hernia	K42	1.000 (0.998, 1.001)	0.735710599
POSTN	Diagnoses - main ICD10: K56 Paralytic ileus and intestinal obstruction without hernia	K56	1.000 (0.998, 1.001)	0.704959597
POSTN	Diagnoses - main ICD10: K58 Irritable bowel syndrome	K58	1.000 (0.999, 1.002)	0.408669934
POSTN	Diagnoses - main ICD10: K59 Other functional intestinal disorders	K59	1.000 (0.998, 1.002)	0.928117091
POSTN	Diagnoses - main ICD10: K61 Abscess of anal and rectal regions	K61	1.000 (0.999, 1.001)	0.713345236
POSTN	Diagnoses - main ICD10: K63 Other diseases of intestine	K63	0.999 (0.996, 1.002)	0.665348671
POSTN	Diagnoses - main ICD10: K81 Cholecystitis	K81	1.002 (1.000, 1.003)	0.024999592
POSTN	Diagnoses - main ICD10: K92 Other diseases of digestive system	K92	0.999 (0.997, 1.002)	0.565306883
POSTN	Diagnoses - main ICD10: L02 Cutaneous abscess, furuncle and carbuncle	L02	1.000 (0.999, 1.001)	0.90622097
POSTN	Diagnoses - main ICD10: L57 Skin changes due to chronic exposure to nonionising radiation	L57	1.000 (0.998, 1.001)	0.526304947
POSTN	Diagnoses - main ICD10: L72 Follicular cysts of skin and subcutaneous tissue	L72	0.999 (0.996, 1.001)	0.357608772
POSTN	Diagnoses - main ICD10: L90 Atrophic disorders of skin	L90	0.999 (0.998, 1.001)	0.326210174
POSTN	Diagnoses - main ICD10: L98 Other disorders of skin and subcutaneous tissue, not elsewhere classified	L98	1.000 (0.998, 1.002)	0.751753454
POSTN	Diagnoses - main ICD10: M06 Other rheumatoid arthritis	M06	0.999 (0.998, 1.001)	0.359335284
POSTN	Diagnoses - main ICD10: M13 Other arthritis	M13	1.001 (0.999, 1.002)	0.287745709
POSTN	Diagnoses - main ICD10: M15 Polyarthrosis	M15	0.999 (0.998, 1.001)	0.289382847
POSTN	Diagnoses - main ICD10: M19 Other arthrosis	M19	1.000 (0.998, 1.002)	0.877940049
POSTN	Diagnoses - main ICD10: M47 Spondylosis	M47	1.000 (0.998, 1.001)	0.930895062
POSTN	Diagnoses - main ICD10: M48 Other spondylopathies	M48	1.001 (1.000, 1.003)	0.119586327
POSTN	Diagnoses - main ICD10: M51 Other intervertebral disk disorders	M51	1.000 (0.997, 1.002)	0.739283668
POSTN	Diagnoses - main ICD10: M65 Synovitis and tenosynovitis	M65	1.000 (0.998, 1.001)	0.623905294
POSTN	Diagnoses - main ICD10: M75 Shoulder lesions	M75	1.000 (0.997, 1.003)	0.889350174
POSTN	Diagnoses - main ICD10: M79 Other soft tissue disorders, not elsewhere classified	M79	0.998 (0.996, 1.001)	0.283594958
POSTN	Diagnoses - main ICD10: M84 Disorders of continuity of bone	M84	1.001 (1.000, 1.002)	0.06646319
POSTN	Diagnoses - main ICD10: N13 Obstructive and reflux uropathy	N13	0.999 (0.998, 1.000)	0.149997155
POSTN	Diagnoses - main ICD10: N30 Cystitis	N30	1.000 (0.998, 1.001)	0.535636643
POSTN	Diagnoses - main ICD10: N31 Neuromuscular dysfunction of bladder, not elsewhere classified	N31	1.000 (0.999, 1.001)	0.697646975
POSTN	Diagnoses - main ICD10: N35 Urethral stricture	N35	1.000 (0.999, 1.002)	0.758691759
POSTN	Diagnoses - main ICD10: N39 Other disorders of urinary system	N39	0.997 (0.994, 1.001)	0.098778239
POSTN	Diagnoses - main ICD10: N48 Other disorders of penis	N48	1.000 (0.999, 1.001)	0.956939271
POSTN	Diagnoses - main ICD10: N50 Other disorders of male genital organs	N50	1.000 (0.999, 1.002)	0.538818925
POSTN	Diagnoses - main ICD10: N60 Benign mammary dysplasia	N60	1.000 (0.999, 1.002)	0.416454558
POSTN	Diagnoses - main ICD10: N80 Endometriosis	N80	0.999 (0.998, 1.000)	0.115928739
POSTN	Diagnoses - main ICD10: N83 Noninflammatory disorders of ovary, Fallopian tube and broad ligament	N83	0.999 (0.998, 1.001)	0.504358223
POSTN	Diagnoses - main ICD10: N84 Polyp of female genital tract	N84	0.999 (0.996, 1.002)	0.52987342
POSTN	Diagnoses - main ICD10: N85 Other noninflammatory disorders of uterus, except cervix	N85	0.999 (0.997, 1.000)	0.04679083
POSTN	Diagnoses - main ICD10: N87 Dysplasia of cervix uteri	N87	1.000 (0.999, 1.001)	0.56986639

POSTN	Diagnoses - main ICD10: N90 Other noninflammatory disorders of vulva and perineum	N90	1.000 (0.999, 1.001)	0.880591874
POSTN	Diagnoses - main ICD10: N93 Other abnormal uterine and vaginal bleeding	N93	1.000 (0.999, 1.002)	0.617409835
POSTN	Diagnoses - main ICD10: N94 Pain and other conditions associated with female genital organs and menst	N94	1.001 (1.000, 1.002)	0.133841083
POSTN	Diagnoses - main ICD10: N95 Menopausal and other perimenopausal disorders	N95	0.999 (0.997, 1.002)	0.68191659
POSTN	Diagnoses - main ICD10: O02 Other abnormal products of conception	O02	1.000 (0.999, 1.001)	0.883946202
POSTN	Diagnoses - main ICD10: O03 Spontaneous abortion	O03	1.000 (0.999, 1.001)	0.999339602
POSTN	Diagnoses - main ICD10: O26 Maternal care for other conditions predominantly related to pregnancy	O26	1.000 (0.999, 1.002)	0.512570852
POSTN	Diagnoses - main ICD10: O36 Maternal care for other known or suspected foetal problems	O36	1.000 (0.999, 1.001)	0.663441985
POSTN	Diagnoses - main ICD10: O63 Long labour	O63	1.000 (0.999, 1.001)	0.741939318
POSTN	Diagnoses - main ICD10: O68 Labour and delivery complicated by foetal stress [distress]	O68	1.000 (0.998, 1.001)	0.615601246
POSTN	Diagnoses - main ICD10: O70 Perineal laceration during delivery	O70	1.000 (0.998, 1.001)	0.694135014
POSTN	Diagnoses - main ICD10: O80 Single spontaneous delivery	O80	1.000 (0.999, 1.001)	0.86595421
POSTN	Diagnoses - main ICD10: R00 Abnormalities of heart beat	R00	1.000 (0.999, 1.002)	0.605449724
POSTN	Diagnoses - main ICD10: R06 Abnormalities of breathing	R06	1.001 (0.999, 1.004)	0.178466056
POSTN	Diagnoses - main ICD10: R19 Other symptoms and signs involving the digestive system and abdomen	R19	0.997 (0.993, 1.000)	0.027391093
POSTN	Diagnoses - main ICD10: R22 Localised swelling, mass and lump of skin and subcutaneous tissue	R22	1.000 (0.999, 1.002)	0.488265153
POSTN	Diagnoses - main ICD10: R39 Other symptoms and signs involving the urinary system	R39	1.001 (1.000, 1.003)	0.153573694
POSTN	Diagnoses - main ICD10: R50 Fever of unknown origin	R50	1.000 (0.999, 1.001)	0.572440055
POSTN	Diagnoses - main ICD10: R56 Convulsions, not elsewhere classified	R56	0.999 (0.998, 1.000)	0.215956372
POSTN	Diagnoses - main ICD10: R59 Enlarged lymph nodes	R59	1.001 (1.000, 1.002)	0.141411565
POSTN	Diagnoses - main ICD10: R63 Symptoms and signs concerning food and fluid intake	R63	1.000 (0.999, 1.002)	0.566410082
POSTN	Diagnoses - main ICD10: R79 Other abnormal findings of blood chemistry	R79	1.000 (0.998, 1.002)	0.89983728
POSTN	Diagnoses - main ICD10: R87 Abnormal findings in specimens from female genital organs	R87	1.000 (0.999, 1.001)	0.851713928
POSTN	Diagnoses - main ICD10: R93 Abnormal findings on diagnostic imaging of other body structures	R93	1.000 (0.999, 1.001)	0.94343402
POSTN	Diagnoses - main ICD10: S01 Open wound of head	S01	0.999 (0.998, 1.000)	0.190938005
POSTN	Diagnoses - main ICD10: S02 Fracture of skull and facial bones	S02	1.000 (0.999, 1.002)	0.538691745
POSTN	Diagnoses - main ICD10: S42 Fracture of shoulder and upper arm	S42	1.000 (0.999, 1.001)	0.9821177
POSTN	Diagnoses - main ICD10: S61 Open wound of wrist and hand	S61	1.000 (0.999, 1.002)	0.70992077
POSTN	Diagnoses - main ICD10: S62 Fracture at wrist and hand level	S62	1.000 (0.999, 1.001)	0.987860599
POSTN	Diagnoses - main ICD10: S72 Fracture of femur	S72	1.000 (0.999, 1.001)	0.927577595
POSTN	Diagnoses - main ICD10: S82 Fracture of lower leg, including ankle	S82	0.999 (0.997, 1.001)	0.275951646
POSTN	Diagnoses - main ICD10: T39 Poisoning by nonopioid analgesics, antipyretics and antirheumatics	T39	0.999 (0.997, 1.000)	0.010150764
POSTN	Diagnoses - main ICD10: T81 Complications of procedures, not elsewhere classified	T81	1.001 (0.999, 1.004)	0.388680086
POSTN	Diagnoses - main ICD10: T82 Complications of cardiac and vascular prosthetic devices, implants and gra	T82	1.000 (0.999, 1.002)	0.418682811
POSTN	Diagnoses - main ICD10: T85 Complications of other internal prosthetic devices, implants and grafts	T85	1.000 (0.999, 1.001)	0.911082714
POSTN	Diagnoses - main ICD10: Z01 Other special examinations and investigations of persons without complain	Z01	1.001 (0.999, 1.002)	0.300144934
POSTN	Diagnoses - main ICD10: Z03 Medical observation and evaluation for suspected diseases and conditions	Z03	0.996 (0.994, 0.999)	0.001756785

POSTN	Diagnoses - main ICD10: Z08 Follow-up examination after treatment for malignant neoplasm	Z08	0.999 (0.997, 1.001)	0.527124959
POSTN	Diagnoses - main ICD10: Z12 Special screening examination for neoplasms	Z12	0.999 (0.997, 1.001)	0.214897638
POSTN	Diagnoses - main ICD10: Z13 Special screening examination for other diseases and disorders	Z13	0.999 (0.998, 1.001)	0.525787005
POSTN	Diagnoses - main ICD10: Z30 Contraceptive management	Z30	1.001 (0.999, 1.004)	0.292930846
POSTN	Diagnoses - main ICD10: Z42 Follow-up care involving plastic surgery	Z42	1.000 (0.999, 1.002)	0.699076248
POSTN	Diagnoses - main ICD10: Z43 Attention to artificial openings	Z43	1.001 (1.000, 1.002)	0.132423082
POSTN	Diagnoses - main ICD10: Z45 Adjustment and management of implanted device	Z45	0.999 (0.997, 1.000)	0.153583755
POSTN	Diagnoses - main ICD10: Z46 Fitting and adjustment of other devices	Z46	0.999 (0.997, 1.001)	0.154077716
POSTN	Diagnoses - main ICD10: Z53 Persons encountering health services for specifie procedures, not carried o	Z53	0.999 (0.997, 1.000)	0.040658978

Table S11 Phenome-wide Mendelian Randomization analysis to reveal potential on-target side effects of RPN1

protein	disease	ICD-10 code	OR (95%CI)	P value
RPN1	Underlying (primary) cause of death: ICD10: E85.4 Organ-limited amyloidosis	E85.4	1.000 (0.997, 1.002)	0.827479325
RPN1	Underlying (primary) cause of death: ICD10: J84.1 Other interstitial pulmonary diseases with fibrosis	J84.1	0.997 (0.988, 1.005)	0.462758705
RPN1	Diagnoses - main ICD10: B37 Candidiasis	B37	1.000 (1.000, 1.000)	0.388812029
RPN1	Diagnoses - main ICD10: C44 Other malignant neoplasms of skin	C44	1.000 (0.998, 1.001)	0.872815781
RPN1	Diagnoses - main ICD10: C50 Malignant neoplasm of breast	C50	1.000 (0.999, 1.002)	0.577415604
RPN1	Diagnoses - main ICD10: C61 Malignant neoplasm of prostate	C61	1.000 (0.999, 1.001)	0.906473073
RPN1	Diagnoses - main ICD10: D12 Benign neoplasm of colon rectum anus and anal canal	D12	1.000 (0.999, 1.002)	0.581170826
RPN1	Diagnoses - main ICD10: D25 Leiomyoma of uterus	D25	0.999 (0.998, 1.001)	0.437408516
RPN1	Diagnoses - main ICD10: E03 Other hypothyroidism	E03	1.000 (1.000, 1.000)	0.385085941
RPN1	Diagnoses - main ICD10: E04 Other non-toxic goitre	E04	1.000 (0.999, 1.000)	0.24154315
RPN1	Diagnoses - main ICD10: F31 Bipolar affective disorder	F31	1.000 (1.000, 1.000)	0.543274445
RPN1	Diagnoses - main ICD10: F43 Reaction to severe stress and adjustment disorders	F43	1.000 (1.000, 1.000)	0.93217342
RPN1	Diagnoses - main ICD10: G47 Sleep disorders	G47	1.001 (1.000, 1.001)	0.234698263
RPN1	Diagnoses - main ICD10: G56 Mononeuropathies of upper limb	G56	1.000 (0.999, 1.002)	0.841037131
RPN1	Diagnoses - main ICD10: H25 Senile cataract	H25	1.001 (1.000, 1.002)	0.193173292
RPN1	Diagnoses - main ICD10: H26 Other cataract	H26	1.000 (0.999, 1.002)	0.553266684
RPN1	Diagnoses - main ICD10: I10 Essential (primary) hypertension	I10	1.000 (0.999, 1.000)	0.692017718
RPN1	Diagnoses - main ICD10: I20 Angina pectoris	I20	1.000 (0.998, 1.001)	0.556191966
RPN1	Diagnoses - main ICD10: I21 Acute myocardial infarction	I21	1.000 (0.999, 1.001)	0.898333805
RPN1	Diagnoses - main ICD10: I25 Chronic ischaemic heart disease	I25	1.001 (0.999, 1.003)	0.413884557
RPN1	Diagnoses - main ICD10: I30 Acute pericarditis	I30	1.000 (1.000, 1.000)	0.815756467
RPN1	Diagnoses - main ICD10: I48 Atrial fibrillation and flutter	I48	0.999 (0.998, 1.000)	0.080997583
RPN1	Diagnoses - main ICD10: I80 Phlebitis and thrombophlebitis	I80	0.999 (0.999, 1.000)	0.159462016
RPN1	Diagnoses - main ICD10: I84 Haemorrhoids	I84	1.000 (0.999, 1.002)	0.617190981
RPN1	Diagnoses - main ICD10: J22 Unspecified acute lower respiratory infection	J22	1.000 (0.999, 1.001)	0.890353513
RPN1	Diagnoses - main ICD10: J33 Nasal polyp	J33	1.000 (0.999, 1.000)	0.438281819
RPN1	Diagnoses - main ICD10: J34 Other disorders of nose and nasal sinuses	J34	1.000 (0.999, 1.001)	0.932791314
RPN1	Diagnoses - main ICD10: J44 Other chronic obstructive pulmonary disease	J44	1.000 (0.999, 1.000)	0.191278497
RPN1	Diagnoses - main ICD10: K20 Oesophagitis	K20	1.000 (0.999, 1.001)	0.616177116
RPN1	Diagnoses - main ICD10: K21 Gastro-oesophageal reflux disease	K21	0.998 (0.997, 1.000)	0.043112059
RPN1	Diagnoses - main ICD10: K22 Other diseases of oesophagus	K22	1.000 (0.998, 1.001)	0.506313569
RPN1	Diagnoses - main ICD10: K29 Gastritis and duodenitis	K29	1.000 (0.999, 1.002)	0.699606674
RPN1	Diagnoses - main ICD10: K35 Acute appendicitis	K35	1.000 (0.999, 1.001)	0.44611846
RPN1	Diagnoses - main ICD10: K40 Inguinal hernia	K40	0.999 (0.997, 1.001)	0.188830694
RPN1	Diagnoses - main ICD10: K43 Ventral hernia	K43	1.000 (0.999, 1.001)	0.530794238

RPN1	Diagnoses - main ICD10: K44 Diaphragmatic hernia	K44	1.000 (0.999, 1.002)	0.743463027
RPN1	Diagnoses - main ICD10: K50 Crohn's disease [regional enteritis]	K50	1.000 (0.999, 1.000)	0.711079969
RPN1	Diagnoses - main ICD10: K51 Ulcerative colitis	K51	1.000 (0.999, 1.001)	0.747169074
RPN1	Diagnoses - main ICD10: K52 Other non-infective gastro-enteritis and colitis	K52	1.001 (0.999, 1.002)	0.341515562
RPN1	Diagnoses - main ICD10: K57 Diverticular disease of intestine	K57	0.999 (0.998, 1.001)	0.510698754
RPN1	Diagnoses - main ICD10: K60 Fissure and fistula of anal and rectal regions	K60	1.000 (0.999, 1.000)	0.385645807
RPN1	Diagnoses - main ICD10: K62 Other diseases of anus and rectum	K62	1.000 (0.998, 1.002)	0.672315984
RPN1	Diagnoses - main ICD10: K76 Other diseases of liver	K76	1.000 (1.000, 1.000)	0.607740809
RPN1	Diagnoses - main ICD10: K80 Cholelithiasis	K80	0.999 (0.997, 1.001)	0.268598624
RPN1	Diagnoses - main ICD10: L03 Cellulitis	L03	0.999 (0.998, 1.001)	0.327751953
RPN1	Diagnoses - main ICD10: M10 Gout	M10	1.000 (1.000, 1.000)	0.59288973
RPN1	Diagnoses - main ICD10: M16 Coxarthrosis [arthrosis of hip]	M16	1.000 (0.999, 1.002)	0.772207718
RPN1	Diagnoses - main ICD10: M17 Gonarthrosis [arthrosis of knee]	M17	1.000 (0.998, 1.001)	0.769959585
RPN1	Diagnoses - main ICD10: M20 Acquired deformities of fingers and toes	M20	0.999 (0.997, 1.000)	0.08643374
RPN1	Diagnoses - main ICD10: M21 Other acquired deformities of limbs	M21	1.000 (1.000, 1.000)	0.741880737
RPN1	Diagnoses - main ICD10: M23 Internal derangement of knee	M23	0.999 (0.997, 1.001)	0.227685789
RPN1	Diagnoses - main ICD10: M24 Other specific joint derangements	M24	0.999 (0.999, 1.000)	0.042786588
RPN1	Diagnoses - main ICD10: M25 Other joint disorders not elsewhere classified	M25	1.001 (0.999, 1.002)	0.313854252
RPN1	Diagnoses - main ICD10: M54 Dorsalgia	M54	0.999 (0.998, 1.001)	0.357341543
RPN1	Diagnoses - main ICD10: M67 Other disorders of synovium and tendon	M67	1.000 (0.999, 1.001)	0.630948157
RPN1	Diagnoses - main ICD10: M70 Soft tissue disorders related to use overuse and pressure	M70	1.000 (0.999, 1.001)	0.960497736
RPN1	Diagnoses - main ICD10: M72 Fibroblastic disorders	M72	0.999 (0.999, 1.000)	0.15949837
RPN1	Diagnoses - main ICD10: N19 Unspecified renal failure	N19	1.000 (1.000, 1.000)	0.966200406
RPN1	Diagnoses - main ICD10: N20 Calculus of kidney and ureter	N20	1.000 (0.999, 1.001)	0.37957817
RPN1	Diagnoses - main ICD10: N32 Other disorders of bladder	N32	1.001 (1.000, 1.002)	0.172210964
RPN1	Diagnoses - main ICD10: N40 Hyperplasia of prostate	N40	1.001 (1.000, 1.002)	0.265059992
RPN1	Diagnoses - main ICD10: N81 Female genital prolapse	N81	1.000 (0.998, 1.001)	0.57004771
RPN1	Diagnoses - main ICD10: N92 Excessive frequent and irregular menstruation	N92	1.003 (1.001, 1.005)	0.000397417
RPN1	Diagnoses - main ICD10: O75 Other complications of labour and delivery not elsewhere classified	O75	1.000 (1.000, 1.000)	0.764937183
RPN1	Diagnoses - main ICD10: R04 Haemorrhage from respiratory passages	R04	1.000 (0.999, 1.000)	0.356042654
RPN1	Diagnoses - main ICD10: R07 Pain in throat and chest	R07	0.998 (0.995, 1.000)	0.110452736
RPN1	Diagnoses - main ICD10: R10 Abdominal and pelvic pain	R10	1.000 (0.998, 1.002)	0.967027876
RPN1	Diagnoses - main ICD10: R11 Nausea and vomiting	R11	1.000 (0.999, 1.001)	0.807845051
RPN1	Diagnoses - main ICD10: R14 Flatulence and related conditions	R14	1.000 (1.000, 1.000)	0.800131895
RPN1	Diagnoses - main ICD10: R31 Unspecified haematuria	R31	1.002 (1.000, 1.004)	0.037161976
RPN1	Diagnoses - main ICD10: R35 Polyuria	R35	1.000 (0.999, 1.001)	0.773494899
RPN1	Diagnoses - main ICD10: R55 Syncope and collapse	R55	1.000 (0.999, 1.001)	0.914129552

RPN1	Diagnoses - main ICD10: R69 Unknown and unspecified causes of morbidity	R69	0.999 (0.997, 1.001)	0.254253233
RPN1	Diagnoses - main ICD10: S09 Other and unspecified injuries of head	S09	1.000 (0.999, 1.000)	0.192633843
RPN1	Diagnoses - main ICD10: S52 Fracture of forearm	S52	1.001 (1.000, 1.002)	0.153802605
RPN1	Diagnoses - main ICD10: S66 Injury of muscle and tendon at wrist and hand level	S66	1.000 (1.000, 1.001)	0.304650215
RPN1	Diagnoses - main ICD10: S76 Injury of muscle and tendon at hip and thigh level	S76	1.000 (1.000, 1.000)	0.232727757
RPN1	Diagnoses - main ICD10: T84 Complications of internal orthopaedic prosthetic devices implants and gra	T84	1.000 (0.999, 1.001)	0.74397809
RPN1	Diagnoses - main ICD10: Z09 Follow-up examination after treatment for conditions other than malignan	Z09	1.000 (0.999, 1.002)	0.642636758
RPN1	Diagnoses - main ICD10: Z47 Other orthopaedic follow-up care	Z47	1.000 (0.999, 1.001)	0.854272072
RPN1	Diagnoses - main ICD10: Z80 Family history of malignant neoplasm	Z80	1.000 (1.000, 1.000)	0.617141501
RPN1	Diagnoses - secondary ICD10: Z50.1 Other physical therapy	Z50.1	1.000 (0.999, 1.000)	0.152668528
RPN1	Diagnoses - main ICD10: Z09.0 Follow-up examination after surgery for other conditions	Z09.0	0.999 (0.998, 1.000)	0.008133779
RPN1	Diagnoses - secondary ICD10: K21.0 Gastro-oesophageal reflux disease with oesophagitis	K21.0	1.001 (1.000, 1.001)	0.136161338
RPN1	Type of cancer: ICD10: C44.3 Skin of other and unspecified parts of face	C44.3	1.000 (0.999, 1.001)	0.940122912
RPN1	Diagnoses - main ICD10: K80.0 Calculus of gallbladder with acute cholecystitis	K80.0	1.000 (0.999, 1.000)	0.298602047
RPN1	Diagnoses - secondary ICD10: R19.4 Change in bowel habit	R19.4	1.000 (0.999, 1.001)	0.917196378
RPN1	Diagnoses - secondary ICD10: R10.1 Pain localised to upper abdomen	R10.1	1.000 (0.999, 1.000)	0.390690971
RPN1	Diagnoses - secondary ICD10: Z03.8 Observation for other suspected diseases and conditions	Z03.8	1.000 (0.999, 1.001)	0.965622298
RPN1	Diagnoses - secondary ICD10: I50.1 Left ventricular failure	I50.1	1.000 (0.999, 1.001)	0.833593207
RPN1	Diagnoses - main ICD10: D12.5 Sigmoid colon	D12.5	1.000 (0.999, 1.001)	0.942577674
RPN1	Diagnoses - main ICD10: Z09.9 Follow-up examination after unspecified treatment for other conditions	Z09.9	1.001 (1.000, 1.001)	0.040805082
RPN1	Diagnoses - main ICD10: N47 Redundant prepuce, phimosis and paraphimosis	N47	1.001 (1.000, 1.001)	0.068734051
RPN1	Diagnoses - secondary ICD10: E10.9 Without complications	E10.9	1.000 (0.999, 1.001)	0.768808087
RPN1	Diagnoses - main ICD10: O70.1 Second degree perineal laceration during delivery	O70.1	1.000 (0.999, 1.000)	0.263258473
RPN1	Diagnoses - secondary ICD10: I20.0 Unstable angina	I20.0	1.000 (0.999, 1.001)	0.796606065
RPN1	Diagnoses - main ICD10: R07.4 Chest pain, unspecified	R07.4	1.000 (0.998, 1.002)	0.770561045
RPN1	Diagnoses - main ICD10: N93.9 Abnormal uterine and vaginal bleeding, unspecified	N93.9	1.000 (0.999, 1.000)	0.4727845
RPN1	Diagnoses - main ICD10: K57.3 Diverticular disease of large intestine without perforation or abscess	K57.3	0.999 (0.998, 1.000)	0.111274173
RPN1	Diagnoses - main ICD10: M54.59 Low back pain (Site unspecified)	M54.59	1.000 (0.999, 1.001)	0.936252244
RPN1	Diagnoses - secondary ICD10: Z87.4 Personal history of diseases of the genito-urinary system	Z87.4	1.000 (0.999, 1.001)	0.407692692
RPN1	Diagnoses - secondary ICD10: Z92.2 Personal history of long-term (current) use of other medicaments	Z92.2	1.000 (0.999, 1.001)	0.813344641
RPN1	Diagnoses - main ICD10: K80.2 Calculus of gallbladder without cholecystitis	K80.2	1.000 (0.999, 1.001)	0.732648808
RPN1	Diagnoses - secondary ICD10: M13.9 Arthritis, unspecified	M13.9	1.000 (1.000, 1.001)	0.518996471
RPN1	Diagnoses - main ICD10: R79.8 Other specified abnormal findings of blood chemistry	R79.8	1.000 (0.999, 1.001)	0.926315287
RPN1	Diagnoses - secondary ICD10: Z95.1 Presence of aortocoronary bypass graft	Z95.1	1.000 (0.999, 1.001)	0.842471207
RPN1	Diagnoses - secondary ICD10: W01.0 Home	W01.0	1.000 (0.999, 1.000)	0.969400203
RPN1	Diagnoses - secondary ICD10: Z85.8 Personal history of malignant neoplasms of other organs and syster	Z85.8	1.000 (0.999, 1.001)	0.743429387
RPN1	Diagnoses - main ICD10: I84.9 Unspecified haemorrhoids without complication	I84.9	1.000 (0.999, 1.002)	0.510923468

RPN1	Diagnoses - main ICD10: K60.2 Anal fissure, unspecified	K60.2	1.000 (1.000, 1.001)	0.801554649
RPN1	Diagnoses - main ICD10: N81.1 Cystocele	N81.1	1.000 (0.999, 1.001)	0.889469852
RPN1	Diagnoses - secondary ICD10: F41.9 Anxiety disorder, unspecified	F41.9	0.999 (0.999, 1.000)	0.012861324
RPN1	Diagnoses - main ICD10: K40.2 Bilateral inguinal hernia, without obstruction or gangrene	K40.2	1.000 (0.999, 1.000)	0.689057822
RPN1	Diagnoses - main ICD10: L90.5 Scar conditions and fibrosis of skin	L90.5	1.000 (0.999, 1.001)	0.932519973
RPN1	Diagnoses - main ICD10: R04.0 Epistaxis	R04.0	1.000 (1.000, 1.001)	0.68896284
RPN1	Diagnoses - secondary ICD10: Z51.1 Chemotherapy session for neoplasm	Z51.1	1.000 (0.999, 1.001)	0.841568262
RPN1	Diagnoses - main ICD10: R32 Unspecified urinary incontinence	R32	1.000 (1.000, 1.001)	0.277098562
RPN1	Diagnoses - main ICD10: N92.0 Excessive and frequent menstruation with regular cycle	N92.0	1.002 (1.000, 1.003)	0.006327083
RPN1	Diagnoses - main ICD10: K62.5 Haemorrhage of anus and rectum	K62.5	1.000 (0.998, 1.001)	0.511354389
RPN1	Diagnoses - secondary ICD10: Z96.1 Presence of intraocular lens	Z96.1	1.000 (0.999, 1.000)	0.520143575
RPN1	Diagnoses - secondary ICD10: R00.1 Bradycardia, unspecified	R00.1	1.000 (0.999, 1.001)	0.990364562
RPN1	Diagnoses - secondary ICD10: K66.0 Peritoneal adhesions	K66.0	1.000 (1.000, 1.001)	0.49805883
RPN1	Diagnoses - main ICD10: T84.8 Other complications of internal orthopaedic prosthetic devices, implants	T84.8	1.000 (0.999, 1.000)	0.290829655
RPN1	Diagnoses - secondary ICD10: Z85.4 Personal history of malignant neoplasm of genital organs	Z85.4	0.999 (0.999, 1.000)	0.155711634
RPN1	Diagnoses - main ICD10: I47.1 Supraventricular tachycardia	I47.1	1.000 (0.999, 1.000)	0.732824658
RPN1	Diagnoses - secondary ICD10: F10.0 Acute intoxication	F10.0	1.000 (1.000, 1.001)	0.489323513
RPN1	Diagnoses - main ICD10: K08.3 Retained dental root	K08.3	1.000 (1.000, 1.001)	0.372130297
RPN1	Diagnoses - secondary ICD10: M06.99 Rheumatoid arthritis, unspecified (Site unspecified)	M06.99	1.000 (0.999, 1.000)	0.582398873
RPN1	Diagnoses - main ICD10: L03.1 Cellulitis of other parts of limb	L03.1	0.999 (0.999, 1.000)	0.242916898
RPN1	Diagnoses - secondary ICD10: R53 Malaise and fatigue	R53	1.001 (1.000, 1.001)	0.020860586
RPN1	Diagnoses - secondary ICD10: W01.9 Unspecified place	W01.9	1.000 (1.000, 1.001)	0.437517823
RPN1	Diagnoses - main ICD10: C50.4 Upper-outer quadrant of breast	C50.4	1.000 (1.000, 1.001)	0.351359053
RPN1	Diagnoses - main ICD10: T39.1 4-Aminophenol derivatives	T39.1	1.000 (1.000, 1.001)	0.216607323
RPN1	Diagnoses - secondary ICD10: K29.5 Chronic gastritis, unspecified	K29.5	1.000 (0.999, 1.000)	0.846066487
RPN1	Diagnoses - secondary ICD10: G55.1 Nerve root and plexus compressions in intervertebral disk disorder	G55.1	1.000 (1.000, 1.001)	0.427371038
RPN1	Diagnoses - secondary ICD10: K25.9 Unspecified as acute or chronic, without haemorrhage or perforatio	K25.9	1.000 (0.999, 1.000)	0.276545155
RPN1	Diagnoses - main ICD10: I80.2 Phlebitis and thrombophlebitis of other deep vessels of lower extremities	I80.2	1.000 (0.999, 1.000)	0.18477274
RPN1	Diagnoses - secondary ICD10: I84.6 Residual haemorrhoidal skin tags	I84.6	1.000 (0.999, 1.000)	0.359398101
RPN1	Diagnoses - main ICD10: N32.0 Bladder-neck obstruction	N32.0	1.000 (1.000, 1.001)	0.273136708
RPN1	Diagnoses - main ICD10: K29.8 Duodenitis	K29.8	1.000 (0.999, 1.000)	0.397053639
RPN1	Diagnoses - main ICD10: M67.4 Ganglion	M67.4	1.000 (0.999, 1.000)	0.243320141
RPN1	Diagnoses - main ICD10: H26.9 Cataract, unspecified	H26.9	1.001 (0.999, 1.002)	0.426213013
RPN1	Type of cancer: ICD10: C20 Malignant neoplasm of rectum	C20	1.001 (1.000, 1.001)	0.005233517
RPN1	Diagnoses - secondary ICD10: K30 Dyspepsia	K30	1.000 (0.999, 1.001)	0.812930511
RPN1	Diagnoses - secondary ICD10: I84.2 Internal haemorrhoids without complication	I84.2	1.000 (1.000, 1.001)	0.25402211
RPN1	Diagnoses - secondary ICD10: N94.6 Dysmenorrhoea, unspecified	N94.6	1.001 (1.000, 1.001)	0.004021782

RPN1	Diagnoses - main ICD10: Z08.0 Follow-up examination after surgery for malignant neoplasm	Z08.0	1.001 (1.000, 1.002)	0.007195296
RPN1	Diagnoses - main ICD10: M79.86 Other specified soft tissue disorders (Lower leg)	M79.86	1.000 (0.999, 1.000)	0.165821136
RPN1	Diagnoses - secondary ICD10: E78.0 Pure hypercholesterolaemia	E78.0	1.000 (0.998, 1.002)	0.833596677
RPN1	Diagnoses - secondary ICD10: M10.99 Gout, unspecified (Site unspecified)	M10.99	1.000 (0.999, 1.000)	0.455482266
RPN1	Diagnoses - main ICD10: M51.2 Other specified intervertebral disk displacement	M51.2	1.000 (1.000, 1.001)	0.162508787
RPN1	Diagnoses - main ICD10: N84.0 Polyp of corpus uteri	N84.0	1.000 (0.999, 1.001)	0.898017513
RPN1	Diagnoses - secondary ICD10: M17.1 Other primary gonarthrosis	M17.1	1.000 (0.999, 1.000)	0.562389569
RPN1	Diagnoses - main ICD10: M25.5 Pain in joint	M25.5	1.000 (1.000, 1.001)	0.333739404
RPN1	Diagnoses - main ICD10: K29.6 Other gastritis	K29.6	1.001 (1.000, 1.001)	0.070537733
RPN1	Type of cancer: ICD10: C64 Malignant neoplasm of kidney, except renal pelvis	C64	1.000 (1.000, 1.001)	0.357138196
RPN1	Diagnoses - main ICD10: G43.9 Migraine, unspecified	G43.9	1.000 (1.000, 1.001)	0.831838053
RPN1	Diagnoses - secondary ICD10: Z80.0 Family history of malignant neoplasm of digestive organs	Z80.0	1.000 (0.999, 1.001)	0.952044075
RPN1	Diagnoses - main ICD10: G45.9 Transient cerebral ischaemic attack, unspecified	G45.9	1.000 (1.000, 1.001)	0.547667493
RPN1	Diagnoses - main ICD10: O02.1 Missed abortion	O02.1	1.000 (0.999, 1.000)	0.382578888
RPN1	Diagnoses - main ICD10: K62.8 Other specified diseases of anus and rectum	K62.8	1.000 (1.000, 1.001)	0.472611295
RPN1	Diagnoses - main ICD10: M72.0 Palmar fascial fibromatosis [Dupuytren]	M72.0	1.000 (0.999, 1.000)	0.396751186
RPN1	Diagnoses - secondary ICD10: I34.0 Mitral (valve) insufficiency	I34.0	1.000 (0.999, 1.000)	0.337467371
RPN1	Diagnoses - main ICD10: O04.9 Complete or unspecified, without complication	O04.9	1.000 (1.000, 1.001)	0.769739471
RPN1	Diagnoses - main ICD10: K22.1 Ulcer of oesophagus	K22.1	1.000 (0.999, 1.001)	0.845559859
RPN1	Diagnoses - secondary ICD10: Z86.7 Personal history of diseases of the circulatory system	Z86.7	1.000 (0.998, 1.001)	0.665509252
RPN1	Diagnoses - secondary ICD10: Y83.1 Surgical operation with implant of artificial internal device	Y83.1	1.000 (0.999, 1.001)	0.586313771
RPN1	Diagnoses - secondary ICD10: R63.4 Abnormal weight loss	R63.4	1.000 (0.999, 1.000)	0.33330489
RPN1	Diagnoses - main ICD10: K21.9 Gastro-oesophageal reflux disease without oesophagitis	K21.9	1.000 (0.999, 1.001)	0.987047825
RPN1	Diagnoses - main ICD10: N93.8 Other specified abnormal uterine and vaginal bleeding	N93.8	1.000 (1.000, 1.001)	0.342927319
RPN1	Diagnoses - main ICD10: M75.4 Impingement syndrome of shoulder	M75.4	1.001 (1.000, 1.001)	0.15420219
RPN1	Diagnoses - main ICD10: I20.9 Angina pectoris, unspecified	I20.9	1.000 (0.999, 1.001)	0.355483908
RPN1	Diagnoses - secondary ICD10: X59.9 Unspecified place	X59.9	1.001 (1.000, 1.002)	0.035637581
RPN1	Diagnoses - main ICD10: I84.1 Internal haemorrhoids with other complications	I84.1	1.000 (0.999, 1.001)	0.918030967
RPN1	Diagnoses - secondary ICD10: K57.9 Diverticular disease of intestine, part unspecified, without perforati	K57.9	1.000 (0.999, 1.001)	0.644975533
RPN1	Diagnoses - main ICD10: L72.9 Follicular cyst of skin and subcutaneous tissue, unspecified	L72.9	1.000 (0.999, 1.000)	0.077518611
RPN1	Diagnoses - secondary ICD10: Z92.3 Personal history of irradiation	Z92.3	1.000 (0.999, 1.000)	0.888521796
RPN1	Diagnoses - main ICD10: Z09.8 Follow-up examination after other treatment for other conditions	Z09.8	1.000 (1.000, 1.001)	0.175394098
RPN1	Diagnoses - main ICD10: K42.9 Umbilical hernia without obstruction or gangrene	K42.9	1.000 (0.999, 1.000)	0.473801035
RPN1	Diagnoses - main ICD10: R07.3 Other chest pain	R07.3	0.999 (0.998, 1.001)	0.300167852
RPN1	Diagnoses - main ICD10: N92.6 Irregular menstruation, unspecified	N92.6	1.000 (1.000, 1.001)	0.471802772
RPN1	Diagnoses - main ICD10: T84.0 Mechanical complication of internal joint prosthesis	T84.0	1.000 (1.000, 1.001)	0.613725827
RPN1	Diagnoses - main ICD10: D64.9 Anaemia, unspecified	D64.9	1.000 (0.999, 1.001)	0.748937887

RPN1	Diagnoses - secondary ICD10: R00.2 Palpitations	R00.2	0.999 (0.999, 1.000)	0.00240492
RPN1	Diagnoses - secondary ICD10: Z90.4 Acquired absence of other parts of digestive tract	Z90.4	1.001 (1.000, 1.001)	0.207820327
RPN1	Type of cancer: ICD10: C54.1 Endometrium	C54.1	1.001 (1.000, 1.001)	0.046118558
RPN1	Diagnoses - main ICD10: K92.2 Gastro-intestinal haemorrhage, unspecified	K92.2	1.000 (1.000, 1.001)	0.286509562
RPN1	Diagnoses - main ICD10: L72.0 Epidermal cyst	L72.0	1.000 (1.000, 1.001)	0.387623774
RPN1	Diagnoses - secondary ICD10: M13.99 Arthritis, unspecified (Site unspecified)	M13.99	1.000 (0.999, 1.001)	0.74298474
RPN1	Diagnoses - secondary ICD10: Z95.8 Presence of other cardiac and vascular implants and grafts	Z95.8	1.000 (0.999, 1.000)	0.340475658
RPN1	Diagnoses - secondary ICD10: K29.7 Gastritis, unspecified	K29.7	1.001 (1.000, 1.002)	0.075243294
RPN1	Diagnoses - secondary ICD10: E66.9 Obesity, unspecified	E66.9	1.000 (0.999, 1.001)	0.497615749
RPN1	Diagnoses - main ICD10: M54.5 Low back pain	M54.5	1.000 (0.999, 1.001)	0.938246229
RPN1	Diagnoses - main ICD10: J18.9 Pneumonia, unspecified	J18.9	1.000 (0.999, 1.000)	0.416699425
RPN1	Type of cancer: ICD10: C44.5 Skin of trunk	C44.5	1.000 (0.999, 1.000)	0.512254679
RPN1	Diagnoses - secondary ICD10: Z95.5 Presence of coronary angioplasty implant and graft	Z95.5	1.001 (1.000, 1.002)	0.034415536
RPN1	Type of cancer: ICD10: C18.7 Sigmoid colon	C18.7	1.000 (1.000, 1.001)	0.52141446
RPN1	Diagnoses - main ICD10: R87.6 Abnormal cytological findings	R87.6	1.000 (0.999, 1.000)	0.332551432
RPN1	Diagnoses - secondary ICD10: Z86.4 Personal history of psychoactive substance abuse	Z86.4	1.001 (0.999, 1.002)	0.387334352
RPN1	Diagnoses - secondary ICD10: Z51.5 Palliative care	Z51.5	1.000 (0.999, 1.001)	0.964401543
RPN1	Diagnoses - secondary ICD10: G40.9 Epilepsy, unspecified	G40.9	1.000 (0.999, 1.001)	0.775460786
RPN1	Diagnoses - secondary ICD10: Z51.0 Radiotherapy session	Z51.0	1.000 (1.000, 1.001)	0.229576435
RPN1	Diagnoses - secondary ICD10: F10.1 Harmful use	F10.1	1.000 (0.999, 1.000)	0.161511519
RPN1	Diagnoses - secondary ICD10: I51.7 Cardiomegaly	I51.7	1.000 (0.999, 1.001)	0.936039395
RPN1	Diagnoses - main ICD10: N23 Unspecified renal colic	N23	1.000 (0.999, 1.000)	0.437246283
RPN1	Diagnoses - secondary ICD10: E86 Volume depletion	E86	1.000 (1.000, 1.001)	0.635420097
RPN1	Diagnoses - secondary ICD10: Z87.1 Personal history of diseases of the digestive system	Z87.1	1.001 (1.000, 1.003)	0.167052639
RPN1	Diagnoses - secondary ICD10: Z85.0 Personal history of malignant neoplasm of digestive organs	Z85.0	1.001 (1.000, 1.002)	0.104312646
RPN1	Diagnoses - secondary ICD10: R03.0 Elevated blood-pressure reading, without diagnosis of hypertensio	R03.0	1.000 (1.000, 1.001)	0.288486822
RPN1	Diagnoses - secondary ICD10: I25.8 Other forms of chronic ischaemic heart disease	I25.8	1.000 (0.999, 1.001)	0.623667596
RPN1	Diagnoses - secondary ICD10: Z90.1 Acquired absence of breast(s)	Z90.1	1.000 (0.999, 1.001)	0.962375262
RPN1	Diagnoses - secondary ICD10: I25.2 Old myocardial infarction	I25.2	1.000 (0.999, 1.001)	0.766072881
RPN1	Diagnoses - main ICD10: I25.1 Atherosclerotic heart disease	I25.1	1.001 (0.999, 1.003)	0.179714249
RPN1	Diagnoses - secondary ICD10: Z91.0 Personal history of allergy, other than to drugs and biological subs	Z91.0	1.000 (0.999, 1.001)	0.852399023
RPN1	Diagnoses - secondary ICD10: C78.7 Secondary malignant neoplasm of liver	C78.7	1.000 (0.999, 1.000)	0.599209609
RPN1	Diagnoses - secondary ICD10: J44.9 Chronic obstructive pulmonary disease, unspecified	J44.9	0.999 (0.998, 1.000)	0.012999041
RPN1	Diagnoses - main ICD10: G47.3 Sleep apnoea	G47.3	1.001 (1.000, 1.001)	0.145182948
RPN1	Diagnoses - secondary ICD10: N95.0 Postmenopausal bleeding	N95.0	1.000 (0.999, 1.001)	0.946979777
RPN1	Diagnoses - main ICD10: S09.9 Unspecified injury of head	S09.9	1.000 (1.000, 1.001)	0.649282673
RPN1	Diagnoses - secondary ICD10: Z95.0 Presence of cardiac pacemaker	Z95.0	1.000 (1.000, 1.001)	0.650021349

RPN1	Diagnoses - secondary ICD10: C77.3 Axillary and upper limb lymph nodes	C77.3	1.000 (0.999, 1.001)	0.882595384
RPN1	Type of cancer: ICD10: C44.4 Skin of scalp and neck	C44.4	1.000 (0.999, 1.001)	0.979834224
RPN1	Diagnoses - secondary ICD10: J45.9 Asthma, unspecified	J45.9	0.999 (0.997, 1.001)	0.319518991
RPN1	Diagnoses - main ICD10: R39.8 Other and unspecified symptoms and signs involving the urinary system	R39.8	1.000 (0.999, 1.000)	0.442443216
RPN1	Diagnoses - secondary ICD10: R00.0 Tachycardia, unspecified	R00.0	1.000 (0.999, 1.000)	0.669167485
RPN1	Diagnoses - main ICD10: R10.3 Pain localised to other parts of lower abdomen	R10.3	1.000 (0.999, 1.001)	0.367845142
RPN1	Diagnoses - secondary ICD10: E78.5 Hyperlipidaemia, unspecified	E78.5	1.000 (0.999, 1.001)	0.933140292
RPN1	Diagnoses - secondary ICD10: E14.9 Without complications	E14.9	1.000 (1.000, 1.001)	0.786319135
RPN1	Diagnoses - main ICD10: T81.0 Haemorrhage and haematoma complicating a procedure, not elsewhere	T81.0	1.000 (0.999, 1.000)	0.648141587
RPN1	Diagnoses - secondary ICD10: J18.1 Lobar pneumonia, unspecified	J18.1	1.000 (1.000, 1.001)	0.69939787
RPN1	Diagnoses - secondary ICD10: M81.99 Osteoporosis, unspecified (Site unspecified)	M81.99	1.000 (0.999, 1.000)	0.41219591
RPN1	Diagnoses - secondary ICD10: Y83.8 Other surgical procedures	Y83.8	1.001 (1.000, 1.002)	0.255308942
RPN1	Diagnoses - secondary ICD10: K63.5 Polyp of colon	K63.5	1.000 (0.999, 1.001)	0.799715684
RPN1	Diagnoses - main ICD10: K58.9 Irritable bowel syndrome without diarrhoea	K58.9	1.000 (1.000, 1.001)	0.880661749
RPN1	Diagnoses - secondary ICD10: K59.0 Constipation	K59.0	1.000 (0.999, 1.001)	0.618962869
RPN1	Diagnoses - secondary ICD10: Z87.3 Personal history of diseases of the musculoskeletal system and conn	Z87.3	1.000 (1.000, 1.001)	0.209914798
RPN1	Type of cancer: ICD10: C56 Malignant neoplasm of ovary	C56	1.000 (0.999, 1.000)	0.489285064
RPN1	Diagnoses - main ICD10: M51.1 Lumbar and other intervertebral disk disorders with radiculopathy	M51.1	1.000 (0.999, 1.000)	0.575047562
RPN1	Diagnoses - main ICD10: I26.9 Pulmonary embolism without mention of acute cor pulmonale	I26.9	1.000 (1.000, 1.001)	0.165431947
RPN1	Diagnoses - main ICD10: N20.0 Calculus of kidney	N20.0	1.000 (0.999, 1.000)	0.442294894
RPN1	Diagnoses - main ICD10: M17.9 Gonarthrosis, unspecified	M17.9	1.000 (0.999, 1.001)	0.980494397
RPN1	Diagnoses - main ICD10: K92.0 Haematemesis	K92.0	1.000 (0.999, 1.000)	0.488146542
RPN1	Diagnoses - main ICD10: N20.1 Calculus of ureter	N20.1	1.000 (0.999, 1.000)	0.138198084
RPN1	Diagnoses - main ICD10: K13.7 Other and unspecified lesions of oral mucosa	K13.7	1.000 (1.000, 1.001)	0.889194255
RPN1	Type of cancer: ICD10: C34.1 Upper lobe, bronchus or lung	C34.1	1.000 (1.000, 1.001)	0.118547438
RPN1	Diagnoses - main ICD10: Z42.1 Follow-up care involving plastic surgery of breast	Z42.1	1.000 (0.999, 1.001)	0.972992785
RPN1	Type of cancer: ICD10: C50.9 Breast, unspecified	C50.9	1.001 (1.000, 1.002)	0.160256358
RPN1	Diagnoses - main ICD10: J34.8 Other specified disorders of nose and nasal sinuses	J34.8	1.000 (1.000, 1.001)	0.513214118
RPN1	Type of cancer: ICD10: C44.6 Skin of upper limb, including shoulder	C44.6	1.000 (0.999, 1.000)	0.827486243
RPN1	Diagnoses - secondary ICD10: R50.9 Fever, unspecified	R50.9	0.999 (0.999, 1.000)	0.102676608
RPN1	Diagnoses - secondary ICD10: W10.0 Home	W10.0	1.000 (1.000, 1.001)	0.950517407
RPN1	Diagnoses - main ICD10: N84.1 Polyp of cervix uteri	N84.1	1.000 (0.999, 1.001)	0.707809473
RPN1	Diagnoses - secondary ICD10: R33 Retention of urine	R33	1.000 (0.999, 1.001)	0.692219056
RPN1	Diagnoses - main ICD10: D12.6 Colon, unspecified	D12.6	1.000 (1.000, 1.001)	0.443600131
RPN1	Diagnoses - main ICD10: I63.9 Cerebral infarction, unspecified	I63.9	0.999 (0.999, 1.000)	0.034089079
RPN1	Diagnoses - main ICD10: K51.9 Ulcerative colitis, unspecified	K51.9	1.000 (0.999, 1.001)	0.968926426
RPN1	Diagnoses - main ICD10: K85 Acute pancreatitis	K85	1.000 (1.000, 1.001)	0.72657967

RPN1	Diagnoses - secondary ICD10: R06.0 Dyspnoea	R06.0	1.000 (0.999, 1.001)	0.974998664
RPN1	Diagnoses - secondary ICD10: R10.4 Other and unspecified abdominal pain	R10.4	1.000 (0.999, 1.001)	0.375270646
RPN1	Diagnoses - main ICD10: Z47.0 Follow-up care involving removal of fracture plate and other internal fi	Z47.0	1.000 (0.999, 1.001)	0.936058815
RPN1	Diagnoses - secondary ICD10: F10.2 Dependence syndrome	F10.2	1.000 (1.000, 1.001)	0.797112218
RPN1	Diagnoses - main ICD10: R06.5 Mouth breathing	R06.5	1.000 (0.999, 1.000)	0.442051604
RPN1	Diagnoses - main ICD10: R51 Headache	R51	1.000 (0.999, 1.001)	0.580668547
RPN1	Diagnoses - main ICD10: K62.1 Rectal polyp	K62.1	1.000 (1.000, 1.001)	0.222541471
RPN1	Diagnoses - main ICD10: M51.3 Other specified intervertebral disk degeneration	M51.3	1.000 (0.999, 1.000)	0.139425171
RPN1	Diagnoses - main ICD10: M54.56 Low back pain (Lumbar region)	M54.56	1.000 (1.000, 1.001)	0.779075862
RPN1	Diagnoses - main ICD10: M16.9 Coxarthrosis, unspecified	M16.9	1.000 (0.999, 1.001)	0.649763013
RPN1	Diagnoses - secondary ICD10: Z86.1 Personal history of infectious and parasitic diseases	Z86.1	1.000 (1.000, 1.001)	0.338443021
RPN1	Diagnoses - secondary ICD10: I12.0 Hypertensive renal disease with renal failure	I12.0	1.000 (0.999, 1.000)	0.766994796
RPN1	Diagnoses - secondary ICD10: R05 Cough	R05	1.000 (1.000, 1.001)	0.583621805
RPN1	Diagnoses - main ICD10: Z13.8 Special screening examination for other specified diseases and disorders	Z13.8	1.000 (0.999, 1.000)	0.559229852
RPN1	Diagnoses - secondary ICD10: F32.9 Depressive episode, unspecified	F32.9	1.000 (0.999, 1.001)	0.881760389
RPN1	Diagnoses - secondary ICD10: Z85.3 Personal history of malignant neoplasm of breast	Z85.3	1.001 (1.000, 1.002)	0.127458926
RPN1	Diagnoses - main ICD10: K52.9 Non-infective gastro-enteritis and colitis, unspecified	K52.9	1.001 (0.999, 1.002)	0.292560345
RPN1	Diagnoses - main ICD10: L98.9 Disorder of skin and subcutaneous tissue, unspecified	L98.9	1.000 (0.999, 1.001)	0.788738521
RPN1	Diagnoses - secondary ICD10: M19.99 Arthrosis, unspecified (Site unspecified)	M19.99	1.000 (1.000, 1.001)	0.458462248
RPN1	Diagnoses - main ICD10: Z12.1 Special screening examination for neoplasm of intestinal tract	Z12.1	1.001 (1.000, 1.002)	0.059514794
RPN1	Diagnoses - main ICD10: R07.2 Precordial pain	R07.2	1.000 (0.999, 1.000)	0.435550277
RPN1	Diagnoses - secondary ICD10: Z90.7 Acquired absence of genital organ(s)	Z90.7	1.000 (0.999, 1.001)	0.472549421
RPN1	Diagnoses - secondary ICD10: Z96.6 Presence of orthopaedic joint implants	Z96.6	1.000 (0.999, 1.001)	0.796272268
RPN1	Diagnoses - secondary ICD10: B96.8 Other specified bacterial agents as the cause of diseases classified to	B96.8	1.000 (0.999, 1.001)	0.607749333
RPN1	Diagnoses - secondary ICD10: R42 Dizziness and giddiness	R42	1.000 (0.999, 1.001)	0.958090982
RPN1	Diagnoses - secondary ICD10: N81.2 Incomplete uterovaginal prolapse	N81.2	1.000 (0.999, 1.000)	0.117864701
RPN1	Diagnoses - main ICD10: M25.56 Pain in joint (Lower leg)	M25.56	1.000 (1.000, 1.001)	0.588665103
RPN1	Diagnoses - main ICD10: D17.2 Benign lipomatous neoplasm of skin and subcutaneous tissue of limbs	D17.2	1.000 (0.999, 1.000)	0.321100268
RPN1	Diagnoses - main ICD10: O63.1 Prolonged second stage (of labour)	O63.1	1.000 (0.999, 1.000)	0.110439768
RPN1	Diagnoses - secondary ICD10: Z82.4 Family history of ischaemic heart disease and other diseases of the	Z82.4	1.000 (0.998, 1.001)	0.740530121
RPN1	Diagnoses - main ICD10: H25.0 Senile incipient cataract	H25.0	1.000 (1.000, 1.001)	0.33961022
RPN1	Diagnoses - main ICD10: D17.1 Benign lipomatous neoplasm of skin and subcutaneous tissue of trunk	D17.1	1.000 (0.999, 1.001)	0.837827292
RPN1	Diagnoses - secondary ICD10: M47.82 Other spondylosis (Cervical region)	M47.82	1.000 (1.000, 1.000)	0.975206875
RPN1	Diagnoses - secondary ICD10: Z98.0 Intestinal bypass and anastomosis status	Z98.0	1.000 (1.000, 1.001)	0.805948578
RPN1	Type of cancer: ICD10: C43.7 Malignant melanoma of lower limb, including hip	C43.7	1.000 (1.000, 1.001)	0.122997798
RPN1	Diagnoses - main ICD10: S52.50 Fracture of lower end of radius (closed)	S52.50	1.000 (0.999, 1.001)	0.93825113
RPN1	Diagnoses - secondary ICD10: W19.9 Unspecified place	W19.9	1.000 (0.999, 1.000)	0.320579457

RPN1	Diagnoses - main ICD10: M16.1 Other primary coxarthrosis	M16.1	1.000 (0.999, 1.001)	0.948401995
RPN1	Diagnoses - secondary ICD10: Z60.2 Living alone	Z60.2	1.001 (1.000, 1.001)	0.217948423
RPN1	Diagnoses - secondary ICD10: F17.1 Harmful use	F17.1	1.000 (0.999, 1.000)	0.150371694
RPN1	Diagnoses - secondary ICD10: K31.8 Other specified diseases of stomach and duodenum	K31.8	1.000 (0.999, 1.000)	0.175534774
RPN1	Diagnoses - secondary ICD10: K31.7 Polyp of stomach and duodenum	K31.7	1.000 (0.999, 1.000)	0.071342661
RPN1	Diagnoses - main ICD10: H25.1 Senile nuclear cataract	H25.1	1.001 (1.000, 1.001)	0.111553699
RPN1	Diagnoses - secondary ICD10: Y83.2 Surgical operation with anastomosis, bypass or graft	Y83.2	1.000 (1.000, 1.001)	0.810922309
RPN1	Diagnoses - secondary ICD10: D70 Agranulocytosis	D70	1.000 (1.000, 1.001)	0.513498709
RPN1	Diagnoses - main ICD10: D50.9 Iron deficiency anaemia, unspecified	D50.9	1.000 (0.999, 1.001)	0.766827347
RPN1	Diagnoses - secondary ICD10: N81.6 Rectocele	N81.6	1.000 (0.999, 1.000)	0.34105276
RPN1	Diagnoses - main ICD10: I21.9 Acute myocardial infarction, unspecified	I21.9	1.000 (1.000, 1.001)	0.278461923
RPN1	Diagnoses - main ICD10: D22.3 Melanocytic naevi of other and unspecified parts of face	D22.3	1.000 (0.999, 1.000)	0.524210274
RPN1	Diagnoses - secondary ICD10: K44.9 Diaphragmatic hernia without obstruction or gangrene	K44.9	0.999 (0.998, 1.001)	0.538858434
RPN1	Diagnoses - main ICD10: B34.9 Viral infection, unspecified	B34.9	1.000 (0.999, 1.000)	0.307615906
RPN1	Diagnoses - main ICD10: N32.8 Other specified disorders of bladder	N32.8	1.000 (1.000, 1.001)	0.463920172
RPN1	Diagnoses - main ICD10: J34.2 Deviated nasal septum	J34.2	1.000 (0.999, 1.001)	0.626392542
RPN1	Diagnoses - main ICD10: N50.8 Other specified disorders of male genital organs	N50.8	1.000 (0.999, 1.001)	0.904771276
RPN1	Diagnoses - secondary ICD10: Z86.0 Personal history of other neoplasms	Z86.0	1.000 (0.999, 1.000)	0.360735525
RPN1	Diagnoses - secondary ICD10: Z72.1 Alcohol use	Z72.1	1.000 (0.999, 1.000)	0.369222833
RPN1	Diagnoses - main ICD10: G56.0 Carpal tunnel syndrome	G56.0	1.000 (0.999, 1.002)	0.675428775
RPN1	Diagnoses - main ICD10: I25.9 Chronic ischaemic heart disease, unspecified	I25.9	1.000 (0.999, 1.000)	0.331975733
RPN1	Diagnoses - secondary ICD10: Z37.0 Single live birth	Z37.0	1.000 (0.998, 1.001)	0.666985346
RPN1	Diagnoses - secondary ICD10: E03.9 Hypothyroidism, unspecified	E03.9	0.998 (0.997, 1.000)	0.021368792
RPN1	Diagnoses - secondary ICD10: Z88.6 Personal history of allergy to analgesic agent	Z88.6	0.999 (0.999, 1.000)	0.087438921
RPN1	Diagnoses - main ICD10: M20.2 Hallux rigidus	M20.2	1.000 (0.999, 1.000)	0.139775719
RPN1	Diagnoses - secondary ICD10: B95.6 Staphylococcus aureus as the cause of diseases classified to other ch	B95.6	1.000 (0.999, 1.001)	0.760580444
RPN1	Diagnoses - main ICD10: R04.2 Haemoptysis	R04.2	1.000 (0.999, 1.000)	0.942545935
RPN1	Diagnoses - main ICD10: I21.0 Acute transmural myocardial infarction of anterior wall	I21.0	1.000 (0.999, 1.000)	0.644254866
RPN1	Diagnoses - secondary ICD10: R94.5 Abnormal results of liver function studies	R94.5	1.000 (0.999, 1.001)	0.70235441
RPN1	Diagnoses - secondary ICD10: T81.4 Infection following a procedure, not elsewhere classified	T81.4	1.000 (0.999, 1.001)	0.816199612
RPN1	Diagnoses - secondary ICD10: Z88.8 Personal history of allergy to other drugs, medicaments and biolog	Z88.8	1.000 (0.999, 1.001)	0.608835788
RPN1	Diagnoses - main ICD10: H00.1 Chalazion	H00.1	1.000 (0.999, 1.001)	0.878191116
RPN1	Type of cancer: ICD10: D05.1 Intraductal carcinoma in situ	D05.1	1.000 (1.000, 1.001)	0.551975575
RPN1	Diagnoses - secondary ICD10: Y43.3 Other antineoplastic drugs	Y43.3	1.000 (0.999, 1.000)	0.257706994
RPN1	Diagnoses - main ICD10: K02.9 Dental caries, unspecified	K02.9	1.000 (1.000, 1.001)	0.192619461
RPN1	Diagnoses - main ICD10: M72.04 Palmar fascial fibromatosis [Dupuytren]-Hand	M72.04	1.000 (1.000, 1.001)	0.662891829
RPN1	Diagnoses - secondary ICD10: N83.2 Other and unspecified ovarian cysts	N83.2	1.000 (0.999, 1.001)	0.848038789

RPN1	Diagnoses - main ICD10: M75.0 Adhesive capsulitis of shoulder	M75.0	1.000 (1.000, 1.001)	0.558681018
RPN1	Diagnoses - secondary ICD10: I73.9 Peripheral vascular disease, unspecified	I73.9	1.000 (0.999, 1.001)	0.871248597
RPN1	Diagnoses - secondary ICD10: N17.9 Acute renal failure, unspecified	N17.9	1.000 (1.000, 1.001)	0.614808058
RPN1	Diagnoses - main ICD10: M75.1 Rotator cuff syndrome	M75.1	1.001 (1.000, 1.001)	0.105782383
RPN1	Diagnoses - main ICD10: L82 Seborrhoeic keratosis	L82	0.999 (0.999, 1.000)	0.0886484
RPN1	Diagnoses - main ICD10: K61.0 Anal abscess	K61.0	1.000 (0.999, 1.000)	0.136469575
RPN1	Diagnoses - secondary ICD10: Z53.2 Procedure not carried out because of patient's decision for other an	Z53.2	1.000 (0.999, 1.000)	0.511889881
RPN1	Diagnoses - secondary ICD10: Z72.0 Tobacco use	Z72.0	1.000 (0.998, 1.001)	0.780693974
RPN1	Diagnoses - main ICD10: I21.1 Acute transmural myocardial infarction of inferior wall	I21.1	1.000 (1.000, 1.001)	0.480401929
RPN1	Diagnoses - secondary ICD10: N39.3 Stress incontinence	N39.3	1.000 (0.999, 1.001)	0.911629673
RPN1	Diagnoses - main ICD10: K40.9 Unilateral or unspecified inguinal hernia, without obstruction or gangre	K40.9	0.999 (0.998, 1.001)	0.292255186
RPN1	Diagnoses - main ICD10: C67.9 Bladder, unspecified	C67.9	1.001 (1.000, 1.001)	0.001996805
RPN1	Diagnoses - secondary ICD10: Z13.0 Special screening examination for diseases of the blood and blood-	Z13.0	1.000 (1.000, 1.001)	0.903406461
RPN1	Diagnoses - secondary ICD10: Z86.6 Personal history of diseases of the nervous system and sense organs	Z86.6	1.000 (0.999, 1.001)	0.914266056
RPN1	Diagnoses - main ICD10: K92.1 Melaena	K92.1	1.000 (0.999, 1.000)	0.679368751
RPN1	Diagnoses - secondary ICD10: J34.3 Hypertrophy of nasal turbinates	J34.3	1.000 (1.000, 1.001)	0.116200454
RPN1	Diagnoses - secondary ICD10: Z88.0 Personal history of allergy to penicillin	Z88.0	0.999 (0.998, 1.001)	0.341360344
RPN1	Diagnoses - main ICD10: S82.80 Fractures of other parts of lower leg (closed)	S82.80	1.000 (0.999, 1.000)	0.586297509
RPN1	Diagnoses - main ICD10: M20.1 Hallux valgus (acquired)	M20.1	0.999 (0.998, 1.000)	0.150862742
RPN1	Diagnoses - secondary ICD10: N39.0 Urinary tract infection, site not specified	N39.0	1.000 (0.999, 1.001)	0.584587655
RPN1	Diagnoses - secondary ICD10: Z03.4 Observation for suspected myocardial infarction	Z03.4	1.000 (0.999, 1.001)	0.903836092
RPN1	Diagnoses - main ICD10: O80.0 Spontaneous vertex delivery	O80.0	1.000 (0.999, 1.001)	0.792710281
RPN1	Diagnoses - secondary ICD10: N73.6 Female pelvic peritoneal adhesions	N73.6	1.000 (0.999, 1.001)	0.734222603
RPN1	Diagnoses - secondary ICD10: D25.9 Leiomyoma of uterus, unspecified	D25.9	1.000 (0.999, 1.001)	0.771651437
RPN1	Diagnoses - secondary ICD10: K26.9 Unspecified as acute or chronic, without haemorrhage or perforatio	K26.9	1.001 (1.000, 1.001)	0.020136667
RPN1	Diagnoses - main ICD10: K60.3 Anal fistula	K60.3	1.000 (0.999, 1.000)	0.463282858
RPN1	Diagnoses - main ICD10: K43.9 Ventral hernia without obstruction or gangrene	K43.9	1.000 (0.999, 1.000)	0.322222114
RPN1	Diagnoses - main ICD10: K35.9 Acute appendicitis, unspecified	K35.9	1.000 (1.000, 1.001)	0.246707307
RPN1	Type of cancer: ICD10: C44.7 Skin of lower limb, including hip	C44.7	1.000 (0.999, 1.000)	0.350681929
RPN1	Diagnoses - secondary ICD10: M15.9 Polyarthrosis, unspecified	M15.9	1.000 (1.000, 1.001)	0.428130544
RPN1	Diagnoses - main ICD10: R13 Dysphagia	R13	0.999 (0.999, 1.000)	0.152342018
RPN1	Diagnoses - main ICD10: M23.23 Derangement of meniscus due to old tear or injury (Medial collateral l	M23.23	1.000 (0.999, 1.000)	0.339021497
RPN1	Diagnoses - secondary ICD10: Z51.2 Other chemotherapy	Z51.2	1.000 (0.999, 1.000)	0.39452223
RPN1	Diagnoses - main ICD10: J33.9 Nasal polyp, unspecified	J33.9	1.000 (0.999, 1.000)	0.524404031
RPN1	Diagnoses - secondary ICD10: Z85.5 Personal history of malignant neoplasm of urinary tract	Z85.5	1.001 (1.000, 1.002)	0.008670521
RPN1	Diagnoses - secondary ICD10: Z92.1 Personal history of long-term (current) use of anticoagulants	Z92.1	1.000 (0.999, 1.001)	0.56552573
RPN1	Diagnoses - main ICD10: Z30.2 Sterilisation	Z30.2	1.000 (0.999, 1.002)	0.651965225

RPN1	Diagnoses - main ICD10: N63 Unspecified lump in breast	N63	1.000 (0.999, 1.001)	0.832566663
RPN1	Diagnoses - main ICD10: I84.8 Unspecified haemorrhoids with other complications	I84.8	1.000 (0.999, 1.000)	0.49538531
RPN1	Diagnoses - main ICD10: Z46.6 Fitting and adjustment of urinary device	Z46.6	1.000 (1.000, 1.001)	0.406217344
RPN1	Diagnoses - main ICD10: K01.1 Impacted teeth	K01.1	1.000 (0.999, 1.001)	0.887737166
RPN1	Diagnoses - secondary ICD10: Z80.3 Family history of malignant neoplasm of breast	Z80.3	1.000 (1.000, 1.001)	0.774155503
RPN1	Diagnoses - secondary ICD10: Y83.6 Removal of other organ (partial) (total)	Y83.6	1.000 (0.999, 1.000)	0.264713034
RPN1	Diagnoses - main ICD10: M65.3 Trigger finger	M65.3	1.000 (1.000, 1.001)	0.32335582
RPN1	Diagnoses - secondary ICD10: Z92.4 Personal history of major surgery, not elsewhere classified	Z92.4	1.000 (1.000, 1.001)	0.424122323
RPN1	Diagnoses - main ICD10: M23.2 Derangement of meniscus due to old tear or injury	M23.2	1.000 (0.999, 1.000)	0.444340994
RPN1	Diagnoses - main ICD10: K80.5 Calculus of bile duct without cholangitis or cholecystitis	K80.5	1.000 (1.000, 1.001)	0.202062877
RPN1	Type of cancer: ICD10: C44.9 Malignant neoplasm of skin, unspecified	C44.9	1.000 (0.999, 1.001)	0.659948413
RPN1	Diagnoses - main ICD10: D24 Benign neoplasm of breast	D24	1.000 (1.000, 1.001)	0.260922393
RPN1	Diagnoses - secondary ICD10: N85.8 Other specified noninflammatory disorders of uterus	N85.8	1.000 (1.000, 1.001)	0.447567036
RPN1	Diagnoses - secondary ICD10: I95.9 Hypotension, unspecified	I95.9	1.000 (0.999, 1.001)	0.794659581
RPN1	Diagnoses - secondary ICD10: B96.2 Escherichia coli [E. coli] as the cause of diseases classified to other	B96.2	1.000 (1.000, 1.001)	0.551954307
RPN1	Diagnoses - main ICD10: K80.1 Calculus of gallbladder with other cholecystitis	K80.1	0.999 (0.998, 1.000)	0.0542157
RPN1	Diagnoses - secondary ICD10: Z53.0 Procedure not carried out because of contraindication	Z53.0	1.000 (0.999, 1.001)	0.741677709
RPN1	Diagnoses - secondary ICD10: Z53.8 Procedure not carried out for other reasons	Z53.8	1.000 (0.999, 1.002)	0.780380095
RPN1	Diagnoses - main ICD10: N35.9 Urethral stricture, unspecified	N35.9	1.000 (0.999, 1.001)	0.660446216
RPN1	Type of cancer: ICD10: D06.9 Cervix, unspecified	D06.9	1.000 (1.000, 1.001)	0.253903609
RPN1	Diagnoses - secondary ICD10: Z53.9 Procedure not carried out, unspecified reason	Z53.9	1.000 (0.999, 1.001)	0.920717454
RPN1	Diagnoses - main ICD10: O68.0 Labour and delivery complicated by foetal heart rate anomaly	O68.0	1.000 (1.000, 1.000)	0.980408004
RPN1	Diagnoses - main ICD10: D12.8 Rectum	D12.8	1.000 (0.999, 1.001)	0.987712793
RPN1	Diagnoses - secondary ICD10: M19.9 Arthrosis, unspecified	M19.9	1.000 (1.000, 1.001)	0.59151336
RPN1	Diagnoses - main ICD10: H02.8 Other specified disorders of eyelid	H02.8	1.000 (1.000, 1.001)	0.503075266
RPN1	Diagnoses - main ICD10: N92.1 Excessive and frequent menstruation with irregular cycle	N92.1	1.000 (1.000, 1.001)	0.217217422
RPN1	Diagnoses - main ICD10: O70.0 First degree perineal laceration during delivery	O70.0	1.000 (0.999, 1.000)	0.736906511
RPN1	Diagnoses - secondary ICD10: N80.0 Endometriosis of uterus	N80.0	1.000 (1.000, 1.001)	0.203236511
RPN1	Diagnoses - main ICD10: M23.22 Derangement of meniscus due to old tear or injury (Posterior cruciate	M23.22	1.000 (0.999, 1.000)	0.565210558
RPN1	Diagnoses - secondary ICD10: E11.9 Without complications	E11.9	1.000 (0.999, 1.002)	0.832001028
RPN1	Diagnoses - main ICD10: M79.66 Pain in limb (Lower leg)	M79.66	1.000 (0.999, 1.000)	0.337651192
RPN1	Diagnoses - main ICD10: O26.8 Other specified pregnancy-related conditions	O26.8	1.000 (0.999, 1.000)	0.611647169
RPN1	Diagnoses - main ICD10: K56.6 Other and unspecified intestinal obstruction	K56.6	1.000 (1.000, 1.001)	0.136058437
RPN1	Diagnoses - secondary ICD10: Z88.1 Personal history of allergy to other antibiotic agents	Z88.1	1.000 (0.999, 1.001)	0.754026453
RPN1	Diagnoses - secondary ICD10: J90 Pleural effusion, not elsewhere classified	J90	1.000 (0.999, 1.001)	0.759097434
RPN1	Underlying (primary) cause of death: ICD10: C34.9 Bronchus or lung, unspecified	C34.9	0.987 (0.964, 1.010)	0.269164112
RPN1	Diagnoses - main ICD10: A41 Other septicaemia	A41	1.000 (0.999, 1.000)	0.497631073

RPN1	Diagnoses - main ICD10: C18 Malignant neoplasm of colon	C18	1.001 (1.000, 1.002)	0.206809008
RPN1	Diagnoses - main ICD10: C34 Malignant neoplasm of bronchus and lung	C34	1.000 (0.999, 1.001)	0.763944888
RPN1	Diagnoses - main ICD10: C43 Malignant melanoma of skin	C43	1.000 (1.000, 1.001)	0.430808573
RPN1	Diagnoses - main ICD10: C67 Malignant neoplasm of bladder	C67	1.001 (1.000, 1.001)	0.100791967
RPN1	Diagnoses - main ICD10: C78 Secondary malignant neoplasm of respiratory and digestive organs	C78	1.000 (1.000, 1.001)	0.197866529
RPN1	Diagnoses - main ICD10: C79 Secondary malignant neoplasm of other sites	C79	1.000 (1.000, 1.001)	0.612842783
RPN1	Diagnoses - main ICD10: D05 Carcinoma in situ of breast	D05	1.000 (1.000, 1.001)	0.365858295
RPN1	Diagnoses - main ICD10: D17 Benign lipomatous neoplasm	D17	1.000 (0.999, 1.001)	0.987401327
RPN1	Diagnoses - main ICD10: D22 Melanocytic naevi	D22	1.000 (0.999, 1.001)	0.59050496
RPN1	Diagnoses - main ICD10: D23 Other benign neoplasms of skin	D23	1.000 (0.999, 1.001)	0.706545665
RPN1	Diagnoses - main ICD10: D50 Iron deficiency anaemia	D50	1.000 (0.999, 1.001)	0.638788834
RPN1	Diagnoses - main ICD10: D64 Other anaemias	D64	1.000 (0.999, 1.001)	0.915698297
RPN1	Diagnoses - main ICD10: G43 Migraine	G43	1.000 (0.999, 1.000)	0.313299573
RPN1	Diagnoses - main ICD10: G45 Transient cerebral ischaemic attacks and related syndromes	G45	1.000 (0.999, 1.001)	0.844373268
RPN1	Diagnoses - main ICD10: G57 Mononeuropathies of lower limb	G57	1.000 (0.999, 1.001)	0.860301614
RPN1	Diagnoses - main ICD10: H00 Hordeolum and chalazion	H00	1.000 (0.999, 1.001)	0.944563327
RPN1	Diagnoses - main ICD10: H02 Other disorders of eyelid	H02	1.000 (0.998, 1.001)	0.606125345
RPN1	Diagnoses - main ICD10: H04 Disorders of lachrymal system	H04	1.000 (0.999, 1.000)	0.44315996
RPN1	Diagnoses - main ICD10: H33 Retinal detachments and breaks	H33	1.000 (0.999, 1.001)	0.461340228
RPN1	Diagnoses - main ICD10: H35 Other retinal disorders	H35	1.000 (0.999, 1.001)	0.933950762
RPN1	Diagnoses - main ICD10: H40 Glaucoma	H40	1.000 (0.999, 1.000)	0.257005242
RPN1	Diagnoses - main ICD10: I26 Pulmonary embolism	I26	1.001 (1.000, 1.002)	0.013043091
RPN1	Diagnoses - main ICD10: I47 Paroxysmal tachycardia	I47	1.000 (0.999, 1.001)	0.663614222
RPN1	Diagnoses - main ICD10: I50 Heart failure	I50	1.000 (0.999, 1.000)	0.193892151
RPN1	Diagnoses - main ICD10: I63 Cerebral infarction	I63	1.000 (0.999, 1.001)	0.69417548
RPN1	Diagnoses - main ICD10: J18 Pneumonia, organism unspecified	J18	1.000 (0.998, 1.001)	0.54842792
RPN1	Diagnoses - main ICD10: J32 Chronic sinusitis	J32	1.000 (1.000, 1.001)	0.478896167
RPN1	Diagnoses - main ICD10: J38 Diseases of vocal cords and larynx, not elsewhere classified	J38	1.000 (1.000, 1.001)	0.504769417
RPN1	Diagnoses - main ICD10: J45 Asthma	J45	1.000 (0.999, 1.001)	0.792252113
RPN1	Diagnoses - main ICD10: K01 Embedded and impacted teeth	K01	1.000 (0.999, 1.001)	0.803389276
RPN1	Diagnoses - main ICD10: K02 Dental caries	K02	1.000 (0.999, 1.001)	0.489831878
RPN1	Diagnoses - main ICD10: K04 Diseases of pulp and periapical tissues	K04	1.000 (0.999, 1.001)	0.804492715
RPN1	Diagnoses - main ICD10: K08 Other disorders of teeth and supporting structures	K08	1.000 (1.000, 1.001)	0.420302809
RPN1	Diagnoses - main ICD10: K13 Other diseases of lip and oral mucosa	K13	1.000 (0.999, 1.001)	0.896966207
RPN1	Diagnoses - main ICD10: K25 Gastric ulcer	K25	1.000 (0.999, 1.001)	0.884343661
RPN1	Diagnoses - main ICD10: K26 Duodenal ulcer	K26	1.000 (1.000, 1.001)	0.493728334
RPN1	Diagnoses - main ICD10: K31 Other diseases of stomach and duodenum	K31	1.000 (0.999, 1.001)	0.517367838

RPN1	Diagnoses - main ICD10: K42 Umbilical hernia	K42	1.000 (0.999, 1.001)	0.458278803
RPN1	Diagnoses - main ICD10: K56 Paralytic ileus and intestinal obstruction without hernia	K56	1.001 (1.000, 1.002)	0.094437228
RPN1	Diagnoses - main ICD10: K58 Irritable bowel syndrome	K58	1.000 (0.999, 1.001)	0.732259774
RPN1	Diagnoses - main ICD10: K59 Other functional intestinal disorders	K59	1.000 (0.999, 1.001)	0.771821808
RPN1	Diagnoses - main ICD10: K61 Abscess of anal and rectal regions	K61	1.000 (0.999, 1.000)	0.315552249
RPN1	Diagnoses - main ICD10: K63 Other diseases of intestine	K63	1.000 (0.998, 1.002)	0.981364488
RPN1	Diagnoses - main ICD10: K81 Cholecystitis	K81	1.000 (1.000, 1.001)	0.265126684
RPN1	Diagnoses - main ICD10: K92 Other diseases of digestive system	K92	1.000 (0.998, 1.001)	0.783069871
RPN1	Diagnoses - main ICD10: L02 Cutaneous abscess, furuncle and carbuncle	L02	1.000 (0.999, 1.001)	0.792726598
RPN1	Diagnoses - main ICD10: L57 Skin changes due to chronic exposure to nonionising radiation	L57	1.000 (0.999, 1.001)	0.668459325
RPN1	Diagnoses - main ICD10: L72 Follicular cysts of skin and subcutaneous tissue	L72	0.999 (0.998, 1.001)	0.274749465
RPN1	Diagnoses - main ICD10: L90 Atrophic disorders of skin	L90	1.000 (0.999, 1.001)	0.911862508
RPN1	Diagnoses - main ICD10: L98 Other disorders of skin and subcutaneous tissue, not elsewhere classified	L98	0.999 (0.998, 1.000)	0.095504666
RPN1	Diagnoses - main ICD10: M06 Other rheumatoid arthritis	M06	1.000 (0.999, 1.000)	0.388423588
RPN1	Diagnoses - main ICD10: M13 Other arthritis	M13	1.000 (0.999, 1.000)	0.602724761
RPN1	Diagnoses - main ICD10: M15 Polyarthrosis	M15	0.999 (0.999, 1.000)	0.092565622
RPN1	Diagnoses - main ICD10: M19 Other arthrosis	M19	1.001 (0.999, 1.002)	0.388118373
RPN1	Diagnoses - main ICD10: M47 Spondylosis	M47	1.000 (0.999, 1.001)	0.863454035
RPN1	Diagnoses - main ICD10: M48 Other spondylopathies	M48	0.999 (0.999, 1.000)	0.175904202
RPN1	Diagnoses - main ICD10: M51 Other intervertebral disk disorders	M51	1.000 (0.998, 1.001)	0.559649388
RPN1	Diagnoses - main ICD10: M65 Synovitis and tenosynovitis	M65	1.001 (1.000, 1.002)	0.221780049
RPN1	Diagnoses - main ICD10: M75 Shoulder lesions	M75	1.001 (0.999, 1.002)	0.458466963
RPN1	Diagnoses - main ICD10: M79 Other soft tissue disorders, not elsewhere classified	M79	1.000 (0.998, 1.001)	0.793771045
RPN1	Diagnoses - main ICD10: M84 Disorders of continuity of bone	M84	1.000 (0.999, 1.001)	0.786481375
RPN1	Diagnoses - main ICD10: N13 Obstructive and reflux uropathy	N13	1.000 (1.000, 1.001)	0.374875917
RPN1	Diagnoses - main ICD10: N30 Cystitis	N30	1.000 (1.000, 1.001)	0.419973493
RPN1	Diagnoses - main ICD10: N31 Neuromuscular dysfunction of bladder, not elsewhere classified	N31	1.000 (0.999, 1.000)	0.652645758
RPN1	Diagnoses - main ICD10: N35 Urethral stricture	N35	1.000 (1.000, 1.001)	0.352406477
RPN1	Diagnoses - main ICD10: N39 Other disorders of urinary system	N39	0.999 (0.997, 1.001)	0.566333931
RPN1	Diagnoses - main ICD10: N48 Other disorders of penis	N48	1.001 (1.000, 1.001)	0.110806291
RPN1	Diagnoses - main ICD10: N50 Other disorders of male genital organs	N50	1.000 (0.999, 1.001)	0.554245833
RPN1	Diagnoses - main ICD10: N60 Benign mammary dysplasia	N60	1.000 (0.999, 1.000)	0.201819399
RPN1	Diagnoses - main ICD10: N80 Endometriosis	N80	1.000 (0.999, 1.001)	0.549916554
RPN1	Diagnoses - main ICD10: N83 Noninflammatory disorders of ovary, Fallopian tube and broad ligament	N83	1.001 (1.000, 1.002)	0.085240533
RPN1	Diagnoses - main ICD10: N84 Polyp of female genital tract	N84	1.000 (0.998, 1.001)	0.641255086
RPN1	Diagnoses - main ICD10: N85 Other noninflammatory disorders of uterus, except cervix	N85	1.000 (1.000, 1.001)	0.528083181
RPN1	Diagnoses - main ICD10: N87 Dysplasia of cervix uteri	N87	1.000 (0.999, 1.000)	0.200733585

RPN1	Diagnoses - main ICD10: N90 Other noninflammatory disorders of vulva and perineum	N90	1.000 (0.999, 1.001)	0.868266662
RPN1	Diagnoses - main ICD10: N93 Other abnormal uterine and vaginal bleeding	N93	1.000 (0.999, 1.001)	0.491849396
RPN1	Diagnoses - main ICD10: N94 Pain and other conditions associated with female genital organs and menst	N94	1.000 (0.999, 1.001)	0.737809388
RPN1	Diagnoses - main ICD10: N95 Menopausal and other perimenopausal disorders	N95	1.001 (0.999, 1.002)	0.253402106
RPN1	Diagnoses - main ICD10: O02 Other abnormal products of conception	O02	0.999 (0.999, 1.000)	0.080089104
RPN1	Diagnoses - main ICD10: O03 Spontaneous abortion	O03	1.000 (0.999, 1.000)	0.267840901
RPN1	Diagnoses - main ICD10: O26 Maternal care for other conditions predominantly related to pregnancy	O26	1.000 (0.999, 1.001)	0.915440093
RPN1	Diagnoses - main ICD10: O36 Maternal care for other known or suspected foetal problems	O36	1.000 (0.999, 1.000)	0.246461513
RPN1	Diagnoses - main ICD10: O63 Long labour	O63	0.999 (0.999, 1.000)	0.007137706
RPN1	Diagnoses - main ICD10: O68 Labour and delivery complicated by foetal stress [distress]	O68	1.000 (0.999, 1.001)	0.935034348
RPN1	Diagnoses - main ICD10: O70 Perineal laceration during delivery	O70	0.999 (0.998, 1.000)	0.178185839
RPN1	Diagnoses - main ICD10: O80 Single spontaneous delivery	O80	1.000 (0.999, 1.000)	0.45232254
RPN1	Diagnoses - main ICD10: R00 Abnormalities of heart beat	R00	0.999 (0.998, 1.000)	0.073738461
RPN1	Diagnoses - main ICD10: R06 Abnormalities of breathing	R06	0.999 (0.998, 1.001)	0.416109237
RPN1	Diagnoses - main ICD10: R19 Other symptoms and signs involving the digestive system and abdomen	R19	1.000 (0.998, 1.001)	0.667466467
RPN1	Diagnoses - main ICD10: R22 Localised swelling, mass and lump of skin and subcutaneous tissue	R22	1.000 (0.999, 1.001)	0.69963818
RPN1	Diagnoses - main ICD10: R39 Other symptoms and signs involving the urinary system	R39	1.000 (0.999, 1.001)	0.759935773
RPN1	Diagnoses - main ICD10: R50 Fever of unknown origin	R50	1.000 (1.000, 1.001)	0.4711257
RPN1	Diagnoses - main ICD10: R56 Convulsions, not elsewhere classified	R56	1.000 (1.000, 1.001)	0.647706386
RPN1	Diagnoses - main ICD10: R59 Enlarged lymph nodes	R59	1.000 (0.999, 1.001)	0.929686915
RPN1	Diagnoses - main ICD10: R63 Symptoms and signs concerning food and fluid intake	R63	1.000 (0.999, 1.001)	0.784339551
RPN1	Diagnoses - main ICD10: R79 Other abnormal findings of blood chemistry	R79	1.000 (0.999, 1.001)	0.876859165
RPN1	Diagnoses - main ICD10: R87 Abnormal findings in specimens from female genital organs	R87	1.000 (0.999, 1.001)	0.978701643
RPN1	Diagnoses - main ICD10: R93 Abnormal findings on diagnostic imaging of other body structures	R93	1.000 (0.999, 1.001)	0.948547067
RPN1	Diagnoses - main ICD10: S01 Open wound of head	S01	1.000 (1.000, 1.001)	0.335632556
RPN1	Diagnoses - main ICD10: S02 Fracture of skull and facial bones	S02	1.000 (0.999, 1.001)	0.765766179
RPN1	Diagnoses - main ICD10: S42 Fracture of shoulder and upper arm	S42	1.000 (0.999, 1.000)	0.417332391
RPN1	Diagnoses - main ICD10: S61 Open wound of wrist and hand	S61	1.000 (0.999, 1.001)	0.628252041
RPN1	Diagnoses - main ICD10: S62 Fracture at wrist and hand level	S62	1.000 (0.999, 1.000)	0.280436634
RPN1	Diagnoses - main ICD10: S72 Fracture of femur	S72	1.000 (0.999, 1.001)	0.742490408
RPN1	Diagnoses - main ICD10: S82 Fracture of lower leg, including ankle	S82	0.999 (0.998, 1.000)	0.091951901
RPN1	Diagnoses - main ICD10: T39 Poisoning by nonopioid analgesics, antipyretics and antirheumatics	T39	1.000 (0.999, 1.000)	0.253008795
RPN1	Diagnoses - main ICD10: T81 Complications of procedures, not elsewhere classified	T81	1.000 (0.998, 1.001)	0.82117519
RPN1	Diagnoses - main ICD10: T82 Complications of cardiac and vascular prosthetic devices, implants and gra	T82	1.000 (1.000, 1.001)	0.467505704
RPN1	Diagnoses - main ICD10: T85 Complications of other internal prosthetic devices, implants and grafts	T85	1.000 (0.999, 1.001)	0.769263064
RPN1	Diagnoses - main ICD10: Z01 Other special examinations and investigations of persons without complair	Z01	1.000 (0.999, 1.001)	0.613369563
RPN1	Diagnoses - main ICD10: Z03 Medical observation and evaluation for suspected diseases and conditions	Z03	0.999 (0.998, 1.001)	0.458943014

RPN1	Diagnoses - main ICD10: Z08 Follow-up examination after treatment for malignant neoplasm	Z08	1.001 (1.000, 1.002)	0.050330893
RPN1	Diagnoses - main ICD10: Z12 Special screening examination for neoplasms	Z12	1.000 (0.998, 1.001)	0.594855418
RPN1	Diagnoses - main ICD10: Z13 Special screening examination for other diseases and disorders	Z13	1.000 (0.999, 1.001)	0.757356653
RPN1	Diagnoses - main ICD10: Z30 Contraceptive management	Z30	1.000 (0.999, 1.002)	0.738619262
RPN1	Diagnoses - main ICD10: Z42 Follow-up care involving plastic surgery	Z42	1.000 (0.999, 1.001)	0.56805317
RPN1	Diagnoses - main ICD10: Z43 Attention to artificial openings	Z43	1.000 (1.000, 1.001)	0.330727518
RPN1	Diagnoses - main ICD10: Z45 Adjustment and management of implanted device	Z45	1.000 (0.999, 1.001)	0.606744556
RPN1	Diagnoses - main ICD10: Z46 Fitting and adjustment of other devices	Z46	1.000 (0.999, 1.002)	0.392010465
RPN1	Diagnoses - main ICD10: Z53 Persons encountering health services for specifie procedures, not carried o	Z53	1.000 (0.999, 1.001)	0.842808577

Table S12 Phenome-wide Mendelian Randomization analysis to reveal potential on-target side effects of RSPO3

**protein**	**disease**	**ICD-10 code**	**OR (95%CI)**	**P value**
RSPO3	Underlying (primary) cause of death: ICD10: E85.4 Organ-limited amyloidosis	E85.4	1.001 (0.999, 1.003)	0.491191395
RSPO3	Underlying (primary) cause of death: ICD10: J84.1 Other interstitial pulmonary diseases with fibrosis	J84.1	0.999 (0.992, 1.007)	0.881544896
RSPO3	Diagnoses - main ICD10: B37 Candidiasis	B37	1.000 (1.000, 1.000)	0.72864158
RSPO3	Diagnoses - main ICD10: C44 Other malignant neoplasms of skin	C44	1.000 (0.999, 1.002)	0.430533008
RSPO3	Diagnoses - main ICD10: C50 Malignant neoplasm of breast	C50	0.998 (0.997, 1.000)	0.012417852
RSPO3	Diagnoses - main ICD10: C61 Malignant neoplasm of prostate	C61	1.000 (0.999, 1.001)	0.978721642
RSPO3	Diagnoses - main ICD10: D12 Benign neoplasm of colon rectum anus and anal canal	D12	1.000 (0.998, 1.001)	0.398211199
RSPO3	Diagnoses - main ICD10: D25 Leiomyoma of uterus	D25	1.001 (1.000, 1.002)	0.271515528
RSPO3	Diagnoses - main ICD10: E03 Other hypothyroidism	E03	1.000 (1.000, 1.000)	0.482283286
RSPO3	Diagnoses - main ICD10: E04 Other non-toxic goitre	E04	1.000 (0.999, 1.000)	0.494028242
RSPO3	Diagnoses - main ICD10: F31 Bipolar affective disorder	F31	1.000 (1.000, 1.000)	0.44696516
RSPO3	Diagnoses - main ICD10: F43 Reaction to severe stress and adjustment disorders	F43	1.000 (1.000, 1.000)	0.298748441
RSPO3	Diagnoses - main ICD10: G47 Sleep disorders	G47	1.001 (1.000, 1.001)	0.094087944
RSPO3	Diagnoses - main ICD10: G56 Mononeuropathies of upper limb	G56	1.001 (0.999, 1.002)	0.286986383
RSPO3	Diagnoses - main ICD10: H25 Senile cataract	H25	1.001 (1.000, 1.002)	0.041376983
RSPO3	Diagnoses - main ICD10: H26 Other cataract	H26	1.001 (1.000, 1.002)	0.218426584
RSPO3	Diagnoses - main ICD10: I10 Essential (primary) hypertension	I10	1.000 (0.999, 1.000)	0.23989211
RSPO3	Diagnoses - main ICD10: I20 Angina pectoris	I20	1.000 (0.998, 1.001)	0.517191323
RSPO3	Diagnoses - main ICD10: I21 Acute myocardial infarction	I21	1.001 (1.000, 1.002)	0.159246338
RSPO3	Diagnoses - main ICD10: I25 Chronic ischaemic heart disease	I25	1.002 (1.000, 1.003)	0.042284297
RSPO3	Diagnoses - main ICD10: I30 Acute pericarditis	I30	1.000 (1.000, 1.000)	0.529960954
RSPO3	Diagnoses - main ICD10: I48 Atrial fibrillation and flutter	I48	1.001 (1.000, 1.002)	0.14987265
RSPO3	Diagnoses - main ICD10: I80 Phlebitis and thrombophlebitis	I80	1.000 (0.999, 1.000)	0.17596172
RSPO3	Diagnoses - main ICD10: I84 Haemorrhoids	I84	1.001 (1.000, 1.003)	0.126509047
RSPO3	Diagnoses - main ICD10: J22 Unspecified acute lower respiratory infection	J22	1.000 (0.999, 1.000)	0.32975309
RSPO3	Diagnoses - main ICD10: J33 Nasal polyp	J33	0.999 (0.999, 1.000)	0.039700606
RSPO3	Diagnoses - main ICD10: J34 Other disorders of nose and nasal sinuses	J34	1.000 (0.999, 1.000)	0.328479314
RSPO3	Diagnoses - main ICD10: J44 Other chronic obstructive pulmonary disease	J44	1.000 (1.000, 1.001)	0.92922457
RSPO3	Diagnoses - main ICD10: K20 Oesophagitis	K20	1.001 (1.000, 1.002)	0.113289361
RSPO3	Diagnoses - main ICD10: K21 Gastro-oesophageal reflux disease	K21	1.001 (0.999, 1.002)	0.226767618
RSPO3	Diagnoses - main ICD10: K22 Other diseases of oesophagus	K22	1.001 (1.000, 1.002)	0.289862355
RSPO3	Diagnoses - main ICD10: K29 Gastritis and duodenitis	K29	1.000 (0.998, 1.001)	0.982938399
RSPO3	Diagnoses - main ICD10: K35 Acute appendicitis	K35	1.000 (0.999, 1.001)	0.997791162
RSPO3	Diagnoses - main ICD10: K40 Inguinal hernia	K40	0.999 (0.997, 1.000)	0.131287939
RSPO3	Diagnoses - main ICD10: K43 Ventral hernia	K43	1.000 (1.000, 1.001)	0.42057518

RSPO3	Diagnoses - main ICD10: K44 Diaphragmatic hernia	K44	1.001 (1.000, 1.002)	0.139416346
RSPO3	Diagnoses - main ICD10: K50 Crohn's disease [regional enteritis]	K50	1.000 (1.000, 1.001)	0.269648397
RSPO3	Diagnoses - main ICD10: K51 Ulcerative colitis	K51	1.000 (0.999, 1.000)	0.44602398
RSPO3	Diagnoses - main ICD10: K52 Other non-infective gastro-enteritis and colitis	K52	1.001 (0.999, 1.002)	0.399301122
RSPO3	Diagnoses - main ICD10: K57 Diverticular disease of intestine	K57	1.001 (0.999, 1.002)	0.405834937
RSPO3	Diagnoses - main ICD10: K60 Fissure and fistula of anal and rectal regions	K60	1.000 (1.000, 1.001)	0.302532549
RSPO3	Diagnoses - main ICD10: K62 Other diseases of anus and rectum	K62	0.999 (0.998, 1.001)	0.512710791
RSPO3	Diagnoses - main ICD10: K76 Other diseases of liver	K76	1.000 (1.000, 1.000)	0.433211766
RSPO3	Diagnoses - main ICD10: K80 Cholelithiasis	K80	1.000 (0.999, 1.002)	0.754621946
RSPO3	Diagnoses - main ICD10: L03 Cellulitis	L03	1.000 (0.999, 1.001)	0.856938069
RSPO3	Diagnoses - main ICD10: M10 Gout	M10	1.000 (1.000, 1.000)	0.83968203
RSPO3	Diagnoses - main ICD10: M16 Coxarthrosis [arthrosis of hip]	M16	1.000 (0.998, 1.001)	0.518800622
RSPO3	Diagnoses - main ICD10: M17 Gonarthrosis [arthrosis of knee]	M17	1.001 (1.000, 1.002)	0.207617096
RSPO3	Diagnoses - main ICD10: M20 Acquired deformities of fingers and toes	M20	1.001 (1.000, 1.002)	0.153885276
RSPO3	Diagnoses - main ICD10: M21 Other acquired deformities of limbs	M21	1.000 (1.000, 1.000)	0.687441424
RSPO3	Diagnoses - main ICD10: M23 Internal derangement of knee	M23	1.000 (0.998, 1.001)	0.500351067
RSPO3	Diagnoses - main ICD10: M24 Other specific joint derangements	M24	1.000 (0.999, 1.000)	0.900470774
RSPO3	Diagnoses - main ICD10: M25 Other joint disorders not elsewhere classified	M25	0.999 (0.998, 1.001)	0.380137078
RSPO3	Diagnoses - main ICD10: M54 Dorsalgia	M54	1.000 (0.999, 1.001)	0.715620259
RSPO3	Diagnoses - main ICD10: M67 Other disorders of synovium and tendon	M67	1.000 (0.999, 1.001)	0.878034624
RSPO3	Diagnoses - main ICD10: M70 Soft tissue disorders related to use overuse and pressure	M70	1.000 (0.999, 1.000)	0.132565962
RSPO3	Diagnoses - main ICD10: M72 Fibroblastic disorders	M72	1.000 (1.000, 1.001)	0.476276143
RSPO3	Diagnoses - main ICD10: N19 Unspecified renal failure	N19	1.000 (1.000, 1.000)	0.643082783
RSPO3	Diagnoses - main ICD10: N20 Calculus of kidney and ureter	N20	0.999 (0.999, 1.000)	0.117802922
RSPO3	Diagnoses - main ICD10: N32 Other disorders of bladder	N32	1.000 (0.999, 1.001)	0.585644489
RSPO3	Diagnoses - main ICD10: N40 Hyperplasia of prostate	N40	1.000 (1.000, 1.001)	0.350215681
RSPO3	Diagnoses - main ICD10: N81 Female genital prolapse	N81	1.001 (1.000, 1.002)	0.105991694
RSPO3	Diagnoses - main ICD10: N92 Excessive frequent and irregular menstruation	N92	1.002 (1.000, 1.003)	0.02591344
RSPO3	Diagnoses - main ICD10: O75 Other complications of labour and delivery not elsewhere classified	O75	1.000 (1.000, 1.000)	0.080033724
RSPO3	Diagnoses - main ICD10: R04 Haemorrhage from respiratory passages	R04	1.000 (1.000, 1.001)	0.373528095
RSPO3	Diagnoses - main ICD10: R07 Pain in throat and chest	R07	0.999 (0.997, 1.001)	0.326780508
RSPO3	Diagnoses - main ICD10: R10 Abdominal and pelvic pain	R10	1.000 (0.998, 1.002)	0.824590575
RSPO3	Diagnoses - main ICD10: R11 Nausea and vomiting	R11	1.000 (1.000, 1.001)	0.647393826
RSPO3	Diagnoses - main ICD10: R14 Flatulence and related conditions	R14	1.000 (1.000, 1.000)	0.264258145
RSPO3	Diagnoses - main ICD10: R31 Unspecified haematuria	R31	0.999 (0.998, 1.001)	0.43141957
RSPO3	Diagnoses - main ICD10: R35 Polyuria	R35	1.000 (0.999, 1.001)	0.852411451
RSPO3	Diagnoses - main ICD10: R55 Syncope and collapse	R55	1.000 (0.999, 1.001)	0.587465682

RSPO3	Diagnoses - main ICD10: R69 Unknown and unspecified causes of morbidity	R69	1.001 (1.000, 1.003)	0.041788594
RSPO3	Diagnoses - main ICD10: S09 Other and unspecified injuries of head	S09	1.000 (0.999, 1.000)	0.546895748
RSPO3	Diagnoses - main ICD10: S52 Fracture of forearm	S52	0.997 (0.996, 0.998)	7.56696E-09
RSPO3	Diagnoses - main ICD10: S66 Injury of muscle and tendon at wrist and hand level	S66	1.000 (1.000, 1.000)	0.934974039
RSPO3	Diagnoses - main ICD10: S76 Injury of muscle and tendon at hip and thigh level	S76	1.000 (1.000, 1.000)	0.79917123
RSPO3	Diagnoses - main ICD10: T84 Complications of internal orthopaedic prosthetic devices implants and g	T84	0.999 (0.999, 1.000)	0.088129785
RSPO3	Diagnoses - main ICD10: Z09 Follow-up examination after treatment for conditions other than maligna	Z09	1.000 (0.998, 1.001)	0.572872315
RSPO3	Diagnoses - main ICD10: Z47 Other orthopaedic follow-up care	Z47	0.998 (0.998, 0.999)	8.30476E-06
RSPO3	Diagnoses - main ICD10: Z80 Family history of malignant neoplasm	Z80	1.000 (1.000, 1.000)	0.337780906
RSPO3	Diagnoses - secondary ICD10: Z50.1 Other physical therapy	Z50.1	1.000 (0.999, 1.001)	0.950560773
RSPO3	Diagnoses - main ICD10: Z09.0 Follow-up examination after surgery for other conditions	Z09.0	0.999 (0.999, 1.000)	0.061569871
RSPO3	Diagnoses - secondary ICD10: K21.0 Gastro-oesophageal reflux disease with oesophagitis	K21.0	1.000 (0.999, 1.001)	0.854253674
RSPO3	Type of cancer: ICD10: C44.3 Skin of other and unspecified parts of face	C44.3	1.001 (1.000, 1.002)	0.112232469
RSPO3	Diagnoses - main ICD10: K80.0 Calculus of gallbladder with acute cholecystitis	K80.0	1.000 (1.000, 1.000)	0.974873323
RSPO3	Diagnoses - secondary ICD10: R19.4 Change in bowel habit	R19.4	1.000 (1.000, 1.001)	0.368652597
RSPO3	Diagnoses - secondary ICD10: R10.1 Pain localised to upper abdomen	R10.1	1.000 (1.000, 1.000)	0.986383536
RSPO3	Diagnoses - secondary ICD10: Z03.8 Observation for other suspected diseases and conditions	Z03.8	1.000 (0.999, 1.000)	0.680635223
RSPO3	Diagnoses - secondary ICD10: I50.1 Left ventricular failure	I50.1	1.000 (0.999, 1.000)	0.632492347
RSPO3	Diagnoses - main ICD10: D12.5 Sigmoid colon	D12.5	1.000 (1.000, 1.001)	0.543992933
RSPO3	Diagnoses - main ICD10: Z09.9 Follow-up examination after unspecified treatment for other condition	Z09.9	1.000 (1.000, 1.001)	0.508189868
RSPO3	Diagnoses - main ICD10: N47 Redundant prepuce, phimosis and paraphimosis	N47	1.000 (0.999, 1.000)	0.710387858
RSPO3	Diagnoses - secondary ICD10: E10.9 Without complications	E10.9	1.000 (0.999, 1.000)	0.549903108
RSPO3	Diagnoses - main ICD10: O70.1 Second degree perineal laceration during delivery	O70.1	1.000 (1.000, 1.001)	0.522291773
RSPO3	Diagnoses - secondary ICD10: I20.0 Unstable angina	I20.0	1.000 (0.999, 1.000)	0.353209803
RSPO3	Diagnoses - main ICD10: R07.4 Chest pain, unspecified	R07.4	0.999 (0.997, 1.000)	0.134542004
RSPO3	Diagnoses - main ICD10: N93.9 Abnormal uterine and vaginal bleeding, unspecified	N93.9	1.000 (1.000, 1.001)	0.52484897
RSPO3	Diagnoses - main ICD10: K57.3 Diverticular disease of large intestine without perforation or abscess	K57.3	1.001 (1.000, 1.002)	0.077057429
RSPO3	Diagnoses - main ICD10: M54.59 Low back pain (Site unspecified)	M54.59	1.000 (1.000, 1.000)	0.955497403
RSPO3	Diagnoses - secondary ICD10: Z87.4 Personal history of diseases of the genito-urinary system	Z87.4	1.000 (0.999, 1.000)	0.288645707
RSPO3	Diagnoses - secondary ICD10: Z92.2 Personal history of long-term (current) use of other medicaments	Z92.2	0.999 (0.998, 1.000)	0.196184046
RSPO3	Diagnoses - main ICD10: K80.2 Calculus of gallbladder without cholecystitis	K80.2	1.000 (0.999, 1.001)	0.624277008
RSPO3	Diagnoses - secondary ICD10: M13.9 Arthritis, unspecified	M13.9	1.000 (0.999, 1.000)	0.733481501
RSPO3	Diagnoses - main ICD10: R79.8 Other specified abnormal findings of blood chemistry	R79.8	1.000 (0.999, 1.000)	0.687246964
RSPO3	Diagnoses - secondary ICD10: Z95.1 Presence of aortocoronary bypass graft	Z95.1	1.001 (1.000, 1.001)	0.10378774
RSPO3	Diagnoses - secondary ICD10: W01.0 Home	W01.0	1.000 (1.000, 1.000)	0.734987396
RSPO3	Diagnoses - secondary ICD10: Z85.8 Personal history of malignant neoplasms of other organs and syst	Z85.8	0.999 (0.999, 1.000)	0.093989764
RSPO3	Diagnoses - main ICD10: I84.9 Unspecified haemorrhoids without complication	I84.9	1.000 (0.999, 1.001)	0.768629632

RSPO3	Diagnoses - main ICD10: K60.2 Anal fissure, unspecified	K60.2	1.000 (1.000, 1.001)	0.055889617
RSPO3	Diagnoses - main ICD10: N81.1 Cystocele	N81.1	1.000 (0.999, 1.001)	0.79679528
RSPO3	Diagnoses - secondary ICD10: F41.9 Anxiety disorder, unspecified	F41.9	1.000 (0.999, 1.000)	0.610082587
RSPO3	Diagnoses - main ICD10: K40.2 Bilateral inguinal hernia, without obstruction or gangrene	K40.2	1.000 (0.999, 1.000)	0.04895327
RSPO3	Diagnoses - main ICD10: L90.5 Scar conditions and fibrosis of skin	L90.5	1.000 (1.000, 1.001)	0.246031433
RSPO3	Diagnoses - main ICD10: R04.0 Epistaxis	R04.0	1.000 (1.000, 1.001)	0.275800349
RSPO3	Diagnoses - secondary ICD10: Z51.1 Chemotherapy session for neoplasm	Z51.1	1.000 (0.999, 1.001)	0.72233445
RSPO3	Diagnoses - main ICD10: R32 Unspecified urinary incontinence	R32	1.000 (1.000, 1.000)	0.931682106
RSPO3	Diagnoses - main ICD10: N92.0 Excessive and frequent menstruation with regular cycle	N92.0	1.001 (1.000, 1.002)	0.015375948
RSPO3	Diagnoses - main ICD10: K62.5 Haemorrhage of anus and rectum	K62.5	0.999 (0.998, 1.001)	0.267050862
RSPO3	Diagnoses - secondary ICD10: Z96.1 Presence of intraocular lens	Z96.1	1.000 (1.000, 1.001)	0.241364037
RSPO3	Diagnoses - secondary ICD10: R00.1 Bradycardia, unspecified	R00.1	1.000 (1.000, 1.001)	0.664726859
RSPO3	Diagnoses - secondary ICD10: K66.0 Peritoneal adhesions	K66.0	1.000 (0.999, 1.000)	0.217214717
RSPO3	Diagnoses - main ICD10: T84.8 Other complications of internal orthopaedic prosthetic devices, implan	T84.8	1.000 (1.000, 1.000)	0.893915969
RSPO3	Diagnoses - secondary ICD10: Z85.4 Personal history of malignant neoplasm of genital organs	Z85.4	1.000 (0.999, 1.000)	0.279245655
RSPO3	Diagnoses - main ICD10: I47.1 Supraventricular tachycardia	I47.1	1.000 (0.999, 1.000)	0.738751783
RSPO3	Diagnoses - secondary ICD10: F10.0 Acute intoxication	F10.0	1.000 (0.999, 1.000)	0.038444483
RSPO3	Diagnoses - main ICD10: K08.3 Retained dental root	K08.3	1.000 (1.000, 1.001)	0.089850826
RSPO3	Diagnoses - secondary ICD10: M06.99 Rheumatoid arthritis, unspecified (Site unspecified)	M06.99	1.000 (1.000, 1.001)	0.145353947
RSPO3	Diagnoses - main ICD10: L03.1 Cellulitis of other parts of limb	L03.1	1.000 (1.000, 1.001)	0.216154867
RSPO3	Diagnoses - secondary ICD10: R53 Malaise and fatigue	R53	1.000 (0.999, 1.000)	0.522276296
RSPO3	Diagnoses - secondary ICD10: W01.9 Unspecified place	W01.9	0.999 (0.999, 1.000)	0.003282407
RSPO3	Diagnoses - main ICD10: C50.4 Upper-outer quadrant of breast	C50.4	1.000 (0.999, 1.000)	0.052192114
RSPO3	Diagnoses - main ICD10: T39.1 4-Aminophenol derivatives	T39.1	1.000 (1.000, 1.000)	0.739792488
RSPO3	Diagnoses - secondary ICD10: K29.5 Chronic gastritis, unspecified	K29.5	1.000 (1.000, 1.000)	0.99452454
RSPO3	Diagnoses - secondary ICD10: G55.1 Nerve root and plexus compressions in intervertebral disk disord	G55.1	1.000 (1.000, 1.001)	0.658000571
RSPO3	Diagnoses - secondary ICD10: K25.9 Unspecified as acute or chronic, without haemorrhage or perfora	K25.9	1.000 (1.000, 1.001)	0.146265657
RSPO3	Diagnoses - main ICD10: I80.2 Phlebitis and thrombophlebitis of other deep vessels of lower extremiti	I80.2	1.000 (0.999, 1.000)	0.473200204
RSPO3	Diagnoses - secondary ICD10: I84.6 Residual haemorrhoidal skin tags	I84.6	1.000 (0.999, 1.000)	0.313284139
RSPO3	Diagnoses - main ICD10: N32.0 Bladder-neck obstruction	N32.0	1.000 (1.000, 1.001)	0.372765097
RSPO3	Diagnoses - main ICD10: K29.8 Duodenitis	K29.8	1.000 (1.000, 1.001)	0.666248506
RSPO3	Diagnoses - main ICD10: M67.4 Ganglion	M67.4	1.000 (0.999, 1.000)	0.485354228
RSPO3	Diagnoses - main ICD10: H26.9 Cataract, unspecified	H26.9	1.001 (1.000, 1.002)	0.202733861
RSPO3	Type of cancer: ICD10: C20 Malignant neoplasm of rectum	C20	1.000 (0.999, 1.000)	0.881026703
RSPO3	Diagnoses - secondary ICD10: K30 Dyspepsia	K30	1.000 (1.000, 1.001)	0.509797461
RSPO3	Diagnoses - secondary ICD10: I84.2 Internal haemorrhoids without complication	I84.2	1.000 (1.000, 1.001)	0.140157852
RSPO3	Diagnoses - secondary ICD10: N94.6 Dysmenorrhoea, unspecified	N94.6	1.000 (1.000, 1.001)	0.189554854

RSPO3	Diagnoses - main ICD10: Z08.0 Follow-up examination after surgery for malignant neoplasm	Z08.0	1.000 (1.000, 1.001)	0.186350124
RSPO3	Diagnoses - main ICD10: M79.86 Other specified soft tissue disorders (Lower leg)	M79.86	1.000 (1.000, 1.001)	0.439650775
RSPO3	Diagnoses - secondary ICD10: E78.0 Pure hypercholesterolaemia	E78.0	1.000 (0.998, 1.002)	0.898835529
RSPO3	Diagnoses - secondary ICD10: M10.99 Gout, unspecified (Site unspecified)	M10.99	1.000 (0.999, 1.000)	0.027912874
RSPO3	Diagnoses - main ICD10: M51.2 Other specified intervertebral disk displacement	M51.2	1.000 (1.000, 1.001)	0.439203247
RSPO3	Diagnoses - main ICD10: N84.0 Polyp of corpus uteri	N84.0	1.001 (1.000, 1.002)	0.099976426
RSPO3	Diagnoses - secondary ICD10: M17.1 Other primary gonarthrosis	M17.1	1.000 (1.000, 1.001)	0.32518667
RSPO3	Diagnoses - main ICD10: M25.5 Pain in joint	M25.5	1.000 (0.999, 1.000)	0.138234879
RSPO3	Diagnoses - main ICD10: K29.6 Other gastritis	K29.6	1.000 (1.000, 1.001)	0.41903745
RSPO3	Type of cancer: ICD10: C64 Malignant neoplasm of kidney, except renal pelvis	C64	1.000 (0.999, 1.000)	0.316322318
RSPO3	Diagnoses - main ICD10: G43.9 Migraine, unspecified	G43.9	1.000 (1.000, 1.000)	0.93866551
RSPO3	Diagnoses - secondary ICD10: Z80.0 Family history of malignant neoplasm of digestive organs	Z80.0	1.001 (1.000, 1.002)	0.064668833
RSPO3	Diagnoses - main ICD10: G45.9 Transient cerebral ischaemic attack, unspecified	G45.9	1.000 (1.000, 1.001)	0.322748317
RSPO3	Diagnoses - main ICD10: O02.1 Missed abortion	O02.1	1.000 (1.000, 1.001)	0.095397486
RSPO3	Diagnoses - main ICD10: K62.8 Other specified diseases of anus and rectum	K62.8	1.000 (0.999, 1.000)	0.179913476
RSPO3	Diagnoses - main ICD10: M72.0 Palmar fascial fibromatosis [Dupuytren]	M72.0	1.000 (0.999, 1.000)	0.437782265
RSPO3	Diagnoses - secondary ICD10: I34.0 Mitral (valve) insufficiency	I34.0	1.000 (1.000, 1.000)	0.892553725
RSPO3	Diagnoses - main ICD10: O04.9 Complete or unspecified, without complication	O04.9	1.000 (1.000, 1.001)	0.154553348
RSPO3	Diagnoses - main ICD10: K22.1 Ulcer of oesophagus	K22.1	1.000 (0.999, 1.001)	0.624839531
RSPO3	Diagnoses - secondary ICD10: Z86.7 Personal history of diseases of the circulatory system	Z86.7	1.000 (0.999, 1.001)	0.943164561
RSPO3	Diagnoses - secondary ICD10: Y83.1 Surgical operation with implant of artificial internal device	Y83.1	1.000 (0.999, 1.000)	0.312667885
RSPO3	Diagnoses - secondary ICD10: R63.4 Abnormal weight loss	R63.4	1.000 (1.000, 1.001)	0.227356918
RSPO3	Diagnoses - main ICD10: K21.9 Gastro-oesophageal reflux disease without oesophagitis	K21.9	1.000 (0.999, 1.001)	0.883289137
RSPO3	Diagnoses - main ICD10: N93.8 Other specified abnormal uterine and vaginal bleeding	N93.8	1.000 (1.000, 1.001)	0.219169061
RSPO3	Diagnoses - main ICD10: M75.4 Impingement syndrome of shoulder	M75.4	1.000 (0.999, 1.001)	0.685750689
RSPO3	Diagnoses - main ICD10: I20.9 Angina pectoris, unspecified	I20.9	1.000 (0.999, 1.001)	0.963478184
RSPO3	Diagnoses - secondary ICD10: X59.9 Unspecified place	X59.9	0.999 (0.999, 1.000)	0.03517265
RSPO3	Diagnoses - main ICD10: I84.1 Internal haemorrhoids with other complications	I84.1	1.000 (1.000, 1.001)	0.101883616
RSPO3	Diagnoses - secondary ICD10: K57.9 Diverticular disease of intestine, part unspecified, without perfora	K57.9	1.000 (0.999, 1.000)	0.254064176
RSPO3	Diagnoses - main ICD10: L72.9 Follicular cyst of skin and subcutaneous tissue, unspecified	L72.9	1.000 (1.000, 1.000)	0.802723355
RSPO3	Diagnoses - secondary ICD10: Z92.3 Personal history of irradiation	Z92.3	1.000 (0.999, 1.000)	0.562434634
RSPO3	Diagnoses - main ICD10: Z09.8 Follow-up examination after other treatment for other conditions	Z09.8	1.000 (0.999, 1.000)	0.581710427
RSPO3	Diagnoses - main ICD10: K42.9 Umbilical hernia without obstruction or gangrene	K42.9	1.000 (0.999, 1.001)	0.800534565
RSPO3	Diagnoses - main ICD10: R07.3 Other chest pain	R07.3	1.000 (0.999, 1.001)	0.597964321
RSPO3	Diagnoses - main ICD10: N92.6 Irregular menstruation, unspecified	N92.6	1.000 (0.999, 1.000)	0.656737728
RSPO3	Diagnoses - main ICD10: T84.0 Mechanical complication of internal joint prosthesis	T84.0	1.000 (1.000, 1.000)	0.906256765
RSPO3	Diagnoses - main ICD10: D64.9 Anaemia, unspecified	D64.9	1.001 (1.000, 1.001)	0.170448935

RSPO3	Diagnoses - secondary ICD10: R00.2 Palpitations	R00.2	1.000 (1.000, 1.001)	0.625105316
RSPO3	Diagnoses - secondary ICD10: Z90.4 Acquired absence of other parts of digestive tract	Z90.4	1.000 (0.999, 1.001)	0.653519398
RSPO3	Type of cancer: ICD10: C54.1 Endometrium	C54.1	1.000 (0.999, 1.000)	0.672839294
RSPO3	Diagnoses - main ICD10: K92.2 Gastro-intestinal haemorrhage, unspecified	K92.2	1.000 (1.000, 1.000)	0.979708089
RSPO3	Diagnoses - main ICD10: L72.0 Epidermal cyst	L72.0	1.000 (0.999, 1.001)	0.978798559
RSPO3	Diagnoses - secondary ICD10: M13.99 Arthritis, unspecified (Site unspecified)	M13.99	1.000 (1.000, 1.001)	0.514870543
RSPO3	Diagnoses - secondary ICD10: Z95.8 Presence of other cardiac and vascular implants and grafts	Z95.8	1.000 (0.999, 1.000)	0.073088488
RSPO3	Diagnoses - secondary ICD10: K29.7 Gastritis, unspecified	K29.7	1.001 (1.000, 1.002)	0.137399281
RSPO3	Diagnoses - secondary ICD10: E66.9 Obesity, unspecified	E66.9	1.000 (1.000, 1.001)	0.411820902
RSPO3	Diagnoses - main ICD10: M54.5 Low back pain	M54.5	1.000 (0.999, 1.001)	0.848092094
RSPO3	Diagnoses - main ICD10: J18.9 Pneumonia, unspecified	J18.9	1.000 (0.999, 1.000)	0.740699097
RSPO3	Type of cancer: ICD10: C44.5 Skin of trunk	C44.5	1.000 (0.999, 1.001)	0.754524842
RSPO3	Diagnoses - secondary ICD10: Z95.5 Presence of coronary angioplasty implant and graft	Z95.5	1.000 (0.999, 1.000)	0.449180985
RSPO3	Type of cancer: ICD10: C18.7 Sigmoid colon	C18.7	1.000 (1.000, 1.001)	0.147059036
RSPO3	Diagnoses - main ICD10: R87.6 Abnormal cytological findings	R87.6	1.000 (0.999, 1.000)	0.038319493
RSPO3	Diagnoses - secondary ICD10: Z86.4 Personal history of psychoactive substance abuse	Z86.4	0.999 (0.998, 1.000)	0.135134366
RSPO3	Diagnoses - secondary ICD10: Z51.5 Palliative care	Z51.5	1.000 (0.999, 1.000)	0.247048718
RSPO3	Diagnoses - secondary ICD10: G40.9 Epilepsy, unspecified	G40.9	1.000 (1.000, 1.001)	0.441629189
RSPO3	Diagnoses - secondary ICD10: Z51.0 Radiotherapy session	Z51.0	1.000 (0.999, 1.000)	0.123614971
RSPO3	Diagnoses - secondary ICD10: F10.1 Harmful use	F10.1	0.999 (0.999, 1.000)	0.032014964
RSPO3	Diagnoses - secondary ICD10: I51.7 Cardiomegaly	I51.7	1.000 (1.000, 1.000)	0.890587486
RSPO3	Diagnoses - main ICD10: N23 Unspecified renal colic	N23	1.000 (1.000, 1.001)	0.263857224
RSPO3	Diagnoses - secondary ICD10: E86 Volume depletion	E86	1.000 (1.000, 1.000)	0.940857121
RSPO3	Diagnoses - secondary ICD10: Z87.1 Personal history of diseases of the digestive system	Z87.1	0.999 (0.998, 1.001)	0.371560882
RSPO3	Diagnoses - secondary ICD10: Z85.0 Personal history of malignant neoplasm of digestive organs	Z85.0	1.000 (1.000, 1.001)	0.224877103
RSPO3	Diagnoses - secondary ICD10: R03.0 Elevated blood-pressure reading, without diagnosis of hypertens	R03.0	1.000 (0.999, 1.000)	0.049536661
RSPO3	Diagnoses - secondary ICD10: I25.8 Other forms of chronic ischaemic heart disease	I25.8	1.001 (1.000, 1.002)	0.104278166
RSPO3	Diagnoses - secondary ICD10: Z90.1 Acquired absence of breast(s)	Z90.1	1.000 (1.000, 1.001)	0.44703013
RSPO3	Diagnoses - secondary ICD10: I25.2 Old myocardial infarction	I25.2	1.000 (0.999, 1.001)	0.80189553
RSPO3	Diagnoses - main ICD10: I25.1 Atherosclerotic heart disease	I25.1	1.001 (1.000, 1.003)	0.057259203
RSPO3	Diagnoses - secondary ICD10: Z91.0 Personal history of allergy, other than to drugs and biological sul	Z91.0	1.000 (0.999, 1.000)	0.428417848
RSPO3	Diagnoses - secondary ICD10: C78.7 Secondary malignant neoplasm of liver	C78.7	1.000 (1.000, 1.000)	0.864498163
RSPO3	Diagnoses - secondary ICD10: J44.9 Chronic obstructive pulmonary disease, unspecified	J44.9	0.999 (0.999, 1.000)	0.120869754
RSPO3	Diagnoses - main ICD10: G47.3 Sleep apnoea	G47.3	1.001 (1.000, 1.001)	0.053513368
RSPO3	Diagnoses - secondary ICD10: N95.0 Postmenopausal bleeding	N95.0	1.000 (0.999, 1.001)	0.948488724
RSPO3	Diagnoses - main ICD10: S09.9 Unspecified injury of head	S09.9	1.000 (0.999, 1.000)	0.71570012
RSPO3	Diagnoses - secondary ICD10: Z95.0 Presence of cardiac pacemaker	Z95.0	1.000 (0.999, 1.000)	0.208174403

RSPO3	Diagnoses - secondary ICD10: C77.3 Axillary and upper limb lymph nodes	C77.3	1.000 (0.999, 1.000)	0.108466048
RSPO3	Type of cancer: ICD10: C44.4 Skin of scalp and neck	C44.4	1.000 (1.000, 1.001)	0.629150696
RSPO3	Diagnoses - secondary ICD10: J45.9 Asthma, unspecified	J45.9	0.999 (0.998, 1.001)	0.452545256
RSPO3	Diagnoses - main ICD10: R39.8 Other and unspecified symptoms and signs involving the urinary syste	R39.8	1.000 (1.000, 1.001)	0.426917632
RSPO3	Diagnoses - secondary ICD10: R00.0 Tachycardia, unspecified	R00.0	0.999 (0.999, 1.000)	0.003703127
RSPO3	Diagnoses - main ICD10: R10.3 Pain localised to other parts of lower abdomen	R10.3	1.000 (1.000, 1.001)	0.406385295
RSPO3	Diagnoses - secondary ICD10: E78.5 Hyperlipidaemia, unspecified	E78.5	1.000 (1.000, 1.001)	0.465048554
RSPO3	Diagnoses - secondary ICD10: E14.9 Without complications	E14.9	1.000 (0.999, 1.000)	0.29351894
RSPO3	Diagnoses - main ICD10: T81.0 Haemorrhage and haematoma complicating a procedure, not elsewher	T81.0	1.000 (0.999, 1.000)	0.158995684
RSPO3	Diagnoses - secondary ICD10: J18.1 Lobar pneumonia, unspecified	J18.1	1.000 (1.000, 1.001)	0.58750049
RSPO3	Diagnoses - secondary ICD10: M81.99 Osteoporosis, unspecified (Site unspecified)	M81.99	0.999 (0.998, 1.000)	0.000234415
RSPO3	Diagnoses - secondary ICD10: Y83.8 Other surgical procedures	Y83.8	0.999 (0.998, 1.000)	0.107597632
RSPO3	Diagnoses - secondary ICD10: K63.5 Polyp of colon	K63.5	1.000 (1.000, 1.001)	0.583886572
RSPO3	Diagnoses - main ICD10: K58.9 Irritable bowel syndrome without diarrhoea	K58.9	1.000 (0.999, 1.000)	0.466479178
RSPO3	Diagnoses - secondary ICD10: K59.0 Constipation	K59.0	1.000 (0.999, 1.001)	0.714305235
RSPO3	Diagnoses - secondary ICD10: Z87.3 Personal history of diseases of the musculoskeletal system and co	Z87.3	1.000 (1.000, 1.001)	0.457402143
RSPO3	Type of cancer: ICD10: C56 Malignant neoplasm of ovary	C56	1.000 (0.999, 1.000)	0.542354638
RSPO3	Diagnoses - main ICD10: M51.1 Lumbar and other intervertebral disk disorders with radiculopathy	M51.1	1.000 (0.999, 1.001)	0.988744108
RSPO3	Diagnoses - main ICD10: I26.9 Pulmonary embolism without mention of acute cor pulmonale	I26.9	1.000 (0.999, 1.000)	0.626291532
RSPO3	Diagnoses - main ICD10: N20.0 Calculus of kidney	N20.0	1.000 (0.999, 1.000)	0.268429323
RSPO3	Diagnoses - main ICD10: M17.9 Gonarthrosis, unspecified	M17.9	1.000 (0.999, 1.001)	0.985263696
RSPO3	Diagnoses - main ICD10: K92.0 Haematemesis	K92.0	1.000 (1.000, 1.001)	0.307235259
RSPO3	Diagnoses - main ICD10: N20.1 Calculus of ureter	N20.1	1.000 (1.000, 1.001)	0.709667467
RSPO3	Diagnoses - main ICD10: K13.7 Other and unspecified lesions of oral mucosa	K13.7	1.000 (1.000, 1.001)	0.374399353
RSPO3	Type of cancer: ICD10: C34.1 Upper lobe, bronchus or lung	C34.1	1.000 (1.000, 1.000)	0.624583719
RSPO3	Diagnoses - main ICD10: Z42.1 Follow-up care involving plastic surgery of breast	Z42.1	1.000 (0.999, 1.000)	0.592670809
RSPO3	Type of cancer: ICD10: C50.9 Breast, unspecified	C50.9	0.999 (0.998, 1.000)	0.064679578
RSPO3	Diagnoses - main ICD10: J34.8 Other specified disorders of nose and nasal sinuses	J34.8	1.000 (0.999, 1.000)	0.163072621
RSPO3	Type of cancer: ICD10: C44.6 Skin of upper limb, including shoulder	C44.6	1.000 (1.000, 1.001)	0.492909717
RSPO3	Diagnoses - secondary ICD10: R50.9 Fever, unspecified	R50.9	1.000 (1.000, 1.001)	0.146474916
RSPO3	Diagnoses - secondary ICD10: W10.0 Home	W10.0	1.000 (1.000, 1.001)	0.559130372
RSPO3	Diagnoses - main ICD10: N84.1 Polyp of cervix uteri	N84.1	1.000 (0.999, 1.000)	0.72144184
RSPO3	Diagnoses - secondary ICD10: R33 Retention of urine	R33	1.000 (0.999, 1.001)	0.71854694
RSPO3	Diagnoses - main ICD10: D12.6 Colon, unspecified	D12.6	1.000 (0.999, 1.000)	0.594426895
RSPO3	Diagnoses - main ICD10: I63.9 Cerebral infarction, unspecified	I63.9	1.000 (0.999, 1.000)	0.337766581
RSPO3	Diagnoses - main ICD10: K51.9 Ulcerative colitis, unspecified	K51.9	1.000 (0.999, 1.000)	0.625825576
RSPO3	Diagnoses - main ICD10: K85 Acute pancreatitis	K85	1.000 (1.000, 1.001)	0.108237592

RSPO3	Diagnoses - secondary ICD10: R06.0 Dyspnoea	R06.0	1.000 (0.999, 1.000)	0.402271209
RSPO3	Diagnoses - secondary ICD10: R10.4 Other and unspecified abdominal pain	R10.4	1.000 (0.999, 1.001)	0.850952979
RSPO3	Diagnoses - main ICD10: Z47.0 Follow-up care involving removal of fracture plate and other internal	Z47.0	0.999 (0.998, 1.000)	0.003216228
RSPO3	Diagnoses - secondary ICD10: F10.2 Dependence syndrome	F10.2	1.000 (0.999, 1.000)	0.037970441
RSPO3	Diagnoses - main ICD10: R06.5 Mouth breathing	R06.5	1.000 (1.000, 1.001)	0.493783902
RSPO3	Diagnoses - main ICD10: R51 Headache	R51	1.000 (1.000, 1.001)	0.375275977
RSPO3	Diagnoses - main ICD10: K62.1 Rectal polyp	K62.1	1.000 (1.000, 1.001)	0.481435021
RSPO3	Diagnoses - main ICD10: M51.3 Other specified intervertebral disk degeneration	M51.3	1.000 (0.999, 1.000)	0.019118771
RSPO3	Diagnoses - main ICD10: M54.56 Low back pain (Lumbar region)	M54.56	1.000 (1.000, 1.001)	0.679649742
RSPO3	Diagnoses - main ICD10: M16.9 Coxarthrosis, unspecified	M16.9	1.000 (1.000, 1.001)	0.394910773
RSPO3	Diagnoses - secondary ICD10: Z86.1 Personal history of infectious and parasitic diseases	Z86.1	1.000 (0.999, 1.000)	0.802849501
RSPO3	Diagnoses - secondary ICD10: I12.0 Hypertensive renal disease with renal failure	I12.0	1.000 (0.999, 1.000)	0.299484986
RSPO3	Diagnoses - secondary ICD10: R05 Cough	R05	1.000 (0.999, 1.000)	0.119417713
RSPO3	Diagnoses - main ICD10: Z13.8 Special screening examination for other specified diseases and disorde	Z13.8	1.000 (1.000, 1.001)	0.536480761
RSPO3	Diagnoses - secondary ICD10: F32.9 Depressive episode, unspecified	F32.9	1.000 (0.999, 1.001)	0.839818867
RSPO3	Diagnoses - secondary ICD10: Z85.3 Personal history of malignant neoplasm of breast	Z85.3	0.999 (0.998, 1.000)	0.102734098
RSPO3	Diagnoses - main ICD10: K52.9 Non-infective gastro-enteritis and colitis, unspecified	K52.9	1.000 (0.999, 1.002)	0.436512564
RSPO3	Diagnoses - main ICD10: L98.9 Disorder of skin and subcutaneous tissue, unspecified	L98.9	1.000 (0.999, 1.000)	0.26737325
RSPO3	Diagnoses - secondary ICD10: M19.99 Arthrosis, unspecified (Site unspecified)	M19.99	1.000 (0.999, 1.001)	0.905653262
RSPO3	Diagnoses - main ICD10: Z12.1 Special screening examination for neoplasm of intestinal tract	Z12.1	1.001 (1.000, 1.001)	0.130114843
RSPO3	Diagnoses - main ICD10: R07.2 Precordial pain	R07.2	1.000 (0.999, 1.000)	0.596115261
RSPO3	Diagnoses - secondary ICD10: Z90.7 Acquired absence of genital organ(s)	Z90.7	1.000 (0.999, 1.001)	0.793378083
RSPO3	Diagnoses - secondary ICD10: Z96.6 Presence of orthopaedic joint implants	Z96.6	1.000 (0.999, 1.001)	0.623912559
RSPO3	Diagnoses - secondary ICD10: B96.8 Other specified bacterial agents as the cause of diseases classified	B96.8	0.999 (0.999, 1.000)	0.221643972
RSPO3	Diagnoses - secondary ICD10: R42 Dizziness and giddiness	R42	1.000 (1.000, 1.001)	0.413124754
RSPO3	Diagnoses - secondary ICD10: N81.2 Incomplete uterovaginal prolapse	N81.2	1.000 (0.999, 1.000)	0.483550597
RSPO3	Diagnoses - main ICD10: M25.56 Pain in joint (Lower leg)	M25.56	1.000 (1.000, 1.001)	0.769176875
RSPO3	Diagnoses - main ICD10: D17.2 Benign lipomatous neoplasm of skin and subcutaneous tissue of limbs	D17.2	1.000 (1.000, 1.001)	0.300480927
RSPO3	Diagnoses - main ICD10: O63.1 Prolonged second stage (of labour)	O63.1	1.000 (1.000, 1.001)	0.560761856
RSPO3	Diagnoses - secondary ICD10: Z82.4 Family history of ischaemic heart disease and other diseases of th	Z82.4	1.000 (0.999, 1.001)	0.906061191
RSPO3	Diagnoses - main ICD10: H25.0 Senile incipient cataract	H25.0	1.000 (1.000, 1.000)	0.679654168
RSPO3	Diagnoses - main ICD10: D17.1 Benign lipomatous neoplasm of skin and subcutaneous tissue of trunk	D17.1	1.000 (1.000, 1.001)	0.307041616
RSPO3	Diagnoses - secondary ICD10: M47.82 Other spondylosis (Cervical region)	M47.82	1.000 (1.000, 1.001)	0.450647839
RSPO3	Diagnoses - secondary ICD10: Z98.0 Intestinal bypass and anastomosis status	Z98.0	1.000 (1.000, 1.001)	0.501567252
RSPO3	Type of cancer: ICD10: C43.7 Malignant melanoma of lower limb, including hip	C43.7	1.000 (1.000, 1.000)	0.8070045
RSPO3	Diagnoses - main ICD10: S52.50 Fracture of lower end of radius (closed)	S52.50	0.999 (0.998, 0.999)	2.86374E-05
RSPO3	Diagnoses - secondary ICD10: W19.9 Unspecified place	W19.9	0.999 (0.999, 1.000)	0.057947855

RSPO3	Diagnoses - main ICD10: M16.1 Other primary coxarthrosis	M16.1	1.000 (0.999, 1.001)	0.680347664
RSPO3	Diagnoses - secondary ICD10: Z60.2 Living alone	Z60.2	1.000 (0.999, 1.001)	0.874233087
RSPO3	Diagnoses - secondary ICD10: F17.1 Harmful use	F17.1	1.000 (0.999, 1.000)	0.291330296
RSPO3	Diagnoses - secondary ICD10: K31.8 Other specified diseases of stomach and duodenum	K31.8	1.000 (1.000, 1.001)	0.22534014
RSPO3	Diagnoses - secondary ICD10: K31.7 Polyp of stomach and duodenum	K31.7	1.000 (0.999, 1.000)	0.528894661
RSPO3	Diagnoses - main ICD10: H25.1 Senile nuclear cataract	H25.1	1.000 (1.000, 1.001)	0.48184936
RSPO3	Diagnoses - secondary ICD10: Y83.2 Surgical operation with anastomosis, bypass or graft	Y83.2	1.000 (1.000, 1.000)	0.913764105
RSPO3	Diagnoses - secondary ICD10: D70 Agranulocytosis	D70	1.000 (0.999, 1.000)	0.107259055
RSPO3	Diagnoses - main ICD10: D50.9 Iron deficiency anaemia, unspecified	D50.9	1.000 (0.999, 1.000)	0.227508365
RSPO3	Diagnoses - secondary ICD10: N81.6 Rectocele	N81.6	1.000 (1.000, 1.001)	0.7353548
RSPO3	Diagnoses - main ICD10: I21.9 Acute myocardial infarction, unspecified	I21.9	1.000 (1.000, 1.001)	0.43138945
RSPO3	Diagnoses - main ICD10: D22.3 Melanocytic naevi of other and unspecified parts of face	D22.3	1.000 (1.000, 1.000)	0.957299892
RSPO3	Diagnoses - secondary ICD10: K44.9 Diaphragmatic hernia without obstruction or gangrene	K44.9	1.001 (1.000, 1.003)	0.121691967
RSPO3	Diagnoses - main ICD10: B34.9 Viral infection, unspecified	B34.9	1.000 (1.000, 1.001)	0.034595584
RSPO3	Diagnoses - main ICD10: N32.8 Other specified disorders of bladder	N32.8	1.000 (1.000, 1.001)	0.754678725
RSPO3	Diagnoses - main ICD10: J34.2 Deviated nasal septum	J34.2	1.000 (0.999, 1.001)	0.884814657
RSPO3	Diagnoses - main ICD10: N50.8 Other specified disorders of male genital organs	N50.8	1.000 (0.999, 1.000)	0.816669265
RSPO3	Diagnoses - secondary ICD10: Z86.0 Personal history of other neoplasms	Z86.0	1.000 (0.999, 1.000)	0.372612435
RSPO3	Diagnoses - secondary ICD10: Z72.1 Alcohol use	Z72.1	0.999 (0.999, 1.000)	0.045789489
RSPO3	Diagnoses - main ICD10: G56.0 Carpal tunnel syndrome	G56.0	1.001 (1.000, 1.002)	0.243193511
RSPO3	Diagnoses - main ICD10: I25.9 Chronic ischaemic heart disease, unspecified	I25.9	1.000 (1.000, 1.000)	0.731380031
RSPO3	Diagnoses - secondary ICD10: Z37.0 Single live birth	Z37.0	1.000 (0.998, 1.001)	0.566950276
RSPO3	Diagnoses - secondary ICD10: E03.9 Hypothyroidism, unspecified	E03.9	0.999 (0.998, 1.000)	0.182398529
RSPO3	Diagnoses - secondary ICD10: Z88.6 Personal history of allergy to analgesic agent	Z88.6	1.000 (0.999, 1.000)	0.125167221
RSPO3	Diagnoses - main ICD10: M20.2 Hallux rigidus	M20.2	1.000 (1.000, 1.001)	0.455966577
RSPO3	Diagnoses - secondary ICD10: B95.6 Staphylococcus aureus as the cause of diseases classified to other	B95.6	1.000 (0.999, 1.000)	0.585084886
RSPO3	Diagnoses - main ICD10: R04.2 Haemoptysis	R04.2	1.000 (1.000, 1.000)	0.922753493
RSPO3	Diagnoses - main ICD10: I21.0 Acute transmural myocardial infarction of anterior wall	I21.0	1.000 (1.000, 1.001)	0.617267555
RSPO3	Diagnoses - secondary ICD10: R94.5 Abnormal results of liver function studies	R94.5	1.000 (1.000, 1.001)	0.481743636
RSPO3	Diagnoses - secondary ICD10: T81.4 Infection following a procedure, not elsewhere classified	T81.4	1.000 (0.999, 1.000)	0.313785021
RSPO3	Diagnoses - secondary ICD10: Z88.8 Personal history of allergy to other drugs, medicaments and biol	Z88.8	1.000 (0.999, 1.000)	0.291272289
RSPO3	Diagnoses - main ICD10: H00.1 Chalazion	H00.1	1.000 (1.000, 1.001)	0.617499
RSPO3	Type of cancer: ICD10: D05.1 Intraductal carcinoma in situ	D05.1	1.000 (0.999, 1.000)	0.341179956
RSPO3	Diagnoses - secondary ICD10: Y43.3 Other antineoplastic drugs	Y43.3	1.000 (1.000, 1.000)	0.811124233
RSPO3	Diagnoses - main ICD10: K02.9 Dental caries, unspecified	K02.9	1.000 (1.000, 1.001)	0.866296906
RSPO3	Diagnoses - main ICD10: M72.04 Palmar fascial fibromatosis [Dupuytren]-Hand	M72.04	1.000 (1.000, 1.001)	0.144028144
RSPO3	Diagnoses - secondary ICD10: N83.2 Other and unspecified ovarian cysts	N83.2	1.000 (0.999, 1.000)	0.762338479

RSPO3	Diagnoses - main ICD10: M75.0 Adhesive capsulitis of shoulder	M75.0	1.000 (1.000, 1.000)	0.723495685
RSPO3	Diagnoses - secondary ICD10: I73.9 Peripheral vascular disease, unspecified	I73.9	1.000 (1.000, 1.001)	0.669724671
RSPO3	Diagnoses - secondary ICD10: N17.9 Acute renal failure, unspecified	N17.9	1.000 (1.000, 1.001)	0.64789838
RSPO3	Diagnoses - main ICD10: M75.1 Rotator cuff syndrome	M75.1	1.000 (0.999, 1.000)	0.314435073
RSPO3	Diagnoses - main ICD10: L82 Seborrhoeic keratosis	L82	1.000 (1.000, 1.001)	0.831580674
RSPO3	Diagnoses - main ICD10: K61.0 Anal abscess	K61.0	1.000 (1.000, 1.001)	0.19251439
RSPO3	Diagnoses - secondary ICD10: Z53.2 Procedure not carried out because of patient's decision for other	Z53.2	1.000 (1.000, 1.001)	0.144684153
RSPO3	Diagnoses - secondary ICD10: Z72.0 Tobacco use	Z72.0	0.999 (0.998, 1.001)	0.421723444
RSPO3	Diagnoses - main ICD10: I21.1 Acute transmural myocardial infarction of inferior wall	I21.1	1.000 (1.000, 1.001)	0.601257893
RSPO3	Diagnoses - secondary ICD10: N39.3 Stress incontinence	N39.3	1.000 (1.000, 1.000)	0.928315426
RSPO3	Diagnoses - main ICD10: K40.9 Unilateral or unspecified inguinal hernia, without obstruction or gang	K40.9	0.998 (0.997, 1.000)	0.019258485
RSPO3	Diagnoses - main ICD10: C67.9 Bladder, unspecified	C67.9	1.000 (1.000, 1.001)	0.061111268
RSPO3	Diagnoses - secondary ICD10: Z13.0 Special screening examination for diseases of the blood and bloo	Z13.0	1.000 (0.999, 1.000)	0.42963575
RSPO3	Diagnoses - secondary ICD10: Z86.6 Personal history of diseases of the nervous system and sense orga	Z86.6	1.000 (0.999, 1.001)	0.856330944
RSPO3	Diagnoses - main ICD10: K92.1 Melaena	K92.1	1.000 (0.999, 1.000)	0.731835721
RSPO3	Diagnoses - secondary ICD10: J34.3 Hypertrophy of nasal turbinates	J34.3	1.000 (1.000, 1.000)	0.736080903
RSPO3	Diagnoses - secondary ICD10: Z88.0 Personal history of allergy to penicillin	Z88.0	1.000 (0.999, 1.001)	0.901484553
RSPO3	Diagnoses - main ICD10: S82.80 Fractures of other parts of lower leg (closed)	S82.80	0.999 (0.999, 0.999)	4.00001E-05
RSPO3	Diagnoses - main ICD10: M20.1 Hallux valgus (acquired)	M20.1	1.001 (1.000, 1.002)	0.082700859
RSPO3	Diagnoses - secondary ICD10: N39.0 Urinary tract infection, site not specified	N39.0	1.000 (0.999, 1.000)	0.407434232
RSPO3	Diagnoses - secondary ICD10: Z03.4 Observation for suspected myocardial infarction	Z03.4	1.000 (1.000, 1.001)	0.475149268
RSPO3	Diagnoses - main ICD10: O80.0 Spontaneous vertex delivery	O80.0	1.000 (0.999, 1.000)	0.225396502
RSPO3	Diagnoses - secondary ICD10: N73.6 Female pelvic peritoneal adhesions	N73.6	1.000 (1.000, 1.001)	0.412667084
RSPO3	Diagnoses - secondary ICD10: D25.9 Leiomyoma of uterus, unspecified	D25.9	1.000 (1.000, 1.001)	0.355521938
RSPO3	Diagnoses - secondary ICD10: K26.9 Unspecified as acute or chronic, without haemorrhage or perfora	K26.9	1.000 (1.000, 1.000)	0.728381979
RSPO3	Diagnoses - main ICD10: K60.3 Anal fistula	K60.3	1.000 (1.000, 1.000)	0.586317437
RSPO3	Diagnoses - main ICD10: K43.9 Ventral hernia without obstruction or gangrene	K43.9	1.000 (1.000, 1.001)	0.47315554
RSPO3	Diagnoses - main ICD10: K35.9 Acute appendicitis, unspecified	K35.9	1.000 (1.000, 1.001)	0.480422594
RSPO3	Type of cancer: ICD10: C44.7 Skin of lower limb, including hip	C44.7	1.000 (1.000, 1.000)	0.820138165
RSPO3	Diagnoses - secondary ICD10: M15.9 Polyarthrosis, unspecified	M15.9	1.000 (0.999, 1.000)	0.146568418
RSPO3	Diagnoses - main ICD10: R13 Dysphagia	R13	1.000 (0.999, 1.000)	0.381288621
RSPO3	Diagnoses - main ICD10: M23.23 Derangement of meniscus due to old tear or injury (Medial collatera	M23.23	1.000 (0.999, 1.000)	0.372957786
RSPO3	Diagnoses - secondary ICD10: Z51.2 Other chemotherapy	Z51.2	0.999 (0.999, 1.000)	0.020678664
RSPO3	Diagnoses - main ICD10: J33.9 Nasal polyp, unspecified	J33.9	1.000 (0.999, 1.000)	0.123456671
RSPO3	Diagnoses - secondary ICD10: Z85.5 Personal history of malignant neoplasm of urinary tract	Z85.5	1.001 (1.000, 1.001)	0.048749224
RSPO3	Diagnoses - secondary ICD10: Z92.1 Personal history of long-term (current) use of anticoagulants	Z92.1	1.000 (0.999, 1.001)	0.582425276
RSPO3	Diagnoses - main ICD10: Z30.2 Sterilisation	Z30.2	1.000 (0.999, 1.001)	0.749066067

RSPO3	Diagnoses - main ICD10: N63 Unspecified lump in breast	N63	0.999 (0.999, 1.000)	0.032530378
RSPO3	Diagnoses - main ICD10: I84.8 Unspecified haemorrhoids with other complications	I84.8	1.000 (0.999, 1.001)	0.846480561
RSPO3	Diagnoses - main ICD10: Z46.6 Fitting and adjustment of urinary device	Z46.6	1.000 (0.999, 1.000)	0.653845981
RSPO3	Diagnoses - main ICD10: K01.1 Impacted teeth	K01.1	1.000 (1.000, 1.001)	0.300882613
RSPO3	Diagnoses - secondary ICD10: Z80.3 Family history of malignant neoplasm of breast	Z80.3	1.000 (0.999, 1.000)	0.034563536
RSPO3	Diagnoses - secondary ICD10: Y83.6 Removal of other organ (partial) (total)	Y83.6	1.000 (0.999, 1.001)	0.795855381
RSPO3	Diagnoses - main ICD10: M65.3 Trigger finger	M65.3	1.000 (1.000, 1.000)	0.751225055
RSPO3	Diagnoses - secondary ICD10: Z92.4 Personal history of major surgery, not elsewhere classified	Z92.4	1.000 (1.000, 1.001)	0.374760105
RSPO3	Diagnoses - main ICD10: M23.2 Derangement of meniscus due to old tear or injury	M23.2	1.000 (0.999, 1.000)	0.361638174
RSPO3	Diagnoses - main ICD10: K80.5 Calculus of bile duct without cholangitis or cholecystitis	K80.5	1.000 (0.999, 1.000)	0.552165951
RSPO3	Type of cancer: ICD10: C44.9 Malignant neoplasm of skin, unspecified	C44.9	1.000 (0.999, 1.001)	0.634078119
RSPO3	Diagnoses - main ICD10: D24 Benign neoplasm of breast	D24	1.000 (1.000, 1.000)	0.936794388
RSPO3	Diagnoses - secondary ICD10: N85.8 Other specified noninflammatory disorders of uterus	N85.8	1.000 (1.000, 1.000)	0.878401769
RSPO3	Diagnoses - secondary ICD10: I95.9 Hypotension, unspecified	I95.9	1.000 (1.000, 1.001)	0.665437788
RSPO3	Diagnoses - secondary ICD10: B96.2 Escherichia coli [E. coli] as the cause of diseases classified to oth	B96.2	1.000 (0.999, 1.000)	0.578330413
RSPO3	Diagnoses - main ICD10: K80.1 Calculus of gallbladder with other cholecystitis	K80.1	1.000 (0.999, 1.001)	0.73561878
RSPO3	Diagnoses - secondary ICD10: Z53.0 Procedure not carried out because of contraindication	Z53.0	1.000 (0.999, 1.001)	0.646553772
RSPO3	Diagnoses - secondary ICD10: Z53.8 Procedure not carried out for other reasons	Z53.8	1.000 (0.999, 1.001)	0.857550323
RSPO3	Diagnoses - main ICD10: N35.9 Urethral stricture, unspecified	N35.9	1.000 (1.000, 1.001)	0.411423816
RSPO3	Type of cancer: ICD10: D06.9 Cervix, unspecified	D06.9	1.000 (0.999, 1.000)	0.506460364
RSPO3	Diagnoses - secondary ICD10: Z53.9 Procedure not carried out, unspecified reason	Z53.9	1.000 (0.999, 1.000)	0.607600825
RSPO3	Diagnoses - main ICD10: O68.0 Labour and delivery complicated by foetal heart rate anomaly	O68.0	1.000 (1.000, 1.000)	0.733975757
RSPO3	Diagnoses - main ICD10: D12.8 Rectum	D12.8	1.000 (1.000, 1.001)	0.616643674
RSPO3	Diagnoses - secondary ICD10: M19.9 Arthrosis, unspecified	M19.9	1.000 (0.999, 1.000)	0.230916525
RSPO3	Diagnoses - main ICD10: H02.8 Other specified disorders of eyelid	H02.8	1.000 (1.000, 1.001)	0.396839506
RSPO3	Diagnoses - main ICD10: N92.1 Excessive and frequent menstruation with irregular cycle	N92.1	1.000 (1.000, 1.001)	0.07747123
RSPO3	Diagnoses - main ICD10: O70.0 First degree perineal laceration during delivery	O70.0	1.000 (0.999, 1.000)	0.50084981
RSPO3	Diagnoses - secondary ICD10: N80.0 Endometriosis of uterus	N80.0	1.000 (0.999, 1.000)	0.276705513
RSPO3	Diagnoses - main ICD10: M23.22 Derangement of meniscus due to old tear or injury (Posterior cruciat	M23.22	1.000 (1.000, 1.001)	0.322859199
RSPO3	Diagnoses - secondary ICD10: E11.9 Without complications	E11.9	1.000 (0.998, 1.001)	0.473178926
RSPO3	Diagnoses - main ICD10: M79.66 Pain in limb (Lower leg)	M79.66	1.000 (1.000, 1.001)	0.322615343
RSPO3	Diagnoses - main ICD10: O26.8 Other specified pregnancy-related conditions	O26.8	1.000 (1.000, 1.001)	0.617380592
RSPO3	Diagnoses - main ICD10: K56.6 Other and unspecified intestinal obstruction	K56.6	1.000 (0.999, 1.000)	0.057247822
RSPO3	Diagnoses - secondary ICD10: Z88.1 Personal history of allergy to other antibiotic agents	Z88.1	1.000 (0.999, 1.000)	0.605294264
RSPO3	Diagnoses - secondary ICD10: J90 Pleural effusion, not elsewhere classified	J90	1.000 (0.999, 1.001)	0.893354815
RSPO3	Underlying (primary) cause of death: ICD10: C34.9 Bronchus or lung, unspecified	C34.9	1.010 (0.990, 1.029)	0.338273008
RSPO3	Diagnoses - main ICD10: A41 Other septicaemia	A41	1.000 (0.999, 1.000)	0.6287668

RSPO3	Diagnoses - main ICD10: C18 Malignant neoplasm of colon	C18	1.001 (1.000, 1.001)	0.191406855
RSPO3	Diagnoses - main ICD10: C34 Malignant neoplasm of bronchus and lung	C34	1.000 (0.999, 1.000)	0.453449443
RSPO3	Diagnoses - main ICD10: C43 Malignant melanoma of skin	C43	1.000 (0.999, 1.001)	0.770818482
RSPO3	Diagnoses - main ICD10: C67 Malignant neoplasm of bladder	C67	1.001 (1.000, 1.001)	0.032332676
RSPO3	Diagnoses - main ICD10: C78 Secondary malignant neoplasm of respiratory and digestive organs	C78	1.000 (0.999, 1.001)	0.803830382
RSPO3	Diagnoses - main ICD10: C79 Secondary malignant neoplasm of other sites	C79	1.000 (0.999, 1.001)	0.976500517
RSPO3	Diagnoses - main ICD10: D05 Carcinoma in situ of breast	D05	1.000 (0.999, 1.000)	0.223783574
RSPO3	Diagnoses - main ICD10: D17 Benign lipomatous neoplasm	D17	1.000 (0.999, 1.001)	0.58128199
RSPO3	Diagnoses - main ICD10: D22 Melanocytic naevi	D22	1.000 (0.999, 1.001)	0.432828347
RSPO3	Diagnoses - main ICD10: D23 Other benign neoplasms of skin	D23	1.000 (0.999, 1.001)	0.47102051
RSPO3	Diagnoses - main ICD10: D50 Iron deficiency anaemia	D50	1.000 (0.999, 1.001)	0.65679328
RSPO3	Diagnoses - main ICD10: D64 Other anaemias	D64	1.000 (0.999, 1.001)	0.551121259
RSPO3	Diagnoses - main ICD10: G43 Migraine	G43	1.000 (1.000, 1.001)	0.47653939
RSPO3	Diagnoses - main ICD10: G45 Transient cerebral ischaemic attacks and related syndromes	G45	1.000 (0.999, 1.001)	0.948939914
RSPO3	Diagnoses - main ICD10: G57 Mononeuropathies of lower limb	G57	1.000 (1.000, 1.001)	0.310459401
RSPO3	Diagnoses - main ICD10: H00 Hordeolum and chalazion	H00	1.000 (1.000, 1.001)	0.632718952
RSPO3	Diagnoses - main ICD10: H02 Other disorders of eyelid	H02	1.000 (0.999, 1.001)	0.403314831
RSPO3	Diagnoses - main ICD10: H04 Disorders of lachrymal system	H04	1.000 (0.999, 1.001)	0.842351738
RSPO3	Diagnoses - main ICD10: H33 Retinal detachments and breaks	H33	1.000 (0.999, 1.001)	0.574052329
RSPO3	Diagnoses - main ICD10: H35 Other retinal disorders	H35	0.999 (0.999, 1.000)	0.019825162
RSPO3	Diagnoses - main ICD10: H40 Glaucoma	H40	1.000 (1.000, 1.001)	0.297642536
RSPO3	Diagnoses - main ICD10: I26 Pulmonary embolism	I26	1.000 (1.000, 1.001)	0.270705483
RSPO3	Diagnoses - main ICD10: I47 Paroxysmal tachycardia	I47	1.000 (0.999, 1.000)	0.31692475
RSPO3	Diagnoses - main ICD10: I50 Heart failure	I50	1.000 (0.999, 1.001)	0.933348429
RSPO3	Diagnoses - main ICD10: I63 Cerebral infarction	I63	1.000 (0.999, 1.001)	0.845993081
RSPO3	Diagnoses - main ICD10: J18 Pneumonia, organism unspecified	J18	0.999 (0.998, 1.000)	0.054331128
RSPO3	Diagnoses - main ICD10: J32 Chronic sinusitis	J32	1.000 (0.999, 1.000)	0.10620805
RSPO3	Diagnoses - main ICD10: J38 Diseases of vocal cords and larynx, not elsewhere classified	J38	1.000 (0.999, 1.000)	0.802309715
RSPO3	Diagnoses - main ICD10: J45 Asthma	J45	1.000 (0.999, 1.001)	0.650945319
RSPO3	Diagnoses - main ICD10: K01 Embedded and impacted teeth	K01	1.001 (1.000, 1.001)	0.077212328
RSPO3	Diagnoses - main ICD10: K02 Dental caries	K02	1.000 (0.999, 1.001)	0.613046287
RSPO3	Diagnoses - main ICD10: K04 Diseases of pulp and periapical tissues	K04	1.000 (0.999, 1.001)	0.698189203
RSPO3	Diagnoses - main ICD10: K08 Other disorders of teeth and supporting structures	K08	1.000 (1.000, 1.001)	0.37043797
RSPO3	Diagnoses - main ICD10: K13 Other diseases of lip and oral mucosa	K13	1.000 (0.999, 1.001)	0.743308588
RSPO3	Diagnoses - main ICD10: K25 Gastric ulcer	K25	1.001 (1.000, 1.001)	0.079825563
RSPO3	Diagnoses - main ICD10: K26 Duodenal ulcer	K26	1.000 (0.999, 1.000)	0.120294246
RSPO3	Diagnoses - main ICD10: K31 Other diseases of stomach and duodenum	K31	1.001 (1.000, 1.001)	0.146319059

RSPO3	Diagnoses - main ICD10: K42 Umbilical hernia	K42	0.999 (0.998, 1.000)	0.045689939
RSPO3	Diagnoses - main ICD10: K56 Paralytic ileus and intestinal obstruction without hernia	K56	1.000 (0.999, 1.001)	0.703270579
RSPO3	Diagnoses - main ICD10: K58 Irritable bowel syndrome	K58	1.000 (0.999, 1.000)	0.866183981
RSPO3	Diagnoses - main ICD10: K59 Other functional intestinal disorders	K59	1.000 (0.999, 1.001)	0.792041997
RSPO3	Diagnoses - main ICD10: K61 Abscess of anal and rectal regions	K61	1.000 (1.000, 1.001)	0.622421308
RSPO3	Diagnoses - main ICD10: K63 Other diseases of intestine	K63	1.002 (1.000, 1.003)	0.039296923
RSPO3	Diagnoses - main ICD10: K81 Cholecystitis	K81	1.001 (1.000, 1.002)	0.002047986
RSPO3	Diagnoses - main ICD10: K92 Other diseases of digestive system	K92	1.000 (0.999, 1.001)	0.847604683
RSPO3	Diagnoses - main ICD10: L02 Cutaneous abscess, furuncle and carbuncle	L02	1.000 (0.999, 1.000)	0.226559217
RSPO3	Diagnoses - main ICD10: L57 Skin changes due to chronic exposure to nonionising radiation	L57	1.000 (1.000, 1.001)	0.527765716
RSPO3	Diagnoses - main ICD10: L72 Follicular cysts of skin and subcutaneous tissue	L72	1.000 (0.999, 1.002)	0.670670378
RSPO3	Diagnoses - main ICD10: L90 Atrophic disorders of skin	L90	1.000 (1.000, 1.001)	0.356533391
RSPO3	Diagnoses - main ICD10: L98 Other disorders of skin and subcutaneous tissue, not elsewhere classified	L98	1.000 (0.999, 1.001)	0.758098354
RSPO3	Diagnoses - main ICD10: M06 Other rheumatoid arthritis	M06	1.000 (1.000, 1.001)	0.324851647
RSPO3	Diagnoses - main ICD10: M13 Other arthritis	M13	1.000 (1.000, 1.001)	0.58324681
RSPO3	Diagnoses - main ICD10: M15 Polyarthrosis	M15	1.000 (0.999, 1.000)	0.222775793
RSPO3	Diagnoses - main ICD10: M19 Other arthrosis	M19	1.000 (0.999, 1.001)	0.615911832
RSPO3	Diagnoses - main ICD10: M47 Spondylosis	M47	1.000 (0.999, 1.001)	0.879384232
RSPO3	Diagnoses - main ICD10: M48 Other spondylopathies	M48	1.000 (0.999, 1.000)	0.5443032
RSPO3	Diagnoses - main ICD10: M51 Other intervertebral disk disorders	M51	1.000 (0.999, 1.001)	0.844042592
RSPO3	Diagnoses - main ICD10: M65 Synovitis and tenosynovitis	M65	1.000 (0.999, 1.001)	0.715233883
RSPO3	Diagnoses - main ICD10: M75 Shoulder lesions	M75	1.000 (0.998, 1.001)	0.627721288
RSPO3	Diagnoses - main ICD10: M79 Other soft tissue disorders, not elsewhere classified	M79	1.001 (0.999, 1.002)	0.428085802
RSPO3	Diagnoses - main ICD10: M84 Disorders of continuity of bone	M84	1.000 (0.999, 1.000)	0.402309386
RSPO3	Diagnoses - main ICD10: N13 Obstructive and reflux uropathy	N13	1.000 (0.999, 1.000)	0.277091535
RSPO3	Diagnoses - main ICD10: N30 Cystitis	N30	1.000 (0.999, 1.001)	0.744200371
RSPO3	Diagnoses - main ICD10: N31 Neuromuscular dysfunction of bladder, not elsewhere classified	N31	1.000 (0.999, 1.000)	0.70345293
RSPO3	Diagnoses - main ICD10: N35 Urethral stricture	N35	1.000 (0.999, 1.001)	0.869841635
RSPO3	Diagnoses - main ICD10: N39 Other disorders of urinary system	N39	1.000 (0.998, 1.001)	0.684254333
RSPO3	Diagnoses - main ICD10: N48 Other disorders of penis	N48	1.000 (1.000, 1.001)	0.131957167
RSPO3	Diagnoses - main ICD10: N50 Other disorders of male genital organs	N50	1.000 (0.999, 1.001)	0.961341775
RSPO3	Diagnoses - main ICD10: N60 Benign mammary dysplasia	N60	1.000 (0.999, 1.000)	0.344640348
RSPO3	Diagnoses - main ICD10: N80 Endometriosis	N80	1.001 (1.000, 1.002)	0.002602009
RSPO3	Diagnoses - main ICD10: N83 Noninflammatory disorders of ovary, Fallopian tube and broad ligamen	N83	1.000 (0.999, 1.000)	0.347725762
RSPO3	Diagnoses - main ICD10: N84 Polyp of female genital tract	N84	1.000 (0.999, 1.002)	0.739837479
RSPO3	Diagnoses - main ICD10: N85 Other noninflammatory disorders of uterus, except cervix	N85	1.000 (1.000, 1.001)	0.334185778
RSPO3	Diagnoses - main ICD10: N87 Dysplasia of cervix uteri	N87	1.000 (1.000, 1.001)	0.468499603

RSPO3	Diagnoses - main ICD10: N90 Other noninflammatory disorders of vulva and perineum	N90	1.000 (0.999, 1.000)	0.708482868
RSPO3	Diagnoses - main ICD10: N93 Other abnormal uterine and vaginal bleeding	N93	1.000 (0.999, 1.001)	0.897633324
RSPO3	Diagnoses - main ICD10: N94 Pain and other conditions associated with female genital organs and mer	N94	1.000 (0.999, 1.000)	0.356521229
RSPO3	Diagnoses - main ICD10: N95 Menopausal and other perimenopausal disorders	N95	1.000 (0.999, 1.002)	0.649452649
RSPO3	Diagnoses - main ICD10: O02 Other abnormal products of conception	O02	1.001 (1.000, 1.001)	0.049001418
RSPO3	Diagnoses - main ICD10: O03 Spontaneous abortion	O03	1.000 (1.000, 1.001)	0.187588635
RSPO3	Diagnoses - main ICD10: O26 Maternal care for other conditions predominantly related to pregnancy	O26	1.000 (1.000, 1.001)	0.171686638
RSPO3	Diagnoses - main ICD10: O36 Maternal care for other known or suspected foetal problems	O36	1.000 (1.000, 1.001)	0.53773154
RSPO3	Diagnoses - main ICD10: O63 Long labour	O63	1.000 (0.999, 1.000)	0.712418888
RSPO3	Diagnoses - main ICD10: O68 Labour and delivery complicated by foetal stress [distress]	O68	1.000 (0.999, 1.000)	0.541389499
RSPO3	Diagnoses - main ICD10: O70 Perineal laceration during delivery	O70	1.000 (0.999, 1.001)	0.566465935
RSPO3	Diagnoses - main ICD10: O80 Single spontaneous delivery	O80	1.000 (0.999, 1.000)	0.138229068
RSPO3	Diagnoses - main ICD10: R00 Abnormalities of heart beat	R00	1.000 (0.999, 1.001)	0.973473554
RSPO3	Diagnoses - main ICD10: R06 Abnormalities of breathing	R06	0.999 (0.998, 1.000)	0.179261561
RSPO3	Diagnoses - main ICD10: R19 Other symptoms and signs involving the digestive system and abdomen	R19	1.000 (0.998, 1.001)	0.891396687
RSPO3	Diagnoses - main ICD10: R22 Localised swelling, mass and lump of skin and subcutaneous tissue	R22	1.001 (1.000, 1.001)	0.084317845
RSPO3	Diagnoses - main ICD10: R39 Other symptoms and signs involving the urinary system	R39	1.000 (0.999, 1.001)	0.628080236
RSPO3	Diagnoses - main ICD10: R50 Fever of unknown origin	R50	1.000 (0.999, 1.001)	0.899879052
RSPO3	Diagnoses - main ICD10: R56 Convulsions, not elsewhere classified	R56	1.000 (1.000, 1.001)	0.368146607
RSPO3	Diagnoses - main ICD10: R59 Enlarged lymph nodes	R59	1.000 (1.000, 1.001)	0.950283518
RSPO3	Diagnoses - main ICD10: R63 Symptoms and signs concerning food and fluid intake	R63	1.000 (1.000, 1.001)	0.144698053
RSPO3	Diagnoses - main ICD10: R79 Other abnormal findings of blood chemistry	R79	1.000 (0.999, 1.000)	0.361212531
RSPO3	Diagnoses - main ICD10: R87 Abnormal findings in specimens from female genital organs	R87	1.000 (0.999, 1.000)	0.060015274
RSPO3	Diagnoses - main ICD10: R93 Abnormal findings on diagnostic imaging of other body structures	R93	1.000 (0.999, 1.000)	0.61726681
RSPO3	Diagnoses - main ICD10: S01 Open wound of head	S01	1.000 (1.000, 1.001)	0.301098363
RSPO3	Diagnoses - main ICD10: S02 Fracture of skull and facial bones	S02	1.000 (0.999, 1.000)	0.356382559
RSPO3	Diagnoses - main ICD10: S42 Fracture of shoulder and upper arm	S42	0.999 (0.998, 1.000)	0.007683806
RSPO3	Diagnoses - main ICD10: S61 Open wound of wrist and hand	S61	1.000 (0.999, 1.001)	0.906957776
RSPO3	Diagnoses - main ICD10: S62 Fracture at wrist and hand level	S62	0.999 (0.998, 1.000)	0.00706381
RSPO3	Diagnoses - main ICD10: S72 Fracture of femur	S72	0.999 (0.999, 1.000)	0.082339296
RSPO3	Diagnoses - main ICD10: S82 Fracture of lower leg, including ankle	S82	0.998 (0.997, 0.999)	0.001182101
RSPO3	Diagnoses - main ICD10: T39 Poisoning by nonopioid analgesics, antipyretics and antirheumatics	T39	1.000 (0.999, 1.000)	0.427073017
RSPO3	Diagnoses - main ICD10: T81 Complications of procedures, not elsewhere classified	T81	0.999 (0.998, 1.000)	0.209192266
RSPO3	Diagnoses - main ICD10: T82 Complications of cardiac and vascular prosthetic devices, implants and g	T82	1.000 (0.999, 1.001)	0.935840698
RSPO3	Diagnoses - main ICD10: T85 Complications of other internal prosthetic devices, implants and grafts	T85	1.000 (0.999, 1.000)	0.119389558
RSPO3	Diagnoses - main ICD10: Z01 Other special examinations and investigations of persons without compla	Z01	1.000 (1.000, 1.001)	0.18023042
RSPO3	Diagnoses - main ICD10: Z03 Medical observation and evaluation for suspected diseases and conditior	Z03	0.999 (0.998, 1.000)	0.216221379

RSPO3	Diagnoses - main ICD10: Z08 Follow-up examination after treatment for malignant neoplasm	Z08	1.001 (1.000, 1.002)	0.16755703
RSPO3	Diagnoses - main ICD10: Z12 Special screening examination for neoplasms	Z12	1.001 (1.000, 1.002)	0.16374401
RSPO3	Diagnoses - main ICD10: Z13 Special screening examination for other diseases and disorders	Z13	1.000 (1.000, 1.001)	0.335886455
RSPO3	Diagnoses - main ICD10: Z30 Contraceptive management	Z30	1.000 (0.999, 1.001)	0.741094339
RSPO3	Diagnoses - main ICD10: Z42 Follow-up care involving plastic surgery	Z42	0.999 (0.999, 1.000)	0.0998924
RSPO3	Diagnoses - main ICD10: Z43 Attention to artificial openings	Z43	1.000 (1.000, 1.001)	0.403798311
RSPO3	Diagnoses - main ICD10: Z45 Adjustment and management of implanted device	Z45	1.000 (0.999, 1.001)	0.758920027
RSPO3	Diagnoses - main ICD10: Z46 Fitting and adjustment of other devices	Z46	0.999 (0.998, 1.000)	0.16581745
RSPO3	Diagnoses - main ICD10: Z53 Persons encountering health services for specifie procedures, not carried	Z53	1.000 (1.000, 1.001)	0.634042636

Table S13 Phenome-wide Mendelian Randomization analysis to reveal potential on-target side effects of SARS2

protein	disease	ICD-10 code	OR (95%CI)	P value
SARS2	Underlying (primary) cause of death: ICD10: E85.4 Organ-limited amyloidosis	E85.4	0.997 (0.988, 1.006)	0.471738302
SARS2	Underlying (primary) cause of death: ICD10: J84.1 Other interstitial pulmonary diseases with fibrosis	J84.1	1.001 (0.969, 1.034)	0.952240401
SARS2	Diagnoses - main ICD10: B37 Candidiasis	B37	1.000 (0.999, 1.001)	0.9773832
SARS2	Diagnoses - main ICD10: C44 Other malignant neoplasms of skin	C44	0.997 (0.992, 1.002)	0.304283901
SARS2	Diagnoses - main ICD10: C50 Malignant neoplasm of breast	C50	1.003 (0.998, 1.009)	0.247587726
SARS2	Diagnoses - main ICD10: C61 Malignant neoplasm of prostate	C61	0.998 (0.995, 1.002)	0.30518227
SARS2	Diagnoses - main ICD10: D12 Benign neoplasm of colon rectum anus and anal canal	D12	1.003 (0.998, 1.008)	0.308837299
SARS2	Diagnoses - main ICD10: D25 Leiomyoma of uterus	D25	0.999 (0.995, 1.004)	0.832695041
SARS2	Diagnoses - main ICD10: E03 Other hypothyroidism	E03	0.999 (0.999, 1.000)	0.072244707
SARS2	Diagnoses - main ICD10: E04 Other non-toxic goitre	E04	1.001 (0.999, 1.003)	0.396922314
SARS2	Diagnoses - main ICD10: F31 Bipolar affective disorder	F31	1.001 (0.999, 1.002)	0.233140297
SARS2	Diagnoses - main ICD10: F43 Reaction to severe stress and adjustment disorders	F43	1.000 (0.999, 1.001)	0.505175851
SARS2	Diagnoses - main ICD10: G47 Sleep disorders	G47	0.997 (0.994, 1.000)	0.090019703
SARS2	Diagnoses - main ICD10: G56 Mononeuropathies of upper limb	G56	1.005 (0.999, 1.011)	0.081249577
SARS2	Diagnoses - main ICD10: H25 Senile cataract	H25	1.000 (0.996, 1.003)	0.836787387
SARS2	Diagnoses - main ICD10: H26 Other cataract	H26	1.004 (0.998, 1.009)	0.201813115
SARS2	Diagnoses - main ICD10: I10 Essential (primary) hypertension	I10	1.001 (1.000, 1.003)	0.133055901
SARS2	Diagnoses - main ICD10: I20 Angina pectoris	I20	0.999 (0.994, 1.004)	0.602602566
SARS2	Diagnoses - main ICD10: I21 Acute myocardial infarction	I21	0.999 (0.995, 1.004)	0.744026085
SARS2	Diagnoses - main ICD10: I25 Chronic ischaemic heart disease	I25	0.994 (0.988, 1.001)	0.099107302
SARS2	Diagnoses - main ICD10: I30 Acute pericarditis	I30	1.000 (0.999, 1.001)	0.648979343
SARS2	Diagnoses - main ICD10: I48 Atrial fibrillation and flutter	I48	0.997 (0.993, 1.002)	0.274174661
SARS2	Diagnoses - main ICD10: I80 Phlebitis and thrombophlebitis	I80	1.001 (0.998, 1.004)	0.425284214
SARS2	Diagnoses - main ICD10: I84 Haemorrhoids	I84	0.993 (0.986, 1.000)	0.03514707
SARS2	Diagnoses - main ICD10: J22 Unspecified acute lower respiratory infection	J22	1.000 (0.997, 1.004)	0.91055375
SARS2	Diagnoses - main ICD10: J33 Nasal polyp	J33	1.000 (0.997, 1.003)	0.853156089
SARS2	Diagnoses - main ICD10: J34 Other disorders of nose and nasal sinuses	J34	0.995 (0.991, 0.999)	0.023930229
SARS2	Diagnoses - main ICD10: J44 Other chronic obstructive pulmonary disease	J44	1.002 (1.000, 1.004)	0.11443785
SARS2	Diagnoses - main ICD10: K20 Oesophagitis	K20	1.000 (0.996, 1.005)	0.879111378
SARS2	Diagnoses - main ICD10: K21 Gastro-oesophageal reflux disease	K21	0.998 (0.992, 1.004)	0.570412521
SARS2	Diagnoses - main ICD10: K22 Other diseases of oesophagus	K22	1.001 (0.997, 1.005)	0.678484945
SARS2	Diagnoses - main ICD10: K29 Gastritis and duodenitis	K29	1.004 (0.997, 1.010)	0.262314353
SARS2	Diagnoses - main ICD10: K35 Acute appendicitis	K35	1.001 (0.998, 1.004)	0.498353196
SARS2	Diagnoses - main ICD10: K40 Inguinal hernia	K40	1.002 (0.995, 1.009)	0.554253296
SARS2	Diagnoses - main ICD10: K43 Ventral hernia	K43	1.004 (1.001, 1.007)	0.007098123

SARS2	Diagnoses - main ICD10: K44 Diaphragmatic hernia	K44	0.999 (0.994, 1.004)	0.687152537
SARS2	Diagnoses - main ICD10: K50 Crohn's disease [regional enteritis]	K50	1.000 (0.998, 1.002)	0.657194284
SARS2	Diagnoses - main ICD10: K51 Ulcerative colitis	K51	0.997 (0.994, 1.000)	0.059592607
SARS2	Diagnoses - main ICD10: K52 Other non-infective gastro-enteritis and colitis	K52	1.001 (0.995, 1.007)	0.677496356
SARS2	Diagnoses - main ICD10: K57 Diverticular disease of intestine	K57	1.005 (0.999, 1.011)	0.087950171
SARS2	Diagnoses - main ICD10: K60 Fissure and fistula of anal and rectal regions	K60	0.999 (0.996, 1.002)	0.710162417
SARS2	Diagnoses - main ICD10: K62 Other diseases of anus and rectum	K62	1.006 (0.999, 1.013)	0.098375406
SARS2	Diagnoses - main ICD10: K76 Other diseases of liver	K76	1.000 (0.999, 1.002)	0.678244691
SARS2	Diagnoses - main ICD10: K80 Cholelithiasis	K80	1.001 (0.995, 1.007)	0.755920947
SARS2	Diagnoses - main ICD10: L03 Cellulitis	L03	0.999 (0.995, 1.003)	0.627590097
SARS2	Diagnoses - main ICD10: M10 Gout	M10	1.000 (0.999, 1.001)	0.390602313
SARS2	Diagnoses - main ICD10: M16 Coxarthrosis [arthrosis of hip]	M16	1.002 (0.997, 1.007)	0.511552158
SARS2	Diagnoses - main ICD10: M17 Gonarthrosis [arthrosis of knee]	M17	0.994 (0.989, 1.000)	0.068709331
SARS2	Diagnoses - main ICD10: M20 Acquired deformities of fingers and toes	M20	1.001 (0.996, 1.006)	0.698960372
SARS2	Diagnoses - main ICD10: M21 Other acquired deformities of limbs	M21	1.000 (0.998, 1.001)	0.938001447
SARS2	Diagnoses - main ICD10: M23 Internal derangement of knee	M23	1.002 (0.995, 1.008)	0.609067484
SARS2	Diagnoses - main ICD10: M24 Other specific joint derangements	M24	1.002 (1.000, 1.004)	0.125930232
SARS2	Diagnoses - main ICD10: M25 Other joint disorders not elsewhere classified	M25	0.995 (0.990, 1.001)	0.076859315
SARS2	Diagnoses - main ICD10: M54 Dorsalgia	M54	0.997 (0.991, 1.002)	0.217436791
SARS2	Diagnoses - main ICD10: M67 Other disorders of synovium and tendon	M67	1.000 (0.997, 1.003)	0.976228404
SARS2	Diagnoses - main ICD10: M70 Soft tissue disorders related to use overuse and pressure	M70	0.997 (0.995, 0.999)	0.004620894
SARS2	Diagnoses - main ICD10: M72 Fibroblastic disorders	M72	1.000 (0.997, 1.003)	0.93793862
SARS2	Diagnoses - main ICD10: N19 Unspecified renal failure	N19	1.000 (0.999, 1.001)	0.510727955
SARS2	Diagnoses - main ICD10: N20 Calculus of kidney and ureter	N20	0.998 (0.994, 1.001)	0.241305783
SARS2	Diagnoses - main ICD10: N32 Other disorders of bladder	N32	1.000 (0.996, 1.004)	0.878280604
SARS2	Diagnoses - main ICD10: N40 Hyperplasia of prostate	N40	1.004 (0.999, 1.008)	0.087818905
SARS2	Diagnoses - main ICD10: N81 Female genital prolapse	N81	0.999 (0.994, 1.004)	0.693696073
SARS2	Diagnoses - main ICD10: N92 Excessive frequent and irregular menstruation	N92	0.999 (0.993, 1.005)	0.801452675
SARS2	Diagnoses - main ICD10: O75 Other complications of labour and delivery not elsewhere classified	O75	1.000 (0.999, 1.001)	0.679748398
SARS2	Diagnoses - main ICD10: R04 Haemorrhage from respiratory passages	R04	1.002 (0.999, 1.005)	0.175482715
SARS2	Diagnoses - main ICD10: R07 Pain in throat and chest	R07	1.005 (0.996, 1.015)	0.276474742
SARS2	Diagnoses - main ICD10: R10 Abdominal and pelvic pain	R10	0.998 (0.989, 1.006)	0.619845181
SARS2	Diagnoses - main ICD10: R11 Nausea and vomiting	R11	1.002 (0.999, 1.004)	0.27843943
SARS2	Diagnoses - main ICD10: R14 Flatulence and related conditions	R14	1.000 (0.998, 1.001)	0.378949996
SARS2	Diagnoses - main ICD10: R31 Unspecified haematuria	R31	0.992 (0.986, 0.998)	0.012073826
SARS2	Diagnoses - main ICD10: R35 Polyuria	R35	1.001 (0.998, 1.003)	0.631950341
SARS2	Diagnoses - main ICD10: R55 Syncope and collapse	R55	1.004 (1.000, 1.008)	0.047124947

SARS2	Diagnoses - main ICD10: R69 Unknown and unspecified causes of morbidity	R69	1.004 (0.998, 1.010)	0.234791899
SARS2	Diagnoses - main ICD10: S09 Other and unspecified injuries of head	S09	1.000 (0.997, 1.002)	0.805257336
SARS2	Diagnoses - main ICD10: S52 Fracture of forearm	S52	1.001 (0.997, 1.005)	0.672500354
SARS2	Diagnoses - main ICD10: S66 Injury of muscle and tendon at wrist and hand level	S66	1.000 (0.999, 1.002)	0.760103936
SARS2	Diagnoses - main ICD10: S76 Injury of muscle and tendon at hip and thigh level	S76	1.000 (0.999, 1.001)	0.629667171
SARS2	Diagnoses - main ICD10: T84 Complications of internal orthopaedic prosthetic devices implants and g	T84	0.996 (0.993, 1.000)	0.035754411
SARS2	Diagnoses - main ICD10: Z09 Follow-up examination after treatment for conditions other than maligna	Z09	0.995 (0.990, 1.001)	0.087455788
SARS2	Diagnoses - main ICD10: Z47 Other orthopaedic follow-up care	Z47	1.000 (0.996, 1.003)	0.84362847
SARS2	Diagnoses - main ICD10: Z80 Family history of malignant neoplasm	Z80	0.999 (0.998, 1.001)	0.271140285
SARS2	Diagnoses - secondary ICD10: Z50.1 Other physical therapy	Z50.1	0.999 (0.997, 1.001)	0.48046744
SARS2	Diagnoses - main ICD10: Z09.0 Follow-up examination after surgery for other conditions	Z09.0	0.998 (0.995, 1.001)	0.1605716
SARS2	Diagnoses - secondary ICD10: K21.0 Gastro-oesophageal reflux disease with oesophagitis	K21.0	1.001 (0.999, 1.004)	0.291191199
SARS2	Type of cancer: ICD10: C44.3 Skin of other and unspecified parts of face	C44.3	0.998 (0.994, 1.002)	0.370208134
SARS2	Diagnoses - main ICD10: K80.0 Calculus of gallbladder with acute cholecystitis	K80.0	1.000 (0.998, 1.002)	0.990731711
SARS2	Diagnoses - secondary ICD10: R19.4 Change in bowel habit	R19.4	1.003 (1.000, 1.006)	0.036330316
SARS2	Diagnoses - secondary ICD10: R10.1 Pain localised to upper abdomen	R10.1	0.997 (0.995, 0.999)	0.012175286
SARS2	Diagnoses - secondary ICD10: Z03.8 Observation for other suspected diseases and conditions	Z03.8	1.000 (0.997, 1.002)	0.966173461
SARS2	Diagnoses - secondary ICD10: I50.1 Left ventricular failure	I50.1	1.000 (0.998, 1.003)	0.752847418
SARS2	Diagnoses - main ICD10: D12.5 Sigmoid colon	D12.5	1.002 (0.999, 1.005)	0.150024165
SARS2	Diagnoses - main ICD10: Z09.9 Follow-up examination after unspecified treatment for other condition	Z09.9	1.000 (0.997, 1.002)	0.921314449
SARS2	Diagnoses - main ICD10: N47 Redundant prepuce, phimosis and paraphimosis	N47	1.002 (1.000, 1.004)	0.036781866
SARS2	Diagnoses - secondary ICD10: E10.9 Without complications	E10.9	0.999 (0.996, 1.001)	0.234134322
SARS2	Diagnoses - main ICD10: O70.1 Second degree perineal laceration during delivery	O70.1	1.000 (0.997, 1.003)	0.974233109
SARS2	Diagnoses - secondary ICD10: I20.0 Unstable angina	I20.0	0.999 (0.997, 1.001)	0.23009323
SARS2	Diagnoses - main ICD10: R07.4 Chest pain, unspecified	R07.4	1.002 (0.995, 1.009)	0.575755221
SARS2	Diagnoses - main ICD10: N93.9 Abnormal uterine and vaginal bleeding, unspecified	N93.9	1.001 (0.999, 1.002)	0.477549183
SARS2	Diagnoses - main ICD10: K57.3 Diverticular disease of large intestine without perforation or abscess	K57.3	1.004 (0.999, 1.009)	0.153887177
SARS2	Diagnoses - main ICD10: M54.59 Low back pain (Site unspecified)	M54.59	0.998 (0.996, 1.000)	0.072943426
SARS2	Diagnoses - secondary ICD10: Z87.4 Personal history of diseases of the genito-urinary system	Z87.4	0.998 (0.994, 1.002)	0.27071517
SARS2	Diagnoses - secondary ICD10: Z92.2 Personal history of long-term (current) use of other medicaments	Z92.2	1.000 (0.995, 1.005)	0.947067198
SARS2	Diagnoses - main ICD10: K80.2 Calculus of gallbladder without cholecystitis	K80.2	1.001 (0.997, 1.005)	0.531743494
SARS2	Diagnoses - secondary ICD10: M13.9 Arthritis, unspecified	M13.9	1.000 (0.998, 1.003)	0.650582337
SARS2	Diagnoses - main ICD10: R79.8 Other specified abnormal findings of blood chemistry	R79.8	0.999 (0.996, 1.001)	0.309289219
SARS2	Diagnoses - secondary ICD10: Z95.1 Presence of aortocoronary bypass graft	Z95.1	1.000 (0.997, 1.004)	0.80095187
SARS2	Diagnoses - secondary ICD10: W01.0 Home	W01.0	1.001 (0.999, 1.003)	0.308692889
SARS2	Diagnoses - secondary ICD10: Z85.8 Personal history of malignant neoplasms of other organs and syst	Z85.8	0.999 (0.996, 1.002)	0.436315112
SARS2	Diagnoses - main ICD10: I84.9 Unspecified haemorrhoids without complication	I84.9	0.997 (0.992, 1.001)	0.11180242

SARS2	Diagnoses - main ICD10: K60.2 Anal fissure, unspecified	K60.2	0.999 (0.998, 1.001)	0.568036285
SARS2	Diagnoses - main ICD10: N81.1 Cystocele	N81.1	1.000 (0.997, 1.003)	0.869102965
SARS2	Diagnoses - secondary ICD10: F41.9 Anxiety disorder, unspecified	F41.9	0.998 (0.996, 1.001)	0.13591726
SARS2	Diagnoses - main ICD10: K40.2 Bilateral inguinal hernia, without obstruction or gangrene	K40.2	1.001 (0.999, 1.003)	0.398827957
SARS2	Diagnoses - main ICD10: L90.5 Scar conditions and fibrosis of skin	L90.5	0.999 (0.997, 1.001)	0.402794569
SARS2	Diagnoses - main ICD10: R04.0 Epistaxis	R04.0	1.001 (0.999, 1.003)	0.244284601
SARS2	Diagnoses - secondary ICD10: Z51.1 Chemotherapy session for neoplasm	Z51.1	0.998 (0.994, 1.003)	0.50859651
SARS2	Diagnoses - main ICD10: R32 Unspecified urinary incontinence	R32	0.999 (0.998, 1.001)	0.377787663
SARS2	Diagnoses - main ICD10: N92.0 Excessive and frequent menstruation with regular cycle	N92.0	0.999 (0.995, 1.004)	0.806549835
SARS2	Diagnoses - main ICD10: K62.5 Haemorrhage of anus and rectum	K62.5	1.000 (0.995, 1.006)	0.899666161
SARS2	Diagnoses - secondary ICD10: Z96.1 Presence of intraocular lens	Z96.1	0.999 (0.996, 1.001)	0.255997383
SARS2	Diagnoses - secondary ICD10: R00.1 Bradycardia, unspecified	R00.1	1.001 (0.999, 1.003)	0.243334082
SARS2	Diagnoses - secondary ICD10: K66.0 Peritoneal adhesions	K66.0	1.002 (1.000, 1.004)	0.073281162
SARS2	Diagnoses - main ICD10: T84.8 Other complications of internal orthopaedic prosthetic devices, implan	T84.8	0.999 (0.997, 1.001)	0.175541125
SARS2	Diagnoses - secondary ICD10: Z85.4 Personal history of malignant neoplasm of genital organs	Z85.4	0.999 (0.996, 1.002)	0.472010397
SARS2	Diagnoses - main ICD10: I47.1 Supraventricular tachycardia	I47.1	1.001 (0.999, 1.003)	0.515043739
SARS2	Diagnoses - secondary ICD10: F10.0 Acute intoxication	F10.0	1.000 (0.998, 1.002)	0.75165625
SARS2	Diagnoses - main ICD10: K08.3 Retained dental root	K08.3	1.000 (0.998, 1.002)	0.802402463
SARS2	Diagnoses - secondary ICD10: M06.99 Rheumatoid arthritis, unspecified (Site unspecified)	M06.99	1.000 (0.998, 1.002)	0.857688287
SARS2	Diagnoses - main ICD10: L03.1 Cellulitis of other parts of limb	L03.1	1.000 (0.997, 1.003)	0.942826098
SARS2	Diagnoses - secondary ICD10: R53 Malaise and fatigue	R53	1.000 (0.998, 1.002)	0.725930383
SARS2	Diagnoses - secondary ICD10: W01.9 Unspecified place	W01.9	1.001 (0.999, 1.003)	0.394247989
SARS2	Diagnoses - main ICD10: C50.4 Upper-outer quadrant of breast	C50.4	1.002 (1.000, 1.004)	0.04683122
SARS2	Diagnoses - main ICD10: T39.1 4-Aminophenol derivatives	T39.1	0.999 (0.998, 1.001)	0.3788673
SARS2	Diagnoses - secondary ICD10: K29.5 Chronic gastritis, unspecified	K29.5	1.000 (0.999, 1.002)	0.745541469
SARS2	Diagnoses - secondary ICD10: G55.1 Nerve root and plexus compressions in intervertebral disk disord	G55.1	0.999 (0.996, 1.002)	0.437208874
SARS2	Diagnoses - secondary ICD10: K25.9 Unspecified as acute or chronic, without haemorrhage or perfora	K25.9	1.002 (1.000, 1.004)	0.066795558
SARS2	Diagnoses - main ICD10: I80.2 Phlebitis and thrombophlebitis of other deep vessels of lower extremiti	I80.2	1.001 (0.998, 1.003)	0.532474002
SARS2	Diagnoses - secondary ICD10: I84.6 Residual haemorrhoidal skin tags	I84.6	1.000 (0.998, 1.002)	0.99796785
SARS2	Diagnoses - main ICD10: N32.0 Bladder-neck obstruction	N32.0	1.001 (0.999, 1.003)	0.549912635
SARS2	Diagnoses - main ICD10: K29.8 Duodenitis	K29.8	0.999 (0.997, 1.002)	0.62740457
SARS2	Diagnoses - main ICD10: M67.4 Ganglion	M67.4	1.001 (0.998, 1.003)	0.5338501
SARS2	Diagnoses - main ICD10: H26.9 Cataract, unspecified	H26.9	1.004 (0.999, 1.009)	0.119109216
SARS2	Type of cancer: ICD10: C20 Malignant neoplasm of rectum	C20	1.001 (0.999, 1.003)	0.3552038
SARS2	Diagnoses - secondary ICD10: K30 Dyspepsia	K30	1.001 (0.998, 1.003)	0.545741219
SARS2	Diagnoses - secondary ICD10: I84.2 Internal haemorrhoids without complication	I84.2	1.001 (0.999, 1.003)	0.368387775
SARS2	Diagnoses - secondary ICD10: N94.6 Dysmenorrhoea, unspecified	N94.6	1.002 (1.000, 1.003)	0.094739625

SARS2	Diagnoses - main ICD10: Z08.0 Follow-up examination after surgery for malignant neoplasm	Z08.0	1.001 (0.998, 1.004)	0.488249091
SARS2	Diagnoses - main ICD10: M79.86 Other specified soft tissue disorders (Lower leg)	M79.86	1.001 (0.999, 1.003)	0.292947662
SARS2	Diagnoses - secondary ICD10: E78.0 Pure hypercholesterolaemia	E78.0	1.004 (0.996, 1.012)	0.275525444
SARS2	Diagnoses - secondary ICD10: M10.99 Gout, unspecified (Site unspecified)	M10.99	1.001 (0.999, 1.002)	0.416936144
SARS2	Diagnoses - main ICD10: M51.2 Other specified intervertebral disk displacement	M51.2	1.000 (0.998, 1.002)	0.961557849
SARS2	Diagnoses - main ICD10: N84.0 Polyp of corpus uteri	N84.0	1.001 (0.998, 1.005)	0.472555155
SARS2	Diagnoses - secondary ICD10: M17.1 Other primary gonarthrosis	M17.1	0.999 (0.997, 1.001)	0.53023187
SARS2	Diagnoses - main ICD10: M25.5 Pain in joint	M25.5	1.000 (0.998, 1.002)	0.904434288
SARS2	Diagnoses - main ICD10: K29.6 Other gastritis	K29.6	1.003 (1.000, 1.005)	0.017586257
SARS2	Type of cancer: ICD10: C64 Malignant neoplasm of kidney, except renal pelvis	C64	1.001 (0.999, 1.002)	0.479920643
SARS2	Diagnoses - main ICD10: G43.9 Migraine, unspecified	G43.9	1.000 (0.998, 1.002)	0.997081781
SARS2	Diagnoses - secondary ICD10: Z80.0 Family history of malignant neoplasm of digestive organs	Z80.0	0.998 (0.994, 1.003)	0.460120997
SARS2	Diagnoses - main ICD10: G45.9 Transient cerebral ischaemic attack, unspecified	G45.9	1.000 (0.998, 1.002)	0.699358368
SARS2	Diagnoses - main ICD10: O02.1 Missed abortion	O02.1	1.001 (0.999, 1.003)	0.314306866
SARS2	Diagnoses - main ICD10: K62.8 Other specified diseases of anus and rectum	K62.8	1.001 (0.999, 1.003)	0.16477881
SARS2	Diagnoses - main ICD10: M72.0 Palmar fascial fibromatosis [Dupuytren]	M72.0	1.002 (1.000, 1.004)	0.033032157
SARS2	Diagnoses - secondary ICD10: I34.0 Mitral (valve) insufficiency	I34.0	1.000 (0.999, 1.002)	0.725939232
SARS2	Diagnoses - main ICD10: O04.9 Complete or unspecified, without complication	O04.9	1.000 (0.998, 1.001)	0.633794439
SARS2	Diagnoses - main ICD10: K22.1 Ulcer of oesophagus	K22.1	1.003 (1.000, 1.006)	0.043003016
SARS2	Diagnoses - secondary ICD10: Z86.7 Personal history of diseases of the circulatory system	Z86.7	1.004 (0.998, 1.009)	0.186141693
SARS2	Diagnoses - secondary ICD10: Y83.1 Surgical operation with implant of artificial internal device	Y83.1	0.998 (0.995, 1.002)	0.392134868
SARS2	Diagnoses - secondary ICD10: R63.4 Abnormal weight loss	R63.4	1.000 (0.997, 1.003)	0.853032225
SARS2	Diagnoses - main ICD10: K21.9 Gastro-oesophageal reflux disease without oesophagitis	K21.9	1.001 (0.997, 1.005)	0.545177358
SARS2	Diagnoses - main ICD10: N93.8 Other specified abnormal uterine and vaginal bleeding	N93.8	1.000 (0.998, 1.002)	0.724507771
SARS2	Diagnoses - main ICD10: M75.4 Impingement syndrome of shoulder	M75.4	1.001 (0.998, 1.004)	0.56857164
SARS2	Diagnoses - main ICD10: I20.9 Angina pectoris, unspecified	I20.9	0.999 (0.996, 1.003)	0.6720676
SARS2	Diagnoses - secondary ICD10: X59.9 Unspecified place	X59.9	1.001 (0.998, 1.004)	0.588760748
SARS2	Diagnoses - main ICD10: I84.1 Internal haemorrhoids with other complications	I84.1	0.999 (0.996, 1.001)	0.200000048
SARS2	Diagnoses - secondary ICD10: K57.9 Diverticular disease of intestine, part unspecified, without perfora	K57.9	1.000 (0.997, 1.003)	0.869327117
SARS2	Diagnoses - main ICD10: L72.9 Follicular cyst of skin and subcutaneous tissue, unspecified	L72.9	1.002 (1.001, 1.004)	0.008874936
SARS2	Diagnoses - secondary ICD10: Z92.3 Personal history of irradiation	Z92.3	1.001 (0.999, 1.003)	0.434530038
SARS2	Diagnoses - main ICD10: Z09.8 Follow-up examination after other treatment for other conditions	Z09.8	1.002 (1.000, 1.005)	0.095934261
SARS2	Diagnoses - main ICD10: K42.9 Umbilical hernia without obstruction or gangrene	K42.9	0.999 (0.996, 1.001)	0.270692285
SARS2	Diagnoses - main ICD10: R07.3 Other chest pain	R07.3	1.003 (0.998, 1.007)	0.247564442
SARS2	Diagnoses - main ICD10: N92.6 Irregular menstruation, unspecified	N92.6	0.999 (0.997, 1.001)	0.214842158
SARS2	Diagnoses - main ICD10: T84.0 Mechanical complication of internal joint prosthesis	T84.0	1.001 (0.999, 1.003)	0.388919222
SARS2	Diagnoses - main ICD10: D64.9 Anaemia, unspecified	D64.9	0.999 (0.995, 1.002)	0.423618239

SARS2	Diagnoses - secondary ICD10: R00.2 Palpitations	R00.2	1.001 (0.999, 1.003)	0.474461463
SARS2	Diagnoses - secondary ICD10: Z90.4 Acquired absence of other parts of digestive tract	Z90.4	0.999 (0.996, 1.002)	0.563657593
SARS2	Type of cancer: ICD10: C54.1 Endometrium	C54.1	1.000 (0.998, 1.002)	0.952103132
SARS2	Diagnoses - main ICD10: K92.2 Gastro-intestinal haemorrhage, unspecified	K92.2	1.000 (0.998, 1.002)	0.847291402
SARS2	Diagnoses - main ICD10: L72.0 Epidermal cyst	L72.0	1.002 (1.000, 1.004)	0.091908267
SARS2	Diagnoses - secondary ICD10: M13.99 Arthritis, unspecified (Site unspecified)	M13.99	1.001 (0.998, 1.004)	0.600697573
SARS2	Diagnoses - secondary ICD10: Z95.8 Presence of other cardiac and vascular implants and grafts	Z95.8	0.999 (0.997, 1.001)	0.200922491
SARS2	Diagnoses - secondary ICD10: K29.7 Gastritis, unspecified	K29.7	1.003 (0.999, 1.007)	0.194947058
SARS2	Diagnoses - secondary ICD10: E66.9 Obesity, unspecified	E66.9	0.998 (0.994, 1.002)	0.292592093
SARS2	Diagnoses - main ICD10: M54.5 Low back pain	M54.5	0.998 (0.995, 1.000)	0.104396813
SARS2	Diagnoses - main ICD10: J18.9 Pneumonia, unspecified	J18.9	1.000 (0.998, 1.002)	0.78276864
SARS2	Type of cancer: ICD10: C44.5 Skin of trunk	C44.5	1.001 (0.998, 1.003)	0.652201147
SARS2	Diagnoses - secondary ICD10: Z95.5 Presence of coronary angioplasty implant and graft	Z95.5	1.002 (0.999, 1.005)	0.21282698
SARS2	Type of cancer: ICD10: C18.7 Sigmoid colon	C18.7	1.000 (0.998, 1.002)	0.840498536
SARS2	Diagnoses - main ICD10: R87.6 Abnormal cytological findings	R87.6	1.000 (0.998, 1.002)	0.774421982
SARS2	Diagnoses - secondary ICD10: Z86.4 Personal history of psychoactive substance abuse	Z86.4	0.999 (0.993, 1.005)	0.703864325
SARS2	Diagnoses - secondary ICD10: Z51.5 Palliative care	Z51.5	0.999 (0.997, 1.001)	0.198234437
SARS2	Diagnoses - secondary ICD10: G40.9 Epilepsy, unspecified	G40.9	0.999 (0.997, 1.002)	0.517815277
SARS2	Diagnoses - secondary ICD10: Z51.0 Radiotherapy session	Z51.0	1.002 (0.999, 1.004)	0.167891314
SARS2	Diagnoses - secondary ICD10: F10.1 Harmful use	F10.1	1.002 (0.999, 1.004)	0.161855938
SARS2	Diagnoses - secondary ICD10: I51.7 Cardiomegaly	I51.7	1.001 (0.999, 1.003)	0.188389623
SARS2	Diagnoses - main ICD10: N23 Unspecified renal colic	N23	0.998 (0.995, 1.000)	0.034000148
SARS2	Diagnoses - secondary ICD10: E86 Volume depletion	E86	1.001 (0.999, 1.003)	0.211617865
SARS2	Diagnoses - secondary ICD10: Z87.1 Personal history of diseases of the digestive system	Z87.1	1.002 (0.997, 1.008)	0.377123057
SARS2	Diagnoses - secondary ICD10: Z85.0 Personal history of malignant neoplasm of digestive organs	Z85.0	0.999 (0.996, 1.003)	0.690236958
SARS2	Diagnoses - secondary ICD10: R03.0 Elevated blood-pressure reading, without diagnosis of hypertensi	R03.0	0.999 (0.997, 1.001)	0.32888456
SARS2	Diagnoses - secondary ICD10: I25.8 Other forms of chronic ischaemic heart disease	I25.8	1.001 (0.997, 1.005)	0.60211646
SARS2	Diagnoses - secondary ICD10: Z90.1 Acquired absence of breast(s)	Z90.1	1.001 (0.999, 1.003)	0.306627717
SARS2	Diagnoses - secondary ICD10: I25.2 Old myocardial infarction	I25.2	1.000 (0.997, 1.003)	0.894896417
SARS2	Diagnoses - main ICD10: I25.1 Atherosclerotic heart disease	I25.1	0.996 (0.991, 1.002)	0.233702929
SARS2	Diagnoses - secondary ICD10: Z91.0 Personal history of allergy, other than to drugs and biological sub	Z91.0	0.999 (0.997, 1.001)	0.495017963
SARS2	Diagnoses - secondary ICD10: C78.7 Secondary malignant neoplasm of liver	C78.7	0.998 (0.996, 1.000)	0.061045698
SARS2	Diagnoses - secondary ICD10: J44.9 Chronic obstructive pulmonary disease, unspecified	J44.9	1.000 (0.997, 1.003)	0.97419132
SARS2	Diagnoses - main ICD10: G47.3 Sleep apnoea	G47.3	0.997 (0.995, 1.000)	0.04512519
SARS2	Diagnoses - secondary ICD10: N95.0 Postmenopausal bleeding	N95.0	1.000 (0.998, 1.003)	0.822541169
SARS2	Diagnoses - main ICD10: S09.9 Unspecified injury of head	S09.9	1.000 (0.998, 1.002)	0.864750849
SARS2	Diagnoses - secondary ICD10: Z95.0 Presence of cardiac pacemaker	Z95.0	1.001 (0.999, 1.003)	0.546084476

SARS2	Diagnoses - secondary ICD10: C77.3 Axillary and upper limb lymph nodes	C77.3	1.001 (0.999, 1.003)	0.490395414
SARS2	Type of cancer: ICD10: C44.4 Skin of scalp and neck	C44.4	1.000 (0.998, 1.002)	0.844489093
SARS2	Diagnoses - secondary ICD10: J45.9 Asthma, unspecified	J45.9	0.997 (0.990, 1.004)	0.386899117
SARS2	Diagnoses - main ICD10: R39.8 Other and unspecified symptoms and signs involving the urinary syste	R39.8	1.001 (0.999, 1.003)	0.362440681
SARS2	Diagnoses - secondary ICD10: R00.0 Tachycardia, unspecified	R00.0	1.002 (1.000, 1.003)	0.050793552
SARS2	Diagnoses - main ICD10: R10.3 Pain localised to other parts of lower abdomen	R10.3	0.998 (0.995, 1.002)	0.414622778
SARS2	Diagnoses - secondary ICD10: E78.5 Hyperlipidaemia, unspecified	E78.5	1.001 (0.998, 1.004)	0.47714596
SARS2	Diagnoses - secondary ICD10: E14.9 Without complications	E14.9	0.999 (0.997, 1.001)	0.403286464
SARS2	Diagnoses - main ICD10: T81.0 Haemorrhage and haematoma complicating a procedure, not elsewhere	T81.0	1.000 (0.997, 1.002)	0.855241151
SARS2	Diagnoses - secondary ICD10: J18.1 Lobar pneumonia, unspecified	J18.1	1.000 (0.998, 1.002)	0.784297084
SARS2	Diagnoses - secondary ICD10: M81.99 Osteoporosis, unspecified (Site unspecified)	M81.99	1.001 (0.998, 1.003)	0.547844305
SARS2	Diagnoses - secondary ICD10: Y83.8 Other surgical procedures	Y83.8	1.001 (0.997, 1.005)	0.575279234
SARS2	Diagnoses - secondary ICD10: K63.5 Polyp of colon	K63.5	0.999 (0.996, 1.002)	0.511866885
SARS2	Diagnoses - main ICD10: K58.9 Irritable bowel syndrome without diarrhoea	K58.9	1.000 (0.999, 1.002)	0.692909133
SARS2	Diagnoses - secondary ICD10: K59.0 Constipation	K59.0	0.999 (0.996, 1.002)	0.571861791
SARS2	Diagnoses - secondary ICD10: Z87.3 Personal history of diseases of the musculoskeletal system and co	Z87.3	0.999 (0.997, 1.000)	0.136799895
SARS2	Type of cancer: ICD10: C56 Malignant neoplasm of ovary	C56	1.000 (0.998, 1.002)	0.887179918
SARS2	Diagnoses - main ICD10: M51.1 Lumbar and other intervertebral disk disorders with radiculopathy	M51.1	0.999 (0.997, 1.002)	0.606520736
SARS2	Diagnoses - main ICD10: I26.9 Pulmonary embolism without mention of acute cor pulmonale	I26.9	1.001 (0.999, 1.004)	0.270285822
SARS2	Diagnoses - main ICD10: N20.0 Calculus of kidney	N20.0	0.999 (0.997, 1.002)	0.567703871
SARS2	Diagnoses - main ICD10: M17.9 Gonarthrosis, unspecified	M17.9	0.998 (0.994, 1.003)	0.474787871
SARS2	Diagnoses - main ICD10: K92.0 Haematemesis	K92.0	1.000 (0.998, 1.002)	0.896379503
SARS2	Diagnoses - main ICD10: N20.1 Calculus of ureter	N20.1	0.999 (0.996, 1.001)	0.325583073
SARS2	Diagnoses - main ICD10: K13.7 Other and unspecified lesions of oral mucosa	K13.7	0.998 (0.996, 0.999)	0.011102131
SARS2	Type of cancer: ICD10: C34.1 Upper lobe, bronchus or lung	C34.1	1.000 (0.998, 1.002)	0.93117159
SARS2	Diagnoses - main ICD10: Z42.1 Follow-up care involving plastic surgery of breast	Z42.1	1.000 (0.998, 1.002)	0.877806302
SARS2	Type of cancer: ICD10: C50.9 Breast, unspecified	C50.9	0.996 (0.992, 1.000)	0.082172623
SARS2	Diagnoses - main ICD10: J34.8 Other specified disorders of nose and nasal sinuses	J34.8	0.999 (0.997, 1.001)	0.354387453
SARS2	Type of cancer: ICD10: C44.6 Skin of upper limb, including shoulder	C44.6	1.001 (0.999, 1.003)	0.605352506
SARS2	Diagnoses - secondary ICD10: R50.9 Fever, unspecified	R50.9	0.999 (0.996, 1.001)	0.267731096
SARS2	Diagnoses - secondary ICD10: W10.0 Home	W10.0	0.999 (0.997, 1.001)	0.159672017
SARS2	Diagnoses - main ICD10: N84.1 Polyp of cervix uteri	N84.1	1.000 (0.998, 1.003)	0.811092949
SARS2	Diagnoses - secondary ICD10: R33 Retention of urine	R33	0.999 (0.996, 1.002)	0.39650353
SARS2	Diagnoses - main ICD10: D12.6 Colon, unspecified	D12.6	1.000 (0.998, 1.003)	0.734213434
SARS2	Diagnoses - main ICD10: I63.9 Cerebral infarction, unspecified	I63.9	1.000 (0.998, 1.002)	0.671344331
SARS2	Diagnoses - main ICD10: K51.9 Ulcerative colitis, unspecified	K51.9	0.997 (0.995, 1.000)	0.04075591
SARS2	Diagnoses - main ICD10: K85 Acute pancreatitis	K85	0.999 (0.997, 1.001)	0.448599379

SARS2	Diagnoses - secondary ICD10: R06.0 Dyspnoea	R06.0	1.000 (0.997, 1.003)	0.971936454
SARS2	Diagnoses - secondary ICD10: R10.4 Other and unspecified abdominal pain	R10.4	1.000 (0.996, 1.004)	0.927734182
SARS2	Diagnoses - main ICD10: Z47.0 Follow-up care involving removal of fracture plate and other internal	Z47.0	1.000 (0.997, 1.003)	0.903518383
SARS2	Diagnoses - secondary ICD10: F10.2 Dependence syndrome	F10.2	1.001 (0.999, 1.003)	0.380006149
SARS2	Diagnoses - main ICD10: R06.5 Mouth breathing	R06.5	1.001 (0.999, 1.003)	0.30020126
SARS2	Diagnoses - main ICD10: R51 Headache	R51	1.000 (0.996, 1.003)	0.91484523
SARS2	Diagnoses - main ICD10: K62.1 Rectal polyp	K62.1	0.999 (0.996, 1.002)	0.545260108
SARS2	Diagnoses - main ICD10: M51.3 Other specified intervertebral disk degeneration	M51.3	1.001 (0.999, 1.003)	0.170518278
SARS2	Diagnoses - main ICD10: M54.56 Low back pain (Lumbar region)	M54.56	0.999 (0.997, 1.001)	0.305804389
SARS2	Diagnoses - main ICD10: M16.9 Coxarthrosis, unspecified	M16.9	1.003 (1.000, 1.007)	0.08095526
SARS2	Diagnoses - secondary ICD10: Z86.1 Personal history of infectious and parasitic diseases	Z86.1	1.001 (0.999, 1.003)	0.188652122
SARS2	Diagnoses - secondary ICD10: I12.0 Hypertensive renal disease with renal failure	I12.0	0.999 (0.997, 1.001)	0.314444417
SARS2	Diagnoses - secondary ICD10: R05 Cough	R05	1.001 (0.999, 1.003)	0.169157129
SARS2	Diagnoses - main ICD10: Z13.8 Special screening examination for other specified diseases and disorde	Z13.8	1.002 (1.000, 1.003)	0.110983936
SARS2	Diagnoses - secondary ICD10: F32.9 Depressive episode, unspecified	F32.9	0.997 (0.993, 1.002)	0.215931901
SARS2	Diagnoses - secondary ICD10: Z85.3 Personal history of malignant neoplasm of breast	Z85.3	0.998 (0.994, 1.002)	0.38570828
SARS2	Diagnoses - main ICD10: K52.9 Non-infective gastro-enteritis and colitis, unspecified	K52.9	1.000 (0.995, 1.005)	0.989467834
SARS2	Diagnoses - main ICD10: L98.9 Disorder of skin and subcutaneous tissue, unspecified	L98.9	1.000 (0.997, 1.003)	0.810085207
SARS2	Diagnoses - secondary ICD10: M19.99 Arthrosis, unspecified (Site unspecified)	M19.99	1.000 (0.997, 1.003)	0.874359613
SARS2	Diagnoses - main ICD10: Z12.1 Special screening examination for neoplasm of intestinal tract	Z12.1	0.997 (0.994, 1.000)	0.082765897
SARS2	Diagnoses - main ICD10: R07.2 Precordial pain	R07.2	0.999 (0.996, 1.001)	0.343266499
SARS2	Diagnoses - secondary ICD10: Z90.7 Acquired absence of genital organ(s)	Z90.7	0.998 (0.995, 1.001)	0.281883248
SARS2	Diagnoses - secondary ICD10: Z96.6 Presence of orthopaedic joint implants	Z96.6	0.998 (0.994, 1.002)	0.402423911
SARS2	Diagnoses - secondary ICD10: B96.8 Other specified bacterial agents as the cause of diseases classified	B96.8	1.006 (1.002, 1.009)	0.001824704
SARS2	Diagnoses - secondary ICD10: R42 Dizziness and giddiness	R42	1.002 (0.999, 1.004)	0.1734
SARS2	Diagnoses - secondary ICD10: N81.2 Incomplete uterovaginal prolapse	N81.2	1.002 (1.000, 1.004)	0.031615532
SARS2	Diagnoses - main ICD10: M25.56 Pain in joint (Lower leg)	M25.56	1.000 (0.997, 1.002)	0.726737443
SARS2	Diagnoses - main ICD10: D17.2 Benign lipomatous neoplasm of skin and subcutaneous tissue of limbs	D17.2	1.001 (0.999, 1.003)	0.548716235
SARS2	Diagnoses - main ICD10: O63.1 Prolonged second stage (of labour)	O63.1	1.001 (0.999, 1.002)	0.547412754
SARS2	Diagnoses - secondary ICD10: Z82.4 Family history of ischaemic heart disease and other diseases of th	Z82.4	0.999 (0.994, 1.004)	0.615304789
SARS2	Diagnoses - main ICD10: H25.0 Senile incipient cataract	H25.0	1.001 (0.999, 1.003)	0.224386569
SARS2	Diagnoses - main ICD10: D17.1 Benign lipomatous neoplasm of skin and subcutaneous tissue of trunk	D17.1	0.999 (0.997, 1.001)	0.434846561
SARS2	Diagnoses - secondary ICD10: M47.82 Other spondylosis (Cervical region)	M47.82	0.999 (0.998, 1.001)	0.416025381
SARS2	Diagnoses - secondary ICD10: Z98.0 Intestinal bypass and anastomosis status	Z98.0	0.999 (0.997, 1.001)	0.450592298
SARS2	Type of cancer: ICD10: C43.7 Malignant melanoma of lower limb, including hip	C43.7	0.999 (0.997, 1.001)	0.216834047
SARS2	Diagnoses - main ICD10: S52.50 Fracture of lower end of radius (closed)	S52.50	1.001 (0.998, 1.004)	0.47175399
SARS2	Diagnoses - secondary ICD10: W19.9 Unspecified place	W19.9	1.002 (1.000, 1.005)	0.0975021

SARS2	Diagnoses - main ICD10: M16.1 Other primary coxarthrosis	M16.1	0.998 (0.995, 1.001)	0.146011767
SARS2	Diagnoses - secondary ICD10: Z60.2 Living alone	Z60.2	0.998 (0.995, 1.001)	0.249517872
SARS2	Diagnoses - secondary ICD10: F17.1 Harmful use	F17.1	1.000 (0.998, 1.003)	0.657851242
SARS2	Diagnoses - secondary ICD10: K31.8 Other specified diseases of stomach and duodenum	K31.8	1.003 (1.001, 1.005)	0.006176088
SARS2	Diagnoses - secondary ICD10: K31.7 Polyp of stomach and duodenum	K31.7	1.001 (0.999, 1.003)	0.240776456
SARS2	Diagnoses - main ICD10: H25.1 Senile nuclear cataract	H25.1	0.999 (0.996, 1.001)	0.242532793
SARS2	Diagnoses - secondary ICD10: Y83.2 Surgical operation with anastomosis, bypass or graft	Y83.2	1.001 (0.999, 1.003)	0.590789215
SARS2	Diagnoses - secondary ICD10: D70 Agranulocytosis	D70	1.001 (0.999, 1.003)	0.341936669
SARS2	Diagnoses - main ICD10: D50.9 Iron deficiency anaemia, unspecified	D50.9	1.001 (0.998, 1.003)	0.487155724
SARS2	Diagnoses - secondary ICD10: N81.6 Rectocele	N81.6	0.999 (0.996, 1.001)	0.390589477
SARS2	Diagnoses - main ICD10: I21.9 Acute myocardial infarction, unspecified	I21.9	1.000 (0.998, 1.003)	0.791896178
SARS2	Diagnoses - main ICD10: D22.3 Melanocytic naevi of other and unspecified parts of face	D22.3	1.000 (0.998, 1.002)	0.898263688
SARS2	Diagnoses - secondary ICD10: K44.9 Diaphragmatic hernia without obstruction or gangrene	K44.9	1.002 (0.996, 1.009)	0.499124881
SARS2	Diagnoses - main ICD10: B34.9 Viral infection, unspecified	B34.9	1.001 (0.999, 1.002)	0.422801751
SARS2	Diagnoses - main ICD10: N32.8 Other specified disorders of bladder	N32.8	0.999 (0.997, 1.002)	0.592552707
SARS2	Diagnoses - main ICD10: J34.2 Deviated nasal septum	J34.2	0.998 (0.995, 1.001)	0.197440575
SARS2	Diagnoses - main ICD10: N50.8 Other specified disorders of male genital organs	N50.8	1.000 (0.998, 1.002)	0.843526008
SARS2	Diagnoses - secondary ICD10: Z86.0 Personal history of other neoplasms	Z86.0	0.998 (0.995, 1.002)	0.329248599
SARS2	Diagnoses - secondary ICD10: Z72.1 Alcohol use	Z72.1	0.999 (0.996, 1.003)	0.753610629
SARS2	Diagnoses - main ICD10: G56.0 Carpal tunnel syndrome	G56.0	1.003 (0.998, 1.008)	0.201479081
SARS2	Diagnoses - main ICD10: I25.9 Chronic ischaemic heart disease, unspecified	I25.9	1.000 (0.998, 1.002)	0.791265969
SARS2	Diagnoses - secondary ICD10: Z37.0 Single live birth	Z37.0	0.993 (0.987, 0.999)	0.014408108
SARS2	Diagnoses - secondary ICD10: E03.9 Hypothyroidism, unspecified	E03.9	0.995 (0.990, 1.000)	0.074351781
SARS2	Diagnoses - secondary ICD10: Z88.6 Personal history of allergy to analgesic agent	Z88.6	0.998 (0.995, 1.001)	0.117990128
SARS2	Diagnoses - main ICD10: M20.2 Hallux rigidus	M20.2	1.000 (0.998, 1.002)	0.931801751
SARS2	Diagnoses - secondary ICD10: B95.6 Staphylococcus aureus as the cause of diseases classified to other	B95.6	0.999 (0.997, 1.002)	0.632625149
SARS2	Diagnoses - main ICD10: R04.2 Haemoptysis	R04.2	1.001 (0.999, 1.002)	0.569278935
SARS2	Diagnoses - main ICD10: I21.0 Acute transmural myocardial infarction of anterior wall	I21.0	1.001 (0.999, 1.003)	0.179202002
SARS2	Diagnoses - secondary ICD10: R94.5 Abnormal results of liver function studies	R94.5	1.003 (1.001, 1.005)	0.014556262
SARS2	Diagnoses - secondary ICD10: T81.4 Infection following a procedure, not elsewhere classified	T81.4	1.001 (0.999, 1.003)	0.535166577
SARS2	Diagnoses - secondary ICD10: Z88.8 Personal history of allergy to other drugs, medicaments and biolo	Z88.8	0.998 (0.995, 1.001)	0.141856158
SARS2	Diagnoses - main ICD10: H00.1 Chalazion	H00.1	1.002 (1.000, 1.004)	0.075319516
SARS2	Type of cancer: ICD10: D05.1 Intraductal carcinoma in situ	D05.1	1.000 (0.998, 1.002)	0.913193042
SARS2	Diagnoses - secondary ICD10: Y43.3 Other antineoplastic drugs	Y43.3	1.000 (0.998, 1.001)	0.659527378
SARS2	Diagnoses - main ICD10: K02.9 Dental caries, unspecified	K02.9	0.999 (0.997, 1.002)	0.545835733
SARS2	Diagnoses - main ICD10: M72.04 Palmar fascial fibromatosis [Dupuytren]-Hand	M72.04	0.999 (0.997, 1.002)	0.595100144
SARS2	Diagnoses - secondary ICD10: N83.2 Other and unspecified ovarian cysts	N83.2	1.001 (0.999, 1.003)	0.401135637

SARS2	Diagnoses - main ICD10: M75.0 Adhesive capsulitis of shoulder	M75.0	1.000 (0.999, 1.002)	0.734437515
SARS2	Diagnoses - secondary ICD10: I73.9 Peripheral vascular disease, unspecified	I73.9	1.000 (0.997, 1.002)	0.645380422
SARS2	Diagnoses - secondary ICD10: N17.9 Acute renal failure, unspecified	N17.9	1.001 (0.999, 1.003)	0.578275409
SARS2	Diagnoses - main ICD10: M75.1 Rotator cuff syndrome	M75.1	0.999 (0.997, 1.001)	0.328007796
SARS2	Diagnoses - main ICD10: L82 Seborrhoeic keratosis	L82	0.998 (0.995, 1.000)	0.053249633
SARS2	Diagnoses - main ICD10: K61.0 Anal abscess	K61.0	0.999 (0.997, 1.001)	0.344615245
SARS2	Diagnoses - secondary ICD10: Z53.2 Procedure not carried out because of patient's decision for other a	Z53.2	1.000 (0.998, 1.002)	0.794778819
SARS2	Diagnoses - secondary ICD10: Z72.0 Tobacco use	Z72.0	1.000 (0.994, 1.006)	0.924686737
SARS2	Diagnoses - main ICD10: I21.1 Acute transmural myocardial infarction of inferior wall	I21.1	1.000 (0.997, 1.002)	0.774562625
SARS2	Diagnoses - secondary ICD10: N39.3 Stress incontinence	N39.3	1.000 (0.998, 1.002)	0.825044776
SARS2	Diagnoses - main ICD10: K40.9 Unilateral or unspecified inguinal hernia, without obstruction or gangr	K40.9	1.002 (0.996, 1.008)	0.494023836
SARS2	Diagnoses - main ICD10: C67.9 Bladder, unspecified	C67.9	1.000 (0.998, 1.002)	0.987356546
SARS2	Diagnoses - secondary ICD10: Z13.0 Special screening examination for diseases of the blood and blood	Z13.0	0.999 (0.998, 1.001)	0.427488881
SARS2	Diagnoses - secondary ICD10: Z86.6 Personal history of diseases of the nervous system and sense orga	Z86.6	0.999 (0.996, 1.002)	0.500647231
SARS2	Diagnoses - main ICD10: K92.1 Melaena	K92.1	1.001 (0.999, 1.003)	0.335960586
SARS2	Diagnoses - secondary ICD10: J34.3 Hypertrophy of nasal turbinates	J34.3	0.996 (0.995, 0.998)	0.000257483
SARS2	Diagnoses - secondary ICD10: Z88.0 Personal history of allergy to penicillin	Z88.0	0.998 (0.992, 1.004)	0.502515202
SARS2	Diagnoses - main ICD10: S82.80 Fractures of other parts of lower leg (closed)	S82.80	1.001 (0.999, 1.003)	0.491303576
SARS2	Diagnoses - main ICD10: M20.1 Hallux valgus (acquired)	M20.1	1.001 (0.997, 1.005)	0.689454507
SARS2	Diagnoses - secondary ICD10: N39.0 Urinary tract infection, site not specified	N39.0	1.001 (0.998, 1.005)	0.475870833
SARS2	Diagnoses - secondary ICD10: Z03.4 Observation for suspected myocardial infarction	Z03.4	0.997 (0.994, 1.000)	0.027875023
SARS2	Diagnoses - main ICD10: O80.0 Spontaneous vertex delivery	O80.0	0.997 (0.995, 1.000)	0.022697482
SARS2	Diagnoses - secondary ICD10: N73.6 Female pelvic peritoneal adhesions	N73.6	1.000 (0.998, 1.002)	0.975168778
SARS2	Diagnoses - secondary ICD10: D25.9 Leiomyoma of uterus, unspecified	D25.9	0.999 (0.996, 1.002)	0.457580079
SARS2	Diagnoses - secondary ICD10: K26.9 Unspecified as acute or chronic, without haemorrhage or perfora	K26.9	1.001 (0.999, 1.003)	0.215006129
SARS2	Diagnoses - main ICD10: K60.3 Anal fistula	K60.3	1.001 (0.999, 1.002)	0.501827982
SARS2	Diagnoses - main ICD10: K43.9 Ventral hernia without obstruction or gangrene	K43.9	1.004 (1.001, 1.006)	0.003744021
SARS2	Diagnoses - main ICD10: K35.9 Acute appendicitis, unspecified	K35.9	1.001 (0.999, 1.003)	0.507660198
SARS2	Type of cancer: ICD10: C44.7 Skin of lower limb, including hip	C44.7	1.000 (0.998, 1.002)	0.998530316
SARS2	Diagnoses - secondary ICD10: M15.9 Polyarthrosis, unspecified	M15.9	1.000 (0.998, 1.002)	0.978646535
SARS2	Diagnoses - main ICD10: R13 Dysphagia	R13	1.001 (0.998, 1.004)	0.50866662
SARS2	Diagnoses - main ICD10: M23.23 Derangement of meniscus due to old tear or injury (Medial collateral	M23.23	0.998 (0.996, 1.001)	0.231648383
SARS2	Diagnoses - secondary ICD10: Z51.2 Other chemotherapy	Z51.2	0.999 (0.996, 1.002)	0.69544184
SARS2	Diagnoses - main ICD10: J33.9 Nasal polyp, unspecified	J33.9	1.000 (0.998, 1.002)	0.935307324
SARS2	Diagnoses - secondary ICD10: Z85.5 Personal history of malignant neoplasm of urinary tract	Z85.5	1.000 (0.998, 1.003)	0.699556458
SARS2	Diagnoses - secondary ICD10: Z92.1 Personal history of long-term (current) use of anticoagulants	Z92.1	0.998 (0.995, 1.002)	0.45025395
SARS2	Diagnoses - main ICD10: Z30.2 Sterilisation	Z30.2	0.999 (0.994, 1.003)	0.579708902

SARS2	Diagnoses - main ICD10: N63 Unspecified lump in breast	N63	1.000 (0.998, 1.002)	0.951477379
SARS2	Diagnoses - main ICD10: I84.8 Unspecified haemorrhoids with other complications	I84.8	0.999 (0.996, 1.002)	0.434793944
SARS2	Diagnoses - main ICD10: Z46.6 Fitting and adjustment of urinary device	Z46.6	0.999 (0.997, 1.002)	0.706902521
SARS2	Diagnoses - main ICD10: K01.1 Impacted teeth	K01.1	0.998 (0.996, 1.001)	0.159224696
SARS2	Diagnoses - secondary ICD10: Z80.3 Family history of malignant neoplasm of breast	Z80.3	1.000 (0.998, 1.001)	0.613266433
SARS2	Diagnoses - secondary ICD10: Y83.6 Removal of other organ (partial) (total)	Y83.6	1.001 (0.998, 1.004)	0.624342816
SARS2	Diagnoses - main ICD10: M65.3 Trigger finger	M65.3	0.999 (0.997, 1.001)	0.190840275
SARS2	Diagnoses - secondary ICD10: Z92.4 Personal history of major surgery, not elsewhere classified	Z92.4	1.001 (0.999, 1.002)	0.499490711
SARS2	Diagnoses - main ICD10: M23.2 Derangement of meniscus due to old tear or injury	M23.2	1.000 (0.998, 1.002)	0.989797418
SARS2	Diagnoses - main ICD10: K80.5 Calculus of bile duct without cholangitis or cholecystitis	K80.5	1.000 (0.997, 1.002)	0.715841011
SARS2	Type of cancer: ICD10: C44.9 Malignant neoplasm of skin, unspecified	C44.9	1.000 (0.997, 1.003)	0.965454821
SARS2	Diagnoses - main ICD10: D24 Benign neoplasm of breast	D24	1.001 (0.999, 1.003)	0.256449824
SARS2	Diagnoses - secondary ICD10: N85.8 Other specified noninflammatory disorders of uterus	N85.8	1.000 (0.998, 1.002)	0.802517881
SARS2	Diagnoses - secondary ICD10: I95.9 Hypotension, unspecified	I95.9	1.001 (0.999, 1.003)	0.485674639
SARS2	Diagnoses - secondary ICD10: B96.2 Escherichia coli [E. coli] as the cause of diseases classified to othe	B96.2	1.000 (0.998, 1.002)	0.885783678
SARS2	Diagnoses - main ICD10: K80.1 Calculus of gallbladder with other cholecystitis	K80.1	1.000 (0.997, 1.004)	0.831517632
SARS2	Diagnoses - secondary ICD10: Z53.0 Procedure not carried out because of contraindication	Z53.0	0.999 (0.995, 1.002)	0.490672493
SARS2	Diagnoses - secondary ICD10: Z53.8 Procedure not carried out for other reasons	Z53.8	1.002 (0.996, 1.008)	0.489282513
SARS2	Diagnoses - main ICD10: N35.9 Urethral stricture, unspecified	N35.9	1.002 (0.999, 1.004)	0.202598534
SARS2	Type of cancer: ICD10: D06.9 Cervix, unspecified	D06.9	0.995 (0.992, 0.998)	0.002607817
SARS2	Diagnoses - secondary ICD10: Z53.9 Procedure not carried out, unspecified reason	Z53.9	1.001 (0.999, 1.004)	0.306128803
SARS2	Diagnoses - main ICD10: O68.0 Labour and delivery complicated by foetal heart rate anomaly	O68.0	0.999 (0.998, 1.001)	0.390578094
SARS2	Diagnoses - main ICD10: D12.8 Rectum	D12.8	1.000 (0.998, 1.002)	0.887428087
SARS2	Diagnoses - secondary ICD10: M19.9 Arthrosis, unspecified	M19.9	1.001 (0.999, 1.003)	0.421891046
SARS2	Diagnoses - main ICD10: H02.8 Other specified disorders of eyelid	H02.8	0.999 (0.997, 1.001)	0.359258132
SARS2	Diagnoses - main ICD10: N92.1 Excessive and frequent menstruation with irregular cycle	N92.1	0.999 (0.997, 1.002)	0.531272348
SARS2	Diagnoses - main ICD10: O70.0 First degree perineal laceration during delivery	O70.0	1.000 (0.997, 1.002)	0.691100238
SARS2	Diagnoses - secondary ICD10: N80.0 Endometriosis of uterus	N80.0	1.000 (0.998, 1.001)	0.610516751
SARS2	Diagnoses - main ICD10: M23.22 Derangement of meniscus due to old tear or injury (Posterior cruciat	M23.22	0.999 (0.997, 1.002)	0.489161197
SARS2	Diagnoses - secondary ICD10: E11.9 Without complications	E11.9	0.993 (0.987, 0.998)	0.012758248
SARS2	Diagnoses - main ICD10: M79.66 Pain in limb (Lower leg)	M79.66	1.001 (0.998, 1.003)	0.583277685
SARS2	Diagnoses - main ICD10: O26.8 Other specified pregnancy-related conditions	O26.8	1.000 (0.998, 1.002)	0.850762241
SARS2	Diagnoses - main ICD10: K56.6 Other and unspecified intestinal obstruction	K56.6	1.001 (0.999, 1.002)	0.383321053
SARS2	Diagnoses - secondary ICD10: Z88.1 Personal history of allergy to other antibiotic agents	Z88.1	1.000 (0.997, 1.002)	0.909566781
SARS2	Diagnoses - secondary ICD10: J90 Pleural effusion, not elsewhere classified	J90	1.000 (0.997, 1.002)	0.891530218
SARS2	Underlying (primary) cause of death: ICD10: C34.9 Bronchus or lung, unspecified	C34.9	0.968 (0.888, 1.054)	0.452891131
SARS2	Diagnoses - main ICD10: A41 Other septicaemia	A41	1.001 (0.998, 1.004)	0.539803319

SARS2	Diagnoses - main ICD10: C18 Malignant neoplasm of colon	C18	1.001 (0.997, 1.004)	0.625009106
SARS2	Diagnoses - main ICD10: C34 Malignant neoplasm of bronchus and lung	C34	0.999 (0.996, 1.002)	0.603089703
SARS2	Diagnoses - main ICD10: C43 Malignant melanoma of skin	C43	0.999 (0.996, 1.002)	0.376136953
SARS2	Diagnoses - main ICD10: C67 Malignant neoplasm of bladder	C67	0.999 (0.996, 1.002)	0.577018696
SARS2	Diagnoses - main ICD10: C78 Secondary malignant neoplasm of respiratory and digestive organs	C78	1.002 (0.999, 1.004)	0.227337105
SARS2	Diagnoses - main ICD10: C79 Secondary malignant neoplasm of other sites	C79	1.000 (0.997, 1.002)	0.740873527
SARS2	Diagnoses - main ICD10: D05 Carcinoma in situ of breast	D05	1.001 (0.998, 1.003)	0.620613951
SARS2	Diagnoses - main ICD10: D17 Benign lipomatous neoplasm	D17	1.000 (0.996, 1.005)	0.853888978
SARS2	Diagnoses - main ICD10: D22 Melanocytic naevi	D22	0.997 (0.993, 1.001)	0.122833163
SARS2	Diagnoses - main ICD10: D23 Other benign neoplasms of skin	D23	1.000 (0.996, 1.004)	0.929195151
SARS2	Diagnoses - main ICD10: D50 Iron deficiency anaemia	D50	0.998 (0.994, 1.003)	0.442194497
SARS2	Diagnoses - main ICD10: D64 Other anaemias	D64	0.999 (0.995, 1.004)	0.731039303
SARS2	Diagnoses - main ICD10: G43 Migraine	G43	1.000 (0.998, 1.003)	0.84227436
SARS2	Diagnoses - main ICD10: G45 Transient cerebral ischaemic attacks and related syndromes	G45	0.999 (0.996, 1.002)	0.565763381
SARS2	Diagnoses - main ICD10: G57 Mononeuropathies of lower limb	G57	0.998 (0.996, 1.000)	0.109556575
SARS2	Diagnoses - main ICD10: H00 Hordeolum and chalazion	H00	1.002 (0.999, 1.005)	0.177942225
SARS2	Diagnoses - main ICD10: H02 Other disorders of eyelid	H02	1.000 (0.995, 1.005)	0.955929938
SARS2	Diagnoses - main ICD10: H04 Disorders of lachrymal system	H04	1.000 (0.997, 1.003)	0.898027555
SARS2	Diagnoses - main ICD10: H33 Retinal detachments and breaks	H33	0.999 (0.996, 1.003)	0.774544358
SARS2	Diagnoses - main ICD10: H35 Other retinal disorders	H35	0.999 (0.996, 1.002)	0.508544771
SARS2	Diagnoses - main ICD10: H40 Glaucoma	H40	0.999 (0.996, 1.002)	0.475355061
SARS2	Diagnoses - main ICD10: I26 Pulmonary embolism	I26	1.004 (1.001, 1.008)	0.011664527
SARS2	Diagnoses - main ICD10: I47 Paroxysmal tachycardia	I47	1.001 (0.998, 1.004)	0.595164897
SARS2	Diagnoses - main ICD10: I50 Heart failure	I50	0.999 (0.997, 1.002)	0.466980883
SARS2	Diagnoses - main ICD10: I63 Cerebral infarction	I63	0.999 (0.996, 1.003)	0.765330627
SARS2	Diagnoses - main ICD10: J18 Pneumonia, organism unspecified	J18	0.999 (0.994, 1.004)	0.823516633
SARS2	Diagnoses - main ICD10: J32 Chronic sinusitis	J32	0.999 (0.996, 1.001)	0.253353713
SARS2	Diagnoses - main ICD10: J38 Diseases of vocal cords and larynx, not elsewhere classified	J38	0.999 (0.997, 1.002)	0.58359038
SARS2	Diagnoses - main ICD10: J45 Asthma	J45	1.000 (0.997, 1.003)	0.797223622
SARS2	Diagnoses - main ICD10: K01 Embedded and impacted teeth	K01	0.998 (0.996, 1.001)	0.210411028
SARS2	Diagnoses - main ICD10: K02 Dental caries	K02	0.999 (0.996, 1.002)	0.542495613
SARS2	Diagnoses - main ICD10: K04 Diseases of pulp and periapical tissues	K04	1.001 (0.998, 1.003)	0.662137649
SARS2	Diagnoses - main ICD10: K08 Other disorders of teeth and supporting structures	K08	0.999 (0.996, 1.002)	0.60813703
SARS2	Diagnoses - main ICD10: K13 Other diseases of lip and oral mucosa	K13	0.996 (0.993, 1.000)	0.027935137
SARS2	Diagnoses - main ICD10: K25 Gastric ulcer	K25	1.001 (0.998, 1.004)	0.391886903
SARS2	Diagnoses - main ICD10: K26 Duodenal ulcer	K26	1.001 (0.998, 1.004)	0.421711504
SARS2	Diagnoses - main ICD10: K31 Other diseases of stomach and duodenum	K31	0.997 (0.993, 1.000)	0.065907682

SARS2	Diagnoses - main ICD10: K42 Umbilical hernia	K42	0.996 (0.993, 1.000)	0.041503159
SARS2	Diagnoses - main ICD10: K56 Paralytic ileus and intestinal obstruction without hernia	K56	1.001 (0.998, 1.004)	0.642717519
SARS2	Diagnoses - main ICD10: K58 Irritable bowel syndrome	K58	1.000 (0.998, 1.003)	0.733635542
SARS2	Diagnoses - main ICD10: K59 Other functional intestinal disorders	K59	0.998 (0.994, 1.003)	0.483760692
SARS2	Diagnoses - main ICD10: K61 Abscess of anal and rectal regions	K61	0.997 (0.995, 1.000)	0.019953472
SARS2	Diagnoses - main ICD10: K63 Other diseases of intestine	K63	0.998 (0.991, 1.004)	0.440882388
SARS2	Diagnoses - main ICD10: K81 Cholecystitis	K81	1.001 (0.998, 1.004)	0.492137228
SARS2	Diagnoses - main ICD10: K92 Other diseases of digestive system	K92	0.998 (0.993, 1.003)	0.512718016
SARS2	Diagnoses - main ICD10: L02 Cutaneous abscess, furuncle and carbuncle	L02	0.998 (0.995, 1.001)	0.127003467
SARS2	Diagnoses - main ICD10: L57 Skin changes due to chronic exposure to nonionising radiation	L57	1.003 (1.000, 1.006)	0.025569097
SARS2	Diagnoses - main ICD10: L72 Follicular cysts of skin and subcutaneous tissue	L72	1.006 (1.001, 1.012)	0.032331984
SARS2	Diagnoses - main ICD10: L90 Atrophic disorders of skin	L90	1.000 (0.997, 1.003)	0.844319091
SARS2	Diagnoses - main ICD10: L98 Other disorders of skin and subcutaneous tissue, not elsewhere classified	L98	0.999 (0.995, 1.004)	0.676719535
SARS2	Diagnoses - main ICD10: M06 Other rheumatoid arthritis	M06	1.002 (0.999, 1.004)	0.225679595
SARS2	Diagnoses - main ICD10: M13 Other arthritis	M13	1.001 (0.998, 1.003)	0.619073478
SARS2	Diagnoses - main ICD10: M15 Polyarthrosis	M15	1.000 (0.998, 1.003)	0.869482118
SARS2	Diagnoses - main ICD10: M19 Other arthrosis	M19	1.002 (0.997, 1.007)	0.405285365
SARS2	Diagnoses - main ICD10: M47 Spondylosis	M47	1.002 (0.998, 1.005)	0.365266655
SARS2	Diagnoses - main ICD10: M48 Other spondylopathies	M48	0.997 (0.994, 1.000)	0.071805833
SARS2	Diagnoses - main ICD10: M51 Other intervertebral disk disorders	M51	0.999 (0.994, 1.004)	0.59262727
SARS2	Diagnoses - main ICD10: M65 Synovitis and tenosynovitis	M65	1.002 (0.999, 1.006)	0.228240897
SARS2	Diagnoses - main ICD10: M75 Shoulder lesions	M75	0.999 (0.993, 1.005)	0.767766468
SARS2	Diagnoses - main ICD10: M79 Other soft tissue disorders, not elsewhere classified	M79	1.000 (0.994, 1.006)	0.971873229
SARS2	Diagnoses - main ICD10: M84 Disorders of continuity of bone	M84	1.000 (0.997, 1.002)	0.791496297
SARS2	Diagnoses - main ICD10: N13 Obstructive and reflux uropathy	N13	1.001 (0.998, 1.003)	0.580868482
SARS2	Diagnoses - main ICD10: N30 Cystitis	N30	1.001 (0.998, 1.004)	0.563391642
SARS2	Diagnoses - main ICD10: N31 Neuromuscular dysfunction of bladder, not elsewhere classified	N31	1.001 (0.999, 1.004)	0.291454083
SARS2	Diagnoses - main ICD10: N35 Urethral stricture	N35	1.002 (0.998, 1.005)	0.338759612
SARS2	Diagnoses - main ICD10: N39 Other disorders of urinary system	N39	1.001 (0.994, 1.009)	0.689701007
SARS2	Diagnoses - main ICD10: N48 Other disorders of penis	N48	1.000 (0.997, 1.003)	0.993546243
SARS2	Diagnoses - main ICD10: N50 Other disorders of male genital organs	N50	0.998 (0.995, 1.001)	0.304999351
SARS2	Diagnoses - main ICD10: N60 Benign mammary dysplasia	N60	1.001 (0.999, 1.004)	0.272960295
SARS2	Diagnoses - main ICD10: N80 Endometriosis	N80	0.999 (0.996, 1.001)	0.306110667
SARS2	Diagnoses - main ICD10: N83 Noninflammatory disorders of ovary, Fallopian tube and broad ligamen	N83	0.998 (0.994, 1.001)	0.137238376
SARS2	Diagnoses - main ICD10: N84 Polyp of female genital tract	N84	1.002 (0.996, 1.008)	0.462791518
SARS2	Diagnoses - main ICD10: N85 Other noninflammatory disorders of uterus, except cervix	N85	1.000 (0.998, 1.003)	0.718238792
SARS2	Diagnoses - main ICD10: N87 Dysplasia of cervix uteri	N87	0.998 (0.996, 1.001)	0.152760968

SARS2	Diagnoses - main ICD10: N90 Other noninflammatory disorders of vulva and perineum	N90	1.001 (0.998, 1.003)	0.536111905
SARS2	Diagnoses - main ICD10: N93 Other abnormal uterine and vaginal bleeding	N93	0.998 (0.995, 1.002)	0.370159144
SARS2	Diagnoses - main ICD10: N94 Pain and other conditions associated with female genital organs and men	N94	0.999 (0.997, 1.002)	0.455440911
SARS2	Diagnoses - main ICD10: N95 Menopausal and other perimenopausal disorders	N95	1.001 (0.995, 1.006)	0.797582104
SARS2	Diagnoses - main ICD10: O02 Other abnormal products of conception	O02	1.003 (1.001, 1.006)	0.005615036
SARS2	Diagnoses - main ICD10: O03 Spontaneous abortion	O03	0.999 (0.997, 1.001)	0.352412665
SARS2	Diagnoses - main ICD10: O26 Maternal care for other conditions predominantly related to pregnancy	O26	1.002 (0.999, 1.004)	0.16291161
SARS2	Diagnoses - main ICD10: O36 Maternal care for other known or suspected foetal problems	O36	1.001 (0.999, 1.003)	0.487306736
SARS2	Diagnoses - main ICD10: O63 Long labour	O63	1.000 (0.998, 1.002)	0.889656522
SARS2	Diagnoses - main ICD10: O68 Labour and delivery complicated by foetal stress [distress]	O68	0.997 (0.994, 1.000)	0.067488983
SARS2	Diagnoses - main ICD10: O70 Perineal laceration during delivery	O70	1.002 (0.998, 1.006)	0.249438889
SARS2	Diagnoses - main ICD10: O80 Single spontaneous delivery	O80	0.997 (0.994, 0.999)	0.019923741
SARS2	Diagnoses - main ICD10: R00 Abnormalities of heart beat	R00	1.001 (0.998, 1.005)	0.509166971
SARS2	Diagnoses - main ICD10: R06 Abnormalities of breathing	R06	1.000 (0.995, 1.004)	0.972174628
SARS2	Diagnoses - main ICD10: R19 Other symptoms and signs involving the digestive system and abdomen	R19	0.998 (0.992, 1.005)	0.593017706
SARS2	Diagnoses - main ICD10: R22 Localised swelling, mass and lump of skin and subcutaneous tissue	R22	1.001 (0.998, 1.003)	0.702955426
SARS2	Diagnoses - main ICD10: R39 Other symptoms and signs involving the urinary system	R39	1.001 (0.997, 1.004)	0.69649353
SARS2	Diagnoses - main ICD10: R50 Fever of unknown origin	R50	1.000 (0.998, 1.003)	0.89298434
SARS2	Diagnoses - main ICD10: R56 Convulsions, not elsewhere classified	R56	1.001 (0.998, 1.003)	0.510002859
SARS2	Diagnoses - main ICD10: R59 Enlarged lymph nodes	R59	0.999 (0.996, 1.001)	0.231863518
SARS2	Diagnoses - main ICD10: R63 Symptoms and signs concerning food and fluid intake	R63	0.999 (0.997, 1.002)	0.444975959
SARS2	Diagnoses - main ICD10: R79 Other abnormal findings of blood chemistry	R79	1.000 (0.996, 1.004)	0.987802311
SARS2	Diagnoses - main ICD10: R87 Abnormal findings in specimens from female genital organs	R87	1.000 (0.997, 1.002)	0.730961392
SARS2	Diagnoses - main ICD10: R93 Abnormal findings on diagnostic imaging of other body structures	R93	1.003 (1.001, 1.006)	0.010488213
SARS2	Diagnoses - main ICD10: S01 Open wound of head	S01	1.000 (0.997, 1.003)	0.969330024
SARS2	Diagnoses - main ICD10: S02 Fracture of skull and facial bones	S02	1.001 (0.999, 1.004)	0.368926106
SARS2	Diagnoses - main ICD10: S42 Fracture of shoulder and upper arm	S42	1.004 (1.001, 1.007)	0.013613819
SARS2	Diagnoses - main ICD10: S61 Open wound of wrist and hand	S61	1.003 (1.000, 1.006)	0.058914852
SARS2	Diagnoses - main ICD10: S62 Fracture at wrist and hand level	S62	1.000 (0.997, 1.003)	0.930983398
SARS2	Diagnoses - main ICD10: S72 Fracture of femur	S72	1.000 (0.997, 1.003)	0.953968988
SARS2	Diagnoses - main ICD10: S82 Fracture of lower leg, including ankle	S82	1.001 (0.997, 1.006)	0.539311403
SARS2	Diagnoses - main ICD10: T39 Poisoning by nonopioid analgesics, antipyretics and antirheumatics	T39	0.999 (0.997, 1.001)	0.443472192
SARS2	Diagnoses - main ICD10: T81 Complications of procedures, not elsewhere classified	T81	0.997 (0.992, 1.003)	0.339488798
SARS2	Diagnoses - main ICD10: T82 Complications of cardiac and vascular prosthetic devices, implants and g	T82	0.999 (0.997, 1.002)	0.625830572
SARS2	Diagnoses - main ICD10: T85 Complications of other internal prosthetic devices, implants and grafts	T85	0.998 (0.995, 1.001)	0.117057113
SARS2	Diagnoses - main ICD10: Z01 Other special examinations and investigations of persons without compla	Z01	1.001 (0.998, 1.003)	0.517358479
SARS2	Diagnoses - main ICD10: Z03 Medical observation and evaluation for suspected diseases and condition	Z03	1.002 (0.997, 1.007)	0.506503258

SARS2	Diagnoses - main ICD10: Z08 Follow-up examination after treatment for malignant neoplasm	Z08	1.002 (0.997, 1.006)	0.462015926
SARS2	Diagnoses - main ICD10: Z12 Special screening examination for neoplasms	Z12	0.999 (0.995, 1.004)	0.791285222
SARS2	Diagnoses - main ICD10: Z13 Special screening examination for other diseases and disorders	Z13	1.001 (0.998, 1.005)	0.38026484
SARS2	Diagnoses - main ICD10: Z30 Contraceptive management	Z30	0.999 (0.993, 1.004)	0.688834239
SARS2	Diagnoses - main ICD10: Z42 Follow-up care involving plastic surgery	Z42	1.001 (0.998, 1.004)	0.495194145
SARS2	Diagnoses - main ICD10: Z43 Attention to artificial openings	Z43	1.002 (1.000, 1.005)	0.091062241
SARS2	Diagnoses - main ICD10: Z45 Adjustment and management of implanted device	Z45	1.002 (0.999, 1.005)	0.227016751
SARS2	Diagnoses - main ICD10: Z46 Fitting and adjustment of other devices	Z46	1.001 (0.997, 1.005)	0.641087676
SARS2	Diagnoses - main ICD10: Z53 Persons encountering health services for specifie procedures, not carried	Z53	1.003 (1.000, 1.006)	0.05034429

Table S14 Phenome-wide Mendelian Randomization analysis to reveal potential on-target side effects of VAT1

protein	disease	ICD-10 code	OR (95%CI)	P value
VAT1	Underlying (primary) cause of death: ICD10: E85.4 Organ-limited amyloidosis	E85.4	0.993 (0.983, 1.004)	0.213005843
VAT1	Underlying (primary) cause of death: ICD10: J84.1 Other interstitial pulmonary diseases with fibrosis	J84.1	1.017 (0.978, 1.057)	0.408909818
VAT1	Diagnoses - main ICD10: B37 Candidiasis	B37	1.000 (0.999, 1.001)	0.771631706
VAT1	Diagnoses - main ICD10: C44 Other malignant neoplasms of skin	C44	1.008 (1.002, 1.014)	0.014506538
VAT1	Diagnoses - main ICD10: C50 Malignant neoplasm of breast	C50	1.004 (0.998, 1.011)	0.214526961
VAT1	Diagnoses - main ICD10: C61 Malignant neoplasm of prostate	C61	0.999 (0.995, 1.004)	0.801630737
VAT1	Diagnoses - main ICD10: D12 Benign neoplasm of colon rectum anus and anal canal	D12	1.001 (0.995, 1.007)	0.767799462
VAT1	Diagnoses - main ICD10: D25 Leiomyoma of uterus	D25	1.010 (1.004, 1.016)	0.001361097
VAT1	Diagnoses - main ICD10: E03 Other hypothyroidism	E03	1.000 (0.999, 1.001)	0.471796651
VAT1	Diagnoses - main ICD10: E04 Other non-toxic goitre	E04	1.001 (0.999, 1.004)	0.241616481
VAT1	Diagnoses - main ICD10: F31 Bipolar affective disorder	F31	1.000 (0.998, 1.001)	0.545978255
VAT1	Diagnoses - main ICD10: F43 Reaction to severe stress and adjustment disorders	F43	1.000 (0.999, 1.001)	0.729256283
VAT1	Diagnoses - main ICD10: G47 Sleep disorders	G47	1.000 (0.996, 1.004)	0.873384842
VAT1	Diagnoses - main ICD10: G56 Mononeuropathies of upper limb	G56	0.998 (0.991, 1.005)	0.592683648
VAT1	Diagnoses - main ICD10: H25 Senile cataract	H25	1.000 (0.996, 1.005)	0.84081848
VAT1	Diagnoses - main ICD10: H26 Other cataract	H26	0.992 (0.985, 0.999)	0.025372866
VAT1	Diagnoses - main ICD10: I10 Essential (primary) hypertension	I10	1.001 (0.999, 1.003)	0.275648199
VAT1	Diagnoses - main ICD10: I20 Angina pectoris	I20	1.000 (0.993, 1.006)	0.904198771
VAT1	Diagnoses - main ICD10: I21 Acute myocardial infarction	I21	0.999 (0.993, 1.004)	0.642484994
VAT1	Diagnoses - main ICD10: I25 Chronic ischaemic heart disease	I25	0.995 (0.987, 1.003)	0.22635058
VAT1	Diagnoses - main ICD10: I30 Acute pericarditis	I30	1.000 (0.999, 1.001)	0.393831481
VAT1	Diagnoses - main ICD10: I48 Atrial fibrillation and flutter	I48	0.999 (0.993, 1.005)	0.730640792
VAT1	Diagnoses - main ICD10: I80 Phlebitis and thrombophlebitis	I80	1.000 (0.997, 1.004)	0.853649689
VAT1	Diagnoses - main ICD10: I84 Haemorrhoids	I84	1.004 (0.996, 1.012)	0.312400253
VAT1	Diagnoses - main ICD10: J22 Unspecified acute lower respiratory infection	J22	1.000 (0.996, 1.004)	0.940113345
VAT1	Diagnoses - main ICD10: J33 Nasal polyp	J33	1.000 (0.996, 1.003)	0.860096103
VAT1	Diagnoses - main ICD10: J34 Other disorders of nose and nasal sinuses	J34	1.004 (0.999, 1.010)	0.108228549
VAT1	Diagnoses - main ICD10: J44 Other chronic obstructive pulmonary disease	J44	1.000 (0.997, 1.003)	0.91304344
VAT1	Diagnoses - main ICD10: K20 Oesophagitis	K20	1.002 (0.996, 1.007)	0.552112032
VAT1	Diagnoses - main ICD10: K21 Gastro-oesophageal reflux disease	K21	0.996 (0.989, 1.004)	0.348613616
VAT1	Diagnoses - main ICD10: K22 Other diseases of oesophagus	K22	0.998 (0.993, 1.003)	0.425440531
VAT1	Diagnoses - main ICD10: K29 Gastritis and duodenitis	K29	0.998 (0.990, 1.006)	0.588100347
VAT1	Diagnoses - main ICD10: K35 Acute appendicitis	K35	1.001 (0.997, 1.005)	0.662610951
VAT1	Diagnoses - main ICD10: K40 Inguinal hernia	K40	0.999 (0.991, 1.008)	0.847970663
VAT1	Diagnoses - main ICD10: K43 Ventral hernia	K43	1.000 (0.996, 1.004)	0.993945259

VAT1	Diagnoses - main ICD10: K44 Diaphragmatic hernia	K44	1.000 (0.993, 1.007)	0.990661021
VAT1	Diagnoses - main ICD10: K50 Crohn's disease [regional enteritis]	K50	1.001 (0.999, 1.004)	0.237514941
VAT1	Diagnoses - main ICD10: K51 Ulcerative colitis	K51	1.002 (0.999, 1.006)	0.229100433
VAT1	Diagnoses - main ICD10: K52 Other non-infective gastro-enteritis and colitis	K52	0.993 (0.986, 1.001)	0.073435059
VAT1	Diagnoses - main ICD10: K57 Diverticular disease of intestine	K57	1.002 (0.994, 1.009)	0.692713467
VAT1	Diagnoses - main ICD10: K60 Fissure and fistula of anal and rectal regions	K60	0.997 (0.993, 1.000)	0.059621586
VAT1	Diagnoses - main ICD10: K62 Other diseases of anus and rectum	K62	0.998 (0.989, 1.007)	0.710441056
VAT1	Diagnoses - main ICD10: K76 Other diseases of liver	K76	0.999 (0.998, 1.001)	0.551789373
VAT1	Diagnoses - main ICD10: K80 Cholelithiasis	K80	1.005 (0.998, 1.013)	0.180964284
VAT1	Diagnoses - main ICD10: L03 Cellulitis	L03	1.001 (0.996, 1.006)	0.740927766
VAT1	Diagnoses - main ICD10: M10 Gout	M10	0.999 (0.998, 1.001)	0.333007958
VAT1	Diagnoses - main ICD10: M16 Coxarthrosis [arthrosis of hip]	M16	0.997 (0.991, 1.004)	0.391341258
VAT1	Diagnoses - main ICD10: M17 Gonarthrosis [arthrosis of knee]	M17	1.007 (1.000, 1.015)	0.04691753
VAT1	Diagnoses - main ICD10: M20 Acquired deformities of fingers and toes	M20	1.000 (0.993, 1.006)	0.967617376
VAT1	Diagnoses - main ICD10: M21 Other acquired deformities of limbs	M21	1.000 (0.999, 1.002)	0.622173234
VAT1	Diagnoses - main ICD10: M23 Internal derangement of knee	M23	1.005 (0.998, 1.013)	0.172445286
VAT1	Diagnoses - main ICD10: M24 Other specific joint derangements	M24	1.000 (0.998, 1.003)	0.867354466
VAT1	Diagnoses - main ICD10: M25 Other joint disorders not elsewhere classified	M25	1.002 (0.996, 1.009)	0.501701727
VAT1	Diagnoses - main ICD10: M54 Dorsalgia	M54	1.000 (0.993, 1.007)	0.970313619
VAT1	Diagnoses - main ICD10: M67 Other disorders of synovium and tendon	M67	1.001 (0.997, 1.005)	0.521173135
VAT1	Diagnoses - main ICD10: M70 Soft tissue disorders related to use overuse and pressure	M70	1.001 (0.998, 1.003)	0.507074042
VAT1	Diagnoses - main ICD10: M72 Fibroblastic disorders	M72	0.999 (0.996, 1.003)	0.778163358
VAT1	Diagnoses - main ICD10: N19 Unspecified renal failure	N19	1.001 (1.000, 1.002)	0.073416339
VAT1	Diagnoses - main ICD10: N20 Calculus of kidney and ureter	N20	1.004 (0.999, 1.008)	0.106876476
VAT1	Diagnoses - main ICD10: N32 Other disorders of bladder	N32	1.000 (0.995, 1.005)	0.933268446
VAT1	Diagnoses - main ICD10: N40 Hyperplasia of prostate	N40	0.997 (0.992, 1.002)	0.312241442
VAT1	Diagnoses - main ICD10: N81 Female genital prolapse	N81	0.990 (0.984, 0.997)	0.002389059
VAT1	Diagnoses - main ICD10: N92 Excessive frequent and irregular menstruation	N92	0.996 (0.989, 1.003)	0.277371815
VAT1	Diagnoses - main ICD10: O75 Other complications of labour and delivery not elsewhere classified	O75	1.000 (0.999, 1.001)	0.852555345
VAT1	Diagnoses - main ICD10: R04 Haemorrhage from respiratory passages	R04	1.002 (0.998, 1.006)	0.387258561
VAT1	Diagnoses - main ICD10: R07 Pain in throat and chest	R07	0.991 (0.980, 1.002)	0.124659164
VAT1	Diagnoses - main ICD10: R10 Abdominal and pelvic pain	R10	1.002 (0.991, 1.012)	0.77413546
VAT1	Diagnoses - main ICD10: R11 Nausea and vomiting	R11	1.002 (0.998, 1.005)	0.368756736
VAT1	Diagnoses - main ICD10: R14 Flatulence and related conditions	R14	1.001 (0.999, 1.002)	0.423402746
VAT1	Diagnoses - main ICD10: R31 Unspecified haematuria	R31	1.000 (0.992, 1.007)	0.934404342
VAT1	Diagnoses - main ICD10: R35 Polyuria	R35	1.000 (0.996, 1.003)	0.86463225
VAT1	Diagnoses - main ICD10: R55 Syncope and collapse	R55	0.998 (0.993, 1.003)	0.455317471

VAT1	Diagnoses - main ICD10: R69 Unknown and unspecified causes of morbidity	R69	0.996 (0.989, 1.004)	0.324427528
VAT1	Diagnoses - main ICD10: S09 Other and unspecified injuries of head	S09	1.000 (0.998, 1.003)	0.756849401
VAT1	Diagnoses - main ICD10: S52 Fracture of forearm	S52	1.001 (0.996, 1.006)	0.653843521
VAT1	Diagnoses - main ICD10: S66 Injury of muscle and tendon at wrist and hand level	S66	1.000 (0.998, 1.002)	0.987486425
VAT1	Diagnoses - main ICD10: S76 Injury of muscle and tendon at hip and thigh level	S76	1.000 (0.999, 1.001)	0.970483577
VAT1	Diagnoses - main ICD10: T84 Complications of internal orthopaedic prosthetic devices implants and gr	T84	1.000 (0.996, 1.004)	0.964394766
VAT1	Diagnoses - main ICD10: Z09 Follow-up examination after treatment for conditions other than maligna	Z09	1.001 (0.995, 1.008)	0.661575778
VAT1	Diagnoses - main ICD10: Z47 Other orthopaedic follow-up care	Z47	1.001 (0.997, 1.005)	0.617750538
VAT1	Diagnoses - main ICD10: Z80 Family history of malignant neoplasm	Z80	1.001 (1.000, 1.003)	0.16427611
VAT1	Diagnoses - secondary ICD10: Z50.1 Other physical therapy	Z50.1	1.000 (0.998, 1.003)	0.814456602
VAT1	Diagnoses - main ICD10: Z09.0 Follow-up examination after surgery for other conditions	Z09.0	1.000 (0.996, 1.004)	0.986973364
VAT1	Diagnoses - secondary ICD10: K21.0 Gastro-oesophageal reflux disease with oesophagitis	K21.0	0.998 (0.995, 1.001)	0.169718753
VAT1	Type of cancer: ICD10: C44.3 Skin of other and unspecified parts of face	C44.3	1.001 (0.996, 1.006)	0.800925982
VAT1	Diagnoses - main ICD10: K80.0 Calculus of gallbladder with acute cholecystitis	K80.0	1.001 (0.999, 1.004)	0.231845143
VAT1	Diagnoses - secondary ICD10: R19.4 Change in bowel habit	R19.4	1.000 (0.997, 1.003)	0.998929183
VAT1	Diagnoses - secondary ICD10: R10.1 Pain localised to upper abdomen	R10.1	0.999 (0.997, 1.002)	0.551958671
VAT1	Diagnoses - secondary ICD10: Z03.8 Observation for other suspected diseases and conditions	Z03.8	1.000 (0.997, 1.003)	0.945415684
VAT1	Diagnoses - secondary ICD10: I50.1 Left ventricular failure	I50.1	1.000 (0.997, 1.003)	0.89115506
VAT1	Diagnoses - main ICD10: D12.5 Sigmoid colon	D12.5	0.999 (0.995, 1.002)	0.413441561
VAT1	Diagnoses - main ICD10: Z09.9 Follow-up examination after unspecified treatment for other conditions	Z09.9	1.001 (0.998, 1.004)	0.629824494
VAT1	Diagnoses - main ICD10: N47 Redundant prepuce, phimosis and paraphimosis	N47	0.998 (0.995, 1.000)	0.096445768
VAT1	Diagnoses - secondary ICD10: E10.9 Without complications	E10.9	1.000 (0.997, 1.003)	0.885868455
VAT1	Diagnoses - main ICD10: O70.1 Second degree perineal laceration during delivery	O70.1	0.997 (0.994, 1.000)	0.055815195
VAT1	Diagnoses - secondary ICD10: I20.0 Unstable angina	I20.0	1.001 (0.998, 1.004)	0.413419863
VAT1	Diagnoses - main ICD10: R07.4 Chest pain, unspecified	R07.4	0.990 (0.982, 0.999)	0.026092594
VAT1	Diagnoses - main ICD10: N93.9 Abnormal uterine and vaginal bleeding, unspecified	N93.9	1.000 (0.997, 1.002)	0.667506642
VAT1	Diagnoses - main ICD10: K57.3 Diverticular disease of large intestine without perforation or abscess	K57.3	1.002 (0.996, 1.008)	0.518968039
VAT1	Diagnoses - main ICD10: M54.59 Low back pain (Site unspecified)	M54.59	1.001 (0.998, 1.003)	0.480135149
VAT1	Diagnoses - secondary ICD10: Z87.4 Personal history of diseases of the genito-urinary system	Z87.4	0.998 (0.993, 1.002)	0.314608666
VAT1	Diagnoses - secondary ICD10: Z92.2 Personal history of long-term (current) use of other medicaments	Z92.2	0.998 (0.992, 1.004)	0.506489878
VAT1	Diagnoses - main ICD10: K80.2 Calculus of gallbladder without cholecystitis	K80.2	1.003 (0.998, 1.008)	0.274124641
VAT1	Diagnoses - secondary ICD10: M13.9 Arthritis, unspecified	M13.9	1.001 (0.999, 1.004)	0.41030902
VAT1	Diagnoses - main ICD10: R79.8 Other specified abnormal findings of blood chemistry	R79.8	0.998 (0.995, 1.001)	0.148724194
VAT1	Diagnoses - secondary ICD10: Z95.1 Presence of aortocoronary bypass graft	Z95.1	1.000 (0.997, 1.004)	0.837213969
VAT1	Diagnoses - secondary ICD10: W01.0 Home	W01.0	1.003 (1.001, 1.005)	0.006518167
VAT1	Diagnoses - secondary ICD10: Z85.8 Personal history of malignant neoplasms of other organs and syste	Z85.8	1.002 (0.998, 1.006)	0.248954903
VAT1	Diagnoses - main ICD10: I84.9 Unspecified haemorrhoids without complication	I84.9	1.005 (0.999, 1.010)	0.087348103

VAT1	Diagnoses - main ICD10: K60.2 Anal fissure, unspecified	K60.2	0.999 (0.997, 1.001)	0.538908691
VAT1	Diagnoses - main ICD10: N81.1 Cystocele	N81.1	0.999 (0.995, 1.002)	0.437106171
VAT1	Diagnoses - secondary ICD10: F41.9 Anxiety disorder, unspecified	F41.9	1.000 (0.997, 1.002)	0.917591297
VAT1	Diagnoses - main ICD10: K40.2 Bilateral inguinal hernia, without obstruction or gangrene	K40.2	1.002 (1.000, 1.004)	0.095640715
VAT1	Diagnoses - main ICD10: L90.5 Scar conditions and fibrosis of skin	L90.5	1.003 (1.001, 1.006)	0.017800067
VAT1	Diagnoses - main ICD10: R04.0 Epistaxis	R04.0	1.000 (0.997, 1.002)	0.751181455
VAT1	Diagnoses - secondary ICD10: Z51.1 Chemotherapy session for neoplasm	Z51.1	1.005 (0.999, 1.010)	0.120221366
VAT1	Diagnoses - main ICD10: R32 Unspecified urinary incontinence	R32	1.000 (0.998, 1.002)	0.990865906
VAT1	Diagnoses - main ICD10: N92.0 Excessive and frequent menstruation with regular cycle	N92.0	0.998 (0.993, 1.003)	0.498370673
VAT1	Diagnoses - main ICD10: K62.5 Haemorrhage of anus and rectum	K62.5	1.001 (0.995, 1.007)	0.774890436
VAT1	Diagnoses - secondary ICD10: Z96.1 Presence of intraocular lens	Z96.1	1.001 (0.997, 1.004)	0.734592967
VAT1	Diagnoses - secondary ICD10: R00.1 Bradycardia, unspecified	R00.1	0.999 (0.997, 1.001)	0.377240185
VAT1	Diagnoses - secondary ICD10: K66.0 Peritoneal adhesions	K66.0	1.001 (0.998, 1.003)	0.567449082
VAT1	Diagnoses - main ICD10: T84.8 Other complications of internal orthopaedic prosthetic devices, implant	T84.8	0.999 (0.997, 1.001)	0.512401893
VAT1	Diagnoses - secondary ICD10: Z85.4 Personal history of malignant neoplasm of genital organs	Z85.4	1.001 (0.997, 1.004)	0.769709249
VAT1	Diagnoses - main ICD10: I47.1 Supraventricular tachycardia	I47.1	0.999 (0.996, 1.001)	0.246523876
VAT1	Diagnoses - secondary ICD10: F10.0 Acute intoxication	F10.0	1.001 (0.999, 1.004)	0.298386805
VAT1	Diagnoses - main ICD10: K08.3 Retained dental root	K08.3	1.001 (0.999, 1.003)	0.432519017
VAT1	Diagnoses - secondary ICD10: M06.99 Rheumatoid arthritis, unspecified (Site unspecified)	M06.99	1.000 (0.998, 1.003)	0.826257573
VAT1	Diagnoses - main ICD10: L03.1 Cellulitis of other parts of limb	L03.1	0.998 (0.995, 1.002)	0.43385912
VAT1	Diagnoses - secondary ICD10: R53 Malaise and fatigue	R53	1.000 (0.998, 1.002)	0.904688691
VAT1	Diagnoses - secondary ICD10: W01.9 Unspecified place	W01.9	1.001 (0.999, 1.003)	0.390858763
VAT1	Diagnoses - main ICD10: C50.4 Upper-outer quadrant of breast	C50.4	0.999 (0.997, 1.002)	0.587872531
VAT1	Diagnoses - main ICD10: T39.1 4-Aminophenol derivatives	T39.1	1.000 (0.998, 1.002)	0.996020799
VAT1	Diagnoses - secondary ICD10: K29.5 Chronic gastritis, unspecified	K29.5	1.001 (0.999, 1.004)	0.203459224
VAT1	Diagnoses - secondary ICD10: G55.1 Nerve root and plexus compressions in intervertebral disk disorde	G55.1	1.001 (0.998, 1.004)	0.582008682
VAT1	Diagnoses - secondary ICD10: K25.9 Unspecified as acute or chronic, without haemorrhage or perforat	K25.9	1.002 (0.999, 1.004)	0.130480479
VAT1	Diagnoses - main ICD10: I80.2 Phlebitis and thrombophlebitis of other deep vessels of lower extremitie	I80.2	1.000 (0.997, 1.002)	0.72916219
VAT1	Diagnoses - secondary ICD10: I84.6 Residual haemorrhoidal skin tags	I84.6	1.000 (0.997, 1.003)	0.906813307
VAT1	Diagnoses - main ICD10: N32.0 Bladder-neck obstruction	N32.0	1.001 (0.999, 1.004)	0.261915569
VAT1	Diagnoses - main ICD10: K29.8 Duodenitis	K29.8	1.000 (0.997, 1.003)	0.996025552
VAT1	Diagnoses - main ICD10: M67.4 Ganglion	M67.4	1.000 (0.997, 1.004)	0.774720188
VAT1	Diagnoses - main ICD10: H26.9 Cataract, unspecified	H26.9	0.998 (0.992, 1.005)	0.608822014
VAT1	Type of cancer: ICD10: C20 Malignant neoplasm of rectum	C20	1.002 (1.000, 1.005)	0.079188296
VAT1	Diagnoses - secondary ICD10: K30 Dyspepsia	K30	0.998 (0.995, 1.001)	0.127758
VAT1	Diagnoses - secondary ICD10: I84.2 Internal haemorrhoids without complication	I84.2	1.000 (0.998, 1.002)	0.949217133
VAT1	Diagnoses - secondary ICD10: N94.6 Dysmenorrhoea, unspecified	N94.6	0.999 (0.997, 1.002)	0.578721953

VAT1	Diagnoses - main ICD10: Z08.0 Follow-up examination after surgery for malignant neoplasm	Z08.0	1.002 (0.999, 1.006)	0.229888654
VAT1	Diagnoses - main ICD10: M79.86 Other specified soft tissue disorders (Lower leg)	M79.86	1.002 (0.999, 1.005)	0.150515712
VAT1	Diagnoses - secondary ICD10: E78.0 Pure hypercholesterolaemia	E78.0	0.997 (0.987, 1.007)	0.540628167
VAT1	Diagnoses - secondary ICD10: M10.99 Gout, unspecified (Site unspecified)	M10.99	1.001 (0.999, 1.003)	0.357860311
VAT1	Diagnoses - main ICD10: M51.2 Other specified intervertebral disk displacement	M51.2	1.003 (1.000, 1.005)	0.039276794
VAT1	Diagnoses - main ICD10: N84.0 Polyp of corpus uteri	N84.0	1.002 (0.997, 1.007)	0.34943807
VAT1	Diagnoses - secondary ICD10: M17.1 Other primary gonarthrosis	M17.1	1.000 (0.997, 1.002)	0.8630588
VAT1	Diagnoses - main ICD10: M25.5 Pain in joint	M25.5	1.000 (0.998, 1.003)	0.752468556
VAT1	Diagnoses - main ICD10: K29.6 Other gastritis	K29.6	0.996 (0.994, 0.999)	0.013898671
VAT1	Type of cancer: ICD10: C64 Malignant neoplasm of kidney, except renal pelvis	C64	1.002 (1.000, 1.004)	0.053382211
VAT1	Diagnoses - secondary ICD10: Z80.0 Family history of malignant neoplasm of digestive organs	Z80.0	0.999 (0.994, 1.004)	0.674466844
VAT1	Diagnoses - main ICD10: G45.9 Transient cerebral ischaemic attack, unspecified	G45.9	1.001 (0.998, 1.003)	0.624678292
VAT1	Diagnoses - main ICD10: O02.1 Missed abortion	O02.1	1.001 (0.999, 1.004)	0.338493493
VAT1	Diagnoses - main ICD10: K62.8 Other specified diseases of anus and rectum	K62.8	1.001 (0.998, 1.003)	0.533098832
VAT1	Diagnoses - main ICD10: M72.0 Palmar fascial fibromatosis [Dupuytren]	M72.0	1.000 (0.997, 1.002)	0.767592322
VAT1	Diagnoses - secondary ICD10: I34.0 Mitral (valve) insufficiency	I34.0	1.000 (0.998, 1.002)	0.727450481
VAT1	Diagnoses - main ICD10: O04.9 Complete or unspecified, without complication	O04.9	1.000 (0.998, 1.002)	0.94102508
VAT1	Diagnoses - main ICD10: K22.1 Ulcer of oesophagus	K22.1	0.999 (0.996, 1.003)	0.752338473
VAT1	Diagnoses - secondary ICD10: Z86.7 Personal history of diseases of the circulatory system	Z86.7	0.999 (0.993, 1.006)	0.876288795
VAT1	Diagnoses - secondary ICD10: Y83.1 Surgical operation with implant of artificial internal device	Y83.1	0.999 (0.994, 1.003)	0.518511985
VAT1	Diagnoses - secondary ICD10: R63.4 Abnormal weight loss	R63.4	0.999 (0.995, 1.002)	0.400154773
VAT1	Diagnoses - main ICD10: K21.9 Gastro-oesophageal reflux disease without oesophagitis	K21.9	1.001 (0.996, 1.005)	0.776125838
VAT1	Diagnoses - main ICD10: N93.8 Other specified abnormal uterine and vaginal bleeding	N93.8	1.000 (0.998, 1.003)	0.939325989
VAT1	Diagnoses - main ICD10: M75.4 Impingement syndrome of shoulder	M75.4	0.998 (0.994, 1.002)	0.266174324
VAT1	Diagnoses - main ICD10: I20.9 Angina pectoris, unspecified	I20.9	0.997 (0.993, 1.002)	0.244695766
VAT1	Diagnoses - secondary ICD10: X59.9 Unspecified place	X59.9	0.999 (0.995, 1.003)	0.561282453
VAT1	Diagnoses - main ICD10: I84.1 Internal haemorrhoids with other complications	I84.1	1.001 (0.998, 1.003)	0.547899729
VAT1	Diagnoses - secondary ICD10: K57.9 Diverticular disease of intestine, part unspecified, without perforat	K57.9	1.001 (0.998, 1.004)	0.605748321
VAT1	Diagnoses - secondary ICD10: Z92.3 Personal history of irradiation	Z92.3	1.003 (1.000, 1.005)	0.018858569
VAT1	Diagnoses - main ICD10: Z09.8 Follow-up examination after other treatment for other conditions	Z09.8	0.999 (0.996, 1.002)	0.538208677
VAT1	Diagnoses - main ICD10: K42.9 Umbilical hernia without obstruction or gangrene	K42.9	0.998 (0.995, 1.001)	0.273453854
VAT1	Diagnoses - main ICD10: R07.3 Other chest pain	R07.3	0.998 (0.993, 1.004)	0.59116205
VAT1	Diagnoses - main ICD10: N92.6 Irregular menstruation, unspecified	N92.6	1.000 (0.998, 1.002)	0.9583482
VAT1	Diagnoses - main ICD10: T84.0 Mechanical complication of internal joint prosthesis	T84.0	1.000 (0.998, 1.003)	0.735104811
VAT1	Diagnoses - main ICD10: D64.9 Anaemia, unspecified	D64.9	0.998 (0.994, 1.002)	0.425514619
VAT1	Diagnoses - secondary ICD10: R00.2 Palpitations	R00.2	1.000 (0.997, 1.002)	0.692040773
VAT1	Diagnoses - secondary ICD10: Z90.4 Acquired absence of other parts of digestive tract	Z90.4	1.002 (0.998, 1.005)	0.418372877

VAT1	Type of cancer: ICD10: C54.1 Endometrium	C54.1	1.001 (0.998, 1.004)	0.421763109
VAT1	Diagnoses - main ICD10: K92.2 Gastro-intestinal haemorrhage, unspecified	K92.2	1.001 (0.998, 1.003)	0.521287739
VAT1	Diagnoses - main ICD10: L72.0 Epidermal cyst	L72.0	1.002 (0.999, 1.005)	0.242310401
VAT1	Diagnoses - secondary ICD10: M13.99 Arthritis, unspecified (Site unspecified)	M13.99	1.000 (0.997, 1.004)	0.857516385
VAT1	Diagnoses - secondary ICD10: Z95.8 Presence of other cardiac and vascular implants and grafts	Z95.8	0.999 (0.997, 1.001)	0.43040364
VAT1	Diagnoses - secondary ICD10: K29.7 Gastritis, unspecified	K29.7	1.001 (0.996, 1.006)	0.817786714
VAT1	Diagnoses - secondary ICD10: E66.9 Obesity, unspecified	E66.9	1.001 (0.996, 1.005)	0.800347617
VAT1	Diagnoses - main ICD10: M54.5 Low back pain	M54.5	0.998 (0.994, 1.001)	0.155396261
VAT1	Diagnoses - main ICD10: J18.9 Pneumonia, unspecified	J18.9	1.001 (0.998, 1.003)	0.593992639
VAT1	Type of cancer: ICD10: C44.5 Skin of trunk	C44.5	1.000 (0.997, 1.003)	0.995529525
VAT1	Diagnoses - secondary ICD10: Z95.5 Presence of coronary angioplasty implant and graft	Z95.5	0.999 (0.995, 1.003)	0.562166786
VAT1	Type of cancer: ICD10: C18.7 Sigmoid colon	C18.7	1.000 (0.998, 1.002)	0.976994172
VAT1	Diagnoses - main ICD10: R87.6 Abnormal cytological findings	R87.6	1.001 (0.998, 1.003)	0.578549199
VAT1	Diagnoses - secondary ICD10: Z86.4 Personal history of psychoactive substance abuse	Z86.4	0.997 (0.990, 1.004)	0.435448399
VAT1	Diagnoses - secondary ICD10: Z51.5 Palliative care	Z51.5	1.000 (0.998, 1.002)	0.939670917
VAT1	Diagnoses - secondary ICD10: G40.9 Epilepsy, unspecified	G40.9	1.002 (0.999, 1.005)	0.184973889
VAT1	Diagnoses - secondary ICD10: Z51.0 Radiotherapy session	Z51.0	1.000 (0.998, 1.003)	0.740149902
VAT1	Diagnoses - secondary ICD10: F10.1 Harmful use	F10.1	1.002 (1.000, 1.005)	0.097336233
VAT1	Diagnoses - secondary ICD10: I51.7 Cardiomegaly	I51.7	1.000 (0.997, 1.002)	0.909692465
VAT1	Diagnoses - main ICD10: N23 Unspecified renal colic	N23	0.999 (0.996, 1.002)	0.460501727
VAT1	Diagnoses - secondary ICD10: E86 Volume depletion	E86	1.001 (0.999, 1.003)	0.382664987
VAT1	Diagnoses - secondary ICD10: Z87.1 Personal history of diseases of the digestive system	Z87.1	1.002 (0.996, 1.009)	0.51627882
VAT1	Diagnoses - secondary ICD10: Z85.0 Personal history of malignant neoplasm of digestive organs	Z85.0	1.000 (0.996, 1.004)	0.933829218
VAT1	Diagnoses - secondary ICD10: R03.0 Elevated blood-pressure reading, without diagnosis of hypertensio	R03.0	1.000 (0.998, 1.003)	0.736527171
VAT1	Diagnoses - secondary ICD10: I25.8 Other forms of chronic ischaemic heart disease	I25.8	0.997 (0.992, 1.002)	0.206834668
VAT1	Diagnoses - secondary ICD10: Z90.1 Acquired absence of breast(s)	Z90.1	1.000 (0.997, 1.003)	0.943913826
VAT1	Diagnoses - secondary ICD10: I25.2 Old myocardial infarction	I25.2	1.001 (0.997, 1.004)	0.773766824
VAT1	Diagnoses - main ICD10: I25.1 Atherosclerotic heart disease	I25.1	0.994 (0.987, 1.001)	0.118164742
VAT1	Diagnoses - secondary ICD10: Z91.0 Personal history of allergy, other than to drugs and biological sub	Z91.0	1.000 (0.997, 1.003)	0.951793138
VAT1	Diagnoses - secondary ICD10: C78.7 Secondary malignant neoplasm of liver	C78.7	1.000 (0.997, 1.002)	0.798040724
VAT1	Diagnoses - secondary ICD10: J44.9 Chronic obstructive pulmonary disease, unspecified	J44.9	0.999 (0.995, 1.003)	0.571722913
VAT1	Diagnoses - main ICD10: G47.3 Sleep apnoea	G47.3	0.998 (0.995, 1.001)	0.294033985
VAT1	Diagnoses - secondary ICD10: N95.0 Postmenopausal bleeding	N95.0	1.001 (0.998, 1.004)	0.60520345
VAT1	Diagnoses - main ICD10: S09.9 Unspecified injury of head	S09.9	1.000 (0.997, 1.002)	0.874403876
VAT1	Diagnoses - secondary ICD10: Z95.0 Presence of cardiac pacemaker	Z95.0	1.000 (0.998, 1.002)	0.924028758
VAT1	Diagnoses - secondary ICD10: C77.3 Axillary and upper limb lymph nodes	C77.3	1.000 (0.998, 1.003)	0.741645192
VAT1	Type of cancer: ICD10: C44.4 Skin of scalp and neck	C44.4	1.001 (0.999, 1.003)	0.478173791

VAT1	Diagnoses - secondary ICD10: J45.9 Asthma, unspecified	J45.9	1.003 (0.994, 1.012)	0.565385796
VAT1	Diagnoses - main ICD10: R39.8 Other and unspecified symptoms and signs involving the urinary syster	R39.8	0.999 (0.996, 1.002)	0.364020645
VAT1	Diagnoses - main ICD10: R10.3 Pain localised to other parts of lower abdomen	R10.3	0.999 (0.995, 1.004)	0.738922063
VAT1	Diagnoses - secondary ICD10: E78.5 Hyperlipidaemia, unspecified	E78.5	1.001 (0.998, 1.005)	0.462470976
VAT1	Diagnoses - secondary ICD10: E14.9 Without complications	E14.9	0.999 (0.997, 1.002)	0.672145104
VAT1	Diagnoses - main ICD10: T81.0 Haemorrhage and haematoma complicating a procedure, not elsewhere	T81.0	0.999 (0.997, 1.002)	0.680351364
VAT1	Diagnoses - secondary ICD10: J18.1 Lobar pneumonia, unspecified	J18.1	1.001 (0.998, 1.003)	0.633046574
VAT1	Diagnoses - secondary ICD10: M81.99 Osteoporosis, unspecified (Site unspecified)	M81.99	1.003 (1.000, 1.006)	0.080704849
VAT1	Diagnoses - secondary ICD10: Y83.8 Other surgical procedures	Y83.8	0.998 (0.994, 1.003)	0.502401186
VAT1	Diagnoses - secondary ICD10: K63.5 Polyp of colon	K63.5	1.000 (0.997, 1.003)	0.964999727
VAT1	Diagnoses - main ICD10: K58.9 Irritable bowel syndrome without diarrhoea	K58.9	0.999 (0.997, 1.001)	0.335221209
VAT1	Diagnoses - secondary ICD10: K59.0 Constipation	K59.0	1.004 (1.000, 1.008)	0.05284037
VAT1	Diagnoses - secondary ICD10: Z87.3 Personal history of diseases of the musculoskeletal system and con	Z87.3	0.998 (0.996, 1.001)	0.206739257
VAT1	Type of cancer: ICD10: C56 Malignant neoplasm of ovary	C56	1.000 (0.998, 1.002)	0.988572752
VAT1	Diagnoses - main ICD10: M51.1 Lumbar and other intervertebral disk disorders with radiculopathy	M51.1	1.002 (0.999, 1.005)	0.135064714
VAT1	Diagnoses - main ICD10: I26.9 Pulmonary embolism without mention of acute cor pulmonale	I26.9	0.999 (0.996, 1.002)	0.478865699
VAT1	Diagnoses - main ICD10: N20.0 Calculus of kidney	N20.0	1.003 (1.000, 1.006)	0.094689146
VAT1	Diagnoses - main ICD10: M17.9 Gonarthrosis, unspecified	M17.9	1.002 (0.997, 1.007)	0.488902903
VAT1	Diagnoses - main ICD10: K92.0 Haematemesis	K92.0	1.000 (0.998, 1.003)	0.678412015
VAT1	Diagnoses - main ICD10: N20.1 Calculus of ureter	N20.1	0.999 (0.996, 1.002)	0.627987806
VAT1	Diagnoses - main ICD10: K13.7 Other and unspecified lesions of oral mucosa	K13.7	1.001 (0.999, 1.003)	0.404182602
VAT1	Diagnoses - main ICD10: Z42.1 Follow-up care involving plastic surgery of breast	Z42.1	0.998 (0.996, 1.001)	0.178295653
VAT1	Type of cancer: ICD10: C50.9 Breast, unspecified	C50.9	1.001 (0.996, 1.006)	0.629783745
VAT1	Diagnoses - main ICD10: J34.8 Other specified disorders of nose and nasal sinuses	J34.8	1.001 (0.998, 1.003)	0.552026513
VAT1	Type of cancer: ICD10: C44.6 Skin of upper limb, including shoulder	C44.6	1.000 (0.997, 1.002)	0.835990008
VAT1	Diagnoses - secondary ICD10: R50.9 Fever, unspecified	R50.9	0.999 (0.997, 1.002)	0.685000132
VAT1	Diagnoses - secondary ICD10: W10.0 Home	W10.0	1.000 (0.998, 1.002)	0.937413866
VAT1	Diagnoses - main ICD10: N84.1 Polyp of cervix uteri	N84.1	1.000 (0.997, 1.003)	0.937903364
VAT1	Diagnoses - secondary ICD10: R33 Retention of urine	R33	0.998 (0.995, 1.002)	0.359908318
VAT1	Diagnoses - main ICD10: D12.6 Colon, unspecified	D12.6	1.001 (0.998, 1.003)	0.72315588
VAT1	Diagnoses - main ICD10: I63.9 Cerebral infarction, unspecified	I63.9	0.999 (0.997, 1.002)	0.551947341
VAT1	Diagnoses - main ICD10: K51.9 Ulcerative colitis, unspecified	K51.9	1.002 (0.999, 1.004)	0.308929705
VAT1	Diagnoses - main ICD10: K85 Acute pancreatitis	K85	1.001 (0.998, 1.003)	0.506462124
VAT1	Diagnoses - secondary ICD10: R06.0 Dyspnoea	R06.0	0.998 (0.995, 1.001)	0.183660765
VAT1	Diagnoses - secondary ICD10: R10.4 Other and unspecified abdominal pain	R10.4	0.995 (0.991, 1.000)	0.048060732
VAT1	Diagnoses - main ICD10: Z47.0 Follow-up care involving removal of fracture plate and other internal f	Z47.0	1.000 (0.996, 1.003)	0.913669954
VAT1	Diagnoses - secondary ICD10: F10.2 Dependence syndrome	F10.2	1.001 (0.999, 1.003)	0.370193094

VAT1	Diagnoses - main ICD10: R06.5 Mouth breathing	R06.5	1.002 (0.999, 1.005)	0.125414464
VAT1	Diagnoses - main ICD10: R51 Headache	R51	0.999 (0.995, 1.004)	0.711048618
VAT1	Diagnoses - main ICD10: K62.1 Rectal polyp	K62.1	0.999 (0.995, 1.002)	0.433974911
VAT1	Diagnoses - main ICD10: M51.3 Other specified intervertebral disk degeneration	M51.3	1.000 (0.998, 1.002)	0.801823265
VAT1	Diagnoses - main ICD10: M54.56 Low back pain (Lumbar region)	M54.56	0.999 (0.996, 1.001)	0.32943856
VAT1	Diagnoses - main ICD10: M16.9 Coxarthrosis, unspecified	M16.9	0.999 (0.995, 1.003)	0.701091962
VAT1	Diagnoses - secondary ICD10: Z86.1 Personal history of infectious and parasitic diseases	Z86.1	1.000 (0.998, 1.002)	0.997027244
VAT1	Diagnoses - secondary ICD10: I12.0 Hypertensive renal disease with renal failure	I12.0	1.000 (0.998, 1.003)	0.752158184
VAT1	Diagnoses - secondary ICD10: R05 Cough	R05	1.000 (0.997, 1.002)	0.684777449
VAT1	Diagnoses - main ICD10: Z13.8 Special screening examination for other specified diseases and disorder	Z13.8	1.000 (0.997, 1.002)	0.864067092
VAT1	Diagnoses - secondary ICD10: F32.9 Depressive episode, unspecified	F32.9	1.001 (0.996, 1.006)	0.792586673
VAT1	Diagnoses - secondary ICD10: Z85.3 Personal history of malignant neoplasm of breast	Z85.3	1.001 (0.996, 1.006)	0.646175263
VAT1	Diagnoses - main ICD10: K52.9 Non-infective gastro-enteritis and colitis, unspecified	K52.9	0.995 (0.989, 1.002)	0.135858162
VAT1	Diagnoses - main ICD10: L98.9 Disorder of skin and subcutaneous tissue, unspecified	L98.9	1.001 (0.998, 1.005)	0.502476698
VAT1	Diagnoses - secondary ICD10: M19.99 Arthrosis, unspecified (Site unspecified)	M19.99	1.001 (0.997, 1.004)	0.732238611
VAT1	Diagnoses - main ICD10: Z12.1 Special screening examination for neoplasm of intestinal tract	Z12.1	1.000 (0.996, 1.003)	0.924013577
VAT1	Diagnoses - main ICD10: R07.2 Precordial pain	R07.2	1.001 (0.998, 1.004)	0.601077463
VAT1	Diagnoses - secondary ICD10: Z90.7 Acquired absence of genital organ(s)	Z90.7	0.999 (0.995, 1.003)	0.576580043
VAT1	Diagnoses - secondary ICD10: Z96.6 Presence of orthopaedic joint implants	Z96.6	1.007 (1.002, 1.012)	0.003806858
VAT1	Diagnoses - secondary ICD10: B96.8 Other specified bacterial agents as the cause of diseases classified t	B96.8	0.999 (0.995, 1.004)	0.686955305
VAT1	Diagnoses - secondary ICD10: R42 Dizziness and giddiness	R42	0.999 (0.996, 1.002)	0.412593314
VAT1	Diagnoses - secondary ICD10: N81.2 Incomplete uterovaginal prolapse	N81.2	1.002 (0.999, 1.004)	0.166526245
VAT1	Diagnoses - main ICD10: M25.56 Pain in joint (Lower leg)	M25.56	0.999 (0.996, 1.002)	0.42681442
VAT1	Diagnoses - main ICD10: D17.2 Benign lipomatous neoplasm of skin and subcutaneous tissue of limbs	D17.2	1.002 (0.999, 1.004)	0.230980321
VAT1	Diagnoses - main ICD10: O63.1 Prolonged second stage (of labour)	O63.1	1.002 (1.000, 1.004)	0.081752289
VAT1	Diagnoses - secondary ICD10: Z82.4 Family history of ischaemic heart disease and other diseases of the	Z82.4	0.995 (0.989, 1.001)	0.125321188
VAT1	Diagnoses - main ICD10: H25.0 Senile incipient cataract	H25.0	1.000 (0.998, 1.002)	0.880569132
VAT1	Diagnoses - main ICD10: D17.1 Benign lipomatous neoplasm of skin and subcutaneous tissue of trunk	D17.1	0.999 (0.996, 1.002)	0.354795822
VAT1	Diagnoses - secondary ICD10: M47.82 Other spondylosis (Cervical region)	M47.82	1.002 (1.000, 1.004)	0.031389577
VAT1	Diagnoses - secondary ICD10: Z98.0 Intestinal bypass and anastomosis status	Z98.0	1.001 (0.999, 1.004)	0.23721966
VAT1	Type of cancer: ICD10: C43.7 Malignant melanoma of lower limb, including hip	C43.7	1.000 (0.998, 1.002)	0.791397222
VAT1	Diagnoses - main ICD10: S52.50 Fracture of lower end of radius (closed)	S52.50	1.001 (0.998, 1.005)	0.411904023
VAT1	Diagnoses - secondary ICD10: W19.9 Unspecified place	W19.9	1.003 (1.000, 1.007)	0.039603409
VAT1	Diagnoses - main ICD10: M16.1 Other primary coxarthrosis	M16.1	1.000 (0.996, 1.003)	0.806350175
VAT1	Diagnoses - secondary ICD10: Z60.2 Living alone	Z60.2	1.005 (1.001, 1.009)	0.014587639
VAT1	Diagnoses - secondary ICD10: F17.1 Harmful use	F17.1	0.998 (0.996, 1.001)	0.217453924
VAT1	Diagnoses - secondary ICD10: K31.8 Other specified diseases of stomach and duodenum	K31.8	1.000 (0.997, 1.002)	0.854362392

VAT1	Diagnoses - secondary ICD10: K31.7 Polyp of stomach and duodenum	K31.7	1.001 (0.999, 1.004)	0.233231598
VAT1	Diagnoses - main ICD10: H25.1 Senile nuclear cataract	H25.1	1.001 (0.998, 1.004)	0.417158171
VAT1	Diagnoses - secondary ICD10: Y83.2 Surgical operation with anastomosis, bypass or graft	Y83.2	0.999 (0.997, 1.002)	0.573103362
VAT1	Diagnoses - secondary ICD10: D70 Agranulocytosis	D70	0.999 (0.997, 1.002)	0.624560335
VAT1	Diagnoses - main ICD10: D50.9 Iron deficiency anaemia, unspecified	D50.9	1.002 (0.999, 1.005)	0.285275234
VAT1	Diagnoses - secondary ICD10: N81.6 Rectocele	N81.6	1.001 (0.998, 1.004)	0.628434243
VAT1	Diagnoses - main ICD10: I21.9 Acute myocardial infarction, unspecified	I21.9	1.000 (0.997, 1.004)	0.827254733
VAT1	Diagnoses - main ICD10: D22.3 Melanocytic naevi of other and unspecified parts of face	D22.3	1.003 (1.000, 1.005)	0.047821646
VAT1	Diagnoses - secondary ICD10: K44.9 Diaphragmatic hernia without obstruction or gangrene	K44.9	0.998 (0.990, 1.006)	0.626698783
VAT1	Diagnoses - main ICD10: B34.9 Viral infection, unspecified	B34.9	0.998 (0.996, 1.000)	0.067579773
VAT1	Diagnoses - main ICD10: N32.8 Other specified disorders of bladder	N32.8	0.999 (0.996, 1.002)	0.470525822
VAT1	Diagnoses - main ICD10: J34.2 Deviated nasal septum	J34.2	0.998 (0.995, 1.002)	0.381848631
VAT1	Diagnoses - main ICD10: N50.8 Other specified disorders of male genital organs	N50.8	1.002 (0.999, 1.004)	0.176772934
VAT1	Diagnoses - secondary ICD10: Z86.0 Personal history of other neoplasms	Z86.0	1.002 (0.998, 1.006)	0.403436869
VAT1	Diagnoses - secondary ICD10: Z72.1 Alcohol use	Z72.1	1.002 (0.998, 1.006)	0.280211861
VAT1	Diagnoses - main ICD10: G56.0 Carpal tunnel syndrome	G56.0	0.997 (0.992, 1.003)	0.406572935
VAT1	Diagnoses - main ICD10: I25.9 Chronic ischaemic heart disease, unspecified	I25.9	1.000 (0.998, 1.002)	0.94035985
VAT1	Diagnoses - secondary ICD10: Z37.0 Single live birth	Z37.0	0.990 (0.983, 0.997)	0.006059419
VAT1	Diagnoses - secondary ICD10: E03.9 Hypothyroidism, unspecified	E03.9	1.000 (0.993, 1.006)	0.884710281
VAT1	Diagnoses - secondary ICD10: Z88.6 Personal history of allergy to analgesic agent	Z88.6	1.000 (0.997, 1.004)	0.873136777
VAT1	Diagnoses - main ICD10: M20.2 Hallux rigidus	M20.2	1.000 (0.998, 1.002)	0.927743529
VAT1	Diagnoses - secondary ICD10: B95.6 Staphylococcus aureus as the cause of diseases classified to other c	B95.6	1.000 (0.998, 1.003)	0.83228793
VAT1	Diagnoses - main ICD10: R04.2 Haemoptysis	R04.2	1.000 (0.997, 1.002)	0.774041368
VAT1	Diagnoses - main ICD10: I21.0 Acute transmural myocardial infarction of anterior wall	I21.0	1.000 (0.997, 1.002)	0.80448364
VAT1	Diagnoses - secondary ICD10: R94.5 Abnormal results of liver function studies	R94.5	1.002 (0.999, 1.004)	0.280328181
VAT1	Diagnoses - secondary ICD10: T81.4 Infection following a procedure, not elsewhere classified	T81.4	0.999 (0.996, 1.002)	0.431154562
VAT1	Diagnoses - secondary ICD10: Z88.8 Personal history of allergy to other drugs, medicaments and biolo	Z88.8	0.998 (0.995, 1.001)	0.235876153
VAT1	Diagnoses - main ICD10: H00.1 Chalazion	H00.1	1.002 (1.000, 1.005)	0.107371961
VAT1	Type of cancer: ICD10: D05.1 Intraductal carcinoma in situ	D05.1	0.999 (0.996, 1.002)	0.529626822
VAT1	Diagnoses - secondary ICD10: Y43.3 Other antineoplastic drugs	Y43.3	0.998 (0.996, 1.000)	0.097658172
VAT1	Diagnoses - main ICD10: K02.9 Dental caries, unspecified	K02.9	0.999 (0.996, 1.002)	0.59280873
VAT1	Diagnoses - main ICD10: M72.04 Palmar fascial fibromatosis [Dupuytren]-Hand	M72.04	1.000 (0.998, 1.003)	0.737410449
VAT1	Diagnoses - secondary ICD10: N83.2 Other and unspecified ovarian cysts	N83.2	0.999 (0.996, 1.001)	0.385298463
VAT1	Diagnoses - main ICD10: M75.0 Adhesive capsulitis of shoulder	M75.0	1.001 (0.998, 1.003)	0.554676284
VAT1	Diagnoses - secondary ICD10: I73.9 Peripheral vascular disease, unspecified	I73.9	0.999 (0.996, 1.001)	0.363214955
VAT1	Diagnoses - secondary ICD10: N17.9 Acute renal failure, unspecified	N17.9	1.001 (0.998, 1.003)	0.688322461
VAT1	Diagnoses - main ICD10: M75.1 Rotator cuff syndrome	M75.1	0.998 (0.995, 1.001)	0.171299504

VAT1	Diagnoses - main ICD10: L82 Seborrhoeic keratosis	L82	0.999 (0.996, 1.002)	0.436586816
VAT1	Diagnoses - main ICD10: K61.0 Anal abscess	K61.0	0.999 (0.997, 1.002)	0.582396943
VAT1	Diagnoses - secondary ICD10: Z53.2 Procedure not carried out because of patient's decision for other a	Z53.2	0.999 (0.997, 1.002)	0.662260668
VAT1	Diagnoses - secondary ICD10: Z72.0 Tobacco use	Z72.0	0.999 (0.991, 1.006)	0.740249409
VAT1	Diagnoses - main ICD10: I21.1 Acute transmural myocardial infarction of inferior wall	I21.1	1.001 (0.999, 1.004)	0.312500805
VAT1	Diagnoses - secondary ICD10: N39.3 Stress incontinence	N39.3	0.999 (0.997, 1.002)	0.562499536
VAT1	Diagnoses - main ICD10: K40.9 Unilateral or unspecified inguinal hernia, without obstruction or gangr	K40.9	1.000 (0.993, 1.007)	0.985934534
VAT1	Diagnoses - main ICD10: C67.9 Bladder, unspecified	C67.9	1.002 (0.999, 1.004)	0.206943654
VAT1	Diagnoses - secondary ICD10: Z13.0 Special screening examination for diseases of the blood and blood	Z13.0	0.999 (0.997, 1.001)	0.36324354
VAT1	Diagnoses - secondary ICD10: Z86.6 Personal history of diseases of the nervous system and sense orga	Z86.6	1.003 (0.999, 1.006)	0.160425033
VAT1	Diagnoses - main ICD10: K92.1 Melaena	K92.1	0.999 (0.996, 1.001)	0.294749466
VAT1	Diagnoses - secondary ICD10: J34.3 Hypertrophy of nasal turbinates	J34.3	0.999 (0.997, 1.001)	0.318069097
VAT1	Diagnoses - secondary ICD10: Z88.0 Personal history of allergy to penicillin	Z88.0	1.000 (0.993, 1.007)	0.89956213
VAT1	Diagnoses - main ICD10: S82.80 Fractures of other parts of lower leg (closed)	S82.80	1.002 (1.000, 1.005)	0.082487513
VAT1	Diagnoses - main ICD10: M20.1 Hallux valgus (acquired)	M20.1	1.004 (0.999, 1.009)	0.083317803
VAT1	Diagnoses - secondary ICD10: N39.0 Urinary tract infection, site not specified	N39.0	1.003 (0.998, 1.007)	0.237488697
VAT1	Diagnoses - secondary ICD10: Z03.4 Observation for suspected myocardial infarction	Z03.4	1.001 (0.998, 1.004)	0.602165364
VAT1	Diagnoses - main ICD10: O80.0 Spontaneous vertex delivery	O80.0	1.001 (0.998, 1.004)	0.575514446
VAT1	Diagnoses - secondary ICD10: N73.6 Female pelvic peritoneal adhesions	N73.6	0.999 (0.996, 1.002)	0.467002467
VAT1	Diagnoses - secondary ICD10: D25.9 Leiomyoma of uterus, unspecified	D25.9	0.999 (0.996, 1.003)	0.650902177
VAT1	Diagnoses - secondary ICD10: K26.9 Unspecified as acute or chronic, without haemorrhage or perforat	K26.9	1.000 (0.998, 1.002)	0.930416105
VAT1	Diagnoses - main ICD10: K43.9 Ventral hernia without obstruction or gangrene	K43.9	0.999 (0.996, 1.002)	0.671533879
VAT1	Diagnoses - main ICD10: K35.9 Acute appendicitis, unspecified	K35.9	1.000 (0.998, 1.003)	0.802758349
VAT1	Type of cancer: ICD10: C44.7 Skin of lower limb, including hip	C44.7	1.001 (0.999, 1.003)	0.326442639
VAT1	Diagnoses - secondary ICD10: M15.9 Polyarthrosis, unspecified	M15.9	1.000 (0.998, 1.003)	0.698740852
VAT1	Diagnoses - main ICD10: R13 Dysphagia	R13	0.999 (0.996, 1.003)	0.7204349
VAT1	Diagnoses - main ICD10: M23.23 Derangement of meniscus due to old tear or injury (Medial collateral	M23.23	0.999 (0.995, 1.002)	0.456952637
VAT1	Diagnoses - secondary ICD10: Z51.2 Other chemotherapy	Z51.2	0.999 (0.996, 1.003)	0.748995452
VAT1	Diagnoses - main ICD10: J33.9 Nasal polyp, unspecified	J33.9	0.999 (0.997, 1.002)	0.612789485
VAT1	Diagnoses - secondary ICD10: Z85.5 Personal history of malignant neoplasm of urinary tract	Z85.5	1.002 (0.999, 1.005)	0.168563323
VAT1	Diagnoses - secondary ICD10: Z92.1 Personal history of long-term (current) use of anticoagulants	Z92.1	0.999 (0.995, 1.004)	0.823210238
VAT1	Diagnoses - main ICD10: Z30.2 Sterilisation	Z30.2	0.998 (0.993, 1.004)	0.559609712
VAT1	Diagnoses - main ICD10: N63 Unspecified lump in breast	N63	0.999 (0.996, 1.001)	0.290640043
VAT1	Diagnoses - main ICD10: I84.8 Unspecified haemorrhoids with other complications	I84.8	0.999 (0.996, 1.003)	0.719735736
VAT1	Diagnoses - main ICD10: Z46.6 Fitting and adjustment of urinary device	Z46.6	1.003 (1.000, 1.007)	0.050046745
VAT1	Diagnoses - main ICD10: K01.1 Impacted teeth	K01.1	1.001 (0.998, 1.003)	0.55628045
VAT1	Diagnoses - secondary ICD10: Z80.3 Family history of malignant neoplasm of breast	Z80.3	0.999 (0.997, 1.001)	0.310708136

VAT1	Diagnoses - secondary ICD10: Y83.6 Removal of other organ (partial) (total)	Y83.6	1.002 (0.998, 1.006)	0.302600855
VAT1	Diagnoses - main ICD10: M65.3 Trigger finger	M65.3	0.997 (0.995, 0.999)	0.01238506
VAT1	Diagnoses - secondary ICD10: Z92.4 Personal history of major surgery, not elsewhere classified	Z92.4	1.000 (0.998, 1.002)	0.961965108
VAT1	Diagnoses - main ICD10: M23.2 Derangement of meniscus due to old tear or injury	M23.2	1.000 (0.997, 1.002)	0.938824222
VAT1	Diagnoses - main ICD10: K80.5 Calculus of bile duct without cholangitis or cholecystitis	K80.5	1.001 (0.998, 1.004)	0.539982588
VAT1	Type of cancer: ICD10: C44.9 Malignant neoplasm of skin, unspecified	C44.9	1.004 (1.000, 1.008)	0.052184618
VAT1	Diagnoses - main ICD10: D24 Benign neoplasm of breast	D24	1.001 (0.999, 1.003)	0.435560285
VAT1	Diagnoses - secondary ICD10: N85.8 Other specified noninflammatory disorders of uterus	N85.8	0.998 (0.996, 1.001)	0.201748612
VAT1	Diagnoses - secondary ICD10: I95.9 Hypotension, unspecified	I95.9	0.998 (0.996, 1.001)	0.207881761
VAT1	Diagnoses - secondary ICD10: B96.2 Escherichia coli [E. coli] as the cause of diseases classified to other	B96.2	1.000 (0.998, 1.003)	0.767508743
VAT1	Diagnoses - main ICD10: K80.1 Calculus of gallbladder with other cholecystitis	K80.1	1.002 (0.997, 1.006)	0.443696797
VAT1	Diagnoses - secondary ICD10: Z53.0 Procedure not carried out because of contraindication	Z53.0	0.999 (0.995, 1.004)	0.738930593
VAT1	Diagnoses - secondary ICD10: Z53.8 Procedure not carried out for other reasons	Z53.8	1.001 (0.995, 1.008)	0.691677665
VAT1	Diagnoses - main ICD10: N35.9 Urethral stricture, unspecified	N35.9	1.000 (0.997, 1.003)	0.913141629
VAT1	Type of cancer: ICD10: D06.9 Cervix, unspecified	D06.9	1.000 (0.997, 1.004)	0.835559974
VAT1	Diagnoses - secondary ICD10: Z53.9 Procedure not carried out, unspecified reason	Z53.9	0.999 (0.996, 1.002)	0.633124439
VAT1	Diagnoses - main ICD10: D12.8 Rectum	D12.8	1.000 (0.998, 1.003)	0.83056159
VAT1	Diagnoses - secondary ICD10: M19.9 Arthrosis, unspecified	M19.9	1.000 (0.997, 1.003)	0.840006024
VAT1	Diagnoses - main ICD10: H02.8 Other specified disorders of eyelid	H02.8	1.002 (1.000, 1.005)	0.095454786
VAT1	Diagnoses - main ICD10: N92.1 Excessive and frequent menstruation with irregular cycle	N92.1	0.998 (0.995, 1.001)	0.288116829
VAT1	Diagnoses - main ICD10: O70.0 First degree perineal laceration during delivery	O70.0	0.998 (0.996, 1.001)	0.17157295
VAT1	Diagnoses - secondary ICD10: N80.0 Endometriosis of uterus	N80.0	1.001 (0.998, 1.003)	0.583039075
VAT1	Diagnoses - main ICD10: M23.22 Derangement of meniscus due to old tear or injury (Posterior cruciate	M23.22	1.001 (0.998, 1.004)	0.603290243
VAT1	Diagnoses - secondary ICD10: E11.9 Without complications	E11.9	0.999 (0.992, 1.006)	0.772045761
VAT1	Diagnoses - main ICD10: M79.66 Pain in limb (Lower leg)	M79.66	0.998 (0.996, 1.001)	0.205198742
VAT1	Diagnoses - main ICD10: O26.8 Other specified pregnancy-related conditions	O26.8	0.999 (0.996, 1.001)	0.234302636
VAT1	Diagnoses - secondary ICD10: Z88.1 Personal history of allergy to other antibiotic agents	Z88.1	1.000 (0.997, 1.003)	0.948895142
VAT1	Diagnoses - secondary ICD10: J90 Pleural effusion, not elsewhere classified	J90	1.002 (0.999, 1.005)	0.242733629
VAT1	Underlying (primary) cause of death: ICD10: C34.9 Bronchus or lung, unspecified	C34.9	0.995 (0.897, 1.103)	0.91978759
VAT1	Diagnoses - main ICD10: A41 Other septicaemia	A41	1.004 (1.000, 1.007)	0.028235511
VAT1	Diagnoses - main ICD10: C18 Malignant neoplasm of colon	C18	0.998 (0.993, 1.002)	0.260265146
VAT1	Diagnoses - main ICD10: C34 Malignant neoplasm of bronchus and lung	C34	1.000 (0.997, 1.004)	0.957379986
VAT1	Diagnoses - main ICD10: C43 Malignant melanoma of skin	C43	1.000 (0.996, 1.004)	0.980304078
VAT1	Diagnoses - main ICD10: C67 Malignant neoplasm of bladder	C67	1.000 (0.997, 1.004)	0.919349639
VAT1	Diagnoses - main ICD10: C78 Secondary malignant neoplasm of respiratory and digestive organs	C78	1.001 (0.998, 1.004)	0.533265927
VAT1	Diagnoses - main ICD10: C79 Secondary malignant neoplasm of other sites	C79	1.003 (1.000, 1.006)	0.071929125
VAT1	Diagnoses - main ICD10: D05 Carcinoma in situ of breast	D05	0.995 (0.992, 0.999)	0.0042791

VAT1	Diagnoses - main ICD10: D17 Benign lipomatous neoplasm	D17	1.003 (0.997, 1.009)	0.316671969
VAT1	Diagnoses - main ICD10: D22 Melanocytic naevi	D22	1.003 (0.998, 1.008)	0.227826814
VAT1	Diagnoses - main ICD10: D23 Other benign neoplasms of skin	D23	1.001 (0.996, 1.006)	0.624526737
VAT1	Diagnoses - main ICD10: D50 Iron deficiency anaemia	D50	1.000 (0.995, 1.005)	0.993625584
VAT1	Diagnoses - main ICD10: D64 Other anaemias	D64	1.000 (0.994, 1.005)	0.87333447
VAT1	Diagnoses - main ICD10: G43 Migraine	G43	0.997 (0.994, 1.000)	0.084576398
VAT1	Diagnoses - main ICD10: G45 Transient cerebral ischaemic attacks and related syndromes	G45	1.000 (0.997, 1.004)	0.849139904
VAT1	Diagnoses - main ICD10: G57 Mononeuropathies of lower limb	G57	1.000 (0.997, 1.003)	0.909732942
VAT1	Diagnoses - main ICD10: H00 Hordeolum and chalazion	H00	1.003 (1.000, 1.007)	0.046354577
VAT1	Diagnoses - main ICD10: H02 Other disorders of eyelid	H02	1.001 (0.995, 1.006)	0.851798619
VAT1	Diagnoses - main ICD10: H04 Disorders of lachrymal system	H04	0.997 (0.994, 1.000)	0.070975939
VAT1	Diagnoses - main ICD10: H33 Retinal detachments and breaks	H33	1.002 (0.997, 1.006)	0.407851811
VAT1	Diagnoses - main ICD10: H35 Other retinal disorders	H35	1.000 (0.997, 1.004)	0.793588993
VAT1	Diagnoses - main ICD10: H40 Glaucoma	H40	1.001 (0.997, 1.004)	0.618793773
VAT1	Diagnoses - main ICD10: I26 Pulmonary embolism	I26	1.001 (0.997, 1.005)	0.753251988
VAT1	Diagnoses - main ICD10: I47 Paroxysmal tachycardia	I47	0.997 (0.994, 1.001)	0.134691641
VAT1	Diagnoses - main ICD10: I50 Heart failure	I50	0.998 (0.995, 1.001)	0.172844138
VAT1	Diagnoses - main ICD10: I63 Cerebral infarction	I63	1.000 (0.996, 1.004)	0.964941979
VAT1	Diagnoses - main ICD10: J18 Pneumonia, organism unspecified	J18	1.000 (0.994, 1.007)	0.912203411
VAT1	Diagnoses - main ICD10: J32 Chronic sinusitis	J32	1.000 (0.998, 1.003)	0.754053738
VAT1	Diagnoses - main ICD10: J38 Diseases of vocal cords and larynx, not elsewhere classified	J38	1.000 (0.997, 1.003)	0.767782385
VAT1	Diagnoses - main ICD10: J45 Asthma	J45	1.002 (0.999, 1.006)	0.221489794
VAT1	Diagnoses - main ICD10: K01 Embedded and impacted teeth	K01	1.001 (0.998, 1.005)	0.367409058
VAT1	Diagnoses - main ICD10: K02 Dental caries	K02	0.998 (0.994, 1.002)	0.244898378
VAT1	Diagnoses - main ICD10: K04 Diseases of pulp and periapical tissues	K04	1.000 (0.997, 1.004)	0.889210268
VAT1	Diagnoses - main ICD10: K08 Other disorders of teeth and supporting structures	K08	1.001 (0.997, 1.004)	0.699575602
VAT1	Diagnoses - main ICD10: K13 Other diseases of lip and oral mucosa	K13	1.001 (0.997, 1.005)	0.798571059
VAT1	Diagnoses - main ICD10: K25 Gastric ulcer	K25	1.003 (0.999, 1.006)	0.183507286
VAT1	Diagnoses - main ICD10: K26 Duodenal ulcer	K26	1.003 (1.000, 1.006)	0.078049483
VAT1	Diagnoses - main ICD10: K31 Other diseases of stomach and duodenum	K31	1.000 (0.996, 1.005)	0.866534811
VAT1	Diagnoses - main ICD10: K42 Umbilical hernia	K42	0.999 (0.995, 1.004)	0.759538817
VAT1	Diagnoses - main ICD10: K56 Paralytic ileus and intestinal obstruction without hernia	K56	1.001 (0.997, 1.005)	0.59599044
VAT1	Diagnoses - main ICD10: K58 Irritable bowel syndrome	K58	0.998 (0.996, 1.001)	0.288317208
VAT1	Diagnoses - main ICD10: K59 Other functional intestinal disorders	K59	1.000 (0.995, 1.006)	0.942397081
VAT1	Diagnoses - main ICD10: K61 Abscess of anal and rectal regions	K61	1.000 (0.998, 1.003)	0.775730672
VAT1	Diagnoses - main ICD10: K63 Other diseases of intestine	K63	1.008 (1.001, 1.016)	0.035749661
VAT1	Diagnoses - main ICD10: K81 Cholecystitis	K81	1.000 (0.996, 1.004)	0.939237737

VAT1	Diagnoses - main ICD10: K92 Other diseases of digestive system	K92	1.000 (0.994, 1.007)	0.884185085
VAT1	Diagnoses - main ICD10: L02 Cutaneous abscess, furuncle and carbuncle	L02	1.000 (0.996, 1.003)	0.946592829
VAT1	Diagnoses - main ICD10: L57 Skin changes due to chronic exposure to nonionising radiation	L57	1.000 (0.997, 1.004)	0.837284742
VAT1	Diagnoses - main ICD10: L72 Follicular cysts of skin and subcutaneous tissue	L72	1.004 (0.997, 1.011)	0.24390433
VAT1	Diagnoses - main ICD10: L90 Atrophic disorders of skin	L90	1.004 (1.001, 1.008)	0.018299518
VAT1	Diagnoses - main ICD10: L98 Other disorders of skin and subcutaneous tissue, not elsewhere classified	L98	0.999 (0.994, 1.005)	0.762987874
VAT1	Diagnoses - main ICD10: M06 Other rheumatoid arthritis	M06	1.000 (0.997, 1.004)	0.882237803
VAT1	Diagnoses - main ICD10: M13 Other arthritis	M13	1.001 (0.998, 1.004)	0.427259866
VAT1	Diagnoses - main ICD10: M15 Polyarthrosis	M15	0.998 (0.995, 1.001)	0.29756225
VAT1	Diagnoses - main ICD10: M19 Other arthrosis	M19	1.003 (0.998, 1.009)	0.221841841
VAT1	Diagnoses - main ICD10: M47 Spondylosis	M47	0.998 (0.994, 1.002)	0.433226113
VAT1	Diagnoses - main ICD10: M48 Other spondylopathies	M48	1.000 (0.996, 1.004)	0.92118277
VAT1	Diagnoses - main ICD10: M51 Other intervertebral disk disorders	M51	1.003 (0.997, 1.009)	0.261046062
VAT1	Diagnoses - main ICD10: M65 Synovitis and tenosynovitis	M65	0.998 (0.993, 1.002)	0.331231708
VAT1	Diagnoses - main ICD10: M75 Shoulder lesions	M75	0.994 (0.987, 1.001)	0.11806531
VAT1	Diagnoses - main ICD10: M79 Other soft tissue disorders, not elsewhere classified	M79	1.000 (0.993, 1.007)	0.996708425
VAT1	Diagnoses - main ICD10: M84 Disorders of continuity of bone	M84	1.000 (0.997, 1.003)	0.889147495
VAT1	Diagnoses - main ICD10: N13 Obstructive and reflux uropathy	N13	1.002 (0.998, 1.005)	0.359056674
VAT1	Diagnoses - main ICD10: N30 Cystitis	N30	1.002 (0.998, 1.005)	0.365740797
VAT1	Diagnoses - main ICD10: N31 Neuromuscular dysfunction of bladder, not elsewhere classified	N31	1.001 (0.999, 1.004)	0.34644045
VAT1	Diagnoses - main ICD10: N35 Urethral stricture	N35	1.001 (0.997, 1.005)	0.523567752
VAT1	Diagnoses - main ICD10: N39 Other disorders of urinary system	N39	0.993 (0.984, 1.001)	0.101753122
VAT1	Diagnoses - main ICD10: N48 Other disorders of penis	N48	1.000 (0.997, 1.003)	0.841055353
VAT1	Diagnoses - main ICD10: N50 Other disorders of male genital organs	N50	1.002 (0.998, 1.005)	0.326284301
VAT1	Diagnoses - main ICD10: N60 Benign mammary dysplasia	N60	0.999 (0.996, 1.002)	0.566221758
VAT1	Diagnoses - main ICD10: N80 Endometriosis	N80	0.999 (0.996, 1.002)	0.584479396
VAT1	Diagnoses - main ICD10: N83 Noninflammatory disorders of ovary, Fallopian tube and broad ligament	N83	0.995 (0.991, 0.999)	0.014607915
VAT1	Diagnoses - main ICD10: N84 Polyp of female genital tract	N84	1.001 (0.994, 1.008)	0.690074452
VAT1	Diagnoses - main ICD10: N85 Other noninflammatory disorders of uterus, except cervix	N85	0.998 (0.995, 1.001)	0.228532145
VAT1	Diagnoses - main ICD10: N87 Dysplasia of cervix uteri	N87	1.001 (0.998, 1.004)	0.411863796
VAT1	Diagnoses - main ICD10: N90 Other noninflammatory disorders of vulva and perineum	N90	0.996 (0.993, 0.998)	0.002475947
VAT1	Diagnoses - main ICD10: N93 Other abnormal uterine and vaginal bleeding	N93	1.001 (0.997, 1.005)	0.692145036
VAT1	Diagnoses - main ICD10: N94 Pain and other conditions associated with female genital organs and mens	N94	0.998 (0.995, 1.001)	0.120834065
VAT1	Diagnoses - main ICD10: N95 Menopausal and other perimenopausal disorders	N95	1.003 (0.996, 1.009)	0.428360719
VAT1	Diagnoses - main ICD10: O02 Other abnormal products of conception	O02	1.001 (0.998, 1.004)	0.408993564
VAT1	Diagnoses - main ICD10: O03 Spontaneous abortion	O03	1.001 (0.998, 1.003)	0.726743661
VAT1	Diagnoses - main ICD10: O26 Maternal care for other conditions predominantly related to pregnancy	O26	0.997 (0.994, 1.000)	0.046902559

VAT1	Diagnoses - main ICD10: O36 Maternal care for other known or suspected foetal problems	O36	0.997 (0.995, 1.000)	0.067074258
VAT1	Diagnoses - main ICD10: O63 Long labour	O63	1.001 (0.998, 1.003)	0.580991162
VAT1	Diagnoses - main ICD10: O68 Labour and delivery complicated by foetal stress [distress]	O68	0.997 (0.993, 1.000)	0.086428071
VAT1	Diagnoses - main ICD10: O70 Perineal laceration during delivery	O70	0.996 (0.991, 1.000)	0.05591815
VAT1	Diagnoses - main ICD10: O80 Single spontaneous delivery	O80	1.000 (0.997, 1.004)	0.859317756
VAT1	Diagnoses - main ICD10: R00 Abnormalities of heart beat	R00	0.997 (0.993, 1.002)	0.228332898
VAT1	Diagnoses - main ICD10: R06 Abnormalities of breathing	R06	0.999 (0.993, 1.004)	0.679683839
VAT1	Diagnoses - main ICD10: R19 Other symptoms and signs involving the digestive system and abdomen	R19	1.002 (0.994, 1.010)	0.664572745
VAT1	Diagnoses - main ICD10: R22 Localised swelling, mass and lump of skin and subcutaneous tissue	R22	0.997 (0.993, 1.000)	0.031628694
VAT1	Diagnoses - main ICD10: R39 Other symptoms and signs involving the urinary system	R39	0.998 (0.994, 1.003)	0.502902251
VAT1	Diagnoses - main ICD10: R50 Fever of unknown origin	R50	1.005 (1.002, 1.008)	0.001773055
VAT1	Diagnoses - main ICD10: R56 Convulsions, not elsewhere classified	R56	1.000 (0.997, 1.003)	0.895654773
VAT1	Diagnoses - main ICD10: R59 Enlarged lymph nodes	R59	1.002 (1.000, 1.005)	0.076631722
VAT1	Diagnoses - main ICD10: R63 Symptoms and signs concerning food and fluid intake	R63	1.002 (0.999, 1.005)	0.154864764
VAT1	Diagnoses - main ICD10: R79 Other abnormal findings of blood chemistry	R79	0.997 (0.993, 1.002)	0.225294137
VAT1	Diagnoses - main ICD10: R87 Abnormal findings in specimens from female genital organs	R87	1.000 (0.997, 1.003)	0.922823353
VAT1	Diagnoses - main ICD10: R93 Abnormal findings on diagnostic imaging of other body structures	R93	1.000 (0.997, 1.003)	0.764348178
VAT1	Diagnoses - main ICD10: S01 Open wound of head	S01	1.003 (1.000, 1.007)	0.091297124
VAT1	Diagnoses - main ICD10: S02 Fracture of skull and facial bones	S02	1.001 (0.998, 1.004)	0.542180096
VAT1	Diagnoses - main ICD10: S42 Fracture of shoulder and upper arm	S42	1.000 (0.996, 1.003)	0.818981562
VAT1	Diagnoses - main ICD10: S61 Open wound of wrist and hand	S61	1.000 (0.996, 1.003)	0.906789341
VAT1	Diagnoses - main ICD10: S62 Fracture at wrist and hand level	S62	1.000 (0.996, 1.003)	0.9021347
VAT1	Diagnoses - main ICD10: S72 Fracture of femur	S72	0.998 (0.994, 1.001)	0.216781609
VAT1	Diagnoses - main ICD10: S82 Fracture of lower leg, including ankle	S82	1.003 (0.997, 1.009)	0.299529111
VAT1	Diagnoses - main ICD10: T39 Poisoning by nonopioid analgesics, antipyretics and antirheumatics	T39	0.999 (0.996, 1.002)	0.681964609
VAT1	Diagnoses - main ICD10: T81 Complications of procedures, not elsewhere classified	T81	1.004 (0.998, 1.011)	0.195897663
VAT1	Diagnoses - main ICD10: T82 Complications of cardiac and vascular prosthetic devices, implants and gr	T82	1.003 (1.000, 1.006)	0.086904834
VAT1	Diagnoses - main ICD10: T85 Complications of other internal prosthetic devices, implants and grafts	T85	0.999 (0.996, 1.002)	0.544165208
VAT1	Diagnoses - main ICD10: Z01 Other special examinations and investigations of persons without complai	Z01	0.999 (0.996, 1.002)	0.652872473
VAT1	Diagnoses - main ICD10: Z03 Medical observation and evaluation for suspected diseases and conditions	Z03	1.001 (0.995, 1.007)	0.844087508
VAT1	Diagnoses - main ICD10: Z08 Follow-up examination after treatment for malignant neoplasm	Z08	0.999 (0.993, 1.004)	0.615833164
VAT1	Diagnoses - main ICD10: Z12 Special screening examination for neoplasms	Z12	1.001 (0.995, 1.006)	0.758778983
VAT1	Diagnoses - main ICD10: Z13 Special screening examination for other diseases and disorders	Z13	1.001 (0.997, 1.005)	0.794746312
VAT1	Diagnoses - main ICD10: Z30 Contraceptive management	Z30	1.002 (0.995, 1.008)	0.594435723
VAT1	Diagnoses - main ICD10: Z42 Follow-up care involving plastic surgery	Z42	1.001 (0.997, 1.004)	0.775033956
VAT1	Diagnoses - main ICD10: Z43 Attention to artificial openings	Z43	1.002 (0.998, 1.005)	0.312193832
VAT1	Diagnoses - main ICD10: Z45 Adjustment and management of implanted device	Z45	1.006 (1.002, 1.010)	0.004471888

VAT1	Diagnoses - main ICD10: Z46 Fitting and adjustment of other devices	Z46	1.003 (0.998, 1.008)	0.262778365
VAT1	Diagnoses - main ICD10: Z53 Persons encountering health services for specifie procedures, not carried	Z53	0.999 (0.996, 1.003)	0.649700132
